# Nutritional evaluation, characteristic component differences and non-targeted metabolomics analysis of traditional fermented glutinous Rice in four Chinese provinces

**DOI:** 10.1016/j.fochx.2026.103534

**Published:** 2026-01-13

**Authors:** Yu Pan, Keyun Lin, Jinxing Zeng, Yaya Zhou, Diqin Yang, Xia Zhu, Minghui Shi, Quanmin Sun, Chengzhang Ou, Yu Wang, Yudan Xie, Shuying Yang, Kuan Lu

**Affiliations:** a Guizhou Biotechnology Research and Development Base Co., Ltd., Guiyang 550014, PR China.; b Guizhou Kehui Inspection and Testing Research Institute Co., Ltd., Guiyang 550014, PR China.

**Keywords:** Fermented glutinous rice, Nutritional evaluation, Traditional fermented foods, Regional comparison, Metabolomics, Volatile compounds, Antioxidant capacity

## Abstract

This study aimed to compare the nutritional characteristics, antioxidant activity, and flavor differences of traditional fermented glutinous rice from four regions of China, and to elucidate the underlying metabolic mechanisms using metabolomics. Physicochemical assays, HPLC, GC–MS, and LC-MS analyzed five samples (HB_MPP, SC, JS, GZ_LYM, SWJD). Significant differences in antioxidant capacity and amino acids were found, with lysine as the first limiting amino acid. Dominant organic acids were lactic, malic, and fumaric acids. Forty volatile compounds (mainly esters, alcohols, acids) were detected, with phenylethanol, ethyl hexadecanoate, and ethyl decanoate as key flavor markers (VIP ≥ 1). Metabolomics identified 3027 metabolites, dominated by lipids (27.59%) and organic acids (26.99%). Key pathways included plant secondary metabolite biosynthesis and amino acid metabolism. This first multi-omics comparison highlights regional characteristic bases, aiding traditional fermented food quality evaluation and industrial application.

## Highlights

• Multi-technique analysis identifies distinct regional differences in 5 Chinese fermented glutinous rice.
• Lysine (first limiting amino acid), phenylethanol (VIP = 2.5380) and lactic/malic/fumaric acids are key markers.
• Untargeted metabolomics identifies 3027 metabolites and 3 core differential pathways.
• SWJD exhibits highest antioxidant activity via phenolics, guiding functional raw material optimization.
• This work clarifies fermented glutinous rice regional basis, supporting its quality evaluation and industrial upgrading.

## 1 Introduction

Fermented glutinous rice, also known as sweet rice wine, is a traditional semi-solid fermented food in China (Lv et al., 2015). It is primarily made from glutinous rice and produced through natural fermentation with the aid of fermentation starters (commonly known as qu) (Wang et al., 2025; Yu et al., 2019)**.** During fermentation, starch is enzymatically hydrolyzed, resulting in an increased content of low-molecular-weight polysaccharides primarily composed of glucose. These sugars serve as essential nutrients for fermentative microorganisms, which further metabolize them into various compounds such as higher alcohols, esters, alcohols, aldehydes, and organic acids. These metabolites contribute to the wine's characteristic mellow sweetness, soft texture, and unique aroma (Qian et al., 2023) the raw materials and preparation techniques appear relatively simple, the fermentation process involves complex interactions among diverse microorganisms. These microbial interactions not only shape the flavor profile but also determine the formation of nutritional and functional components. As a culturally important component of the Chinese diet, fermented glutinous rice is also notable for its nutritional value. Beyond the intrinsic nutrients of the raw materials, traditional brewing processes generate a range of bioactive substances including peptides,sugars (Shen et al., 2011), proteins, vitamins, minerals, and free amino acids (Cai et al., 2018; Chen et al., 2021; Liang et al., 2020), which are easily absorbed and utilized by the human body. These components are associated with various health benefits such as promoting digestion, warming the stomach and spleen, enhancing immunity, and relieving fatigue (Kumari et al., 2022; Sunil et al., 2024), factors such as regional climate, raw material types, aging duration, microbial communities, and fermentation techniques can lead to substantial variation in flavor characteristics, nutritional composition, and safety among fermented glutinous rice produced in different provinces (Peng et al., 2023). Although these regional variations are widely recognized, they have not yet been systematically characterized at the metabolic level.

Despite recent progress in studying traditional fermented foods, comprehensive analyses of nutrients and functional metabolites in fermented glutinous rice across China-especially their metabolic networks-are still scarce. Untargeted metabolomics, as a powerful approach for comprehensive profiling of small-molecule metabolites, has shown great potential in elucidating the flavor characteristics of fermented glutinous rice (Deng et al., 2025; Devanthi & Gkatzionis, 2019; Wang, Shang, et al., 2024). This method facilitates the exploration of phenotype differences driven by microbial metabolic activities and highlights the key roles of organic acids, alcohols, and esters (Wang, Wang, et al., 2024; Yang et al., 2024) in flavor formation. Moreover, when integrated with GC–MS, HPLC, and multivariate statistical tools (e.g., PCA and OPLS-DA), metabolomics can be applied to identify characteristic flavor and nutritional markers, thereby providing new insights into the regional characteristics of fermented glutinous rice.

Most existing studies focus on single-origin products, isolated flavor or nutrient components, or specific fermentation techniques (Yang et al., 2020, Yang et al., 2021), and few have integrated physicochemical, nutritional, volatile, and metabolomic analyses across multiple regions. Moreover, the influence of regional environments and fermentation practices on metabolic differences is still unclear, leaving gaps in quality control and geographical traceability. Given the growing demand for high-quality traditional fermented foods, a comprehensive regional comparison is urgently needed.

In this study, fermented glutinous rice samples from multiple production areas across China were, for the first time, systematically analyzed through a combination of physicochemical properties, nutritional composition, volatile compounds, and non-targeted Metabolomics. The findings reveal distinct regional characteristics in terms of flavor and nutritional differences. These results provide a valuable basis for quality control and flavor mechanism research, while also contributing to the modernization and high-value development of traditional fermented foods.

## 2 Materials and methods

### 2.1 Materials

The reagents and instruments used in this study were as follows: methanol, acetonitrile, formic acid, water and isopropanol (HPLC grade, Fisher Scientific, USA). Ultra-high-performance liquid chromatography coupled with Orbitrap Exploris 240 mass spectrometry (UHPLC–Orbitrap Exploris 240, Thermo Fisher Scientific, USA) was used for metabolite analysis. An HSS T3 column (100 mm × 2.1 mm i.d., 1.8 μm; Waters, USA) was employed for chromatographic separation. Additional equipment included a JXDC-20 nitrogen evaporator (Shanghai Jingxin Industrial Development Co., Ltd., China); an LNG-T88 benchtop rapid centrifugal vacuum concentrator (Taicang Huamei Biochemical Instrument Factory, China); a Wonbio-96c high-throughput tissue grinder (Shanghai Wanbo Biotechnology Co., Ltd., China); an SBL-10DT ultrasonic cleaner (300 W-10 L; Ningbo Xinzhi Biotechnology Co., Ltd., China); a Centrifuge 5430R high-speed refrigerated centrifuge (Eppendorf, Germany); a NewClassic MF MS105DU electronic analytical balance (Mettler Toledo, Switzerland); and S-433Dup Fully Automatic Amino Acid Analyzer (Sykam, Germany).

Sample Collection: The selected fermented glutinous rice samples were obtained from different provinces representing major production regions in China, including Central China (Hubei, 30°55′N, 113°54′E), Southwest China (Sichuan, 30°34′N, 104°03′E and Guizhou, 26°34′N, 106°43′E), and East China (Jiangsu, 31°18′N,120°35′E). The samples were designated as HB_MPP (Hubei, Batch 20,250,330), SC (Sichuan, Batch 20,250,423), JS (Jiangsu, Batch 20,250,512), GZ_LYM (Guizhou, Batch 20,250,331), and SWJD (Guizhou, Batch 20,250,406). All samples were selected from the same batch, with 10 replicates per batch. All samples were prepared using water, glutinous rice, and traditional fermentation starter (jiuqu); notably, the SWJD sample also contained added Osmanthus flowers. Samples were stored at 0–4 °C after collection for subsequent analyses. Collectively, the diversity in geographical origin, raw materials, and fermentation practices ensures that the selected samples are representative of traditional fermented glutinous rice products in China.

### 2.2 Methods

#### 2.2.1 Assessment of antioxidant activity and physicochemical characteristics

The total phenolic compounds were determined using the Folin–Ciocalteu method (Paixão et al., 2007). Total flavonoids were measured by the aluminum nitrate colorimetric method (Amby et al., 2025; de Lima et al., 2024). The antioxidant activities, including DPPH (2,2-diphenyl-1-picrylhydrazyl) radical scavenging capacity, ABTS (2,2′-azino-bis (3-ethylbenzothiazoline-6-sulfonic acid)) radical cation scavenging capacity, the DPPH and ABTS assays were performed according to the Chinese national standard GB/T 39100–2020: Determination of Antioxidant Activity of Peptides-DPPH and ABTS Methods, and FRAP (ferric reducing antioxidant power), were evaluated according to the method described by Rumpf et al. (Rumpf et al., 2023), with slight modifications. The FRAP reagent was freshly prepared by mixing 10 mmol/L TPTZ, 20 mmol/L FeCl₃, and 300 mmol/L acetate buffer at a volume ratio of 1:1:10. Samples were diluted with 40% ethanol to a final concentration of 2 mg/mL. Then, 0.2 mL of the sample solution was added to the FRAP reagent preheated to 37 °C and incubated in a 37 °C water bath for 10 min. 40% ethanol was used as the blank control. The absorbance was measured at 593 nm, and a standard curve was prepared using Trolox solution (Vitamin E) within a concentration range of 0.01–0.1 mg/mL. The hydroxyl radical scavenging activity was determined based on the method reported by Pavithra et al. (Pavithra & Vadivukkarasi, 2015), with minor modifications. One milliliter of fermented rice wine sample was mixed with 1 mL of 9 mmol/L ferrous sulfate solution, 1 mL of 9 mmol/L salicylic acid ethanol solution, and 1 mL of 9 mmol/L hydrogen peroxide solution. The mixture was incubated in the dark at room temperature for 30 min, followed by centrifugation at 6000 r/min for 10 min. The absorbance of the resulting supernatant was measured at 510 nm. A control group and a blank group were included in the assay.These methods were chosen as they collectively cover radical scavenging, reducing power, and hydroxyl radical inhibition, providing a comprehensive evaluation. The contents of total sugars and reducing sugars were determined using the 3,5-dinitrosalicylic acid (DNS) method (Miller, 1959). Extraction of Reducing Sugars:Accurately weigh 3.00 g of fermented rice wine and place it in a 100 mL beaker. A small amount of distilled water was first added to form a paste, followed by 50 mL of distilled water. The mixture was homogenized and incubated in a 50 °C water bath for 20 min to allow the extraction of reducing sugars. The extract, including the precipitate, was transferred to a 50 mL centrifuge tube and centrifuged at 4000 r/min for 5 min. The precipitate was washed once with 20 mL distilled water and centrifuged again. The supernatants from both centrifugations were combined in a 100 mL volumetric flask and diluted to the mark with distilled water. The solution was thoroughly mixed and used as the reducing sugar test solution. Hydrolysis and Extraction of Total Sugars:Accurately weigh 1.00 g of fermented rice wine and place it in a 100 mL conical flask. Add 15 mL distilled water and 10 mL of 6 mol/L HCl, and heat in a boiling water bath for 30 min to hydrolyze the sugars. The completeness of hydrolysis was checked using an iodine‑potassium iodide solution. After cooling, add one drop of phenolphthalein as an indicator and neutralize with 6 mol/L NaOH until a faint pink color appears. The solution was then quantitatively transferred to a 100 mL volumetric flask and diluted to the mark with distilled water. After thorough mixing, the hydrolysate was filtered, and 10 mL of the filtrate was transferred to another 100 mL volumetric flask and diluted to the mark with distilled water. The resulting solution was thoroughly mixed and used as the total sugar test solution. One milliliter of the test solution was mixed with 1 mL of distilled water and 1.5 mL of 3,5-dinitrosalicylic acid (DNS) reagent in a 25 mL stoppered colorimetric tube. The absorbance was measured at 540 nm. Total acidity and amino acid nitrogen were measured using a pH meter titration method. Protein content was analyzed by the Kjeldahl method, while fat content was determined by the Soxhlet extraction method. Starch content was determined using a chemical titration method.

#### 2.2.2 Determination of organic acids by HPLC

(1) Sample Preparation.

Fermented glutinous rice samples were first homogenized thoroughly and stored at −20 °C prior to analysis. An accurately weighed portion of 10.00 g (±0.01 g) of the homogenized sample was transferred into a 50 mL polypropylene centrifuge tube, followed by the addition of 20 mL of deionized water. The mixture was homogenized at 15,000 rpm for 2 min using a high-speed homogenizer, and subsequently centrifuged at 4000 rpm for 5 min. The resulting supernatant was transferred to a 50 mL volumetric flask. The residue was subjected to a second extraction under the same conditions using an additional 20 mL of deionized water. Both supernatants were combined and the volume was adjusted to 50 mL with deionized water. The final extract was filtered through a 0.45 μm aqueous-phase membrane filter prior to HPLC analysis.

(2) HPLC Conditions and Analysis

Organic acids including tartaric acid, malic acid, lactic acid, citric acid, succinic acid, fumaric acid, and adipic acid were quantified using high-performance liquid chromatography (HPLC). The analysis was performed on a CAPECELL PAK MG S5 C_18_ column (4.6 mm × 250 mm, 5 μm). The mobile phases and elution programs were as follows:

For tartaric acid, malic acid, lactic acid, citric acid, succinic acid, and fumaric acid:

The mobile phase consisted of 0.1% (*v*/v) phosphoric acid solution and methanol in a 97.5:2.5 (v/v) ratio. Isocratic elution was performed for 10 min, followed by a short gradient to 100% methanol with a 5 min hold, and then returned to the original composition for re-equilibration for another 5 min.

For adipic acid: Isocratic elution was performed using a 0.1% (v/v) phosphoric acid–methanol mixture in a 75:25 (v/v) ratio for 10 min. Other chromatographic parameters remained the same.

For all analyses, the column temperature was maintained at 40 °C. The injection volume was 20 μL, and detection was carried out using a UV detector at 210 nm.

#### 2.2.3 Determination and evaluation of amino acids in fermented glutinous Rice

The determination of amino acids in fermented glutinous rice was carried out in accordance with the National Food Safety Standard of China, GB 5009.124—2016 Determination of Amino Acids in Foods. After hydrolysis and derivatization, amino acid contents were quantified using an automated amino acid analyzer. The results were expressed as mg/100 g of sample. To evaluate the nutritional quality of essential amino acids, the amino acid score was calculated based on the amino acid scoring pattern recommended by the Food and Agriculture Organization (FAO) and the World Health Organization (WHO). The amino acid score was calculated using the following equation:

Amino Acid Score (%) = aa/AA×100%.

where aa represents the content of a specific essential amino acid in the sample (mg/g protein), and AA refers to the corresponding reference value in the FAO/WHO amino acid scoring pattern (mg/g protein).

#### 2.2.4 Determination of volatile compounds in fermented glutinous Rice

(1) Sample Preparation and SPME Conditions.

Sample Preparation: A 5 mL aliquot of fermented glutinous rice sample was accurately transferred into a 20 mL headspace vial and sealed. Volatile compounds were extracted using solid-phase microextraction (SPME). SPME Conditions:The extraction procedure involved incubating the sample at 60 °C for 20 min with agitation at 400 rpm, followed by headspace extraction for 25 min. After extraction, the fiber was desorbed in the injection port for 180 s. Triple-phase coated SPME fiber:A Smart SPME fiber (DVB/C-WR/PDMS, Divinylbenzene/Carbon Wide Range/Polydimethylsiloxane) with a coating thickness of 80 μm and a length of 10 mm.

(2) GC–MS Analysis

Analysis was performed using an Agilent 8890 gas chromatograph coupled with a 7000E triple quadrupole mass spectrometer (GC–MS/MS). The separation was carried out on a DB-WAX capillary column (30 m × 0.25 mm i.d., 0.25 μm film thickness; Agilent 122–7032). The oven temperature program was as follows: initial temperature of 40 °C held for 3 min, ramped at 10 °C/min to 230 °C, and held for 15 min. Helium was used as the carrier gas at a constant flow rate of 0.8 mL/min. The injector temperature was set at 250 °C.

(3) MS Conditions

The mass spectrometer was operated in electron ionization (EI) mode at 70 eV. The ion source temperature was 250 °C, and the quadrupole temperature was 150 °C. A solvent delay of 0.20 min was applied. Full-scan mass spectra were recorded in the range of 30–550 AMU.

(4) Identification and Quantification

Qualitative analysis was performed using the Agilent MassHunter Unknowns Analysis software. Compounds were identified by comparing their mass spectra with those in the NIST 20.L mass spectral database, and further confirmed by retention indices, manual spectral interpretation, and literature references. Quantification was based on the relative peak area using the peak area normalization method, and the results were expressed as the percentage of the total chromatographic peak area.

#### 2.2.5 Non-targeted metabolomics analysis

Metabolite Extraction: A 50 μL aliquot of each sample was transferred into a 1.5 mL microcentrifuge tube and mixed with 400 μL of extraction solution (methanol: acetonitrile = 1:1, *v*/v) containing four internal standards, including L-2-chlorophenylalanine (0.02 mg/mL). The mixture was vortexed for 30 s and subjected to ultrasonic extraction at 5 °Cand 40 kHz for 30 min. Afterward, the samples were incubated at −20 °C for 30 min and centrifuged at 13,000 ×*g* for 15 min at 4 °C. The resulting supernatant was carefully transferred and evaporated to dryness under a gentle stream of nitrogen. The dried residue was reconstituted in 100 μL of reconstitution solution (acetonitrile:water = 1:1, v/v), vortexed for 30 s, and subjected to a second round of ultrasonic extraction at 5 °C for 5 min. Following centrifugation at 13,000 ×*g* for 10 min at 4 °C, the supernatant was transferred into an autosampler vial with an internal insert for subsequent UHPLC-MS/MS analysis. Additionally, 20 μL of supernatant from each sample was pooled to generate a quality control (QC) sample for system stability assessment.

(1) LC–MS/MS Analysis

Metabolomic profiling was performed using an ultra-high-performance liquid chromatography system coupled with a high-resolution Orbitrap mass spectrometer (UHPLC–Orbitrap Exploris 240, Thermo Fisher Scientific, USA).

Chromatographic Conditions: Chromatographic separation was carried out on an ACQUITY UPLC HSS T3 column (100 mm × 2.1 mm i.d., 1.8 μm; Waters, Milford, USA). The mobile phase consisted of solvent A: 95% water +5% acetonitrile (containing 0.1% formic acid), and solvent B: 47.5% acetonitrile +47.5% isopropanol +5% water (containing 0.1% formic acid). The injection volume was 3 μL, and the column temperature was maintained at 40 °C.Mass Spectrometry Conditions: Samples were ionized using electrospray ionization (ESI) and analyzed in both positive and negative ion modes. Full scan MS data were acquired using data-dependent acquisition (DDA). For full MS (MS1) scans, the automatic gain control (AGC) target was set to 1 × 10^6^ with a maximum injection time of 50 ms. For MS/MS (MS2) scans, the AGC target was set to 1 × 10^5^ with a maximum injection time of 22 ms (See Table 1).

**Table 1:** Mass spectrometry parameters.

Description	Parameters
Scan type(*m*/*z*)	70–1050
Sheath gas flow rate (arb)	60
Aux gas flow rate (arb)	20
Heater temp(°C)	350
Capillary temp (°C)	320
Spray voltage(+)(V)	3400
Spray voltage(−)(V)	−3000
S-Lens RF Level	70
Normalized collision energy(%)	20,40,60
Resolution (Full MS)	60,000
Resolution(Ms^2^)	15,000

### 2.3 Statistical analysis

Data were analyzed using one-way ANOVA followed by Tukey's post-hoc test, after confirming normality (Shapiro–Wilk) and homogeneity of variance (Levene's test). Biological triplicates were used. Statistical analysis was conducted using one-way analysis of variance (ANOVA) with SPSS 26.0 (IBM, USA), and differences were considered significant at *p* < 0.05*.* Orthogonal partial least squares discriminant analysis (OPLS-DA) was carried out using SIMCA 14.1 (Sartorius Stedim Data Analytics AB, Sweden). Graphs were generated using GraphPad Prism 9.0 (GraphPad Software, USA), and figures were prepared with Adobe Illustrator 2020 (Adobe Systems, USA).

Raw LC-MS data were imported into Progenesis QI software version 3.0 (Waters Corporation, Milford, USA) for baseline filtering, peak detection, integration, retention time correction, and peak alignment. This process generated a data matrix containing retention time, mass-to-charge ratio (*m*/*z*), and peak intensities. Feature peaks were annotated by matching MS and MS/MS spectra against metabolite databases using the same software. The mass tolerance was set to less than 10 ppm for MS data. Metabolites were identified based on secondary mass spectrometry matching scores. Databases used for annotation included the Human Metabolome Database (HMDB, https://www.hmdb.ca/), Metlin (https://metlin.scripps.edu/), and the Majorbio database. Multivariate statistical analyses, including principal component analysis (PCA) and orthogonal partial least squares discriminant analysis (OPLS-DA), were performed on the Majorbio cloud platform, and the stability of the model was assessed using seven-fold cross-validation. The reliability of the OPLS-DA model was further assessed using 1000 permutation tests, with a false discovery rate (FDR) threshold of <0.05.Variable importance in projection (VIP) scores were calculated, with VIP >1 and *p* < 0.05 considered as criteria for differential metabolites. Metabolic pathway annotation and enrichment analysis were conducted using the Kyoto Encyclopedia of Genes and Genomes (KEGG, https://www.genome.jp/kegg/). Pathway enrichment statistics were performed using the Python package scipy.stats (https://docs.scipy.org/doc/scipy/).

## 3 Results and discussion

### 3.1 Antioxidant activity and physicochemical properties of fermented glutinous Rice

**Fig. 1:**
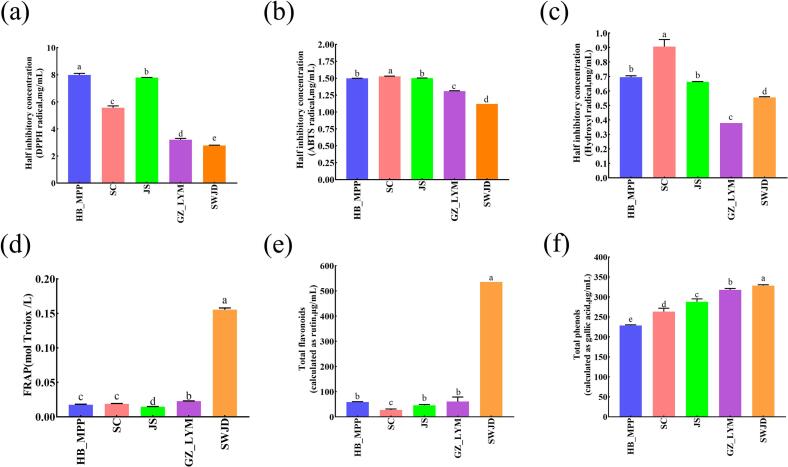
Antioxidant activities of fermented glutinous rice (HB_MPP, SC, JS, GZ_LYM, SWJD). (a) Half inhibitory concentration of fermented glutinous rice (DPPH radical). (b) Half inhibitory concentration of fermented glutinous rice (ABTS radical). (c) Half inhibitory concentration of fermented glutinous rice (Hydroxyl radical). (d) Ferric reducing antioxidant power (FRAP). (e) Total flavonoid content. (f) Total phenolic content. Data are presented as mean ± SD (*n* = 3).Different letters indicate significant differences *(p* < 0.05).

Significant differences in antioxidant activity were observed among the five fermented glutinous rice samples (HB_MPP, SC, JS, GZ_LYM, and SWJD). SWJD exhibited the strongest in vitro antioxidant activity, with the lowest half-maximal inhibitory concentrations (IC₅₀) for DPPH radicals, ABTS radicals, and hydroxyl radicals (Fig. 1a–c), measured at 2.78, 1.12, and 0.56 mg/mL, respectively. GZ_LYM followed, with IC₅₀ values of 3.10, 1.31, and 0.38 mg/mL. In contrast, HB_MPP and SC showed comparatively weaker antioxidant capacities. The differences observed among the samples across the three assays indicate that their antioxidant activities are jointly driven by multiple bioactive constituents, which is consistent with previous studies on traditional fermented foods(Bruce et al., 2025; Ma et al., 2024). DPPH is more sensitive to lipophilic antioxidants, ABTS reflects both hydrophilic and lipophilic components, and ·OH scavenging ability indicates the capacity to neutralize highly reactive radicals. Previous research has highlighted polyphenols and flavonoids as major contributors to the antioxidant potential of fermented foods, with their levels strongly influenced by raw material types, microbial communities, and fermentation processes. The superior antioxidant activities of GZ_LYM and SWJD in this study are likely associated with their higher polyphenol and flavonoid contents, in agreement with findings reported for other fermented foods (Bian et al., 2025; Yin et al., 2025) Furthermore, the FRAP assay results (Fig. 1d) indicated that SWJD exhibited the highest FRAP value, reflecting the strongest electron-donating capacity among all samples. SWJD also showed significantly higher total flavonoid (Fig. 1e) and total phenolic contents (Fig. 1f) compared to the other samples (*p* < 0.05), further confirming its superior antioxidant performance. As key natural antioxidants, flavonoids and phenolic compounds play a vital role in free radical scavenging, and these differences may be related to the biosynthesis of plant secondary metabolites, amino acid biosynthesis and metabolic enrichment in fermented glutinous rice. The outstanding antioxidant capacity of SWJD across multiple assays is likely attributed to its higher levels of these bioactive compounds, which can effectively neutralize reactive oxygen species through mechanisms such as hydrogen atom donation, electron transfer, and metal ion chelation. Compared with the antioxidant activity reported by Xu et al. (2021), SWJD showed higher activity. The reason may lie in the added Osmanthus flowers, as these flowers are rich in phenolic compounds.

In addition, the physicochemical properties of the five fermented glutinous rice samples were further assessed, including starch, total sugars, reducing sugars, protein, total acid, and amino acid nitrogen (Table 2). The results showed significant differences among the samples in the aforementioned physicochemical parameters (*p* < 0.05). JS had the highest starch content (40.59 g/100 g), while SWJD had the lowest (28.58 g/100 g). As a fermentation substrate, starch content directly affects sugar conversion efficiency. JS also had significantly higher total sugar and reducing sugar contents than the other samples (2401.53 mg/g and 1969.83 mg/g, respectively), indicating stronger fermentation activity. SWJD, on the other hand, had the lowest sugar content (555.10 mg/g and 200.96 mg/g, respectively), reflecting lower sugar conversion efficiency. Regarding protein content, JS and SWJD had relatively high levels (3.05 g/100 mL and 2.94 g/100 mL, respectively), while SC and GZ_LYM had levels below 2 g/100 mL. The GZ_LYM sample had the highest total acid content (16.93 g/g), significantly higher than the other samples, indicating a more complete lactic acid fermentation process, which facilitates the accumulation of flavor compounds. The SC and JS samples also had relatively high total acid content, while the SWJD sample had the lowest. Amino acid nitrogen, a comprehensive indicator of the degree of protein hydrolysis and the formation of flavor amino acids, was highest in the JS sample (0.92 g/g), reflecting more complete protein degradation, which contributes to enhanced umami flavor and nutritional value. The SC and GZ_LYM samples had intermediate amino acid nitrogen content, while the HB_MPP and SWJD samples had relatively low levels. Previous studies have shown that higher starch content in glutinous rice substrates can promote sugar release and fermentation activity, leading to increased ethanol and acid production (Gong et al., 2025). It has also been reported that the level of amino acid nitrogen is positively correlated with the umami taste and nutritional quality of fermented glutinous rice (Li et al., 2024). Compared with these studies, our results highlight the metabolic differences among fermented rice samples from different regions, emphasizing the critical role of the balance between yeast and lactic acid bacteria in fermentation efficiency and flavor development.

**Table 2:** Physicochemical properties of fermented glutinous rice (HB_MPP, SC, JS, GZ_LYM, SWJD).

Sample Name	Physicochemical properties						
	Starch/(g/100 g)	Total sugar/(mg/g)	Reducing sugar/(mg/g)	Protein/(g/100 mL)	Fat/(mg/100 g)	Total acid/(g/g)	Amino acid nitrogen/(g/g)
HB_MPP	27.99 ± 1.08^c^	1349.27 ± 80.68^c^	908.79 ± 123.20^b^	2.03 ± 0.09^b^	–	8.79 ± 0.14^d^	0.47 ± 0.02^d^
SC	29.72 ± 1.50^c^	1624.90 ± 108.24^b^	978.95 ± 15.31^b^	1.42 ± 0.04^c^	–	15.07 ± 0.41^b^	0.56 ± 0.02^c^
JS	40.59 ± 0.15^a^	2401.53 ± 86.90^a^	1969.83 ± 48.13^a^	3.05 ± 0.29^a^	–	13.94 ± 0.37^c^	0.92 ± 0.04^a^
GZ_LYM	36.47 ± 0.50^b^	1320.91 ± 8.36^c^	937.47 ± 20.41^b^	1.49 ± 0.03^c^	–	16.93 ± 1.12^a^	0.65 ± 0.03^b^
SWJD	28.58 ± 0.63^c^	555.10 ± 166.58^d^	200.96 ± 4.28^c^	2.92 ± 0.16^a^	–	6.97 ± 0.29^e^	0.45 ± 0.03^d^

In this study, the differences in the physicochemical indices of the five fermented glutinous rice samples reflected the metabolic activity and interactions between lactic acid bacteria and yeast. Starch, as the initial substrate for fermentation, determines the abundance of potential carbon sources, while the levels of total sugars and reducing sugars reveal the dynamic balance between saccharification efficiency and microbial consumption. These sugar sources are competitively utilized by microorganisms. Yeast converts them into ethanol, while lactic acid bacteria mainly produce acid, which is directly reflected in the total acid content. In addition, nitrogen metabolism is key to flavor formation. Protein is degraded into amino acid nitrogen by microbial proteases, generating a wealth of umami substances and flavor precursors, significantly enhancing the value of the product. In summary, these indicators are interrelated and together constitute the core system for evaluating fermentation efficiency.

“-” indicates not detected. Different letters indicate significant differences (*p* < 0.05).

### 3.2 Organic acids

Organic acids play a crucial role in shaping the flavor characteristics, taste perception, and overall sensory profile of fermented foods. In traditional Chinese fermented glutinous rice, organic acids contribute significantly to flavor development. For instance, lactic acid imparts a mild sourness, while malic acid provides a refreshing taste with a lingering aftertaste (Qiu, Liu, et al., 2025). The presence of appropriate levels of organic acids enhances the mouthfeel, enriches flavor complexity, and improves overall product quality. Moreover, organic acids serve as important precursors in flavor biosynthesis, as they can undergo esterification with alcohols to generate esters with distinctive aromatic properties, further contributing to the characteristic aroma of fermented glutinous rice (He et al., 2024; Qiu et al., 2024).

Organic acid analysis of the five sample groups revealed that lactic acid, malic acid, and fumaric acid were the most abundant and widely distributed organic acids (Table 3). GZ_LYM had the highest lactic acid and malic acid contents, at 279.88 mg/kg and 86.98 mg/kg, respectively, followed by SWJD at 170.62 mg/kg and 74.50 mg/kg, respectively. The organic acid composition trends of HB_MPP, GZ_LYM, and SWJD samples were largely consistent, with lactic acid > malic acid > fumaric acid.

**Table 3:** Organic acid content in different fermented glutinous rice samples (HB_MPP,SC,JS,GZ_LYM,SWJD).

Indicators	Calibration curve, R2	Retention time (min)	Content (mg/kg)					Acidic taste characteristics
			HB_MPP	SC	JS	GZ_LYM	SWJD	
Lactic acid	Y = -0.92 + 5.86×,R2 = 0.998	6.3200	168.02 ± 18.92^b^	164.04 ± 13.29^b^	92.76 ± 2.77^c^	279.88 ± 0.00^a^	170.62 ± 37.93^b^	Mild acidity with a strong sour sensation
Malic acid	Y = -1.35 + 10.54×,R2 = 0.998	5.1870	67.94 ± 4.55^b^	–	–	86.98 ± 7.95^a^	74.50 ± 3.14^a^	Apple-like aroma, refreshing taste, and long-lasting aftertaste
Citric acid	Y = -1.1 + 14.11×,R2 = 0.998	8.1870	–	–	35.04 ± 0.11^a^	–	–	Rounded acidity, palatable, and brisk
Tartaric acid	Y = -0.49 + 19.25×,R2 = 0.999	4.3200	–	–	–	–	–	Slight astringency with pronounced acidity
Succinic acid	Y = -3.28 + 7.58×,R2 = 0.9983	9.0530	–	–	–	–	–	Slightly sour and astringent
Fumaric acid	Y = -1.61 + 10.36×,R2 = 0.999	10.6370	0.51 ± 0.00^c^	2.00 ± 0.04^a^	0.13 ± 0.01^e^	0.68 ± 0.04^b^	0.26 ± 0.00^d^	Strong astringency and intense sourness

The distribution pattern of organic acids is closely related to the dominant microbial flora and their metabolic characteristics within the sample. No malic acid was detected in SC and JS, and their organic acid compositions showed a lactic acid > fumaric acid pattern. The samples were enriched with homofermentative lactic acid bacteria (such as *Lactobacillus* and *Oenococcus oeni*) with malic-lactic enzyme (MLE) activity. These bacteria decarboxylate malic acid into lactic acid and CO₂, leading to its depletion. This metabolism not only reduces the sharpness of the acidity but also softens the mouthfeel. In contrast, higher levels of malic acid were detected in GZ_LYM and SWJD samples, possibly indicating that the dominant bacterial communities were heterofermentative lactic acid bacteria or yeasts lacking MLE activity (Landete et al., 2013; Schümann et al., 2013). Furthermore, citric acid (35.04 mg/kg) was only detected in JS; it was not detected in the other samples. Citric acid is a characteristic organic acid found in many fruits. Its presence in trace amounts contributes to pleasant fruity aromas and serves as an important precursor for microbial degradation, contributing to the formation of ester aromas such as acetate. Tartaric acid and succinic acid were not detected in any samples, suggesting that these acids were either below the detection limit or metabolized by microorganisms during fermentation. The differences in the types and contents of these organic acids directly shape the unique sourness profile of each sample, and through esterification reactions with compounds such as phenylethanol, isobutanol and n-propanol, potentially determine the aroma complexity and typicality of the final product.

“-” indicates not detected. Different letters indicate significant differences (*p* < 0.05).

### 3.3 Amino acids

Fermented glutinous rice is nutritionally rich, containing a variety of amino acids, including essential amino acids (EAAs) and non-essential amino acids (NEAAs), providing an important source of nutrients for the human body. The free amino acid profiles of five fermented glutinous rice samples were analyzed (Table 4), revealing a total of 18 free amino acids, including seven EAAs, ten NEAAs, and one physiologically active non-protein amino acid, γ-aminobutyric acid (GABA). Total amino acid (TAA) contents ranged from 19.09 to 28.53 mg/g, with the JS sample exhibiting the highest TAA content (28.53 mg/g) and HB_MPP the lowest (19.09 mg/g). EAA contents ranged from 6.61 to 9.99 mg/g, also highest in JS and lowest in HB_MPP. Among EAAs, leucine (Leu) and valine (Val) were relatively abundant, whereas methionine (Met) content was consistently the lowest. Among NEAAs, glutamic acid (Glu) was the most abundant in all samples, far exceeding other amino acids, while GABA exhibited the lowest content. The contents of TAA, EAA, and NEAA followed a consistent trend: JS > GZ_LYM > SWJD > SC > HB_MPP.

**Table 4:** Amino Acid Content of Five Types of Fermented Glutinous Rice Samples (HB_MPP,SC,JS,GZ_LYM,SWJD).

Amino Acid		Amino Acid Content/(mg/g)				
		HB_MPP	SC	JS	GZ_LYM	SWJD
Essential Amino Acids	Threonine (Thr)	0.96 ± 0.11^d^	1.03 ± 0.00^cd^	1.26 ± 0.01^a^	1.24 ± 0.00^ab^	1.05 ± 0.06^bc^
	Valine (Val)	1.20 ± 0.02^d^	1.26 ± 0.04^d^	1.86 ± 0.09^a^	1.67 ± 0.01^b^	1.42 ± 0.01^c^
	Methionine (Met)	0.12 ± 0.05^bc^	0.11 ± 0.03^bc^	0.21 ± 0.03^a^	0.19 ± 0.03^b^	0.06 ± 0.01^c^
	Isoleucine (Ile)	0.96 ± 0.05^b^	1.00 ± 0.09^b^	1.40 ± 0.01^a^	1.40 ± 0.17^a^	1.21 ± 0.07^a^
	Leucine (Leu)	1.76 ± 0.03^d^	1.85 ± 0.03^c^	2.73 ± 0.01^a^	2.58 ± 0.05^b^	2.28 ± 0.09^b^
	Phenylalanine (Phe)	1.08 ± 0.01^c^	1.14 ± 0.01^c^	1.67 ± 0.01^a^	1.50 ± 0.01^b^	1.36 ± 0.18^b^
	Lysine (Lys)	0.52 ± 0.01^e^	0.67 ± 0.00^d^	0.85 ± 0.00^a^	0.75 ± 0.00^b^	0.73 ± 0.01^c^
	Total Amount of Essential Amino Acids	6.61 ± 0.23^e^	7.05 ± 0.18^d^	9.99 ± 0.12^a^	9.33 ± 0.21^b^	8.12 ± 0.30^c^
Non-essential Amino Acids	Aspartic acid (Asp)	1.47 ± 0.05^e^	1.61 ± 0.01^d^	2.19 ± 0.00^a^	2.02 ± 0.01^b^	1.69 ± 0.03^c^
	Serine (Ser)	1.16 ± 0.05^e^	1.28 ± 0.00^d^	1.73 ± 0.01^a^	1.61 ± 0.01^b^	1.38 ± 0.02^c^
	Glutamic acid (Glu)	3.23 ± 0.5^b^	3.07 ± 0.01^b^	4.32 ± 0.01^a^	4.49 ± 0.70^a^	3.30 ± 0.00^ab^
	Glycine (Gly)	0.87 ± 0.00^b^	1.18 ± 0.17^a^	1.33 ± 0.02^a^	1.30 ± 0.070^a^	1.14 ± 0.05^a^
	Alanine (Ala)	1.00 ± 0.19^c^	1.31 ± 0.07^b^	1.67 ± 0.02^a^	1.66 ± 0.09^a^	1.42 ± 0.00^ab^
	Cystine (Cys)	0.38 ± 0.05^a^	0.27 ± 0.14^a^	0.26 ± 0.33^a^	0.76 ± 0.09^a^	0.36 ± 0.18^a^
	Tyrosine (Tyr)	1.06 ± 0.00^b^	1.24 ± 0.06^b^	1.43 ± 0.00^ab^	1.56 ± 0.10^ab^	1.78 ± 0.42^a^
	Gamma-aminobutyric acid (γ-GABA)	0.02 ± 0.01^d^	0.05 ± 0.00^c^	0.06 ± 0.00^b^	0.07 ± 0.00^a^	0.07 ± 0.00^a^
	Histidine (His)	0.49 ± 0.00^e^	0.57 ± 0.00^d^	0.77 ± 0.00^a^	0.72 ± 0.01^b^	0.59 ± 0.00^c^
	Arginine (Arg)	1.82 ± 0.02^e^	2.13 ± 0.01^d^	3.05 ± 0.02^a^	2.74 ± 0.00^b^	2.34 ± 0.02^c^
	Proline (Pro)	0.98 ± 0.00^d^	1.84 ± 0.00^a^	1.73 ± 0.01^a^	1.60 ± 0.06^b^	1.39 ± 0.01^c^
	Total Amount of Non-essential Amino Acids	12.48 ± 0.40^d^	14.54 ± 0.18^c^	18.54 ± 0.33^a^	18.51 ± 1.12^a^	15.45 ± 0.16^b^
Total		19.09	21.59	28.53	27.84	23.57

Comparison with the WHO/FAO ideal protein pattern and calculation of amino acid scores (AAS) indicated that lysine (Lys) was the first limiting amino acid in all five fermented glutinous rice samples, whereas the highest scores were observed for phenylalanine plus tyrosine (Phe + Tyr)(Fig.S1). This finding is consistent with many cereal-based fermented foods, such as traditional Chinese huangjiu (Li et al., 2025) and fermented glutinous rice, where lysine limitation is common due to its low content in rice and susceptibility to loss during the Maillard reaction. Nonetheless, the EAA/TAA ratios of fermented glutinous rice (approximately 32–35%) were relatively high compared with other cereal-based fermented products, suggesting a favorable amino acid nutritional profile. The exceptionally high total amino acid and EAA contents in JS were closely associated with its strong proteolytic activity observed previously. During fermentation, extracellular proteases and peptidases secreted by microbial communities (e.g., *Aspergillus*, yeasts, and lactic acid bacteria) extensively hydrolyze substrate proteins into free amino acids (Christensen et al., 2022). The higher amino acid nitrogen and total acidity observed in JS indicate a microbial community with elevated protease activity and more complex microbial interactions, resulting in more thorough protein degradation and amino acid release. This not only enhances the nutritional value of the product but also lays a foundation for its flavor development.

It is noteworthy that these amino acids are not only nutrient carriers but also key precursors of flavor compounds. Glutamic acid (Glu), the most abundant amino acid, contributes significantly to umami taste (Sakai et al., 2024), while aspartic acid (Asp) also imparts umami, together conferring a rich and mellow flavor to fermented glutinous rice (Ramesh et al., 2025). Phenylalanine (Phe) serves as a precursor for aromatic volatile compounds, such as 2-phenylethanol, which imparts a rose-like aroma and is further transformed by microbial metabolism (Rajendran et al., 2024). Collectively, the amino acid profile of fermented glutinous rice reflects the metabolic intensity of the fermenting microbial community and comprehensively determines the nutritional quality and flavor characteristics of the product.

Different letters indicate significant differences (*p* < 0.05).

### 3.4 Volatile compounds

**Fig. 2:**
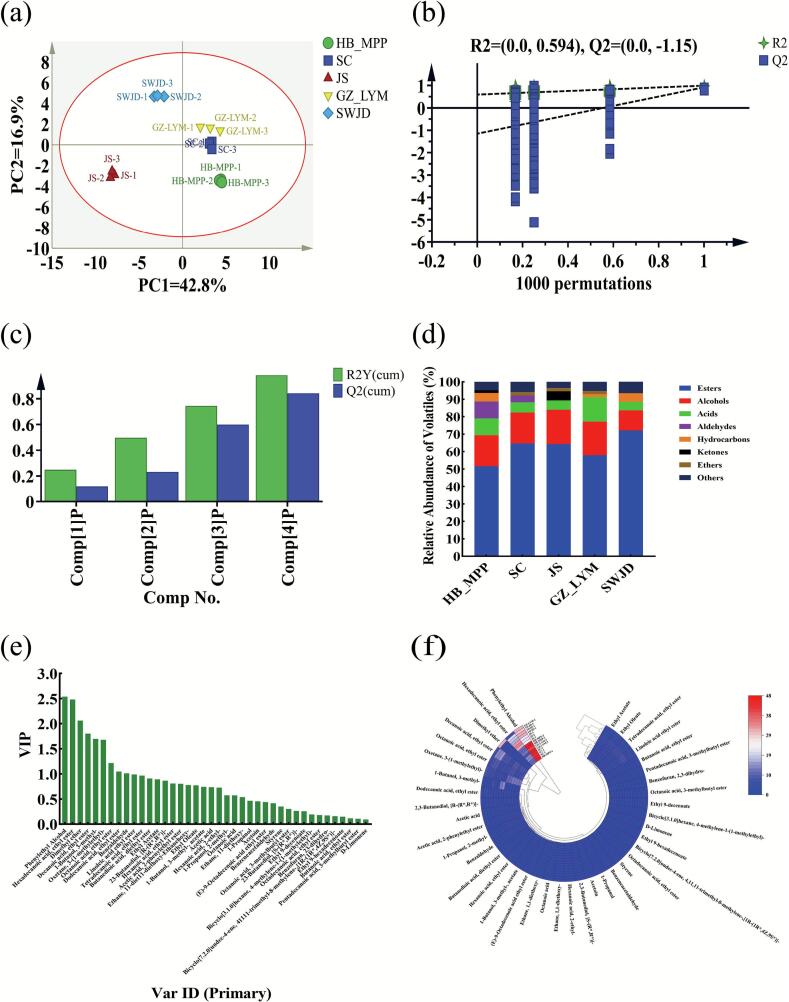
Multivariate statistical and classification analysis of volatile compounds (HB_MPP,SC,JS,GZ_LYM,SWJD). (a) Principal Component Analysis (PCA) score plot. (b) Orthogonal Partial Least Squares Discriminant Analysis (OPLS-DA) permutation test. The model showed strong performance with R^2^X (cum) = 0.980 and Q^2^ (cum) = 0.842, indicating good model fit and predictive ability. (c) OPLS-DA model evaluation: R^2^Y and Q^2^ represent the model's explanatory and predictive abilities, respectively. Higher cumulative values of R^2^Y and Q^2^ suggest better model robustness and reliability. (d) Relative abundance of volatile compound categories. (e) Variable Importance in Projection (VIP) scores of volatile metabolites. A VIP score ≥ 1 indicates a metabolite with significant contribution to intergroup discrimination. (f) Heatmap analysis of VOCs. The color coding scale from blue to red corresponds to the change in volatile compound content from low to high. (For interpretation of the references to color in this figure legend, the reader is referred to the web version of this article.)

The flavor compounds of fermented glutinous rice are primarily derived from the complex synergistic interactions among various volatile organic compounds (VOCs) (Liu et al., 2020). Due to differences in VOCs composition and relative concentrations, fermented glutinous rice samples from different regions exhibit distinctive aroma characteristics. In this study, GC–MS was employed to analyze the differences in volatile compounds among fermented glutinous rice samples from different regions of China. A total of 40 VOCs were preliminarily identified (Table S1), including esters, alcohols, acids, aldehydes, hydrocarbons, ketones, ethers, and others, which collectively form the primary aroma profile of fermented glutinous rice. To further investigate the variation in VOCs, multivariate statistical analyses were performed on the 40 identified aroma compounds. The PCA results revealed a clear separation among the five sample groups along the first and second principal components. Specifically, SWJD was located in the upper quadrant, JS in the lower left, HB_MPP in the lower right, and GZ_LYM and SC were centrally positioned and relatively close to each other. These findings indicate significant regional differences in the VOC profiles of fermented glutinous rice (Fig. 2a). To gain deeper insights into the changes in volatile compounds, an OPLS-DA model was constructed. The performance of the OPLS-DA models was evaluated using R^2^X (cum), R^2^Y (cum), and Q^2^ values, which represent the cumulative explained variance of the predictor matrix (X), the response matrix (Y), and the predictive ability of the model, respectively. Generally, values approaching 1.0 for all three indicators suggest a robust and reliable model, while a Q^2^ value greater than 0.5 is indicative of good predictive performance. The model showed strong explanatory and predictive power, with R^2^X(cum) = 0.980 and Q^2^(cum) = 0.842. Additionally, a permutation test with 1000 iterations confirmed the robustness of the model and the absence of overfitting, as evidenced by the Q^2^ intercept being below zero (Fig. 2b-2c).

The relative abundances of various VOC classes in fermented glutinous rice exhibited pronounced changes, with esters, alcohols, and acids showing the most marked variations. Such shifts likely reflect the microbial degradation of glutinous rice starch during fermentation, which releases a wealth of aroma precursors; regional factors may further modulate VOC accumulation and release patterns. A clustered heat map of VOC relative abundances (Fig. 2d) revealed that, despite a broadly similar aroma profile across regions, each compound class differed significantly in concentration. Specifically, aldehydes were enriched in both HB_MPP and SC samples; hydrocarbons predominated in HB_MPP, GZ_LYM, and SWJD; and ketones were characteristic of HB_MPP and JS. By selecting variables with VIP ≥ 1 in the OPLS-DA model (Qiu, Ru, et al., 2025), nine key aroma-active compounds were identified (Fig. 2e): phenylethanol (VIP = 2.5380), hexadecanoic acid ethyl ester (VIP = 2.4795), dimethyl ether (VIP = 2.0612), decanoic acid ethyl ester (VIP = 1.8004), 3-methyl-1-butanol (VIP = 1.6966), 3-(1-methylethyl)-oxetane (VIP = 1.6769), octanoic acid ethyl ester (VIP = 1.2158), dodecanoic acid ethyl ester (VIP = 1.0459), and benzaldehyde (VIP = 1.0098). These compounds constitute the chemical basis of fermented glutinous rice's signature aroma.

Fig.2f illustrates the relative abundance of volatile compounds (VOCs) across different fermented glutinous rice samples. Notably, 2-phenylethanol (VIP = 2.5380) played a dominant role in differentiating the samples. Known for its rose- and honey-like aroma, 2-phenylethanol exhibited the highest relative abundance in this study, imparting a rich and distinctive floral note to fermented glutinous rice. Consistent with previous analyses of fermented glutinous rice and huangjiu, 2-phenylethanol is recognized as a key aroma compound, further confirming its central role in the aroma profile of fermented glutinous rice. Previous studies have reported that 2-phenylethanol is biosynthesized via the Ehrlich pathway, in which yeast decarboxylates and reduces phenylalanine, a process particularly active during peak fermentation (Zhang et al., 2025). Metabolomic KEGG analysis in this study also revealed significant enrichment of the aromatic amino acid metabolism pathway, indicating that 2-phenylethanol production is closely linked to microbial phenylalanine metabolism medium-chain esters, such as ethyl hexadecanoate and ethyl decanoate, typically contribute sweet, milky, and fruity notes and are formed via esterification of ethanol with fatty acids catalyzed by microbial esterases (Gong et al., 2022). Their increased abundance in fermented glutinous rice not only enhanced overall richness and tropical fruitiness but also aligns with previous reports on microbial regulation of lipid metabolism, highlighting the importance of fatty acid–alcohol esterification in aroma accumulation (Chen et al., 2022) ether (VIP = 2.0612), although present at relatively low abundance, has an extremely low odor threshold and imparts a fresh ether-like note with subtle spiciness, thereby enhancing the top-note complexity of the aroma profile. Its formation may involve dehydration or cleavage reactions of carbohydrate or ethanol metabolism intermediates. 3-Methyl-1-butanol (isoamyl alcohol, VIP = 1.6966) is a typical higher alcohol in fermented beverages, contributing banana, malt, and body notes. It is derived from leucine via the Ehrlich pathway and can be further esterified to produce esters such as isoamyl acetate, conferring sweet and fruity characteristics that enrich fermented glutinous rice flavor. Interestingly, 3-(1-methylethyl) oxetane (VIP = 1.6769) also exhibited significant variation among samples. As a less-reported oxygen-containing heterocyclic compound, it may be generated during fermentation through cyclization of alcohols or aldehydes with small molecules. Although the precise metabolic pathway remains unclear, its light, clean aroma and structural stability may allow it to serve as a background aroma contributor (Zhang et al., 2024) Among all esters, ethyl octanoate (VIP = 1.2158) and ethyl dodecanoate (VIP = 1.0459) impart mild fatty and creamy notes, further enhancing the overall fullness of the aroma. Notably, ethyl octanoate, in combination with higher alcohols such as isoamyl alcohol, generates pronounced tropical fruity characteristics. Benzaldehyde (VIP = 1.0098), despite its low relative abundance, contributes almond-like and sweet notes that are important for the overall aroma structure (Yang et al., 2025).

Sensory correlations indicate that the aroma profile of fermented glutinous rice is primarily constructed by esters and alcohols, with 2-phenylethanol contributing floral notes and medium-chain esters such as ethyl decanoate and ethyl hexadecanoate enhancing fruity and creamy layers. These aroma characteristics are highly consistent with the sensory attributes of regional fermented glutinous rice. Collectively, the accumulation of 2-phenylethanol and medium- to long-chain fatty acid ethyl esters reflects active microbial amino acid transformation and lipid esterification during fermentation, highlighting the central roles of *Rhizopus* and *Saccharomyces* species in shaping the characteristic aroma profile of fermented glutinous rice.

### 3.5 Non-targeted metabolomics analysis

#### 3.5.1 Metabolite classification and principal component analysis (PCA)

**Fig. 3:**
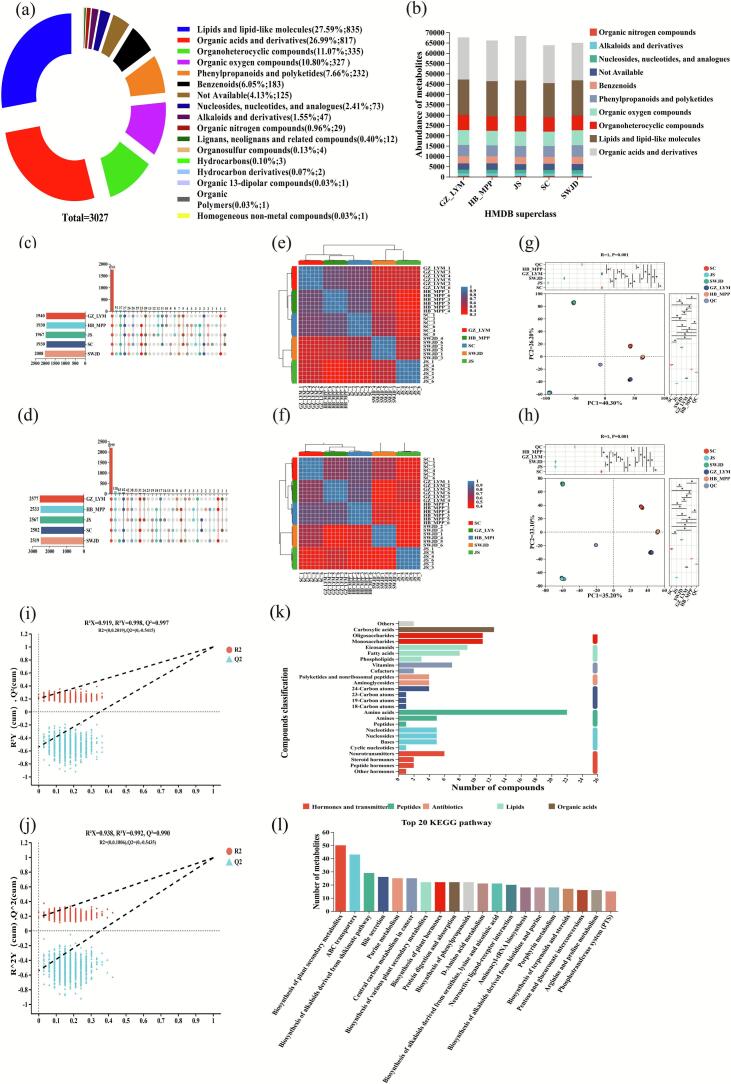
Multivariate statistical analysis and functional annotation of metabolites in fermented glutinous rice (HB_MPP,SC,JS,GZ_LYM,SWJD). (a) Number of annotated metabolites. (b) Heatmap of metabolite abundances. (c-d) Upset plots showing shared and unique metabolites across samples in POS and NEG modes. (e-f) Hierarchical clustering heatmaps of samples under POS and NEG modes. (g-h) Principal component analysis (PCA) score plots in positive (POS) and negative (NEG) ionization modes. (i-j) PLS-DA permutation test results for model validation. (k) KEGG-based classification of identified compounds. (l) Enriched KEGG metabolic pathways. The performance of the PLS-DA models was evaluated using R^2^X (cum), R^2^Y (cum), and Q^2^ values, which represent the cumulative explained variance of the predictor matrix (X), the response matrix (Y), and the predictive ability of the model, respectively. Values approaching 1.0 for all three indicators suggest a robust and reliable model, while a Q^2^ value greater than 0.5 is indicative of good predictive performance. In this study, the cationic PLS-DA model exhibited R^2^X (cum), R^2^Y (cum), and Q^2^ values of 0.919, 0.997, and 0.998, respectively, whereas the anionic PLS-DA model yielded values of 0.938, 0.992, and 0.990, respectively.

In this study, a total of 2044 metabolites were identified in positive ion mode and 2682 in negative ion mode, with 3027 metabolites annotated in total (Fig. 3a). The metabolites were primarily classified as lipids and lipid-like molecules (27.59%), organic acids and derivatives (26.99%), organic heterocyclic compounds (11.07%), organic oxygen compounds (10.80%), phenylpropanoids and polyketides (7.66%), benzenoids (6.05%), nucleosides and nucleotides (2.41%), alkaloids and derivatives (1.55%), and organic nitrogen compounds (0.96%). Among these, lipids/lipid-like molecules and organic acids and derivatives accounted for more than half of the total metabolites, representing the major drivers of sample differences. HMDB-based classification revealed that samples from different regions exhibited pronounced variation in lipid metabolism and flavor-related metabolites (Fig. 3b), suggesting that the metabolic profiles of fermented glutinous rice may be influenced by raw materials and microbial communities.

Venn analysis indicated that, in positive ion mode, 1761 metabolites were shared among all five samples, with SWJD and JS possessing 51 and 12 unique metabolites, respectively, while GZ_LYM had none. In negative ion mode, 2199 metabolites were shared, with SWJD and JS exhibiting 63 and 16 unique metabolites, respectively, and SC lacking unique metabolites (Fig. 3c-d). Notably, SWJD-specific metabolites were predominantly flavonoids, consistent with its high antioxidant activity, whereas JS-specific metabolites mainly involved lipids and aroma precursors, correlating with its rich aroma and high amino acid content.

Correlation analysis showed that, under both ion modes, HB_MPP shared the highest similarity with SC and GZ_LYM (Fig. 3e-f). Principal component analysis (PCA) revealed tight clustering of six biological replicates for each sample, indicating high reproducibility and reliability, while the distinct separation among groups reflected significant metabolic differences (Fig. 3g-h). In both ion modes, PC1 and PC2 explained 70.00% and 71.90% of the variance, capturing the majority of the metabolic information. The PLS-DA models further confirmed these differences, with positive ion mode R^2^X (cum), R^2^Y (cum), and Q^2^ values of 0.919, 0.997, and 0.998, and negative ion mode values of 0.938, 0.992, and 0.990, indicating robust model fitting and high predictive capability (Fig. 3i-j).

KEGG pathway annotation revealed that the identified metabolites were involved in multiple biological processes, including biosynthesis of plant secondary metabolites, ABC transporters, shikimate pathway-derived alkaloid biosynthesis, bile secretion, purine metabolism, central carbon metabolism, protein digestion and absorption, and phenylpropanoid biosynthesis (Fig. 3k-l). These results indicate that the metabolic differences among fermented glutinous rice samples are primarily driven by lipids, organic acids, and specific flavor precursors, which are closely associated with their antioxidant activity and aroma profiles.

#### 3.5.2 Screening and clustering analysis of differential metabolites

**Fig. 4:**
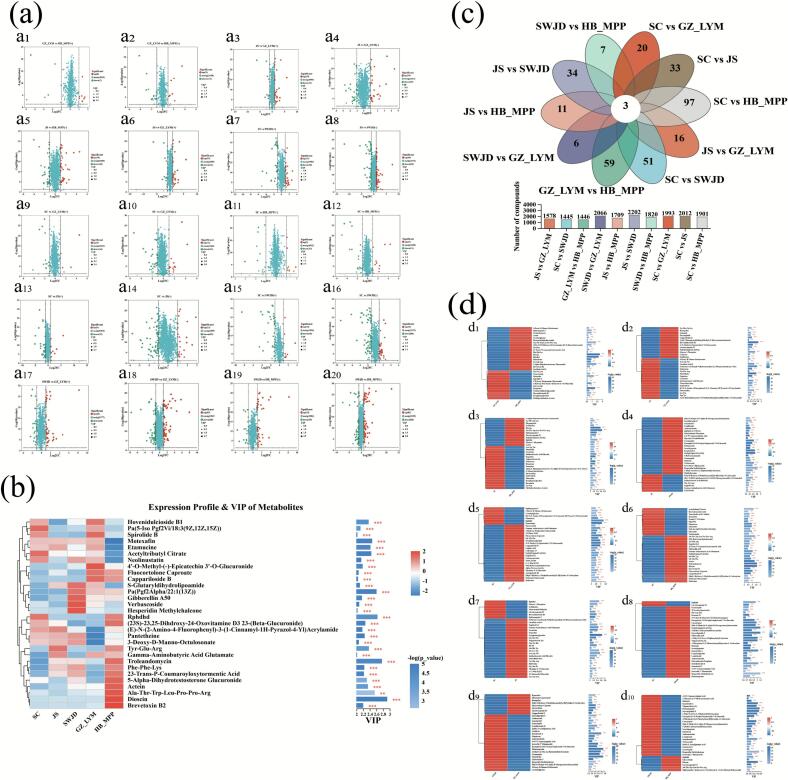
Analysis of differential metabolites in fermented glutinous wine samples (GZ_LYM vs HB_MPP,JS vs GZ_LYM,JS vs HB_MPP,JS vs SWJD,SC vs GZ_LYM,SC vs HB_MPP,SC vs JS,SC vs SWJD,SWJD vs GZ-LYM,SWJD vs HB_MPP). (a) Volcano plots of differential metabolites (a_1_–a_20_): Odd-numbered plots represent metabolites in positive ion mode, even-numbered plots in negative ion mode. Differential metabolites were identified using the criteria of VIP > 1.0, FC >2 or FC < 0.5, and *p* < 0.05. (b) VIP analysis across multiple comparison groups. (c) Petal-shaped Venn diagram showing the distribution of group-specific and shared metabolites. Numbers within each petal represent metabolites unique to that group, while the center indicates metabolites common to all groups. The adjacent bar plot shows the total number of metabolites in each comparison set. (d) VIP analysis between two groups. In both (b) and (d), the left panel displays a hierarchical clustering dendrogram of metabolites; shorter branch lengths indicate more similar expression patterns among metabolites within the group. The right panel shows a VIP bar plot, where bar length represents the contribution of each metabolite to intergroup differentiation, and bar color represents statistical significance. *p* < 0.05 (*), *p* < 0.01 (**), and *p* < 0.001 (***).

Differential metabolites were identified based on three key parameters: variable importance in projection (VIP), fold change (FC), and *p*-value. In this study, metabolites with VIP > 1.0, FC >2 or FC < 0.5, and *p* < 0.05 were considered significantly different. The overall distribution of differential metabolites was visualized using volcano plots. As shown in Fig. 4a, under positive ion mode, the numbers of upregulated metabolites in the comparisons of GZ_LYM vs HB_MPP, JS vs GZ_LYM, JS vs HB_MPP, JS vs SWJD, SC vs GZ_LYM, SC vs HB_MPP, SC vs JS, SC vs SWJD, SWJD vs GZ_LYM, and SWJD vs HB_MPP were 8, 29, 39, 31, 5, 9,9, 10, 43, and 43, respectively, while the corresponding downregulated metabolites were 7, 11, 15, 30, 16, 13, 33,38, 24, and 19. In negative ion mode, the numbers of upregulated metabolites were 9, 29, 36, 60, 14,11, 23, 30 65, and 83, while downregulated metabolites numbered 15, 29, 38, 50, 32, 21, 69, 80, 68, and 60, respectively. Hierarchical clustering heatmaps and VIP bar plots were used to illustrate the expression patterns of differential metabolites across all FGW samples, as well as to indicate the importance of individual metabolites in multivariate and univariate analyses (Fig. 4b). These visualizations provided intuitive insights into metabolite abundance trends and biological relevance. The petal-shaped Venn diagram (Fig. 4c) illustrates the distribution of unique and shared differential metabolites among the pairwise comparisons. Specifically, the numbers of unique metabolites identified in GZ_LYM vs HB_MPP, JS vs GZ_LYM, JS vs HB_MPP, JS vs SWJD, SC vs GZ_LYM, SC vs HB_MPP, SC vs JS, SC vs SWJD, SWJD vs GZ_LYM, and SWJD vs HB_MPP were 59, 16, 11, 34, 20, 97, 33, 51, 6, and 7, respectively. Notably, only three metabolites were found to be common across all comparison groups, highlighting the distinct metabolic profiles among the different fermented glutinous rice samples.Further hierarchical clustering analysis was performed to visualize the variation in expression levels of these differential metabolites across groups, with color intensity reflecting the magnitude of up- or downregulation (Fig. 4d). The top 14 metabolites in both positive and negative ion modes, ranked by VIP scores, are summarized in Tables S2–S11.

Differential metabolite analysis demonstrated pronounced regional divergence in fermented glutinous rice, with major variations observed in flavonoids, glycosides, peptides, saponins, and small organic acids. In SWJD, multiple flavonoids and glycosides (e.g., hesperidin methylchalcone, styraxlignolide F, verbascoside) were significantly upregulated. These compounds are generally associated with free radical scavenging and metal ion chelation, and their enrichment was highly consistent with the antioxidant assay results(Bellavite & Imbriano, 2025), indicating that plant-derived secondary metabolites form a key metabolic basis of the regional characteristics of SWJD. In contrast, HB_MPP and JS were enriched in functional peptides (e.g., Ala-Thr-Trp-Leu-Pro-Pro-Arg) and amino acid–related metabolites. These compounds correlated with higher free amino acid levels and stronger proteolytic activity, reflecting region-specific nitrogen and protein metabolism and explaining the accumulation of umami-related compounds. JS, in particular, showed the highest protein and total free amino acid contents, further highlighting amino acid biosynthesis and metabolism as its dominant metabolic features. Meanwhile, saponin metabolites (e.g., licoricesaponin F3, dioscin, ophiopogonin C) showed significant variation in GZ_LYM and SC. Although commonly linked to immunomodulatory activity in medicinal plants, their precise roles in fermented glutinous rice remain unclear; nonetheless, they may serve as indicators of region-specific accumulation of plant-derived constituents. Notably, differences in lipid derivatives and small organic acids were consistent with GC–MS results on volatile esters and organic acids. For instance, ethyl octanoate and ethyl decanoate—key esters contributing to aroma—showed regional differences in their precursors, suggesting that lipid–alcohol esterification pathways are critical drivers of flavor divergence. These findings highlight that microbial communities and raw material conditions across regions not only regulate amino acid and flavonoid metabolism but also markedly influence flavor compound formation.

In summary, the enrichment of flavonoids and glycosides in SWJD underpins its antioxidant properties; the accumulation of peptides and amino acid derivatives in HB_MPP and JS reveals advantages in amino acid biosynthesis, protein digestion, absorption, and signaling; while variations in saponins and lipid-derived metabolites in GZ_LYM and SC reflect region-specific differences in functional and flavor-related compounds. These metabolic distinctions align well with physicochemical indices, amino acid composition, and aroma analysis, confirming that “regional characteristics” are jointly shaped by microbial activity, raw material variability, and metabolic pathway regulation.

#### 3.5.3 KEGG pathway enrichment analysis of differential metabolites

**Fig. 5:**
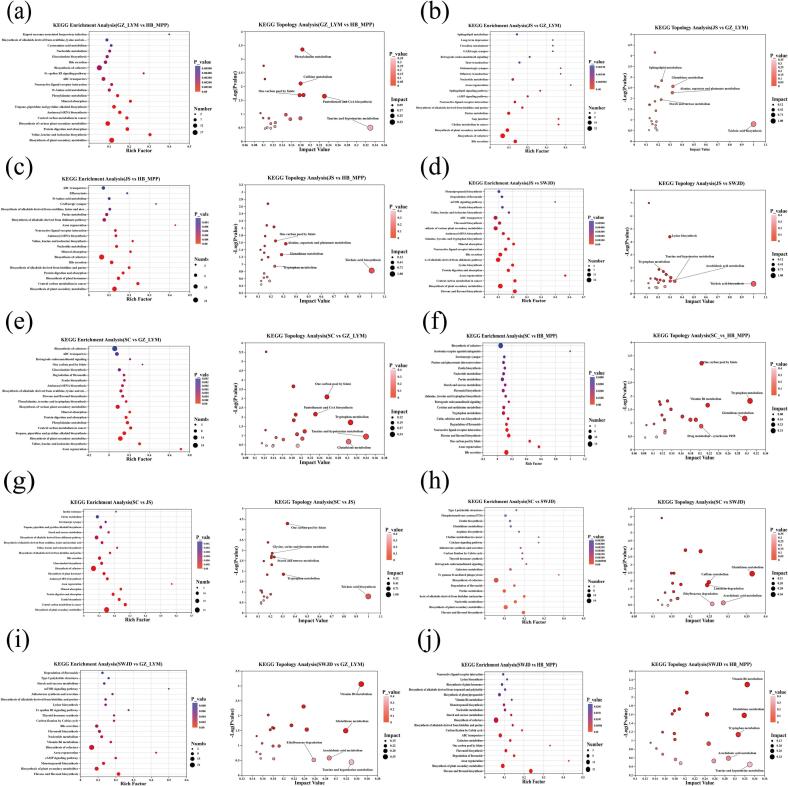
KEGG pathway enrichment and topological analysis of differential metabolites (a, GZ_LYM vs HB_MPP; b, JS vs GZ_LYM; c, JS vs HB_MPP; d, JS vs SWJD; e, SC vs GZ_LYM; f, SC vs HB_MPP; g, SC vs JS; h, SC vs SWJD; i, SWJD vs GZ-LYM; j, SWJD vs HB_MPP). KEGG pathway enrichment bubble plots. Each bubble represents a KEGG pathway; bubble size indicates the number of enriched compounds involved in the pathway, and color reflects the statistical significance (*P*-value), with darker colors representing higher significance. KEGG topological analysis bubble plots. Each bubble corresponds to a KEGG pathway. The x-axis represents the impact value, indicating the relative importance of the metabolites within the pathway, and the y-axis shows pathway enrichment significance as –log10(P-value). Bubble size corresponds to the impact value, with larger bubbles indicating higher pathway importance.

The differential metabolites identified across different comparison groups were mapped to the KEGG database to obtain information on the metabolic pathways involved, and KEGG pathway enrichment bubble plots (top 20 pathways) were generated, as shown in Fig. 5. Under both positive and negative ion modes, in the comparison of GZ_LYM vs HB_MPP, the major enriched pathways were biosynthesis of plant secondary metabolites, valine, leucine and isoleucine biosynthesis, protein digestion and absorption, and biosynthesis of various plant secondary metabolites. In the JS vs GZ_LYM group, the main enriched pathways were bile secretion, biosynthesis of cofactors, biosynthesis of plant secondary metabolites, and choline metabolism in cancer. In the JS vs HB_MPP group, prominent pathways included biosynthesis of plant secondary metabolites, central carbon metabolism in cancer, biosynthesis of plant hormones, and protein digestion and absorption. For JS vs SWJD, the major enriched pathways were Flavone and flavonol biosynthesis, Biosynthesis of plant secondary metabolites, central carbon metabolism pathways, which overlap with energy and amino acid metabolism, and Axon regeneration. The SC vs GZ_LYM group showed significant enrichment in pathways related to Axon regeneration, Valine, leucine and isoleucine biosynthesis, Biosynthesis of plant secondary metabolites, and Tropane, piperidine and pyridine alkaloid biosynthesis. In SC vs HB_MPP, key pathways included bile secretion, axon regeneration, one carbon pool by folate, and flavone and flavonol biosynthesis. For SC vs JS, the main pathways were biosynthesis of plant secondary metabolites, central carbon metabolism in cancer, zeatin biosynthesis, and protein digestion and absorption. SC vs SWJD showed enrichment in flavone and flavonol biosynthesis, biosynthesis of plant secondary metabolites, nucleotide metabolism, and biosynthesis of alkaloids derived from histidine and purine. In the SWJD vs GZ_LYM comparison, the dominant pathways were flavone and flavonol biosynthesis, biosynthesis of plant secondary metabolites, monoterpenoid biosynthesis, and cAMP signaling pathway. Lastly, SWJD vs HB_MPP was enriched mainly in flavone and flavonol biosynthesis, biosynthesis of plant secondary metabolites, axon regeneration, and degradation of flavonoids.

KEGG pathway enrichment analysis of differential metabolites among various fermented glutinous rice comparison groups revealed that metabolic activities in fermented glutinous rice are abundant and highly active. The enriched pathways were mainly concentrated in three core categories: biosynthesis of plant secondary metabolites, amino acid biosynthesis and metabolism, and lipid and ester metabolism. For amino acid biosynthesis and metabolism (flavor precursors), pathways such as valine, leucine and isoleucine biosynthesis and protein digestion and absorption were significantly enriched across multiple comparison groups, indicating active metabolism of amino acids and their derivatives in fermented glutinous rice. This not only provides a material basis for the generation of flavor precursors but also contributes to the characteristic flavor of fermented glutinous rice. Comparative analysis between GZ_LYM and HB_MPP revealed a significant enrichment of amino-acid-related pathways, accompanied by the up-accumulation of Gly-Leu-His, Thr-Pro-Leu, and trans-zeatin. These metabolite shifts imply a potential contribution to the formation and accumulation of flavor-active compounds. In parallel, pathways associated with lipid and ester metabolism—including bile secretion, choline metabolism,and central carbon metabolism (overlapping with energy and amino acid metabolism)-were markedly enriched. The elevated levels of acetyltributyl citrate and methylprednisolone acetate indicate active lipid turnover within the fermentation matrix, which is known to facilitate the generation of aroma-active esters. Consistent enrichment patterns observed in the JS vs GZ_LYM and SC vs HB_MPP comparisons further support the pivotal role of lipid metabolism in aroma development. Moreover, pronounced differences in saponin metabolites (e.g., astragaloside III, cappariloside B) between GZ_LYM and SC provide additional evidence linking saponin-associated lipid pathways to flavor formation in fermented glutinous rice.With regard to plant secondary metabolism, pathways related to plant secondary metabolite biosynthesis, flavonoid and flavonol biosynthesis, and plant hormone biosynthesis were significantly enriched across multiple comparison groups. Notably, the SWJD group exhibited the most pronounced enrichment in the flavonoid and flavonol biosynthesis pathways, characterized by increased levels of kaempferol 3-O-feruloyl-sophoroside 7-O-glucoside, swertiajaponin, and isowertin 2″-rhamnoside. This enrichment pattern is highly consistent with its strong antioxidant capacity and its elevated content of flavonoids and polyphenols. This indicates that plant secondary metabolites not only enhance the sensory properties of fermented glutinous rice but may also confer potential health benefits. Other comparison groups also showed co-enrichment of amino acid metabolism, lipid metabolism, and biosynthesis of plant secondary metabolites, reflecting the systematic nature of the metabolic network in fermented glutinous rice. In summary, metabolic activities in fermented glutinous rice are primarily concentrated in the biosynthesis of plant secondary metabolites, amino acid biosynthesis and metabolism, and lipid and ester metabolism. These metabolic characteristics directly influence the generation of flavor precursors, accumulation of aroma compounds, and antioxidant functions. Differences in these core metabolic pathways among fermented glutinous rice from different production regions provide a molecular basis for variations in sensory properties and bioactivity, and offer theoretical guidance for flavor and functional improvement.

### 3.6 Correlation among physicochemical properties, volatile compounds, and non-volatile compounds

**Fig. 6:**
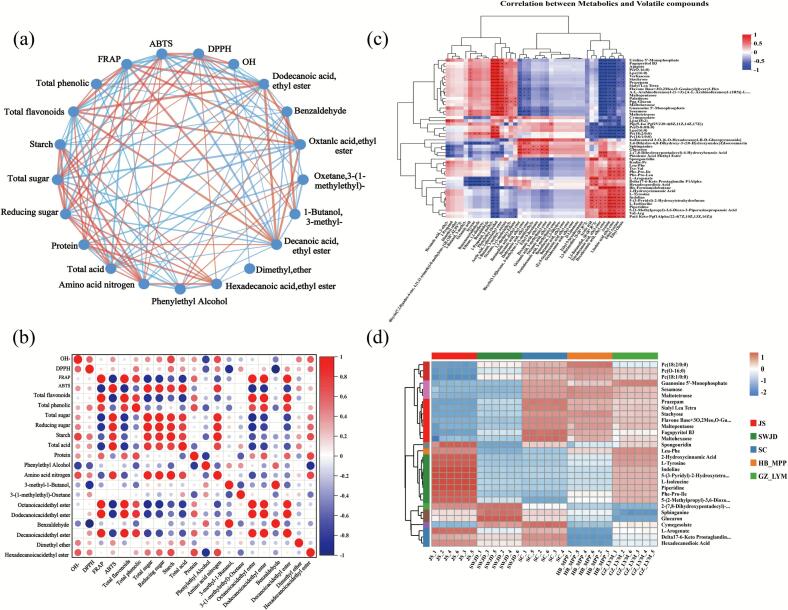
Correlation among physicochemical properties, volatile compounds, and non-volatile compounds (HB_MPP,SC,JS,GZ_LYM,SWJD). (a) Interaction network of physicochemical characteristics and volatile compounds in fermented glutinous rice. (b) Pearson correlation plot between physicochemical characteristics and volatile compounds in fermented glutinous rice. (c) Clustered heat map of volatile compounds versus non-volatile compounds The right axis displays non-volatile metabolite names (TOP 50), while the bottom axis lists volatile compounds. Each cell represents the correlation coefficient between corresponding compounds, with color intensity indicating the magnitude of correlation. * 0.01 < *p* ≤ 0.05, ** 0.001 < *p* ≤ 0.01, *** *p* ≤ 0.001. (d) Hierarchical Clustering Heatmap.

The integration of the interaction network (Fig. 6a) and Pearson correlation heatmap (Fig. 6b) revealed that key flavor compounds were strongly connected with total sugar, total phenols, total flavonoids, total acids, and proteins, highlighting their central roles in flavor and quality formation of fermented glutinous rice. Consistently, proteins and flavonoids showed positive correlations with ethyl octanoate and ethyl dodecanoate, supporting the contribution of physicochemical and antioxidant properties to flavor development. Starch, amino nitrogen, and reducing sugars were also linked with several esters (e.g., ethyl dodecanoate, ethyl hexadecanoate), indicating their function as precursors in Maillard and Strecker reactions. Strong correlations between reducing sugars and amino nitrogen (*r = 0.900*) and between starch and amino nitrogen (*r = 0.932*) further supported this mechanism.

The network also revealed associations between ester compounds (e.g., ethyl dodecanoate, ethyl decanoate) and antioxidant indices (FRAP, DPPH), suggesting shared metabolic origins. Pearson analysis confirmed the correlations between FRAP and ethyl dodecanoate (*r = 0.969*) as well as ethyl decanoate (*r = 0.904*). Correlation analysis between volatile and non-volatile metabolites (Fig. 6c) indicated three major metabolic routes: (1) Amino acid degradation, where branched-chain and aromatic amino acids yielded higher alcohols and aldehydes via Ehrlich and Strecker reactions (Liu et al., 2018); for example, 3-methyl-1-butanol correlated with L-tyrosine and L-isoleucine, while phenylethanol showed strong correlation with stachyose. (2) Fatty acid esterification, where medium- and long-chain ethyl esters correlated negatively with LPCs, indicating that phospholipid hydrolysis supplied free fatty acids for ester synthesis (Shi et al., 2025). (3) Phenolic metabolism, where benzaldehyde correlated with tyrosine and hydroxycinnamic acid, reflecting its origin from phenylalanine degradation and phenolic acid transformation, with potential contributions to antioxidant activity (Xing & Yaylayan, 2024).

Clustering analysis of non-volatile metabolites across five groups (JS, SWJD, SC, HB_MPP, GZ_LYM) (Fig. 6d) revealed clear group-specific profiles. JS showed tight clustering, whereas SWJD, SC, HB_MPP and GZ_LYM also clustered within groups but differed markedly among groups. Oligosaccharides (stachyose, verbascose, maltopentaose, maltotetraose, maltohexaose) and phospholipids (LPC(16:0), PC(18:1/0:0), PC(18:2/0:0)) were enriched in SC, GZ_LYM, and HB_MPP, suggesting contributions to sweetness, viscosity, and nutritional value. Amino acids and derivatives (L-tyrosine, L-isoleucine, Phe-Pro-Leu, Val-Arg) were abundant in JS and GZ_LYM, supporting their roles as flavor precursors. Flavonoid derivatives and nucleotides were enriched in SWJD, consistent with its antioxidant activity and umami characteristics.

Overall, these results demonstrate that major volatile flavor compounds in fermented glutinous rice are derived from interconnected metabolic pathways, primarily involving phospholipid hydrolysis-driven esterification, oligosaccharide and amino acid degradation, and microbial metabolism of short-chain fatty acids. These findings provide a mechanistic basis for quality regulation with a flavor-oriented perspective.

## 4 Conclusion

This study compared the antioxidant activity, physicochemical properties, and flavor-related metabolic characteristics of fermented glutinous rice from different regions of China. Significant variations were observed in amino acid composition, organic acid distribution, and antioxidant capacity among the samples. The volatile flavor profile was dominated by esters, alcohols, and acids, while metabolomics analysis revealed that the key differential metabolites were mainly classified as lipids and lipid-like molecules, organic acid derivatives, and heterocyclic compounds. These metabolites were significantly enriched in pathways related to amino acid metabolism, biosynthesis of plant secondary metabolites, and protein digestion and absorption. Moreover, strong correlations were identified among antioxidant activity, physicochemical parameters, key volatile compounds, and non-volatile metabolites. Overall, these findings highlight the synergistic interactions between physicochemical traits and metabolic constituents, which collectively drive the formation of flavor characteristics and quality attributes of fermented glutinous rice.

## CRediT authorship contribution statement

**Yu Pan:** Writing – original draft, Methodology, Conceptualization. **Keyun Lin:** Formal analysis, Data curation. **Jinxing Zeng:** Formal analysis, Data curation. **Yaya Zhou:** Validation, Investigation. **Diqin Yang:** Validation, Investigation. **Xia Zhu:** Validation, Formal analysis. **Minghui Shi:** Validation, Formal analysis. **Quanmin Sun:** Validation, Formal analysis. **Chengzhang Ou:** Formal analysis, Data curation. **Yu Wang:** Formal analysis, Data curation. **Yudan Xie:** Formal analysis, Data curation. **Shuying Yang:** Writing – review & editing, Visualization, Supervision, Resources, Project administration. **Kuan Lu:** Writing – review & editing, Visualization, Supervision, Resources, Project administration.

## Declaration of competing interest

The authors declare that there are no known financial or personal conflicts of interest that could have influenced the work reported in this paper. All authors have approved the final manuscript and agree with its submission to Food Chemistry:X.

## Data Availability

The data that support the findings of this study are available from the corresponding author upon reasonable request. The data reported in this paper have been deposited in the OMIX, China National Center for Bioinformation/Beijing Institute of Genomics, Chinese Academy of Sciences (https://ngdc.cncb.ac.cn/omix:accession no.OMIX011380).

## Appendix A Supplementary data

Original raw data of all assays (Relative Abundance of Volatile Compounds, Differential Metabolites with the Highest VIP Scores, Complete metabolite list, Full GC–MS peak table with NIST match scores and retention indices, Amino Acid Score Chart, QC validation results, ion mode OPLS-DA permutation test (1000 permutations), HPLC chromatogram of organic acid, Total Ion Chromatogram, Full-size heatmap). Image 1
