# Supplementary material for: Nutritional evaluation, characteristic component differences and non-targeted metabolomics analysis of traditional fermented glutinous Rice in four Chinese provinces
Source: Food Chem X. 2026 Jan 13;33:103534. doi: 10.1016/j.fochx.2026.103534 (PMC12853045; doi:10.1016/j.fochx.2026.103534)
Supplement: Supplementary file 1 — Original raw data of all assays (Relative Abundance of Volatile Compounds, Differential Metabolites with the Highest VIP Scores, Complete metabolite list, Full GC–MS peak table with NIST match scores and retention indices, Amino Acid Score Chart, QC validation results, ion mode OPLS-DA permutation test (1000 permutations), HPLC chromatogram of organic acid, Total Ion Chromatogram, Full-size heatmap). [file mmc1.pdf]

Supplementary Materials

Nutritional Evaluation, Characteristic Component Differences and Non-targeted Metabolomics Analysis of Traditional Fermented Glutinous Rice in four Chinese Provinces

Yu Pan[a], Keyun Lin[a], Jingxin Zeng[a], Yaya Zhou[a], Diqin Yang[a], Xia Zhu[a], Minghui Shi[a], Quanmin Sun[a], Chengzhang Ou[a], Yu Wang[a], Yudan Xie[a], Shuying Yang[a, b], Kuan Lu[a*]

[a]Guizhou Biotechnology Research and Development Base Co., Ltd., Guiyang 550014, P. R. China.

[b]Guizhou Kehui Inspection and Testing Research Institute Co., Ltd., Guiyang 550014, P. R. China.

*Corresponding Authors: Kuan Lu

E-mail address:wukong4608@163.com

CONTEST

Table S1 Relative Abundance of Volatile Compounds 2
Table S2 Top 14 Differential Metabolites with the Highest VIP Scores between GZ_LYM and HB_MPP 3
Table S3 Top 14 Differential Metabolites with the Highest VIP Scores between JS and GZ_LYM 4
Table S4 Top 14 Differential Metabolites with the Highest VIP Scores between JS and HB_MPP 5
Table S5 Top 14 Differential Metabolites with the Highest VIP Scores between JS and SWJD 6
Table S6 Top 14 Differential Metabolites with the Highest VIP Scores between SC and GZ_LYM 7
Table S7 Top 14 Differential Metabolites with the Highest VIP Scores between SC and HB_MPP 8
Table S8 Top 14 Differential Metabolites with the Highest VIP Scores between SC and JS 9
Table S9 Top 14 Differential Metabolites with the Highest VIP Scores between SC and SWJD 10
Table S10 Top 14 Differential Metabolites with the Highest VIP Scores between SWJD and GZ_LYM 11
Table S11 Top 14 Differential Metabolites with the Highest VIP Scores between SWJD and HB_MPP 12
Table S12 Full GC–MS peak table with NIST match scores and retention indices 13
Table S13 Complete metabolite list 13
Table S14 Full GC–MS peak table with NIST match scores and retention indices 63
Fig. S1 Amino Acid Score Chart 171
Fig. S2 QC validation results (TIC overlays,Positive ion mode) 172
Fig. S3 QC validation results (TIC overlays,Negative ion mode) 173
Fig. S4 QC validation results (PCA showing QC clusterings,Positive ion mode) 174
Fig. S5 QC validation results (PCA showing QC clusterings,Negative ion mode) 175
Fig. S6 Positive ion mode OPLS-DA permutation test (1000 permutations) 176
Fig. S7 Negative ion mode OPLS-DA permutation test (1000 permutations) 177
Fig. S8 HPLC chromatogram of organic acid 178
Fig. S9 Total Ion Chromatogram (TIC)(a,HB_MPP;b,SC;c,JS;d:GZ_LYM;e,SWJD) 179
Fig. S10 Full-size heatmap 180

Table S1 Relative Abundance of Volatile Compounds

No.	Volatile Compounds	CAS	Retention Time/（min）	Relative Content（%）				
				HB_MPP	SC	JS	GZ_LYM	SWJD
A1	Ethyl Acetate	141-78-6	4.5551	1.92±0.26	1.91±017	3.78±0.35	1.43±0.32	1.15±0.07
A2	Butanoic acid, ethyl ester	105-54-4	7.0054	-	-	-	-	0.05±0.09
A3	1-Propanol, 2-methyl-	78-83-1	8.0086	2.21±0.07	1.89±0.09	0.76±0.04	1.74±0.42	1.46±0.09
A4	1-Butanol, 3-methyl-, acetate	123-92-2	8.5175	0.46±0.12	1.29±0.21	0.52±0.04	0.58±0.13	0.11±0.10
A5	Ethane, 1,1-diethoxy-	105-57-7	4.6286	0.80±0.09	-	-	-	-
A6	Bicyclo[3.1.0]hexane, 4-methylene-1-(1-methylethyl)-	3387-41-5	8.1994	-	-	-	-	0.11±0.01
A7	Oxetane, 3-(1-methylethyl)-	10317-17-6	9.9619	4.10±0.00	11.54±0.17	2.99±2.59	9.58±2.10	6.01±0.26
A8	D-Limonene	5989-27-5	9.8069	-	-	-	-	0.04±0.00
A9	1-Butanol, 3-methyl-	123-51-3	9.9623	8.46±0.33	-	1.66±0.28	-	-
A10	Hexanoic acid, ethyl ester	123-66-0	10.3621	0.82±0.17	0.72±0.11	0.71±0.08	0.68±0.14	2.21±0.26
A11	Styrene	100-42-5	10.7721	0.28±0.06	-	-	-	-
A12	Acetoin	513-86-0	11.2403	0.12±0.07	-	0.66±0.03	-	-
A13	Octanoic acid, ethyl ester	106-32-1	13.3011	5.45±1.04	4.68±0.72	4.60±0.53	5.87±1.14	8.46±0.81
A14	Acetic acid	64-19-7	13.4892	0.61±0.04	1.39±0.09	1.61±0.10	1.40±0.33	0.55±0.17
A15	Benzaldehyde	100-52-7	14.638	2.56±0.16	-	-	-	-
A16	2,3-Butanediol, [R-(R*,R*)]-	24347-58-8	15.1438	0.23±0.04	1.50±0.73	1.92±0.18	1.13±0.62	0.27±0.00
A17	Decanoic acid, ethyl ester	110-38-3	15.8957	3.94±0.75	6.91±0.94	7.01±0.38	7.44±1.43	14.04±1.05
A18	Octanoic acid, 3-methylbutyl ester	2035-99-6	16.1487	-	-	-	-	0.21±0.02
A19	Benzeneacetaldehyde	122-78-1	16.0752	0.39±0.06	-	-	-	-
A20	Acetic acid, 2-phenylethyl ester	103-45-7	18.0757	1.47±0.11	2.45±0.19	1.09±0.06	2.14±0.39	0.68±0.05
A21	Phenylethyl Alcohol	1960/12/8	19.1171	46.99±0.43	44.30±0.92	21.87±0.67	39.02±8.2<br>0	30.37±0.86
A22	Tetradecanoic acid, ethyl ester	124-06-1	20.358	2.05±0.13	1.53±0.12	5.18±0.23	1.47±0.29	-
A23	Octanoic acid	124-07-2	20.465	0.73±0.04	-	-	-	-
A24	Hexadecanoic acid, ethyl ester	628-97-7	22.3175	10.77±0.10	12.48±1.33	32.26±1.05	8.09±3.19	18.13±1.32
A25	Ethyl Oleate	111-62-6	24.8584	2.13±0.08	2.24±0.20	4.37±0.58	2.60±0.78	2.07±1.83
A26	Linoleic acid ethyl ester	544-35-4	25.5495	2.87±0.05	2.95±0.32	6.51±0.80	3.19±1.03	3.44±0.74
A27	Dodecanoic acid, ethyl ester	106-33-2	18.2359	0.71±0.62	1.31±0.13	1.59±0.05	1.76±0.30	4.02±0.13
A28	Butanedioic acid, diethyl ester	123-25-1	16.3565	-	-	-	-	2.10±0.04
A29	Ethyl 9-decenoate	67233-91-4	16.5274	-	-	-	-	0.18±0.17
A30	Pentadecanoic acid, 3-methylbutyl ester	2306-91-4	18.4455	-	-	-	-	0.05±0.08
A31	Benzofuran, 2,3-dihydro-	496-16-2	23.8453	-	-	-	-	0.12±0.21
A32	(E)-9-Octadecenoic acid ethyl ester	6114-18-7	24.8542	-	-	-	-	0.77±0.34
A33	Ethyl 9-hexadecenoate	54546-22-4	22.6049	-	-	-	-	0.10±0.01
A34	Octadecanoic acid, ethyl ester	111-61-5	24.547	-	-	-	-	0.13±0.03
A35	1-Propanol	71-23-8	7.1056	-	-	-	0.35±0.35	-
A36	Hexanoic acid, 2-ethyl-	149-57-5	19.3583	-	-	-	0.76±0.15	-
A37	Dimethyl ether	115-10-6	5.4042	-	0.16±0.08	0.07±0.00	0.06±0.10	-
A38	Bicyclo[7.2.0]undec-4-ene, 4,11,11-trimethyl-8-methylene-,[1R-(1R*,4Z,9S *)]-	118-65-0	15.628	-	-	-	0.06±0.10	-
A39	2,3-Butanediol, [S-(R*,R*)]-	19132-06-0	14.7012	-	-	0.39±0.00	-	-
A40	Ethane, 1,1-diethoxy-	105-57-7	4.6299	-	0.74±0.04	-	-	-

‘-’indicates not detected. Data are presented as mean ± standard deviation.

Table S2 Top 14 Differential Metabolites with the Highest VIP Scores between GZ_LYM and HB_MPP

Mode	GZ_LYM VS HB_MPP		
	Metabolite	VIP_pred_OPLS-DA	Regulate
POS	Ala-Thr-Trp-Leu-Pro-Pro-Arg	3.358	down
	Hovenidulcioside B1	2.8934	up
	Tyr-Glu-Arg	2.8682	down
	Pyroglutamylproline	2.7918	up
	Brevetoxin B2	2.775	down
	Cysteinylglycine	2.6857	down
	Gly-Leu-His	2.5752	up
	Spirolide B	2.5651	up
	Methylprednisolone Acetate	2.5446	up
	Thr-Pro-Leu	2.5401	up
	4'-O-Methyl-(-)-Epicatechin 3'-O-Glucuronide	2.5143	up
	Neolinustatin	2.4969	up
	Acetyltributyl Citrate	2.3707	up
	Cer(8:0_2O/14:0)	2.3585	down
NEG	Dioscin	3.7846	down
	5-Alpha-Dihydrotestosterone Glucuronide	3.4414	down
	Etamucine	3.3234	up
	Motexafin	3.1954	up
	Ophiopogonin C	2.9798	down
	Troleandomycin	2.9719	down
	3-Hydroxy-Beta-Ionol 3-[Glucosyl-(1->6)-Glucoside]	2.9692	up
	Pantetheine	2.9653	down
	(23S)-23,25-Dihdroxy-24-Oxovitamine D3 23-(Beta-Glucuronide)	2.9613	down
	Trans-Zeatin	2.8248	up
	S-Glutaryldihydrolipoamide	2.7698	up
	23-Trans-P-Coumaroyloxytormentic Acid	2.7556	down
	3-Deoxy-D-Manno-Octulosonate	2.7501	down
	Actein	2.6595	down

Table S3 Top 14 Differential Metabolites with the Highest VIP Scores between JS and GZ_LYM

Mode	JS VS GZ_LYM		
	Metabolite	VIP_pred_OPLS-DA	Regulate
POS	Hovenidulcioside B1	2.7997	down
	5-(Furan-2-Carbonyl)-9-(Hydroxymethyl)-1,5-Diazacycloundecan-2-One	2.7292	up
	Strictosidine	2.5416	up
	Imidazoleacetic Acid Riboside	2.4999	up
	4'-O-Methyl-(-)-Epicatechin 3'-O-Glucuronide	2.4703	down
	Vinorelbine Base	2.4498	up
	Fagomine	2.4368	up
	Pc(20:5/0:0)	2.4354	up
	Cappariloside B	2.4214	down
	Lys-Trp	2.3963	up
	Tyr-Phe-Glu-Lys	2.394	down
	Iodamide	2.3623	down
	Physangulide	2.3447	down
	1-(3,5-Dimethylbenzoyl)-N-(3-Methylsulfanylphenyl)Piperidine-3-Carboxamide	2.2858	up
NEG	Pantetheine	2.7405	up
	Glucose 1-Phosphate	2.6639	down
	Lucidenic Acid A	2.6139	up
	Licoricesaponin F3	2.4869	down
	Gimatecan	2.482	up
	Trp-Tyr	2.4058	up
	3-Deoxy-D-Manno-Octulosonate	2.3784	up
	Cefminox	2.3621	down
	(E)-N-(2-Amino-4-Fluorophenyl)-3-(1-Cinnamyl-1H-Pyrazol-4-Yl)Acrylamide	2.3371	up
	Pa(Pgf2Alpha/22:1(13Z))	2.3314	down
	Goldinodox	2.3191	down
	Ramiprilat	2.2999	down
	3-(((4-Chlorophenyl)Sulfonyl)Methyl)-N-Hydroxybenzimidamide	2.2883	down
	Melatonin Glucuronide	2.2695	down

Table S4 Top 14 Differential Metabolites with the Highest VIP Scores between JS and HB_MPP

Mode	JS VS HB_MPP		
	Metabolite	VIP_pred_OPLS-DA	Regulate
POS	Ala-Thr-Trp-Leu-Pro-Pro-Arg	2.6292	down
	Strictosidine	2.3209	up
	Pyroglutamylproline	2.3085	up
	Imidazoleacetic Acid Riboside	2.3076	up
	Fagomine	2.2544	up
	Pc(20:5/0:0)	2.2379	up
	Alpha-L-Rhamnopyranosyl-(1->3)-Alpha-D-Galactopyranosyl-(1->3)-L-Fucose	2.237	up
	Leonurine	2.2077	up
	Asn-Asp-Val	2.2074	up
	Lys-Trp	2.2052	up
	Tyr-Phe-Glu-Lys	2.1981	down
	Montirelin	2.1699	up
	S-Adenosylmethionine	2.088	up
	Physangulide	2.0817	down
NEG	Dioscin	2.6247	down
	Ophiopogonin C	2.4006	down
	Licoricesaponin F3	2.3533	down
	Gimatecan	2.3222	up
	Etamucine	2.3038	up
	Glucose 1-Phosphate	2.2886	down
	Actein	2.2553	down
	Goldinodox	2.2364	down
	Rosamicin	2.2066	up
	Motexafin	2.1756	up
	5-Alpha-Dihydrotestosterone Glucuronide	2.1734	down
	Semustine	2.1423	up
	Pa(Pgf2Alpha/22:1(13Z))	2.1002	down
	Ganosporeric Acid A	2.0739	down

Table S5 Top 14 Differential Metabolites with the Highest VIP Scores between JS and SWJD

Mode	JS VS SWJD		
	Metabolite	VIP_pred_OPLS-DA	Regulate
POS	Hesperidin Methylchalcone	2.9128	down
	1-Ethyl 4-(2-Oxo-1,2-Diphenylethyl) Succinate	2.6343	down
	Skimmin	2.5162	down
	Imidazoleacetic Acid Riboside	2.5087	up
	Dide-O-Methyl-4-O-Alpha-D-Glucopyranosylsimmondsin	2.4914	down
	Dg(Pgd2/A-17:0/0:0)	2.4446	down
	Lamiidoside	2.4377	down
	4-Hydroxycoumarin	2.3887	down
	Pc(20:5/0:0)	2.3686	up
	Physcion 8-Gentiobioside	2.355	down
	Gmp-N-Epsilon-(N-Alpha-Acetyl Lysine Methyl Ester) 5'-Phosphoramidate	2.3469	down
	Ile-Trp	2.3298	up
	Gibberellin A50	2.313	down
	Fagomine	2.2895	up
NEG	Pa(Pgf2Alpha/22:1(13Z))	2.6158	down
	Kanokoside A	2.5995	down
	Cucumopine	2.5468	down
	Verbascoside	2.5436	down
	(R)-1-O-[B-D-Apiofuranosyl-(1->2)-B-D-Glucopyranoside]-1,3-Octanediol	2.4898	up
	Isowertin 2''-Rhamnoside	2.4734	down
	Kaempferol 3-O-Feruloyl-Sophoroside 7-O-Glucoside	2.4612	down
	Scutellarioside Ii	2.4295	down
	Echinacoside	2.3696	down
	Andromedoside	2.3692	down
	Histidyltyrosine	2.3591	down
	Swertiajaponin	2.3448	down
	Granotapide	2.3356	down
	4-O-P-Coumaroylquinic Acid	2.3164	down

Table S6 Top 14 Differential Metabolites with the Highest VIP Scores between SC and GZ_LYM

Mode	SC VS GZ_LYM		
	Metabolite	VIP_pred_OPLS-DA	Regulate
POS	Rphdhd	3.4295	up
	4'-O-Methyl-(-)-Epicatechin 3'-O-Glucuronide	3.1015	down
	Cappariloside B	3.06	down
	Pyroglutamylproline	3.0589	down
	Gly-Gly-Phe	2.8526	down
	Astragaloside Iii	2.6445	down
	Cysteinylglycine	2.6307	up
	(+/-)-Win 55,212	2.6156	down
	N(6)-Hydroxymethyladenosine	2.6125	down
	Gly Cys Val	2.4486	down
	2'-(E)-Feruloyl-3-(Arabinosylxylose)	2.4377	down
	Spb(20:0_2O)	2.4122	up
	Niazicinin	2.4078	down
	Methionine Sulfoxide	2.3924	up
NEG	Pantetheine	2.9711	up
	Fluocortolone Caproate	2.9518	down
	Gamma-Aminobutyric Acid Glutamate	2.8117	down
	Melatonin Glucuronide	2.6648	down
	Pa(Pgf2Alpha/22:1(13Z))	2.6465	down
	Gimatecan	2.585	up
	Thesinine 4'-O-Glucoside	2.5774	down
	Euglobal Ia1	2.5774	down
	3-Deoxy-D-Manno-Octulosonate	2.5357	up
	Lupinic Acid	2.5295	down
	2-Hydroxy-Desipramine Glucuronide	2.5078	down
	(E)-N-(2-Amino-4-Fluorophenyl)-3-(1-Cinnamyl-1H-Pyrazol-4-Yl)Acrylamide	2.4722	up
	Trans-Zeatin	2.425	down
	2,3-Dinor-Txb2	2.4039	down

Table S7 Top 14 Differential Metabolites with the Highest VIP Scores between SC and HB_MPP

Mode	SC VS HB_MPP		
	Metabolite	VIP_pred_OPLS-DA	Regulate
POS	Ala-Thr-Trp-Leu-Pro-Pro-Arg	3.4318	down
	Acetyltributyl Citrate	3.2427	up
	N(6)-Hydroxymethyladenosine	2.9676	down
	Gly-Gly-Phe	2.8745	down
	Cappariloside B	2.8637	down
	Brevetoxin B2	2.8065	down
	Tyr-Glu-Arg	2.7152	down
	Neolinustatin	2.6332	up
	Mg(15:0)	2.5344	up
	Prunin 6"-O-Gallate	2.5132	up
	Gibberellin A50	2.4996	down
	Astragaloside Iii	2.4403	down
	Quercetin 7-Glucoside	2.336	up
	Tricin	2.2675	up
NEG	Troleandomycin	3.5341	down
	Dioscin	3.5286	down
	Actein	3.2387	down
	Gamma-Aminobutyric Acid Glutamate	3.0029	down
	Fluocortolone Caproate	2.9841	down
	Motexafin	2.9061	up
	Gimatecan	2.8956	up
	Etamucine	2.8845	up
	Pa(Pgf2Alpha/22:1(13Z))	2.8589	down
	Pa(5-Iso Pgf2Vi/18:3(9Z,12Z,15Z))	2.6973	up
	Phe-Phe-Lys	2.6611	down
	Melatonin Glucuronide	2.6399	down
	Euglobal Ia1	2.6339	down
	Taurocholic Acid 3-Sulfate	2.6155	up

Table S8 Top 14 Differential Metabolites with the Highest VIP Scores between SC and JS

Mode	SC VS JS		
	Metabolite	VIP_pred_OPLS-DA	Regulate
POS	Pyroglutamylproline	2.6676	down
	Asn-Asp-Val	2.5604	down
	Gly-Cys-Val	2.5112	down
	Leonurine	2.4394	down
	Imidazoleacetic Acid Riboside	2.3929	down
	His-Ile	2.3848	down
	Hovenidulcioside B1	2.3784	up
	Gly-Gly-Phe	2.352	down
	Vinorelbine Base	2.28	down
	5-(Furan-2-Carbonyl)-9-(Hydroxymethyl)-1,5-Diazacycloundecan-2-One	2.2427	down
	(+/-)-Win 55,212	2.1538	down
	Valganciclovir, (S)-	2.1367	down
	Tyr-Glu-Arg	2.1016	down
	Fagomine	2.0658	down
NEG	Gamma-Aminobutyric Acid Glutamate	2.4218	down
	Euglobal Ia1	2.3688	down
	Glucose 1-Phosphate	2.3628	up
	Thesinine 4'-O-Glucoside	2.2995	down
	Licoricesaponin F3	2.2696	up
	Ophiopogonin C	2.2616	up
	Trp-Tyr-His	2.1718	down
	Goldinodox	2.166	up
	2,3-Butanediol Glucoside	2.1236	down
	Butirosina	2.1158	down
	Semustine	2.1145	down
	Lys-Asn-Asn	2.0862	down
	Lucidenic Acid A	2.0683	down
	Ala-His-Tyr	2.0515	down

Table S9 Top 14 Differential Metabolites with the Highest VIP Scores between SC and SWJD

Mode	SC VS SWJD		
	Metabolite	VIP_pred_OPLS-DA	Regulate
POS	Styraxlignolide F	3.0983	down
	Hesperidin Methylchalcone	3.0964	down
	1-Ethyl 4-(2-Oxo-1,2-Diphenylethyl) Succinate	3.0167	down
	Valganciclovir, (S)-	3.0137	down
	Dide-O-Methyl-4-O-Alpha-D-Glucopyranosylsimmondsin	2.9591	down
	Gibberellin A50	2.8513	down
	Skimmin	2.8223	down
	Lamiidoside	2.7093	down
	4'-O-Beta-D-Glucosyl-5-O-Methylvisamminol	2.6704	down
	4-Hydroxycoumarin	2.6587	down
	Leonurine	2.6439	down
	Gmp-N-Epsilon-(N-Alpha-Acetyl Lysine Methyl Ester) 5'-Phosphoramidate	2.6131	down
	[(2E,6E)-1-Oxo-8-Hydroxy-2,6-Dimethylocta-2,6-Dien-1-Yl]Beta-D-Glucopyranoside	2.5604	down
	Physcion 8-Gentiobioside	2.5454	down
NEG	Pa(Pgf2Alpha/22:1(13Z))	3.0911	down
	Scutellarioside Ii	3.031	down
	Kanokoside A	3.0267	down
	Cucumopine	2.9792	down
	Kaempferol 3-O-Feruloyl-Sophoroside 7-O-Glucoside	2.9205	down
	5-(Delta-Carboxybutyl)Homocysteine	2.8419	down
	Ganoderiol I	2.8227	down
	Andromedoside	2.8074	down
	Swertiajaponin	2.7972	down
	Plantamajoside	2.7889	down
	Dextran-70	2.7694	down
	Licoricesaponin F3	2.7574	up
	Granotapide	2.748	down
	10-Acetoxyligustroside	2.7419	down

Table S10 Top 14 Differential Metabolites with the Highest VIP Scores between SWJD and GZ_LYM

Mode	SWJD VS GZ_LYM		
	Metabolite	VIP_pred_OPLS-DA	Regulate
POS	1-Ethyl 4-(2-Oxo-1,2-Diphenylethyl) Succinate	2.7352	up
	Styraxlignolide F	2.6032	up
	Hesperidin Methylchalcone	2.5795	up
	Glycyrrhizin	2.5712	down
	Ryanodine	2.5689	down
	Dide-O-Methyl-4-O-Alpha-D-Glucopyranosylsimmondsin	2.5059	up
	Cichorioside J	2.5004	up
	Cappariloside B	2.4476	down
	Cysteinylglycine	2.4309	up
	4'-O-Beta-D-Glucosyl-5-O-Methylvisamminol	2.4005	up
	Skimmin	2.3997	up
	Coagulin R 3-Glucoside	2.3474	down
	Physcion 8-Gentiobioside	2.3373	up
	N-Cyclopentyl-2-(Diethylamino)-2-(6-Nitro-1,3-Benzodioxol-5-Yl)Acetamide	2.3242	down
NEG	Plantamajoside	2.6687	up
	Pantetheine	2.6668	up
	Kanokoside A	2.6073	up
	Cucumopine	2.5625	up
	Isowertin 2''-Rhamnoside	2.5149	up
	Kaempferol 3-O-Feruloyl-Sophoroside 7-O-Glucoside	2.487	up
	Scutellarioside Ii	2.4858	up
	Fluocortolone Caproate	2.4478	down
	Indole-3-Acetylglutamic Acid	2.4161	up
	Gimatecan	2.4158	up
	Andromedoside	2.3932	up
	Ganoderiol I	2.3736	up
	Licoricesaponin F3	2.3686	down
	Ethyl 4-Hydroxy-3-Methylisoxazolo[5,4-B]Pyridine-5-Carboxylate	2.3453	down

Table S11 Top 14 Differential Metabolites with the Highest VIP Scores between SWJD and HB_MPP

Mode	SWJD VS HB_MPP		
	Metabolite	VIP_pred_OPLS-DA	Regulate
POS	Hesperidin Methylchalcone	2.872	up
	Styraxlignolide F	2.8689	up
	1-Ethyl 4-(2-Oxo-1,2-Diphenylethyl) Succinate	2.8049	up
	Valganciclovir, (S)-	2.7801	up
	Dide-O-Methyl-4-O-Alpha-D-Glucopyranosylsimmondsin	2.7371	up
	Rphdhd	2.662	down
	Ala-Thr-Trp-Leu-Pro-Pro-Arg	2.6109	down
	Skimmin	2.5995	up
	Glycyrrhizin	2.5155	down
	Lamiidoside	2.4964	up
	S-Adenosylmethionine	2.446	up
	4-Hydroxycoumarin	2.4457	up
	Cichorioside J	2.4408	up
	Gmp-N-Epsilon-(N-Alpha-Acetyl Lysine Methyl Ester) 5'-Phosphoramidate	2.4028	up
NEG	Plantamajoside	2.7977	up
	Dioscin	2.7333	down
	Kanokoside A	2.732	up
	Scutellarioside Ii	2.7169	up
	Cucumopine	2.6816	up
	Kaempferol 3-O-Feruloyl-Sophoroside 7-O-Glucoside	2.6085	up
	5-(Delta-Carboxybutyl)Homocysteine	2.5406	up
	Isowertin 2''-Rhamnoside	2.5321	up
	Gimatecan	2.5297	up
	Andromedoside	2.5096	up
	Licoricesaponin F3	2.507	down
	Verbascoside	2.5013	up
	Indole-3-Acetylglutamic Acid	2.4953	up
	Swertiajaponin	2.4908	up

Table S12 Full GC–MS peak table with NIST match scores and retention indices

	Retention Time	Compound Name	Match Factor	Molecular Formula	CAS Number	Library Weight	Molecular
HB_MPP	4.5551	Ethyl Acetate	94.9694	$C_4H_8O_2$	141-78-6	88.0520	
	8.0086	1-Propanol, 2-methyl-	96.2996	$C_4H_{10}O$	78-83-1	74.0730	
	8.5175	1-Butanol, 3-methyl-, acetate	95.2545	$C_7H_{14}O_2$	123-92-2	130.0990	
	9.9619	Oxetane, 3-(1-methylethyl)-	92.1470	$C_6H_{12}O$	10317-17-6	100.0890	
	10.3621	Hexanoic acid, ethyl ester	96.8933	$C_8H_{16}O_2$	123-66-0	144.1150	
	10.7721	Styrene	95.6823	C_8H_8	100-42-5	104.0630	
	11.2403	Acetoin	94.3320	C_4H_8O2	513-86-0	88.0520	
	13.3011	Octanoic acid, ethyl ester	94.9619	$C_{10}H_{20}O_2$	106-32-1	172.1460	
	13.4892	Acetic acid	96.3677	$C_2H_4O_2$	64-19-7	60.0210	
	14.6380	Benzaldehyde	93.4603	C_7H_6O	100-52-7	106.0420	
	15.1438	2,3-Butanediol, [R-(R*,R*)]-	90.6618	$C_4H_{10}O_2$	24347-58-8	90.0680	
	15.8957	Decanoic acid, ethyl ester	96.9137	$C_{12}H_{24}O_2$	110-38-3	200.1780	
	16.0752	Benzeneacetaldehyde	93.2977	C_8H_8O	122-78-1	120.0580	
	18.0757	Acetic acid, 2-phenylethyl ester	94.9378	$C_{10}H_{12}O_2$	103-45-7	164.0840	
	19.1171	Phenylethyl Alcohol	96.9647	$C_8H_{10}O$	60-12-8	122.0730	
	20.3580	Tetradecanoic acid, ethyl ester	96.2982	$C_{16}H_{32}O_2$	124-06-1	256.2400	
	20.4650	Octanoic acid	93.9371	$C_8H_{16}O_2$	124-07-2	144.1150	
	22.3175	Hexadecanoic acid, ethyl ester	94.1002	$C_{18}H_{36}O_2$	628-97-7	284.2720	
	24.8584	Ethyl Oleate	93.0289	$C_{20}H_{38}O_2$	111-62-6	310.2870	
	25.5495	Linoleic acid ethyl ester	94.6559	$C_{20}H_{36}O_2$	544-35-4	308.2720	
SC	4.5539	Ethyl Acetate	95.8809	$C_4H_8O_2$	141-78-6	88.0520	
	8.0178	1-Propanol, 2-methyl-	96.3103	$C_4H_{10}O$	78-83-1	74.0730	
	8.5145	1-Butanol, 3-methyl-, acetate	96.8636	$C_7H_{14}O_2$	123-92-2	130.0990	
	9.9619	Oxetane, 3-(1-methylethyl)-	91.4028	$C_6H_{12}O$	10317-17-6	100.0890	
	10.3612	Hexanoic acid, ethyl ester	94.8826	$C_8H_{16}O_2$	123-66-0	144.1150	
	13.3007	Octanoic acid, ethyl ester	96.0510	$C_{10}H_{20}O_2$	106-32-1	172.1460	
	13.4839	Acetic acid	97.8887	$C_2H_4O_2$	64-19-7	60.0210	
	14.7005	2,3-Butanediol, [R-(R*,R*)]-	93.0764	$C_4H_{10}O_2$	24347-58-8	90.0680	
	15.1432	2,3-Butanediol, [R-(R*,R*)]-	92.4144	$C_4H_{10}O_2$	24347-58-8	90.0680	
	15.8957	Decanoic acid, ethyl ester	95.9198	$C_{12}H_{24}O_2$	110-38-3	200.1780	
	18.0739	Acetic acid, 2-phenylethyl ester	95.2049	$C_{10}H_{12}O_2$	103-45-7	164.0840	
	18.2311	Dodecanoic acid, ethyl ester	93.2403	$C_{14}H_{28}O_2$	106-33-2	228.2090	
	19.1154	Phenylethyl Alcohol	97.0948	$C_8H_{10}O$	60-12-8	122.0730	
	20.3572	Tetradecanoic acid, ethyl ester	96.0981	$C_{16}H_{32}O_2$	124-06-1	256.2400	
	22.3154	Hexadecanoic acid, ethyl ester	94.2687	$C_{18}H_{36}O_2$	628-97-7	284.2720	
	24.8548	Ethyl Oleate	92.9356	$C_{20}H_{38}O_2$	111-62-6	310.2870	
	25.5451	Linoleic acid ethyl ester	94.1803	$C_{20}H_{36}O_2$	544-35-4	308.2720	
JS	4.5544	Ethyl Acetate	96.8842	$C_4H_8O_2$	141-78-6	88.0520	
	6.9935	Dimethyl ether	95.1924	C_2H_6O	115-10-6	46.0420	
	8.0151	1-Propanol, 2-methyl-	94.5706	$C_4H_{10}O$	78-83-1	74.0730	
	8.5135	1-Butanol, 3-methyl-, acetate	95.8846	$C_7H_{14}O_2$	123-92-2	130.0990	
	9.9579	Oxetane, 3-(1-methylethyl)-	91.3312	$C_6H_{12}O$	10317-17-6	100.0890	
	10.3611	Hexanoic acid, ethyl ester	95.4828	$C_8H_{16}O_2$	123-66-0	144.1150	
	11.2426	Acetoin	94.9424	$C_4H_8O_2$	513-86-0	88.0520	
	13.3010	Octanoic acid, ethyl ester	94.9155	$C_{10}H_{20}O_2$	106-32-1	172.1460	
	13.4806	Acetic acid	97.4523	$C_2H_4O_2$	64-19-7	60.0210	
	14.7013	2,3-Butanediol, [R-(R*,R*)]-	92.7275	$C_4H_{10}O_2$	24347-58-8	90.0680	
	15.1429	2,3-Butanediol, [R-(R*,R*)]-	94.9422	$C_4H_{10}O_2$	24347-58-8	90.0680	
	15.8963	Decanoic acid, ethyl ester	96.0875	$C_{12}H_{24}O_2$	110-38-3	200.1780	
	16.3575	Butanedioic acid, diethyl ester	94.1696	$C_8H_{14}O_4$	123-25-1	174.0890	
	18.0748	Acetic acid, 2-phenylethyl ester	91.0063	$C_{10}H_{12}O_2$	103-45-7	164.0840	
	18.2313	Dodecanoic acid, ethyl ester	94.3067	$C_{14}H_{28}O_2$	106-33-2	228.2090	
	19.1151	Phenylethyl Alcohol	97.2556	$C_8H_{10}O$	60-12-8	122.0730	
	20.3586	Tetradecanoic acid, ethyl ester	96.7241	$C_{16}H_{32}O_2$	124-06-1	256.2400	
	22.3253	Hexadecanoic acid, ethyl ester	93.8867	$C_{18}H_{36}O_2$	628-97-7	284.2720	
	24.8561	Ethyl Oleate	93.8826	$C_{20}H_{38}O_2$	111-62-6	310.2870	
	25.5492	Linoleic acid ethyl ester	94.8593	$C_{20}H_{36}O_2$	544-35-4	308.2720	
GZ_LYM	4.5542	Ethyl Acetate	94.9469	$C_4H_8O_2$	141-78-6	88.0520	
	7.1056	1-Propanol	92.0836	C_3H_8O	71-23-8	60.0580	
	8.0469	1-Propanol, 2-methyl-	96.0841	$C_4H_{10}O$	78-83-1	74.0730	
	8.5024	1-Butanol, 3-methyl-, acetate	92.0547	$C_7H_{14}O_2$	123-92-2	130.0990	

	9.9647	Oxetane, 3-(1-methylethyl)-	91.0746	$C_6H_{12}O$	10317-17-6	100.0890
	10.3545	Hexanoic acid, ethyl ester	95.7601	$C_8H_{16}O_2$	123-66-0	144.1150
	13.2992	Octanoic acid, ethyl ester	94.5000	$C_{10}H_{20}O_2$	106-32-1	172.1460
	13.4838	Acetic acid	96.2310	$C_2H_4O_2$	64-19-7	60.0210
	15.1423	2,3-Butanediol, [R-(R*,R*)]-	92.6983	$C_4H_{10}O_2$	24347-58-8	90.0680
	15.6268	Bicyclo[5.2.0]nonane, 2-methylene-4,8,8-trimethyl-4-vinyl-	90.9491	$C_{15}H_{24}$	242794-76-9	204.1880
	15.8959	Decanoic acid, ethyl ester	96.3530	$C_{12}H_{24}O_2$	110-38-3	200.1780
	18.0750	Acetic acid, 2-phenylethyl ester	92.0253	$C_{10}H_{12}O_2$	103-45-7	164.0840
	18.2312	Dodecanoic acid, ethyl ester	95.4820	$C_{14}H_{28}O_2$	106-33-2	228.2090
	19.1152	Phenylethyl Alcohol	97.1904	$C_8H_{10}O$	60-12-8	122.0730
	19.3583	Hexanoic acid, 2-ethyl-	90.9118	$C_8H_{16}O_2$	149-57-5	144.1150
	20.3573	Tetradecanoic acid, ethyl ester	95.0375	$C_{16}H_{32}O_2$	124-06-1	256.2400
	22.3160	Hexadecanoic acid, ethyl ester	95.0560	$C_{18}H_{36}O_2$	628-97-7	284.2720
	24.8541	Ethyl Oleate	93.9783	$C_{20}H_{38}O_2$	111-62-6	310.2870
	25.5484	Linoleic acid ethyl ester	94.0721	$C_{20}H_{36}O_2$	544-35-4	308.2720
SWJD	4.5529	Ethyl Acetate	92.7731	$C_4H_8O_2$	141-78-6	88.0520
	7.0054	Butanoic acid, ethyl ester	92.5002	$C_6H_{12}O_2$	105-54-4	116.0840
	8.0083	1-Propanol, 2-methyl-	95.6498	$C_4H_{10}O$	78-83-1	74.0730
	8.5144	1-Butanol, 3-methyl-, acetate	93.5466	$C_7H_{14}O_2$	123-92-2	130.0990
	9.9575	Oxetane, 3-(1-methylethyl)-	90.7057	$C_6H_{12}O$	10317-17-6	100.0890
	10.3603	Hexanoic acid, ethyl ester	96.8467	$C_8H_{16}O_2$	123-66-0	144.1150
	13.3018	Octanoic acid, ethyl ester	94.5468	$C_{10}H_{20}O_2$	106-32-1	172.1460
	13.4887	Acetic acid	92.2736	$C_2H_4O_2$	64-19-7	60.0210
	15.8996	Decanoic acid, ethyl ester	95.2483	$C_{12}H_{24}O_2$	110-38-3	200.1780
	16.1487	Octanoic acid, 3-methylbutyl ester	92.6240	$C_{13}H_{26}O_2$	2035-99-6	214.1930
	16.3566	Butanedioic acid, diethyl ester	95.1168	$C_8H_{14}O_4$	123-25-1	174.0890
	16.5284	Ethyl 9-decenoate	91.0390	$C_{12}H_{22}O_2$	67233-91-4	198.1620
	18.0732	Acetic acid, 2-phenylethyl ester	91.0926	$C_{10}H_{12}O_2$	103-45-7	164.0840
	18.2312	Dodecanoic acid, ethyl ester	94.7376	$C_{14}H_{28}O_2$	106-33-2	228.2090
	18.4455	Pentadecanoic acid, 3-methylbutyl ester	90.1777	$C_{15}H_{30}O_2$	2306-91-4	242.2250
	19.1135	Phenylethyl Alcohol	96.9649	$C_8H_{10}O$	60-12-8	122.0730
	20.3566	Tetradecanoic acid, ethyl ester	96.3816	$C_{16}H_{32}O_2$	124-06-1	256.2400
	22.3192	Hexadecanoic acid, ethyl ester	94.2798	$C_{18}H_{36}O_2$	628-97-7	284.2720
	23.8453	Benzofuran, 2,3-dihydro-	90.7035	C_8H_8O	496-16-2	120.0580
	24.8542	(E)-9-Octadecenoic acid ethyl ester	93.8707	$C_{20}H_{38}O_2$	6114-18-7	310.2870
	25.5451	Linoleic acid ethyl ester	95.5014	$C_{20}H_{36}O_2$	544-35-4	308.2720

Table S13 Complete metabolite list

Metabolite	Metab ID	VIP_PLS-DA	P_value	FDR	SWJD_mean	SWJD_SD	SC_mean	SC_SD	GZ_LYM_mean	GZ_LYM_SD	JS_mean	JS_SD	HB_MPP_mean	HB_MPP_SD
Dioscin	metab_33907	2.915	1.325E-5	4.891E-5	0.2857	0.001578	0.2503	0.0005142	0.3217	0.003523	0.3555	0.002074	5.691	0.01265
Troleandomycin	metab_45619	2.759	3.028E-5	5.339E-5	3.889	0.1669	0.5954	0.0008756	2.4	1.322	3.776	0.3347	6.053	0.01069
Rphdhd	metab_16480	2.622	6.394E-5	8.64E-5	0.4567	0.3781	5.617	0.04449	0.244	0.001161	0.5588	0.7634	5.28	0.03074
Pa(Pgf2Alpha/22:1(13Z))	metab_42081	2.594	1.99E-5	4.891E-5	6.119	0.009246	0.4975	0.0008002	4.336	0.219	0.6518	0.002887	4.075	0.1554
Ala-Thr-Trp-Leu-Pro-Pro-Arg	metab_10859	2.539	0.001655	0.001722	0.6826	1.672	0.6973	1.087	1.036	1.362	0.0001698	2.56E-6	5.706	0.02935
Motexafin	metab_33775	2.461	1.9E-5	4.891E-5	5.271	0.01354	5.433	0.01062	5.572	0.02012	5.412	0.02307	1.618	0.7834
Acetyltributyl Citrate	metab_16515	2.436	1.616E-5	4.891E-5	4.161	0.1044	6.612	0.008527	4.65	0.05922	4.295	0.0545	1.781	1.903
Etamucine	metab_43867	2.412	3.38E-5	5.646E-5	2.98	1.281	4.036	0.08863	4.538	0.0329	4.509	0.0422	0.3983	0.006084
Tyr-Glu-Arg	metab_17135	2.363	0.0001388	0.0001638	1.696	0.8485	1.556	0.3076	1.413	0.002594	5.039	0.03387	4.601	0.04577
Hovenidulcioside B1	metab_19649	2.356	5.893E-5	8.092E-5	2.615	1.299	4.512	0.1179	5.626	0.01713	0.07758	0.001073	1.883	1.673
Actein	metab_47138	2.334	0.0001345	0.0001594	2.456	1.381	0.6422	0.0009059	2.248	1.162	1.232	0.5076	5.225	0.01397
5-Alpha-Dihydrotestosterone Glucuronide	metab_35390	2.301	0.0005327	0.0005737	2.922	0.1484	2.476	1.133	0.7014	0.5621	1.196	1.372	5.2	0.00734
(23S)-23,25-Dihdroxy-24-Oxovitamine D3 23-(Beta-Glucuronide)	metab_44094	2.257	1.9E-5	4.891E-5	1.249	0.5439	4.25	0.07128	1.791	0.8566	3.188	0.1273	5.243	0.01428
Pantetheine	metab_46832	2.236	1.325E-5	4.891E-5	6.164	0.00719	5.362	0.006278	0.5371	0.006847	5.614	0.008883	3.834	0.06182
Phe-Phe-Lys	metab_34656	2.226	1.616E-5	4.891E-5	4.758	0.01336	1.961	0.07762	2.394	0.4185	4.248	0.1537	5.057	0.01441
23-Trans-P-Coumaroyloxytormentic Acid	metab_51130	2.215	2.515E-5	4.891E-5	5.392	0.01259	3.25	0.2516	3.135	0.5226	4.849	0.04719	6.056	0.008633
Brevetoxin B2	metab_10466	2.192	2.298E-5	4.891E-5	2.755	0.001282	2.744	0.001856	2.956	0.3299	2.826	0.006595	5.97	0.02147
4'-O-Methyl-(-)-Epicatechin 3'-O-Glucuronide	metab_18637	2.176	3.603E-5	5.851E-5	1.843	0.4967	1.026	0.001684	5.42	0.01367	1.101	0.006077	2.626	1.188
(E)-N-(2-Amino-4-Fluorophenyl)-3-(1-Cinnamyl-1H-Pyrazol-4-Yl)Acrylamide	metab_39195	2.167	1.325E-5	4.891E-5	3.314	0.1882	4.754	0.01047	1.303	0.6926	5.1	0.01144	3.883	0.08323
Gibberellin A50	metab_17190	2.156	2.751E-5	5.075E-5	5.714	0.006497	0.9518	0.001652	3.363	0.3479	1.697	1.05	3.516	0.1266
Neolinustatin	metab_21852	2.134	1.325E-5	4.891E-5	6.029	0.01326	6.217	0.009138	5.807	0.01212	4.404	0.1351	3.287	0.5763
Pa(5-Iso Pgf2Vi/18:3(9Z,12Z,15Z))	metab_38641	2.111	0.0001388	0.0001638	1.746	1.035	4.772	0.02643	3.825	0.1333	1.501	0.7356	1.521	0.531
S-Glutaryldihydrolipoamide	metab_45837	2.093	2.038E-5	4.891E-5	5.028	0.02241	1.047	0.6144	3.774	0.09341	3.542	0.1106	0.8958	0.008889
Verbascoside	metab_37652	2.072	1.325E-5	4.891E-5	8.373	0.003317	4.215	0.04663	5.336	0.333	3.197	0.2077	3.84	0.192
3-Deoxy-D-Manno-Octulosonate	metab_49915	2.060	1.325E-5	4.891E-5	5.679	0.002847	4.949	0.01475	1.112	1.301	5.23	0.01652	4.307	0.09105
Fluocortolone Caproate	metab_48960	2.053	1.325E-5	4.891E-5	0.1496	0.0009537	0.1285	0.0003007	4.891	0.03107	1.552	1.5	4.02	0.0327
Cappariloside B	metab_8657	2.036	1.325E-5	4.891E-5	1.468	0.00124	1.457	0.001794	5.734	0.005652	1.582	0.08039	4.815	0.02795
Hesperidin Methylchalcone	metab_20258	2.032	0.0007421	0.0007889	6.28	0.01097	0.4835	1.145	1.123	1.712	0.2131	0.4749	0.5023	1.182
Spirolide B	metab_11532	2.018	1.518E-5	4.891E-5	2.079	0.001273	4.815	0.06449	5.431	0.03356	2.182	0.08077	2.782	0.5744
Gamma-Aminobutyric Acid Glutamate	metab_32782	2.008	2.075E-5	4.891E-5	0.9969	1.041	0.3949	0.1677	4.721	0.02695	5.199	0.02881	4.341	0.02159
Pyroglutamylproline	metab_29889	1.999	1.325E-5	4.891E-5	3.845	0.2062	0.6322	0.001426	4.907	0.01898	6.208	0.005351	1.551	1.257
5-(Furan-2-Carbonyl)-9-(Hydroxymethyl)-1,5-Diazacycloundecan-2-One	metab_19790	1.988	0.0002594	0.0002887	2.196	0.3396	1.064	1.447	0.08659	0.1186	5.361	0.00914	2.58	1.264
Cysteinylglycine	metab_1754	1.974	1.9E-5	4.891E-5	4.954	0.01215	3.96	0.0269	0.5035	1.185	4.013	0.1174	3.615	0.061

3-Hydroxy-Beta-Ionol 3-[Glucosyl-(1->6)-Glucoside]	metab_44510	1.972	1.9E-5	4.891E-5	2.312	0.0951	2.991	0.5483	5.003	0.00984	4.192	0.05366	1.694	0.1368
Gimatecan	metab_47901	1.971	1.325E-5	4.891E-5	4.815	0.03679	3.851	0.08814	0.1972	0.002453	4.362	0.03898	0.1854	0.003506
Purpurogallin	metab_44484	1.967	1.325E-5	4.891E-5	5.498	0.005101	5.466	0.004766	5.433	0.006838	5.41	0.004767	2.985	0.141
Kanokoside A	metab_43312	1.955	0.0005133	0.0005535	5.401	0.00856	0.01104	0.02118	0.02109	0.04854	0.00146	1.241E-5	0.001172	2.703E-5
Lucidenic Acid A	metab_43637	1.945	6.909E-5	9.185E-5	4.029	0.06208	1.265	1.497	0.5411	0.2488	5.171	0.00365	3.026	0.147
Scutellarioside Ii	metab_38123	1.943	0.0004279	0.0004648	5.623	0.007684	0.2063	0.283	0.7237	0.2425	0.8339	0.6547	0.2722	0.28
Ophiopogonin C	metab_37131	1.937	0.0001491	0.0001742	2.344	1.401	4.832	0.02437	1.445	1.314	0.6466	0.002877	5.11	0.02367
Phe-Asp-Gln	metab_47743	1.924	1.712E-5	4.891E-5	5.125	0.01578	0.9598	0.09936	3.626	0.05458	3.471	0.1188	2.356	0.5175
Cucumopine	metab_43438	1.920	1.325E-5	4.891E-5	5.294	0.0103	0.0717	0.0001787	0.09786	0.001354	0.1112	0.0008365	0.09137	0.001909
Lamiidoside	metab_12054	1.909	0.0001007	0.0001251	5.194	0.02434	0.8943	0.001622	2.182	1.338	0.9665	0.005886	0.9741	0.002914
Melatonin Glucuronide	metab_34717	1.909	9.361E-5	0.0001176	0.912	0.9217	1.212	1.269	5.385	0.01565	1.736	0.8834	4.596	0.02605
1-Ethyl 4-(2-Oxo-1,2-Diphenylethyl) Succinate	metab_20781	1.908	1.9E-5	4.891E-5	5.346	0.007745	0.01485	6.257E-5	0.01767	0.0001077	0.3707	0.481	0.01821	0.0001334
Spinacetin 3-[P-Coumaroyl-(->2)-Glucosyl-(1->6)-[Apiosyl-(1->2)]-Glucoside]	metab_38023	1.907	1.325E-5	4.891E-5	6.283	0.004914	2.426	0.001169	3.854	0.1579	2.636	0.003713	2.541	0.01018
5-(Delta-Carboxybutyl)Homocysteine	metab_35138	1.902	1.325E-5	4.891E-5	5.251	0.007843	0.4994	0.0008019	2.644	1.011	3.684	0.2216	0.5814	0.007496
Taurocholic Acid 3-Sulfate	metab_35979	1.896	1.518E-5	4.891E-5	3.533	0.217	4.958	0.02094	3.03	0.3168	4.623	0.04226	1.821	0.7743
N(6)-Hydroxymethyladenosine	metab_29356	1.895	4.118E-5	6.295E-5	1.313	0.2709	1.469	0.731	4.715	0.02818	2.188	1.041	5.189	0.01177
Pe(6 Keto-Pgf1Alpha/18:4(6Z,9Z,12Z,15Z))	metab_38069	1.892	1.874E-5	4.891E-5	6.005	0.006964	5.992	0.006868	5.976	0.008976	5.801	0.009631	3.723	0.3474
Styraxlignolide F	metab_17941	1.891	2.818E-5	5.132E-5	5.948	0.007835	0.3244	0.0009789	1.031	0.7402	2.417	0.29	0.3743	0.001881
Trans-Zeatin	metab_48333	1.881	2.49E-5	4.891E-5	3.589	0.09046	1.819	0.7835	5.177	0.02041	5.031	0.01123	2.053	0.7445
Kaempferol 3-O-Feruloyl-Sophoroside 7-O-Glucoside	metab_38352	1.877	1.325E-5	4.891E-5	5.741	0.01047	0.7231	0.0009514	0.8472	0.005799	0.9013	0.003252	0.8189	0.008635
Dide-O-Methyl-4-O-Alpha-D-Glucopyranosylsimmondsin	metab_18068	1.864	8.997E-5	0.0001139	5.521	0.01024	0.3921	0.001106	0.8656	1.043	0.9148	1.062	0.448	0.002096
Trp-Tyr-His	metab_46462	1.862	3.353E-5	5.632E-5	2.339	0.6488	1.381	0.07358	1.822	0.3664	5.242	0.01311	3.571	0.08311
Mg(15:0)	metab_16936	1.860	0.0001306	0.0001556	2.911	0.5474	5.123	0.05581	3.106	0.4932	4.882	0.05165	2.492	0.003251
Pa(Pgj2/I-12:0)	metab_37692	1.855	4.159E-5	6.337E-5	2.744	1.422	3.941	0.05158	2.489	1.152	5.624	0.01269	4.75	0.02928
Gly Gly Phe	metab_25236	1.851	1.325E-5	4.891E-5	2.16	1.172	1.393	0.001784	5.11	0.02436	5.727	0.01556	4.777	0.04363
Fumagillin	metab_36022	1.849	1.325E-5	4.891E-5	3.928	0.04991	3.217	0.266	3.804	0.02471	5.023	0.01851	5.525	0.007223
Asp-Ile-His	metab_11395	1.847	2.49E-5	4.891E-5	2.956	0.6531	4.526	0.04471	2.75	0.5757	5.179	0.02518	4.696	0.04125
Ac-Pro-Gly-Pro-Oh	metab_44386	1.846	2.006E-5	4.891E-5	4.971	0.0184	4.535	0.06226	4.909	0.03643	4.858	0.0191	2.558	0.177
Skimmin	metab_10827	1.845	0.0001253	0.0001501	6.93	0.002271	2.264	0.001849	2.75	0.6364	2.42	0.1825	2.354	0.003247
Quercetin 7-Glucoside	metab_21943	1.844	2.49E-5	4.891E-5	5.335	0.02018	5.173	0.0338	5.046	0.01295	5.162	0.01853	2.83	0.5717
2-Hydroxy-Desipramine Glucuronide	metab_49141	1.838	2.53E-5	4.891E-5	2.854	0.2586	1.947	0.01812	5.385	0.04383	4.147	0.3217	2.791	0.4039
Swertiajaponin	metab_43911	1.831	2.359E-5	4.891E-5	7.036	0.003924	2.433	0.001169	2.927	0.5523	2.643	0.003713	2.548	0.01018
Valganciclovir, (S)-	metab_18138	1.830	1.9E-5	4.891E-5	6.632	0.007728	1.312	0.001769	2.667	0.9237	4.889	0.01503	1.398	0.003131
Rosamicin	metab_43819	1.826	6.206E-5	8.437E-5	2.452	1.117	3.954	0.03094	1.909	1.248	4.727	0.02055	0.7888	0.912
Spb(20:0_2O)	metab_16735	1.825	2.298E-5	4.891E-5	5.411	0.119	6.426	0.01576	3.754	0.2126	5.739	0.04254	5.783	0.07855

Trp-Tyr	metab_45709	1.824	1.325E-5	4.891E-5	4.063	0.05215	3.761	0.0792	0.9446	0.8754	4.996	0.01561	3.008	0.2087
Histidyltyrosine	metab_37111	1.816	0.0006215	0.0006652	4.928	0.01416	1.077	1.144	2.57	1.133	0.4492	0.4161	1.997	1.36
10-Acetoxyligustroside	metab_39080	1.814	1.9E-5	4.891E-5	6.262	0.008622	1.8	0.4587	3.261	0.1392	3.847	0.125	3.402	0.08355
Euglobal Ia1	metab_44194	1.810	1.807E-5	4.891E-5	1.615	0.7774	1.293	0.2719	4.941	0.03214	5.898	0.01229	4.345	0.06737
Ligustroside	metab_39200	1.808	3.114E-5	5.392E-5	7.192	0.004083	2.859	1.223	4.272	0.2822	3.975	0.07036	3.167	0.1752
Andromedoside	metab_39004	1.805	1.325E-5	4.891E-5	5.119	0.0244	0.4822	0.0007869	0.5872	0.005004	0.6344	0.002853	0.5628	0.007378
Desacetylvinblastine Amide	metab_45273	1.799	0.0004458	0.0004835	3.123	0.5542	3.459	0.1413	2.783	1.071	2.332	1.111	5.65	0.01319
Methylprednisolone Acetate	metab_17466	1.793	1.421E-5	4.891E-5	4.711	0.07042	3.931	0.2432	4.552	0.02374	5.566	0.02376	1.829	0.8898
Dextran-70	metab_46758	1.792	6.64E-5	8.907E-5	4.863	0.01278	0.3505	0.00065	1.523	0.5737	1.418	0.839	0.993	0.2859
Prunin 6''-O-Gallate	metab_9762	1.780	9.232E-5	0.0001162	4.999	0.1898	4.937	0.1451	3.959	0.2873	3.083	0.912	2.343	0.05307
Pi(6 Keto-Pgf1Alpha/16:2(9Z,12Z))	metab_44638	1.776	6.992E-5	9.261E-5	2.748	0.3127	3.793	0.192	3.241	0.3378	2.649	0.003713	5.713	0.009234
Nystatin	metab_38441	1.776	0.0006012	0.0006443	2.526	0.6581	3.103	0.3515	2.239	0.6891	1.853	0.003669	4.925	0.033
Granotapide	metab_37632	1.773	0.001078	0.001133	5.192	0.05432	0.716	0.4173	0.9096	0.3538	0.8289	0.1378	0.8661	0.3672
Ganoderiol I	metab_40623	1.770	0.0003018	0.0003331	6.367	0.01761	1.662	0.31	1.89	0.326	2.293	0.503	2.017	0.4142
4-Hydroxycoumarin	metab_20276	1.768	0.0001007	0.0001251	6.594	0.002228	2.453	0.001853	3.028	0.5602	2.535	0.006585	2.543	0.003252
Lpa(3:0)	metab_47359	1.768	2.751E-5	5.075E-5	2.867	0.2844	4.909	0.008469	2.494	0.006749	3.227	0.3425	4.554	0.01468
Mimic A	metab_45506	1.755	1.518E-5	4.891E-5	4.891	0.01391	1.72	0.4514	3.991	0.04227	3.538	0.0559	2.796	0.3047
Ethyl 4-Hydroxy-3-Methylisoxazolo[5,4-B]Pyridine-5-Carboxylate	metab_32860	1.752	5.066E-5	7.205E-5	0.02299	0.0001688	1.195	1.297	4.375	0.04055	4.146	0.09738	4.156	0.0267
4-O-P-Coumaroylquinic Acid	metab_38191	1.748	0.0001141	0.0001388	5.433	0.01408	1.082	0.3133	1.088	0.006213	1.146	0.003454	1.058	0.009299
Capryloylglycine	metab_39710	1.747	2.835E-5	5.147E-5	4.797	0.02449	0.4714	0.0007772	0.7384	0.4008	3.361	0.2181	2.066	1.189
4'-O-Beta-D-Glucosyl-5-O-Methylvisamminol	metab_12194	1.742	2.53E-5	4.891E-5	5.447	0.01072	1.27	0.001759	1.343	0.002576	3.336	0.2522	2.783	1.11
Isowertin 2''-Rhamnoside	metab_37231	1.740	0.001171	0.001228	5.284	0.01101	0.911	1.083	0.2794	0.003193	0.3895	0.195	0.5568	0.7182
P-Hpea-Eda	metab_39564	1.739	2.411E-5	4.891E-5	5.069	0.01912	2.158	0.1852	3.782	0.04084	2.297	0.2972	3.358	0.068
4-{Methyl[3-Phenyl-3-(Pyridin-2-Yl)Propyl]Amino}-4-Oxobutanoic Acid	metab_18952	1.739	2.411E-5	4.891E-5	3.053	0.2229	3.999	0.0876	2.929	0.178	4.217	0.02506	4.828	0.01627
N-Formyl-Met-Leu-Phe-Lys	metab_45612	1.736	9.087E-5	0.0001148	2.872	0.1544	3.884	0.09232	3.869	0.1296	3.972	0.1103	5.811	0.008835
Licoagrodin	metab_45220	1.735	2.23E-5	4.891E-5	2.068	0.3155	5.495	0.009262	5.169	0.01741	3.543	0.2763	3.729	0.1003
Metronidazole Phosphate	metab_36507	1.735	1.325E-5	4.891E-5	5.638	0.0231	1.341	0.00112	1.484	0.006547	1.544	0.003614	1.452	0.00984
Osmanthuside A	metab_38977	1.732	2.359E-5	4.891E-5	5.904	0.003785	2.178	1.03	3.66	0.1507	4.161	0.05091	4.121	0.05483
(4R)-4-Hydroxy-L-Glutamic Acid	metab_2044	1.728	1.325E-5	4.891E-5	5.186	0.006652	4.863	0.01084	2.58	0.05143	5.301	0.008455	4.662	0.01201
Serylvalylglycylglutamic Acid	metab_31873	1.724	5.251E-5	7.419E-5	2.547	0.3282	3.709	0.8115	3.212	0.5383	5.256	0.03491	4.951	0.07209
Butirosina	metab_44176	1.723	1.807E-5	4.891E-5	2.696	1.04	1.574	1.038	4.958	0.0844	5.452	0.05006	4.571	0.03235
Plantamajoside	metab_36754	1.720	7.436E-5	9.684E-5	6.382	0.002159	1.669	0.8995	0.7466	0.005546	3.74	0.2002	0.7195	0.008232
Gmp-N-Epsilon-(N-Alpha-Acetyl Lysine Methyl Ester) 5'-Phosphoramidate	metab_13584	1.713	8.98E-5	0.0001137	6.885	0.006993	2.885	0.001857	3.289	0.5056	2.966	0.006598	2.975	0.003258
Amarogentin	metab_37674	1.708	2.075E-5	4.891E-5	7.208	0.004389	3.84	0.07287	4.762	0.08895	4.363	0.03265	3.524	0.3092
Asp-Tyr-Gln	metab_34596	1.703	1.325E-5	4.891E-5	5.011	0.01981	4.716	0.02593	5.199	0.01299	4.849	0.01415	3.216	0.2728

Methionine Sulfoxide	metab_29314	1.703	1.325E-5	4.891E-5	5.375	0.003228	5.561	0.008442	2.92	0.2883	5.32	0.01863	4.801	0.009598
Penitrem B	metab_35835	1.702	1.421E-5	4.891E-5	3.851	0.1375	5.216	0.0169	5.073	0.02683	4.449	0.09282	3.441	0.213
3,5-Dimethyl-3'-Isopropyl-L-Thyronine	metab_21875	1.701	1.325E-5	4.891E-5	5.512	0.01388	4.122	0.2071	4.655	0.05871	4.862	0.08011	2.498	0.5113
Astragaloside Iii	metab_23754	1.700	4.085E-5	6.262E-5	2.368	0.0344	2.615	0.6069	5.899	0.01141	3.568	0.1928	5.168	0.07319
Ps(5-Iso Pgf2Vi/14:0)	metab_38570	1.696	2.156E-5	4.891E-5	1.85	0.003229	3.503	0.06502	2.897	0.2509	2.48	0.4346	5.08	0.01153
Lupinic Acid	metab_42466	1.693	2.455E-5	4.891E-5	0.8957	0.287	1.055	0.3165	4.576	0.01789	3.791	0.03205	3.553	0.07462
Thesinine 4'-O-Glucoside	metab_36085	1.687	3.36E-5	5.633E-5	2.326	1.056	0.7856	0.712	4.526	0.01539	5.205	0.01299	2.941	0.5328
2'-(E)-Feruloyl-3-(Arabinosylxylose)	metab_12502	1.687	1.325E-5	4.891E-5	2.789	0.001282	2.778	0.001856	5.492	0.01119	2.859	0.006596	3.717	0.2228
Vinorelbine Base	metab_3165	1.685	2.455E-5	4.891E-5	4.921	0.07418	1.776	0.3242	1.598	0.3633	5.875	0.1329	2.906	0.2579
Cucurbitacin A	metab_36614	1.684	2.52E-5	4.891E-5	2.964	0.1327	3.705	0.2104	3.472	0.3231	4.288	0.05013	5.227	0.03432
Cer(8:0_2O/14:0)	metab_16824	1.683	7.784E-5	0.0001007	3.515	0.2708	5.274	0.0262	3.389	0.04959	3.67	0.2925	5.545	0.01582
Tricin	metab_13873	1.683	1.325E-5	4.891E-5	4.86	0.02227	4.967	0.02765	4.423	0.02106	4.568	0.03128	2.857	0.105
Alpha-Hydroxy-N-Desmethyltamoxifen	metab_39385	1.681	1.616E-5	4.891E-5	3.449	0.0691	4.356	0.06092	3.253	0.1649	4.518	0.03273	5.005	0.01282
2-Glucosyloxy-4-Methoxycinnamic Acid	metab_36806	1.676	0.0002594	0.0002887	5.922	0.009751	3.554	0.9689	3.111	0.5375	2.63	0.9548	1.714	0.01001
Aucubin	metab_23295	1.675	2.515E-5	4.891E-5	5.243	0.0109	4.808	0.03141	5.048	0.03237	5.036	0.01649	3.13	0.1287
Z-Asp-Glu-Val-Asp-Fluoromethylketone	metab_43889	1.674	2.576E-5	4.929E-5	5.489	0.009186	1.335	0.5506	2.252	0.2721	3.277	0.1115	2.576	0.2057
Benzoylmesaconine	metab_38184	1.674	1.421E-5	4.891E-5	5.009	0.01297	4.255	0.07098	2.017	0.7069	5.408	0.01397	3.954	0.1205
Citbismine A	metab_45565	1.670	1.325E-5	4.891E-5	5.363	0.01482	1.381	0.001124	1.524	0.006567	1.585	0.003624	1.492	0.009872
Cinnzeylanol	metab_33126	1.668	2.038E-5	4.891E-5	3.109	0.1629	1.962	0.00116	2.338	0.553	4.764	0.3855	3.816	0.1183
Gly-Leu-His	metab_1398	1.668	1.9E-5	4.891E-5	3.509	0.1654	1.777	0.6752	4.163	0.1242	4.792	0.03925	1.589	0.003177
Blennin D	metab_35892	1.666	1.325E-5	4.891E-5	2.536	0.1461	2.096	0.001164	4.716	0.01773	2.306	0.003703	3.206	0.1721
Ergocornine	metab_42878	1.665	0.00108	0.001135	5.061	0.2542	4.323	2.081	2.377	1.582	4.187	0.5786	0.9333	0.9765
Licoricesaponin F3	metab_47293	1.664	1.325E-5	4.891E-5	1.252	0.003092	5.725	0.01758	5.691	0.007733	1.5	0.2272	5.799	0.012
Thiomorpholine 3-Carboxylate	metab_29732	1.663	1.325E-5	4.891E-5	5.182	0.009077	5.311	0.0114	2.777	0.2831	5.131	0.02092	4.57	0.02596
Toxin T2 Tetrol	metab_47079	1.660	1.325E-5	4.891E-5	2.423	0.003263	3.877	0.05564	3.335	0.08008	4.736	0.02722	4.839	0.006833
Desethylenenorfloxacin	metab_38637	1.657	1.9E-5	4.891E-5	4.834	0.01263	3.046	0.9141	3.897	0.07268	4.457	0.02542	1.596	0.5755
Dihydroergocornine	metab_37902	1.657	1.712E-5	4.891E-5	4.625	0.04132	5.27	0.009264	3.85	0.1227	4.695	0.0491	5.814	0.008218
Boldenone	metab_41949	1.655	0.0001141	0.0001388	5.881	0.01466	1.97	0.09492	2.078	0.006714	2.14	0.003694	2.045	0.01011
Neoacrimarine H	metab_38120	1.651	4.716E-5	6.838E-5	5.196	0.01519	2.615	0.1966	4.223	0.07105	3.077	0.3454	3.262	0.3686
2,3-Dinor-Txb2	metab_49131	1.648	2.515E-5	4.891E-5	3.107	0.196	2.418	0.02046	5.577	0.04305	4.573	0.07714	4.584	0.06402
N-(3,4-Difluorophenyl)-2-Methoxy-4-Methylpyrimidine-5-Carboxamide	metab_2341	1.647	1.9E-5	4.891E-5	4.936	0.02487	5.211	0.01804	3.084	0.3569	4.779	0.1148	5.03	0.06694
Caldiamide	metab_13750	1.647	2.071E-5	4.891E-5	4.042	0.09111	4.814	0.0265	3.671	0.1551	4.864	0.03153	5.385	0.01892
Pentanoic Acid, 5-(Dipentylamino)-5-Oxo-4-((3-Quinolinylcarbonyl)Amino)-, (R)-	metab_46748	1.641	1.325E-5	4.891E-5	2.796	0.1353	4.873	0.01756	3.619	0.2099	4.641	0.02986	5.063	0.0115
1-(2-Hydroxy-4,6-Dimethoxyphenyl)-3-(3,4,5-Trimethoxyphenyl)Propan-1-One	metab_44938	1.640	0.0006036	0.0006468	5.495	0.003233	2.139	0.2999	2.724	0.4345	2.096	0.2431	2.033	0.3217
Umbelliferone 7-O-Rutinoside	metab_20265	1.634	2.402E-5	4.891E-5	6.497	0.008942	2.707	0.001856	2.784	0.002694	2.788	0.006594	2.797	0.003256

Hydroxyclomipramine	metab_37101	1.634	1.325E-5	4.891E-5	2.365	0.7542	6.266	0.003854	6.033	0.0131	4.279	0.1045	5.045	0.01778
Phe-Gly-Lys	metab_45833	1.632	3.476E-5	5.731E-5	5.513	0.01456	1.498	0.5316	2.892	0.7772	3.661	0.1802	1.902	0.7527
Olitorin	metab_46214	1.631	7.692E-5	9.973E-5	3.28	0.2351	3.604	0.1529	3.921	0.1188	3.534	0.1877	5.609	0.007987
3-[(2-Methyl-3-Furanyl)Thio]-4-Heptanone	metab_49345	1.631	1.325E-5	4.891E-5	4.367	0.01807	4.653	0.008555	2.605	0.5284	5.216	0.04104	4.227	0.02344
Gly-Val-Tyr	metab_44457	1.630	1.99E-5	4.891E-5	3.612	0.1596	4.237	0.04141	3.437	0.1066	4.92	0.01505	5.028	0.007616
Physcion 8-Gentiobioside	metab_21984	1.630	0.0003687	0.0004032	5.774	0.01357	1.934	0.4555	1.879	0.1347	1.828	0.006506	2.131	0.722
Thr Pro Leu	metab_25201	1.629	0.0001655	0.0001904	3.136	0.8284	2.856	1.453	5.172	0.04963	5.667	0.007793	1.987	1.949
Hydrodolasetron	metab_36930	1.624	2.774E-5	5.099E-5	4.835	0.008819	1.017	0.00106	1.85	0.4662	2.179	0.3781	1.209	0.2026
Coniferin	metab_46120	1.623	0.0001563	0.0001813	5.839	0.004118	1.689	0.1298	1.661	0.1328	2.701	0.06723	1.761	0.18
Ribalinium	metab_46413	1.623	1.807E-5	4.891E-5	3.476	0.1049	3.192	0.2453	4.117	0.08973	3.846	0.09061	5.365	0.01311
3'-O-Methyl-(-)-Epicatechin-5-O-Sulphate	metab_38409	1.621	2.411E-5	4.891E-5	5.804	0.006621	2.507	0.00117	3.976	0.06699	3.945	0.04841	3.492	0.06033
Met-Thr-Phe	metab_42760	1.620	3.293E-5	5.561E-5	2.245	0.08673	2.134	0.001165	4.712	0.03515	2.344	0.003704	3.093	0.4128
Lys-Thr-Asn	metab_12751	1.618	1.325E-5	4.891E-5	2.902	0.2209	5.101	0.02537	4.479	0.0433	5.412	0.01062	5.796	0.008015
2''-Methoxy-(S)-Oleuropein	metab_43135	1.616	3.848E-5	6.072E-5	6.053	0.003358	2.043	0.001163	2.428	0.3977	3.049	0.1199	2.394	0.2994
Docosahexaenoic Acid	metab_41814	1.615	0.0005748	0.0006171	4.635	0.4279	3.822	1.202	1.046	0.7109	1.875	1.666	0.8036	0.4195
4-Nitrophenyl 4-(3-Phenoxybenzyl)Piperazine-1-Carboxylate	metab_38202	1.612	1.325E-5	4.891E-5	5.683	0.004847	1.312	0.001116	1.662	0.5067	3.655	0.04066	1.423	0.009815
Taraxacoside	metab_43747	1.612	2.515E-5	4.891E-5	2.636	0.1851	2.81	0.3322	5.832	0.009991	3.638	0.07498	4.665	0.02323
Ganosporeric Acid A	metab_44112	1.611	1.9E-5	4.891E-5	1.795	0.5478	4.401	0.01719	3.923	0.1204	2.472	0.2013	5.813	0.007323
[(2E,6E)-1-Oxo-8-Hydroxy-2,6-Dimethylocta-2,6-Dien-1-Yl]Beta-D-Glucopyranoside	metab_10574	1.607	3.274E-5	5.547E-5	5.246	0.01402	1.406	0.001786	1.481	0.00261	1.749	0.6487	1.493	0.003156
Indole-3-Acetylglutamic Acid	metab_44132	1.603	4.55E-5	6.677E-5	6.427	0.006445	2.524	0.1116	1.801	0.1929	3.028	0.174	1.879	0.4854
25-O-Desacetyl Rifabutin	metab_44738	1.602	2.535E-5	4.891E-5	5.115	0.03125	4.953	0.02981	4.956	0.01624	4.354	0.2072	3.426	0.2086
Imidazoleacetic Acid Riboside	metab_28260	1.602	1.325E-5	4.891E-5	0.7054	0.001031	0.6966	0.001486	0.7592	0.002229	5.205	0.341	0.787	0.04189
Leonurine	metab_2139	1.602	1.99E-5	4.891E-5	4.53	0.08851	0.4334	0.001174	1.522	0.4148	5.096	0.04662	1.027	0.4759
Caffeine	metab_23740	1.601	0.0005224	0.000563	5.939	0.2869	4.759	1.024	4.49	0.6136	4.127	0.8269	2.771	0.003256
Pip(5-Iso Pgf2Vi/16:2(9Z,12Z))	metab_37791	1.600	6.992E-5	9.261E-5	4.097	0.1119	3.969	0.1122	3.636	0.2089	3.846	0.1311	5.391	0.007275
1-(3,5-Dimethylbenzoyl)-N-(3-Methylsulfanylphenyl)Piperidine-3-Carboxamide	metab_21564	1.598	4.604E-5	6.719E-5	3.582	0.2444	3.666	0.1753	1.387	0.8193	5.224	0.02471	1.728	1.1
Glycocholic Acid 3-Sulfate	metab_44878	1.598	2.23E-5	4.891E-5	2.523	0.4005	2.859	0.381	4.326	0.03151	3.747	0.1264	5.319	0.00843
Echinacoside	metab_36558	1.595	0.001774	0.001843	6.145	0.003738	2.931	0.7307	1.863	0.6517	1.659	0.003639	1.995	0.7313
Medicocarpin	metab_17314	1.590	4.286E-5	6.445E-5	5.791	0.008445	2.301	0.00185	2.673	0.2246	2.469	0.1393	2.835	0.1686
Silodosin	metab_46196	1.589	1.325E-5	4.891E-5	5.433	0.006197	5.052	0.0103	5.534	0.009285	6.082	0.006031	3.521	0.134
Castanospermine	metab_48929	1.588	1.616E-5	4.891E-5	3.141	0.3397	2.633	0.223	5.735	0.01271	5.085	0.0506	4.673	0.02796
Pro Asp Ala Lys Ser	metab_12353	1.583	1.325E-5	4.891E-5	5.197	0.02045	4.882	0.03214	4.611	0.007774	3.998	0.09112	3.22	0.1325
N-Acetyl-9-O-Lactoylneuraminic Acid	metab_49012	1.578	1.616E-5	4.891E-5	1.804	0.003224	3.055	0.2692	5.543	0.02879	2.124	0.4567	4.147	0.1234
Acipimox	metab_28775	1.577	2.23E-5	4.891E-5	4.733	0.02971	4.889	0.01774	2.821	0.2456	4.756	0.03756	4.502	0.02574
Homovanillic Acid	metab_12531	1.577	2.345E-5	4.891E-5	5.077	0.009738	2.118	0.001845	3.02	0.2329	2.615	0.4016	3.375	0.1053

Glucose 1-Phosphate	metab_31811	1.577	2.359E-5	4.891E-5	2.512	1.74	5.604	0.01674	5.83	0.03796	0.9783	0.5664	5.099	0.07176
Spb(20:0_3O)	metab_16770	1.576	1.616E-5	4.891E-5	5.122	0.05715	5.778	0.01302	3.92	0.2962	5.746	0.01732	5.383	0.02736
3-(1,1-Dimethylallyl)Scopoletin 7-Glucoside	metab_44937	1.576	1.9E-5	4.891E-5	5.728	0.00796	1.649	0.001147	2.2	0.4485	3.579	0.1447	1.929	0.401
Sesaminol Glucosyl-(1->2)-Glucoside	metab_32068	1.576	1.9E-5	4.891E-5	3.579	0.003275	6.241	0.01579	4.179	0.3006	3.713	0.00372	5.763	0.02974
25-Acetyl-6,7-Didehydrofevicordin F 3-Glucoside	metab_38687	1.575	2.66E-5	5.007E-5	4.113	0.07363	4.309	0.08716	4.039	0.09614	5.034	0.01723	2.267	0.8309
9-Hexadecenoic Acid	metab_14316	1.575	2.156E-5	4.891E-5	4.341	0.09869	5.302	0.01893	3.468	0.281	5.192	0.01309	3.218	0.2383
Trp-Gly-Thr	metab_5815	1.571	1.807E-5	4.891E-5	3.387	0.3493	4.838	0.08513	4.523	0.008708	5.789	0.01746	5.816	0.01528
(1S,2R,4R,8S)-P-Menthane-2,8,9-Triol 2-Glucoside	metab_42802	1.570	2.515E-5	4.891E-5	5.605	0.01207	2.478	0.4214	3.792	0.04075	4.068	0.02416	2.552	0.4055
(+/-)-Win 55,212	metab_18618	1.568	2.063E-5	4.891E-5	2.487	0.768	1.704	0.7233	4.956	0.01801	5.45	0.01986	3.634	0.2592
2'-Deoxymugineic Acid	metab_673	1.567	1.325E-5	4.891E-5	5.78	0.01023	5.329	0.04148	5.522	0.02262	5.416	0.02155	3.859	0.2991
Digitoxigenin 3-[Glucosyl-(1->6)-Glucosyl-(1->4)-2,6-Dideoxyribohexoside]	metab_37320	1.566	1.325E-5	4.891E-5	1.178	1.655	5.201	0.0125	5.684	0.009521	4.126	0.08236	5.973	0.009138
(R)-1-O-[B-D-Apiofuranosyl-(1->2)-B-D-Glucopyranoside]-1,3-Octanediol	metab_33856	1.563	1.874E-5	4.891E-5	0.5197	0.4605	2.374	0.9897	4.558	0.04831	5.508	0.0148	3.384	0.2344
Calystegin A3	metab_33114	1.562	2.298E-5	4.891E-5	2.95	0.36	2.707	0.2451	5.699	0.05535	5.046	0.03638	4.817	0.05939
Gomisin D	metab_47420	1.562	1.421E-5	4.891E-5	3.052	0.1621	5.117	0.01474	4.238	0.08729	5.183	0.01383	3.516	0.1965
Ibuprofen Metabolite A	metab_39647	1.562	1.325E-5	4.891E-5	5.741	0.003051	2.253	0.001167	2.401	0.006744	2.463	0.003708	2.368	0.01016
Alpha-Tetrasaccharide	metab_44252	1.561	1.99E-5	4.891E-5	3.241	0.1171	2.697	0.5743	5.533	0.009012	4.248	0.05413	4.615	0.05837
Gln-Phe-Ser	metab_34418	1.559	2.075E-5	4.891E-5	3.988	0.04927	3.309	0.1454	4.031	0.03433	5.077	0.00621	1.843	0.5909
Belotecan	metab_33384	1.558	0.000141	0.0001661	2.697	0.2232	2.739	0.3211	5.383	0.04835	2.92	0.4227	4.639	0.05722
Mabioside C	metab_36989	1.558	1.99E-5	4.891E-5	4.846	0.03975	3.381	0.5257	5.082	0.03425	2.903	0.2393	4.527	0.06555
Phenobarbital N-Glucoside	metab_46783	1.557	1.807E-5	4.891E-5	3.763	0.09594	5.204	0.01533	3.898	0.09948	5.777	0.004221	4.908	0.01517
3Z-Dodecenedioic Acid	metab_42598	1.556	0.0003849	0.0004201	6.06	0.006306	2.976	0.3107	3.369	0.2177	2.856	0.3314	2.85	0.2234
20-Hydroxy-6Z,15Z-Eicosadienoic Acid	metab_16860	1.555	1.807E-5	4.891E-5	5.224	0.01594	5.184	0.02846	3.3	0.2817	4.061	0.1462	5.406	0.01132
Hispiduloside	metab_44426	1.552	1.518E-5	4.891E-5	6.126	0.005064	2.492	0.001169	2.659	0.05064	3.11	0.3511	2.607	0.01018
N-Phenylanthranilic Acid	metab_9276	1.552	1.325E-5	4.891E-5	1.613	1.453	4.394	0.0176	4.056	0.0789	4.857	0.0133	5.056	0.008891
Tyr Phe Glu Lys	metab_19338	1.551	1.851E-5	4.891E-5	4.403	0.09461	4.142	0.1366	4.91	0.03563	0.6886	0.9411	4.843	0.03188
Tyr Gln Asn Glu	metab_27257	1.550	2.156E-5	4.891E-5	5.35	0.01726	5.368	0.02118	4.682	0.1156	6.116	0.02457	3.085	1.179
(1Xi,2Xi)-1-(4-Hydroxyphenyl)-1,2,3-Propanetriol 2-O-Beta-D-Glucopyranoside	metab_35006	1.550	2.927E-5	5.235E-5	6.891	0.002121	2.947	0.1594	3.491	0.4951	5.516	0.01328	2.78	0.01019
Bedoradrine	metab_133	1.550	4.118E-5	6.295E-5	4.303	0.04802	4.167	0.1656	5.822	0.01485	3.174	0.1544	4.423	0.1058
Pressinoic Acid	metab_50084	1.549	1.518E-5	4.891E-5	2.038	0.2823	5.823	0.02355	4.424	0.1463	2.968	0.8007	5.208	0.04475
Glu-Phe-Tyr	metab_17100	1.549	0.000376	0.0004108	2.415	0.1531	2.994	0.7245	5.372	0.02233	2.775	0.5511	3.535	0.5695
Acetophenone	metab_17590	1.549	0.0001788	0.0002038	5.485	0.007039	2.087	0.1059	2.509	0.2805	2.497	0.2109	2.251	0.1851
3-Trans-P-Coumaroylrotundic Acid	metab_42182	1.547	0.0002135	0.0002402	5.493	0.004905	2.114	0.2771	1.933	0.1988	2.082	0.4395	2.648	0.2561
Pc(20:5/0:0)	metab_6630	1.546	0.003703	0.003794	1.751	0.9579	2.35	1.307	1.58	0.8631	5.915	0.01262	1.622	0.9334
3-[(3-(2-Carboxyethyl)-4-Methylpyrrol-2-Yl)Methylene]-2-Indolinone	metab_38338	1.545	3.518E-5	5.759E-5	4.905	0.02181	1.738	0.6901	3.24	0.09073	3.31	0.101	2.473	0.5308
Antibiotic Ll-Ab 664	metab_44975	1.543	1.9E-5	4.891E-5	3.645	0.03951	2.752	0.04716	5.322	0.007312	4.268	0.06893	4.335	0.03134

Strictosidine	metab_2764	1.542	4.577E-5	6.7E-5	3.957	0.1875	1.972	0.1746	0.6203	0.9477	5.401	0.6188	0.8215	0.6183
Schinalactone C	metab_40178	1.541	3.902E-5	6.072E-5	5.266	0.01031	1.85	0.001157	1.997	0.006702	2.059	0.003688	2.072	0.256
6-Methylcoumarin	metab_47376	1.540	1.325E-5	4.891E-5	4.141	0.03153	5.343	0.006663	3.531	0.04974	4.345	0.01636	5.045	0.01528
Dactolisib	metab_42719	1.539	1.325E-5	4.891E-5	3.249	0.003274	3.172	0.001172	5.763	0.01303	3.686	0.1441	4.61	0.01239
Forsythoside B	metab_45098	1.539	2.535E-5	4.891E-5	5.903	0.009255	2.717	0.3022	3.274	0.07688	3.282	0.1958	3.666	0.05027
Gly Cys Val	metab_28807	1.539	8.427E-5	0.0001077	2.616	1.056	0.8236	0.5205	3.686	0.429	5.809	0.01491	2.658	1.06
Heliotron	metab_22903	1.539	1.325E-5	4.891E-5	2.2	0.001276	5.47	0.009582	3.863	0.06963	2.494	0.316	5.221	0.02063
Glycyrrhizin	metab_6919	1.537	4.577E-5	6.7E-5	1.33	0.001224	3.67	1.171	6.038	0.01231	3.254	1.149	5.615	0.02994
Poncirin	metab_37504	1.535	0.0001141	0.0001388	5.467	0.01211	2.096	0.2042	2.16	0.006724	2.222	0.003699	2.127	0.01013
Dg(Pgd2/A-17:0/0:0)	metab_12138	1.535	0.001421	0.001483	5.232	0.02315	2.305	1.261	1.841	1.344	0.9802	0.005908	3.156	0.4948
Forsythoside H	metab_19572	1.534	0.0004467	0.0004844	6.549	0.006244	3.333	0.3613	3.021	0.4497	2.814	0.1877	3.644	0.2161
Asn Tyr Phe Glu	metab_13833	1.534	3.742E-5	5.985E-5	3.3	0.136	3.2	0.001858	5.154	0.01679	3.282	0.006601	3.553	0.2231
Biopterin	metab_44069	1.530	1.325E-5	4.891E-5	4.478	0.0204	4.524	0.008027	2.345	0.02523	3.31	0.1316	4.304	0.02399
Fagomine	metab_10018	1.530	0.001162	0.001219	0.7241	0.8756	1.035	1.046	0.4055	0.001639	4.609	0.01266	0.4136	0.002
16-(1-Carboxyethoxy)-16-Oxohexadecanoic Acid	metab_40025	1.529	2.591E-5	4.929E-5	6.157	0.008854	2.766	0.1056	3.313	0.2592	3.611	0.1781	2.838	0.01019
(+)-8-Acetoxycarvone	metab_36041	1.528	2.359E-5	4.891E-5	4.342	0.01103	4.157	0.01326	4.349	0.009102	4.291	0.0134	2.764	0.2228
Acuminoside	metab_39052	1.527	0.0001497	0.0001747	1.844	0.8064	3.304	0.1596	2.191	0.8289	5.007	0.02565	1.714	0.8595
S-Adenosylmethionine	metab_30442	1.526	2.359E-5	4.891E-5	6.559	0.01136	4.353	0.2098	2.7	0.5582	6.106	0.01709	2.506	0.09795
Trypanothione	metab_37109	1.526	1.807E-5	4.891E-5	1.694	0.8564	4.966	0.01378	4.764	0.05361	4.022	0.09703	3.875	0.1027
1-O,6-O-Bis(4-Hydroxy-Trans-Cinnamoyl)-Beta-D-Glucopyranose	metab_39198	1.524	1.325E-5	4.891E-5	5.063	0.007977	2.318	0.001168	3.127	0.3112	2.528	0.00371	2.433	0.01017
Pgp(Pge1/I-18:0)	metab_42990	1.524	1.99E-5	4.891E-5	5.541	0.01468	3.784	0.1873	3.964	0.1215	4.539	0.05644	5.232	0.01652
Ryanodine	metab_28352	1.524	1.325E-5	4.891E-5	1.154	0.9968	4.216	0.1115	6.017	0.1001	3.948	0.1029	4.84	0.06672
Bisdemethoxycurcumin	metab_37952	1.523	1.325E-5	4.891E-5	5.407	0.007069	5.72	0.009656	3.872	0.04029	4.884	0.01394	5.652	0.005778
Rollitacin	metab_41790	1.521	0.000485	0.0005242	3.738	0.124	3.387	0.3005	3.439	0.2395	3.28	0.08723	4.964	0.01707
Ser-Ile-His	metab_32925	1.521	1.325E-5	4.891E-5	3.536	0.1025	2.329	0.6204	4.759	0.02093	5.129	0.07415	4.485	0.04627
7-Glucosyl-11-Methylodeoside	metab_6936	1.519	0.001761	0.001829	5.761	0.01089	2.533	0.394	2.3	0.3028	2.181	0.006561	2.364	0.4267
Disperse Red 17	metab_34858	1.519	2.785E-5	5.105E-5	3.828	0.05855	4.336	0.03633	3.978	0.08241	5.293	0.01591	5.304	0.009364
3-Feruloyl-1-Sinapoyl Sucrose	metab_11661	1.519	7.233E-5	9.522E-5	5.22	0.0225	5.315	0.03326	4.367	0.1097	4.013	0.6555	3.297	0.1747
Cefminox	metab_49662	1.514	3.102E-5	5.388E-5	1.312	0.7808	4.494	0.03126	4.907	0.3238	1.116	0.003435	4.076	0.3508
Cichorioside J	metab_17729	1.511	1.421E-5	4.891E-5	6.13	0.007215	3.537	0.1266	1.677	0.002642	3.132	0.1936	2.081	0.2679
Rubiscolin-6	metab_45668	1.511	2.359E-5	4.891E-5	4.408	0.05157	4.342	0.09605	4.734	0.05648	5.165	0.01881	5.935	0.008311
[4-(1-Ethylimidazol-2-Yl)Piperazin-1-Yl]-(5-Methyl-1,2-Oxazol-3-Yl)Methanone	metab_22132	1.510	0.0001965	0.0002226	3.288	0.2332	2.771	0.2238	4.887	0.03469	3.549	0.2484	3.281	0.3353
Pa(6 Keto-Pgf1Alpha/8:0)	metab_37886	1.510	4.093E-5	6.269E-5	2.585	0.8892	4.432	0.05805	3.755	0.1981	3.501	0.2808	5.514	0.009692
Met Ile Phe	metab_27883	1.509	0.0001369	0.0001619	5.359	0.0851	5.405	0.1707	5.601	0.1781	5.047	0.2431	4.157	0.1345
Arginylvaline	metab_2763	1.508	1.99E-5	4.891E-5	2.495	0.1863	1.397	1.108	4.278	0.07198	4.988	0.2495	3.434	0.1219

Preladenant	metab_45015	1.508	1.9E-5	4.891E-5	3.247	0.05931	2.689	0.3306	5.2	0.01626	4.006	0.04015	4.534	0.03549
Methyl (Z)-9,10,11-Trihydroxyoctadec-12-Enoate	metab_39943	1.508	5.641E-5	7.816E-5	3.278	0.2103	4.67	0.05933	2.971	0.1259	3.969	0.1012	3.055	0.04299
Pyridoxine	metab_29022	1.507	1.807E-5	4.891E-5	3.368	0.1302	4.348	0.03444	4.6	0.02829	3.998	0.08775	3.116	0.2216
Asn Asp Val	metab_2789	1.506	4.947E-5	7.073E-5	3.612	0.3224	0.1582	0.2556	1.392	1.457	5.321	0.309	0.9585	1.386
Zizybeoside Ii	metab_42752	1.506	1.9E-5	4.891E-5	3.132	0.003273	3.055	0.001172	5.095	0.02759	3.266	0.003719	3.562	0.2301
Ile Trp	metab_10042	1.506	1.325E-5	4.891E-5	1.596	1.263	4.592	0.02104	5.146	0.01631	5.745	0.001971	4.854	0.02263
Cer(8:1_2O/14:1)	metab_14225	1.505	1.325E-5	4.891E-5	4.582	0.06413	4.855	0.03504	3.459	0.2513	4.16	0.1115	5.252	0.03152
Epsilon-Caprolactone	metab_23250	1.504	1.325E-5	4.891E-5	4.463	0.01435	4.572	0.01564	2.54	0.002691	5.367	0.01043	2.865	0.1703
Niazicinin	metab_30142	1.502	2.359E-5	4.891E-5	2.368	0.1225	2.789	0.766	5.597	0.03044	4.594	0.1354	4.812	0.03596
Ramiprilat	metab_35789	1.500	1.712E-5	4.891E-5	1.506	0.4907	4.462	0.05041	5.849	0.01024	2.071	0.9918	5.374	0.02166
Falecalcitriol	metab_41450	1.498	2.156E-5	4.891E-5	4.207	0.08926	4.933	0.027	3.721	0.1028	4.272	0.04778	3.026	0.01029
Flavonol 3-O-D-Glucoside	metab_13458	1.496	5.87E-5	8.067E-5	5.925	0.007849	3.006	0.2616	3.59	0.1489	3.142	0.1175	3.604	0.1706
Lys-Asn-Asn	metab_31443	1.496	2.535E-5	4.891E-5	1.4	0.9112	1.32	0.7843	3.686	0.07173	5.012	0.02157	3.363	0.1246
Deoxyadenosine	metab_2638	1.495	1.325E-5	4.891E-5	4.68	0.02989	5.035	0.02021	5.111	0.02907	5.698	0.006193	3.464	0.4339
3-Hydroxy-Hexadecanoic Acid	metab_16821	1.494	7.436E-5	9.684E-5	3.871	0.08849	5.327	0.02684	3.859	0.002698	5.189	0.0191	3.872	0.003261
Montirelin	metab_28290	1.492	3.78E-5	6.016E-5	4.017	0.1166	3.375	0.1874	1.88	1.502	5.367	0.5755	1.034	1.549
Reserpine	metab_38775	1.490	1.325E-5	4.891E-5	4.218	0.04596	4.84	0.02752	3.796	0.2024	4.655	0.01753	5.273	0.01172
Endomorphin-2	metab_44501	1.488	1.325E-5	4.891E-5	4.848	0.01987	5.474	0.0106	4.734	0.0119	5.427	0.01236	6.181	0.007682
Arctiin	metab_38893	1.484	0.0001388	0.0001638	6.144	0.01007	2.679	0.1435	2.702	0.006758	3.553	0.09399	2.714	0.1137
Alpha-L-Rhamnopyranosyl-(1->3)-Alpha-D-Galactopyranosyl-(1->3)-L-Fucose	metab_2984	1.483	2.359E-5	4.891E-5	5.005	0.02322	2.787	0.5906	2.558	0.4605	5.355	0.08579	1.224	0.003066
Melledonal B	metab_38697	1.482	1.325E-5	4.891E-5	5.819	0.0107	2.625	0.00117	2.773	0.00676	2.934	0.1417	2.74	0.01019
22-Deoxocucurbitacin D	metab_32786	1.481	1.325E-5	4.891E-5	2.654	0.003268	2.577	0.00117	5.122	0.0773	5.348	0.09222	3.136	0.2323
Glu-Trp-Gln	metab_45707	1.480	2.359E-5	4.891E-5	2.219	0.2117	2.738	0.5423	5.176	0.01286	4.582	0.03351	4.862	0.01416
Pgp(Pgd1/I-22:0)	metab_13709	1.479	1.325E-5	4.891E-5	6.075	0.01597	4.228	0.324	4.704	0.08172	5.143	0.02954	5.696	0.01381
Fluocinolone	metab_36353	1.478	3.32E-5	5.597E-5	2.754	0.00327	2.762	0.1377	5.155	0.01097	3.139	0.2187	4.056	0.06497
Piroxantrone	metab_49289	1.476	0.0005306	0.0005716	4.227	0.2893	2.506	0.7704	3.417	1.286	5.088	0.3699	4.544	0.6018
Thymolphthalein	metab_2722	1.474	4.381E-5	6.533E-5	4.709	0.03657	3.167	0.5042	2.632	0.5619	5.486	0.3944	4.087	0.5401
Becatecarin	metab_46810	1.474	5.812E-5	8.005E-5	5.946	0.005426	3.186	0.1489	2.601	0.134	2.541	0.00371	2.479	0.07473
Plantainoside C	metab_11772	1.471	0.0001126	0.0001373	5.487	0.008025	3.649	0.1772	2.599	0.7104	1.996	0.006538	2.21	0.4551
Galangin 7-Rhamnoside	metab_37473	1.471	5.314E-5	7.474E-5	5.609	0.007741	2.608	0.1634	2.338	0.05124	2.38	0.003706	2.305	0.05281
Oxoamide	metab_43924	1.471	2.411E-5	4.891E-5	5.723	0.008275	3.479	0.139	2.817	0.1273	4.286	0.03791	4.306	0.04592
Cucumerin B	metab_47639	1.468	2.271E-5	4.891E-5	5.1	0.01952	4.742	0.03093	4.823	0.02837	4.781	0.01976	3.4	0.1492
Ustiloxin D	metab_37619	1.468	2.455E-5	4.891E-5	3.043	0.3606	2.856	0.4386	5.497	0.01357	4.661	0.02577	4.73	0.01571
Urea, N'-(2,4-Difluorophenyl)-N-((4-(2,2-Dimethylpropyl)Phenyl)Methyl)-N-Heptyl-	metab_16414	1.466	2.49E-5	4.891E-5	4.652	0.05153	5.273	0.02852	4.985	0.08139	5.281	0.03529	3.741	0.1412
Caffeic Acid 3-Glucoside	metab_19366	1.466	2.402E-5	4.891E-5	5.217	0.009951	2.167	0.001847	2.244	0.002683	2.249	0.006567	2.257	0.003243

Gln-Ala-Lys	metab_17486	1.465	2.075E-5	4.891E-5	4.347	0.05095	4.715	0.02184	3.057	0.1277	5.968	0.01019	3.296	0.3298
1,8,10-Trihydroxy-3-Methoxy-6-Methyl-10-(3-Methylbut-2-Enyl)Anthracen-9-One	metab_13907	1.463	0.0007306	0.0007771	5.958	0.01196	2.928	0.1355	2.899	0.002695	2.904	0.006597	2.912	0.003258
Quercetin 3-O-Sophoroside	metab_36291	1.459	1.325E-5	4.891E-5	0.6684	0.002572	4.021	0.02036	4.776	0.01145	1.813	1.14	4.838	0.01011
L-Glutathione	metab_29310	1.459	1.325E-5	4.891E-5	6.521	0.003459	5.537	0.01441	3.37	0.002698	5.832	0.01697	3.663	0.158
S-Adenosylmethioninamine	metab_30635	1.458	1.325E-5	4.891E-5	4.899	0.03091	5.577	0.009701	3.436	0.1901	4.167	0.1396	5.012	0.03019
5,10-Methylene-Thf	metab_45483	1.457	1.325E-5	4.891E-5	4.976	0.03437	5.423	0.01691	4.81	0.04343	2.886	0.3853	3.899	0.1936
Myotoxin A	metab_36690	1.456	2.075E-5	4.891E-5	4.653	0.04076	4.604	0.0536	3.847	0.2444	5.368	0.01631	2.575	0.8215
Gibberellin A24	metab_35485	1.455	1.325E-5	4.891E-5	4.857	0.006222	3.913	0.06935	2.182	0.2521	4.326	0.03587	1.825	0.01212
2,3-Butanediol Glucoside	metab_33045	1.455	0.0001268	0.0001517	4.796	0.01016	1.266	0.7767	2.137	0.09488	5.081	0.03196	1.691	0.7512
Clopidogrel Acyl Glucuronide	metab_33313	1.455	1.325E-5	4.891E-5	2.799	0.00327	2.722	0.001171	4.965	0.03222	2.933	0.003717	3.988	0.1755
Rose Oxide (Cis)	metab_29301	1.455	3.36E-5	5.633E-5	3.088	0.04269	4.327	0.0865	3.426	0.1914	5.879	0.01927	3.206	0.1265
Benazepril	metab_33348	1.454	1.807E-5	4.891E-5	4.788	0.01658	3.81	0.04998	1.835	0.7753	5.287	0.01609	2.922	0.7761
Capromorelin	metab_38544	1.453	2.535E-5	4.891E-5	2.282	0.1838	2.42	0.4528	4.836	0.01689	4.97	0.008268	4.385	0.04055
Griseolic Acid	metab_49721	1.453	1.325E-5	4.891E-5	4.111	0.09584	5.104	0.01335	5.66	0.05484	2.353	0.003705	4.585	0.07808
Spb(14:0_2O)	metab_14043	1.453	2.49E-5	4.891E-5	5.428	0.01754	5.059	0.03778	3.003	0.002696	3.058	0.07311	3.016	0.003258
Biflavone	metab_28973	1.451	1.325E-5	4.891E-5	2.994	0.001283	2.983	0.001857	5.207	0.01796	3.065	0.006599	4.461	0.0674
Corymboside	metab_37434	1.449	1.325E-5	4.891E-5	5.057	0.02255	5.148	0.00829	4.466	0.01376	4.766	0.0258	3.399	0.2829
Enhydrin	metab_44665	1.449	1.518E-5	4.891E-5	3.092	0.003273	4.712	0.02397	4.656	0.02864	4.971	0.01317	3.406	0.2617
Sclareol	metab_16265	1.449	1.325E-5	4.891E-5	5.712	0.01495	5.13	0.07062	3.674	0.2034	5.521	0.01349	5.256	0.01352
(2S)-6-Amino-2-(Hexanoylamino)Hexanoic Acid	metab_36636	1.448	1.325E-5	4.891E-5	2.692	0.003269	2.615	0.00117	4.868	0.03007	2.907	0.1236	3.931	0.08351
Trp-Asp-Ile	metab_18549	1.446	1.325E-5	4.891E-5	3.891	0.1274	4.633	0.03107	4.544	0.03748	5.444	0.01033	3.114	0.25
3-Indolebutyric Acid	metab_9339	1.445	2.23E-5	4.891E-5	4.319	0.05473	3.082	0.2472	4.5	0.03517	3.232	0.2574	4.618	0.01918
3-(((4-Chlorophenyl)Sulfonyl)Methyl)-N-Hydroxybenzimidamide	metab_49754	1.444	2.535E-5	4.891E-5	2.256	0.1935	4.552	0.04342	5.77	0.01875	2.23	0.003699	4.856	0.04258
Oleuropein	metab_38788	1.444	2.359E-5	4.891E-5	5.569	0.01703	2.633	0.00117	2.883	0.1828	2.843	0.003716	2.748	0.01019
O-Desmethylvenlafaxine Glucuronide	metab_33909	1.442	1.325E-5	4.891E-5	4.261	0.01409	3.099	0.4047	5.48	0.01181	5.175	0.01283	4.499	0.03851
Leu Glu Asp Arg	metab_48992	1.442	1.325E-5	4.891E-5	4.469	0.03413	5.386	0.0135	5.124	0.01255	5.244	0.01809	3.978	0.1176
Lippioside I	metab_34508	1.441	3.668E-5	5.911E-5	5.071	0.0206	4.803	0.02327	4.839	0.0287	4.764	0.04124	3.493	0.1742
Gly-Ala-Ile	metab_33752	1.441	2.075E-5	4.891E-5	5.19	0.01368	2.145	0.8676	3.891	0.1093	5.716	0.02504	2.575	1.024
7,8-Dihydrovomifoliol 9-[Rhamnosyl-(1->6)-Glucoside]	metab_38428	1.440	0.0001388	0.0001638	5.135	0.019	3.259	0.2484	1.218	0.006357	1.372	0.1486	2.099	1.005
Ibuprofen Glucuronide	metab_42910	1.437	0.0003476	0.0003809	5.498	0.008865	2.629	0.184	3.13	0.1869	2.988	0.2333	2.958	0.2754
Beclomethasone Propionate	metab_48532	1.436	1.807E-5	4.891E-5	4.251	0.02875	3.001	0.2371	5.289	0.01218	5.034	0.01193	4.313	0.04642
(+)-Dehydrovomifoliol	metab_39393	1.436	1.325E-5	4.891E-5	6.214	0.006995	3.996	0.05362	5.128	0.01352	4.541	0.01971	4.78	0.01396
Periandrin V	metab_11552	1.436	2.969E-5	5.28E-5	5.781	0.006757	5.775	0.009572	5.66	0.01337	5.631	0.01799	4.39	0.2213
1-[(4-Amino-3-Methylphenyl)Methyl]-5-(2,2-Diphenylacetyl)-6,7-Dihydro-4H-Imidazo[4,5-C]Pyridine-6-Carboxylic Acid	metab_43231	1.435	1.325E-5	4.891E-5	4.606	0.03586	4.983	0.01662	4.294	0.04604	5.815	0.006385	5.497	0.01092
Kanamycin	metab_2564	1.434	3.448E-5	5.698E-5	3.023	0.09718	3.404	0.3576	5.985	0.01698	4.917	0.1447	5.005	0.03831

Retinoyl-Azt	metab_2162	1.434	3.047E-5	5.354E-5	2.276	0.001277	3.098	0.6593	5.669	0.009308	4.55	0.1288	4.697	0.0397
Coagulin R 3-Glucoside	metab_23393	1.433	5.314E-5	7.474E-5	1.545	0.1978	3.654	1.081	5.478	0.01611	1.978	0.9052	4.689	0.07866
Iodamide	metab_13216	1.433	1.99E-5	4.891E-5	2.896	0.1438	4.518	0.08655	5.28	0.01795	1.244	0.6568	4.635	0.1225
2-Ethoxy-6(5-Tetrazolyl)Xanthone	metab_20272	1.433	2.402E-5	4.891E-5	5.596	0.008288	2.681	0.001855	2.758	0.002694	2.763	0.006593	2.771	0.003256
Val-Asn-His	metab_28975	1.433	1.325E-5	4.891E-5	4.928	0.02664	3.777	0.1522	5.774	0.01329	5.151	0.01893	4.61	0.03508
Nodularin-R	metab_33863	1.433	2.075E-5	4.891E-5	1.359	0.815	4.4	0.08653	5.449	0.01504	1.902	1.345	5.091	0.01891
Momorcharaside B	metab_42843	1.431	1.9E-5	4.891E-5	4.779	0.06503	5.157	0.02769	4.846	0.01193	5.334	0.02572	3.619	0.06917
Des-Arg(9)-Bradykinin	metab_48233	1.431	1.99E-5	4.891E-5	3.448	0.1135	5.513	0.02073	5.385	0.01985	5.487	0.02326	4.365	0.0907
10-Hydroperoxy-H4-Neuroprostane	metab_34715	1.429	1.325E-5	4.891E-5	4.167	0.04586	3.59	0.2037	5.987	0.009892	5.254	0.006743	5.133	0.02185
Caffeoyl Quinic Acid	metab_46520	1.429	0.0001141	0.0001388	6.038	0.003845	3.209	0.3516	2.877	0.006762	2.939	0.003717	2.844	0.01019
Indole-3-Carboxaldehyde	metab_5675	1.429	1.325E-5	4.891E-5	4.851	0.01225	4.508	0.03077	5.354	0.006522	5.818	0.0105	3.532	0.09739
Ethyl Caffeate	metab_38427	1.428	2.56E-5	4.922E-5	5.525	0.01452	2.472	0.001169	2.636	0.04215	3.012	0.1731	2.847	0.1757
Glutamine-Glutamate	metab_35679	1.428	1.616E-5	4.891E-5	1.296	0.6167	4.227	0.056	5.232	0.01347	4.352	0.06981	4.769	0.01725
Ser-Tyr	metab_49713	1.427	2.535E-5	4.891E-5	1.973	0.183	1.879	0.6712	4.359	0.1233	4.896	0.0229	3.684	0.3786
10-Hydroxyligstroside	metab_43929	1.427	6.51E-5	8.763E-5	5.514	0.006527	2.535	0.00117	2.725	0.1046	2.892	0.1574	2.754	0.1588
Lobucavir	metab_37338	1.426	1.325E-5	4.891E-5	4.192	0.01889	2.409	0.001169	4.422	0.01323	4.898	0.009335	3.935	0.02707
Procyanidin Dimer B7	metab_33341	1.425	2.455E-5	4.891E-5	2.68	0.003269	2.604	0.00117	4.872	0.0513	3.571	0.1217	3.503	0.1381
N-Cyclopentyl-2-(Diethylamino)-2-(6-Nitro-1,3-Benzodioxol-5-Yl)Acetamide	metab_11722	1.424	1.325E-5	4.891E-5	2.16	0.9813	5.492	0.008246	6.193	0.007812	4.337	0.1275	5.92	0.01288
Tetraxetan	metab_44160	1.424	1.325E-5	4.891E-5	0.7153	0.002644	3.786	0.1117	4.703	0.009962	2.832	0.1043	4.467	0.02254
2-Hydroxy-4-Methylvaleric Acid	metab_44736	1.424	1.712E-5	4.891E-5	5.854	0.003794	6.157	0.002569	4.729	0.01435	5.749	0.008173	6.171	0.007029
Vigabatrin	metab_33218	1.423	2.455E-5	4.891E-5	5.209	0.005394	4.459	0.01988	3.186	0.129	5.981	0.03184	4.461	0.02138
Taxiphyllin	metab_28840	1.423	4.716E-5	6.838E-5	2.712	0.5937	4.456	0.05479	5.659	0.01414	2.27	0.589	4.474	0.05592
Ala-Ala-Ala	metab_9259	1.421	2.075E-5	4.891E-5	1.705	0.3208	2.622	0.7377	4.452	0.03082	5.054	0.01066	4.336	0.01219
Physangulide	metab_21774	1.420	1.325E-5	4.891E-5	4.449	0.05998	5.344	0.01031	6.238	0.01241	2.232	0.7623	5.932	0.01503
Islatravir	metab_29099	1.420	3.065E-5	5.374E-5	3.796	0.1845	3.363	0.2974	5.749	0.007343	4.766	0.03536	4.766	0.03556
Echinocystic Aicd	metab_42063	1.420	0.0001665	0.0001913	5.381	0.01979	2.451	0.08589	2.564	0.006753	2.693	0.0741	2.628	0.1323
Urceolide	metab_42796	1.419	4.55E-5	6.677E-5	5.825	0.0081	3.021	0.389	1.776	0.584	3.638	0.1289	1.872	0.6262
Ile Leu Gln	metab_25266	1.419	2.49E-5	4.891E-5	3.029	0.7749	3.262	0.79	5.496	0.03827	5.643	0.02128	5.318	0.03749
Z-Ile-Glu(O-T-Butyl)-Ala-Leucinal	metab_34895	1.418	1.9E-5	4.891E-5	1.896	0.1475	4.183	0.1973	5.63	0.005142	5.221	0.03153	5.189	0.009897
[3-(2-Aminopropyl)-6-Methylidenecyclohexa-1,3-Dien-1-Yl]Methanediol	metab_18843	1.416	1.9E-5	4.891E-5	3.39	0.1184	4.415	0.02269	3.302	0.002697	4.775	0.01679	4.259	0.01944
Gln-Phe-Leu	metab_43638	1.415	2.852E-5	5.163E-5	4.641	0.01285	1.442	0.001131	1.586	0.006594	4.657	0.02148	1.64	0.2118
Abexinostat	metab_42664	1.415	3.902E-5	6.072E-5	2.998	0.003272	3.293	0.2084	5.393	0.01163	3.161	0.04831	4.321	0.04432
H-Tyr-Gly-Gly-Oh	metab_32517	1.414	1.9E-5	4.891E-5	2.318	0.4421	1.661	0.5368	4.172	0.0851	5.044	0.06356	3.302	0.3315
Eujambolin	metab_44144	1.412	1.325E-5	4.891E-5	5.609	0.006224	2.752	0.001171	2.901	0.006763	2.963	0.003717	2.867	0.01019
N-Succinyl-L,L-2,6-Diaminopimelate	metab_42947	1.410	1.421E-5	4.891E-5	5.278	0.007048	2.427	0.04288	2.558	0.006752	2.62	0.003712	2.525	0.01018

2-Nitrophenyl A-D-Galactopyranoside	metab_30282	1.410	1.325E-5	4.891E-5	4.455	0.08028	5.097	0.02931	3.513	0.002698	5.63	0.01353	3.673	0.2347
Deoxynivalenol	metab_47126	1.407	1.754E-5	4.891E-5	2.966	0.1779	4.938	0.02392	4.264	0.06031	3.956	0.1218	3.597	0.2748
5'-Deoxyadenosine	metab_46888	1.407	1.325E-5	4.891E-5	4.088	0.02457	2.492	0.2231	4.421	0.0183	4.53	0.00735	3.943	0.01735
[(1R,5R)-5-(6-Aminopurin-9-Yl)Cyclohex-3-En-1-Yl]Methanol	metab_22310	1.406	3.34E-5	5.628E-5	3.726	0.2688	4.079	0.07634	3.148	0.1105	5.627	0.005316	4.104	0.06566
6-(4-O-Beta-D-Glucosyl-3-Methyl-Trans-But-2-Enyl-Amino)-Purine	metab_20200	1.406	0.0003469	0.0003802	1.805	0.001264	2.257	0.5447	2.182	0.5431	5.12	0.01785	2.707	0.647
Salidroside	metab_35005	1.406	1.325E-5	4.891E-5	6.353	0.004394	3.539	0.1043	2.704	0.2532	5.044	0.0134	2.499	0.01017
Lys-Trp	metab_25145	1.404	0.0001388	0.0001638	4.493	0.06882	2.426	1.414	1.426	1.003	5.675	0.02906	1.475	1
Eutypine	metab_12911	1.404	1.616E-5	4.891E-5	5.006	0.006785	2.858	0.1308	3.927	0.08351	3.536	0.09966	3.111	0.2022
Dg(11M3/9D3/0:0)	metab_14974	1.404	1.325E-5	4.891E-5	6.232	0.02345	5.316	0.05833	4.147	0.1675	6.36	0.02804	5.566	0.04024
Viloxazine	metab_45829	1.403	4.763E-5	6.883E-5	2.975	0.1911	3.009	0.2516	5.463	0.009367	4.475	0.04348	4.461	0.02761
Cdp-Dg(5-Iso Pgf2Vi/I-20:0)	metab_17853	1.403	0.0001402	0.0001653	3.641	0.5479	5.095	0.1048	3.733	0.3912	3.273	0.05763	5.512	0.06481
Thr-Tyr-Asn	metab_33215	1.401	3.69E-5	5.927E-5	0.7701	0.1775	2.245	0.5405	3.18	0.2989	4.952	0.2837	2.151	0.7636
Helenalin	metab_42618	1.401	1.325E-5	4.891E-5	3.924	0.03059	2.859	0.001172	4.592	0.02109	3.663	0.07813	4.092	0.02196
Leu-Thr-Tyr	metab_35820	1.401	2.455E-5	4.891E-5	6.042	0.004046	4.128	0.02765	5.274	0.01018	4.905	0.02838	5.272	0.01036
Canarigenin 3-[Glucosyl-(1->4)-6-Deoxy-Alloside]	metab_43752	1.399	1.754E-5	4.891E-5	3.161	0.1701	4.22	0.08335	3.637	0.2152	2.503	0.4124	5.384	0.01741
Harmine	metab_38225	1.399	2.411E-5	4.891E-5	2.07	0.3548	2.011	0.5274	4.565	0.01766	3.99	0.06337	3.512	0.1254
Parishin C	metab_44262	1.399	1.325E-5	4.891E-5	5.149	0.01575	2.344	0.001168	2.492	0.006749	2.554	0.003711	2.459	0.01017
Lysyl-Lysine	metab_4799	1.399	1.807E-5	4.891E-5	4.822	0.03522	4.908	0.02579	3.434	0.1077	6.228	0.01107	3.47	0.1614
Arg-Ser-Ile	metab_33146	1.398	1.325E-5	4.891E-5	2.579	0.07389	2.466	0.02256	4.664	0.007874	5.269	0.03684	4.203	0.07255
Trans-Zeatin-O-Glucoside Riboside	metab_43040	1.398	2.535E-5	4.891E-5	2.365	0.2397	2.673	0.594	4.899	0.01463	5.647	0.009473	4.242	0.04946
6''-O-Malonyldaidzin	metab_38411	1.397	1.325E-5	4.891E-5	5.918	0.006149	3.316	0.001173	3.464	0.006769	3.664	0.1089	4.094	0.08273
Decanoyl-L-Carnitine	metab_13925	1.396	1.807E-5	4.891E-5	4.837	0.01818	2.178	0.001847	2.453	0.4856	4.312	0.03909	3.234	0.2752
Aclacinomycin S	metab_43051	1.396	7.511E-5	9.767E-5	3.47	0.3081	2.973	0.301	4.908	0.02621	3.241	0.219	4.494	0.06401
Rimiterol	metab_48206	1.394	1.325E-5	4.891E-5	4.433	0.03091	3.928	0.06369	6.056	0.008182	5.128	0.01826	5.495	0.01132
3-Hydroxy-4-[3-(4-Methoxyphenyl)Propanoyl]Phenyl Beta-D-Mannopyranoside	metab_13733	1.393	0.0001569	0.0001819	5.677	0.01009	2.958	0.08837	3.114	0.06905	3.004	0.006598	3.172	0.2074
Coproporphyrinogen Iii	metab_38269	1.393	1.325E-5	4.891E-5	1.81	0.7372	4.895	0.01945	5.33	0.01899	3.912	0.07104	4.628	0.04632
Glu Leu Gly Lys Gln	metab_21735	1.393	2.156E-5	4.891E-5	4.713	0.1173	4.796	0.04674	4.205	0.1811	3.769	0.1246	5.873	0.02011
Felotaxel	metab_43545	1.392	3.902E-5	6.072E-5	5.347	0.009421	2.06	0.4114	2.066	0.05794	4.365	0.08332	2.006	0.0101
Fructosylvaline	metab_49934	1.391	1.325E-5	4.891E-5	3.1	0.003273	3.023	0.001172	4.996	0.06567	4.06	0.06056	3.45	0.1736
Olivil 4'-O-Glucoside	metab_46415	1.391	3.121E-5	5.392E-5	5.45	0.008168	2.719	0.2629	3.48	0.1338	3.608	0.2042	3.247	0.09951
4-Hydroxyphenylpyruvic Acid	metab_18586	1.390	0.0002052	0.0002315	5.027	0.003973	2.284	0.05697	2.337	0.002686	2.342	0.006575	2.35	0.003247
2-(3,4-Dicarboxy-3-Hydroxybutanoyl)Oxypropane-1,2,3-Tricarboxylic Acid	metab_32200	1.390	2.974E-5	5.28E-5	5.436	0.02647	3.268	0.2382	4.647	0.4823	5.71	0.3319	3.076	0.2035
Glu-Thr-Phe	metab_37218	1.389	2.271E-5	4.891E-5	3.035	0.23	2.647	0.2834	4.607	0.01011	3.388	0.1605	4.378	0.01676
3,4,5-Trimethoxy-N-Pyridin-2-Ylbenzamide	metab_28296	1.388	1.9E-5	4.891E-5	1.628	0.1087	2.477	0.9746	4.44	0.08688	4.97	0.0978	4.109	0.186
Pi(Pgf1Alpha/18:2(9Z,12Z))	metab_33716	1.386	1.421E-5	4.891E-5	5.446	0.0226	4.678	0.0401	4.943	0.0246	2.062	0.7266	5.386	0.02188

Acetylleucine	metab_44722	1.385	1.325E-5	4.891E-5	4.768	0.01281	5.26	0.006045	3.861	0.02215	5.628	0.006947	4.855	0.009635
2",4",6"-Triacetylglycitin	metab_27990	1.385	1.99E-5	4.891E-5	3.518	0.1579	5.093	0.02804	3.718	0.2205	4.27	0.1646	4.715	0.03501
(3Beta,6Alpha,19Alpha)-3,6,19-Trihydroxy-12-Ursen-28-Oic Acid	metab_40103	1.382	0.0005203	0.0005609	6.199	0.009608	3.343	0.2808	3.149	0.1499	3.514	0.1507	3.461	0.1399
2-[(3S)-1-(Oxan-4-Yl)Pyrrolidin-3-Yl]-6-(Trifluoromethyl)-1H-Benzimidazole	metab_11556	1.382	2.535E-5	4.891E-5	2.783	0.4148	3.565	0.1328	4.311	0.0177	5.234	0.0267	2.601	0.4214
Narirutin	metab_18935	1.382	0.0001746	0.0001993	5.39	0.0104	2.643	0.176	2.648	0.002693	2.653	0.00659	2.826	0.4054
Lnaps(3:0/N-22:2)	metab_47905	1.381	2.591E-5	4.929E-5	5.191	0.0109	3.739	0.1758	2.602	1.216	1.434	0.8402	4.185	0.0678
(2-Mercaptomethyl-3-Phenyl-Propionyl)-Glycine	metab_1238	1.380	1.325E-5	4.891E-5	6.226	0.01331	5.191	0.03266	3.399	0.002698	5.43	0.027	3.412	0.00326
Arg-Gly-Asp	metab_8788	1.380	2.359E-5	4.891E-5	3.545	0.1874	3.473	0.1442	2.358	0.002687	5.564	0.007638	2.379	0.01532
Penitrem A	metab_35106	1.378	1.9E-5	4.891E-5	4.632	0.04812	5.35	0.02361	4.143	0.09388	5.32	0.01384	3.716	0.249
Ht-2 Toxin	metab_8848	1.377	2.49E-5	4.891E-5	2.942	0.1809	2.767	0.001856	4.745	0.05932	5.484	0.009902	2.911	0.1319
Pyridinoline	metab_44861	1.377	1.325E-5	4.891E-5	1.991	0.9066	3.359	0.1408	5.493	0.004035	4.888	0.009271	4.806	0.0163
1,2-Diferuloylgentiobiose	metab_50126	1.374	1.9E-5	4.891E-5	4.12	0.003275	6.276	0.01594	4.704	0.2634	4.254	0.003721	5.848	0.1723
Methyltetrahydrofolic Acid	metab_46711	1.374	1.807E-5	4.891E-5	2.811	0.259	2.584	0.08261	4.945	0.01158	5.399	0.008494	4.064	0.05403
Etodolac	metab_32679	1.374	1.325E-5	4.891E-5	2.931	0.06098	2.592	0.1094	4.825	0.169	5.766	0.03268	4.123	0.3625
Sonolisib	metab_46461	1.373	1.325E-5	4.891E-5	1.892	0.2806	4.523	0.05728	4.674	0.02629	4.376	0.02915	3.998	0.05794
Kinetensin 4-8	metab_35153	1.373	2.881E-5	5.187E-5	5.298	0.01219	4.029	0.1645	5.323	0.01477	4.061	0.1134	4.82	0.04372
Gln-Thr-Ala	metab_613	1.371	2.411E-5	4.891E-5	4.653	0.05223	4.441	0.06838	5.568	0.01964	3.979	0.1202	4.404	0.05996
Gly Thr Lys	metab_17227	1.370	1.421E-5	4.891E-5	2.725	0.4422	4.277	0.07307	3.961	0.05733	4.118	0.07325	5.127	0.01071
Vicenin Iii	metab_37107	1.370	3.632E-5	5.862E-5	5.573	0.1483	5.554	0.1036	5.086	0.02318	4.858	0.2226	4.111	0.04787
(2Z)-3-[2-(Beta-D-Glucopyranosyloxy)Phenyl]Acrylic Acid	metab_35916	1.369	0.0005805	0.000623	5.182	0.004188	2.58	0.2477	2.677	0.2313	2.591	0.003712	2.495	0.01017
Brivanib	metab_36393	1.368	2.063E-5	4.891E-5	4.211	0.03982	3.543	0.1323	3.339	0.2361	1.092	1.369	4.715	0.004809
His-Ile	metab_29593	1.368	0.0001155	0.0001402	1.417	1.43	0.1554	0.113	2.803	1.321	4.615	0.04164	1.576	1.588
Val-Pro-Arg	metab_28924	1.367	2.359E-5	4.891E-5	3.785	0.2773	4.442	0.05862	5.296	0.01434	6.004	0.009569	3.584	0.2744
Goldinodox	metab_44796	1.367	1.325E-5	4.891E-5	4.328	0.0948	5.219	0.008195	5.018	0.02381	1.352	0.3702	5.254	0.01629
Hordatine B Glucoside	metab_37225	1.367	1.421E-5	4.891E-5	2.104	0.7838	4.936	0.02892	5.35	0.01615	4.328	0.08865	4.523	0.03864
Saquinavir-No	metab_37857	1.365	5.378E-5	7.549E-5	4.55	0.07343	4.38	0.09929	5.376	0.01195	4.539	0.05391	4.032	0.2429
Isohomovanillic Acid	metab_17589	1.363	2.992E-5	5.288E-5	4.647	0.0094	2.315	0.00185	2.822	0.2826	2.397	0.006578	3.01	0.1748
Phe-Gly-Leu	metab_37992	1.362	1.807E-5	4.891E-5	3.368	0.2472	2.878	0.3122	4.894	0.01567	5.304	0.008316	4.649	0.01366
Cortisone Acetate	metab_36461	1.360	1.325E-5	4.891E-5	2.942	0.2236	2.625	0.00117	4.899	0.01893	4.311	0.06461	3.661	0.1439
Lacto-N-Tetraose	metab_13595	1.359	5.314E-5	7.474E-5	3.656	0.09053	3.619	0.0369	5.428	0.02761	3.686	0.006603	4.324	0.1663
Asparaginylglutamine	metab_47040	1.358	3.894E-5	6.072E-5	3.693	0.1415	3.414	0.2791	3.128	0.2721	5.358	0.01466	4.146	0.04582
Folcepri	metab_47604	1.356	6.206E-5	8.437E-5	3.68	0.1116	5.207	0.02693	3.418	0.1629	3.361	0.00372	3.907	0.1825
4-[[2-(1-Hydroxybut-2-Enyl)-5-Oxooxolan-3-Yl]Amino]Benzoic Acid	metab_12380	1.356	4.424E-5	6.587E-5	4.848	0.01589	4.104	0.06631	2.522	0.6558	4.856	0.01454	2.273	0.5786
Methionine Ethyl Ester	metab_5066	1.355	1.325E-5	4.891E-5	4.651	0.01814	4.599	0.01596	2.987	0.05559	5.467	0.012	3.773	0.2271
Dimethyl Sulfoxide	metab_1213	1.355	0.004199	0.004294	4.806	0.4275	2.686	0.3924	3.133	0.8118	2.411	0.006579	2.419	0.003249

N-Acetylserotonin Glucuronide	metab_34341	1.354	2.91E-5	5.21E-5	2.883	0.1165	3.565	0.0868	5.637	0.01027	3.584	0.3208	4.933	0.01967
Lisinopril, Epsilon-Biotinamidocaproyl-	metab_44091	1.354	2.411E-5	4.891E-5	4.089	0.1002	4.144	0.0944	5.359	0.02564	2.219	0.7878	4.848	0.01468
N-(1-Benzylpiperidin-4-Yl)-6-Phenylthieno[3,2-D]Pyrimidin-4-Amine	metab_36827	1.353	2.898E-5	5.207E-5	3.048	0.1969	2.706	0.1376	4.441	0.02868	5.183	0.008242	4.44	0.03558
Methyl 7-Epi-12-Hydroxyjasmonate Glucoside	metab_36327	1.353	1.421E-5	4.891E-5	3.074	0.02768	4.304	0.04584	6.32	0.01385	4.388	0.02722	5.603	0.009102
Petromyzonol Sulfate	metab_44293	1.353	1.99E-5	4.891E-5	3.047	0.2394	2.678	0.4083	4.925	0.01609	5.585	0.01246	4.193	0.05706
Chrysosplenol D	metab_36808	1.352	0.0001141	0.0001388	5.33	0.01374	2.699	0.3334	2.707	0.006758	2.769	0.003715	2.674	0.01018
Rutinose	metab_31978	1.351	1.325E-5	4.891E-5	6.073	0.01963	3.451	0.001173	3.6	0.00677	3.662	0.00372	3.566	0.0102
Methionyl-Lysine	metab_33279	1.351	1.518E-5	4.891E-5	3.006	0.1634	2.581	0.2592	4.565	0.03449	5.309	0.03345	4.257	0.05807
4-O-Beta-D-Glucosyl-4-Coumaric Acid	metab_46045	1.351	6.747E-5	9.01E-5	5.072	0.006624	2.43	0.09988	2.542	0.05346	2.663	0.05674	2.483	0.01017
Gamma-Delta-Dioxovaleric Acid	metab_29271	1.348	1.616E-5	4.891E-5	5.027	0.01418	4.39	0.01928	2.508	0.01671	4.44	0.02717	3.724	0.06286
Cis-Mulberroside A	metab_19447	1.348	2.402E-5	4.891E-5	5.478	0.01318	2.895	0.001857	2.972	0.002696	2.977	0.006598	2.986	0.003258
D-1-[(3-Carboxypropyl)Amino]-1-Deoxyfructose	metab_50406	1.347	3.121E-5	5.392E-5	3.334	0.003274	3.301	0.1062	5.272	0.03899	3.586	0.183	4.364	0.184
Cyclosquamosin D	metab_38511	1.345	1.712E-5	4.891E-5	2.846	0.09986	4.156	0.07952	4.517	0.07347	3.097	0.2064	5.654	0.00988
Damascenone	metab_13317	1.345	7.261E-5	9.552E-5	4.903	0.01559	2.328	0.001851	2.428	0.05493	2.439	0.07422	2.418	0.003249
Cer(18:0_3O/18:2_(2Oh))	metab_41551	1.344	3.177E-5	5.469E-5	4.285	0.1215	3.95	0.2378	3.563	0.3297	4.452	0.0787	4.787	0.0546
Methyl Gallate	metab_35915	1.343	0.0001645	0.0001894	4.852	0.2957	3.908	0.4202	3.613	0.5145	2.777	0.8103	2.615	0.2443
Monomethyl Phthalate	metab_33703	1.343	1.325E-5	4.891E-5	5.944	0.001974	6.004	0.01515	5.274	0.02489	2.552	0.2929	5.328	0.007013
Carapin	metab_37921	1.342	2.075E-5	4.891E-5	2.687	0.06209	2.57	0.00117	4.488	0.007991	5.022	0.01237	2.763	0.1179
4-(4-Benzyl-3-Oxoquinoxalin-2-Yl)-N-(3-Ethoxypropyl)Piperazine-1-Carboxamide	metab_2452	1.341	1.325E-5	4.891E-5	3.723	0.02961	3.7	0.001859	5.696	0.01083	5.067	0.1109	4.192	0.1612
Ile-Glu-Thr-Asp-Fluoromethyl Ketone	metab_46452	1.341	4.753E-5	6.873E-5	2.929	0.003272	3.015	0.1846	5.244	0.01327	4.289	0.07316	4.321	0.04114
2-(4-Amino-1-Isopropyl-1H-Pyrazolo[3,4-D]Pyrimidin-3-Yl)-1H-Indol-5-Ol	metab_11314	1.341	2.515E-5	4.891E-5	2.879	0.1264	2.922	0.21	3.706	0.1291	5.45	0.005741	4.193	0.04119
Val Arg Val	metab_28319	1.341	1.616E-5	4.891E-5	2.654	0.1565	3.171	0.3413	5.16	0.04388	5.893	0.01219	4.412	0.04388
Ps(Pgf2Alpha/16:1(9Z))	metab_44313	1.339	1.518E-5	4.891E-5	4.205	0.03097	4.686	0.07354	5.563	0.01517	3.27	0.1889	4.433	0.1069
Hovenidulcioside A1	metab_35154	1.339	1.807E-5	4.891E-5	2.56	0.2639	5.163	0.02681	5.544	0.01491	2.701	0.2817	5.855	0.009923
Bellericagenin B	metab_24788	1.339	4.337E-5	6.498E-5	3.969	0.443	5.297	0.02608	4.272	0.1686	4.516	0.1747	5.578	0.02059
(1S,2S,4R,8R)-P-Menthane-1,2,8,9-Tetrol	metab_37383	1.337	9.644E-5	0.0001204	4.59	0.008647	2.018	0.09758	2.125	0.00672	2.198	0.02777	2.092	0.01012
Linalool 3,7-Oxide Beta-Primeveroside	metab_44335	1.337	1.807E-5	4.891E-5	5.673	0.00897	3.429	0.3901	4.306	0.06233	4.085	0.02134	3.779	0.1224
N-Lactoyl-Tryptophan	metab_45480	1.337	1.325E-5	4.891E-5	3.197	0.149	4.386	0.01128	4.039	0.04421	4.726	0.01206	5.024	0.01146
Semustine	metab_43930	1.337	1.421E-5	4.891E-5	3.576	0.1507	0.9785	0.00105	2.069	1.06	4.638	0.03701	1.083	0.00935
Furazabol	metab_11245	1.336	8.511E-5	0.0001086	3.692	0.1728	4.738	0.01116	3.756	0.03963	5.526	0.008304	3.599	0.1689
Val-His	metab_2419	1.336	1.9E-5	4.891E-5	5.401	0.01315	5.004	0.03624	5.424	0.02057	5.742	0.01425	3.987	0.1105
3A-Methyl-2,3,4,5,5A,10,10A,10B-Octahydro-1H-Cyclopenta[A]Fluorene-2,3,7-Triol	metab_49280	1.335	1.712E-5	4.891E-5	1.746	1.926	4.323	0.04965	5.732	0.05337	4.894	0.1412	5.181	0.07709
7-Hydroxycoumarine	metab_19585	1.335	2.402E-5	4.891E-5	5.148	0.007172	2.616	0.001855	2.693	0.002693	2.698	0.006591	2.706	0.003255
4-Hydroxy-17Beta-Estradiol-2-S-Glutathione	metab_48508	1.335	2.075E-5	4.891E-5	5.13	0.02389	5.45	0.006808	5.149	0.01278	5.733	0.01174	4.048	0.2711
Cryptomeridiol 11-Rhamnoside	metab_41985	1.334	1.421E-5	4.891E-5	3.912	0.07454	4.813	0.03071	3.552	0.319	4.216	0.0485	4.731	0.01827

2-(Beta-D-Mannopyranosyl)-L-Tryptophan	metab_48580	1.334	2.53E-5	4.891E-5	2.575	0.0934	2.608	0.1814	4.892	0.0209	4.514	0.0264	3.875	0.07852
Micronomicin	metab_22857	1.334	1.325E-5	4.891E-5	4.15	0.2073	5.842	0.01431	5.355	0.02974	4.847	0.03313	4.644	0.1084
6-Dimethylaminopurine	metab_2784	1.333	3.497E-5	5.736E-5	4.955	0.004023	4.383	0.01564	2.511	0.259	4.571	0.5567	3.758	0.3771
Deflazacort	metab_35822	1.333	1.325E-5	4.891E-5	3.691	0.09929	3.082	0.2065	4.625	0.01268	3.981	0.06882	4.677	0.02475
Ala Asn Glu Arg	metab_29698	1.332	4.286E-5	6.445E-5	4.953	0.03449	2.209	0.6221	3.368	0.6938	5.142	0.02306	2.297	0.6204
7,8-Dihydrobuddlenol B	metab_24184	1.332	2.411E-5	4.891E-5	4.271	0.1243	5.628	0.02825	4.897	0.1092	5.549	0.01612	4.376	0.2279
Gln-Ser-Asn	metab_50461	1.331	1.325E-5	4.891E-5	2.565	0.6199	4.453	0.1454	5.075	0.07116	5.528	0.0143	5.304	0.0694
Levomefolic Acid	metab_33666	1.331	1.325E-5	4.891E-5	2.773	0.00327	3.271	0.1874	5.45	0.009198	4.849	0.03196	4.704	0.02988
Lampranthin Ii	metab_45221	1.329	3.933E-5	6.111E-5	4.805	0.16	4.759	0.1227	4.036	0.1008	3.659	0.3468	3.248	0.0102
Val-Pro	metab_48952	1.329	1.712E-5	4.891E-5	2.713	0.154	2.256	0.2845	4.286	0.03995	5.385	0.04904	3.67	0.1073
Verbenalin	metab_46103	1.328	1.325E-5	4.891E-5	5.106	0.01013	2.574	0.00117	2.723	0.006758	2.785	0.003715	2.689	0.01019
Mesaconitine	metab_45496	1.327	2.53E-5	4.891E-5	4.502	0.04659	4.891	0.04621	3.395	0.1202	4.848	0.0187	4.375	0.07297
Avenestergenin A1	metab_43813	1.327	2.535E-5	4.891E-5	2.897	0.1366	4.82	0.01106	4.073	0.06383	2.915	0.03666	3.559	0.07852
2-Norbornene-5,6-Dicarboxylic Anhydride	metab_12456	1.326	0.00077	0.0008174	3.176	0.1313	3.257	0.1111	3.41	0.1332	3.272	0.1175	4.522	0.01399
Arctignan C	metab_29647	1.325	2.774E-5	5.099E-5	3.742	0.0787	5.138	0.04432	4.147	0.1061	4.903	0.03484	3.995	0.1835
Ala-Met	metab_32780	1.323	1.325E-5	4.891E-5	3.184	0.1809	2.632	0.2897	4.683	0.1241	5.446	0.06462	4.155	0.1082
Ganosporelactone A	metab_13700	1.323	2.49E-5	4.891E-5	5.332	0.03337	3.891	0.09021	3.922	0.138	4.41	0.1015	4.934	0.05063
Apigenin-7-Apioglucoside	metab_38477	1.322	0.000567	0.000609	4.838	0.2519	4.163	1.069	2.717	0.6515	3.249	1.006	2.115	0.01013
4'-Demethylepipodophyllotoxin	metab_47718	1.322	1.325E-5	4.891E-5	1.674	0.03071	4.236	0.02495	4.796	0.01814	2.331	0.4073	5.03	0.007513
2-(Benzylanilino)-1-[1,1'-Biphenyl]-4-Yl-1-Ethanone	metab_2595	1.322	1.325E-5	4.891E-5	2.652	0.2137	5.398	0.03926	5.599	0.02989	4.625	0.1639	5.132	0.04621
Hexosyl Lpe 18:3	metab_42124	1.322	2.359E-5	4.891E-5	4.241	0.003275	4.272	0.07061	6.086	0.01472	4.375	0.003721	5.131	0.01937
(5E,7E)-Undeca-2,5,7-Trienedioylcarnitine	metab_39257	1.322	0.0007537	0.0008009	5.404	0.01147	2.76	0.187	2.291	0.4797	2.928	0.3374	2.868	0.07825
Creatine	metab_825	1.322	0.0002097	0.0002362	5.286	0.2097	4.813	0.3587	3.256	0.4651	3.474	0.672	2.97	0.00578
8-O-Acetyl Shanzhiside Methyl Ester	metab_46074	1.321	1.518E-5	4.891E-5	5.121	0.00425	2.703	0.4845	3.802	0.07757	4.292	0.01142	3.59	0.1225
5-Caffeoylshikimic Acid	metab_45262	1.321	1.325E-5	4.891E-5	5.088	0.01089	2.583	0.00117	2.731	0.006759	2.793	0.003715	2.698	0.01019
Thr-Asp	metab_31924	1.320	1.325E-5	4.891E-5	4.03	0.1335	3.493	0.01911	5.429	0.02489	6.299	0.01849	5.025	0.04884
Thromboxane B2	metab_25580	1.318	1.518E-5	4.891E-5	4.939	0.05773	4.452	0.1193	6.427	0.005386	5.608	0.03419	5.557	0.009028
Tamoxifen-Ol	metab_51132	1.318	2.411E-5	4.891E-5	5.249	0.02224	2.978	0.3871	3.743	0.04965	5.078	0.03597	2.351	1.225
Tirofiban	metab_47629	1.318	1.325E-5	4.891E-5	5.397	0.01173	4.271	0.04555	2.502	0.1197	4.796	0.02165	3.46	0.2363
Musabalbisiane C	metab_43019	1.317	2.455E-5	4.891E-5	5.025	0.01349	3.543	0.2773	4.347	0.04639	3.461	0.1618	4.647	0.01919
7A,12A-Dihydroxy-5B-Cholestan-3-One	metab_41531	1.315	7.601E-5	9.87E-5	4.804	0.02922	1.954	0.1816	2.255	0.2952	3.141	0.2346	2.116	0.3703
Jaceosidin	metab_42822	1.315	1.325E-5	4.891E-5	5.377	0.008353	5.521	0.01656	5.073	0.009923	5.162	0.02334	4.189	0.01659
Trans-3-Indoleacrylic Acid	metab_5677	1.314	1.325E-5	4.891E-5	6.299	0.01103	5.982	0.009304	6.767	0.004005	7.211	0.004797	5.193	0.0119
Epimedin B	metab_48214	1.313	1.712E-5	4.891E-5	4.727	0.02486	5.117	0.01792	4.601	0.05665	4.858	0.07353	3.747	0.217
Senegenin	metab_14594	1.313	1.325E-5	4.891E-5	7.202	0.008613	5.435	0.06274	5.74	0.01961	4.56	0.1236	5.654	0.03311

Phenethyl Sophoroside	metab_37333	1.312	1.712E-5	4.891E-5	5.225	0.01151	2.402	0.001169	2.757	0.2741	3.748	0.1759	2.551	0.08568
Rifametane	metab_13894	1.312	6.331E-5	8.583E-5	3.524	0.09918	3.516	0.159	5.351	0.03654	3.589	0.09716	4.625	0.1268
21-Deoxycortisol	metab_44286	1.312	1.325E-5	4.891E-5	3.99	0.0405	2.716	0.189	4.765	0.02329	5.345	0.02239	3.819	0.07272
Indicine	metab_35898	1.311	2.359E-5	4.891E-5	2.803	0.1421	5.334	0.008236	5.528	0.01567	3.014	0.2524	6.005	0.009669
(+/-)-Tryptophan	metab_5701	1.311	1.325E-5	4.891E-5	5.936	0.009981	5.622	0.01522	6.42	0.005364	6.874	0.006066	4.845	0.01807
Lpe(O-16:1)	metab_41797	1.309	0.0001187	0.0001435	4.42	0.2729	3.896	0.6697	2.218	0.1448	3.069	0.7495	1.973	0.4753
Ganoderic Acid X	metab_45919	1.309	1.325E-5	4.891E-5	5.07	0.008458	4.291	0.02634	5.817	0.0116	4.734	0.02323	5.304	0.008787
Hexosyl Lpe(18:2)	metab_40310	1.308	1.325E-5	4.891E-5	4.057	0.09604	5.41	0.01468	6.424	0.01186	3.185	0.1708	5.901	0.01192
Isoflupredone Acetate	metab_17430	1.308	2.359E-5	4.891E-5	2.606	0.001281	2.872	0.444	4.983	0.02476	5.227	0.02249	4.188	0.09373
Hydroxydesmthyl Doxepin Glucuronide	metab_47249	1.307	1.325E-5	4.891E-5	2.183	0.769	3.935	0.06413	5.402	0.009794	4.899	0.03524	5.104	0.02467
Amoxicilloyl	metab_34959	1.306	1.325E-5	4.891E-5	3.888	0.198	2.999	0.001172	5.092	0.02468	5.548	0.007371	4.299	0.05933
Decenoylcarnitine	metab_42362	1.305	3.902E-5	6.072E-5	5.51	0.01109	2.679	0.1106	2.782	0.00676	3.992	0.04868	2.749	0.01019
Aclacinomycin A Zwitterion	metab_37428	1.305	5.076E-5	7.214E-5	5.398	0.01154	2.443	0.3688	2.987	0.5219	4.521	0.06158	2.832	0.4794
Hgluvalleupnsaspalaglupheoh	metab_44305	1.304	1.754E-5	4.891E-5	2.926	0.08585	3.627	0.2355	5.409	0.03052	3.245	0.1767	4.989	0.02315
Dodecyl Gallate	metab_40470	1.304	1.325E-5	4.891E-5	4.793	0.02393	4.482	0.02764	3.416	0.07466	5.061	0.01858	4.597	0.01665
Methylisopelletierine	metab_39405	1.302	1.325E-5	4.891E-5	4.207	0.01403	3.864	0.03117	2.898	0.1655	4.898	0.01118	3.998	0.03298
Cycloheximide	metab_42551	1.301	1.325E-5	4.891E-5	5.341	0.008427	2.557	0.00117	2.706	0.006758	3.755	0.0833	2.673	0.01018
Epothilone D	metab_37836	1.301	1.325E-5	4.891E-5	4.262	0.04076	3.914	0.06761	6.012	0.006419	5.636	0.01284	5.2	0.01157
Ser-Thr	metab_50472	1.300	1.325E-5	4.891E-5	2.92	0.003271	2.843	0.001171	4.575	0.05933	5.513	0.01679	4.293	0.09814
Alliosterol 1-Rhamnoside 16-Galactoside	metab_47817	1.300	1.325E-5	4.891E-5	5.698	0.0066	4.857	0.03306	4.762	0.0387	2.503	0.4545	5.353	0.01806
Isoacteoside	metab_11529	1.299	0.002261	0.002336	5.743	0.02729	2.75	0.6371	2.156	0.926	3.222	0.4786	2.494	1.318
Sophoraflavanone G	metab_23966	1.299	1.712E-5	4.891E-5	3.246	0.2343	3.894	0.2683	5.832	0.01374	4.159	0.08707	4.779	0.04245
Asp-Gly-Leu	metab_47348	1.298	1.325E-5	4.891E-5	4.871	0.01953	2.74	0.001171	3.882	0.05758	4.453	0.0343	2.892	0.09206
Fahfa(18:3/3:0)	metab_40505	1.298	1.518E-5	4.891E-5	4.422	0.02613	3.541	0.206	5.028	0.02794	4.37	0.009948	4.824	0.02932
Deoxyloganin	metab_920	1.298	3.455E-5	5.698E-5	3.534	0.001284	3.58	0.0902	5.555	0.01412	4.323	0.2362	4.561	0.08372
Butyloxycarbonyl-Leucyl-Glycyl-Arginine-4-Nitroanilide	metab_45867	1.298	2.156E-5	4.891E-5	2.991	0.003272	2.914	0.001172	4.954	0.02105	4.059	0.07243	3.964	0.1069
N-(1-Deoxy-1-Fructosyl)Alanine	metab_49526	1.296	1.616E-5	4.891E-5	3.436	0.0517	3.814	0.05966	2.864	0.1511	5.408	0.06677	3.088	0.09788
Carvone	metab_12078	1.296	9.087E-5	0.0001148	4.368	0.01183	1.935	0.001838	2.062	0.1251	2.147	0.1859	2.024	0.003231
Pengitoxin	metab_12395	1.295	2.576E-5	4.929E-5	5.919	0.01459	3.196	0.2762	4.049	0.2329	5.323	0.03177	3.666	0.285
Tyr Asp	metab_2180	1.294	1.325E-5	4.891E-5	2.775	0.1393	4.389	0.0777	5.141	0.02513	6.041	0.006053	4.854	0.02092
Forskolin	metab_47184	1.294	1.325E-5	4.891E-5	4.227	0.04957	3.91	0.08872	5.94	0.005821	5.292	0.02404	5.052	0.01474
N-(2-Hydroxy-2-Phenylpropyl)-2-(4-Propan-2-Ylsulfanylphenyl)Acetamide	metab_2755	1.294	1.325E-5	4.891E-5	2.331	0.001278	2.32	0.00185	4.341	0.1939	5.171	0.08282	3.158	0.3319
Neuraminic Acid	metab_30311	1.294	4.184E-5	6.344E-5	5.275	0.03059	3.692	0.1909	4.699	0.05514	4.722	0.05522	3.57	0.1031
Ile-Asn	metab_32007	1.294	2.359E-5	4.891E-5	3.217	0.003273	3.412	0.2842	5.305	0.06847	5.587	0.04514	4.904	0.06655
10-Hydroxy-8-Nor-2-Fenchanone Glucoside	metab_45594	1.293	5.251E-5	7.419E-5	2.898	0.5823	5.039	0.01773	3.659	0.2831	3.301	0.7039	4.731	0.02209

Glu-Met	metab_47499	1.293	1.325E-5	4.891E-5	3.036	0.13	3.873	0.05704	5.474	0.004296	5.628	0.008704	5.271	0.007888
[1-(Tert-Butylcarbamoyl)Piperidin-4-Yl] Acetate	metab_23367	1.293	2.204E-5	4.891E-5	3.943	0.07788	4.127	0.1286	2.932	0.3282	5.464	0.009601	3.314	0.1468
L-Tert-Leucine	metab_2871	1.293	3.902E-5	6.072E-5	4.823	0.01026	2.261	0.2145	2.25	0.002684	5.028	0.08326	2.356	0.1705
N-(5-(2,5-Dioxopyrrolidin-1-Yl)Pentyl)-N-Hydroxyacetamide	metab_20202	1.292	4.697E-5	6.825E-5	3.446	0.2644	3.675	0.1133	2.748	0.4149	5.314	0.01145	2.511	0.273
Bufotenine O-Glucoside	metab_43759	1.292	1.325E-5	4.891E-5	2.438	0.003263	2.839	0.2158	4.851	0.02408	5.168	0.006853	3.97	0.03202
Fisetin	metab_42050	1.292	0.0001003	0.0001248	5.37	0.3648	4.505	0.8591	3.56	0.7203	4.028	0.7912	2.706	0.01019
Scaposin	metab_45386	1.292	0.0006239	0.0006676	4.972	0.01127	2.589	0.1707	2.593	0.02267	2.644	0.003713	2.549	0.01018
(E)-6-Hydroxy-6-(1-Hydroxy-4-Methylcyclohex-3-En-1-Yl)-2-Methylhept-2-Enoic Acid	metab_36894	1.292	0.0002265	0.000254	2.34	0.02215	2.295	0.09782	2.403	0.006744	5.233	0.00614	2.52	0.205
Glu-Glu-Gln	metab_28780	1.292	1.325E-5	4.891E-5	2.564	0.001281	3.011	0.1279	5.035	0.04273	4.278	0.03166	4.419	0.04779
Epothilone A	metab_43597	1.291	1.325E-5	4.891E-5	4.836	0.02559	3.997	0.02704	3.134	0.2434	5.391	0.009688	4.231	0.03473
N-Methylthreonine	metab_11049	1.291	2.359E-5	4.891E-5	3.824	0.01948	4.149	0.02249	2.82	0.3312	4.327	0.02632	2.623	0.2175
Pgp(Pgj2/I-15:0)	metab_39012	1.291	2.49E-5	4.891E-5	2.846	0.6971	5.044	0.01429	5.562	0.023	2.79	0.7956	4.733	0.04076
Phyllanthusol A	metab_9983	1.290	1.712E-5	4.891E-5	5.334	0.05788	5.917	0.01078	5.156	0.05276	4.346	0.1832	4.538	0.1049
Phe-His-Ser	metab_23337	1.289	1.807E-5	4.891E-5	4.364	0.03897	3.508	0.1988	4.167	0.09246	5.455	0.01527	2.539	0.8508
Methyl (R)-9-Hydroxy-10-Undecene-5,7-Diynoate Glucoside	metab_8789	1.289	2.535E-5	4.891E-5	4.511	0.05423	4.208	0.04561	3.104	0.2111	5.794	0.01021	2.971	0.003258
Leucyl-Arginine	metab_49924	1.289	2.535E-5	4.891E-5	3.064	0.003273	3.067	0.1042	4.906	0.06431	5.428	0.0665	4.54	0.09611
Melatonin Radical	metab_34820	1.289	1.325E-5	4.891E-5	4.626	0.01928	4.842	0.01134	5.544	0.005149	5.401	0.006172	4.167	0.04638
Solasonine	metab_10077	1.289	9.196E-5	0.000116	6.028	0.01693	4.123	0.2261	4.066	0.3531	3.734	0.1545	4.859	0.1383
Lucuminamide	metab_50144	1.288	2.075E-5	4.891E-5	3.074	0.1448	2.929	0.001172	4.627	0.3738	5.63	0.1909	3.045	0.0102
L-Pyridosine	metab_38360	1.288	1.518E-5	4.891E-5	3.781	0.2872	3.064	0.214	5.125	0.01353	5.335	0.005868	4.392	0.05125
Sacubitrilat	metab_37251	1.288	3.065E-5	5.374E-5	1.79	0.3489	3.036	0.3053	4.754	0.007442	2.564	0.6341	4.377	0.01623
Glu-Thr	metab_31977	1.288	1.325E-5	4.891E-5	3.425	0.01034	3.347	0.005677	5.231	0.05085	6.091	0.0496	4.649	0.08457
L-Tryptophan	metab_34797	1.287	1.325E-5	4.891E-5	5.419	0.006968	5.08	0.01372	5.872	0.004553	6.286	0.008003	4.354	0.01408
Fluphenazine Enanthate	metab_35149	1.286	1.325E-5	4.891E-5	4.877	0.01734	2.983	0.1787	4.648	0.07569	5.532	0.01898	3.706	0.1636
Trp-Asp-Leu	metab_38619	1.286	1.325E-5	4.891E-5	5.257	0.02328	4.278	0.03128	4.148	0.04638	5.102	0.01333	2.983	0.2206
Kudzusaponin Sa4	metab_38112	1.286	1.421E-5	4.891E-5	3.879	0.2611	4.747	0.03676	5.778	0.0127	2.399	1.149	5.425	0.01496
Urofollitropin	metab_8256	1.286	1.9E-5	4.891E-5	5.573	0.04881	6.794	0.003696	6.503	0.01647	5.552	0.006605	5.695	0.0295
Epigallocatechin	metab_2223	1.286	1.807E-5	4.891E-5	5.69	0.0115	4.594	0.06847	4.683	0.05705	3.235	0.006601	4.176	0.09555
Ehretioside B	metab_1977	1.285	1.518E-5	4.891E-5	4.498	0.05598	4.398	0.04971	6.064	0.0102	5.169	0.01783	4.809	0.02498
Ethyl 3-Phenylglycidate	metab_9952	1.285	1.518E-5	4.891E-5	4.222	0.0595	3.531	0.1531	2.718	0.1694	4.746	0.01448	3.836	0.1094
Ganoderic Acid N	metab_37488	1.285	2.298E-5	4.891E-5	5.153	0.01091	5.067	0.02094	4.921	0.02657	4.939	0.01892	3.923	0.06546
Malvidin 3-Rhamnoside	metab_43263	1.285	2.359E-5	4.891E-5	3.678	0.003275	3.601	0.001173	4.972	0.004628	3.812	0.003721	3.81	0.07491
Ancymidol	metab_43391	1.285	1.325E-5	4.891E-5	3.14	0.003273	3.785	0.06486	3.989	0.01924	3.413	0.1111	5.087	0.0115
Mgdg(2:0/13:1)	metab_42473	1.284	0.003155	0.003239	5.515	0.02517	3.172	0.264	2.949	0.3116	2.908	0.3471	3.032	0.2746
Curcumenol	metab_8498	1.284	2.113E-5	4.891E-5	3.888	0.1468	4.094	0.0451	3.126	0.08328	5.705	0.01462	3.532	0.2008

Prizidilol	metab_11613	1.283	2.298E-5	4.891E-5	3.966	0.08469	4.81	0.03097	4.288	0.07873	4.261	0.06233	3.585	0.2486
Gingerglycolipid C	metab_9479	1.283	1.9E-5	4.891E-5	5.665	0.02119	4.617	0.05865	4.684	0.06529	2.69	0.2372	5.106	0.03652
Ile-Ile-His	metab_28245	1.283	3.639E-5	5.871E-5	4.068	0.3051	3.214	0.3722	4.86	0.5225	6.204	0.01741	4.754	0.5645
7,8 Dihydrokawain	metab_17735	1.282	0.0004645	0.0005028	4.879	0.02423	2.606	0.1107	2.769	0.1458	2.642	0.00659	2.681	0.0761
Tryptophyl-Isoleucine	metab_44719	1.282	1.325E-5	4.891E-5	2.436	0.02642	4.283	0.02926	5.061	0.005067	5.487	0.004406	4.714	0.02996
Acetildenafil	metab_22482	1.281	2.075E-5	4.891E-5	4.81	0.04975	4.278	0.05603	5.869	0.008779	4.857	0.03721	5.006	0.06145
Nebularine	metab_49719	1.281	0.001518	0.001582	4.726	0.02329	4.712	0.02133	4.738	0.05013	4.757	0.03827	5.767	0.02066
Cytidine-3'-Monophosphate	metab_49948	1.281	1.325E-5	4.891E-5	4.926	0.02368	5.442	0.01103	6.363	0.05417	3.432	0.1897	5.793	0.04582
5Z-7-Oxozeaenol	metab_13575	1.280	2.455E-5	4.891E-5	5.669	0.01444	3.354	0.1526	2.701	0.002693	3.437	0.06165	2.715	0.003255
Vitamin P	metab_38078	1.279	0.0008307	0.0008799	5.395	0.04578	3.499	0.8539	2.306	0.5081	2.702	0.931	2.261	0.4973
27-O-Demethylrifabutin	metab_48990	1.279	1.325E-5	4.891E-5	3.424	0.3095	4.983	0.01447	5.154	0.03751	6.023	0.01581	4.299	0.08032
Pa(6 Keto-Pgf1Alpha/I-13:0)	metab_39146	1.279	1.807E-5	4.891E-5	2.752	0.3913	5.293	0.02089	5.104	0.01151	3.258	0.4191	4.621	0.03104
Scd1 Inhibitor	metab_44344	1.279	1.518E-5	4.891E-5	3.696	0.1345	3.244	0.2591	4.923	0.009355	5.583	0.005818	4.784	0.008974
Val-Tyr-Lys	metab_18800	1.278	1.9E-5	4.891E-5	4.821	0.01939	4.894	0.03481	3.778	0.07468	5.997	0.005815	3.55	0.2127
Gallic Acid	metab_49213	1.278	0.000284	0.0003143	4.551	0.1991	3.725	0.7738	2.67	0.6544	2.808	0.9156	1.988	0.09983
Pf-3845	metab_36377	1.277	2.359E-5	4.891E-5	3.232	0.003274	3.308	0.1175	5.183	0.01773	3.827	0.0891	4.357	0.0127
N-Docosahexaenoyl Glutamic Acid	metab_34945	1.277	2.359E-5	4.891E-5	3.376	0.003274	3.518	0.1687	5.413	0.01097	5.384	0.01333	4.998	0.02364
Gln-Glu-Gln	metab_49707	1.276	2.591E-5	4.929E-5	2.743	0.00327	4.675	0.02223	5.774	0.1572	5.29	0.1791	5.544	0.1986
Jasmine Lactone	metab_12105	1.275	3.102E-5	5.388E-5	4.475	0.014	2.161	0.001846	2.237	0.002683	2.25	0.02278	2.26	0.02375
N-(Furan-2-Ylmethyl)-1-[(4-Methoxyphenyl)Methyl]Benzotriazole-5-Carboxamide	metab_13576	1.275	5.574E-5	7.755E-5	5.265	0.01785	2.958	0.001857	3.04	0.01088	3.04	0.006599	3.048	0.003259
Fraxinellone	metab_17372	1.275	7.204E-5	9.49E-5	4.715	0.02679	2.399	0.001852	2.512	0.08787	2.526	0.06943	2.489	0.003251
S-Allyl-L-Cysteine	metab_29013	1.274	1.325E-5	4.891E-5	6.533	0.00387	6.223	0.002304	4.922	0.0216	6.65	0.005404	6.031	0.003925
2-Hydroxy-3-Methyl-4H-Pyran-4-One O-(6E-Cinnamoyl-B-D-Glucoside)	metab_47719	1.274	1.325E-5	4.891E-5	2.562	0.003266	4.924	0.009863	5.59	0.01183	4.18	0.07309	5.685	0.007014
Glycinoeclepin C	metab_9882	1.274	1.421E-5	4.891E-5	4.595	0.08016	5.187	0.02484	5.451	0.02878	5.391	0.01699	4.276	0.1388
Linalool Oxide D 3-[Apiosyl-(1->6)-Glucoside]	metab_38460	1.274	1.99E-5	4.891E-5	5.853	0.004395	3.61	0.1532	3.332	0.3296	3.909	0.03299	4.105	0.04424
Pro-Asp-Ser	metab_30480	1.274	1.325E-5	4.891E-5	5.079	0.03234	4.785	0.03077	5.775	0.01265	4.052	0.2575	4.878	0.0214
Cyanidin 3-O-(2-Xylosyl-6'-Glucosyl-Galactoside)	metab_21979	1.274	2.402E-5	4.891E-5	5.809	0.02145	3.504	0.01589	3.574	0.002698	3.579	0.006603	3.588	0.003261
Forsythiaside	metab_38056	1.273	1.325E-5	4.891E-5	6.004	0.006444	3.79	0.04601	4.594	0.01587	4.899	0.01134	4.507	0.01599
Gly-Glu	metab_50414	1.272	2.515E-5	4.891E-5	3.581	0.1609	3.594	0.1603	5.307	0.01623	5.986	0.008237	5.021	0.03117
Gpcho(2:0/7:0)	metab_24207	1.272	1.325E-5	4.891E-5	3.531	0.3073	5.821	0.01204	6.75	0.008738	5.588	0.01618	6.476	0.008311
6''-Acetylliquiritin	metab_10832	1.271	2.402E-5	4.891E-5	5.25	0.02011	2.952	0.001857	3.029	0.002696	3.034	0.006599	3.042	0.003259
Ala-His-Tyr	metab_49630	1.271	1.518E-5	4.891E-5	4.722	0.0324	2.564	0.00117	3.139	0.3026	6.01	0.0813	2.738	0.1459
Cilligen	metab_43923	1.270	5.41E-5	7.584E-5	5.028	0.02298	2.632	0.00117	2.781	0.00676	2.975	0.2232	2.821	0.1207
3-Carboxymedetomidine	metab_36849	1.270	1.325E-5	4.891E-5	3.809	0.03585	4.708	0.00991	4.467	0.006595	5.16	0.006527	5.357	0.003631
Cer(15:0_2O/7:0)	metab_41619	1.269	2.411E-5	4.891E-5	2.912	0.09975	4.128	0.06822	3.55	0.2469	3.67	0.1308	4.663	0.02404

Basellasaponin D	metab_38093	1.269	1.325E-5	4.891E-5	2.758	0.1143	4.999	0.02849	5.814	0.01452	3.318	0.1775	5.612	0.01747
Asteltoxin	metab_2277	1.269	1.518E-5	4.891E-5	4.011	0.1002	3.499	0.237	5.546	0.01976	5.596	0.02518	4.673	0.05038
Arg Val Phe Asp	metab_6313	1.268	1.325E-5	4.891E-5	5.708	0.007762	5.024	0.02168	5.421	0.02242	4.927	0.02605	6.238	0.008558
Purpureaside C	metab_45191	1.268	1.518E-5	4.891E-5	6.012	0.007302	4.288	0.0739	4.668	0.03103	3.702	0.1341	4.737	0.0347
Adenylosuccinic Acid	metab_32651	1.268	0.0001655	0.0001904	2.579	0.1618	3.371	0.1766	4.096	0.6093	2.797	0.2554	2.761	0.3117
Guanidylic Acid (Guanosine Monophosphate)	metab_28908	1.268	1.325E-5	4.891E-5	4.617	0.03924	5.498	0.01002	5.874	0.005596	3.317	0.09215	5.068	0.02331
Arg Pro Gly	metab_28210	1.268	3.757E-5	5.997E-5	3.422	0.4436	3.818	0.1145	4.711	0.08209	5.76	0.215	3.187	0.192
Sec-O-Glucosylhamaudol	metab_49701	1.267	2.359E-5	4.891E-5	3.831	0.003275	3.891	0.09409	5.732	0.01062	4.378	0.1002	5.046	0.02595
1-O-[(2Alpha,3Beta)-2,3-Dihydroxy-28-Oxoolean-12-En-28-Yl]-Beta-D-Glucopyranose	metab_41902	1.267	5.314E-5	7.474E-5	5.319	0.01334	3.037	0.08216	3.209	0.1333	3.214	0.003719	3.119	0.0102
Arg-Gly-Ile	metab_28248	1.267	1.99E-5	4.891E-5	2.783	0.2134	3.559	0.2732	4.759	0.308	6.052	0.02148	4.466	0.4494
Choldienic Acid	metab_14417	1.266	0.0006107	0.0006541	5.378	0.239	4.908	0.8061	3.473	0.486	4	0.8511	3.048	0.2147
Phenylalanyl-Prolyl-Arginine	metab_42987	1.266	1.325E-5	4.891E-5	2.767	0.8379	4.019	0.05924	5.671	0.006304	5.798	0.008374	4.88	0.018
Hericenone B	metab_44936	1.265	1.325E-5	4.891E-5	1.803	0.996	3.254	0.163	4.967	0.01879	4.608	0.04235	4.144	0.04307
Pro-Asn-Ser	metab_22079	1.263	1.421E-5	4.891E-5	4.403	0.02898	4.139	0.0805	3.312	0.101	5.937	0.008485	3.973	0.0538
Homononactic Acid	metab_16691	1.263	0.0001253	0.0001501	5.45	0.007314	2.968	0.4506	2.804	0.1219	5.386	0.01852	3.105	0.4139
Geldanamycin	metab_7467	1.262	1.325E-5	4.891E-5	2.527	0.6163	4.185	0.05159	5.569	0.01093	5.254	0.03296	4.561	0.1176
Phe Asp Glu	metab_4706	1.262	1.837E-5	4.891E-5	5.44	0.02216	3.545	0.1663	4.764	0.1017	5.38	0.02447	4.617	0.08342
Butanal	metab_2291	1.262	1.325E-5	4.891E-5	5.906	0.004018	5.7	0.002941	4.15	0.01815	5.851	0.008949	5.129	0.00614
Asn-Gly-Ile	metab_34369	1.262	1.325E-5	4.891E-5	3.241	0.07995	2.537	0.02454	4.289	0.03093	5.461	0.006283	3.822	0.02571
Osmanthuside H	metab_35176	1.262	4.83E-5	6.945E-5	5.462	0.004859	3.594	0.0455	3.589	0.07753	3.189	0.1604	3.201	0.261
Gdp-Glucose	metab_50111	1.261	1.325E-5	4.891E-5	4.966	0.05486	4.713	0.07709	3.194	0.006767	3.256	0.003719	3.161	0.0102
L-Alanyl-L-Valine	metab_33949	1.261	1.325E-5	4.891E-5	2.695	0.003269	2.618	0.00117	4.424	0.03415	5.073	0.01633	2.999	0.1993
Ethyl L-Tryptophanate	metab_10721	1.260	1.325E-5	4.891E-5	5.075	0.01337	4.581	0.03698	3.58	0.06115	6.11	0.006002	3.302	0.108
15-Hydroxynorandrostene-3,17-Dione Glucuronide	metab_36537	1.260	1.9E-5	4.891E-5	2.999	0.132	5.308	0.01474	5.859	0.01644	3.241	0.2603	5.563	0.01253
Blood Group B Type 1 Tetrasaccharide	metab_34722	1.259	0.000118	0.0001428	4.512	0.06711	5.675	0.01128	4.539	0.07739	4.917	0.06326	4.578	0.07355
N-Allyl-N'-{4-[3,5-Bis(Trifluoromethyl)Phenoxy]Phenyl}Thiourea	metab_48928	1.258	2.724E-5	5.055E-5	4.522	0.0254	4.712	0.02016	4.76	0.064	4.549	0.09539	3.788	0.04908
Methyl 13-Hydroxyoctadeca-9,11-Dienoate	metab_16536	1.258	1.325E-5	4.891E-5	5.386	0.01166	2.73	0.001856	2.807	0.002694	4.795	0.02726	2.82	0.003257
Decanal	metab_42630	1.257	1.518E-5	4.891E-5	4.469	0.02487	3.574	0.1645	5.033	0.0139	4.415	0.03041	4.624	0.006688
6-Beta-Hydroxymedroxyprogesterone	metab_35584	1.257	1.325E-5	4.891E-5	4.278	0.01439	3.58	0.1262	5.416	0.009323	5.093	0.01661	4.48	0.0224
Glu-Gly-Leu	metab_45626	1.257	1.9E-5	4.891E-5	2.47	0.003264	2.439	0.1121	4.269	0.03224	4.07	0.03514	3.905	0.03837
Methyl (10E,12Z)-9-Oxooctadeca-10,12-Dienoate	metab_16385	1.257	1.325E-5	4.891E-5	5.786	0.01438	3.203	0.001858	3.28	0.002697	5.562	0.01562	3.294	0.00326
Piperanine	metab_17984	1.257	2.49E-5	4.891E-5	3.634	0.2527	4.932	0.01625	4.776	0.01931	4.784	0.01408	5.738	0.003908
N2-Succinyl-L-Ornithine	metab_49530	1.256	4.604E-5	6.719E-5	2.696	0.1635	2.439	0.3133	4.338	0.3392	5.179	0.1096	3.777	0.5665
Semagacestat	metab_39313	1.255	1.421E-5	4.891E-5	3.327	0.1845	3.971	0.07189	3.635	0.1426	5.221	0.01586	4.335	0.03167
Sucrose 1',4'-(4,4'-Dihydroxy-3,3'-Dimethoxy-B-Truxinate)	metab_38521	1.254	0.0006337	0.0006777	5.191	0.01227	2.912	0.1666	2.993	0.006764	3.055	0.003718	3.159	0.2527

Succinic Anhydride	metab_32091	1.254	1.99E-5	4.891E-5	5.393	0.009545	5.776	0.00741	4.974	0.01573	2.395	0.003706	5.372	0.01925
5-Methylcytidine	metab_50625	1.254	2.298E-5	4.891E-5	4.472	0.04361	4.034	0.05509	5.181	0.006066	4.065	0.09912	4.266	0.05221
Gln-Ile-Ser	metab_2899	1.254	0.0007165	0.0007626	1.682	0.5641	2.785	0.9087	3.042	0.5413	5.079	0.1886	2.25	1.045
Leukotriene E4	metab_10578	1.254	4.531E-5	6.668E-5	3.688	0.2014	3.632	0.3118	5.057	0.02885	2.978	0.4356	4.294	0.08597
(8Ar)-2-Methyl-7-(2-Pyrazinylcarbonyl)Hexahydroimidazo[1,5-A]Pyrazin-3(2H)-One	metab_1245	1.253	0.0001112	0.000136	4.752	0.05232	4.762	0.05584	6.143	0.005824	4.798	0.09883	5.082	0.01893
Glu-Tyr-Phe	metab_38576	1.253	3.353E-5	5.632E-5	2.227	0.02519	2.729	0.5028	4.552	0.01274	3.22	0.4324	4.221	0.03364
Tetrahydrodipicolinate	metab_46600	1.253	2.359E-5	4.891E-5	3.033	0.1309	2.915	0.2099	2.147	0.1314	4.901	0.008497	2.061	0.01012
Roxadustat	metab_49171	1.253	2.298E-5	4.891E-5	3.21	0.1175	3.324	0.2057	5.065	0.02455	5.464	0.01046	4.721	0.03902
Tubacin	metab_48994	1.253	1.9E-5	4.891E-5	2.304	0.7022	4.642	0.03674	5.465	0.04551	4.568	0.199	5.063	0.0659
Ginsenoside Rh7	metab_41740	1.252	3.69E-5	5.927E-5	5.918	0.06791	3.565	0.2666	3.522	0.3	4.208	0.08691	3.136	0.05684
Licoricone	metab_33519	1.251	2.535E-5	4.891E-5	3.188	0.248	3.901	0.02058	5.732	0.02161	5.199	0.01882	5.199	0.01511
Neuromedin B (4-10)	metab_20192	1.251	4.085E-5	6.262E-5	6.13	0.01141	4.145	0.4809	4.515	0.1748	4.753	0.1606	5.05	0.08539
Dacinostat	metab_13619	1.250	1.994E-5	4.891E-5	4.507	0.01556	2.537	0.7308	3.75	0.07507	5.894	0.01403	3.465	0.2457
S-Methoprene	metab_14854	1.250	0.0001542	0.0001792	6.253	0.02459	3.603	0.3558	3.675	0.2514	5.791	0.0166	3.685	0.3192
Hexapeptide Analogue	metab_46510	1.250	2.515E-5	4.891E-5	2.507	0.1764	4.964	0.02408	4.584	0.03972	2.505	0.1199	4.417	0.05268
Atracurium	metab_43087	1.250	1.807E-5	4.891E-5	5.642	0.01154	4.191	0.08386	3.985	0.268	4.474	0.07032	5.01	0.02954
4-Acetamidobutanoic Acid	metab_47035	1.250	2.974E-5	5.28E-5	2.321	0.03236	2.226	0.001166	2.373	0.006742	5.097	0.005687	2.34	0.01016
Corchoionoside B	metab_45484	1.249	1.325E-5	4.891E-5	5.81	0.005938	6.023	0.008013	5.686	0.01299	4.687	0.05805	4.939	0.05333
Val-Lys	metab_31880	1.249	2.359E-5	4.891E-5	3.573	0.003275	3.587	0.1485	4.776	0.04751	5.552	0.04336	4.999	0.06556
Lucidenic Acid K	metab_8047	1.249	1.9E-5	4.891E-5	4.964	0.01862	4.538	0.1059	5.665	0.01678	5.277	0.02501	4.418	0.09741
Milbemycin Alpha9	metab_16585	1.248	1.616E-5	4.891E-5	4.898	0.001284	4.887	0.001859	6.124	0.008796	4.969	0.006605	5.028	0.03685
Semilepidinoside B	metab_28285	1.247	1.325E-5	4.891E-5	2.965	0.3014	5.315	0.01796	5.932	0.04597	4.976	0.03138	5.408	0.008692
2-Aminooctanedioic Acid	metab_8350	1.247	1.325E-5	4.891E-5	3.768	0.1153	2.986	0.1418	4.772	0.05666	4.561	0.03128	4.143	0.1167
(1Xi,3Xi)-1,2,3,4-Tetrahydro-1-Methyl-Beta-Carboline-3-Carboxylic Acid	metab_45912	1.246	1.325E-5	4.891E-5	4.38	0.01813	5.166	0.01172	4.951	0.01282	5.626	0.006902	5.819	0.008002
Geneticin	metab_47421	1.246	1.325E-5	4.891E-5	3.819	0.07886	6.209	0.006666	6.287	0.005853	5.801	0.009959	6.139	0.01198
Pravastatin	metab_45634	1.246	1.325E-5	4.891E-5	4.316	0.04225	4.617	0.0111	6.449	0.008296	5.144	0.01976	5.638	0.007576
Sarcodon Scabrosus Depsipeptide	metab_37365	1.245	2.455E-5	4.891E-5	4.914	0.01739	4.599	0.09505	4.615	0.03802	6.213	0.006521	3.422	0.1583
Apadenoson	metab_45540	1.244	1.325E-5	4.891E-5	3.08	0.265	4.201	0.04083	5.964	0.005809	4.926	0.009954	5.21	0.01101
25-Acetylvulgaroside	metab_42054	1.244	0.00188	0.00195	5.378	0.2067	5.051	0.9497	3.162	1.266	4.678	0.6237	4.794	0.0175
Pteroside B	metab_35374	1.244	1.325E-5	4.891E-5	3.762	0.03808	3.189	0.1423	5.153	0.01089	5.618	0.005894	4.239	0.01992
Val-Gln	metab_32301	1.243	1.325E-5	4.891E-5	2.934	0.003272	2.858	0.001172	4.674	0.06378	5.452	0.0444	3.993	0.09267
2-(4-Methoxyphenyl)Ethyl 6-O-A-L-Arabinopyranosyl-B-D-Glucopyranoside	metab_20928	1.242	2.402E-5	4.891E-5	5.19	0.02653	2.997	0.001857	3.074	0.002696	3.079	0.006599	3.087	0.003259
Feruloylquinic Acid	metab_45388	1.241	2.359E-5	4.891E-5	5.611	0.005448	4.057	0.03636	4.017	0.0518	3.732	0.08901	3.405	0.1673
Gly Phe Phe	metab_18867	1.241	1.851E-5	4.891E-5	3.633	0.1138	2.875	0.04265	3.337	0.2485	4.875	0.0142	3.989	0.06486
Mgmg(18:3)	metab_40256	1.240	2.53E-5	4.891E-5	6.872	0.008758	4.957	0.03804	5.15	0.05657	4.622	0.1011	5.148	0.04585

Casomorphin	metab_43124	1.240	1.518E-5	4.891E-5	3.049	0.07849	4.894	0.03022	5.862	0.004402	3.345	0.1835	5.675	0.01705
Cortisol 21-Mesylate	metab_34629	1.240	1.325E-5	4.891E-5	2.192	0.5261	4.589	0.02353	4.865	0.02854	3.683	0.09782	5.147	0.01723
Arginylarginine	metab_50916	1.240	2.53E-5	4.891E-5	2.707	0.1768	2.858	0.4183	4.463	0.03226	5.276	0.01029	4.141	0.03339
His Tyr Ile Asp	metab_44927	1.240	2.49E-5	4.891E-5	2.279	0.007399	3.934	0.0921	4.931	0.01967	2.775	0.4226	3.96	0.1215
Nopalinic Acid	metab_36404	1.240	1.325E-5	4.891E-5	3.943	0.04469	4.821	0.007521	4.572	0.01521	5.145	0.009356	5.447	0.01357
Gpcho(7:0/2:0)	metab_23766	1.239	1.325E-5	4.891E-5	3.486	0.1387	5.594	0.009885	6.627	0.006473	4.863	0.0498	6.167	0.006306
Tricin 7-[Sinapoyl-(->2)-Glucuronyl-(1->2)-Glucuronide]	metab_50067	1.239	0.0003694	0.0004039	4.28	0.102	5.847	0.01744	4.247	0.2992	3.833	0.003721	4.731	0.4947
Dimethyl C7	metab_37193	1.238	2.586E-5	4.929E-5	5.148	0.02965	4.978	0.05908	5.234	0.02444	2.689	0.3022	5.013	0.04314
Hexaconazole	metab_29097	1.237	2.23E-5	4.891E-5	5.052	0.01394	5.035	0.02024	5.601	0.01427	3.299	0.1388	4.965	0.02852
Olanzapine	metab_48507	1.237	1.325E-5	4.891E-5	2.935	0.003272	5.083	0.01932	5.743	0.01126	4.265	0.08267	4.94	0.03351
Gly-Ile-Asp	metab_33165	1.237	1.9E-5	4.891E-5	2.296	0.221	3.328	0.1754	4.275	0.06144	4.926	0.1299	4.371	0.1494
Gly-Met-Ser	metab_49429	1.237	2.23E-5	4.891E-5	2.402	0.1507	2.202	0.001166	3.818	0.07289	4.973	0.04771	2.495	0.2193
4-Methoxybenzaldehyde N-[6-(Tert-Butyl)Thieno[3,2-D]Pyrimidin-4-Yl]Hydrazone	metab_49461	1.237	3.497E-5	5.736E-5	2.523	0.2157	4.266	0.01645	5.39	0.2499	5.172	0.05487	4.942	0.2199
Ser-Gly-Tyr	metab_49032	1.236	3.757E-5	5.997E-5	2.119	0.1818	4.197	0.02959	4.687	0.02639	4.6	0.1686	4.514	0.03362
Pro Gln Ala	metab_28976	1.236	2.075E-5	4.891E-5	2.522	0.5718	4.452	0.05588	5.21	0.01593	5.108	0.01067	4.394	0.05461
Phenylalanylhydroxyproline	metab_37179	1.236	1.325E-5	4.891E-5	1.881	0.003232	1.82	0.02506	1.973	0.0541	4.623	0.01291	1.919	0.01008
Met-Glu-His-Phe-Arg-Trp-Gly	metab_46049	1.236	1.325E-5	4.891E-5	5.443	0.01375	6.485	0.007254	6.204	0.007001	4.23	0.08991	5.557	0.01681
1B,3A,7A-Trihydroxy-5B-Cholanoic Acid	metab_40047	1.235	0.001236	0.001295	5.429	0.2435	4.86	1.093	3.938	0.507	4.366	0.8763	3.082	0.3824
Methyl 2-(3-Hydroxy-6-Oxoxanthen-9-Yl)Benzoate	metab_1678	1.235	2.49E-5	4.891E-5	4.766	0.0524	4.746	0.07831	5.636	0.02939	3.199	0.03093	5.151	0.04919
Glu Val Arg	metab_2846	1.234	0.0002693	0.000299	3.078	0.3036	5.481	0.01255	5.359	0.4706	5.042	0.2245	5.476	0.4237
Flunisolide	metab_12568	1.234	0.00584	0.005949	1.29	1.555	1.665	1.786	1.751	1.398	5.116	0.02605	1.562	1.673
Trp-Gln-Leu	metab_17918	1.234	2.55E-5	4.919E-5	4.071	0.09935	3.711	0.307	2.44	0.2704	4.993	0.0156	2.667	0.5028
Gamma-Glutamylproline	metab_33754	1.234	5.53E-5	7.716E-5	3.352	0.07771	3.36	0.1654	2.494	0.006749	5.075	0.0185	2.596	0.2401
2',3'-Isopropylideneadenosine	metab_2794	1.234	0.0007898	0.0008377	2.289	0.5126	3.167	0.4434	3.01	0.6901	5.196	0.07214	2.439	0.8748
Docetaxel	metab_44410	1.233	1.712E-5	4.891E-5	3.684	0.2591	5.03	0.02212	5.367	0.01603	3.332	0.2429	4.442	0.1167
Dimerum Acid	metab_37038	1.233	1.325E-5	4.891E-5	5.055	0.02281	4.154	0.0422	5.97	0.01108	6.647	0.007849	5.331	0.01154
Ile Phe Thr Asp Gln	metab_19762	1.233	1.518E-5	4.891E-5	5.314	0.01197	5.083	0.02876	5.235	0.02137	5.134	0.01492	4.205	0.1698
3',5'-Cyclic Gmp	metab_32834	1.233	1.807E-5	4.891E-5	5.281	0.007409	5.174	0.005261	4.885	0.3057	2.779	0.2642	4.492	0.4371
Withaferin A	metab_19476	1.233	1.712E-5	4.891E-5	2.679	0.2289	4.64	0.07971	5.48	0.02857	2.9	0.2769	5.131	0.0181
Ser Asn Thr	metab_22384	1.233	2.262E-5	4.891E-5	3.782	0.1804	4.256	0.06866	3.557	0.2403	2.965	0.1334	4.991	0.01981
3-Hydroxy-5-[3,4,5-Trihydroxy-6-(Hydroxymethyl)Oxan-2-Yl]Oxydecanoic Acid	metab_35705	1.233	2.927E-5	5.235E-5	5.27	0.01992	2.779	0.1517	3.346	0.293	4.354	0.03015	2.995	0.2052
His Thr Lys Lys	metab_4659	1.233	2.681E-5	5.035E-5	4.386	0.08084	5.26	0.01452	5.33	0.05288	5.832	0.01233	4.288	0.11
Kukoamine D	metab_8018	1.232	1.421E-5	4.891E-5	3.917	0.09974	5.497	0.01614	4.862	0.04652	5.63	0.01445	4.95	0.03692
Goshonoside F7	metab_38317	1.232	3.455E-5	5.698E-5	5.2	0.01432	3.774	0.272	4.075	0.0467	2.989	0.166	3.723	0.1412
Galactosylpyridinoline	metab_25675	1.231	2.49E-5	4.891E-5	3.67	0.1837	5.528	0.02064	5.544	0.02895	6.032	0.02021	5.141	0.06244

Ms 3	metab_3371	1.231	1.712E-5	4.891E-5	3.638	0.273	4.119	0.3042	5.963	0.02426	5.07	0.07701	5.432	0.01938		
6,8-Bis(Sulfanyl)Octanal	metab_50213	1.231	1.325E-5	4.891E-5	4.794	0.03426	6.694	0.005308	5.252	0.05468	4.041	0.4558	6.191	0.02874		
Neocarthamin	metab_50647	1.230	2.23E-5	4.891E-5	3.455	0.08356	4.603	0.04052	5.158	0.02653	4.069	0.08523	4.04	0.0612		
Asn-Gln-Lys	metab_31645	1.229	2.411E-5	4.891E-5	3.325	0.1291	4.217	0.01018	4.382	0.05785	5.219	0.03362	3.298	0.3032		
Madlongiside D	metab_22818	1.228	2.134E-5	4.891E-5	5.505	0.054	5.383	0.04748	5.806	0.0259	3.149	0.4677	5.843	0.0289		
Pro-Glu	metab_29051	1.228	1.99E-5	4.891E-5	2.88	0.1846	2.748	0.2069	4.552	0.05462	5.455	0.01157	3.641	0.1338		
Almorexant	metab_17619	1.228	0.0007656	0.000813	4.601	0.06372	3.18	0.753	4.378	0.1451	4.494	0.1733	4.512	0.06591		
Epothilone B	metab_43261	1.228	1.325E-5	4.891E-5	4.942	0.01013	4.243	0.03495	3.448	0.2464	5.108	0.02688	4.533	0.02227		
6-O-(7-Methyloctanoyl)-Alpha-D-Glucopyranosyl Alpha-D-Glucopyranoside	metab_35958	1.227	3.241E-5	5.532E-5	5.355	0.009445	3.04	0.3414	3.234	0.2863	3.981	0.08632	3.627	0.1696		
Ergovaline	metab_44213	1.227	1.325E-5	4.891E-5	3.086	0.4806	4.511	0.03124	5.921	0.01136	5.216	0.01795	5.623	0.009256		
2-[[2-[[(2S)-2-Amino-3-Methylbutanoyl]Amino]Acetyl]Amino]Pentanedioic Acid	metab_49556	1.227	3.102E-5	5.388E-5	2.445	0.003264	2.393	0.06175	3.929	0.07111	5.001	0.4631	3.629	0.2653		
Zolpidem	metab_10708	1.226	2.535E-5	4.891E-5	4.135	0.1135	4.139	0.0682	5.096	0.01948	2.675	0.006591	4.666	0.02377		
2-Oxoglutaric Acid	metab_50008	1.226	1.807E-5	4.891E-5	5.398	0.004324	5.727	0.009001	5.022	0.02228	2.529	0.00371	5.384	0.01132		
Midazolam	metab_49732	1.226	1.325E-5	4.891E-5	5.77	0.008034	5.993	0.0109	6.574	0.01826	5.627	0.01532	5.559	0.0759		
1-(3-Trifluoromethylphenyl)Piperazine	metab_21833	1.226	1.325E-5	4.891E-5	4.633	0.03043	5.478	0.008916	5.24	0.0099	5.911	0.005527	6.062	0.005224		
Isophorone Diisocyanate	metab_43642	1.225	4.478E-5	6.629E-5	3.553	0.08152	3.658	0.06895	3.721	0.09823	3.989	0.0512	4.606	0.00991		
Lys-Phe-Asp	metab_49234	1.224	4.424E-5	6.587E-5	2.429	0.006059	2.685	0.3559	3.34	0.3657	4.829	0.02362	3.728	0.4575		
Schidigerasaponin E1	metab_21504	1.223	3.215E-5	5.504E-5	5.428	0.01534	4.473	0.1075	4.53	0.2173	3.8	0.006604	5.462	0.02433		
Fraxin	metab_2892	1.223	3.69E-5	5.927E-5	2.937	0.001283	5.062	0.01358	3.762	0.41	3.008	0.006598	4.318	0.6413		
Morphiceptin	metab_12166	1.222	1.325E-5	4.891E-5	2.957	0.8415	5.151	0.03949	5.032	0.02691	4.803	0.04522	4.508	0.03641		
Gly-Gly-Ile	metab_10706	1.222	1.616E-5	4.891E-5	2.696	0.1053	3.329	0.1177	4.667	0.00863	4.618	0.01373	4.711	0.02368		
Crenolanib	metab_20318	1.221	4.469E-5	6.621E-5	3.676	0.2913	3.987	0.1659	5.5	0.01445	4.096	0.07706	4.413	0.06475		
Secologanate	metab_2836	1.221	2.113E-5	4.891E-5	3.584	0.2536	3.123	0.08738	5.027	0.03806	5.323	0.04217	3.9	0.1846		
4-Hydroxyandrostenedione Glucuronide	metab_8812	1.221	6.133E-5	8.356E-5	3.084	1.245	3.693	0.2462	4.812	0.02208	5.456	0.00659	3.197	0.3328		
Chrysoeriol 7-O-(6"-Malonyl-Apiosyl-Glucoside)	metab_33371	1.221	1.325E-5	4.891E-5	2.929	0.003272	4.288	0.06779	3	0.006765	3.063	0.003718	3.851	0.15		
Glu-Asn-Leu	metab_32582	1.221	1.325E-5	4.891E-5	2.447	0.1011	4.087	0.07044	5.166	0.04518	4.945	0.03503	4.276	0.02946		
Arg-Gly	metab_2796	1.221	2.354E-5	4.891E-5	3.74	0.1986	2.918	0.338	4.444	0.3319	5.689	0.281	4.261	0.4265		
N-Acetyllactosamine	metab_49111	1.221	1.325E-5	4.891E-5	2.383	0.003262	4.574	0.02924	5.263	0.01973	4.339	0.04581	4.748	0.01887		
Digitoxigenin Bisdigitoxide	metab_20569	1.220	4.504E-5	6.637E-5	5.712	0.01725	3.78	0.2464	3.927	0.04159	4.541	0.05049	4.55	0.1172		
Tyr Gly Glu	metab_28279	1.220	1.518E-5	4.891E-5	2.413	0.001279	2.402	0.001852	3.531	0.3138	5.451	0.02749	2.641	0.3094		
Polysorbate 20	metab_35860	1.219	2.53E-5	4.891E-5	4.643	0.02734	3.716	0.2003	3.703	0.1417	2.641	0.7349	4.737	0.03871		
Fruleuile	metab_8183	1.219	1.325E-5	4.891E-5	4.445	0.1529	4.669	0.07989	6.508	0.006388	5.656	0.009903	5.579	0.02211		
Quercetin-3'-Glucuronide	metab_32535	1.219	1.9E-5	4.891E-5	5.164	0.02329	5.377	0.02061	6.032	0.05588	3.55	0.1351	5.464	0.09443		
Asp Ser Gln Lys	metab_1767	1.219	2.359E-5	4.891E-5	4.958	0.03104	3.294	0.3012	4.655	0.0459	5.843	0.01081	3.499	0.2665		
Thr Ile Asp Phe Glu	metab_45131	1.219	1.325E-5	4.891E-5	5.17	0.019	3.717	0.09215	4.287	0.04306	4.925	0.02974	4.772	0.01854		

Urolithin A 8-Glucuronide	metab_50118	1.219	1.9E-5	4.891E-5	3.822	0.302	5.924	0.01806	4.694	0.09764	3.429	0.00372	5.27	0.06291		
1-(4-Trifluoromethylphenyl)Piperazine	metab_22648	1.219	1.325E-5	4.891E-5	5.07	0.03745	5.892	0.007709	5.676	0.007331	6.325	0.005659	6.492	0.006381		
Cis-4-Hydroxy-D-Proline	metab_49442	1.219	1.325E-5	4.891E-5	2.104	0.2658	3.151	0.08212	4.759	0.01973	4.406	0.04104	4.186	0.05017		
A-L-Fucopyranosyl-(1->2)-B-D-Galactopyranosyl-(1->2)-D-Xylose	metab_36880	1.218	1.325E-5	4.891E-5	2.694	0.003269	3.123	0.2789	4.873	0.006058	4.141	0.06771	4.465	0.02119		
Val-Asn	metab_50331	1.218	1.325E-5	4.891E-5	3.101	0.05674	4.143	0.04633	5.489	0.05116	5.888	0.09775	5.007	0.06042		
Eriojaposide B	metab_11959	1.218	3.121E-5	5.392E-5	4.664	0.02638	3.757	0.1427	2.754	0.5761	5.276	0.0153	3.594	0.199		
Alpha-Hydroxysalmeterol	metab_36993	1.217	1.518E-5	4.891E-5	2.868	0.2004	3.925	0.08675	5.618	0.006818	4.693	0.03387	4.777	0.03947		
25-Hydroxyvitamin D2-25-Glucuronide	metab_37513	1.217	2.702E-5	5.054E-5	5.293	0.01122	3.505	0.1041	3.42	0.1455	4.033	0.06097	4.215	0.08901		
Thr Phe Arg	metab_23397	1.217	1.325E-5	4.891E-5	3.494	0.1655	4.844	0.07597	5.619	0.02322	5.125	0.02781	4.439	0.1008		
Tyr Trp	metab_9354	1.217	1.325E-5	4.891E-5	2.781	0.2283	4.129	0.1205	5.145	0.01999	5.74	0.008861	4.449	0.04871		
Neoacrimarine A	metab_34242	1.217	3.32E-5	5.597E-5	6.23	0.006079	4.578	0.03106	4.721	0.04425	4.689	0.07815	5.361	0.01904		
Neoannonin B	metab_15141	1.216	1.712E-5	4.891E-5	5.972	0.09012	3.541	0.001859	3.625	0.01053	5.689	0.04668	3.705	0.1143		
Dihydrokaempferol	metab_43471	1.215	0.000116	0.0001406	4.51	0.05017	2.548	0.2191	2.498	0.08085	2.528	0.00371	3.042	0.154		
Ala-Arg-Ile	metab_33239	1.215	1.325E-5	4.891E-5	3.162	0.09001	3.546	0.1176	4.988	0.02865	5.53	0.06475	4.794	0.07481		
Pgp(Pgj2/I-14:0)	metab_46230	1.215	1.325E-5	4.891E-5	3.032	0.003272	4.959	0.03044	5.165	0.0305	3.237	0.1743	5.792	0.00619		
Tangeraxanthin	metab_35028	1.215	2.156E-5	4.891E-5	3.922	0.1086	3.868	0.05969	5.568	0.01679	4.465	0.06012	4.871	0.02433		
Phe-Thr	metab_49132	1.213	2.515E-5	4.891E-5	3.453	0.0771	3.448	0.07225	5.015	0.008487	5.856	0.007404	4.638	0.01897		
L-Beta-Homothreonine	metab_6963	1.213	1.325E-5	4.891E-5	3.419	0.07844	3.674	0.1051	2.642	0.02386	4.762	0.01949	2.991	0.1715		
Val-Pro-Pro	metab_47078	1.211	1.616E-5	4.891E-5	4.515	0.0384	3.486	0.1823	5.041	0.01516	6.219	0.004595	3.78	0.1742		
Decarbamoylsaxitoxin	metab_35883	1.211	3.625E-5	5.857E-5	3.743	0.122	4.68	0.02741	4.654	0.02626	5.274	0.01092	3.725	0.1263		
Antimycin A	metab_34389	1.210	2.53E-5	4.891E-5	2.407	0.489	4.703	0.04554	5.22	0.01294	3.938	0.1241	5.214	0.0201		
3-Carboxy-4-Methyl-5-Propyl-2-Furanpropanoic Acid	metab_37178	1.210	0.0002085	0.000235	5.359	0.006323	3.253	0.09925	3.334	0.07379	3.387	0.03943	3.455	0.07076		
Piperalol	metab_37688	1.209	1.325E-5	4.891E-5	2.492	0.003265	2.984	0.139	2.563	0.006753	4.793	0.008463	2.53	0.01018		
Ps(6 Keto-Pgf1Alpha/16:0)	metab_11980	1.209	1.325E-5	4.891E-5	5.579	0.02463	4.912	0.05674	3.768	0.09602	6.277	0.0163	4.297	0.08256		
Gly-Ala-Met	metab_2691	1.209	1.421E-5	4.891E-5	4.234	0.05261	2.581	0.1026	4.008	0.1107	4.994	0.2051	3.6	0.1411		
Deacetylchloronectrin	metab_2670	1.208	4.744E-5	6.872E-5	4.482	0.1518	4.473	0.04853	6.221	0.0362	5.302	0.1466	5.343	0.03985		
Ps(6 Keto-Pgf1Alpha/15:0)	metab_36229	1.208	1.325E-5	4.891E-5	3.132	0.7624	5.26	0.01281	6.182	0.01296	5.143	0.02538	5.764	0.01299		
Prostaglandin D1 Alcohol	metab_42005	1.208	2.298E-5	4.891E-5	4.799	0.02057	5.67	0.01278	4.644	0.02255	4.931	0.0118	5.664	0.01478		
Leu Ile Gln Asp	metab_35273	1.208	2.53E-5	4.891E-5	4.224	0.04134	4.431	0.02836	4.229	0.04524	4.759	0.02607	5.11	0.02275		
Gly Ile Phe	metab_12030	1.208	2.411E-5	4.891E-5	3.41	0.1869	3.289	0.2994	5.019	0.01824	5.582	0.008986	4.513	0.04175		
Sorbitan Palmitate	metab_41449	1.208	1.421E-5	4.891E-5	5.158	0.01143	5.752	0.01477	4.922	0.01228	5.184	0.006504	4.547	0.03651		
Gly Leu Phe	metab_38322	1.208	1.325E-5	4.891E-5	3.736	0.05341	3.029	0.2584	4.716	0.01208	5.304	0.00532	4.298	0.02229		
Arg-Ala-Ile	metab_49133	1.207	2.927E-5	5.235E-5	2.581	0.003267	2.515	0.02673	4.207	0.5923	5.16	0.4915	3.734	0.5355		
4'-Hydroxycilostazol, Trans-	metab_45559	1.207	1.325E-5	4.891E-5	3.718	0.1026	3.127	0.1672	4.712	0.01086	4.945	0.0122	4.469	0.01949		
De-O-Methylsimmondsin	metab_32609	1.207	3.215E-5	5.504E-5	2.728	0.003269	2.699	0.07758	4.236	0.4948	2.906	0.07015	4.134	0.4617		

Trp-Val	metab_8282	1.206	1.807E-5	4.891E-5	3.153	0.2302	4.335	0.04181	5.148	0.02007	6.129	0.006027	4.42	0.07583
Dihydroceramide C2	metab_14324	1.206	1.325E-5	4.891E-5	6.148	0.01331	5.764	0.02205	4.68	0.04204	5.213	0.01962	5.965	0.02116
Asn-Met	metab_49588	1.206	1.325E-5	4.891E-5	2.186	0.003254	2.11	0.001164	3.454	0.1501	4.839	0.0417	2.261	0.04868
Balagyptin	metab_20448	1.206	0.0001579	0.0001829	3.758	0.1924	4.209	0.3758	5.493	0.01222	3.703	0.1971	4.46	0.2289
Hexcer(19:2_2O/15:0_O)	metab_41456	1.205	9.417E-5	0.0001182	5.583	0.05022	3.284	0.2245	3.326	0.1094	3.915	0.1748	3.231	0.0102
Homovanillic Acid Sulfate	metab_48245	1.205	2.455E-5	4.891E-5	4.75	0.003171	4.249	0.02207	4.539	0.01453	3.717	0.00372	3.715	0.06015
Arg Gln Ser Lys	metab_9744	1.204	4.504E-5	6.637E-5	4.199	0.1194	3.141	0.1833	2.542	0.4102	5.501	0.01599	2.726	0.2562
Catechin Gallate	metab_37635	1.204	0.0006891	0.0007347	4.945	0.3584	4.474	0.9368	3.122	0.5924	3.599	0.938	2.704	0.01019
3-Benzyl-6-(1H-Indol-3-Ylmethyl)Piperazine-2,5-Dione	metab_17355	1.204	1.325E-5	4.891E-5	2.71	0.1952	3.869	0.1994	5.27	0.01846	4.752	0.01915	5.015	0.007202
Pro Met Tyr	metab_6912	1.203	1.325E-5	4.891E-5	2.939	0.7273	4.644	0.04596	5.538	0.01335	5.725	0.01067	5.068	0.03061
Sedoheptulose 7-Phosphate	metab_31834	1.203	3.047E-5	5.354E-5	3.1	0.3713	5.32	0.03226	4.763	0.03288	2.83	0.04567	4.696	0.04057
Asp-Gly-Ile	metab_287	1.203	7.94E-5	0.0001024	2.828	0.3014	3.345	0.7267	4.65	0.09578	3.773	0.3269	4.935	0.02382
Ala-Glu-Leu	metab_46208	1.202	1.325E-5	4.891E-5	2.488	0.003265	4.602	0.01174	5.169	0.007356	4.39	0.0456	5.092	0.008333
Ergosine	metab_22051	1.202	1.325E-5	4.891E-5	3.572	0.7224	5.021	0.0397	5.544	0.008109	4.748	0.09786	6.206	0.008392
L-2-Amino-4-Methylenepentanedioic Acid	metab_32563	1.202	1.325E-5	4.891E-5	2.321	0.00326	3.836	0.04964	4.988	0.01617	3.97	0.0485	4.92	0.004671
Pgp(Pgf1Alpha/I-13:0)	metab_44448	1.202	1.9E-5	4.891E-5	3.146	0.183	4.921	0.06035	5.807	0.009281	3.42	0.2822	5.625	0.0125
Cotinine Glucuronide	metab_37385	1.201	1.325E-5	4.891E-5	2.368	0.003261	2.291	0.001167	2.508	0.1407	5.007	0.0111	2.406	0.01017
Ala Thr Val Glu	metab_35362	1.201	1.421E-5	4.891E-5	5.662	0.007017	5.184	0.01081	5.218	0.006855	5.686	0.01023	4.154	0.07787
Sandoricin	metab_48250	1.200	1.325E-5	4.891E-5	2.444	0.6942	4.716	0.01567	5.399	0.02225	3.873	0.173	4.9	0.04349
Isofloxythepin	metab_33520	1.200	2.262E-5	4.891E-5	3.679	0.12	3.35	0.1444	5.095	0.02928	5.346	0.06784	3.846	0.1891
Ala-Gly-Met	metab_32775	1.199	2.455E-5	4.891E-5	4	0.04844	3.01	0.1718	4.514	0.06384	6.032	0.03056	3.971	0.06425
Manassantin A	metab_44440	1.199	1.325E-5	4.891E-5	4.447	0.06851	4.977	0.02981	4.893	0.05396	4.174	0.1118	6.021	0.008191
Trp-Glu-Ile	metab_45280	1.199	1.325E-5	4.891E-5	4.65	0.02312	3.833	0.1008	5.556	0.007443	6.075	0.0102	4.963	0.01972
Arginyl-Prolyl-Proline	metab_38172	1.199	1.325E-5	4.891E-5	2.243	0.09354	4.124	0.03906	5.099	0.01291	3.091	0.2857	4.806	0.01569
(3A,5B)-24-Oxo-24-[(2-Sulfoethyl)Amino]Cholan-3-Yl-B-D-Glucopyranosiduronic Acid	metab_34896	1.198	1.9E-5	4.891E-5	3.275	0.1297	5.241	0.01362	6.153	0.006613	5.261	0.0172	5.655	0.01606
Fructose-Lysine	metab_50801	1.198	1.518E-5	4.891E-5	5.1	0.02386	4.435	0.01568	5.143	0.0155	4.528	0.0189	4.275	0.03621
Arginine Ethyl Ester	metab_27675	1.197	1.518E-5	4.891E-5	4.404	0.04476	4.293	0.07605	3.342	0.002697	5.675	0.01416	3.355	0.00326
2-Hydroxycaprylic Acid	metab_39719	1.196	0.0002085	0.000235	5.412	0.0108	3.424	0.1494	3.53	0.062	3.408	0.142	3.641	0.05252
Aeglin	metab_2697	1.196	0.0007292	0.0007757	2.105	1.012	1.72	0.6842	2.141	0.9744	5.056	0.2897	1.995	0.6626
Phe-Thr-Ile	metab_43886	1.195	1.325E-5	4.891E-5	1.97	0.3657	3.132	0.1287	3.735	0.05914	4.352	0.02702	3.969	0.02814
Asp-Leu-Ser	metab_33149	1.195	1.325E-5	4.891E-5	2.955	0.1406	4.268	0.0199	4.702	0.01289	5.151	0.009579	5.021	0.04155
Hexosyl Lpe(16:0)	metab_41926	1.195	1.325E-5	4.891E-5	4.402	0.06688	5.697	0.01076	6.182	0.01251	3.373	0.2191	5.812	0.006511
Laninamivir	metab_45569	1.195	1.325E-5	4.891E-5	2.754	0.3352	3.459	0.0644	4.783	0.01782	5.162	0.01641	4.601	0.02002
Phe-Val	metab_12633	1.195	1.616E-5	4.891E-5	4.686	0.007867	3.102	0.1216	3.871	0.08965	4.332	0.03002	4.101	0.1156
Arg-Pro	metab_28941	1.195	1.325E-5	4.891E-5	3.142	0.139	4.36	0.0575	5.035	0.02233	5.847	0.01046	4.163	0.06875

Equilenin	metab_21911	1.194	2.49E-5	4.891E-5	3.127	0.1926	4.283	0.0365	3.907	0.1716	3.188	0.1871	5.065	0.01104
Ps(4:0/16:2)	metab_6106	1.194	2.23E-5	4.891E-5	3.341	0.7627	5.441	0.03309	5.881	0.039	5.695	0.02669	5.674	0.02483
Ile Pro Glu	metab_4968	1.194	2.156E-5	4.891E-5	3.098	0.6766	4.498	0.1305	5.772	0.02301	5.75	0.03438	5.199	0.04684
Sialyl Lewis X Antigen	metab_36904	1.193	1.325E-5	4.891E-5	3.086	0.003273	4.793	0.02963	5.476	0.01466	3.551	0.171	4.647	0.04333
Rutaretin 9-Rutinoside	metab_46239	1.192	2.44E-5	4.891E-5	3.998	0.08309	4.744	0.02914	3.735	0.1079	4.485	0.03699	3.641	0.1264
3-Dehydronobilin	metab_36902	1.192	1.712E-5	4.891E-5	4.759	0.01741	4.062	0.1514	4.933	0.02278	3.787	0.1789	5.177	0.01911
17-Aag	metab_37456	1.192	2.285E-5	4.891E-5	4.772	0.03333	3.396	0.3288	4.275	0.05064	4.073	0.1495	4.355	0.05045
Chitobiose	metab_36864	1.192	1.325E-5	4.891E-5	2.377	0.2943	3.732	0.08103	5.084	0.01287	4.477	0.02531	4.679	0.01813
Benzyloxycarbonyl-Val-Ala-Asp(Ome)-Fluoromethylketone	metab_37744	1.192	1.325E-5	4.891E-5	3.63	0.1766	4.24	0.01462	5.935	0.01189	5.752	0.006445	5.261	0.01151
N-Cyclopropyl-4-[[1-(3,4-Dimethylbenzoyl)Piperidin-4-Yl]Methoxy]Benzamide	metab_22092	1.191	1.325E-5	4.891E-5	4.968	0.03008	5.185	0.01622	6.881	0.00638	5.86	0.007182	6.031	0.005375
3-Hydroxybutyric Acid	metab_23226	1.190	1.325E-5	4.891E-5	4.818	0.009175	4.932	0.003969	3.741	0.07765	5.663	0.00549	4.124	0.02971
Panax Ginseng Tetrapeptide	metab_38770	1.190	2.411E-5	4.891E-5	4.495	0.01023	5.101	0.01293	3.932	0.1214	4.515	0.05926	4.848	0.04466
His-Asp-Arg	metab_7094	1.189	1.325E-5	4.891E-5	4.276	0.1235	4.542	0.1067	6.3	0.008238	5.656	0.01189	5.316	0.01158
Scorzonoside	metab_37398	1.189	1.851E-5	4.891E-5	4.006	0.06386	3.313	0.1584	4.195	0.07674	5.176	0.01821	2.791	0.3473
4-Coumaric Acid	metab_44363	1.189	1.325E-5	4.891E-5	6.119	0.002255	5.492	0.003037	5.432	0.005564	5.366	0.005131	4.568	0.01218
Kaempfetrin	metab_44000	1.189	0.0005265	0.0005672	5.231	0.5154	3.678	0.5168	3.693	0.7033	3.256	0.2909	3.042	0.0102
Mmda	metab_21416	1.189	2.455E-5	4.891E-5	4.082	0.05145	2.825	0.2874	2.563	0.1708	4.981	0.02371	3.206	0.1007
poststatin	metab_25137	1.189	1.9E-5	4.891E-5	3.978	0.2655	3.523	0.001859	5.011	0.04199	5.97	0.01115	3.689	0.08273
Lotaustralin	metab_1711	1.189	1.325E-5	4.891E-5	4.789	0.02264	4.881	0.01817	6.226	0.0116	5.121	0.02564	5.211	0.01347
Glu-Ile-Asp	metab_35650	1.188	1.325E-5	4.891E-5	3.941	0.1235	5.711	0.005394	6.343	0.006961	5.655	0.005412	6.556	0.007567
(+/-)-Threo-1-(P-Hydroxyphenyl)Propylene Glycol 4'-Glucoside	metab_38050	1.188	1.325E-5	4.891E-5	2.838	0.1385	4.761	0.01781	4.828	0.03686	3.5	0.09955	4.16	0.07953
Glycochenodeoxycholate-3-Sulfate	metab_34869	1.188	1.325E-5	4.891E-5	3.024	0.244	4.365	0.0306	5.795	0.01164	4.826	0.03396	4.95	0.03556
Asterinin D	metab_12919	1.187	2.455E-5	4.891E-5	5.105	0.05214	4.262	0.1843	3.41	0.2004	5.433	0.02879	4.337	0.08806
Ac2Pim1(18:2/14:0)	metab_37257	1.187	4.268E-5	6.436E-5	5.606	0.01167	4.252	0.08711	4.217	0.1369	4.082	0.003721	5.019	0.02578
Biotripyrrin-A	metab_48624	1.186	1.99E-5	4.891E-5	4.002	0.09097	4.958	0.00753	4.62	0.04853	5.907	0.007568	4.11	0.084
Glu Pro Leu	metab_5897	1.186	4.337E-5	6.498E-5	3.538	0.1961	4.123	0.238	5.128	0.0505	6.198	0.01405	3.96	0.3564
Car(14:3)	metab_16460	1.186	2.708E-5	5.054E-5	5.145	0.03212	5.07	0.02812	3.717	0.1134	5.164	0.01991	4.571	0.06285
Ser-Ala	metab_50420	1.186	1.712E-5	4.891E-5	3.862	0.1698	3.614	0.1137	5.064	0.03923	5.696	0.02571	4.911	0.0291
Pro Arg	metab_8905	1.186	1.9E-5	4.891E-5	4.836	0.02291	3.774	0.0256	4.687	0.02994	3.84	0.1019	4.36	0.05811
Protease-Activated Receptor-4	metab_36900	1.185	1.325E-5	4.891E-5	5.429	0.009996	4.663	0.02312	5.548	0.01118	4.732	0.03579	5.722	0.01232
Vignatic Acid A	metab_43260	1.184	1.325E-5	4.891E-5	4.815	0.02368	4.586	0.01722	3.762	0.06873	5.035	0.01268	4.735	0.02245
5-Hydroxyprimaquine	metab_36141	1.184	1.421E-5	4.891E-5	4.923	0.02559	3.97	0.05598	4.322	0.04332	2.647	0.4138	4.241	0.01208
Cdp-Dg(Pgd2/A-17:0)	metab_38543	1.183	2.23E-5	4.891E-5	4.405	0.08684	3.869	0.128	4.484	0.09979	5.243	0.02602	5.014	0.04115
N2-Fructopyranosylarginine	metab_30445	1.181	1.325E-5	4.891E-5	5.61	0.02299	5.343	0.0137	6.302	0.0105	5.258	0.02943	5.453	0.02431
Betanin	metab_33397	1.181	2.359E-5	4.891E-5	3.177	0.003273	4.572	0.0212	3.379	0.1392	3.311	0.003719	4.175	0.04097

Lobetyolin	metab_37660	1.181	1.325E-5	4.891E-5	3.925	0.1035	5.437	0.009097	5.148	0.0054	5.629	0.008516	4.749	0.01675
Leu-Asn	metab_49789	1.181	1.325E-5	4.891E-5	3.041	0.1689	3.99	0.04722	5.138	0.06225	5.597	0.03758	4.9	0.05108
Doxantrazole	metab_32765	1.180	1.958E-5	4.891E-5	4.102	0.03109	3.75	0.1147	3.483	0.2137	3.22	0.2389	2.706	0.01019
Sayaendoside	metab_37347	1.180	3.902E-5	6.072E-5	5.02	0.0133	2.76	0.09588	2.868	0.006762	3.564	0.2514	2.835	0.01019
Encaleret	metab_20117	1.180	1.325E-5	4.891E-5	4.071	0.2915	4.813	0.01914	6.455	0.006454	6.223	0.006253	5.713	0.008769
(3-Ethyl-3-Hydroxy-7-Azaspiro[3.5]Nonan-7-Yl)-Thiophen-2-Ylmethanone	metab_29358	1.180	2.939E-5	5.243E-5	4.929	0.02186	4.959	0.02149	6.274	0.008818	5.257	0.0196	5.271	0.0102
Methyl Helianthenoate F Glucoside	metab_23833	1.180	3.441E-5	5.698E-5	3.79	0.1644	3.285	0.2155	4.675	0.05774	5.406	0.01183	3.297	0.2274
Indole-3-Lactic Acid	metab_38261	1.179	1.325E-5	4.891E-5	5.049	0.007281	5.073	0.004942	3.7	0.07119	4.134	0.02345	4.913	0.00962
Trp-Thr	metab_35864	1.179	1.325E-5	4.891E-5	2.381	0.003262	2.756	0.2748	4.179	0.01847	4.841	0.02001	3.813	0.03599
Cinobufotalin	metab_36776	1.179	1.325E-5	4.891E-5	4.462	0.02723	4.887	0.01488	6.591	0.007042	5.663	0.006778	5.841	0.005915
Ligstroside-Aglycone	metab_42898	1.179	0.0001479	0.000173	4.844	0.0236	2.812	0.1221	2.244	0.2107	2.928	0.09538	2.802	0.2092
N-(1-Deoxy-1-Fructosyl)Valine	metab_29814	1.178	2.455E-5	4.891E-5	4.815	0.02983	4.908	0.0376	6.17	0.004741	5.149	0.03395	5.14	0.03001
Herculin	metab_2817	1.177	7.436E-5	9.684E-5	3.014	0.2843	2.68	0.001855	2.757	0.002694	5.396	0.5993	2.77	0.003256
Glu-Val-Leu	metab_44163	1.177	1.325E-5	4.891E-5	3.648	0.091	5.69	0.008731	6.426	0.009211	4.919	0.01119	6.193	0.007524
4A-Methylzymosterol-4-Carboxylic Acid	metab_40851	1.176	1.325E-5	4.891E-5	5.416	0.07297	3.068	0.001172	3.216	0.006767	4.678	0.03099	3.183	0.0102
Ethyl P-Coumarate	metab_39668	1.176	1.325E-5	4.891E-5	4.473	0.01634	3.697	0.03811	3.494	0.04115	4.557	0.02136	2.549	0.1125
Oripavine	metab_48015	1.176	1.99E-5	4.891E-5	3.143	0.8049	5.038	0.02091	4.437	0.04861	4.761	0.03768	4.473	0.03056
Gln Val Tyr Asp	metab_5933	1.176	2.113E-5	4.891E-5	5.877	0.01875	4.565	0.1938	4.904	0.07169	5.811	0.02129	3.74	0.7621
Alpha-Carissanol	metab_47240	1.175	5.988E-5	8.196E-5	3.213	0.1879	2.853	0.293	2.47	0.02493	5.147	0.008332	2.513	0.1275
Gln Asn Ala	metab_29632	1.175	4.337E-5	6.498E-5	3.284	0.0979	2.58	0.5523	4.257	0.08508	4.936	0.01685	2.94	0.5978
Ganoderenic Acid D	metab_11459	1.175	1.325E-5	4.891E-5	6.542	0.004767	4.864	0.04379	5.406	0.02115	6.154	0.007934	5.766	0.02075
Vignatic Acid B	metab_38716	1.175	1.325E-5	4.891E-5	5.28	0.01178	4.768	0.01922	3.995	0.06478	5.219	0.01403	5.017	0.01731
Gly-Val-His	metab_28747	1.173	2.53E-5	4.891E-5	4.705	0.1893	4.646	0.06031	6.27	0.1198	5.647	0.07398	5.231	0.1166
Arg-Lys	metab_50985	1.173	1.99E-5	4.891E-5	2.47	0.1908	2.89	0.387	4.11	0.06297	5.089	0.02422	3.85	0.0833
Pectolinarin	metab_1390	1.173	2.535E-5	4.891E-5	3.892	0.3454	6.169	0.01423	5.929	0.02556	3.886	0.3744	6.125	0.01056
Fahfa(18:2/4:0)	metab_41730	1.173	1.325E-5	4.891E-5	3.533	0.003274	3.456	0.001173	4.924	0.02566	3.796	0.09103	4.608	0.04065
6-(4-Methoxyphenyl)Pyrimidine-2,4-Diamine	metab_20374	1.173	2.53E-5	4.891E-5	5.11	0.01525	4.697	0.03216	3.758	0.1189	6.1	0.004224	3.735	0.1078
Ethyl Butylacetylaminopropionate	metab_21356	1.172	1.421E-5	4.891E-5	3.825	0.04827	3.097	0.2784	2.655	0.002693	5.422	0.01427	2.684	0.04092
Aspartyl-Valine	metab_49524	1.172	2.075E-5	4.891E-5	3.452	0.1109	3.356	0.08457	4.909	0.01676	5.623	0.01363	4.464	0.01849
H-Asp-Ala-His-Lys-Oh	metab_28297	1.172	0.0001048	0.0001293	4.784	0.05755	3.017	0.2625	3.182	0.4244	5.756	0.3012	2.738	0.09295
(8S)-8-Amino-7-Oxononanoyl-Coa	metab_50113	1.172	2.455E-5	4.891E-5	4.253	0.1222	5.271	0.03047	5.413	0.01751	2.611	0.003712	5.418	0.02916
1H,1H,7H-Dodecafluoroheptan-1-Ol	metab_32754	1.171	4.184E-5	6.344E-5	4.862	0.00912	4.361	0.04433	2.87	0.006762	3.386	0.3323	3.036	0.1975
Dihydrostreptomycin	metab_28993	1.171	1.325E-5	4.891E-5	4.5	0.001284	4.489	0.001859	5.608	0.02738	4.571	0.006605	4.646	0.05548
Epigallocatechin Gallate	metab_36541	1.170	0.0002365	0.0002645	4.772	0.2707	4.028	0.8054	2.601	0.07121	3.17	0.7307	2.539	0.01018
Kaempferol-3-O-Rutinoside	metab_38600	1.170	0.000331	0.0003637	5.041	0.2079	3.647	0.5977	3.137	0.5781	3.092	0.5484	2.647	0.01018

Fructosyl-Lysine	metab_348	1.169	1.325E-5	4.891E-5	5.739	0.01555	5.372	0.04288	5.793	0.02106	4.546	0.1274	5.059	0.04599
Histidinohydroxylysinonorleucine	metab_49157	1.169	2.774E-5	5.099E-5	2.566	0.1198	2.441	0.001169	2.914	0.4481	5.252	0.1106	2.556	0.01018
Dihydrovaltrate	metab_12822	1.168	1.421E-5	4.891E-5	4.922	0.03386	4.193	0.08605	2.867	0.002695	4.365	0.04503	2.936	0.1354
Ethyl 1-(Furan-2-Ylmethyl)-4-Hydroxy-5-Oxo-2H-Pyrrole-3-Carboxylate	metab_36186	1.167	1.325E-5	4.891E-5	3.914	0.04441	3.762	0.04487	4.878	0.01163	3.392	0.1747	4.147	0.02408
L-Acetylcarnitine	metab_19454	1.167	1.99E-5	4.891E-5	3.608	0.1063	3.499	0.07003	5.229	0.006053	5.07	0.01414	4.561	0.01089
Trans-Cinnamoyl-Beta-D-Glucoside	metab_36861	1.167	2.411E-5	4.891E-5	4.731	0.01796	3.963	0.03476	3.993	0.0433	4.234	0.03215	3.063	0.112
Ser-Val	metab_49675	1.167	1.712E-5	4.891E-5	3.23	0.2418	3.559	0.06696	4.969	0.01225	5.736	0.004077	4.512	0.005316
Arg-Gly-Asp-Ser	metab_35605	1.167	1.325E-5	4.891E-5	5.077	0.008862	5.102	0.01113	5.38	0.009075	4.906	0.02334	4.528	0.02677
4-(1,7-Dihydroxyoctyl)-2-Hydroxy-3-Methyl-2H-Furan-5-One	metab_39722	1.166	1.325E-5	4.891E-5	5.257	0.01211	4.473	0.03165	5.665	0.01264	5.057	0.01567	5.158	0.01493
Pyripyropene A	metab_47470	1.166	1.325E-5	4.891E-5	3.716	0.1556	5.565	0.006085	5.372	0.02383	3.208	0.1453	5.067	0.05007
(15A,20R)-Dihydroxypregn-4-En-3-One 20-[Glucosyl-(1->4)-6-Acetyl-Glucoside]	metab_16629	1.166	2.164E-5	4.891E-5	6.346	0.02074	5.424	0.1306	5.237	0.05255	3.854	0.3784	5.082	0.07262
Glu-Ala	metab_28751	1.166	3.428E-5	5.698E-5	3.354	0.4569	3.614	0.13	5.198	0.03012	5.134	0.05798	4.886	0.02576
Ps(6 Keto-Pgf1Alpha/18:1(11Z))	metab_48644	1.166	1.325E-5	4.891E-5	6.074	0.008781	5.089	0.02435	4.737	0.05299	5.729	0.0119	3.928	0.3023
Pi(Pgf1Alpha/20:1(11Z))	metab_36805	1.166	4.604E-5	6.719E-5	3.985	0.003275	4.164	0.1561	5.654	0.01327	4.222	0.09086	4.978	0.02561
(3Beta,17Alpha,23S)-17,23-Epoxy-3,28,29-Trihydroxy-27-Norlanost-8-En-24-One	metab_40809	1.166	1.325E-5	4.891E-5	5.116	0.03365	3.044	0.001172	3.193	0.006767	3.504	0.1731	3.159	0.0102
Periandrin I	metab_37990	1.165	2.156E-5	4.891E-5	3.605	0.127	4.582	0.06661	5.723	0.01412	3.378	0.3749	5.345	0.02555
Physagulin E	metab_38548	1.165	4.744E-5	6.872E-5	3.141	0.2257	5.215	0.02265	4.557	0.03094	3.118	0.1186	4.531	0.06122
Asp-Ser-Pro	metab_32571	1.165	5.443E-5	7.614E-5	3.03	0.2692	2.547	0.177	3.862	0.06952	4.826	0.04095	2.538	0.02102
1-Myristoyl-Sn-Glycerol 3-Phosphate	metab_41774	1.165	1.807E-5	4.891E-5	3.743	0.07982	5.095	0.02711	5.351	0.01532	3.449	0.2109	4.592	0.0108
Candidin	metab_47361	1.165	2.23E-5	4.891E-5	3.402	0.2857	5.176	0.04418	5.405	0.02187	3.677	0.3274	5.988	0.01165
Oleoside Dimethyl Ester	metab_49109	1.165	0.000183	0.0002083	1.822	0.003226	1.946	0.3645	2.825	0.8208	4.796	0.2701	2.562	0.7165
4-(Beta-D-Glucopyranosyloxy)Phenylacetic Acid	metab_47998	1.165	2.53E-5	4.891E-5	4.677	0.01052	3.996	0.04319	3.979	0.0265	3.573	0.07763	3.221	0.1429
Gentamicin B	metab_19394	1.165	0.0002504	0.0002792	3.243	0.3626	4.665	0.07012	3.862	0.1591	3.82	0.2044	3.664	0.2243
Phe-Asn-Pro	metab_45211	1.164	1.616E-5	4.891E-5	4.559	0.0244	3.429	0.1893	4.81	0.01347	5.922	0.004457	4.511	0.02476
Muramic Acid	metab_30325	1.164	7.305E-5	9.59E-5	4.713	0.05097	4.018	0.2673	5.006	0.03418	4.309	0.09457	4.187	0.173
Kanzonol V	metab_46589	1.163	1.635E-5	4.891E-5	3.97	0.1177	4.281	0.134	5.579	0.01849	3.601	0.3051	5.048	0.02093
Arg-Tyr	metab_28397	1.163	4.927E-5	7.065E-5	2.743	0.1794	4.581	0.08983	5.264	0.2469	4.594	0.2279	5.267	0.218
Hydroxyhomodestruxin B	metab_12372	1.163	3.518E-5	5.759E-5	4.371	0.04256	4.463	0.07642	3.33	0.3546	5.256	0.01943	3.447	0.3501
Glu-Thr-Tyr	metab_47069	1.163	1.325E-5	4.891E-5	2.459	0.2902	3.588	0.1195	5.032	0.01531	4.253	0.05481	4.537	0.03109
Swertiamarin	metab_35952	1.163	1.325E-5	4.891E-5	4.941	0.01245	3.677	0.07632	3.007	0.09666	5.118	0.01143	2.651	0.01018
3'-Deoxythymidine	metab_29330	1.162	1.325E-5	4.891E-5	4.459	0.1355	3.963	0.1243	5.54	0.008671	6.617	0.006487	4.786	0.01295
5,6-Dihydroxytetradecanedioic Acid	metab_36839	1.162	1.325E-5	4.891E-5	4.201	0.02917	3.227	0.2553	4.624	0.01502	4.456	0.02369	4.089	0.03262
Batrachotoxin	metab_37060	1.162	1.712E-5	4.891E-5	4.329	0.0219	4.424	0.01652	3.571	0.2447	5.721	0.008167	3.9	0.1044
Trp-Asn-Ser	metab_2696	1.161	2.038E-5	4.891E-5	3.324	0.2649	3.081	0.2076	4.572	0.05888	5.713	0.01086	4.001	0.2877
Tyr-Gly-Lys	metab_36198	1.161	3.441E-5	5.698E-5	4.505	0.009705	2.405	0.353	3.221	0.2193	3.834	0.0656	2.894	0.4381

Chloramphenicol	metab_50657	1.160	1.325E-5	4.891E-5	3.938	0.0652	5.313	0.007146	5.537	0.01719	3.589	0.06685	4.826	0.008057
Na-P-Hydroxycoumaroyltryptophan	metab_45834	1.160	1.518E-5	4.891E-5	2.847	0.2354	2.419	0.06888	4.003	0.03025	4.785	0.01498	3.493	0.04602
(1Xi,3S)-1,2,3,4-Tetrahydro-1-Methyl-Beta-Carboline-1,3-Dicarboxylic Acid	metab_7288	1.160	1.421E-5	4.891E-5	3.856	0.02576	3.017	0.07801	4.56	0.03	5.482	0.0093	3.457	0.2092
Phe-Lys	metab_2135	1.159	1.325E-5	4.891E-5	3.827	0.1255	4.275	0.04923	5.537	0.01035	6.294	0.005915	5.233	0.009186
Thapsigargin	metab_44196	1.159	2.49E-5	4.891E-5	3.021	0.5749	4.699	0.01632	5.346	0.01128	2.757	0.6146	5.214	0.01113
Deacetylvinblastine	metab_48171	1.159	1.325E-5	4.891E-5	3.619	0.2577	4.972	0.02961	6.252	0.01108	5.582	0.012	5.448	0.0134
(1R,2R,4S)-P-Menthane-1,2,8-Triol 8-Glucoside	metab_7503	1.159	1.325E-5	4.891E-5	6.354	0.002938	5.248	0.04402	4.991	0.04896	3.973	0.1779	4.838	0.03164
Cervonoyl Ethanolamide	metab_16912	1.158	0.004159	0.004254	5.158	0.3044	4.849	1.248	3.077	0.7618	3.767	1.089	4.743	0.04827
Carbidopa	metab_28230	1.158	2.591E-5	4.929E-5	2.39	0.2309	2.148	0.001846	3.477	0.4048	4.634	0.3285	2.238	0.003243
Ala-Leu-His	metab_2781	1.157	3.81E-5	6.046E-5	3.541	0.1521	3.143	0.3265	4.514	0.3673	5.157	0.235	4.463	0.2059
N-Desmethyltamoxifen	metab_33988	1.157	2.359E-5	4.891E-5	3.652	0.07298	3.685	0.0605	5.138	0.02481	4.804	0.02999	4.981	0.02
Dihydrodigoxin	metab_12067	1.157	2.53E-5	4.891E-5	5.7	0.0228	4.962	0.0859	4.968	0.07201	3.462	0.006603	5.605	0.02229
Cyclo(Phe-Leu)	metab_17284	1.157	1.325E-5	4.891E-5	2.883	0.09932	4.155	0.04029	5.476	0.008721	4.387	0.02673	5.083	0.00987
Hovenine A	metab_51126	1.157	1.712E-5	4.891E-5	5.653	0.009808	4.2	0.03171	3.342	0.2001	4.566	0.03983	4.306	0.05984
Hispidin	metab_35281	1.156	0.0003442	0.0003774	4.667	0.2258	4.084	0.8022	2.814	0.3162	3.127	0.7338	2.577	0.01018
Dulcoside A	metab_50873	1.156	2.515E-5	4.891E-5	4.512	0.07062	3.549	0.2331	4.518	0.1124	5.143	0.0933	4.73	0.02387
L-Cis-Cyclo(Aspartylphenylalanyl)	metab_36290	1.156	2.411E-5	4.891E-5	2.938	0.2049	4.769	0.01522	5.505	0.008843	4.34	0.03976	5.501	0.009446
Met-Glu-Leu	metab_44320	1.155	1.325E-5	4.891E-5	2.416	0.003263	3.336	0.1785	4.571	0.02487	2.61	0.07745	4.35	0.02939
Arg-Val-Arg	metab_646	1.155	1.616E-5	4.891E-5	5.619	0.02507	4.799	0.07401	4.409	0.1385	5.185	0.04261	5.377	0.09482
Amastatin	metab_37755	1.154	2.075E-5	4.891E-5	4.551	0.03622	4.52	0.03717	6.002	0.01399	4.856	0.04199	5.427	0.01344
Dienogest	metab_27888	1.154	2.075E-5	4.891E-5	5.951	0.006819	5.066	0.04065	5.011	0.0638	3.485	0.1455	5.445	0.01748
Phe-Met	metab_46275	1.154	1.421E-5	4.891E-5	2.709	0.2137	3.183	0.1751	4.568	0.01758	4.912	0.01584	4.269	0.02288
7-Acetyllycopsamine	metab_48387	1.153	1.421E-5	4.891E-5	4.317	0.02685	3.729	0.07064	3.228	0.2874	5.463	0.009111	3.879	0.04398
6-Quinolinecarboxylic Acid	metab_35648	1.153	0.0001061	0.0001307	4.522	0.01777	2.839	0.2128	2.43	0.1634	2.395	0.01705	2.369	0.1301
Tylosin	metab_33335	1.153	2.298E-5	4.891E-5	5.708	0.007247	4.33	0.05395	3.515	0.3513	5.468	0.03701	3.3	0.2703
Gibberellin A20 13-Glucoside	metab_33223	1.153	2.654E-5	5.005E-5	3.253	0.3334	3.043	0.4037	4.319	0.149	5.789	0.3455	3.91	0.1523
Pravastatin Lactone	metab_46085	1.152	1.325E-5	4.891E-5	4.21	0.01949	4.441	0.03511	6.07	0.009134	5.283	0.01086	5.236	0.009159
3',5'-Di-C-Glucosylphloretin	metab_43940	1.152	0.0001216	0.0001465	4.928	0.07268	3.82	0.6823	2.639	0.006756	3.035	0.5948	2.606	0.01018
Neosaxitoxin	metab_28753	1.152	1.807E-5	4.891E-5	3.642	0.1506	4.164	0.08752	5.66	0.03019	4.775	0.1033	4.622	0.06835
Pe(O-12:0/6:0)	metab_15003	1.152	3.49E-5	5.736E-5	5.594	0.1373	5.122	0.1905	3.389	0.9471	4.993	0.1267	3.692	1.073
Indole-3-Carboxylic Acid	metab_28760	1.152	1.325E-5	4.891E-5	5.074	0.00808	4.829	0.004916	4.952	0.00652	4.747	0.006681	4.103	0.04181
Asp-Glu	metab_50344	1.152	2.359E-5	4.891E-5	3.156	0.1893	2.936	0.001172	4.338	0.104	5.521	0.0531	3.284	0.2945
Trp-Glu	metab_34526	1.151	2.063E-5	4.891E-5	2.695	0.2465	3.432	0.08074	3.961	0.0457	5.145	0.01215	3.017	0.2794
Algestone	metab_47995	1.151	1.325E-5	4.891E-5	3.843	0.04486	3.671	0.09409	5.315	0.01224	4.854	0.01392	4.513	0.03081
Verlukast	metab_50431	1.151	4.504E-5	6.637E-5	4.652	0.1197	5.273	0.08033	3.95	0.006771	4.012	0.003721	4.073	0.1981

Ala-Val-Pro	metab_35616	1.151	1.325E-5	4.891E-5	2.469	0.2635	3.498	0.04134	4.666	0.01007	4.984	0.01258	4.212	0.01084
Edulisin I	metab_47659	1.151	1.754E-5	4.891E-5	4.945	0.01555	3.403	0.193	2.809	0.4371	4.375	0.02652	3.701	0.1527
Phe-Gly-Ser	metab_48817	1.150	1.325E-5	4.891E-5	2.609	0.5658	3.753	0.02654	4.486	0.02917	5.203	0.01369	3.612	0.04337
Cyclo(D-Trp-D-Asp-Pro-D-Val-Leu)	metab_48339	1.150	2.075E-5	4.891E-5	3.771	0.08973	4.682	0.02092	4.447	0.0657	5.528	0.01433	3.859	0.1187
Lps(16:0)	metab_40668	1.150	1.325E-5	4.891E-5	3.639	0.2504	5.38	0.01457	4.594	0.03902	4.408	0.04928	5.147	0.01995
Syrups, Hydrolyzed Starch, Hydrogenated	metab_49958	1.150	0.0001245	0.0001495	5.236	0.0112	3.326	0.1796	3.311	0.185	5.69	0.03716	3.228	0.07777
Pgp(5-Iso Pgf2Vi/22:5(7Z,10Z,13Z,16Z,19Z))	metab_11610	1.149	1.9E-5	4.891E-5	3.559	0.07268	4.706	0.04289	5.791	0.02378	3.6	0.006603	5.441	0.03745
Gly-Pro-Arg-Pro	metab_37282	1.149	1.518E-5	4.891E-5	4.855	0.0148	4.025	0.08328	4.979	0.01988	4.632	0.02085	5.008	0.0101
Hypaphorine	metab_10358	1.149	2.317E-5	4.891E-5	3.532	0.134	3.809	0.1465	5.325	0.009553	5.313	0.009003	4.985	0.01419
N-Fructosyl Tyrosine	metab_2237	1.149	1.325E-5	4.891E-5	4.552	0.04249	5.139	0.01985	6.592	0.004349	5.513	0.015	5.616	0.004252
Ser Thr Glu	metab_29657	1.148	1.9E-5	4.891E-5	3.222	0.001283	3.239	0.06567	4.683	0.05224	5.079	0.03085	4.416	0.0357
Smgdg(O-24:5/3:0)	metab_51142	1.148	5.107E-5	7.248E-5	3.352	0.1884	4.093	0.1877	3.912	0.1199	5.464	0.01727	4.209	0.08663
Rosarin	metab_50404	1.148	2.535E-5	4.891E-5	5.33	0.0198	3.893	0.001173	4.931	0.1066	5.32	0.07091	4.009	0.01021
Cucurbitacin I	metab_23630	1.148	0.0001023	0.0001268	4.595	0.03596	4.497	0.08754	5.656	0.02395	5.036	0.03426	4.597	0.1596
1-(2,6-Dimethoxyphenoxy)-3-[4-(4-Fluorophenyl)Piperazin-1-Yl]Propan-2-Ol	metab_25278	1.147	2.535E-5	4.891E-5	5.019	0.06776	4.46	0.1518	5.94	0.005111	5.669	0.01825	5.014	0.03748
Hordatine B	metab_24473	1.147	2.23E-5	4.891E-5	4.824	0.03712	5.545	0.02178	4.883	0.08535	2.309	1.46	5.602	0.01806
(9E)-10-Nitrooctadec-9-Enoylcarnitine	metab_42075	1.147	1.325E-5	4.891E-5	5.453	0.02546	3.556	0.001173	3.704	0.00677	3.767	0.003721	3.671	0.0102
Phenylalanyl-Gamma-Glutamate	metab_33167	1.147	2.156E-5	4.891E-5	3.727	0.07479	3.789	0.07408	5.267	0.009096	5.886	0.009794	4.82	0.01038
Glu Lys Phe Asp	metab_45604	1.147	1.325E-5	4.891E-5	3.255	0.2536	4.708	0.03588	5.101	0.01819	5.178	0.01612	5.382	0.00918
Phe Gly Phe Gly	metab_20134	1.147	2.411E-5	4.891E-5	3.72	0.212	4.304	0.07543	4.67	0.07526	5.831	0.007744	3.675	0.1398
Arg-Asp-Asp	metab_36231	1.147	2.53E-5	4.891E-5	3.257	0.1916	4.035	0.05776	5.243	0.01607	5.165	0.008797	5.165	0.01446
Arg-Asp-Tyr	metab_2719	1.146	2.49E-5	4.891E-5	5.588	0.01523	4.816	0.02369	3.868	0.1407	6.156	0.01923	3.9	0.1472
Phe-Thr-Tyr	metab_49114	1.145	2.075E-5	4.891E-5	2.83	0.1848	4.299	0.06689	4.429	0.03966	4.233	0.05675	4.981	0.01084
Asp-Leu-Ile	metab_44853	1.145	2.515E-5	4.891E-5	2.528	0.02635	3.421	0.2256	4.119	0.05339	5.272	0.00821	3.488	0.134
Pi(5-Iso Pgf2Vi/20:4(5Z,8Z,11Z,14Z))	metab_45057	1.144	1.325E-5	4.891E-5	5.656	0.01473	4.449	0.08318	4.153	0.1347	3.318	0.1178	4.824	0.02348
Arg-Glu-Leu	metab_49290	1.144	0.001155	0.001212	1.422	0.7672	1.043	0.07151	1.839	1.347	3.266	1.09	2.605	1.269
Asp-Ala	metab_50391	1.144	1.325E-5	4.891E-5	2.847	0.003271	2.77	0.001171	4.002	0.289	5.187	0.03338	2.885	0.01019
Fahfa(6:0/22:5)	metab_40598	1.143	6.281E-5	8.531E-5	4.569	0.02268	3.061	0.5193	3.698	0.2262	4.562	0.03913	2.634	0.2151
Corynoxeine	metab_39066	1.143	1.99E-5	4.891E-5	4.746	0.05884	4.578	0.07438	5.473	0.01682	4.801	0.03004	5.814	0.00598
3-(1-(Cyclohexylmethyl)-1H-Indazole-3-Carboxamido)-2,2-Dimethylsuccinicacid	metab_36056	1.143	2.23E-5	4.891E-5	4.823	0.01836	4.047	0.09472	4.835	0.01848	4.418	0.07755	5.041	0.02031
Sulfo Jasmonate	metab_46130	1.142	0.0002337	0.0002617	4.837	0.01062	3.271	0.09503	3.399	0.1354	3.256	0.1448	2.927	0.2244
Riboflavin	metab_45909	1.142	6.72E-5	8.996E-5	4.705	0.01347	3.152	0.3327	3.83	0.1501	3.436	0.2276	3.61	0.1222
Edoxaban	metab_50994	1.142	2.411E-5	4.891E-5	5.199	0.02653	3.473	0.001173	4.252	0.1276	5.376	0.01166	4.339	0.06102
Gibberellin A8	metab_32968	1.142	0.0001551	0.00018	3.076	0.1285	4.82	0.06439	5.335	0.1455	5.123	0.09832	5.304	0.2998
Avermectin A1A	metab_45263	1.141	1.616E-5	4.891E-5	3.258	0.003274	4.57	0.07422	5.443	0.02136	3.392	0.00372	5.481	0.02491

Lamiide	metab_45930	1.140	1.325E-5	4.891E-5	5.09	0.01232	3.142	0.001172	3.29	0.006768	3.512	0.1065	3.257	0.0102
Glu-Ser-Ala	metab_32291	1.140	1.325E-5	4.891E-5	3.529	0.1677	3.014	0.1267	4.634	0.01829	4.778	0.02966	4.036	0.04118
Ethyl 5-Hexenoate	metab_10319	1.140	1.9E-5	4.891E-5	3.514	0.06747	3.09	0.1467	2.339	0.06166	4.716	0.01709	2.341	0.02936
Arg-Thr-Ile	metab_49472	1.140	6.394E-5	8.64E-5	3.388	0.1212	2.988	0.1217	4.474	0.3835	4.267	0.3332	4.32	0.4078
Milataxel	metab_45376	1.140	1.325E-5	4.891E-5	4.934	0.04353	4.268	0.08259	4.512	0.06553	3.326	0.211	5.178	0.01467
Thr Val Val	metab_10975	1.139	4.184E-5	6.344E-5	4.392	0.0327	4.387	0.0487	3.349	0.4646	5.395	0.01002	3.634	0.1071
Morroniside	metab_28897	1.139	1.325E-5	4.891E-5	6.942	0.004205	6.036	0.004059	4.843	0.02852	6.76	0.009596	5.077	0.02153
Petanin	metab_32248	1.138	2.359E-5	4.891E-5	3.315	0.003274	5.356	0.05936	4.736	0.1534	3.463	0.03436	4.747	0.2815
Ganoderic Acid I	metab_37071	1.138	3.441E-5	5.698E-5	2.731	0.0455	3.712	0.1438	4.776	0.03949	4.779	0.02505	4.702	0.02473
5-Fluoro-3-[3-[4-(5-Methoxypyrimidin-4-Yl)Piperazin-1-Yl]Propyl]-1H-Indole	metab_38453	1.137	2.53E-5	4.891E-5	3.019	0.5451	4.855	0.01359	4.694	0.02285	2.911	0.322	5.377	0.01765
Flecainide	metab_44908	1.137	1.807E-5	4.891E-5	3.276	0.1027	3.919	0.07416	5.245	0.01098	5.226	0.01435	5.007	0.01052
Acorafloxacin	metab_36907	1.137	2.455E-5	4.891E-5	3.704	0.1203	3.648	0.1327	4.96	0.01056	5.301	0.0105	4.875	0.01891
Leukotriene D5	metab_47445	1.137	1.325E-5	4.891E-5	5.209	0.01214	4.221	0.06618	3.938	0.09483	2.431	0.9989	4.454	0.0625
Glu-Glu-Leu	metab_37020	1.137	1.518E-5	4.891E-5	2.791	0.2122	4.452	0.01723	5.235	0.01399	3.41	0.1124	5.203	0.01775
Arg-Leu	metab_32756	1.137	1.325E-5	4.891E-5	4.03	0.04856	3.866	0.05214	5.36	0.06076	5.94	0.005439	4.938	0.06281
Thr-Asn-Pro	metab_28959	1.137	0.0002384	0.0002665	2.439	0.3881	2.552	0.311	3.315	0.4908	5.222	0.05786	2.775	0.2545
Asn Gly Phe	metab_5863	1.136	3.848E-5	6.072E-5	3.338	0.1419	3.285	0.07892	4.67	0.1624	5.643	0.007686	3.61	0.2639
Verbasoside	metab_47730	1.136	1.325E-5	4.891E-5	5.997	0.004296	3.942	0.104	4.541	0.05211	5.51	0.014	4.425	0.03451
Uridine 2'-Phosphate	metab_32553	1.136	1.325E-5	4.891E-5	4.093	0.03972	5.059	0.0105	5.437	0.08211	2.839	0.003716	5.235	0.05685
Ser-Glu-Gln	metab_32528	1.135	2.23E-5	4.891E-5	2.66	0.003268	3.862	0.08451	4.934	0.06324	4.942	0.01938	4.578	0.0513
Virginiamycin M1	metab_34983	1.135	1.325E-5	4.891E-5	3.124	0.003273	4.801	0.03303	5.423	0.01734	4.936	0.02773	4.708	0.04813
Ser-Glu-Met	metab_49163	1.135	1.939E-5	4.891E-5	4.492	0.02322	3.01	0.2772	4.367	0.1868	5.284	0.0502	3.517	0.3945
N-Fructosyl Phenylalanine	metab_28128	1.135	1.325E-5	4.891E-5	4.611	0.02016	5.421	0.00834	6.803	0.008633	6.041	0.01684	5.799	0.006142
2-O-Galloylgalactaric Acid	metab_49803	1.134	3.902E-5	6.072E-5	5.491	0.01512	3.407	0.2117	3.173	0.006767	4.893	0.01997	3.139	0.0102
3,5,6-Trihydroxy-5-(Hydroxymethyl)-2-Methoxy-2-Cyclohexen-1-One	metab_36947	1.134	2.359E-5	4.891E-5	4.372	0.02082	3.477	0.04459	2.198	0.006728	3.802	0.04203	2.41	0.285
Val-Gly-Leu	metab_10249	1.133	2.455E-5	4.891E-5	4.547	0.03346	4.079	0.06382	3.551	0.0909	6.017	0.008907	3.475	0.1642
Abbott-195773	metab_44411	1.133	2.535E-5	4.891E-5	5.203	0.02481	4.54	0.05442	4.54	0.05539	2.85	1.099	5.286	0.01432
Nicotinic Acid	metab_29379	1.133	1.9E-5	4.891E-5	4.659	0.06497	4.228	0.136	5.161	0.00584	4.066	0.05507	4.51	0.01866
Evodenoson	metab_35163	1.132	2.681E-5	5.035E-5	2.847	0.2033	4.277	0.01568	5.034	0.02574	4.996	0.0208	4.237	0.04507
N-((Tetrahydro-5-Oxo-2-Furanyl)Carbonyl)-L-Histidyl-L-Prolinamide	metab_48840	1.132	1.421E-5	4.891E-5	4.457	0.02494	3.949	0.04448	4.187	0.1203	5.236	0.0217	3.072	0.2604
Leu-Ser-Leu	metab_45156	1.132	5.443E-5	7.614E-5	2.445	0.003264	2.569	0.2181	3.859	0.03413	4.697	0.01176	2.729	0.2262
Asp-Ile-Leu	metab_37658	1.132	1.325E-5	4.891E-5	2.908	0.1937	4.33	0.02483	4.564	0.01747	5.236	0.01742	4.424	0.03618
L-Beta-Aspartyl-L-Phenylalanine	metab_18585	1.131	2.075E-5	4.891E-5	3.017	0.2727	3.246	0.1218	4.329	0.05227	4.558	0.02224	4.475	0.02454
Eriojaposide A	metab_13513	1.131	2.53E-5	4.891E-5	5.216	0.0205	3.647	0.3403	3.667	0.2827	4.849	0.03238	4.346	0.07098
Asn-Asn-Ile	metab_33094	1.130	1.712E-5	4.891E-5	3.829	0.0587	3.237	0.1493	4.798	0.2289	5.525	0.01545	4.227	0.2419

Cys-Arg-Glu-Lys-Ala	metab_44701	1.130	2.075E-5	4.891E-5	2.976	0.3849	3.916	0.1819	4.8	0.04149	5.316	0.009847	4.746	0.05514
Amikacin	metab_39422	1.130	3.497E-5	5.736E-5	3.247	0.3807	2.153	0.08398	2.286	0.0457	4.866	0.02641	2.233	0.01015
2-Hydroxy-Imipramine Glucuronide	metab_24249	1.130	2.075E-5	4.891E-5	4.061	0.1853	4.231	0.1889	5.787	0.01702	5.097	0.04646	4.855	0.09106
Leucomycin	metab_44534	1.130	1.9E-5	4.891E-5	4.987	0.01015	4.612	0.03319	4.868	0.02463	3.042	0.03262	4.564	0.03581
3-Feruloyl-1,5-Quinolactone	metab_13191	1.129	0.0004106	0.0004468	5.454	0.01348	3.635	0.04473	3.753	0.1447	3.726	0.06722	3.78	0.1789
Cis-3-Hexenyl Acetate	metab_18988	1.129	2.992E-5	5.288E-5	3.834	0.09044	3.96	0.05033	3.329	0.1568	5.298	0.008663	3.761	0.07054
Glu-Arg-His	metab_9167	1.129	1.325E-5	4.891E-5	5.33	0.04239	5.531	0.05215	7.048	0.008538	6.134	0.01135	6.25	0.01399
Ser-Met	metab_49605	1.129	1.325E-5	4.891E-5	3.78	0.09986	4.247	0.03012	5.609	0.01856	6.001	0.02151	5.179	0.01259
Phe Cys Thr	metab_10082	1.129	2.359E-5	4.891E-5	3.247	0.2222	3.16	0.2915	4.638	0.02072	5.249	0.01202	4.225	0.1608
Cilastatin	metab_37702	1.128	1.9E-5	4.891E-5	3.425	0.003274	3.499	0.0971	4.834	0.01291	5.082	0.009538	4.686	0.01808
Ser-Leu	metab_49083	1.128	2.53E-5	4.891E-5	3.786	0.06511	3.776	0.04042	5.117	0.02879	6.137	0.007245	4.582	0.01731
Doramectin	metab_45957	1.128	1.9E-5	4.891E-5	4.062	0.374	5.1	0.04312	5.931	0.01154	3.517	0.09332	5.714	0.01891
Atractyloside B	metab_36409	1.128	1.325E-5	4.891E-5	5.431	0.008416	4.433	0.03717	4.248	0.04018	3.758	0.1463	5.102	0.01483
28-Hydroxywithanolide E	metab_6131	1.127	1.325E-5	4.891E-5	5.06	0.01858	5.406	0.0116	5.994	0.02018	6.489	0.009977	4.828	0.05967
Val Val Thr	metab_2852	1.126	1.807E-5	4.891E-5	2.925	0.2964	3.831	0.1642	4.676	0.1963	5.569	0.01817	4.356	0.2753
Penicillin V	metab_50476	1.126	2.455E-5	4.891E-5	3.898	0.2853	6.062	0.01762	5.955	0.0337	4.74	0.06961	5.938	0.05053
Mabioside E	metab_18955	1.126	1.325E-5	4.891E-5	5.81	0.012	6.042	0.01552	6.081	0.01017	4.892	0.04714	5.384	0.07111
Dihydroceramide	metab_39862	1.126	2.164E-5	4.891E-5	3.131	0.05103	3.448	0.2021	4.77	0.02078	3.244	0.003719	4.493	0.02284
N-Acetyl-Glucosamine 1-Phosphate	metab_33362	1.125	2.359E-5	4.891E-5	3.347	0.11	4.254	0.04315	4.92	0.03024	3.327	0.003719	4.036	0.02529
Trp Ser	metab_27077	1.125	2.075E-5	4.891E-5	3.986	0.1252	3.789	0.2544	4.558	0.07031	5.456	0.01965	3.302	0.03942
4-Acetyl-3-Hydroxy-5-Methylphenyl Beta-D-Glucopyranoside	metab_37931	1.125	1.325E-5	4.891E-5	4.797	0.007636	5.011	0.0149	4.039	0.05443	4.631	0.03241	4.97	0.008202
Eleutherazineb	metab_19341	1.125	1.635E-5	4.891E-5	5.448	0.02089	4.418	0.06518	3.204	0.4994	4.068	0.1278	4.265	0.08589
5-Benzylacyclouridine	metab_36403	1.124	1.421E-5	4.891E-5	3.614	0.003275	3.679	0.0501	4.958	0.01414	3.821	0.05399	4.832	0.006365
Moschamine	metab_19007	1.124	1.712E-5	4.891E-5	4.254	0.04542	3.609	0.1395	4.562	0.04218	5.519	0.01125	3.31	0.2164
Pro-Ile-His	metab_24142	1.124	2.455E-5	4.891E-5	4.578	0.06841	4.756	0.05195	5.865	0.01449	5.263	0.03119	4.781	0.0735
Glu-Phe-Asp	metab_34262	1.124	1.807E-5	4.891E-5	4.935	0.006374	3.432	0.1726	4.383	0.02989	5.027	0.0158	4.33	0.03859
Cyclo(His-Pro)	metab_2859	1.124	1.518E-5	4.891E-5	3.123	0.2806	3.908	0.08918	5.029	0.02494	5.506	0.1533	4.059	0.04769
Bergamottin	metab_36651	1.124	1.325E-5	4.891E-5	5.591	0.01124	5.417	0.005535	4.163	0.06026	4.774	0.01677	5.236	0.00486
Lexaptepid Pegol	metab_48761	1.123	1.616E-5	4.891E-5	4.416	0.06141	5.657	0.0197	5.037	0.09201	5.292	0.05351	4.883	0.0868
Oxytetracycline	metab_8356	1.123	1.325E-5	4.891E-5	4.82	0.001284	4.809	0.001859	5.873	0.006517	4.891	0.006605	4.997	0.06622
Phenazopyridine	metab_38314	1.123	1.9E-5	4.891E-5	2.818	0.00327	3.324	0.4162	4.831	0.01429	4.918	0.02075	3.909	0.1064
Bpd-Ma	metab_34096	1.123	1.712E-5	4.891E-5	5.24	0.008161	5.591	0.01255	5.28	0.02478	5.865	0.008519	4.576	0.02395
D-Fructose 1-Phosphate	metab_610	1.123	2.298E-5	4.891E-5	3.438	0.08648	5.495	0.01533	5.055	0.01876	3.396	0.006602	5.315	0.01017
Gly-Ala-Asp	metab_31899	1.122	1.325E-5	4.891E-5	4.006	0.08291	3.661	0.02619	5.075	0.03746	5.994	0.02158	4.612	0.05913
Hesperetin 7-O-Rutinoside	metab_38492	1.122	0.0007464	0.0007935	4.384	0.3629	3.87	0.82	2.394	0.006744	3.213	0.7591	2.419	0.1434

Alpha-Hydroxy-Tamoxifen-O-Glucuronide	metab_39056	1.122	1.421E-5	4.891E-5	3.017	0.344	3.816	0.1446	5.044	0.01312	4.135	0.0862	5.016	0.01255
Pi(6:0/16:2)	metab_47930	1.122	1.518E-5	4.891E-5	5.602	0.005412	4.742	0.03197	5.043	0.02832	3.856	0.1014	4.667	0.0307
Teumarin	metab_13979	1.121	0.001037	0.001091	4.851	0.02217	3.063	0.1828	3.007	0.002696	3.028	0.04021	3.02	0.003258
D-Ribose 5-Phosphate	metab_50508	1.121	1.325E-5	4.891E-5	3.075	0.6407	5.129	0.02657	4.886	0.03672	4	0.07553	4.599	0.0579
Desacetoxyvindoline	metab_38210	1.121	1.325E-5	4.891E-5	4.748	0.01029	3.944	0.01934	3.247	0.1311	4.693	0.02535	2.771	0.09794
Cyclo(Arg-Gly-Asp-D-Phe-Val)	metab_45257	1.120	1.325E-5	4.891E-5	2.666	0.2027	3.635	0.08898	4.856	0.04007	4.984	0.02193	4.22	0.05771
Leu-Ser-His	metab_49691	1.120	1.616E-5	4.891E-5	2.504	0.003265	2.427	0.001169	3.534	0.4836	4.927	0.09561	2.557	0.02464
His-Gln-Lys	metab_171	1.120	1.712E-5	4.891E-5	5.595	0.01917	4.22	0.1867	5.117	0.05629	5.839	0.01601	5.189	0.04951
Isoschaftoside	metab_9745	1.120	9.644E-5	0.0001204	5.103	0.1706	4.992	0.14	4.186	0.1542	3.975	0.334	3.783	0.003261
Arthrobactin	metab_19463	1.120	1.325E-5	4.891E-5	5.517	0.01404	4.886	0.03979	3.825	0.4011	5.741	0.01778	4.571	0.03933
N-Fructosyl Isoleucine	metab_28583	1.120	1.325E-5	4.891E-5	5.338	0.01537	5.56	0.01305	6.899	0.008866	6.059	0.01199	5.927	0.01194
Atractysucrose Iiia	metab_17903	1.120	0.0003449	0.0003781	4.218	0.1831	5.235	0.03845	4.34	0.1691	4.586	0.07477	4.25	0.3505
Gdc-0834 Racemate	metab_49014	1.120	1.325E-5	4.891E-5	3.505	0.003274	5.535	0.008853	5.412	0.02924	3.983	0.2244	5.787	0.01614
Trp-Tyr-Phe	metab_38636	1.120	1.325E-5	4.891E-5	5.554	0.005996	4.441	0.04487	4.809	0.02034	5.231	0.01939	3.864	0.1312
Leu-Gln	metab_32709	1.120	1.325E-5	4.891E-5	4.284	0.0496	4.713	0.02122	5.937	0.05233	6.472	0.01066	5.674	0.06925
Coumaroyl(3-Ome)(-3)Glc(A1-2B)L-Fruf	metab_36542	1.119	1.325E-5	4.891E-5	5.165	0.009542	5.112	0.01606	4.611	0.0157	4.867	0.02154	4.017	0.05144
Dronedarone	metab_2732	1.119	0.0007421	0.0007889	4.965	0.02752	5.401	0.01593	4.733	0.3777	5.441	0.8247	4.07	0.1812
N(Epsilon)-(Carboxyethyl)Lysine	metab_28264	1.119	1.712E-5	4.891E-5	4.286	0.1614	4.016	0.08839	5.502	0.126	6.379	0.02345	4.824	0.2233
(+/-)-Ethylketocyclazocine	metab_44748	1.119	3.902E-5	6.072E-5	2.503	0.08102	2.278	0.001167	2.426	0.006746	4.681	0.01856	2.393	0.01016
Asn-Thr-Tyr	metab_32603	1.118	3.75E-5	5.994E-5	2.724	0.003269	3.129	0.2636	4.675	0.1131	4.948	0.1862	3.882	0.5598
Adenosine, 8-(Butylamino)-N-Cyclopentyl-	metab_43266	1.118	1.325E-5	4.891E-5	3.635	0.2251	3.862	0.04653	5.439	0.01004	5.467	0.007684	4.589	0.01631
Val-Val-Pro	metab_47580	1.118	2.455E-5	4.891E-5	3.134	0.2552	3.099	0.2342	4.646	0.01287	5.157	0.008416	3.966	0.06159
Aloesol 7-Glucoside	metab_39050	1.117	3.625E-5	5.857E-5	5.493	0.01678	3.534	0.1506	3.193	0.1323	4.192	0.08555	3.144	0.1547
N-(3,5-Dimethylphenyl)-2-(5-Pyrazin-2-Yl-1,3,4-Oxadiazol-2-Yl)Acetamide	metab_28085	1.117	1.325E-5	4.891E-5	4.679	0.04485	5.237	0.01543	6.603	0.009522	5.859	0.01827	5.594	0.01871
Dgdg(18:3/18:3)	metab_15124	1.117	0.0002784	0.0003085	5.916	0.0854	4.014	0.4445	3.91	0.002698	4.074	0.3914	3.923	0.003261
3-Pyridinemethanol, 6-Amino-Alpha-(((1-Methyl-4-Phenylbutyl)Amino)Methyl)-	metab_49435	1.117	1.325E-5	4.891E-5	4.17	0.06148	3.888	0.05408	5.264	0.09351	6.052	0.02435	4.915	0.04889
(3S,5S)-5-(Hydroxymethyl)-3-[(1R)-1-Hydroxy-6-Methylheptyl]Oxolan-2-One	metab_17320	1.117	3.102E-5	5.388E-5	4.819	0.01615	2.975	0.3364	3.367	0.06086	3.578	0.08403	2.993	0.31
Arg-Gln-Ile	metab_33129	1.116	1.325E-5	4.891E-5	3.114	0.1809	3.657	0.09876	4.772	0.009136	5.62	0.01502	4.323	0.0459
Afn911	metab_47685	1.116	2.075E-5	4.891E-5	4.071	0.07283	4.139	0.0409	5.733	0.005543	5.533	0.006601	5.03	0.02318
(2S,3R,4R)-2-Amino-3,4-Dihydroxy-2-(Hydroxymethyl)-14-Oxoicos-6-Enoic Acid	metab_40033	1.116	0.0001223	0.0001471	3.853	0.119	3.669	0.151	4.788	0.03215	4.006	0.1026	3.895	0.1138
5'-((Z)-Feruloyl) 3-(2'-Methylarabinosylxylose)	metab_36879	1.116	1.325E-5	4.891E-5	2.818	0.199	3.714	0.087	5.065	0.01537	4.438	0.03071	4.678	0.02125
N-Acetyl-Dl-Glutamic Acid	metab_49666	1.115	6.432E-5	8.676E-5	4.373	0.01492	4.394	0.01515	3.578	0.04522	4.572	0.06153	4.413	0.06859
Tyr Gln	metab_28887	1.115	1.325E-5	4.891E-5	3.857	0.2053	4.589	0.05127	5.609	0.0078	6.433	0.004423	5.057	0.01493
Tyr-Asn	metab_1972	1.114	1.325E-5	4.891E-5	3.335	0.2062	4.209	0.006715	5.164	0.01776	5.897	0.01448	4.619	0.03204
Dg(9M5/9D3/0:0)	metab_16274	1.114	1.616E-5	4.891E-5	6.362	0.02198	5.678	0.03979	4.907	0.1329	6.394	0.008409	5.799	0.01556

Arg Arg	metab_349	1.114	3.802E-5	6.037E-5	3.208	0.05423	3.289	0.1284	4.633	0.09287	5.577	0.02262	3.787	0.3044
Gly-Trp	metab_35754	1.114	1.325E-5	4.891E-5	2.557	0.2617	3.69	0.06079	4.429	0.016	5.077	0.01221	3.905	0.03628
Phe-Ala-Thr	metab_47746	1.114	1.616E-5	4.891E-5	4.021	0.08212	3.899	0.03065	5.115	0.01311	6.214	0.005588	4.788	0.01497
D-Erythroascorbic Acid 1'-A-D-Xylopyranoside	metab_32915	1.113	1.325E-5	4.891E-5	5.471	0.01461	4.688	0.01463	5.573	0.03835	5.119	0.05027	4.883	0.03642
Beclomethasone	metab_33058	1.113	0.000331	0.0003637	3.891	0.06022	2.526	0.00117	3.983	0.7883	4.545	0.0631	3.659	0.6875
Trp-Met	metab_21994	1.113	0.0001061	0.0001307	2.586	0.001281	2.777	0.2264	3.441	0.2551	5.094	0.02212	2.68	0.03952
Val Leu Asp Glu	metab_5154	1.113	1.616E-5	4.891E-5	5.762	0.0253	5.505	0.03697	5.82	0.03875	4.582	0.2701	5.146	0.07476
Ala Glu Asn Arg	metab_30225	1.113	4.11E-5	6.288E-5	5.841	0.0355	3.992	0.02065	4.628	0.1166	5.877	0.03235	4.653	0.1263
Cyasterone	metab_7534	1.113	1.518E-5	4.891E-5	4.455	0.06296	5.134	0.07587	4.985	0.08277	5.619	0.02299	4.251	0.0912
3-Isopropylmalic Acid	metab_35983	1.112	1.421E-5	4.891E-5	3.643	0.07483	4.812	0.006945	3.481	0.06121	2.66	0.2676	4.431	0.01614
Momorcharaside A	metab_10801	1.112	1.325E-5	4.891E-5	6.424	0.0115	5.5	0.01659	6.038	0.009735	4.845	0.09934	5.913	0.01506
Ethylphosphate	metab_31995	1.112	2.075E-5	4.891E-5	4.705	0.01747	5.057	0.01193	4.128	0.08108	5.067	0.02931	4.816	0.0293
Ent-6R,16Boh,17-Trihydroxy-7-Oxo-6,7-Seco-19,6-Kauranolide 6-O-Glucoside	metab_33376	1.112	5.443E-5	7.614E-5	4.663	0.0885	4.298	0.06955	5.564	0.04415	4.844	0.06943	4.813	0.2089
Arg-Thr-Lys-Arg	metab_33547	1.111	1.518E-5	4.891E-5	3.184	0.2989	4.194	0.09512	5.506	0.03153	4.414	0.05714	5.14	0.0242
Mycophenolic Acid O-Acyl-Glucuronide	metab_39106	1.111	9.123E-5	0.0001152	5.31	0.0135	3.377	0.1611	3.509	0.2667	4.03	0.1056	3.229	0.1988
Gly-Pro-Arg	metab_25802	1.111	1.9E-5	4.891E-5	4.836	0.03563	4.531	0.04803	5.8	0.01158	5.536	0.0138	4.767	0.06397
Xanthosine	metab_49041	1.110	1.325E-5	4.891E-5	4.487	0.034	3.856	0.02202	4.751	0.01664	4.611	0.0229	3.781	0.05975
Asparaginyl-Proline	metab_38946	1.110	1.325E-5	4.891E-5	3.014	0.1227	4.262	0.06316	4.723	0.04813	3.887	0.1042	5.229	0.01236
Leu-Glu-Asp	metab_47019	1.110	1.9E-5	4.891E-5	3.499	0.003274	3.682	0.1363	4.83	0.01443	4.31	0.04906	4.922	0.02242
Armillane	metab_33910	1.110	1.325E-5	4.891E-5	4.25	0.04026	4.707	0.03638	6.227	0.008862	5.556	0.01345	5.519	0.006038
Isoquercetin	metab_38272	1.110	0.0002016	0.000228	4.753	0.133	3.555	0.5906	2.616	0.07623	2.924	0.5656	2.525	0.01018
Foliandrin	metab_37465	1.109	2.156E-5	4.891E-5	4.848	0.02271	4.834	0.02197	4.622	0.03894	2.428	0.864	5.112	0.01219
Rh-34	metab_46044	1.109	5.686E-5	7.861E-5	2.634	0.003268	2.731	0.1681	3.924	0.04704	4.908	0.01443	2.895	0.2535
Ser-Thr-Ile	metab_33314	1.109	2.535E-5	4.891E-5	2.442	0.003264	2.605	0.3067	3.946	0.04691	4.729	0.05296	3.418	0.05717
Pelargonidin 3-Rutinoside	metab_32067	1.109	8.444E-5	0.0001079	5.442	0.04113	5.354	0.03145	3.865	0.1716	4.136	0.1972	4.189	0.3307
D-Xylulose 5-Phosphate	metab_32547	1.109	1.325E-5	4.891E-5	4.472	0.03642	5.338	0.007647	5.824	0.02667	3.447	0.1285	5.447	0.02049
Asp His	metab_30647	1.109	1.807E-5	4.891E-5	3.269	0.2221	3.081	0.05289	4.605	0.02586	5.107	0.03224	3.992	0.06302
Niazidin	metab_7980	1.109	1.325E-5	4.891E-5	5.2	0.0192	3.122	0.02706	3.187	0.002697	4.348	0.02166	3.201	0.00326
2-(Acetylamino)-2-Deoxy-Alpha-D-Mannopyranose	metab_2793	1.108	0.004312	0.004409	2.313	0.2136	2.52	0.324	2.368	0.1336	4.551	0.6297	2.372	0.2687
Thr-Ala	metab_50368	1.108	1.325E-5	4.891E-5	3.376	0.02539	4.598	0.04977	5.442	0.02018	5.666	0.02181	5.135	0.03914
Citreoviridinol A1	metab_45003	1.108	1.325E-5	4.891E-5	3.03	0.03917	4.264	0.1043	4.934	0.02396	3.533	0.1963	5.285	0.0212
1,2,3,4-Tetrahydroisoquinoline-3-Carboxylic Acid	metab_48947	1.108	1.325E-5	4.891E-5	3.717	0.05344	4.287	0.01394	5.678	0.008185	4.917	0.009834	4.733	0.007264
Pro-Leu	metab_48390	1.107	1.325E-5	4.891E-5	4.222	0.02003	3.968	0.02401	5.435	0.01017	5.408	0.004736	5.046	0.008756
Isonicotinamide	metab_29132	1.105	1.325E-5	4.891E-5	4.004	0.1197	4.938	0.007975	5.088	0.01003	2.63	0.2641	4.916	0.005285
[2-(4-Propylphenyl)Quinolin-4-Yl]-(4-Pyridin-2-Ylpiperazin-1-Yl)Methanone	metab_22056	1.105	1.325E-5	4.891E-5	5.013	0.009756	5.248	0.01456	5.361	0.02577	5.6	0.02431	4.455	0.1314

Pa(Pgj2/I-13:0)	metab_45275	1.105	1.325E-5	4.891E-5	5.316	0.0066	5.115	0.03752	5.183	0.01543	3.244	0.1291	5.625	0.01266
Gln-Met	metab_2317	1.105	2.455E-5	4.891E-5	4.36	0.07798	3.519	0.0657	4.835	0.01448	6.001	0.02353	4.372	0.01154
Thr-Phe	metab_34816	1.105	1.325E-5	4.891E-5	4.293	0.02998	4.015	0.06101	5.53	0.006482	6.082	0.009576	4.862	0.01653
Alpha-Hederin	metab_37213	1.104	2.49E-5	4.891E-5	3.372	0.3022	4.232	0.05237	5.324	0.01074	3.321	0.1829	4.998	0.02786
(E)-3-Hydroxy Tamoxifen O-|A-D-Glucuronide	metab_33024	1.104	1.616E-5	4.891E-5	3.8	0.1223	3.094	0.2354	4.517	0.2038	5.039	0.2843	4.179	0.0466
Ala-Lys	metab_50853	1.104	1.616E-5	4.891E-5	3.484	0.08658	3.237	0.1999	4.426	0.01778	5.357	0.009795	4.264	0.02871
Selamectin	metab_48279	1.104	1.325E-5	4.891E-5	4.259	0.05745	4.931	0.02549	4.52	0.05234	2.242	0.9819	5.261	0.02338
Paramethasone	metab_45697	1.104	1.616E-5	4.891E-5	2.735	0.1076	3.15	0.2517	4.356	0.07801	5.163	0.01581	3.817	0.1365
25-Acetyl-6,7-Didehydrofevicordin F 3-[Glucosyl-(1->6)-Glucoside]	metab_44182	1.104	1.325E-5	4.891E-5	4.818	0.02445	4.512	0.06709	5.271	0.02072	3.511	0.3344	4.93	0.03916
Asn-Ile-Asp	metab_2761	1.103	1.518E-5	4.891E-5	3.493	0.1008	4.079	0.08503	5.117	0.02136	5.232	0.02342	5.149	0.01358
Beta-Funaltrexamine	metab_16065	1.103	2.455E-5	4.891E-5	3.565	0.2779	5.208	0.0435	5.093	0.03993	3.641	0.259	4.594	0.06943
Calystegine B2	metab_1278	1.103	4.55E-5	6.677E-5	4.282	0.1553	4.113	0.2467	5.451	0.01997	5.027	0.04991	4.432	0.127
Hordatine A	metab_3097	1.102	2.23E-5	4.891E-5	5.03	0.04523	4.445	0.1364	5.645	0.03924	5.085	0.04812	5.228	0.02651
N6-Methyl-2'-Deoxyadenosine	metab_30258	1.102	1.325E-5	4.891E-5	5.378	0.01248	5.192	0.02856	6.239	0.00963	5.058	0.02421	5.598	0.00755
Lappaol D	metab_6962	1.101	2.075E-5	4.891E-5	4.069	0.08312	4.589	0.1027	5.575	0.01323	5.217	0.03403	4.451	0.1805
Sl(11:0_O/18:4)	metab_43018	1.101	0.0005348	0.0005759	2.753	0.1432	2.695	0.1852	2.767	0.00676	4.809	0.02152	2.919	0.153
N-Formyl-Met-Leu-Phe	metab_49081	1.101	1.807E-5	4.891E-5	2.653	0.003268	2.577	0.001776	2.971	0.2816	5.007	0.04905	2.792	0.2501
8-(3-Hydroxybutyl)-1,5-Dimethyl-6-Oxabicyclo[3.2.1]Octan-3-Ol	metab_14613	1.101	0.003399	0.003486	4.933	0.01493	3.162	0.3356	3.278	0.3227	3.221	0.1725	3.408	0.1744
Galnon	metab_22869	1.101	2.807E-5	5.127E-5	3.555	0.522	5.021	0.04442	5.976	0.01238	5.194	0.0445	5.184	0.1014
2-[[3-Methyl-2-(Pyridine-3-Carbonylamino)Butanoyl]Amino]-3-Phenylpropanoic Acid	metab_19253	1.101	1.616E-5	4.891E-5	3.23	0.3461	3.68	0.1314	4.334	0.03319	5.103	0.0151	4.598	0.04331
Homogentisic Acid	metab_48845	1.100	2.53E-5	4.891E-5	4.264	0.02089	3.887	0.0445	5.003	0.1027	3.791	0.3073	4.633	0.03094
Val-Asp	metab_50299	1.100	1.616E-5	4.891E-5	3.332	0.25	4.597	0.02252	5.416	0.0547	5.513	0.03857	5.188	0.05125
Hydratopyrrhoxanthinol	metab_11689	1.100	1.325E-5	4.891E-5	4.448	0.01444	5.253	0.03171	5.974	0.006617	3.656	0.1954	5.676	0.008476
2-(3-Hexyl-4-Methyl-2,5-Dioxopyrrol-1-Yl)-3-Hydroxybutanoic Acid	metab_42932	1.099	0.0001011	0.0001255	4.982	0.008364	2.945	0.1525	2.861	0.006762	4.157	0.02884	2.977	0.1465
13:4+4O Fatty Acyl Hexoside	metab_33110	1.099	0.002508	0.002586	0.3234	0.001719	2.005	1.35	3.524	0.9687	2.113	1.518	3.3	1.624
Tranexamic Acid	metab_38113	1.099	1.325E-5	4.891E-5	4.702	0.0161	4.63	0.01069	6.16	0.01025	6.05	0.006058	5.577	0.009268
Absinthin	metab_47148	1.099	1.712E-5	4.891E-5	4.811	0.04612	4.566	0.07642	5.458	0.008404	5.212	0.06257	4.437	0.03984
Glu-Gly-Asp	metab_32564	1.099	2.359E-5	4.891E-5	2.705	0.1719	3.768	0.07694	4.747	0.02632	4.642	0.04901	4.625	0.05308
2-Acetamido-3-(4-Acetamidophenyl)Prop-2-Enoic Acid	metab_35252	1.098	1.325E-5	4.891E-5	4.168	0.02558	3.72	0.03074	4.743	0.01763	3.88	0.05257	4.665	0.0145
Ile-Ile-Ile-Pro	metab_12915	1.098	1.325E-5	4.891E-5	6.269	0.004407	5.145	0.03949	5.012	0.01818	5.273	0.02232	5.773	0.0125
5,6-Dimethoxyindan-1-One	metab_5957	1.098	2.455E-5	4.891E-5	4.723	0.04213	4.631	0.03628	3.906	0.1655	5.968	0.009689	3.931	0.1833
Isopetasoside	metab_39619	1.097	1.325E-5	4.891E-5	3.435	0.1472	5.349	0.01823	5.474	0.01585	4.658	0.03662	5.15	0.01604
Tryptophyl-Asparagine	metab_35692	1.097	1.325E-5	4.891E-5	2.483	0.0566	3.241	0.2875	4.249	0.02611	4.864	0.01227	3.913	0.06672
Ziritaxestat	metab_24723	1.097	1.325E-5	4.891E-5	5.235	0.01283	5.952	0.0155	6.104	0.0127	3.795	0.7023	5.7	0.01711
Nelfinavir	metab_42919	1.097	2.335E-5	4.891E-5	3.387	0.2894	5.08	0.0232	4.438	0.0613	3.841	0.2121	4.357	0.03366

Dg(Pgj2/I-12:0/0:0)	metab_39880	1.097	1.421E-5	4.891E-5	5.695	0.01483	5.252	0.01892	4.328	0.1103	5.489	0.01421	4.052	0.09903
10-Gingerol	metab_4538	1.096	2.49E-5	4.891E-5	5.137	0.02225	4.497	0.04501	3.573	0.2221	3.461	0.006603	4.687	0.03779
Trp-Met-His	metab_29681	1.096	5.8E-5	7.997E-5	2.85	0.3667	2.567	0.3867	3.965	0.3181	5.07	0.01732	3.383	0.4884
Dg(18:2/18:3)	metab_15178	1.096	1.874E-5	4.891E-5	4.163	0.3122	5.401	0.03326	5.031	0.07969	3.61	0.2088	4.577	0.1219
N-Acetyl-9-Aminominocycline, (4R)-	metab_45071	1.096	3.034E-5	5.345E-5	3.802	0.06414	3.563	0.1335	4.335	0.02417	4.345	0.04477	4.67	0.02198
Pro-Tyr-Arg	metab_26406	1.096	1.325E-5	4.891E-5	5.55	0.02262	5.758	0.007911	5.924	0.01074	6.523	0.009452	4.955	0.05548
Ethyl Cinnamate	metab_22387	1.096	2.164E-5	4.891E-5	4.106	0.02823	3.175	0.3222	2.571	0.01166	4.949	0.01169	2.859	0.1674
Glu-His-Thr	metab_46338	1.095	2.53E-5	4.891E-5	3.285	0.08863	3.326	0.2251	4.712	0.0075	5.077	0.01725	4.354	0.01486
Proline Betaine	metab_45991	1.095	1.325E-5	4.891E-5	5.406	0.008719	4.793	0.003488	6.142	0.009503	5.948	0.004426	5.644	0.007278
Tyrosylalanine	metab_49430	1.095	2.317E-5	4.891E-5	3.258	0.1154	3.378	0.09295	4.541	0.05823	5.198	0.04913	4.389	0.1092
Ser-Ile-Lys	metab_2779	1.095	5.988E-5	8.196E-5	4.128	0.02681	4.345	0.05006	4.513	0.2266	5.08	0.2718	3.469	0.4364
Fluocortin	metab_37546	1.095	1.518E-5	4.891E-5	3.577	0.003275	4.139	0.08804	5.404	0.01869	3.931	0.1159	4.716	0.03121
Asp Glu Phe	metab_7182	1.094	1.325E-5	4.891E-5	4.578	0.05595	3.882	0.1395	5.033	0.02094	6.097	0.007829	4.882	0.04158
Phlorizin	metab_33403	1.094	1.325E-5	4.891E-5	5.1	0.008054	5.256	0.01638	5.357	0.03	5.551	0.0309	4.488	0.02314
Trp Trp	metab_12320	1.094	9.876E-5	0.000123	2.942	0.2641	2.757	0.001856	2.835	0.004921	5.001	0.0105	2.847	0.003257
2-{2-Oxo-2-[4-(1H-Pyrrol-1-Yl)Piperidino]Ethoxy}Acetic Acid	metab_5733	1.093	1.325E-5	4.891E-5	4.855	0.05469	4.528	0.0276	6.026	0.01007	6.527	0.005344	5.349	0.02118
Fevicordin B 2-Gentiobioside	metab_9532	1.093	7.511E-5	9.767E-5	5.913	0.03512	3.948	0.2652	4.029	0.1761	4.994	0.08699	4.299	0.2582
Deoxyadenosine Monophosphate	metab_33518	1.093	1.325E-5	4.891E-5	2.872	0.1696	4.684	0.02686	4.976	0.01237	3.534	0.1756	4.495	0.02903
Val-Phe-Leu	metab_38646	1.093	1.325E-5	4.891E-5	5.16	0.005617	3.928	0.041	4.417	0.02425	4.53	0.01225	4.706	0.01585
Calophymembranside B	metab_33589	1.093	1.807E-5	4.891E-5	5.026	0.0198	4.916	0.02357	4.605	0.07857	2.68	0.003713	4.982	0.03153
Glutamylphenylalanine	metab_48689	1.092	1.325E-5	4.891E-5	3.919	0.07195	4.112	0.02881	5.185	0.01509	6.135	0.007813	4.936	0.008759
Trp-Asp	metab_48080	1.092	1.325E-5	4.891E-5	3.125	0.1927	4.263	0.005013	4.756	0.01685	5.437	0.009077	4.18	0.02004
Lpc(18:5-Sn2)	metab_28235	1.091	1.325E-5	4.891E-5	5.013	0.02176	4.896	0.03087	5.896	0.08282	6.719	0.02439	4.73	0.08209
Plazomicin	metab_43653	1.091	2.156E-5	4.891E-5	4.203	0.05597	5.014	0.01687	4.417	0.02871	5.056	0.01375	4.998	0.01638
Phe Asn	metab_25953	1.091	1.325E-5	4.891E-5	4.655	0.05322	4.436	0.05071	5.851	0.006632	6.219	0.007882	5.454	0.01156
Gly-His	metab_31500	1.091	1.325E-5	4.891E-5	3.299	0.104	2.535	0.00117	3.906	0.02303	4.91	0.005241	3.081	0.09134
Cucurbitacin I 2-Glucoside	metab_44712	1.090	3.28E-5	5.556E-5	4.031	0.05094	3.907	0.174	3.211	0.175	5.266	0.01363	3.56	0.2087
Dihydroouabain	metab_37441	1.089	1.527E-5	4.891E-5	5.498	0.00888	4.354	0.04947	3.95	0.06797	6.304	0.008019	4.183	0.08846
Formacidine	metab_26144	1.089	1.325E-5	4.891E-5	4.011	0.1302	5.668	0.01932	6.168	0.01567	5.821	0.03968	6.006	0.01194
Prostaglandin Pge2 Glyceryl Ester	metab_44907	1.089	0.0001461	0.0001711	3.834	0.04409	2.893	0.1898	2.887	0.006762	5.291	0.01135	2.887	0.06953
Fexaramine	metab_47858	1.088	1.325E-5	4.891E-5	3.401	0.1339	4.329	0.01344	5.48	0.01571	4.905	0.01956	4.541	0.02849
Dgmg(18:3)	metab_40161	1.088	2.402E-5	4.891E-5	5.887	0.0171	4.953	0.107	4.751	0.04206	3.589	0.5247	4.718	0.02792
Homostachydrine	metab_26612	1.087	0.0001061	0.0001307	3.46	0.1073	3.044	0.2268	2.952	0.002696	5.258	0.01177	2.965	0.003258
Pro Trp His	metab_9993	1.087	1.712E-5	4.891E-5	5.434	0.01612	4.663	0.05996	5.128	0.02382	5.455	0.009433	4.14	0.12
Imazapyr	metab_21344	1.087	1.712E-5	4.891E-5	3.424	0.2808	2.76	0.001856	3.882	0.08443	5.365	0.01256	3.026	0.235

Cytidine 3'-Phosphate	metab_32017	1.087	1.9E-5	4.891E-5	4.319	0.06055	4.755	0.04169	5.883	0.03708	4.391	0.003721	5.151	0.04315
Asn-Ile	metab_48678	1.086	1.99E-5	4.891E-5	4.695	0.03846	4.664	0.01465	5.867	0.006365	6.344	0.005641	5.733	0.008684
Ile-Lys	metab_50319	1.086	0.0001965	0.0002226	3.709	0.003275	3.946	0.2242	5.057	0.07358	5.088	0.1151	5.098	0.0448
(4E,7E,10E,13E)-Hexadeca-4,7,10,13-Tetraenoic Acid	metab_20858	1.086	9.492E-5	0.000119	3.919	0.07491	3.174	0.2458	3.137	0.2456	5.54	0.01238	2.916	0.1568
N-(1-Deoxy-1-Fructosyl)Leucine	metab_49402	1.086	1.325E-5	4.891E-5	4.662	0.02486	4.592	0.01482	5.974	0.06614	5.48	0.05882	5.095	0.04777
Aclacinomycin N	metab_38994	1.086	2.535E-5	4.891E-5	3.472	0.2644	5.224	0.0186	5.419	0.0166	3.496	0.246	5.606	0.01863
Glycinoeclepin A	metab_13607	1.086	2.359E-5	4.891E-5	5.511	0.02159	4.223	0.1576	4.163	0.1266	4.52	0.0638	4.839	0.03882
Cyproteron	metab_46858	1.086	6.909E-5	9.185E-5	3.086	0.317	2.614	0.00117	2.762	0.00676	5.017	0.01331	2.729	0.01019
Arginylserine	metab_31525	1.085	1.807E-5	4.891E-5	3.717	0.07613	3.616	0.0649	4.668	0.03908	5.627	0.02299	4.568	0.0247
Ser Val Asn Glu	metab_49814	1.085	2.23E-5	4.891E-5	3.225	0.003273	3.148	0.001172	4.149	0.07532	5.134	0.08812	4.102	0.1765
1-Methyl-4-Piperidyl Acetate	metab_32899	1.085	0.0001453	0.0001704	3.933	0.01585	3.952	0.04104	5.323	0.2778	4.67	0.2361	4.322	0.3829
Ogyline	metab_33345	1.085	1.325E-5	4.891E-5	3.781	0.2579	4.634	0.02378	5.92	0.003054	5.595	0.02888	5.428	0.014
Leu-Lys-His	metab_30206	1.085	1.9E-5	4.891E-5	5.11	0.02558	3.989	0.1855	4.903	0.04726	5.867	0.01862	4.961	0.04754
Sphinganine	metab_16833	1.085	1.325E-5	4.891E-5	7.785	0.03322	7.331	0.01175	6.133	0.08376	7.269	0.009994	6.992	0.01543
Galactosylhydroxylysine	metab_48688	1.085	1.325E-5	4.891E-5	3.776	0.04997	4.249	0.0139	5.131	0.04405	5.35	0.08149	4.016	0.06331
2-[1-(2-Oxo-2-{[3-(Trifluoromethyl)Benzyl]Amino}Ethyl)Cyclohexyl]Acetic Acid	metab_34272	1.084	1.325E-5	4.891E-5	3.324	0.1386	4.41	0.02788	5.34	0.01153	5.089	0.01008	5.266	0.009997
Vpgpr Enterostatin	metab_39588	1.083	2.359E-5	4.891E-5	4.899	0.03081	4.916	0.02518	5.317	0.01367	5.046	0.01456	4.467	0.03995
Propafenone Glucuronide	metab_17763	1.083	2.378E-5	4.891E-5	5.158	0.03211	4.311	0.1396	3.505	0.3192	5.211	0.02429	3.266	0.2557
4-Hydroxyisoleucine	metab_2013	1.083	1.421E-5	4.891E-5	4.709	0.01705	5.322	0.005927	5.302	0.01028	5.551	0.009007	4.549	0.01654
Adenosine Phosphate	metab_2591	1.082	1.325E-5	4.891E-5	5.862	0.01381	6.711	0.01205	6.919	0.01397	4.78	0.0617	6.498	0.005386
Lys Ser Thr Leu Thr	metab_28431	1.082	1.9E-5	4.891E-5	4.574	0.001284	4.704	0.09482	6.087	0.03812	5.129	0.09417	5.503	0.04557
1-[6-(2-Carboxypyrrolidin-1-Yl)-6-Oxohexanoyl]Pyrrolidine-2-Carboxylic Acid	metab_34967	1.082	1.325E-5	4.891E-5	3.073	0.1288	3.943	0.07945	5.139	0.0131	4.644	0.03411	4.883	0.0158
Ps(Pgf2Alpha/18:2(9Z,12Z))	metab_37292	1.082	1.99E-5	4.891E-5	5.433	0.01955	4.418	0.04888	5.085	0.01875	4.555	0.003721	5.066	0.01771
Gln Gly	metab_659	1.082	1.325E-5	4.891E-5	3.731	0.3182	4.252	0.1352	5.293	0.01904	5.895	0.007534	5.099	0.03573
2,5-Anhydro-D-Mannose	metab_49193	1.082	1.99E-5	4.891E-5	3.338	0.06679	4.651	0.009197	4.153	0.03681	2.051	0.1348	4.626	0.0383
4'-O-Methylhydrangenol	metab_39648	1.082	0.0002036	0.00023	5.37	0.00739	3.763	0.04979	3.766	0.07091	3.643	0.1036	3.815	0.05006
Candoxatrilat	metab_33359	1.081	2.904E-5	5.21E-5	3.266	0.2542	5.095	0.01976	4.856	0.05211	3.182	0.003719	4.726	0.04035
Gln-Ile-Leu	metab_45501	1.081	4.005E-5	6.188E-5	2.999	0.1978	3.664	0.06754	3.342	0.2087	4.869	0.01428	3.428	0.109
Ps(Pgf1Alpha/22:5(7Z,10Z,13Z,16Z,19Z))	metab_45515	1.081	1.807E-5	4.891E-5	5.059	0.01972	4.781	0.02116	4.814	0.01784	3.25	0.08	5.447	0.01378
Asn Leu	metab_27556	1.081	2.075E-5	4.891E-5	4.999	0.02868	4.972	0.03098	6.193	0.01698	6.56	0.007261	6.046	0.02109
Sabeluzole	metab_36552	1.080	1.807E-5	4.891E-5	4.536	0.05713	5.608	0.01588	4.409	0.09951	3.224	0.1471	4.82	0.03171
Succinamic Acid	metab_31823	1.080	1.325E-5	4.891E-5	4.696	0.02039	2.765	0.001171	2.997	0.1789	3.745	0.1343	2.88	0.01019
7-Methylguanosine	metab_33144	1.080	1.518E-5	4.891E-5	4.079	0.06672	4.193	0.0247	5.482	0.01381	5.924	0.007879	5.155	0.0227
Lys-Gly-Tyr	metab_20730	1.079	0.0008944	0.0009452	3.669	0.2545	3.609	0.1832	3.45	0.1771	5.423	0.0116	3.75	0.07316
Pro-Tyr-Gln	metab_4394	1.079	0.0001048	0.0001293	4.529	0.1064	4.59	0.08018	5.6	0.01307	6.338	0.00877	4.482	0.1063

Chenodeoxycholylaspartic Acid	metab_45155	1.079	0.000645	0.0006893	3.734	0.09646	3.675	0.1723	3.463	0.006769	5.482	0.007368	3.709	0.1988
Arg-Met-Tyr	metab_46441	1.078	2.515E-5	4.891E-5	4.619	0.0227	3.832	0.0381	3.062	0.3104	3.811	0.08427	4.061	0.02331
Tryptophyl-Serine	metab_33889	1.078	4.118E-5	6.295E-5	2.932	0.05599	3.18	0.07359	4.1	0.04392	4.981	0.01516	3.065	0.1848
4-[(1R,2S)-3-(4-Benzylpiperidin-1-Yl)-1-Hydroxy-2-Methylpropyl]Phenol	metab_17982	1.078	2.156E-5	4.891E-5	5.068	0.01567	4.81	0.02105	4.046	0.09749	6.106	0.008569	4.208	0.2066
Shexcer(32:4_3O)	metab_37171	1.078	3.353E-5	5.632E-5	5.335	0.01123	5.047	0.02207	5.3	0.01613	3.43	0.1243	5.327	0.01276
Allyl Cysteine	metab_46685	1.078	1.325E-5	4.891E-5	2.877	0.2656	3.545	0.04316	4.71	0.007688	5.129	0.003515	4.115	0.01767
Dgdg(O-18:5/5:0)	metab_33635	1.078	1.325E-5	4.891E-5	4.885	0.0223	4.493	0.03356	5.017	0.02733	3.569	0.0799	5.325	0.01749
Pi(17:2/18:5)	metab_37641	1.078	1.712E-5	4.891E-5	5.166	0.05344	4.798	0.02589	5.298	0.02312	3.526	0.2858	5.263	0.01544
Gamma-Glutamylglutamine	metab_50367	1.077	1.9E-5	4.891E-5	3.563	0.003275	3.516	0.07202	4.746	0.04711	5.698	0.03392	4.26	0.1531
Pi(Pgf1Alpha/18:1(9Z))	metab_37280	1.077	1.325E-5	4.891E-5	5.917	0.01553	5.489	0.007073	5.691	0.008825	4.06	0.1684	6.075	0.01304
N-Acetyl-L-Alanine	metab_32777	1.077	1.325E-5	4.891E-5	4.56	0.01014	4.673	0.00771	3.903	0.03727	5.604	0.05027	4.308	0.04159
Chymostatin	metab_35513	1.077	1.325E-5	4.891E-5	2.782	1.02	4.483	0.04625	5.315	0.01873	4.034	0.1309	5.176	0.007466
Gly-Ala-Gln	metab_50447	1.077	8.511E-5	0.0001086	3.859	0.3372	3.545	0.08819	4.568	0.05516	5.976	0.01599	3.68	0.1002
Dermatan L-Iduronate	metab_48247	1.077	1.421E-5	4.891E-5	5.066	0.007368	4.53	0.06032	4.987	0.03781	3.904	0.2214	4.432	0.02357
Phe-Thr-Ala	metab_7765	1.077	6.293E-5	8.542E-5	4.29	0.05731	3.599	0.2597	3.264	0.1651	5.569	0.01258	3.534	0.2141
Karanjachromene	metab_19159	1.076	1.325E-5	4.891E-5	2.903	0.001282	2.892	0.001857	2.969	0.002696	4.952	0.01394	2.982	0.003258
Taurodeoxycholic Acid	metab_37474	1.076	1.325E-5	4.891E-5	5.838	0.001989	4.817	0.03105	5.202	0.0105	5.157	0.01614	5.586	0.004106
(S)-A-Amino-2,5-Dihydro-5-Oxo-4-Isoxazolepropanoic Acid N2-Glucoside	metab_33206	1.076	1.807E-5	4.891E-5	3.625	0.1242	3.948	0.03391	5.026	0.0962	5.323	0.06526	4.989	0.1197
Gly Val Leu	metab_22844	1.075	1.325E-5	4.891E-5	3.939	0.369	4.374	0.07441	5.53	0.017	6.244	0.008357	5.03	0.03943
Vomifoliol 9-[Glucosyl-(1->4)-Xylosyl-(1->6)-Glucoside]	metab_47625	1.075	1.325E-5	4.891E-5	3.427	0.2545	4.916	0.05305	5.48	0.0119	5.392	0.02065	5.274	0.02383
Phe-Phe-Ser	metab_43931	1.075	1.325E-5	4.891E-5	2.662	0.06319	3.692	0.07791	4.025	0.04329	4.645	0.02626	4.175	0.01425
Leu-Gly-Asp	metab_32640	1.075	2.591E-5	4.929E-5	3.263	0.003274	3.312	0.0847	4.453	0.1194	5.03	0.08684	4.332	0.1749
Asp-Asn	metab_50468	1.075	1.524E-5	4.891E-5	4.064	0.1401	3.754	0.1001	4.895	0.04782	5.64	0.01622	4.786	0.04712
Pyridoxal	metab_35791	1.075	0.00054	0.0005811	4.247	0.2236	3.841	0.4708	2.697	0.383	2.823	0.6918	2.546	0.2572
Capsicoside C3	metab_33960	1.075	1.325E-5	4.891E-5	5.493	0.005141	4.933	0.02613	4.595	0.05308	3.199	0.4044	5.222	0.01877
Glu-Val-Tyr	metab_45854	1.074	1.325E-5	4.891E-5	3.91	0.1041	4.305	0.02715	5.601	0.008102	5.028	0.01455	5.375	0.007133
Gentamicin C	metab_34890	1.074	2.455E-5	4.891E-5	3.154	0.003273	4.271	0.07558	4.318	0.04621	3.651	0.2621	5.056	0.02903
Amphibine H	metab_40257	1.074	2.818E-5	5.132E-5	3.17	0.3095	3.834	0.2084	4.922	0.01138	3.065	0.003718	4.537	0.03491
Asn Tyr Glu	metab_2625	1.074	3.567E-5	5.816E-5	4.245	0.2074	4.105	0.3714	4.843	0.3033	5.65	0.2938	5.177	0.07162
Mimosine	metab_35717	1.074	1.518E-5	4.891E-5	3.611	0.1026	3.275	0.1552	4.58	0.01655	4.998	0.01879	4.321	0.05262
Val-Met	metab_33496	1.074	1.9E-5	4.891E-5	3.054	0.1774	3.326	0.159	4.574	0.01959	5.081	0.01101	4.192	0.01863
17-O-Deacetylvindoline	metab_20795	1.074	2.075E-5	4.891E-5	3.988	0.2275	4.607	0.06604	5.853	0.01311	4.924	0.03508	4.959	0.02321
Gibberellin A36	metab_48280	1.074	2.075E-5	4.891E-5	4.785	0.02975	4.983	0.02821	4.737	0.04429	2.89	0.6111	5.51	0.009884
1-Monolinolenoyl-Rac-Glycerol	metab_14579	1.074	4.037E-5	6.213E-5	6.089	0.01863	4.677	0.03926	4.536	0.06225	4.58	0.07484	4.211	0.1476
Asn-Leu-Tyr	metab_46263	1.074	1.518E-5	4.891E-5	3.293	0.07652	3.746	0.05813	4.947	0.01422	4.835	0.02691	4.79	0.01438

Euscaphic Acid	metab_40852	1.074	1.325E-5	4.891E-5	4.868	0.0441	3.047	0.001172	3.195	0.006767	3.61	0.2124	3.162	0.0102
Lys-Lys	metab_51054	1.073	3.287E-5	5.561E-5	2.434	0.1784	2.781	0.2601	3.444	0.08622	4.662	0.02331	3.419	0.06321
Ptelatoside B	metab_35143	1.073	1.325E-5	4.891E-5	3.246	0.1614	4.708	0.04828	5.348	0.02724	4.604	0.0207	5.421	0.02265
Nemifitide	metab_37185	1.073	1.325E-5	4.891E-5	4.135	0.08009	4.687	0.03049	4.802	0.02678	2.509	0.6715	5.188	0.01626
Acetoacetanilide	metab_8696	1.072	2.702E-5	5.054E-5	4.639	0.01542	3.37	0.3812	2.496	0.05291	3.743	0.07116	2.476	0.001867
Ps(Pgf2Alpha/22:5(7Z,10Z,13Z,16Z,19Z))	metab_44590	1.072	1.325E-5	4.891E-5	3.417	0.003274	4.902	0.02416	5.654	0.01629	4.564	0.05048	5.592	0.005918
Dihydroisocarveol	metab_42696	1.072	1.325E-5	4.891E-5	5.461	0.00557	4.87	0.01314	5.93	0.006743	5.38	0.005585	5.521	0.01
Ala-Asn-Ser	metab_31888	1.072	1.616E-5	4.891E-5	3.493	0.2869	3.205	0.001172	4.619	0.08002	5.356	0.02531	4.007	0.3523
Lysylmethionine	metab_33639	1.072	2.075E-5	4.891E-5	3.963	0.02462	3.934	0.02879	5.016	0.01397	5.127	0.02235	5.053	0.0117
7,8-Dihydro-3B,6A-Dihydroxy-Alpha-Ionol 9-[Apiosyl-(1->6)-Glucoside]	metab_4067	1.071	1.325E-5	4.891E-5	3.936	0.6017	5.36	0.03693	6.346	0.01173	5.23	0.08786	5.672	0.03016
Pi(O-11:0/22:6)	metab_46668	1.071	1.325E-5	4.891E-5	5.447	0.01088	5.052	0.03262	5.525	0.02557	3.998	0.09254	5.802	0.007622
Gly-Thr-Phe	metab_46875	1.071	1.325E-5	4.891E-5	2.824	0.2193	3.457	0.04932	4.509	0.01878	5.002	0.01204	4.193	0.05078
2-Cis-Abscisate	metab_35033	1.071	1.325E-5	4.891E-5	4.817	0.03678	4.565	0.01797	5.85	0.01154	6.428	0.01043	5.55	0.0109
Ile-Ser	metab_32625	1.071	1.421E-5	4.891E-5	4.485	0.02331	4.426	0.02245	5.76	0.01859	6.179	0.005999	5.382	0.02796
2-Keto-3-Deoxy-D-Gluconic Acid	metab_50419	1.071	1.518E-5	4.891E-5	5.134	0.02513	5.507	0.01466	4.736	0.04175	4.628	0.05785	4.471	0.03935
Goshonoside F4	metab_43744	1.071	1.99E-5	4.891E-5	5.023	0.02337	3.425	0.3353	3.794	0.1616	5.1	0.01339	4.095	0.07121
Ser-Ser-Trp	metab_35819	1.071	1.99E-5	4.891E-5	2.719	0.2265	3.945	0.09092	4.327	0.02465	4.811	0.0184	4.29	0.02963
Phenylpyruvic Acid	metab_39627	1.070	1.325E-5	4.891E-5	3.793	0.04237	4.736	0.06344	3.524	0.07761	2.321	0.06426	4.042	0.01644
2-Acetoxy-3-Geranylgeranyl-1,4-Dihydroxybenzene	metab_40605	1.070	0.000123	0.0001479	4.37	0.1746	4.179	0.118	5.315	0.06824	4.368	0.2625	5.218	0.04052
Ala-Met-Ser	metab_33207	1.070	1.325E-5	4.891E-5	4.041	0.02844	4.176	0.02389	5.347	0.01367	4.633	0.1005	5.295	0.02247
Leucyl-Leucine	metab_36066	1.070	1.325E-5	4.891E-5	3.901	0.04659	4.346	0.03619	5.614	0.01006	5.588	0.0098	5.306	0.007555
Divanillyltetrahydrofuran Ferulate	metab_37455	1.070	2.455E-5	4.891E-5	3.812	0.003275	3.735	0.001173	4.791	0.01468	4.023	0.06168	3.975	0.09633
3A,6B,7A,12A-Tetrahydroxy-5B-Cholanoic Acid	metab_16218	1.070	1.325E-5	4.891E-5	5.684	0.05998	4.736	0.1039	3.668	0.002698	5.375	0.01984	3.987	0.2335
Azaspiracid	metab_39249	1.070	2.359E-5	4.891E-5	3.519	0.2352	4.682	0.08455	5.436	0.03579	3.408	0.00372	5.307	0.02158
Glu Pro Ile	metab_20064	1.070	2.455E-5	4.891E-5	5.019	0.01815	5.264	0.01134	4.343	0.07026	5.523	0.009237	4.315	0.05056
Lpe(22:6)	metab_42072	1.069	0.0009499	0.001002	4.822	0.2626	4.412	0.7519	3.262	0.3134	3.811	0.6742	3.041	0.04687
Humilixanthin	metab_49112	1.069	1.807E-5	4.891E-5	3.664	0.05412	4.063	0.04186	5.097	0.06653	5.448	0.03185	5.04	0.09136
Amylose	metab_32979	1.069	1.325E-5	4.891E-5	5.859	0.007025	5.528	0.008185	4.551	0.01947	6.369	0.01104	4.944	0.03672
(E)-7-(4-Hydroxy-3-Methoxyphenyl)-1-Phenylhept-4-En-3-One	metab_2924	1.069	1.524E-5	4.891E-5	2.796	0.2218	4.466	0.06706	5.083	0.01412	3.601	0.3591	4.61	0.06278
Pro-Ile	metab_27029	1.069	1.616E-5	4.891E-5	5.219	0.02483	5.044	0.02681	6.474	0.00562	6.49	0.01044	5.977	0.009585
Ile-Leu-Leu	metab_10059	1.068	2.354E-5	4.891E-5	5.148	0.02375	3.945	0.1519	3.798	0.1456	3.119	0.2188	4.235	0.09367
Kanokoside C	metab_33369	1.068	0.0001497	0.0001747	5.19	0.006615	3.272	0.07191	3.274	0.188	4.749	0.04117	3.328	0.1121
Prolyl-Phenylalanyl-Arginine-4-Nitroanilide	metab_45498	1.068	2.535E-5	4.891E-5	3.528	0.003274	3.548	0.1034	4.995	0.01327	4.605	0.02483	4.289	0.04642
10-Deacetyltaxol	metab_46690	1.068	2.298E-5	4.891E-5	4.06	0.1349	4.489	0.05456	5.603	0.0153	4.18	0.1509	5.506	0.01527
Asi-222	metab_24992	1.067	3.455E-5	5.698E-5	5.556	0.02483	5.559	0.01917	5.505	0.02156	3.659	0.006603	6.048	0.009971

2,5-Dibenzyl-3-Hydroxy-6-Methoxypyrazine	metab_49065	1.067	1.325E-5	4.891E-5	4.548	0.02298	4.354	0.01805	5.71	0.09311	6.325	0.02893	4.796	0.07656
3-Dehydroteasterone	metab_41536	1.067	1.325E-5	4.891E-5	5.479	0.03621	3.787	0.001173	3.936	0.006771	4.098	0.07124	3.903	0.01021
Nefiracetam	metab_19565	1.067	1.325E-5	4.891E-5	3.435	0.2028	4.062	0.06306	4.72	0.0344	5.584	0.007746	3.86	0.07092
Asparagine Ethyl Ester	metab_4106	1.067	0.002733	0.002814	3.229	0.3612	2.834	0.1501	2.849	0.1027	5.07	0.01281	2.821	0.003257
Apricoxib	metab_48941	1.067	9.087E-5	0.0001148	4.268	0.06722	4.401	0.0467	3.472	0.1785	3.491	0.3195	3.241	0.1686
Clozapine N-Oxide	metab_8927	1.067	1.325E-5	4.891E-5	5.916	0.006258	5.748	0.002981	4.43	0.03636	5.039	0.0259	5.301	0.0089
Pro Gly Phe	metab_23948	1.066	2.535E-5	4.891E-5	4.047	0.1128	4.01	0.1612	5.446	0.0145	5.775	0.009266	4.679	0.06586
Gly Ala Phe	metab_24615	1.066	2.298E-5	4.891E-5	3.967	0.1185	3.863	0.1163	5.109	0.01217	5.874	0.004125	4.731	0.0156
[2,3-Dihydroxy-3-(3-Methoxy-5-Oxo-2H-Furan-2-Yl)-1-Phenylpropyl] Acetate	metab_50175	1.065	1.325E-5	4.891E-5	4.754	0.01478	5.114	0.009074	5.495	0.0173	3.513	0.3042	5.037	0.02219
Pro Ile Trp	metab_12286	1.065	2.858E-5	5.164E-5	3.26	0.3526	3.869	0.1268	3.717	0.1084	4.945	0.01188	4.113	0.04507
Bisoprolol	metab_11135	1.065	0.000108	0.0001326	4.002	0.1065	4.529	0.02547	3.915	0.1112	5.352	0.01464	3.896	0.1692
Lys-His-His	metab_36671	1.065	1.325E-5	4.891E-5	3.984	0.04912	3.08	0.001172	4.216	0.03333	5.487	0.01135	3.385	0.1491
D-3-Phenyllactic Acid	metab_37946	1.065	1.325E-5	4.891E-5	6.381	0.002157	6.577	0.001468	5.627	0.008218	6.137	0.005836	6.48	0.004535
Asn-Phe	metab_47795	1.065	1.325E-5	4.891E-5	4.28	0.02938	4.092	0.02636	5.405	0.008853	5.805	0.005494	5.077	0.006932
Erythromycylamine	metab_45243	1.065	1.712E-5	4.891E-5	5.597	0.008496	5.699	0.009357	5.682	0.01518	3.824	0.1446	6.254	0.01256
5-Methyltetrahydrofolic Acid	metab_47658	1.064	2.23E-5	4.891E-5	4.321	0.05043	4.279	0.06754	5.569	0.01329	4.892	0.01579	5.264	0.008846
Ser-Trp-Ile	metab_44019	1.064	1.325E-5	4.891E-5	2.71	0.1534	3.841	0.05227	4.557	0.02395	4.9	0.01512	4.23	0.03015
N-Hydroxycadaverine	metab_29224	1.064	2.298E-5	4.891E-5	4.837	0.01971	4.224	0.03088	4.26	0.05289	2.768	0.3525	4.616	0.02415
Ribostamycin	metab_43575	1.063	2.881E-5	5.187E-5	3.676	0.1666	4.922	0.01538	4.174	0.04817	3.778	0.1541	4.087	0.06197
Val-Asn-Ser	metab_49436	1.063	2.359E-5	4.891E-5	3.532	0.073	3.483	0.0921	4.691	0.06183	5.336	0.1091	4.422	0.05401
Anamorelin	metab_24222	1.063	1.325E-5	4.891E-5	3.911	0.2251	4.518	0.1245	5.793	0.02797	5.599	0.01247	5.407	0.04149
Equol	metab_37576	1.062	3.772E-5	6.013E-5	3.846	0.0361	4.66	0.0141	3.853	0.00677	3.975	0.04544	4.646	0.009879
Asn-Gly-Lys	metab_31464	1.062	1.616E-5	4.891E-5	2.72	0.003269	2.643	0.00117	3.539	0.2302	5.028	0.01805	3.026	0.2986
Trp Ile	metab_10707	1.062	1.325E-5	4.891E-5	3.918	0.07941	5.043	0.01694	5.732	0.006123	6.114	0.004318	5.377	0.01478
Sodium Lauryl Sulfate	metab_2362	1.062	1.325E-5	4.891E-5	3.626	0.127	4.484	0.03558	5.075	0.01046	5.919	0.0202	4.853	0.0268
Gly-Val	metab_49536	1.062	2.576E-5	4.929E-5	3.519	0.04072	3.378	0.04311	4.803	0.07617	4.606	0.2188	4.314	0.1
Blumenol C O-[Rhamnosyl-(1->6)-Glucoside]	metab_37420	1.062	3.902E-5	6.072E-5	3.192	0.2067	2.739	0.001171	2.887	0.006762	5.03	0.01262	2.854	0.01019
Dhghab	metab_32039	1.062	1.9E-5	4.891E-5	3.342	0.2214	4.712	0.06415	5.508	0.05371	3.693	0.4673	5.335	0.07101
Lysyl-Aspartyl-Glutamyl-Leucine	metab_46725	1.062	1.325E-5	4.891E-5	5.216	0.02099	4.717	0.03536	4.441	0.04732	6.376	0.005237	4.908	0.01643
His-Arg-Arg	metab_5651	1.061	2.217E-5	4.891E-5	3.559	0.001284	4.02	0.3905	5.415	0.04188	4.729	0.1758	4.965	0.03681
Allotetrahydrocortisol	metab_19275	1.060	4.469E-5	6.621E-5	5.029	0.01934	4.403	0.08475	3.188	0.256	3.591	0.1901	3.392	0.1346
St(24:2_O4)	metab_42143	1.060	0.001206	0.001264	4.673	0.257	4.214	0.7767	3.017	0.243	3.584	0.6676	2.878	0.1951
Ser Gly Phe	metab_25103	1.060	1.325E-5	4.891E-5	5.196	0.04208	4.935	0.04136	6.215	0.004655	6.757	0.007688	5.893	0.01315
1-(2-(4-(3-Phenyl-2H-1-Benzopyran-2-Yl)Phenoxy)Ethyl)Piperidine	metab_44301	1.060	1.325E-5	4.891E-5	4.221	0.04794	3.937	0.03844	5.168	0.01046	4.918	0.01585	4.955	0.01371
Ile-Thr-Tyr	metab_23713	1.060	1.325E-5	4.891E-5	5.133	0.01951	4.813	0.04293	5.884	0.007259	5.435	0.01794	5.819	0.01205

Gly-Gly-Leu	metab_19882	1.059	1.518E-5	4.891E-5	3.901	0.09471	3.752	0.04009	5.061	0.01164	5.248	0.006762	4.764	0.008974
(2E,4E)-8-(Beta-D-Glucopyranosyloxy)-2,7-Dimethyldeca-2,4-Dienedioic Acid	metab_36863	1.059	1.325E-5	4.891E-5	4.057	0.03263	4.984	0.009349	6.146	0.007562	5.666	0.008914	5.751	0.004921
Val Lys	metab_697	1.059	1.421E-5	4.891E-5	4.389	0.06433	4.575	0.07516	5.387	0.02056	6.191	0.01104	5.464	0.0108
Thr Gln	metab_30298	1.059	3.121E-5	5.392E-5	3.266	0.001283	3.296	0.1014	4.222	0.1295	5.496	0.01752	3.451	0.1059
Zinostatin	metab_28813	1.058	2.156E-5	4.891E-5	4.114	0.1082	5.735	0.01081	4.921	0.06435	3.926	0.2001	5.126	0.04028
Ile-Lys-His	metab_50373	1.058	2.881E-5	5.187E-5	4.194	0.2558	3.819	0.04214	4.791	0.1427	5.668	0.05387	4.826	0.132
Cucurbic Acid	metab_42502	1.058	3.121E-5	5.392E-5	5.808	0.0136	4.268	0.06261	4.187	0.01897	4.099	0.0385	4.319	0.01708
Bioadykinin Fragment 1-5	metab_36626	1.058	1.518E-5	4.891E-5	5.218	0.02303	4.342	0.05076	3.933	0.1057	4.692	0.03941	4.766	0.03402
Murnac-Tripeptide	metab_48694	1.058	1.325E-5	4.891E-5	3.601	0.2818	4.523	0.03061	5.759	0.01238	4.915	0.03837	5.123	0.03223
Verdamicin	metab_36387	1.057	2.071E-5	4.891E-5	4.63	0.03057	5.455	0.008896	5.124	0.01591	5.108	0.01636	4.687	0.01938
Pro-Ile-Ser	metab_48290	1.057	2.53E-5	4.891E-5	2.947	0.1341	3.825	0.0321	4.507	0.02694	5.185	0.0122	3.845	0.06157
Arg-Glu	metab_50627	1.057	1.958E-5	4.891E-5	3.225	0.1142	4.141	0.04421	4.452	0.1435	5.249	0.04447	4.549	0.02833
Penigequinolone B	metab_8418	1.057	1.99E-5	4.891E-5	5.507	0.03052	4.638	0.09322	3.858	0.1643	5.671	0.009967	4.487	0.117
Lys-Ser-Tyr	metab_2178	1.057	1.939E-5	4.891E-5	3.684	0.2207	3.997	0.1586	4.965	0.03375	5.749	0.0165	4.803	0.07507
Taraxacolide 1-O-B-D-Glucopyranoside	metab_43968	1.057	1.616E-5	4.891E-5	4.674	0.03507	4.358	0.07007	4.596	0.05218	5.105	0.01942	3.636	0.17
Pi(5-Iso Pgf2Vi/18:0)	metab_36488	1.056	1.325E-5	4.891E-5	5.693	0.01707	5.205	0.0289	5.084	0.03634	3.622	0.2924	5.611	0.04334
Minoxidil Glucuronide	metab_44268	1.056	1.325E-5	4.891E-5	3.473	0.1009	4.071	0.02728	4.674	0.01942	5.512	0.01231	3.814	0.0943
Norbinaltorphimine	metab_14658	1.056	3.902E-5	6.072E-5	5.112	0.001284	5.208	0.05625	6.34	0.01922	5.183	0.006605	5.69	0.0277
Meta-O-Dealkylated Flecainide	metab_35282	1.056	2.515E-5	4.891E-5	3.591	0.07595	3.6	0.04509	4.855	0.01003	5.085	0.009735	4.607	0.01089
3,7-Dihydropurin-6-One	metab_32542	1.056	1.994E-5	4.891E-5	4.031	0.09628	3.622	0.1562	3.887	0.07647	4.213	0.04679	4.429	0.04169
Thr-Ile	metab_33962	1.055	1.421E-5	4.891E-5	4.065	0.05532	4.146	0.02032	5.26	0.009004	6.059	0.008226	4.959	0.01539
Ala-Ser	metab_31825	1.055	1.807E-5	4.891E-5	3.766	0.078	4.099	0.07539	4.928	0.04364	5.619	0.02438	4.966	0.03652
Met Pro Met	metab_6927	1.055	4.184E-5	6.344E-5	4.634	0.05258	4.724	0.01451	5.267	0.02126	6.323	0.003744	4.256	0.3379
Tyrosylhydroxyproline	metab_33559	1.055	1.325E-5	4.891E-5	3.916	0.05856	4.887	0.01996	5.682	0.01503	5.822	0.005951	5.603	0.01512
4,4-Dimethyl-2-[3-Carboxylatopropyl]-2-Tridecyloxazolidine 3-Oxide	metab_15024	1.055	1.421E-5	4.891E-5	5.306	0.06849	5.108	0.05576	3.997	0.2445	5.41	0.02989	4.591	0.04918
Ala-Val-Phe	metab_45371	1.055	1.325E-5	4.891E-5	4.574	0.00539	4.343	0.01948	5.286	0.007837	5.101	0.008719	5.403	0.004114
Pro Ser Arg	metab_6388	1.054	1.325E-5	4.891E-5	4.631	0.03034	5.706	0.0135	5.281	0.03306	5.977	0.006523	5.496	0.0243
Val Arg Asn	metab_28393	1.054	2.724E-5	5.055E-5	5.571	0.03156	5.243	0.007295	5.197	0.1889	5.64	0.1372	4.379	0.07167
N1,N10-Dicoumaroylspermidine	metab_34484	1.054	1.421E-5	4.891E-5	3.423	0.1431	4.07	0.07443	5.016	0.01751	5.565	0.008225	4.193	0.03627
Hexyl Glucoside	metab_42695	1.054	1.629E-5	4.891E-5	4.697	0.02086	3.438	0.0909	3.083	0.1561	4.741	0.01404	3.686	0.08985
Bafilomycin A1(Baf-A1)	metab_37772	1.053	1.325E-5	4.891E-5	2.98	0.003272	2.903	0.001172	3.204	0.1513	5.096	0.02535	3.018	0.0102
(2S)-2-(2,6-Dihydroxyphenyl)-5,7-Dihydroxy-2,3-Dihydrochromen-4-One	metab_13975	1.053	2.402E-5	4.891E-5	4.729	0.01938	3.151	0.001858	3.228	0.002697	3.232	0.006601	3.241	0.00326
Pisumoside A	metab_37256	1.053	2.411E-5	4.891E-5	4.499	0.04053	5.113	0.02685	4.27	0.05986	2.852	0.2333	4.322	0.09071
2''-(6''-P-Coumaroylglucosyl)Quercitrin	metab_50107	1.053	2.591E-5	4.929E-5	4.147	0.1916	5.854	0.01202	5.454	0.04418	3.831	0.2574	5.884	0.03192
Met Asn	metab_28851	1.052	2.359E-5	4.891E-5	3.585	0.08909	2.934	0.1595	4.151	0.04513	5.289	0.01219	3.54	0.1076

25-Desacetyl Rifapentine	metab_20079	1.052	1.325E-5	4.891E-5	5.111	0.0308	5.655	0.01419	4.809	0.1126	5.24	0.02989	5.506	0.0153
Pi(O-12:0/26:7)	metab_44038	1.052	2.91E-5	5.21E-5	4.535	0.08046	3.548	0.2595	3.213	0.006767	5.466	0.01455	3.195	0.03638
S-(Formylmethyl)Glutathione	metab_1305	1.052	0.0001197	0.0001444	4.571	0.04306	4.61	0.03368	5.203	0.01469	3.737	0.2792	4.572	0.04825
Ala-Glu-Ser	metab_50317	1.051	2.298E-5	4.891E-5	3.735	0.2129	3.56	0.2697	4.992	0.05357	5.196	0.06938	4.419	0.07212
Asp-Ser	metab_50471	1.051	2.455E-5	4.891E-5	3.815	0.1193	3.997	0.1793	4.86	0.04853	5.532	0.01913	4.927	0.03736
Petasinoside	metab_35961	1.051	1.325E-5	4.891E-5	5.519	0.006142	4.359	0.06547	5.489	0.01525	5.973	0.007181	4.788	0.04914
D-Pantothenoyl-L-Cysteine	metab_38496	1.051	2.298E-5	4.891E-5	3.997	0.05274	3.309	0.08432	4.527	0.02303	4.511	0.02293	4.141	0.03447
Argatroban	metab_24432	1.050	1.712E-5	4.891E-5	4.137	0.2376	4.994	0.06239	5.221	0.03712	5.927	0.01735	4.502	0.1444
Leu-Ile	metab_35670	1.050	1.325E-5	4.891E-5	4.561	0.03367	4.247	0.0155	5.466	0.01073	6.215	0.00561	5.137	0.01292
B-D-Xylopyranosyl-(1->4)-A-L-Rhamnopyranosyl-(1->2)-L-Arabinose	metab_32970	1.050	4.513E-5	6.646E-5	5.698	0.017	4.973	0.04404	3.652	0.5622	4.899	0.1032	4.045	0.4977
Altiloxin B	metab_24055	1.049	5.443E-5	7.614E-5	2.876	0.2637	3.084	0.3349	4.266	0.06498	5.15	0.02863	3.644	0.5101
Aloe-Emodin	metab_42179	1.049	0.0002703	0.0003001	4.586	0.3151	3.835	0.6529	2.996	0.3415	3.291	0.6479	2.652	0.01018
4-Formyl Indole	metab_12616	1.048	1.9E-5	4.891E-5	4.644	0.0088	4.119	0.06062	2.919	0.1328	3.568	0.05656	3.089	0.2312
Ganoderic Acid L	metab_36745	1.048	1.325E-5	4.891E-5	5.676	0.004508	5.509	0.003266	5.489	0.01211	4.501	0.03641	4.959	0.03646
5-Formiminotetrahydrofolic Acid	metab_27629	1.048	3.241E-5	5.532E-5	5.012	0.05013	6.057	0.01424	5.149	0.04009	5.057	0.06752	5.302	0.02268
Monoketocholic Acid	metab_14757	1.048	8.361E-5	0.000107	5.457	0.03604	3.753	0.2703	3.617	0.1119	5.245	0.02207	3.939	0.2748
Phe Tyr Asn Glu	metab_25261	1.048	3.414E-5	5.691E-5	5.688	0.02972	4.129	0.3243	4.743	0.2142	5.271	0.07873	4.809	0.165
Asn Ser Val	metab_28808	1.048	1.889E-5	4.891E-5	3.345	0.3909	3.805	0.1563	4.591	0.14	5.726	0.01619	4.176	0.1555
Arg Ile Phe	metab_22011	1.048	2.359E-5	4.891E-5	4.06	0.01898	4.039	0.09279	5.405	0.01994	5.458	0.01829	4.941	0.02663
8-Acetoxy-4'-Methoxypinoresinol 4-Glucoside	metab_44837	1.048	2.515E-5	4.891E-5	3.702	0.003275	3.625	0.001173	4.717	0.02817	3.957	0.03608	3.963	0.08076
Phe-Ser	metab_33142	1.047	1.325E-5	4.891E-5	4.457	0.05737	4.582	0.007582	5.77	0.008841	6.292	0.002314	5.447	0.008359
Pro-Asn	metab_1595	1.047	1.325E-5	4.891E-5	3.814	0.05401	3.501	0.2533	4.828	0.03003	5.269	0.01754	4.425	0.04522
Taxine B	metab_23177	1.047	1.325E-5	4.891E-5	5.411	0.0241	5.54	0.02848	5.86	0.01162	6.369	0.005634	4.894	0.1013
Ala-Arg-Leu	metab_2879	1.047	0.002159	0.002232	2.775	0.5692	3.305	0.1484	4.657	0.7734	2.779	0.08319	4.036	0.8679
12-Oxo-C-Ltb3	metab_37129	1.047	1.325E-5	4.891E-5	4.07	0.06151	4.622	0.05447	4.853	0.02183	2.701	0.09667	5.078	0.02964
Cholic Acid	metab_40050	1.047	0.0008438	0.0008931	5.382	0.2192	4.761	1.1	3.61	0.7966	4.717	0.3945	3.372	0.2933
2-Isopropylmalic Acid	metab_35945	1.047	1.325E-5	4.891E-5	6.368	0.001738	5.889	0.001844	4.912	0.00814	6.641	0.005539	5.362	0.008473
Anandamide	metab_17586	1.046	1.325E-5	4.891E-5	4.989	0.02563	5.146	0.007824	4.299	0.02133	5.814	0.006093	4.546	0.04614
Phe-Pro	metab_22478	1.046	1.325E-5	4.891E-5	5.063	0.03905	4.75	0.0381	6.034	0.005626	6.671	0.002829	5.604	0.0192
Mesotocin	metab_20548	1.046	4.337E-5	6.498E-5	5.047	0.05494	5.925	0.0118	5.2	0.06162	3.753	0.1076	5.149	0.09224
Phe Gln	metab_2770	1.046	1.325E-5	4.891E-5	4.571	0.04575	4.697	0.014	5.931	0.006052	6.489	0.006473	5.458	0.005732
Arg-Phe-Tyr	metab_28978	1.046	1.99E-5	4.891E-5	3.43	0.111	3.555	0.1292	4.828	0.05157	5.35	0.02297	4.027	0.06812
Asn-Phe-Ile	metab_44122	1.045	3.353E-5	5.632E-5	4.403	0.0163	4.24	0.0249	5.314	0.01911	5.28	0.01077	5.302	0.007642
Taurolithocholic Acid	metab_45196	1.045	4.888E-5	7.017E-5	3.835	0.06648	2.591	0.1227	2.774	0.2016	4.813	0.0122	2.893	0.2036
Cer(14:0_2O/6:0)	metab_40567	1.045	2.801E-5	5.127E-5	4.163	0.04351	4.206	0.0648	4.513	0.03494	4.457	0.02144	5.083	0.02577

Asn-Leu-Leu	metab_37622	1.045	1.325E-5	4.891E-5	4.022	0.06492	4.702	0.01578	5.494	0.008803	5.731	0.007947	5.557	0.005438
D-Phe-L-Pro-L-Arg-(Chloromethyl) Ketone	metab_46729	1.044	2.23E-5	4.891E-5	3.288	0.4355	4.323	0.06429	5.185	0.01265	5.086	0.01691	4.336	0.0207
Delphinidin	metab_20654	1.044	1.807E-5	4.891E-5	3.26	0.204	3.546	0.3285	4.825	0.0182	4.402	0.0423	4.592	0.02107
Tyr-Pro	metab_34021	1.044	1.325E-5	4.891E-5	4.74	0.01716	3.771	0.03789	4.972	0.008795	5.791	0.004055	4.533	0.02069
Leu-Met	metab_34682	1.044	1.325E-5	4.891E-5	3.452	0.1011	3.739	0.07601	4.903	0.01103	5.229	0.007866	4.63	0.01431
Ala-Leu	metab_33312	1.044	2.535E-5	4.891E-5	3.776	0.04341	3.755	0.04331	5.024	0.008957	5.637	0.01239	4.497	0.01392
Thr-Pro	metab_2035	1.044	1.807E-5	4.891E-5	4.547	0.0889	4.432	0.03481	5.686	0.01025	6.429	0.004849	5.137	0.007917
Ethyl 1-(4-Tert-Butylphenyl)-5-Cyano-4-Methyl-6-Oxopyridazine-3-Carboxylate	metab_37607	1.044	1.9E-5	4.891E-5	3.104	0.003273	3.23	0.13	4.492	0.01876	4.98	0.007707	3.995	0.05791
Thr Arg Glu Glu Lys	metab_12967	1.044	2.262E-5	4.891E-5	5.174	0.04554	5.046	0.1267	5.303	0.03851	3.94	0.1582	4.778	0.08614
Tyr-Arg-Leu	metab_46680	1.044	1.325E-5	4.891E-5	3.501	0.09831	4.13	0.07708	4.828	0.02688	5.081	0.01416	5.006	0.01342
Ala-Gln	metab_30242	1.044	1.325E-5	4.891E-5	4.51	0.08601	4.081	0.1441	5.333	0.01381	6.105	0.004735	4.921	0.05386
Thr-Asn	metab_30396	1.043	2.49E-5	4.891E-5	4.389	0.09929	4.4	0.09746	5.459	0.007653	6.144	0.01142	5.308	0.02274
4-(Glutamylamino) Butanoate	metab_33034	1.043	0.0001444	0.0001694	2.427	0.1786	2.073	0.01734	2.467	0.4765	4.433	0.1103	2.392	0.3549
Dgdg(O-9:0/2:0)	metab_16188	1.043	0.001605	0.001671	5.707	0.5564	4.597	0.526	3.746	0.1461	3.988	0.495	3.68	0.003261
Arg-Ala-Leu	metab_2783	1.043	1.325E-5	4.891E-5	4.785	0.02566	5.028	0.01668	6.167	0.02069	6.584	0.01901	5.908	0.02112
Beta-Hydroxyisovaleric Acid	metab_47563	1.043	1.325E-5	4.891E-5	5.807	0.004248	5.856	0.003756	4.788	0.002667	5.677	0.00695	5.479	0.007803
Benzoylhypaconine	metab_45782	1.043	2.53E-5	4.891E-5	3.873	0.169	5.116	0.02191	4.284	0.1003	4.167	0.1277	4.891	0.01582
Leu Gly Val	metab_6944	1.043	1.874E-5	4.891E-5	4.37	0.05024	4.064	0.03861	3.967	0.06582	6.064	0.01734	3.849	0.06601
Erythrono-1,4-Lactone	metab_5510	1.043	2.455E-5	4.891E-5	4.137	0.02728	5.237	0.007803	4.47	0.02393	4.297	0.04533	4.478	0.01744
Lys Asp Leu Ser Leu	metab_12756	1.042	1.9E-5	4.891E-5	3.706	0.1558	4.942	0.03801	3.957	0.2663	2.831	0.2509	4.779	0.07951
Tyr-Lys	metab_29569	1.042	1.325E-5	4.891E-5	2.839	0.4329	3.853	0.0668	4.459	0.04887	5.056	0.01507	4.209	0.04579
N-Desmethyl Zolmitriptan	metab_7739	1.042	4.504E-5	6.637E-5	3.337	0.2336	3.353	0.2382	4.57	0.0455	5.314	0.01434	3.715	0.1863
Trp-His-Arg	metab_23317	1.042	2.49E-5	4.891E-5	4.354	0.06067	4.979	0.08295	5.601	0.01091	3.876	0.2668	4.954	0.02417
Glabranin	metab_15211	1.042	1.421E-5	4.891E-5	5.425	0.1275	3.61	0.001859	3.687	0.002698	5.102	0.06822	3.718	0.04329
(1Alpha,3Beta,20S,22R,24S,25S)-Pubescenin	metab_48087	1.042	1.325E-5	4.891E-5	3.47	0.1829	4.825	0.03007	4.746	0.01314	4.618	0.03583	5.191	0.0318
Val Val	metab_5924	1.041	4.184E-5	6.344E-5	4.481	0.08427	4.027	0.1635	4.012	0.1941	6.056	0.01169	3.57	0.2667
Salinomycin	metab_38960	1.041	1.325E-5	4.891E-5	3.89	0.1839	5.256	0.02386	4.496	0.04964	4.216	0.02094	4.626	0.03583
Nadh	metab_1395	1.041	1.325E-5	4.891E-5	4.355	0.0938	5.83	0.01454	5.507	0.0133	3.572	0.1741	5.724	0.01898
4-Hydroxy-2-Nonenal-Glutathione Conjugate	metab_49001	1.040	2.23E-5	4.891E-5	3.378	0.1603	3.896	0.04301	4.37	0.07895	5.533	0.0132	4.312	0.06509
Y1 Receptor Antagonist 1	metab_44786	1.040	1.518E-5	4.891E-5	3.548	0.1969	4.343	0.05215	4.484	0.04118	5.316	0.01659	3.833	0.1418
(R)-Laudanidine	metab_36817	1.040	1.325E-5	4.891E-5	5.279	0.00939	4.432	0.05338	4.79	0.02976	4.984	0.02125	3.973	0.1079
Arbutin	metab_47200	1.040	1.325E-5	4.891E-5	4.262	0.02555	4.692	0.02789	3.602	0.07655	2.784	0.1604	4.618	0.02774
2-(1,3-Dioxoisoindol-2-Yl)-N-[(5-Methyl-1,2-Oxazol-3-Yl)Methyl]Acetamide	metab_2270	1.040	1.325E-5	4.891E-5	3.339	0.001283	3.328	0.001858	3.405	0.002698	5.254	0.01592	3.419	0.00326
Mikamycin B	metab_44778	1.040	1.325E-5	4.891E-5	4.714	0.047	5.648	0.01171	6.426	0.009702	5.558	0.01168	6.611	0.007585
Asp-Lys-His	metab_31392	1.039	1.325E-5	4.891E-5	3.091	0.003273	3.014	0.001172	4.093	0.1062	5.022	0.03463	3.199	0.1238

Polypodine B	metab_41451	1.039	3.603E-5	5.851E-5	3.554	0.15	4.329	0.03972	3.896	0.07548	4.401	0.08259	3.533	0.1686
Lpa(18:2)	metab_41712	1.039	1.325E-5	4.891E-5	6.583	0.01196	6.632	0.004681	7.144	0.009336	6.122	0.01365	6.461	0.006874
2-Amino-3-Methyl-1-Pyrrolidin-1-Yl-Butan-1-One	metab_49177	1.039	1.325E-5	4.891E-5	4.777	0.02624	4.61	0.00478	5.895	0.01061	6.369	0.003706	5.469	0.0124
Acoric Acid	metab_16803	1.039	1.325E-5	4.891E-5	5.406	0.01064	3.733	0.001859	4.392	0.0405	5.242	0.02901	4.007	0.116
Tyrosyl-Threonine	metab_47538	1.039	1.99E-5	4.891E-5	3.602	0.102	3.692	0.05175	4.987	0.009236	5.165	0.01318	4.581	0.01425
Gly-Leu-Leu	metab_44576	1.038	1.325E-5	4.891E-5	4.869	0.0123	4.449	0.01777	5.766	0.006235	6.183	0.007425	5.301	0.01013
Niacinamide	metab_928	1.038	1.518E-5	4.891E-5	5.478	0.02203	3.878	0.1051	3.571	0.0833	4.789	0.02319	3.374	0.1035
Gibberellin A29	metab_35371	1.038	1.325E-5	4.891E-5	3.439	0.1395	4.751	0.02419	5.158	0.02576	4.269	0.1706	5.501	0.009718
2-(2-Methoxyethoxy)-N-[(7-Oxoazepan-2-Yl)Methyl]Acetamide	metab_8108	1.038	1.325E-5	4.891E-5	3.689	0.1491	4.435	0.04383	5.65	0.01134	4.624	0.02673	5.123	0.01213
C-Quens	metab_47947	1.037	1.421E-5	4.891E-5	2.926	0.2036	3.703	0.09515	4.461	0.02506	5.161	0.01537	3.898	0.07777
1-O-All-Trans-Retinoyl-Beta-Glucuronic Acid	metab_25739	1.037	1.712E-5	4.891E-5	4.807	0.1165	4.616	0.1306	5.936	0.007948	5.713	0.01701	5.12	0.06311
Cystathionine	metab_31735	1.037	1.958E-5	4.891E-5	5.32	0.02497	3.834	0.123	4.275	0.05107	4.506	0.05146	4.345	0.065
Dg(Pgf2Alpha/0:0/A-17:0)	metab_15227	1.037	2.44E-5	4.891E-5	5.488	0.1747	4.889	0.05941	4.134	0.2026	5.939	0.127	4.73	0.2527
Pglu-His-Pro	metab_49705	1.037	1.325E-5	4.891E-5	4.509	0.02806	3.629	0.1174	4.88	0.02511	5.625	0.03473	4.29	0.05591
Gly-Asp	metab_31858	1.036	1.9E-5	4.891E-5	3.774	0.1235	4.002	0.1511	4.768	0.04043	5.369	0.02587	4.907	0.03767
Gly-Phe-Ser	metab_48458	1.036	2.535E-5	4.891E-5	3.418	0.05928	2.791	0.2095	4.028	0.02692	4.992	0.02032	3.415	0.07752
Glu-Val-Lys	metab_24295	1.036	1.99E-5	4.891E-5	5.248	0.01394	4.749	0.07349	3.848	0.2165	5.677	0.01641	4.073	0.1648
Ile Gly Phe	metab_10847	1.036	1.421E-5	4.891E-5	4.24	0.1323	4.442	0.02332	5.771	0.006728	5.683	0.005776	4.943	0.01183
Malvidin 3-(6"-Acetyl-Galactoside)	metab_50483	1.036	1.325E-5	4.891E-5	4.491	0.02172	5.823	0.02278	5.609	0.03085	3.689	0.1384	5.511	0.04935
Tyr-Val	metab_24098	1.035	2.785E-5	5.105E-5	3.232	0.2701	3.79	0.09228	4.487	0.05987	5.408	0.01349	3.74	0.1809
Abacavir	metab_44397	1.035	2.139E-5	4.891E-5	3.637	0.04649	2.829	0.1158	3.356	0.1116	4.906	0.009361	3.523	0.08997
Coronaric Acid	metab_40138	1.035	1.325E-5	4.891E-5	5.254	0.01043	4.892	0.0525	4.204	0.03183	4.674	0.02881	5.114	0.01651
Zalcitabine	metab_50276	1.034	2.53E-5	4.891E-5	3.119	0.1392	3.982	0.06846	5.011	0.0872	5.025	0.05715	4.594	0.08588
Trp-Ala	metab_35826	1.034	1.807E-5	4.891E-5	3.405	0.09285	3.529	0.0657	4.653	0.02107	5.304	0.01079	4.314	0.0211
Pro-Thr-Phe	metab_35975	1.034	1.325E-5	4.891E-5	4.225	0.03104	4.059	0.03736	5.287	0.005299	5.786	0.008844	4.953	0.006081
Arginyl-Gamma-Glutamate	metab_2730	1.034	1.325E-5	4.891E-5	3.896	0.1483	4.391	0.0987	5.339	0.02775	6.051	0.0174	4.958	0.04885
Asn-Tyr	metab_33122	1.034	1.325E-5	4.891E-5	3.342	0.05354	4.172	0.02269	4.752	0.02724	5.465	0.005472	4.594	0.04059
Leu Ile Val	metab_24197	1.033	2.53E-5	4.891E-5	5.475	0.01496	4.314	0.05307	4.311	0.05399	4.019	0.1186	4.8	0.02814
Ala-Gly-His	metab_50659	1.033	1.518E-5	4.891E-5	3.673	0.07866	3.504	0.0774	4.023	0.04127	5.4	0.008061	4.164	0.02771
4-[2-[[2-(4-Hydroxyphenyl)Acetyl]Amino]Ethylamino]-4-Oxobut-2-Enoic Acid	metab_47230	1.033	1.325E-5	4.891E-5	4.32	0.04913	5.258	0.004287	6.271	0.005478	5.891	0.008203	6.019	0.01148
Gly-Gly-Tyr	metab_33197	1.033	1.325E-5	4.891E-5	2.52	0.003266	2.443	0.001169	3.169	0.3108	4.675	0.0312	2.558	0.01018
Trp-Gly-Ile	metab_45333	1.033	1.616E-5	4.891E-5	4.273	0.05917	4.358	0.02981	4.526	0.01412	5.039	0.01133	3.644	0.09181
Val-Asp-Ile	metab_11101	1.032	2.455E-5	4.891E-5	4.711	0.01811	3.83	0.1249	4.038	0.06487	2.959	0.172	4.092	0.06931
Methionyl-Arginine	metab_37968	1.032	1.325E-5	4.891E-5	4.541	0.02723	4.417	0.04413	5.416	0.01277	6.111	0.004659	5.344	0.01162
Myricanene A 5-[Arabinosyl-(1->6)-Glucoside]	metab_9254	1.032	1.325E-5	4.891E-5	3.601	0.4974	4.833	0.04237	5.063	0.04208	4.292	0.1531	5.605	0.03116

15-Hexadecanolide	metab_41752	1.032	1.325E-5	4.891E-5	3.891	0.06094	3.498	0.2761	4.52	0.02329	4.059	0.02502	4.456	0.02092
His-Ala-Ser	metab_50698	1.032	2.156E-5	4.891E-5	3.79	0.0363	3.821	0.06722	4.639	0.01898	5.712	0.01311	4.551	0.02096
Gly-Leu-Ile	metab_11022	1.031	1.325E-5	4.891E-5	5.204	0.02366	4.748	0.02292	6.07	0.003328	6.463	0.005702	5.539	0.006221
Boc-Val-Pro-Arg-Mca	metab_45234	1.031	1.712E-5	4.891E-5	3.721	0.2547	4.809	0.0248	4.368	0.0579	5.026	0.02922	4.501	0.08581
Leu-Thr-Pro	metab_47832	1.031	1.325E-5	4.891E-5	4.203	0.01616	3.574	0.09556	4.819	0.01209	5.59	0.005295	4.334	0.02369
Pe(6 Keto-Pgf1Alpha/14:0)	metab_27109	1.031	1.325E-5	4.891E-5	5.798	0.01686	5.349	0.05307	5.129	0.04179	4.179	0.006604	5.908	0.01022
1-Carboxyethyltyrosine	metab_7418	1.031	1.325E-5	4.891E-5	4.153	0.02093	4.024	0.05843	5.33	0.005808	4.914	0.01674	4.689	0.01657
Isoleucyl-Gamma-Glutamate	metab_35690	1.031	2.411E-5	4.891E-5	3.934	0.02582	3.825	0.03456	3.527	0.04953	5.373	0.004995	3.801	0.04485
Periplocin	metab_12587	1.031	1.325E-5	4.891E-5	6.237	0.009664	6.361	0.01889	6.628	0.009845	4.8	0.07364	6.972	0.003282
Glu-Phe-Thr	metab_35546	1.031	2.67E-5	5.021E-5	3.957	0.02989	3.946	0.07638	5.163	0.01077	5.12	0.01394	4.936	0.004341
Nummularine B	metab_37827	1.031	1.743E-5	4.891E-5	3.62	0.3389	3.081	0.2694	4.178	0.06977	5.164	0.02984	3.966	0.08219
Arg-Ala	metab_50701	1.030	1.325E-5	4.891E-5	3.674	0.04524	3.871	0.03773	4.858	0.03768	5.581	0.03508	4.658	0.02403
7,14-Dihydroxyoctadeca-4,8,10,12-Tetraenedioylcarnitine	metab_44839	1.030	1.325E-5	4.891E-5	4.347	0.04033	4.176	0.0788	5.425	0.006608	4.897	0.01375	4.792	0.02821
Gly Val	metab_28852	1.030	1.325E-5	4.891E-5	4.367	0.03881	4.103	0.0324	5.395	0.007629	5.928	0.01114	4.885	0.007501
Annomuricatin A	metab_33710	1.030	1.325E-5	4.891E-5	3.679	0.1254	5.142	0.01837	5.848	0.01938	4.914	0.05559	5.564	0.02401
Pa(Pge1/I-13:0)	metab_37519	1.030	1.421E-5	4.891E-5	4.744	0.0187	4.299	0.07095	3.967	0.08596	2.947	0.003717	3.75	0.1327
Libenzapril	metab_44162	1.030	1.325E-5	4.891E-5	4.379	0.01853	4.79	0.006836	5.467	0.009899	3.514	0.09116	5.266	0.01295
Cinnzeylanine	metab_49085	1.030	3.757E-5	5.997E-5	4.415	0.1078	4.197	0.1403	5.109	0.1969	6.533	0.3543	4.644	0.2337
H-Ser-Leu-Ile-Gly-Arg-Leu-Oh	metab_48521	1.030	1.325E-5	4.891E-5	5.646	0.01011	5.017	0.02542	4.807	0.04828	3.802	0.295	5.482	0.01251
Poly-G-D-Glutamate	metab_33031	1.030	1.325E-5	4.891E-5	5.828	0.009885	5.417	0.01022	4.269	0.03246	5.555	0.04111	4.786	0.0543
Xi-Linalool 3-[Rhamnosyl-(1->6)-Glucoside]	metab_23674	1.030	1.99E-5	4.891E-5	5.805	0.01192	4.61	0.06761	4.71	0.08689	4.97	0.02991	5.204	0.01241
(9S,10S)-10-Hydroxy-9-(Phosphonooxy)Octadecanoate	metab_17204	1.029	0.0001405	0.0001655	3.527	0.2705	3.73	0.2338	4.903	0.02012	3.583	0.2683	4.342	0.0975
Loperamide	metab_49927	1.029	8.131E-5	0.0001044	3.596	0.2576	3.284	0.001173	3.596	0.3254	5.546	0.02489	3.4	0.0102
Mppa (Methyl Pyropheophorbide-A)	metab_43959	1.029	1.325E-5	4.891E-5	4.8	0.0226	4.461	0.02408	4.294	0.03469	3.77	0.07799	5.139	0.0161
Gly-Phe	metab_47673	1.029	1.421E-5	4.891E-5	4.283	0.03647	4.215	0.018	5.484	0.01124	5.994	0.004702	4.958	0.01185
Arg-Ala-Met	metab_2161	1.029	1.325E-5	4.891E-5	4.197	0.08297	4.697	0.03693	5.913	0.006852	5.885	0.008419	5.447	0.01434
Mucronine D	metab_40569	1.028	2.359E-5	4.891E-5	3.658	0.003275	3.822	0.08723	4.795	0.03701	3.792	0.003721	4.051	0.05253
Matricin	metab_39031	1.028	1.325E-5	4.891E-5	3.881	0.06311	4.748	0.02989	5.446	0.02275	3.295	0.7641	5.176	0.01667
Val-Pro-Leu	metab_36174	1.028	1.325E-5	4.891E-5	4.049	0.05843	4.626	0.0169	5.595	0.01081	6.145	0.003988	4.912	0.01731
2-Hydroxyadipic Acid	metab_1308	1.028	1.325E-5	4.891E-5	5.816	0.008103	6.707	0.004836	7.283	0.003954	5.298	0.01735	6.918	0.004509
2-Keto-3-Deoxy-D-Mannooctanoic Acid	metab_50036	1.028	3.902E-5	6.072E-5	4.992	0.01724	3.341	0.2002	3.03	0.006765	4.482	0.2003	2.996	0.0102
Val-Ser	metab_32006	1.028	2.075E-5	4.891E-5	3.46	0.04547	3.516	0.2501	4.798	0.03849	5.135	0.05946	4.331	0.04814
Trypanothione Disulfide	metab_48003	1.027	1.325E-5	4.891E-5	5.319	0.01125	5.019	0.01434	4.68	0.03906	3.212	0.3676	5.243	0.02716
Ala Tyr Gly	metab_1267	1.027	2.66E-5	5.007E-5	3.487	0.6212	4.124	0.1969	4.948	0.03726	5.773	0.01857	4.251	0.1286
Furaquinocin C	metab_31317	1.027	1.616E-5	4.891E-5	2.714	0.003269	2.638	0.00117	3.316	0.3104	4.868	0.02471	2.822	0.08644

Indolophenanthridine	metab_13617	1.027	1.518E-5	4.891E-5	4.481	0.03215	4.812	0.01945	3.506	0.1638	3.733	0.09449	4.13	0.07589
2'-O-Methylcajanone	metab_6950	1.027	2.751E-5	5.075E-5	4.571	0.07827	4.282	0.2037	5.372	0.04007	4.683	0.07246	5.156	0.07964
Ile-Asp-Leu	metab_44550	1.026	1.325E-5	4.891E-5	2.569	0.04269	3.48	0.1394	4.521	0.02256	3.976	0.05186	4.271	0.0291
2-Naphthylamine	metab_17777	1.026	1.325E-5	4.891E-5	5.349	0.009357	4.636	0.0126	3.598	0.04754	5.054	0.007814	3.866	0.03936
5B-Cyprinol Sulfate	metab_36001	1.026	1.421E-5	4.891E-5	3.455	0.08439	4.859	0.0114	5.295	0.01545	3.799	0.1635	5.464	0.01607
Manumycin A	metab_11670	1.026	2.317E-5	4.891E-5	4.933	0.03016	5.085	0.03308	4.797	0.09453	5.124	0.03575	4.211	0.1457
Acetylsalvipisone	metab_38992	1.025	2.23E-5	4.891E-5	4.133	0.1158	5.917	0.009867	5.866	0.009639	4.721	0.0251	5.859	0.01499
Fumagillol	metab_25852	1.025	2.075E-5	4.891E-5	3.499	0.2717	4.189	0.08763	4.98	0.04848	5.7	0.01765	4.372	0.1926
7-Deacetoxy-7-Oxodeoxygedunin	metab_9326	1.025	2.49E-5	4.891E-5	6.442	0.003931	5.074	0.03185	5.671	0.01786	6.043	0.01331	5.069	0.01852
Gln-Val-Asn	metab_32881	1.025	3.228E-5	5.523E-5	3.664	0.1133	3.307	0.1783	4.484	0.2252	5.014	0.0665	4.305	0.3684
Ala-Phe	metab_25454	1.024	1.518E-5	4.891E-5	4.833	0.07497	4.965	0.01425	6.184	0.003045	6.681	0.005408	5.617	0.01064
Methylprednisolone Hemisuccinate	metab_45270	1.024	1.9E-5	4.891E-5	3.791	0.09184	5.303	0.006856	5.461	0.008362	5.353	0.01574	5.293	0.008784
Buprenorphine 3-Beta-D-Glucuronide	metab_37498	1.024	1.616E-5	4.891E-5	5.137	0.01359	5.166	0.01857	4.689	0.04441	3.209	0.1455	5.408	0.01545
5'-Deoxy-5'-Methylthioadenosine	metab_5516	1.023	2.075E-5	4.891E-5	5.869	0.007643	6.917	0.005151	6.195	0.007558	6.081	0.01174	6.201	0.005602
Thr-Thr-Ile	metab_48558	1.023	1.325E-5	4.891E-5	3.314	0.1868	4.257	0.03065	4.647	0.02287	5.42	0.01311	4.408	0.01813
4-Hydroxy-2-Nonenal-[L-Cys] Conjugate	metab_36717	1.023	2.063E-5	4.891E-5	3.508	0.1654	4.747	0.009306	4.718	0.03959	4.59	0.05867	5.179	0.02687
Saroglitazar	metab_38404	1.023	1.421E-5	4.891E-5	4.929	0.0132	3.643	0.08449	4.525	0.01932	5.523	0.007546	3.798	0.04113
(24E)-3Alpha-Acetoxy-15Alpha-Hydroxy-23-Oxo-7,9(11),24-Lanostatrien-26-Oic Acid	metab_5920	1.023	2.411E-5	4.891E-5	5.938	0.01552	4.89	0.07492	5.405	0.03177	4.877	0.1116	5.208	0.06761
Arg-Gln-Leu	metab_2727	1.023	2.359E-5	4.891E-5	3.989	0.3936	4.744	0.1049	5.321	0.02999	6.215	0.02401	4.812	0.04008
Aspartame	metab_3533	1.023	4.641E-5	6.765E-5	4.418	0.08985	5.436	0.1927	5.596	0.03748	6.402	0.02299	5.406	0.02219
Thr-Thr-Leu	metab_49018	1.022	2.535E-5	4.891E-5	2.61	0.003267	2.77	0.2697	3.838	0.08486	4.679	0.05328	3.341	0.1073
N-4-[2(R)-Amino-3-Mercaptopropyl]Amino-2-Naphthylbenzoyl-(L)-Leucine, Tfa	metab_45190	1.022	1.325E-5	4.891E-5	4.262	0.0675	5.71	0.01149	5.564	0.01115	3.859	0.1026	5.375	0.009078
Toosendanin	metab_48541	1.022	3.78E-5	6.016E-5	4.395	0.05059	4.941	0.02665	4.224	0.1226	2.642	0.654	4.263	0.06863
Asn-Phe-Leu	metab_44445	1.022	1.325E-5	4.891E-5	4.808	0.0158	4.59	0.01687	5.771	0.009187	5.981	0.008839	5.55	0.01299
Hydroxytyrosol	metab_34383	1.022	2.164E-5	4.891E-5	4.913	0.007448	3.562	0.01861	3.381	0.06072	3.185	0.08183	3.454	0.0572
Apigenin	metab_39607	1.022	2.515E-5	4.891E-5	5.403	0.005511	4.308	0.06847	3.528	0.1425	3.504	0.1877	3.833	0.03396
Tyr-Pro-Thr	metab_11037	1.022	1.325E-5	4.891E-5	5.525	0.01706	5.836	0.00321	5.172	0.01107	5.867	0.006883	4.917	0.02671
Enkephalinamide-Leu, Ala(2)-	metab_47867	1.022	2.359E-5	4.891E-5	4.457	0.06031	4.92	0.02346	5.158	0.0223	4.825	0.04185	4.419	0.04881
Imidazolelactic Acid	metab_50376	1.022	9.214E-5	0.000116	5.229	0.03616	3.486	0.2371	3.431	0.006769	4.818	0.02809	3.398	0.0102
Leu Ser Phe	metab_10895	1.021	1.325E-5	4.891E-5	5.732	0.007804	5.174	0.0198	6.14	0.006792	5.697	0.009163	5.841	0.008141
Arg-Thr	metab_50660	1.021	1.518E-5	4.891E-5	3.063	0.2102	3.395	0.1115	4.28	0.07116	5.112	0.04232	4.047	0.06687
Tyr-Phe-His	metab_36695	1.021	1.421E-5	4.891E-5	4.139	0.05496	3.964	0.0687	4.857	0.03165	5.209	0.01671	4.959	0.02532
O-Acetyl-L-Homoserine	metab_29283	1.021	2.177E-5	4.891E-5	4.208	0.03897	4.118	0.05256	3.431	0.09889	5.194	0.01552	3.564	0.07603
Tryptophan Glutamate	metab_36038	1.021	1.518E-5	4.891E-5	3.245	0.2171	4.949	0.02125	4.712	0.02872	4.03	0.0949	4.642	0.03965
Aminodeoxykanamycin	metab_35450	1.021	3.497E-5	5.736E-5	5.138	0.01198	3.732	0.1837	4.045	0.1449	5.916	0.009281	3.919	0.07961

Asn Asp Ala	metab_913	1.021	2.992E-5	5.288E-5	3.766	0.2619	3.616	0.1381	4.496	0.09595	5.656	0.01869	4.334	0.1125
Serotonin	metab_48444	1.021	2.075E-5	4.891E-5	3.778	0.0551	3.732	0.02827	4.796	0.01257	5.507	0.003807	4.564	0.02055
Iprofenin	metab_46473	1.021	2.298E-5	4.891E-5	2.972	0.3104	3.928	0.03096	4.922	0.01542	4.618	0.02173	4.629	0.01739
Met Phe	metab_22094	1.021	1.325E-5	4.891E-5	3.651	0.118	4.494	0.04435	5.637	0.007847	5.004	0.01559	5.091	0.009051
Val-Pro-Tyr	metab_43146	1.021	1.616E-5	4.891E-5	4.304	0.04455	3.856	0.05825	4.731	0.03982	4.613	0.01778	4.785	0.02351
[D-Lys3]-Ghrp-6	metab_3612	1.021	3.902E-5	6.072E-5	5.953	0.02419	4.273	0.1907	4.273	0.002699	5.873	0.03855	4.286	0.003261
N-(2-Cyclohexyl-2-Hydroxyethyl)-2-(2,5-Dioxopyrrolidin-1-Yl)Acetamide	metab_7759	1.021	4.716E-5	6.838E-5	4.317	0.02871	4.307	0.04483	3.602	0.2598	5.323	0.01838	3.732	0.1045
Ile Pro Val	metab_23012	1.021	1.325E-5	4.891E-5	4.955	0.07333	5.548	0.009467	6.445	0.003983	7.021	0.004494	5.741	0.003899
Phe-Ile	metab_37413	1.020	1.325E-5	4.891E-5	4.774	0.02073	4.99	0.008994	6.178	0.007275	6.197	0.003501	5.907	0.006691
Ala-Gly-Ser	metab_50470	1.020	3.248E-5	5.536E-5	4.506	0.08581	4.536	0.03433	5.412	0.04141	6.296	0.01423	5.33	0.03217
Tricyclazole	metab_12692	1.020	2.411E-5	4.891E-5	3.838	0.04783	3.526	0.129	4.485	0.03105	4.313	0.02561	3.587	0.07556
Leu-Ser	metab_33495	1.020	1.9E-5	4.891E-5	4.924	0.0298	4.955	0.009115	6.163	0.006002	6.609	0.00795	5.754	0.007085
7,8-Dimethoxy-1,3-Dihydro-2H-3-Benzazepin-2-One	metab_12161	1.020	2.075E-5	4.891E-5	3.397	0.1258	3.257	0.1729	4.607	0.01681	4.529	0.01699	3.988	0.02546
T2 Triol	metab_14533	1.020	2.969E-5	5.28E-5	5.002	0.03978	5.538	0.01679	5.027	0.0424	4.79	0.0372	4.698	0.07671
Pe(5-Iso Pgf2Vi/Dime(9,3))	metab_35059	1.019	1.518E-5	4.891E-5	5.517	0.01238	5.118	0.03354	4.708	0.05272	3.38	0.00372	5.186	0.03189
Ethyl Linoleate	metab_16125	1.019	1.9E-5	4.891E-5	5.574	0.0451	5.232	0.01586	4.202	0.0985	5.533	0.01916	4.795	0.04875
N-Formylantimycic Acid Methyl Ester	metab_2803	1.019	1.325E-5	4.891E-5	3.745	0.1123	4.284	0.05215	5.102	0.08648	5.925	0.01176	4.545	0.1265
1-(6-Hydroxy-2-Azabicyclo[2.2.1]Heptane-3-Carbonyl)Pyrrolidine-2-Carbonitrile	metab_22488	1.019	1.325E-5	4.891E-5	3.365	0.1519	3.823	0.1427	4.961	0.01953	4.886	0.02164	4.709	0.01805
Statine	metab_19465	1.019	1.325E-5	4.891E-5	4.002	0.01539	3.893	0.04912	5.219	0.008398	5.053	0.01041	4.608	0.007961
Indolapril	metab_44444	1.018	2.751E-5	5.075E-5	3.905	0.05977	4.492	0.03408	3.998	0.0733	5.236	0.02062	4.072	0.04303
Sacubitril	metab_44622	1.018	1.325E-5	4.891E-5	4.116	0.06059	3.898	0.07076	5.023	0.01308	4.482	0.02001	4.776	0.01487
Val-Gly-Val-Ala-Pro-Gly	metab_43797	1.018	1.325E-5	4.891E-5	2.855	0.016	4.012	0.0519	4.845	0.024	4.479	0.03223	4.559	0.02195
Desacetyllevonantradol	metab_37891	1.017	2.359E-5	4.891E-5	3.511	0.1193	3.425	0.1717	4.314	0.01151	4.67	0.01292	4.419	0.03425
Val Tyr Val	metab_7835	1.017	1.325E-5	4.891E-5	5.812	0.005655	5.129	0.009314	5.731	0.006172	5.521	0.00827	5.889	0.00977
Asp-Trp-Leu	metab_44105	1.017	3.028E-5	5.339E-5	4.093	0.06241	2.956	0.3339	3.651	0.1759	4.949	0.01642	3.714	0.1889
Dezinamide	metab_49187	1.016	1.325E-5	4.891E-5	3.157	0.215	3.983	0.03983	4.781	0.03684	5.237	0.0119	4.214	0.0457
Phe-Arg-Leu	metab_46095	1.016	1.518E-5	4.891E-5	3.927	0.05095	3.77	0.09983	5.008	0.01669	5.245	0.01294	4.664	0.0138
4R-Hydroxy Solifenacin	metab_35602	1.016	1.325E-5	4.891E-5	3.376	0.1009	3.729	0.05685	4.965	0.009858	4.89	0.02062	4.53	0.01486
Gln-Gly-His	metab_28354	1.016	7.753E-5	0.0001004	3.329	0.001283	4.11	0.02818	5.238	0.5001	4.689	0.2071	4.311	0.4459
Tyr-Glu	metab_49416	1.016	1.325E-5	4.891E-5	3.187	0.1987	3.816	0.07488	4.538	0.02153	5.323	0.054	4.256	0.04552
Thr-Tyr-Lys	metab_11103	1.016	0.0001461	0.0001711	4.012	0.059	4.524	0.07534	4.021	0.1009	5.347	0.01162	4.024	0.1481
Ile-Pro	metab_47811	1.016	1.421E-5	4.891E-5	4.577	0.03694	4.516	0.01716	5.703	0.008861	6.357	0.003487	5.199	0.008101
Cinncassiol D2 Glucoside	metab_33278	1.016	2.164E-5	4.891E-5	5.178	0.0191	4.553	0.03603	4.741	0.06958	5.062	0.09192	5.245	0.02418
(6R)-N-Ethyl-6-Phenoxy-1,4-Oxazepane-4-Carboxamide	metab_11152	1.016	7.048E-5	9.327E-5	3.791	0.1746	3.807	0.1304	4.136	0.04088	5.781	0.006151	4.056	0.1085
Val-Leu	metab_25859	1.015	1.325E-5	4.891E-5	5.496	0.03098	5.581	0.012	6.795	0.005951	7.035	0.007563	6.427	0.007076

Thr Tyr	metab_2780	1.015	1.325E-5	4.891E-5	4.483	0.05077	4.654	0.008686	5.823	0.007619	6.363	0.006856	5.2	0.01225
Rutacridone Epoxide	metab_32848	1.015	0.0001319	0.0001568	3.583	0.5093	2.84	0.2203	3.333	0.1538	5.149	0.03158	2.661	0.3284
N-Cyano-N'-[2-(Trifluoromethyl)Phenyl]Pyrrolidine-1-Carboximidamide	metab_46448	1.015	3.102E-5	5.388E-5	4.556	0.01683	2.848	0.05718	2.697	0.06583	3.78	0.0579	2.706	0.06646
Glutaminylasparagine	metab_31865	1.014	1.421E-5	4.891E-5	4.239	0.08397	3.789	0.1635	4.719	0.03946	5.954	0.01546	4.476	0.07591
Bipindogulomethyloside	metab_11671	1.014	2.075E-5	4.891E-5	4.338	0.08624	5.272	0.02955	4.683	0.06395	4.967	0.05729	4.636	0.0438
Indole-3-Butyric Acid	metab_37991	1.014	0.0001491	0.0001742	3.518	0.06439	4.434	0.02145	3.487	0.06123	3.502	0.05661	3.791	0.0642
Gly-Lys	metab_50872	1.013	2.411E-5	4.891E-5	3.081	0.2062	2.893	0.37	4.05	0.03177	4.837	0.01065	3.68	0.0541
Hyocholic Acid	metab_39844	1.013	0.002586	0.002665	5.067	0.2695	4.466	1.327	2.857	1.039	3.461	1.274	4.209	0.08385
Cyclosquamosin A	metab_47572	1.013	1.325E-5	4.891E-5	3.87	0.0379	4.933	0.03219	5.918	0.007884	5.169	0.0332	5.508	0.01252
Phenylalanyl-Glycine	metab_25699	1.013	2.075E-5	4.891E-5	4.739	0.05532	4.685	0.03022	5.925	0.007804	6.419	0.004856	5.356	0.00955
2-(Malonylamino)Benzoic Acid	metab_49464	1.013	5.314E-5	7.474E-5	5.622	0.005111	5.37	0.007693	5.548	0.129	5.468	0.09548	4.811	0.1304
Pro-Tyr-Lys	metab_18211	1.012	0.000376	0.0004108	3.12	0.472	2.381	0.3976	3.087	0.4416	4.922	0.02611	2.563	0.3966
Phe-Leu	metab_9118	1.012	1.325E-5	4.891E-5	4.721	0.05352	5.061	0.01808	6.108	0.0093	6.506	0.007596	5.838	0.006463
Leu Ala Gly	metab_28868	1.012	1.325E-5	4.891E-5	5.398	0.04563	5.618	0.004469	6.683	0.004064	7.166	0.007288	6.408	0.003534
Pgp(5-Iso Pgf2Vi/I-14:0)	metab_34565	1.012	1.616E-5	4.891E-5	4.856	0.02162	4.603	0.02625	4.225	0.1111	3.917	0.212	5.149	0.0214
Lycoperoside D	metab_35182	1.012	2.49E-5	4.891E-5	5.089	0.03024	4.573	0.04777	5.093	0.01614	5.496	0.01882	5.418	0.01541
Diglycidyl Ether	metab_23741	1.012	3.902E-5	6.072E-5	3.699	0.0431	2.954	0.2836	2.621	0.002692	4.716	0.01085	2.634	0.003254
Acetovanillone	metab_20136	1.011	1.325E-5	4.891E-5	4.568	0.01475	3.965	0.05127	4.848	0.01165	4.443	0.01267	4.351	0.02071
N-Lauroyl Cysteine	metab_40122	1.011	1.325E-5	4.891E-5	4.951	0.01504	3.429	0.001173	3.577	0.00677	3.734	0.05846	3.544	0.0102
Ethyl 2-Amino-3-(4-Hydroxyphenyl)Propanoate	metab_25165	1.011	1.9E-5	4.891E-5	5.397	0.0157	5.306	0.01772	4.773	0.04142	6.576	0.005891	4.82	0.02539
Trp Gly	metab_7328	1.011	1.325E-5	4.891E-5	3.522	0.1904	4.293	0.06668	5.011	0.01066	5.628	0.008029	4.471	0.04431
Glu-Ala-Leu	metab_36878	1.011	1.325E-5	4.891E-5	3.033	0.003272	3.652	0.04428	4.704	0.01722	3.384	0.1601	4.451	0.03305
(5Z)-(15S)-11Alpha-Hydroxy-9,15-Dioxoprostanoate	metab_34463	1.011	1.325E-5	4.891E-5	3.557	0.07028	5.121	0.004097	5.376	0.01156	4.896	0.01762	5.302	0.01255
1-(2,4,6-Trimethylphenoxy)Propan-2-Amine	metab_39499	1.010	1.9E-5	4.891E-5	2.343	0.003261	2.266	0.001167	2.414	0.006745	4.174	0.02714	2.391	0.0308
2-[[(2E)-2-Hydroxyimino-4-Methylpentanoyl]Amino]-4-Methylpentanoic Acid	metab_22646	1.010	1.325E-5	4.891E-5	4.212	0.103	4.637	0.03439	5.813	0.01098	4.759	0.04187	5.317	0.02238
Mangicrocin	metab_32306	1.009	1.421E-5	4.891E-5	3.495	0.003274	5.158	0.04014	4.706	0.05042	3.634	0.01533	5.012	0.09884
Gly-Gln	metab_31859	1.009	1.325E-5	4.891E-5	4.315	0.05162	4.616	0.04101	5.449	0.01092	6.157	0.01092	5.349	0.0256
S-(9-Hydroxy-Pga2)-Glutathione	metab_35367	1.009	1.325E-5	4.891E-5	4.47	0.03316	5.163	0.01967	4.59	0.02148	2.732	0.9768	5.27	0.01282
Aldoxorubicin	metab_48092	1.009	1.325E-5	4.891E-5	5.558	0.009893	4.502	0.04825	4.792	0.03911	6.037	0.007423	4.026	0.1034
Estriol Succinate	metab_34185	1.009	2.411E-5	4.891E-5	3.676	0.159	5.042	0.01718	5.332	0.01392	5.269	0.01266	5.326	0.01371
Gln-Ile	metab_28151	1.008	1.325E-5	4.891E-5	4.394	0.04158	5.105	0.01669	5.327	0.02187	6.006	0.008958	4.645	0.0301
Gly-Ala-Thr	metab_31913	1.008	1.518E-5	4.891E-5	3.331	0.003274	3.254	0.001173	3.725	0.3332	5.379	0.03611	3.383	0.03609
Pro-His	metab_2858	1.008	8.229E-5	0.0001055	4.375	0.1106	4.288	0.1241	5.366	0.232	6.203	0.04022	4.52	0.2214
Tyr-Thr-Leu	metab_3625	1.008	1.9E-5	4.891E-5	4.846	0.001284	4.89	0.03766	6.108	0.02385	5.419	0.03054	5.591	0.01854
D-Sorbitol	metab_30304	1.008	0.0001977	0.0002239	4.793	0.0359	3.926	0.2088	3.689	0.2446	3.474	0.1768	3.288	0.00326

Ent-8-Iso Prostaglandin F2Alpha	metab_39826	1.008	3.441E-5	5.698E-5	4.254	0.05456	4.365	0.06594	4.61	0.04047	4.241	0.04611	3.908	0.1883
Galactaric Acid	metab_50435	1.008	3.483E-5	5.733E-5	4.994	0.02548	4.438	0.07719	4.108	0.1637	4.35	0.1054	3.564	0.2001
Thr Leu Asp Glu	metab_48853	1.008	2.075E-5	4.891E-5	5.133	0.01447	4.159	0.03899	4.956	0.028	5.839	0.02551	4.981	0.02412
Pa(6 Keto-Pgf1Alpha/19:2(10Z,13Z))	metab_43999	1.007	1.325E-5	4.891E-5	5.849	0.006393	5.387	0.01595	5.299	0.008188	4.149	0.09131	5.882	0.0107
Indacaterol-8-O-Glucuronide	metab_44385	1.007	2.53E-5	4.891E-5	5.59	0.01414	4.383	0.1147	4.374	0.0378	4.609	0.04851	3.907	0.1543
Neoandrographolide	metab_5470	1.007	1.518E-5	4.891E-5	6.074	0.01094	4.983	0.03279	5.051	0.03199	4.56	0.189	5.401	0.02378
Kaempferide 3-[Rhamnopyranosyl-(1->6)-Glucoside] 7-Rhamnoside	metab_22368	1.007	2.402E-5	4.891E-5	5.601	0.008124	4.158	0.001859	4.235	0.002698	4.24	0.006604	4.248	0.003261
Setipiprant	metab_50327	1.007	1.807E-5	4.891E-5	5.577	0.02574	5.988	0.01578	4.862	0.148	5.191	0.04129	5.503	0.05582
Cytidine	metab_29498	1.007	2.075E-5	4.891E-5	5.362	0.01679	5.439	0.01113	4.394	0.07381	5.073	0.008489	5.085	0.01503
Ala-Tyr	metab_28345	1.006	1.325E-5	4.891E-5	5.003	0.03462	5.175	0.01178	6.284	0.003647	6.776	0.007986	5.912	0.002363
Glu Thr Asp Arg	metab_39132	1.006	2.633E-5	4.987E-5	3.351	0.1401	4.752	0.03035	5.111	0.01262	3.403	0.1694	5.149	0.01555
Pro-Tyr-Ser	metab_37295	1.006	1.325E-5	4.891E-5	3.404	0.1403	3.734	0.03099	4.946	0.01131	4.99	0.01336	4.471	0.02457
Cynaroside A	metab_44600	1.006	1.325E-5	4.891E-5	3.281	0.1634	3.671	0.09413	4.469	0.02877	5.421	0.01307	3.977	0.0454
Val Leu Ser Asp	metab_16049	1.006	3.065E-5	5.374E-5	4.809	0.1121	5.898	0.02918	5.841	0.02082	4.815	0.07391	5.325	0.05617
Pro-Phe	metab_11573	1.006	2.53E-5	4.891E-5	4.018	0.04984	5.212	0.005602	6.084	0.004474	5.208	0.01195	5.688	0.008336
Tyr-Tyr-Lys	metab_19510	1.006	1.325E-5	4.891E-5	5.195	0.01507	4.487	0.05769	3.645	0.6754	5.419	0.01526	4.235	0.0683
Ile-Leu	metab_36633	1.005	1.325E-5	4.891E-5	4.994	0.01231	5.2	0.005076	6.385	0.0109	5.736	0.007121	6.086	0.007243
Ala Asp Phe Asp	metab_48301	1.005	2.49E-5	4.891E-5	4.045	0.04161	4.03	0.04142	4.687	0.01942	5.179	0.01055	4.928	0.009405
Homoharringtonine	metab_10395	1.005	1.629E-5	4.891E-5	3.935	0.1445	4.9	0.06976	5.033	0.05198	4.254	0.07891	5.647	0.02109
Pg(Pgj2/A-13:0)	metab_35735	1.005	1.325E-5	4.891E-5	4.207	0.09021	5.525	0.0199	4.948	0.02451	3.332	0.03361	5.345	0.01717
Enkephalin L	metab_26671	1.005	4.381E-5	6.533E-5	4.234	0.1011	4.274	0.1676	4.788	0.1302	6.049	0.02629	4.854	0.08909
Ixocarpalactone B	metab_45653	1.005	5.847E-5	8.037E-5	4.254	0.01379	5.137	0.01139	4.228	0.07498	4.135	0.1009	4.949	0.02334
Nivacortol	metab_38117	1.005	1.325E-5	4.891E-5	5.208	0.01353	4.545	0.01425	3.893	0.08169	5.554	0.00802	4.445	0.01996
3-Ethyl-5-Hydroxy-4,5-Dimethyl-Pyrrolin-2-One	metab_19793	1.005	1.325E-5	4.891E-5	2.95	0.1562	3.464	0.1023	4.538	0.009942	4.699	0.02027	4.186	0.02793
N-Acetyl-L-Arginine	metab_29975	1.005	0.0001361	0.0001611	5.262	0.01635	5.289	0.02676	4.495	0.156	5.273	0.02687	4.294	0.1358
Cefovecin	metab_32550	1.004	2.359E-5	4.891E-5	4.093	0.09712	4.889	0.02904	5.649	0.01899	4.065	0.003721	5.075	0.02241
Bicine	metab_28921	1.004	2.23E-5	4.891E-5	4.567	0.07136	4.632	0.01523	5.69	0.006677	6.452	0.003776	5.267	0.007167
Pinostrobin 5-Glucoside	metab_31932	1.004	3.241E-5	5.532E-5	5.998	0.01789	4.549	0.1785	3.965	0.006771	4.969	0.1467	4.08	0.1821
Leupeptin	metab_15012	1.004	2.075E-5	4.891E-5	5.744	0.06844	4.792	0.09739	4.141	0.215	5.328	0.02104	4.875	0.03165
(2S)-2-Amino-3-[3-[(2S)-2-Amino-3-Phenylpropanoyl]Oxyphenyl]Propanoic Acid	metab_36480	1.004	1.325E-5	4.891E-5	4.232	0.02805	4.051	0.02343	5.189	0.01187	5.876	0.006977	4.797	0.01056
Digitoxigenin	metab_41729	1.004	2.156E-5	4.891E-5	4.124	0.04385	4.072	0.0666	5.136	0.02023	4.854	0.0292	5.047	0.02278
Zaldaride	metab_39865	1.004	0.003405	0.003492	6.252	0.2208	5.825	1.384	4.66	0.6111	5.401	0.6696	6.017	0.01019
Fahfa(18:1/3:0)	metab_41609	1.003	1.325E-5	4.891E-5	5.287	0.06196	4.929	0.005715	5.965	0.02976	5.423	0.01659	5.656	0.01381
Phe Tyr	metab_35715	1.003	1.325E-5	4.891E-5	4.251	0.02924	4.042	0.04558	5.179	0.01411	5.644	0.007251	4.911	0.02116
Stachyoside A	metab_33558	1.003	2.298E-5	4.891E-5	4.292	0.03014	5.265	0.0184	4.268	0.0605	3.96	0.08117	4.543	0.03424

3-Methylbut-2-Enoyl-Coa	metab_29598	1.002	2.359E-5	4.891E-5	3.911	0.3047	5.496	0.02336	5.057	0.07804	3.603	0.006603	5.334	0.03314
Met Ser Ala	metab_2805	1.002	1.325E-5	4.891E-5	4.776	0.02306	4.932	0.009821	5.988	0.002048	5.349	0.007793	5.901	0.003611
Arg-Val-Lys	metab_618	1.002	1.325E-5	4.891E-5	5.231	0.01496	5.108	0.03466	4.653	0.09876	5.474	0.01768	5.329	0.03206
Fahfa(18:2/3:0)	metab_41724	1.002	1.325E-5	4.891E-5	5.924	0.01914	5.578	0.002148	6.577	0.01484	6.002	0.01078	6.33	0.007425
Tetrahydrodeoxycortisol	metab_14603	1.002	7.422E-5	9.684E-5	6.393	0.009635	4.728	0.08532	4.624	0.08091	5.902	0.01425	4.62	0.0718
Gladiatoside C1	metab_48945	1.001	1.325E-5	4.891E-5	4.397	0.02979	5.355	0.007608	4.617	0.03847	4.758	0.0346	5.104	0.009471
Mono(2-Ethyl-5-Hydroxyhexyl)Adipate	metab_42783	1.001	4.522E-5	6.656E-5	3.959	0.1695	2.999	0.1887	3.909	0.09004	4.678	0.02811	3.029	0.1728
Methyl Reserpate	metab_35556	1.001	1.325E-5	4.891E-5	4.614	0.02472	4.269	0.02386	5.119	0.00778	5.173	0.0109	4.197	0.01885
Pe(Pgf2Alpha/14:1(9Z))	metab_48449	1.000	1.325E-5	4.891E-5	5.862	0.008482	5.461	0.008058	5.316	0.01628	4.245	0.1709	5.981	0.01489
Tyr-Lys-Ile	metab_43836	1.000	2.156E-5	4.891E-5	4.26	0.023	3.004	0.03588	3.601	0.1094	4.472	0.02984	3.71	0.1004
8-Isoprostaglandin F1Alpha	metab_42506	1.000	1.421E-5	4.891E-5	5.237	0.01376	5.183	0.02365	4.3	0.03459	5.508	0.01084	4.844	0.01454
3-Hydroxyoctanoic Acid	metab_39574	1.000	1.325E-5	4.891E-5	5.668	0.002832	4.814	0.0111	3.822	0.03087	4.988	0.00635	4.415	0.005511

Table S14 Complete metabolite list

ID	Metabolite	Metab ID	level	Fragmentation Score	Theoretical Fragmentation Score	Library ID	Adducts	Formula	Retention time	KEGG Compound ID	Mode	M/Z	RSD	Score	CAS ID
pos_14	9-Octadecynoic Acid	metab_13	B(i)	67	0	LMFA01030455	M+H-H2O	C18H32O2	0.37218333333333	C08459	pos	263.23613014386	0.25226908897838	50.9	506-24-1;
pos_129	Methacholine	metab_128	B(i)	38.3	0	HMDB0015654	M+Na-H2O	C8H18NO2+	0.49476666666667	C07471	pos	165.11229800841	0.010656120458524	44.9	55-92-5
pos_134	Bedoradrine	metab_133	B(ii)	0	44.7	HMDB0248928	M+ACN+H	C24H32N2O5	0.49476666666667	-	pos	470.26917740764	0.14413784379654	43.8	-
pos_172	His-Gln-Lys	metab_171	B(i)	82	0	-	M+H	C17H29N7O5	0.5054	-	pos	412.22901054864	0.039765586829103	52.9	-
pos_288	Asp-Gly-Ile	metab_287	B(i)	45.4	0	-	M+H	C12H21N3O6	0.52686666666667	-	pos	304.14952121921	0.28978841908022	45.7	-
pos_332	L-Ornithine	metab_331	B(i)	97.8	0	HMDB0000214	M+H	C5H12N2O2	0.5428	C00077	pos	133.09679040354	0.002306823294076	57.7	70-26-8
pos_333	N,N-Dimethylacetoacetamide	metab_332	B(i)	37.8	0	-	M+NH4	C6H11NO2	0.5428	-	pos	147.11237048191	0.008345923294582	46.2	-
pos_338	Lys-Gly	metab_337	B(i)	65.8	0	HMDB0028951	M+H	C8H17N3O3	0.5428	-	pos	204.13373899418	0.010474421445232	51.5	7563/3/3
pos_346	Lys Gln	metab_345	B(i)	77.5	0	-	M+H	C11H22N4O4	0.5428	-	pos	275.17067860294	0.025919924433701	52.8	-
pos_348	Queuine	metab_347	B(ii)	0	59.9	HMDB0001495	M+NH4	C12H15N5O3	0.5428	C01449	pos	295.14914714301	0.033745066663298	48.3	72496-59-4
pos_349	Fructosyl-Lysine	metab_348	B(i)	88.9	0	HMDB0252495	M+H	C12H24N2O7	0.5428	C16488	pos	309.16466388153	0.088741872917221	56.7	-
pos_350	Arg Arg	metab_349	B(i)	82.7	0	-	M+H	C12H26N8O3	0.5428	-	pos	331.21911701762	0.070316212359248	53.4	-
pos_374	2,3-Diaminopyridine	metab_373	B(i)	49.7	0	-	M+H	C5H7N3	0.55341666666667	-	pos	110.0709595	0.019017849198549	48.4	-
pos_400	N6,N6,N6-Trimethyl-L-Lysine	metab_399	B(i)	91.3	0	HMDB0001325;PW_C001022	M+H	C9H20N2O2	0.55876666666667	C03793	pos	189.15920400338	0.016605833643289	56.9	-;19253-88-4
pos_446	6-Succinoaminopurine	metab_445	B(ii)	0	43.6	HMDB0013204	M+H	C9H9N5O3	0.56405	-	pos	236.07888775741	0.019755552809895	46.3	-
pos_492	Beta-L-Dioxolane-Cytidine	metab_491	B(ii)	0	47	HMDB0249137	M+	C8H11N3O4	0.56931666666667	-	pos	213.07421010773	0.009520872557033	47	-
pos_508	Ganoderic Acid Lm2	metab_507	B(i)	47	0	-	M+ACN+H	C30H42O7	0.56931666666667	-	pos	556.32961061228	0.038763900554784	45.3	-
pos_544	Serylarginine	metab_543	B(ii)	0	71.2	HMDB0029033	M+H	C9H19N5O4	0.57986666666667	-	pos	262.15012661781	0.013266347402777	52.4	13261-11-5
pos_548	Gly-Gly-Arg	metab_547	B(i)	74	0	-	M+H	C10H20N6O4	0.57986666666667	-	pos	289.16076266669	0.064138346355149	52	-
pos_555	N-[1-(1-Methyl-6-Oxopyridazin-3-Yl)Piperidin-4-Yl]-3-(Tetrazol-1-Yl)Propanamide	metab_554	B(i)	68.9	0	-	M+H	C14H20N8O2	0.57986666666667	-	pos	333.17641714556	0.23108790157662	49.2	-
pos_575	Thr Arg	metab_574	B(i)	63.6	0	-	M+H	C10H21N5O4	0.58515	-	pos	276.16543881746	0.047384364031415	50.3	-
pos_582	Arg Gln Gly	metab_581	B(i)	79.8	0	-	M+H	C13H25N7O5	0.58515	-	pos	360.19794293765	0.029107779605004	51.1	-
pos_584	Arg Glu Gln	metab_583	B(i)	58.8	0	-	M+H	C16H29N7O7	0.58515	-	pos	432.21967879495	0.10629516700332	49.3	-
pos_604	Serylasparagine	metab_603	B(ii)	0	44.2	HMDB0029034	M+H	C7H13N3O5	0.59043333333333	-	pos	220.09206961661	0.031969640889137	46.9	81466-41-3
pos_611	D-Fructose 1-Phosphate	metab_610	B(i)	42.2	0	HMDB0000399;HMDB0001076;HMDB0060467	M+Na	C6H13O9P	0.59043333333333	C01094;_	pos	283.01799057736	0.15526632442634	46.4	15978-08-2;_
pos_614	Gln-Thr-Ala	metab_613	B(i)	71.4	0	-	M+H	C12H22N4O6	0.59043333333333	-	pos	319.16055234196	0.22865681269926	51.6	-
pos_619	Arg-Val-Lys	metab_618	B(i)	66.4	0	-	M+H	C17H35N7O4	0.59043333333333	-	pos	402.28095776041	0.099899565273694	49.7	-
pos_626	Dl-Asparagine	metab_625	B(ii)	0	66.1	HMDB0251512	M+H	C4H8N2O3	0.5957	C16438	pos	133.06036524276	0.018803715156988	51.8	-
pos_629	Phosphocholine	metab_628	B(i)	60.1	0	HMDB0001565	M+H	C5H14NO4P	0.5957	C00588	pos	184.07278187456	0.097193725933901	50	107-73-3
pos_631	Citrulline	metab_630	B(ii)	0	49.7	HMDB0000904	M+NH4	C6H13N3O3	0.5957	C00327	pos	193.13089471451	0.019243086929285	46.6	372-75-8
pos_640	Gln Asn	metab_639	B(i)	85.5	0	-	M+H	C9H16N4O5	0.5957	-	pos	261.11824193075	0.039725971234096	54.7	-
pos_647	Arg-Val-Arg	metab_646	B(i)	87	0	-	M+H	C17H35N9O4	0.5957	-	pos	430.28694826449	0.15471841949444	54.1	-
pos_652	1-Aminocyclopropane-1-Carboxylate	metab_651	B(i)	45	0	HMDB0036458	M+H-H2O	C4H7NO2	0.60096666666667	C01234	pos	84.044138652362	0.030655682254247	47.8	22059-21-8
pos_653	2,4-Diaminobutyric Acid	metab_652	B(i)	47.6	0	HMDB0002362;HMDB0006284	M+H-H2O	C4H10N2O2	0.60096666666667	_;C03283	pos	101.07062853127	0.06506046768683	47.8	305-62-4;1758-80-1
pos_660	Gln Gly	metab_659	B(i)	77	0	-	M+H	C7H13N3O4	0.60096666666667	-	pos	204.09724718555	0.060917364988231	53.5	-
pos_674	2'-Deoxymugineic Acid	metab_673	B(ii)	0	76.6	HMDB0033909	M+H	C12H20N2O7	0.60096666666667	C15485;_	pos	305.13348068703	0.033827560443735	52.9	74235-24-8;_
pos_678	Thr Gly Arg	metab_677	B(i)	89.2	0	-	M+H	C12H24N6O5	0.60096666666667	-	pos	333.18686291835	0.041472027609708	54.3	-
pos_681	Arg-Ala-Asp	metab_680	B(i)	93.4	0	-	M+H	C13H24N6O6	0.60096666666667	-	pos	361.18181905508	0.098499994737209	56.5	-
pos_683	Ser Glu Arg	metab_682	B(i)	63.9	0	-	M+H	C14H26N6O7	0.60096666666667	-	pos	391.18924709146	0.067725495554892	47.6	-
pos_684	Glutamylleucylarginine	metab_683	B(ii)	0	65.1	HMDB0252758	M+H	C17H32N6O6	0.60096666666667	-	pos	417.24427844583	0.078927587807834	49.9	-
pos_696	Glucose 6-Phosphate	metab_695	B(i)	73.7	0	HMDB0001401;HMDB0246205;PW_C001083	M+H-2H2O	C6H13O9P	0.60623333333333	C00092;C02965	pos	225.01516702597	0.047375279738851	52.6	56-73-5;_

pos_698	Val Lys	metab_697	B(i)	57.3	0	-	M+H	C11H23N3O3	0.60623333333333	-	pos	246.18052205375	0.019948225659518	49.9	-
pos_700	D-Glucose 6-Phosphate	metab_699	B(i)	65.9	0	HMDB0001401	M+H	C6H13O9P	0.60623333333333	C00092	pos	261.03623441122	0.045315111049596	51.7	56-73-5
pos_709	Erythritol	metab_708	B(i)	49.4	0	HMDB0002994	M+H-2H2O	C4H10O4	0.6115	C00503	pos	87.043799713633	0.016358163315749	48.8	149-32-6
pos_710	Gamma-Aminobutyric Acid	metab_709	B(i)	99.1	0	HMDB0000112;PW_C000071	M+H	C4H9NO2	0.6115	C00334	pos	104.07030606012	0.014441793026909	58.7	1956/12/2;56-12-2
pos_718	O-Acetyl-L-Serine	metab_717	B(i)	67.7	0	HMDB0003011	M+H	C5H9NO4	0.61676666666667	C00979	pos	148.05998456376	0.032669071808883	52.2	5147-00-2
pos_746	1,2-Benzoquinone	metab_745	B(ii)	0	78.3	HMDB0012133	M+H	C6H4O2	0.63793333333333	C02351	pos	109.02807773675	0.047224210074042	54.4	583-63-1
pos_747	2-(Hydroxymethyl)-3-Methoxy-2H-Furan-5-One	metab_746	B(i)	74	0	-	M+H	C6H8O4	0.63793333333333	-	pos	145.04908986006	0.16833613999336	53.8	-
pos_748	3-Hydroxy-3-Methyl-Glutaric Acid	metab_747	B(i)	59.1	0	HMDB0000355	M+H	C6H10O5	0.63793333333333	C03761	pos	163.05959756027	0.029462855524395	50.8	503-49-1
pos_756	Turanose	metab_755	B(i)	41.8	0	HMDB0011740;HMDB0260157	M+H-2H2O	C12H22O11	0.63793333333333	C19636;_	pos	307.10146401152	0.16196606332494	45.1	547-25-1;_
pos_757	Sucrose	metab_756	B(i)	83.4	0	HMDB0000258;PW_C000177	M+H-H2O	C12H22O11	0.63793333333333	C00089	pos	325.11193131872	0.012288683137587	55.6	57-50-1
pos_763	Gly-Thr	metab_762	B(i)	76.1	0	HMDB0028851	M+H	C6H12N2O4	0.64326666666667	-	pos	177.08642482668	0.072488528916771	53.4	-
pos_779	2-Keto Valeric Acid	metab_778	B(i)	38.6	0	LMFA01060004	M+H-2H2O	C5H8O3	0.64855	C06255	pos	81.033247223402	0.013255325526237	46.4	0
pos_792	Gln Gln Ser	metab_791	B(i)	92.2	0	-	M+H	C13H23N5O7	0.64855	-	pos	362.16591670922	0.089369992248832	55.4	-
pos_824	Propanedione	metab_823	B(i)	69.9	0	HMDB0001167	M+H	C3H4O2	0.6591	C00546	pos	73.028185844831	0.012014992063946	50.5	78-98-8
pos_825	Alpha-Methylene-Gamma-Butyrolactone	metab_824	B(i)	51.2	0	HMDB0246945	M+H	C5H6O2	0.6591	C20578	pos	99.043760282609	0.1156297762107	49.2	-
pos_826	Creatine	metab_825	B(i)	90.3	0	HMDB0000064;PW_C000046	M+H	C4H9N3O2	0.6591	C00300	pos	132.07635216945	0.047268573579448	56.5	57-00-1
pos_834	Val Arg	metab_833	B(i)	97.2	0	-	M+H	C11H23N5O3	0.6591	-	pos	274.18659123303	0.019763680201594	57.8	-
pos_840	Gln Gln Gln	metab_839	B(i)	98.7	0	-	M+H	C15H26N6O7	0.6591	-	pos	403.19220831788	0.010063700796073	57.5	-
pos_846	Raffinose	metab_845	B(i)	39.2	0	HMDB0003213;PW_C001805	M+H	C18H32O16	0.6591	C00492	pos	505.1746552	0.040595155215584	45.2	512-69-6
pos_850	Palatinose	metab_849	B(i)	55.4	0	-;HMDB0256083	2M+H	C12H22O11	0.6591	C01742;_	pos	685.23704879337	0.028189684477117	48.1	13718-94-0;_
pos_862	Val-Ser-His	metab_861	B(i)	93.8	0	-	M+H	C14H23N5O5	0.66438333333333	-	pos	342.1760973	0.027562913338053	56.8	-
pos_872	Levulinic Acid	metab_871	B(i)	64.8	0	HMDB0000720;LMFA01060006	M+H	C5H8O3	0.66965	-	pos	117.05426105756	0.038899308840904	51.9	123-76-2;
pos_873	2,6-Diaminopimelic Acid	metab_872	B(i)	76.9	0	HMDB0001370	M+H	C7H14N2O4	0.66965	C00666	pos	191.10207690541	0.010874684015807	54.4	583-93-7
pos_880	Hydroxynefazodone	metab_879	B(ii)	0	58	HMDB0061040	M+NH4	C25H32ClN5O	0.66965	-	pos	503.2556166	0.039170904808102	43	-
pos_881	Asn Asn Leu Asn Val	metab_880	B(i)	51.2	0	-	M+H	C23H40N8O9	0.66965	-	pos	573.29714258862	0.034789496209506	47	-
pos_887	Gln Glu	metab_886	B(i)	89.9	0	-	M+H	C10H17N3O6	0.67498333333333	-	pos	276.11818882907	0.017536661064219	56.7	-
pos_894	Arg-Val-Asn	metab_893	B(i)	93.2	0	-	M+H	C15H29N7O5	0.67498333333333	-	pos	388.22927736145	0.084588523791117	55.4	-
pos_908	Ala-Glu	metab_907	B(i)	86.9	0	HMDB0028686	M+H	C8H14N2O5	0.68025	-	pos	219.09686388919	0.020218035138459	55.7	13187-90-1
pos_909	Ala-His	metab_908	B(i)	95.8	0	HMDB0028689	M+H	C9H14N4O3	0.68025	-	pos	227.11321014859	0.017945288731781	57	3253-17-6
pos_914	Asn Asp Ala	metab_913	B(i)	61.7	0	-	M+H	C11H18N4O7	0.68025	-	pos	319.12383584823	0.024861936451465	49.6	-
pos_918	Gln Ala Gln	metab_917	B(i)	92	0	-	M+H	C13H23N5O6	0.68025	-	pos	346.17103096845	0.02484684226421	55.1	-
pos_921	Deoxyloganin	metab_920	B(ii)	0	61.8	HMDB0251007	M+Na	C17H26O9	0.68025	C06071;_	pos	397.14403677431	0.18699408559586	48.1	26660-57-1;_;
pos_929	Niacinamide	metab_928	B(i)	85	0	HMDB0001406;PW_C001086	M+H	C6H6N2O	0.68558333333333	C00153	pos	123.0549331	0.10660402653309	55.6	98-92-0
pos_944	Glu Asp	metab_943	B(i)	49.3	0	-	M+H	C9H14N2O7	0.68558333333333	-	pos	263.08660915617	0.035663844152671	48	-
pos_972	2-Acetamido-1,4-Anhydro-2-Deoxy-D-Arabinitol	metab_971	B(i)	47	0	-	M+H	C7H13NO4	0.69086666666667	-	pos	176.09120009577	0.020583799420841	48.3	-
pos_978	(2Z,4'Z)-2-(5-Methylthio-4-Penten-2-Ynylidene)-1,6-Dioxaspiro[4.4]Non-3-Ene	metab_977	B(ii)	0	44.3	HMDB0032660	M+H	C13H14O2S	0.69086666666667	-	pos	235.0781956	0.11196719598807	45.5	-
pos_1002	Met Arg	metab_1001	B(i)	64.4	0	-	M+H	C11H23N5O3S	0.69613333333333	-	pos	306.15845747727	0.022595420122983	49.8	-
pos_1005	Ala Val Arg	metab_1004	B(i)	74.3	0	-	M+H	C14H28N6O4	0.69613333333333	-	pos	345.22352919514	0.027986367986849	52.8	-
pos_1012	Gly Pro Ala	metab_1011	B(i)	47.8	0	-	M+H	C10H17N3O4	0.70138333333333	-	pos	244.1284775	0.009649071123936	48.6	-
pos_1035	Leu Gly Gly	metab_1034	B(i)	48.3	0	-	M+H	C10H19N3O4	0.70665	-	pos	246.14426998072	0.005631625045726	48.1	-
pos_1068	Nipecotic Acid	metab_1067	B(i)	73.9	0	HMDB0255618	M+H	C6H11NO2	0.71191666666667	-	pos	130.08587986701	0.017443268734018	53.2	-
pos_1081	Pro-Ser	metab_1080	B(i)	40.7	0	HMDB0029026	M+H	C8H14N2O4	0.71191666666667	-	pos	203.10207327458	0.026485995139393	46.6	-
pos_1102	Ser Arg	metab_1101	B(i)	50.1	0	-	M+H	C9H19N5O4	0.71191666666667	-	pos	262.15027927903	0.02816248429267	48.8	-
pos_1145	Glu-Arg-Lys	metab_1144	B(i)	52.3	0	-	M+H	C17H33N7O6	0.71191666666667	-	pos	432.25568011072	0.033941079551198	47.4	-

pos_1201	His-Glu-Lys	metab_1200	B(i)	58.3	0	-	M+H	C17H28N6O6	0.71718333333333	-	pos	413.21397205203	0.037684719739311	48	-
pos_1214	Dimethyl Sulfoxide	metab_1213	B(i)	82.4	0	HMDB0002151	M+H	C2H6OS	0.72245	C11143	pos	79.020986979965	0.012193794787377	54.3	67-68-5
pos_1216	N2-Acetyl-L-Ornithine	metab_1215	B(i)	54.3	0	HMDB0003357	M+H	C7H14N2O3	0.72245	C00437	pos	175.10738478878	0.003991313216895	48.9	6205/8/9
pos_1230	Leu Val Arg	metab_1229	B(i)	90.2	0	-	M+H	C17H34N6O4	0.72245	-	pos	387.27038100601	0.027545926066901	56.2	-
pos_1231	Gln Leu Arg	metab_1230	B(i)	64.2	0	-	M+H	C17H33N7O5	0.72245	-	pos	416.26055390034	0.005626182630157	48.4	-
pos_1239	(2-Mercaptomethyl-3-Phenyl-Propionyl)-Glycine	metab_1238	B(ii)	0	48.2	HMDB0242473	M+CH3OH+Na	C12H15NO3S	0.72773333333333	C01619	pos	308.09023432986	0.042701898931991	45.4	-
pos_1243	Amylopectin	metab_1242	B(ii)	0	53.9	HMDB0003255	M+H-H2O	C30H52O26	0.72773333333333	C00317	pos	811.26840659898	0.023118204288496	48	9037-22-3
pos_1246	(8Ar)-2-Methyl-7-(2-Pyrazinylcarbonyl)Hexahydroimidazo[1,5-A]Pyrazin-3(2H)-One	metab_1245	B(i)	45.4	0	-	M+H	C12H15N5O2	0.73305	-	pos	262.1278077	0.022935280191904	46.3	-
pos_1252	1,3-Butadiene	metab_1251	B(ii)	0	57.2	HMDB0041792	M+NH4	C4H6	0.73833333333333	C16450	pos	72.080563989224	0.009657354610584	49.7	106-99-0
pos_1256	Val Ser Glu	metab_1255	B(i)	66.4	0	-	M+H	C13H23N3O7	0.73833333333333	-	pos	334.15995595774	0.093794068560481	50.4	-
pos_1268	Ala Tyr Gly	metab_1267	B(i)	45.1	0	-	M+H	C14H19N3O5	0.74893333333333	-	pos	310.13902150781	0.12036814648271	46.2	-
pos_1279	Calystegine B2	metab_1278	B(ii)	0	45.3	HMDB0038594	2M+H	C7H13NO4	0.75425	C10851	pos	351.17551105956	0.09543951516351	47.7	127414-85-1
pos_1282	Pelargonidin 3,5-Di-O-Glucoside	metab_1281	B(ii)	0	73.7	HMDB0302904	M+H	C27H31O15+	0.75425	C08725	pos	596.16886276855	0.051899393165046	48.5	-
pos_1296	Phe Arg	metab_1295	B(i)	89.3	0	-	M+H	C15H23N5O3	0.75955	-	pos	322.18657936551	0.01906454600899	56.2	-
pos_1306	S-(Formylmethyl)Glutathione	metab_1305	B(ii)	0	66.7	HMDB0060507	M+H-2H2O	C12H19N3O7S	0.7649	C14871	pos	314.08374914298	0.11371367793103	47.8	-;_
pos_1309	2-Hydroxyadipic Acid	metab_1308	B(i)	80	0	HMDB0000321;LMFA01170049	M+CH3OH+Na	C6H10O5	0.77018333333333	C02360	pos	217.06762882993	0.021691738257844	55	-;18294-85-4;
pos_1332	Wistin	metab_1331	B(ii)	0	53.9	HMDB0030869	M+ACN+Na	C23H24O10	0.78088333333333	-	pos	524.14817549231	0.11362085691665	44.3	19046-26-5
pos_1337	N-(2-Cyano-4,6-Difluorophenyl)Thiophene-2-Carboxamide	metab_1336	B(i)	38.2	0	-	M+H	C12H6F2N2OS	0.78623333333333	-	pos	265.02233591104	0.079078017811638	42.6	-
pos_1356	Gemcitabine	metab_1355	B(ii)	0	43.5	HMDB0014584	M+CH3OH+Na	C9H11F2N3O4	0.79686666666667	C07650	pos	318.08800907354	0.018835910308119	45.7	95058-81-4
pos_1361	L-Methionine	metab_1360	B(i)	98.9	0	HMDB0000696;PW_C000548	M+H	C5H11NO2S	0.80748333333333	C00073	pos	150.05785401139	0.015145432749118	58.3	63-68-3
pos_1375	Threonyllysine	metab_1374	B(ii)	0	42.7	HMDB0029066	M+Na-H2O	C10H21N3O4	0.85523333333333	-	pos	252.13156271244	0.082771093478898	45.3	23161-31-1
pos_1378	Tetramethylcurcumin	metab_1377	B(i)	40	0	-	M+CH3OH+Na	C25H28O6	0.85523333333333	-	pos	479.2081487	0.13103680768711	41.2	-
pos_1390	Verbascotetrose	metab_1389	B(ii)	0	50.3	HMDB0303021	M+H	C24H42O21	0.88185	-	pos	667.22699728497	0.011554351163738	48.3	-
pos_1391	Pectolinarin	metab_1390	B(ii)	0	49.3	HMDB0256193	M+ACN+Na	C29H34O15	0.88185	-	pos	686.20016975837	0.016961537039164	45	-
pos_1395	Flavone Base+3O,2Meo,O-Guaiacylglyceryl-Hex	metab_1394	B(i)	36.2	0	-	M+H	C33H36O16	0.88715	-	pos	689.20862980353	0.010084321191087	45	-
pos_1396	Nadh	metab_1395	B(ii)	0	61.8	HMDB0001487	M+CH3OH+Na	C21H29N7O14P2	0.88715	C00004	pos	720.13796965018	0.11085748011661	50.4	58-68-4
pos_1398	Nicotinamide Riboside	metab_1397	B(ii)	0	45.9	HMDB0000855	M+	C11H15N2O5+	0.89248333333333	C03150	pos	255.09703266212	0.065408943075898	46.7	1341-23-7
pos_1399	Gly-Leu-His	metab_1398	B(i)	83.7	0	-	M+Na	C14H23N5O4	0.89248333333333	-	pos	348.16314778744	0.029958032348127	52.6	-
pos_1406	Melezitose	metab_1405	B(i)	46.5	0	HMDB0011730	M+H-H2O	C18H32O16	0.89778333333333	C08243	pos	487.16409830651	0.002843536717487	47.4	597-12-6
pos_1483	3-Methoxypropylamine	metab_1482	B(ii)	0	70.1	HMDB0245920	M+H-H2O	C4H11NO	0.9617	-	pos	72.080557639284	0.011320376436727	52.5	-
pos_1511	Arg Tyr Arg	metab_1510	B(i)	90.3	0	-	M+H	C21H35N9O5	0.97235	-	pos	494.28184198781	0.13800229092377	55.1	-
pos_1520	A-L-Arabinofuranosyl-(1->3)-[A-L-Arabinofuranosyl-(1R5)]-L-Arabinose	metab_1519	B(ii)	0	43	HMDB0041223	2M+H	C15H26O13	0.97235	-	pos	829.27915645761	0.03031378114288	44.8	-
pos_1530	Ile-His	metab_1529	B(i)	93.1	0	HMDB0028909	M+H	C12H20N4O3	0.97765	-	pos	269.16008095135	0.0349430551389	56.6	-
pos_1558	Negletein 6-[Rhamnosyl-(1->2)-Fucoside]	metab_1557	B(ii)	0	51.7	HMDB0031991	M+Na	C28H32O13	0.98298333333333	C10023	pos	599.1774798	0.16836773676808	44.8	192224-98-9
pos_1575	Arg His	metab_1574	B(i)	80.2	0	-	M+H	C12H21N7O3	0.98828333333333	-	pos	312.17672665562	0.02641437793225	53.4	-
pos_1576	Arg Ser Val	metab_1575	B(i)	55.2	0	-	M+H	C14H28N6O5	0.98828333333333	-	pos	361.21834254748	0.022654730741477	49.1	-
pos_1579	Arg His Leu	metab_1578	B(i)	64	0	-	M+H	C18H32N8O4	0.98828333333333	-	pos	425.26064984745	0.057562514542585	50.6	-
pos_1596	Pro-Asn	metab_1595	B(i)	41.6	0	HMDB0029012	M+H	C9H15N3O4	0.99356666666667	-	pos	230.11292801288	0.025772342292798	46	-
pos_1605	Thr Val Arg	metab_1604	B(i)	82.6	0	-	M+H	C15H30N6O5	0.99356666666667	-	pos	375.23389750669	0.04213710373352	54.2	-
pos_1613	Cytosine	metab_1612	B(i)	67.3	0	HMDB0000630	M+H	C4H5N3O	0.99883333333333	C00380	pos	112.05021719316	0.014580802538611	51.7	71-30-7
pos_1637	Asn Ser Leu Arg	metab_1636	B(i)	54.8	0	-	M+H	C19H36N8O7	0.99883333333333	-	pos	489.27675754716	0.034360848977248	47.9	-
pos_1673	Cytosine Arabinoside Monophosphate	metab_1672	B(i)	53.8	0	-	M+H	C9H14N3O8P	1.0041166666667	-	pos	324.05820449923	0.016464830299632	49.3	-
pos_1679	Methyl 2-(3-Hydroxy-6-Oxoxanthen-9-Yl)Benzoate	metab_1678	B(i)	91.1	0	-	M+CH3OH+H	C21H14O5	1.0041166666667	-	pos	379.11997629267	0.072602009296862	52.4	-
pos_1683	Glu Lys Arg	metab_1682	B(i)	66.1	0	-	M+H	C17H33N7O6	1.0041166666667	-	pos	432.25551324801	0.1213423566522	50.8	-

pos_1712	Lotaustralin	metab_1711	B(i)	48.6	0	HMDB0033865	M+H	C11H19NO6	1.00955	C08334	pos	262.12786580275	0.004418260915013	48.2	534-67-8
pos_1718	Tyr-Arg	metab_1717	B(i)	96.9	0	HMDB0005768	M+H	C15H23N5O4	1.00955	C02993	pos	338.1813297	0.021532063719793	57.6	70904-56-2
pos_1755	Cysteinylglycine	metab_1754	B(i)	93.6	0	HMDB0000078;PW_C000055	M+H	C5H10N2O3S	1.0202166666667	C01419	pos	179.0479787	0.069120826456779	55.9	19246-18-5
pos_1758	Isoleucylarginine	metab_1757	B(i)	93.9	0	HMDB0028901	M+H	C12H25N5O3	1.0202166666667	-	pos	288.20216963489	0.015474106357155	57.2	-
pos_1765	(+/-)-2-Hydroxy-4-(Methylthio)Butanoic Acid	metab_1764	B(ii)	0	54.4	HMDB0037115	M+H-H2O	C5H10O3S	1.0255166666667	-	pos	133.03139596498	0.016286545697389	49.8	120-91-2
pos_1768	Asp Ser Gln Lys	metab_1767	B(i)	51.7	0	-	M+H	C18H32N6O9	1.0255166666667	-	pos	477.22895394905	0.011528513648519	47.6	-
pos_1772	Thiodiglycol	metab_1771	B(ii)	0	51.2	HMDB0042033	M+H	C4H10O2S	1.0308666666667	-	pos	123.04683718102	0.01899357792591	47.2	111-48-8
pos_1806	Pelargonidin 3-Sophoroside 5-Glucoside	metab_1805	B(ii)	0	52.3	HMDB0033687	M+H	C33H41O20+	1.0521	-	pos	758.22137030779	0.069611011802347	44.5	75093-88-8
pos_1847	Ser Ile Arg Arg	metab_1846	B(i)	62.7	0	-	M+H	C21H42N10O6	1.0786666666667	-	pos	531.33447164192	0.12028378513984	50	-
pos_1879	Lys Ile Ala	metab_1878	B(i)	58.9	0	-	M+H	C15H30N4O4	1.0893	-	pos	331.23306390408	0.023635033104041	48.4	-
pos_1888	Sinistrin	metab_1887	B(ii)	0	51.7	HMDB0258303	M+ACN+Na	C30H52O26	1.0893	-	pos	892.29485419039	0.0191233740729	47.4	-
pos_1905	1,2,2'-Triferuloylgentiobiose	metab_1904	B(ii)	0	62	HMDB0301722	M+ACN+H	C42H46O20	1.0947	-	pos	912.29085744861	0.044585145328152	48.9	-
pos_1917	Ala Gln Val Arg	metab_1916	B(i)	60.1	0	-	M+H	C19H36N8O6	1.1000333333333	-	pos	473.28166248212	0.018586650675756	50.3	-
pos_1922	Musabalbisiane A	metab_1921	B(ii)	0	42.6	HMDB0038680	2M+H	C23H28O12	1.1000333333333	-	pos	993.31764773871	0.023300071201517	42.3	143183-61-3
pos_1937	His Gln	metab_1936	B(i)	86.7	0	-	M+H	C11H17N5O4	1.1106333333333	-	pos	284.13447082388	0.036513000691463	54.4	-
pos_1948	Prolylhydroxyproline	metab_1947	B(i)	50.4	0	HMDB0006695	M+NH4	C10H16N2O4	1.11595	-	pos	246.14426708613	0.016270687765953	48.9	18684-24-7
pos_1973	Tyr-Asn	metab_1972	B(i)	92.3	0	HMDB0029100	M+H	C13H17N3O5	1.1319666666667	-	pos	296.12323225095	0.007330033299004	56.3	-
pos_1978	Ehretioside B	metab_1977	B(i)	82.1	0	-	M+H	C14H17NO7	1.1372666666667	-	pos	312.11027292445	0.012282712649988	52.9	-
pos_1983	Peanut Oil, Hydrogenated	metab_1982	B(ii)	0	65.1	HMDB0250835	M+Na-H2O	C30H45N9O5	1.1372666666667	-	pos	616.33921627158	0.11610221885149	47.3	-
pos_1984	2,5-Furandicarboxylic Acid	metab_1983	B(ii)	0	48.9	HMDB0004812	M+H-H2O	C6H4O5	1.1426	C20450	pos	139.00219313893	0.033767950931041	47.8	3238-40-2
pos_1990	N-Octyl-Beta-D-Thioglucopyranoside	metab_1989	B(ii)	0	44.8	HMDB0255216	M+H	C14H28O5S	1.1426	-	pos	309.174536	0.009273906169988	40.2	-
pos_1998	Synephrine	metab_1997	B(i)	54.5	0	HMDB0004826	M+NH4	C9H13NO2	1.1479666666667	C04548	pos	185.12793722002	0.034947458618617	48.3	1994/7/5;94-07-5
pos_2012	Arg Val Leu Asp	metab_2011	B(i)	39.3	0	-	M+H	C21H39N7O7	1.1479666666667	-	pos	502.29688243863	0.02343312332219	45.4	-
pos_2014	4-Hydroxyisoleucine	metab_2013	B(i)	57.1	0	-	M+H	C6H13NO3	1.1533	-	pos	148.09637940523	0.005533882955114	49.9	-
pos_2020	Val-Val-Arg	metab_2019	B(i)	87.3	0	-	M+H	C16H32N6O4	1.1533	-	pos	373.25473701562	0.038247655980272	55.2	-
pos_2034	Pro Phe Ala Thr Glu	metab_2033	B(i)	44.3	0	-	M+H	C26H37N5O9	1.164	-	pos	564.26064730315	0.037147794170959	44.8	-
pos_2036	Thr-Pro	metab_2035	B(i)	74	0	HMDB0029069	M+H	C9H16N2O4	1.1693333333333	-	pos	217.11771545121	0.011509612113946	53.5	46398-79-2
pos_2040	Devd-Cho	metab_2039	B(ii)	0	52	HMDB0251076	M+NH4	C18H28N4O10	1.1693333333333	-	pos	478.21307024839	0.061111817748694	48.2	-
pos_2045	(4R)-4-Hydroxy-L-Glutamic Acid	metab_2044	B(i)	43.6	0	HMDB0002273	M+H	C5H9NO5	1.18	C05947	pos	164.05367535305	0.004500011580606	45.1	2485-33-8
pos_2048	1-[(2R,3R,4S,5R)-3,4-Dihydroxy-5-(Hydroxymethyl)Oxolan-2-Yl]Oxypyrimidin-2-One	metab_2047	B(ii)	0	52.4	HMDB0257989	M+H-H2O	C9H12N2O6	1.18	-	pos	227.06564125288	0.020149816109344	49.6	-
pos_2056	1-Pyrroline-4-Hydroxy-2-Carboxylate	metab_2055	B(ii)	0	47.1	HMDB0002234	M+H	C5H7NO3	1.18535	C04282	pos	130.04950835167	0.005308231617990	48.2	-;9054-77-7
pos_2060	Leu-Gly-Lys	metab_2059	B(i)	92.9	0	-	M+H	C14H28N4O4	1.18535	-	pos	317.21744670357	0.018834438810065	56.3	-
pos_2066	Arecaidine	metab_2065	B(ii)	0	40.5	HMDB0030352	M+NH4	C7H11NO2	1.1906833333333	C10128	pos	159.11236372941	0.066450269945507	45.8	499-04-7
pos_2067	Pro-Ala	metab_2066	B(i)	36.7	0	HMDB0029010	M+NH4	C8H14N2O3	1.1906833333333	-	pos	204.13373361147	0.006586997806628	46.2	-
pos_2084	Nikethamide 1-Oxide	metab_2083	B(i)	35	0	-	M+ACN+Na	C10H15N2O2+	1.2067	-	pos	259.12817579306	0.026963050548653	44.4	-
pos_2086	Arg-Ile-Ser	metab_2085	B(i)	76	0	-	M+H	C15H30N6O5	1.2067	-	pos	375.23400367957	0.003816832188808	53.4	-
pos_2095	Arg Ser	metab_2094	B(i)	72.7	0	-	M+H	C9H19N5O4	1.2226666666667	-	pos	262.15026647558	0.006526364348570	53.1	-
pos_2115	Oxypurinol	metab_2114	B(i)	54.1	0	HMDB0000786;HMDB0002818;HMDB0000786	M+H	C5H4N4O2	1.2546833333333	C07599	pos	153.04027812393	0.031757856709414	48.8	2465-59-0
pos_2122	Tyr Gln Asn	metab_2121	B(i)	93.3	0	-	M+H	C18H25N5O7	1.2546833333333	-	pos	424.18137237859	0.010900812281855	55.9	-
pos_2126	Thr-Val	metab_2125	B(i)	82.6	0	HMDB0029074	M+H	C9H18N2O4	1.2601833333333	-	pos	219.13336961677	0.008501694896852	55.2	99032-17-4
pos_2130	Ile-Ser-His	metab_2129	B(i)	75.1	0	-	M+H	C15H25N5O5	1.2601833333333	-	pos	356.19179047176	0.050654557265568	51	-
pos_2136	Phe-Lys	metab_2135	B(i)	82.3	0	HMDB0029000	M+H	C15H23N3O3	1.2655333333333	-	pos	294.18041035364	0.023248904439757	54.4	6456-72-0
pos_2140	Leonurine	metab_2139	B(i)	49	0	HMDB0254014	M+K	C14H21N3O5	1.2708833333333	C16985	pos	350.10918831334	0.12770926478659	45.2	24697-74-3;_
pos_2142	Fludrocortisone Acetate	metab_2141	B(ii)	0	54.2	HMDB0252329	M+CH3OH+Na	C23H31FO6	1.2708833333333	C08186;_	pos	477.22899704672	0.009228399798355	46.6	514-36-3;_;

pos_2156	Glu-Lys-His	metab_2155	B(i)	61	0	-	M+H	C17H28N6O6	1.28695	-	pos	413.21313053851	0.021281125976708	49.6	-
pos_2157	Fluorogestone Acetate	metab_2156	B(ii)	0	41.4	HMDB0252379	M+CH3OH+Na	C23H31FO5	1.28695	-	pos	461.23363854892	0.035233491324017	43.1	-
pos_2162	Arg-Ala-Met	metab_2161	B(i)	85.8	0	-	M+H	C14H28N6O4S	1.2923	-	pos	377.19547654692	0.01450078495088	52.3	-
pos_2163	Retinoyl-Azt	metab_2162	B(ii)	0	62	HMDB0257175	M+H	C30H39N5O5	1.2923	-	pos	550.30663357842	0.21081204848213	46.5	-
pos_2179	Lys-Ser-Tyr	metab_2178	B(i)	80.2	0	-	M+H	C18H28N4O6	1.3241833333333	-	pos	397.20672726274	0.066141100962968	52.4	-
pos_2181	Tyr Asp	metab_2180	B(i)	54.1	0	-	M+H	C13H16N2O6	1.3295333333333	-	pos	297.10735134123	0.019285672101042	47.2	-
pos_2193	Glucose Lactate Glutamate	metab_2192	B(ii)	0	59.1	HMDB0252780	M+CH3OH+Na	C14H23NO13	1.3404	-	pos	468.13222480504	0.014581166937953	49	-
pos_2198	Benzofuran	metab_2197	B(ii)	0	41.3	HMDB0032929	M+H	C8H6O	1.3510166666667	C14512	pos	119.04883302837	0.015265195432007	47.2	271-89-6
pos_2199	Octopamine	metab_2198	B(i)	97.2	0	HMDB0004825	M+H-H2O	C8H11NO2	1.3510166666667	C04227	pos	136.07528906957	0.002409024148987	58.5	104-14-3
pos_2200	2-Hydroxycinnamic Acid	metab_2199	B(i)	83.5	0	HMDB0002641	M+H	C9H8O3	1.3510166666667	C01772	pos	165.05411161622	0.002972925409515	55.7	614-60-8
pos_2201	Ala-Val	metab_2200	B(i)	91.4	0	HMDB0028700	M+H	C8H16N2O3	1.3510166666667	-	pos	189.12283358806	0.008459618132759	57	3303-45-5
pos_2202	Leu Thr	metab_2201	B(i)	48.5	0	-	M+H	C10H20N2O4	1.3510166666667	-	pos	233.14894416754	0.0101806223014	48.5	-
pos_2207	2,3-Butanediol	metab_2206	B(i)	46.4	0	HMDB0003156	M+H-H2O	C4H10O2	1.3883	-	pos	73.064594019276	0.032005294570393	48.2	513-85-9
pos_2215	N-[5-Methyl-4-(4-Methylphenyl)-1,3-Thiazol-2-Yl]-1-Benzofuran-2-Carboxamide	metab_2214	B(i)	42.6	0	-	M+H	C20H16N2O2S	1.4042	-	pos	349.09702601715	0.16191174872371	42.7	-
pos_2217	Met-Ser	metab_2216	B(i)	45.3	0	HMDB0028982	M+H	C8H16N2O4S	1.4201333333333	-	pos	237.08976325071	0.008775308598611	47.5	-
pos_2224	Epigallocatechin	metab_2223	B(i)	85.8	0	HMDB0038361;HMDB0302404;LMPK12020004	M+H	C15H14O7	1.4630666666667	C12136;_	pos	307.08241564306	0.046062314094988	53.8	970-74-1;_;
pos_2227	Ethyl 2-Amino-3-Sulfanylpropanoate	metab_2226	B(ii)	0	48.3	HMDB0252040	M+H	C5H11NO2S	1.479	-	pos	150.05787361457	0.025720664877584	47.3	-
pos_2238	N-Fructosyl Tyrosine	metab_2237	B(i)	70.6	0	-	M+H	C15H21NO8	1.5003333333333	-	pos	344.13300583069	0.008423456907160	52	-
pos_2239	Ala-Tyr-Arg	metab_2238	B(i)	77.8	0	-	M+H	C18H28N6O5	1.5003333333333	-	pos	409.21803387227	0.044965476767426	52.8	-
pos_2245	Alclofenac	metab_2244	B(ii)	0	42.5	HMDB0248119	M+Na	C11H11ClO3	1.5110833333333	-	pos	249.02753127327	0.16185434238923	40.8	-
pos_2251	Desmethylastemizole	metab_2250	B(ii)	0	40.1	HMDB0061013	M+Na	C27H29FN4O	1.5163833333333	-	pos	467.22145768398	0.035213602184043	42.9	-
pos_2258	Arg Phe	metab_2257	B(i)	63.8	0	-	M+H	C15H23N5O3	1.5376833333333	-	pos	322.18648032701	0.035021016144842	50.8	-
pos_2261	Janthitrem C	metab_2260	B(ii)	0	41.4	HMDB0040684	M+Na-H2O	C37H47NO4	1.5430166666667	C20601;_	pos	574.32895184519	0.02730926098922	43.3	73561-91-8
pos_2269	Asp-Val	metab_2268	B(i)	88	0	HMDB0028766	M+H	C9H16N2O5	1.5857166666667	-	pos	233.11257929182	0.001420021082770	56.5	-
pos_2271	2-(1,3-Dioxoisoindol-2-Yl)-N-[(5-Methyl-1,2-Oxazol-3-Yl)Methyl]Acetamide	metab_2270	B(i)	46.7	0	-	M+H	C15H13N3O4	1.5912166666667	-	pos	300.09811526251	0.21347805269276	46	-
pos_2278	Asteltoxin	metab_2277	B(ii)	0	57.2	HMDB0029464	M+ACN+Na	C23H30O7	1.6498166666667	-	pos	482.21752134042	0.008432588555428	47.4	79663-49-3
pos_2282	Murametide	metab_2281	B(ii)	0	52.6	HMDB0254950	M+H-H2O	C20H34N4O11	1.7031	-	pos	489.22266060411	0.061978325420039	45.8	-
pos_2292	Butanal	metab_2291	B(i)	70.4	0	HMDB0003543	M+H	C4H8O	1.74575	C01412	pos	73.064590119471	0.003181368844612	53.4	123-72-8
pos_2306	Gln-Val-His	metab_2305	B(i)	61.3	0	-	M+H	C16H26N6O5	1.7670166666667	-	pos	383.20268100736	0.27598693700341	49	-
pos_2317	4,6-Dihydroxy-2-Quinolinecarboxylic Acid	metab_2316	B(ii)	0	42.4	HMDB0033528	M+H	C10H7NO4	1.8257	C08480	pos	206.04426366501	0.084526512203258	42.7	-
pos_2318	Gln-Met	metab_2317	B(i)	74.2	0	HMDB0028803	M+H	C10H19N3O4S	1.831	-	pos	278.11618917101	0.008698379174039	52.8	114659-59-5
pos_2322	Ser Asn Val	metab_2321	B(i)	74.4	0	-	M+H	C12H22N4O6	1.85225	-	pos	319.16028784249	0.025848022408459	52.8	-
pos_2342	N-(3,4-Difluorophenyl)-2-Methoxy-4-Methylpyrimidine-5-Carboxamide	metab_2341	B(i)	69.7	0	-	M+H	C13H11F2N3O2	1.9	-	pos	280.08966333128	0.081877220693176	50.7	-
pos_2363	Sodium Lauryl Sulfate	metab_2362	B(i)	79.2	0	-	M+Na	C12H25NaO4S	1.9157833333333	C11166	pos	311.12292501156	0.065685263946858	51.5	151-21-3
pos_2364	Val-Gly-Gln	metab_2363	B(i)	45.9	0	-	M+Na	C12H22N4O5	1.9157833333333	-	pos	325.14811413553	0.089939610613156	46.1	-
pos_2397	Ser-Met-Asn	metab_2396	B(i)	56.9	0	-	M+H	C12H22N4O6S	1.9210666666667	-	pos	351.13229473134	0.020208586020349	48.3	-
pos_2420	Val-His	metab_2419	B(i)	66.5	0	HMDB0029129	M+H	C11H18N4O3	1.9264333333333	-	pos	255.14479184958	0.0385374720199	51.2	13589-07-6
pos_2443	His-Ser-Ile	metab_2442	B(i)	84.1	0	-	M+H	C15H25N5O5	1.9264333333333	-	pos	356.19353873139	0.12375057577627	54.3	-
pos_2453	4-(4-Benzyl-3-Oxoquinoxalin-2-Yl)-N-(3-Ethoxypropyl)Piperazine-1-Carboxamide	metab_2452	B(i)	61.9	0	-	M+H	C25H31N5O3	1.9264333333333	-	pos	450.25457788442	0.2435241055401	46.6	-
pos_2489	Arg-Val	metab_2488	B(i)	82.5	0	HMDB0028722	M+H	C11H23N5O3	1.9317333333333	-	pos	274.18664643407	0.009911666613075	55	2896-20-0
pos_2495	Lysylphenylalanine	metab_2494	B(ii)	0	71.7	HMDB0028958	M+H	C15H23N3O3	1.9317333333333	-	pos	294.18046220128	0.017883164872316	51.8	6235-35-4
pos_2510	Gln Leu Asn	metab_2509	B(i)	51.4	0	-	M+H	C15H27N5O6	1.9317333333333	-	pos	374.20241517927	0.013764594038396	47	-
pos_2513	Val Ile Arg	metab_2512	B(i)	89	0	-	M+H	C17H34N6O4	1.9317333333333	-	pos	387.27039005795	0.010271279921716	56.5	-

pos_2518	Glu Ile Arg	metab_2517	B(i)	45.6	0	-	M+H	C17H32N6O6	1.9317333333333	-	pos	417.24457634505	0.014928560769709	44.6	-
pos_2528	2-Amino-2-Methylpropan-1-Ol	metab_2527	B(i)	74	0	HMDB0244974	M+H-H2O	C4H11NO	1.9370833333333	-	pos	72.080590136192	0.002076817258662	53.9	-
pos_2535	L-Alanyl-L-Proline	metab_2534	B(i)	36.1	0	HMDB0028695	M+NH4	C8H14N2O3	1.9370833333333	-	pos	204.1338721	0.040223149548767	44.8	13485-59-1
pos_2556	Ala-Val-Lys	metab_2555	B(i)	43	0	-	M+H	C14H28N4O4	1.9370833333333	-	pos	317.21746395659	0.027842440678263	45.3	-
pos_2565	Kanamycin	metab_2564	B(ii)	0	60	HMDB0015303	M+	C18H36N4O11	1.9370833333333	C00304;C01822	pos	484.23875489248	0.019405337589426	50.2	8063/7/8;8063-07-8;59-01-8
pos_2592	Adenosine Phosphate	metab_2591	B(i)	83	0	HMDB0000045	M+H	C10H14N5O7P	1.9423833333333	C00020	pos	348.06942571997	0.005310359345754	55.1	61-19-8
pos_2594	Lys-Val-Asn	metab_2593	B(i)	60.5	0	-	M+H	C15H29N5O5	1.9423833333333	-	pos	360.22308526392	0.053924103703111	49.2	-
pos_2596	2-(Benzylanilino)-1-[1,1'-Biphenyl]-4-Yl-1-Ethanone	metab_2595	B(i)	46	0	-	M+H	C27H23NO	1.9423833333333	-	pos	378.18683094681	0.10493260455218	43.1	-
pos_2597	Ile-Asn-His	metab_2596	B(i)	60	0	-	M+H	C16H26N6O5	1.9423833333333	-	pos	383.20287037545	0.072529923920311	48.5	-
pos_2598	Asn-Glu-Lys	metab_2597	B(i)	65.8	0	-	M+H	C15H27N5O7	1.9423833333333	-	pos	390.19752682394	0.062726869703448	49.8	-
pos_2602	Milbemycin Beta3	metab_2601	B(ii)	0	56	HMDB0254729	M+H-2H2O	C31H42O5	1.9423833333333	-	pos	459.29108627995	0.011024777227466	49.1	-
pos_2604	Gibberellin A4 Glucosyl Ester	metab_2603	B(ii)	0	56.4	HMDB0038610	M+H	C25H34O10	1.9423833333333	-	pos	495.21835509791	0.024209440653769	48.7	54788-52-2
pos_2619	Thiamet G	metab_2618	B(i)	41.8	0	-	M+H	C9H16N2O4S	1.9477333333333	-	pos	249.08981540699	0.003132029243164	45.6	-
pos_2621	L-Arogenate	metab_2620	B(ii)	0	54.6	HMDB0304400	M+ACN+H	C10H12NO5-	1.9477333333333	C00826;_	pos	268.10324881681	0.013298857438656	48.4	53078-86-7;_
pos_2623	Isoguanosine	metab_2622	B(i)	91.9	0	-	M+H	C10H13N5O5	1.9477333333333	C08432	pos	284.09817503401	0.012115971652285	56.9	1818-71-9
pos_2625	Ser Leu Gln	metab_2624	B(i)	70.7	0	-	M+H	C14H26N4O6	1.9477333333333	-	pos	347.19155702849	0.021821598396118	52	-
pos_2626	Asn Tyr Glu	metab_2625	B(i)	54.5	0	-	M+H	C18H24N4O8	1.9477333333333	-	pos	425.16548082869	0.061167468952309	47.9	-
pos_2639	Deoxyadenosine	metab_2638	B(i)	74.5	0	HMDB0000101;PW_C000068	M+H	C10H13N5O3	1.9530666666667	C00559	pos	252.10855336451	0.020793411351259	51.8	958-09-8
pos_2641	Tyr Val Asn	metab_2640	B(i)	42.7	0	-	M+H	C18H26N4O6	1.9530666666667	-	pos	395.19097650881	0.001751087462773	45.9	-
pos_2649	Lysylthreonine	metab_2648	B(ii)	0	61.8	HMDB0028961	M+CH3OH+Na	C10H21N3O4	1.9636833333333	-	pos	302.17001589939	0.029214540486116	37.7	97791-84-9
pos_2650	Guanosine 2'-Monophosphate	metab_2649	B(ii)	0	50	HMDB0240586	M+Na	C10H14N5O8P	1.9636833333333	-	pos	386.04622632517	0.22196184745906	46.9	130-50-7
pos_2666	Arg Asn Leu	metab_2665	B(i)	77	0	-	M+H	C16H31N7O5	1.98495	-	pos	402.24483928305	0.27914602326257	51.1	-
pos_2669	4A-Hydroxytetrahydrobiopterin	metab_2668	B(ii)	0	48.2	HMDB0002281	M+CH3OH+H	C9H15N5O4	1.99025	C15522;_	pos	290.14506986221	0.12845910297459	48	-;1379003-93-6;_;70110-58-
pos_2671	Deacetylchloronectrin	metab_2670	B(i)	65.5	0	-	M+H	C23H31ClO5	1.99025	-	pos	423.19620513868	0.027666239610112	44.8	-
pos_2690	17-Ethynyl-16-Fluoroestradiol	metab_2689	B(ii)	0	44.6	HMDB0244769	M+CH3OH+Na	C20H23FO2	2.0172166666667	-	pos	369.18597301381	0.14280597518164	43.6	-
pos_2692	Gly-Ala-Met	metab_2691	B(i)	40.8	0	-	M+H	C10H19N3O4S	2.0225666666667	-	pos	278.11589557421	0.016348492164877	44.5	-
pos_2697	Trp-Asn-Ser	metab_2696	B(i)	75.7	0	-	M+H	C18H23N5O6	2.0331833333333	-	pos	406.17449666947	0.009232496688682	52.8	-
pos_2698	Aeglin	metab_2697	B(ii)	0	51.5	HMDB0041415	M+ACN+H	C25H34O11	2.0331833333333	-	pos	552.23978146893	0.14481051403244	44.4	169626-23-7
pos_2703	Gly-Ile-Ser	metab_2702	B(i)	62.8	0	-	M+H-H2O	C11H21N3O5	2.0650666666667	-	pos	258.14425095156	0.059579941884229	51	-
pos_2708	3-Aminopicolinic Acid	metab_2707	B(ii)	0	44.4	HMDB0245821	M+K	C6H6N2O2	2.0864	-	pos	177.00658592856	0.19162673711697	46.5	-
pos_2712	Tyr Asn Glu	metab_2711	B(i)	72.9	0	-	M+H	C18H24N4O8	2.0920166666667	-	pos	425.16551627924	0.19079707733711	52.6	-
pos_2720	Arg-Asp-Tyr	metab_2719	B(i)	49.5	0	-	M+H	C19H28N6O7	2.1186166666667	-	pos	453.2056348	0.011570891265028	46.7	-
pos_2723	Thymolphthalein	metab_2722	B(ii)	0	43.4	HMDB0259056	M+H	C28H30O4	2.1239833333333	-	pos	431.22424625365	0.1961499179705	43.9	-
pos_2726	Ala Leu Gln	metab_2725	B(i)	76.6	0	-	M+H	C14H26N4O5	2.1293333333333	-	pos	331.19684724933	0.011176852664072	52.7	-
pos_2727	2-Hydroxy-4-Carboxybutyrylhistidylprolinamide	metab_2726	B(ii)	0	62.5	HMDB0251550	M+H	C16H23N5O6	2.1293333333333	-	pos	382.16890993607	0.29390331315691	48	-
pos_2728	Arg-Gln-Leu	metab_2727	B(i)	88.7	0	-	M+H	C17H33N7O5	2.1347	-	pos	416.26060146121	0.026761843717223	56.4	-
pos_2731	Arginyl-Gamma-Glutamate	metab_2730	B(ii)	0	59.1	HMDB0028723	M+H-H2O	C11H22N6O4	2.14005	-	pos	285.1660223	0.016869871697641	47.8	-
pos_2733	Dronedarone	metab_2732	B(i)	62.3	0	HMDB0251621	M+NH4	C31H44N2O5S	2.14005	-	pos	574.32902909958	0.27260283272515	48	-
pos_2752	Glu Phe Pro Leu Ser	metab_2751	B(i)	55.6	0	-	M+H	C28H41N5O9	2.2253	-	pos	592.29182757144	0.017787400538201	46.5	-
pos_2756	N-(2-Hydroxy-2-Phenylpropyl)-2-(4-Propan-2-Ylsulfanylphenyl)Acetamide	metab_2755	B(i)	42.9	0	-	M+H	C20H25NO2S	2.2575833333333	-	pos	344.16847700608	0.047525115809986	45.5	-
pos_2762	Asn-Ile-Asp	metab_2761	B(i)	79.6	0	-	M+H	C14H24N4O7	2.2842333333333	-	pos	361.1710117	0.018073638165147	53.5	-
pos_2764	Arginylvaline	metab_2763	B(ii)	0	54.5	HMDB0028722	M+H-H2O	C11H23N5O3	2.32685	-	pos	256.1762213	0.082336061078607	49.3	2896-20-0
pos_2765	Strictosidine	metab_2764	B(ii)	0	75.2	HMDB0304492	M+H-H2O	C27H34N2O9	2.3322	_;C03470	pos	513.22657494908	0.13775481264208	50.6	20824-29-7;_
pos_2771	Phe Gln	metab_2770	B(i)	89.3	0	-	M+H	C14H19N3O4	2.3804166666667	-	pos	294.14415962092	0.007851347125351	56.6	-

pos_2776	(3S,8As)-3-[(1R)-1-Hydroxyethyl]Hexahydropyrrolo[1,2-A]Pyrazine-1,4-Dione	metab_2775	B(i)	82.8	0	-	M+H	C9H14N2O3	2.41755	-	pos	199.10720275888	0.059542155975743	54.4	-
pos_2778	Ile-Ala-Asp	metab_2777	B(i)	50	0	-	M+H	C13H23N3O6	2.4282333333333	-	pos	318.16528596576	0.039788014444992	47.8	-
pos_2780	Ser-Ile-Lys	metab_2779	B(i)	51.2	0	-	M+H	C15H30N4O5	2.4442833333333	-	pos	347.22807489538	0.27342294043239	47.8	-
pos_2781	Thr Tyr	metab_2780	B(i)	85.9	0	-	M+H	C13H18N2O5	2.4549166666667	-	pos	283.12802185568	0.015146328974086	55.8	-
pos_2782	Ala-Leu-His	metab_2781	B(i)	37.7	0	-	M+ACN+H	C15H25N5O4	2.4549166666667	-	pos	381.22146527081	0.069846099136962	43.3	-
pos_2784	Arg-Ala-Leu	metab_2783	B(i)	93.6	0	-	M+H	C15H30N6O4	2.4815	-	pos	359.23908342827	0.020671592270369	56.7	-
pos_2785	6-Dimethylaminopurine	metab_2784	B(i)	53.1	0	HMDB0000473	M+H	C7H9N5	2.49745	-	pos	164.09260959761	0.067611538916964	48.3	938-55-6
pos_2787	Val Val Glu	metab_2786	B(i)	66.1	0	-	M+H	C15H27N3O6	2.50275	-	pos	346.19630866672	0.047183781206405	46.9	-
pos_2790	Asn Asp Val	metab_2789	B(i)	36.9	0	-	M+H	C13H22N4O7	2.5667666666667	-	pos	347.1553026	0.055765047499942	44.4	-
pos_2793	Ser Tyr Glu	metab_2792	B(i)	84.5	0	-	M+H	C17H23N3O8	2.5828166666667	-	pos	398.15466690308	0.025654792567937	52.8	-
pos_2794	2-(Acetylamino)-2-Deoxy-Alpha-D-Mannopyranose	metab_2793	B(ii)	0	40.6	HMDB0248228	M+ACN+H	C8H15NO6	2.5881666666667	C02711	pos	263.12310311194	0.095897716289371	46.5	-
pos_2795	2',3'-Isopropylideneadenosine	metab_2794	B(i)	55.7	0	-	M+NH4	C13H17N5O4	2.6041166666667	-	pos	325.16089815439	0.055268196062717	49	-
pos_2797	Arg-Gly	metab_2796	B(i)	62.8	0	HMDB0028709	M+H-H2O	C8H17N5O3	2.6147666666667	-	pos	214.12921228297	0.043401767353643	49.8	2418-67-9
pos_2799	Valaciclovir	metab_2798	B(ii)	0	53.9	HMDB0014716	M+NH4	C13H20N6O4	2.6201166666667	C07184	pos	342.18742885047	0.008116570486504	49.1	124832-27-5
pos_2804	N-Formylantimycic Acid Methyl Ester	metab_2803	B(i)	38.5	0	-	M+H	C13H16N2O6	2.6413666666667	-	pos	297.1073242	0.050365306703728	45.8	-
pos_2806	Met Ser Ala	metab_2805	B(i)	60.5	0	-	M+H	C11H21N3O5S	2.6680833333333	-	pos	308.12666063082	0.009972016427802	49.7	-
pos_2818	Herculin	metab_2817	B(ii)	0	44.3	HMDB0030275	M+Na	C16H29NO	2.7319666666667	-	pos	274.21184843666	0.04579380037339	44.5	_;
pos_2826	6Alpha,9Alpha-Difluoroprednisolone-17-Butyrate	metab_2825	B(ii)	0	65.4	HMDB0060797	M+Na-H2O	C25H32F2O6	2.7426	-	pos	471.19244484692	0.039557578077176	47.8	-
pos_2833	Indoline	metab_2832	B(i)	76.2	0	HMDB0253472	M+H	C8H9N	2.7850833333333	-	pos	120.08039433173	0.006691300638011	52.8	-
pos_2834	Cinnamic Acid	metab_2833	B(i)	82.5	0	HMDB0000567;HMDB0000930;HMDB0000567;HMDB0000930	M+H	C9H8O2	2.7850833333333	C10438	pos	149.05926650639	0.0045982157677072	55.5	621-82-9;102-94-3;140-10-3
pos_2837	Secologanate	metab_2836	B(ii)	0	54.6	HMDB0304481	M+Na	C16H22O10	2.7904	C01957;_	pos	397.11003855866	0.047140591200714	47	-;_
pos_2840	Benzoic Aldehyde	metab_2839	B(i)	90.8	0	HMDB0006115	M+H	C7H6O	2.8170333333333	C00261	pos	107.04888734644	0.0783803843088	56.2	100-52-7
pos_2841	Pyroglutamyltyrosine	metab_2840	B(i)	77	0	-	M+H	C14H16N2O5	2.8276666666667	-	pos	293.1124244	0.003875842681018	53.1	-
pos_2847	Glu Val Arg	metab_2846	B(i)	58.3	0	-	M+H-H2O	C16H30N6O6	2.8651166666667	-	pos	385.2182249	0.1159707631989	49.7	-
pos_2848	Tyr Gln Glu	metab_2847	B(i)	88.4	0	-	M+H	C19H26N4O8	2.87575	-	pos	439.1809777	0.13495920029734	53.8	-
pos_2853	Val Val Thr	metab_2852	B(i)	68.1	0	-	M+H	C14H27N3O5	2.9076833333333	-	pos	318.20152490411	0.038298509432322	51.1	-
pos_2859	Pro-His	metab_2858	B(i)	94	0	HMDB0029019	M+H	C11H16N4O3	2.9237166666667	-	pos	253.12898652079	0.008686454411959	56.8	-
pos_2860	Cyclo(His-Pro)	metab_2859	B(ii)	0	42.5	HMDB0250630	M+H	C11H14N4O2	2.9290333333333	-	pos	235.11828396474	0.025770467993034	46.1	-
pos_2862	3-Amino-4-Phenylbutanoic Acid	metab_2861	B(i)	39.8	0	-	M+NH4	C10H13NO2	2.9343166666667	-	pos	197.12799555829	0.29130223343489	45.2	-
pos_2865	L-Hydroxy Arginine	metab_2864	B(i)	35.1	0	-	M+H	C6H14N4O3	2.9396333333333	-	pos	191.1153568	0.087423541125901	43.8	-
pos_2868	Sambacin	metab_2867	B(ii)	0	56.8	HMDB0038883	M+H-H2O	C26H36O12	2.9396333333333	-	pos	523.21331739819	0.047143503069982	47.3	85562-86-3
pos_2871	Pteroside Z	metab_2870	B(ii)	0	53.2	HMDB0032587	M+CH3OH+H	C21H30O7	2.9608833333333	-	pos	427.22877351046	0.094913506125836	47.2	35943-37-4
pos_2872	L-Tert-Leucine	metab_2871	B(i)	37.1	0	-	M+ACN+Na	C6H13NO2	2.9662333333333	-	pos	195.10994197682	0.007298552072248	45.4	-
pos_2874	Dibutyryl Adenosine	metab_2873	B(ii)	0	61.5	HMDB0251178	M+ACN+H	C18H25N5O6	2.9715333333333	-	pos	449.21044237123	0.20071584325112	49.4	-
pos_2880	Ala-Arg-Leu	metab_2879	B(i)	79	0	-	M+H	C15H30N6O4	2.9875666666667	-	pos	359.23923663609	0.2302516649702	53.1	-
pos_2889	Leu-His	metab_2888	B(i)	95.3	0	HMDB0028931	M+H	C12H20N4O3	3.0142333333333	-	pos	269.16018919124	0.008118077819942	57.7	-
pos_2893	Fraxin	metab_2892	B(ii)	0	50.4	HMDB0252486	M+NH4	C16H18O10	3.0142333333333	_;C09266	pos	388.12746589593	0.002392352490130	45.4	524-30-1;_
pos_2900	Gln-Ile-Ser	metab_2899	B(i)	62.7	0	-	M+Na	C14H26N4O6	3.0355333333333	-	pos	369.17381990076	0.11932094688438	49.5	-
pos_2911	Myricatomentoside Ii	metab_2910	B(ii)	0	43.8	HMDB0031584	M+H	C27H34O11	3.0569333333333	-	pos	535.21678114687	0.015207036328111	45.4	191999-63-0
pos_2925	(E)-7-(4-Hydroxy-3-Methoxyphenyl)-1-Phenylhept-4-En-3-One	metab_2924	B(i)	40.1	0	-	M+Na	C20H22O3	3.0782333333333	-	pos	333.14568933443	0.12148234922795	43.7	-
pos_2961	His Ser Val	metab_2960	B(i)	63.8	0	-	M+H	C14H23N5O5	3.1157666666667	-	pos	342.17640173617	0.037762347201378	50.1	-
pos_2967	Pentostatin	metab_2966	B(ii)	0	45.6	HMDB0014692	2M+H	C11H16N4O4	3.1157666666667	_;C02267	pos	537.23884300194	0.022742406932621	44.7	53910-25-1;_
pos_2969	Cynarasaponin E	metab_2968	B(ii)	0	86.3	HMDB0039562	M+Na	C42H66O15	3.1157666666667	-	pos	833.43567844477	0.21905487218272	52.5	117804-11-2
pos_2970	7,8-Diaminopelargonate	metab_2969	B(ii)	0	50.2	HMDB0304236	M+H-H2O	C9H20N2O2	3.1210833333333	C01037	pos	171.14876606682	0.015704831264721	48.7	-

pos_2985	Alpha-L-Rhamnopyranosyl-(1->3)-Alpha-D-Galactopyranosyl-(1->3)-L-Fucose	metab_2984	B(ii)	0	40.4	HMDB0039741	M+H-2H2O	C18H32O14	3.1210833333333	-	pos	437.16153228765	0.0427749330781	43.2	71144-75-7
pos_2994	Ile Leu Arg	metab_2993	B(i)	84.3	0	-	M+H	C18H36N6O4	3.1264333333333	-	pos	401.28608143861	0.17592169660268	54.8	-
pos_3002	2-O-Methyladenosine	metab_3001	B(i)	56.9	0	HMDB0004326	M+H	C11H15N5O4	3.1317833333333	C04779	pos	282.11892212708	0.009090996742806	49.7	2140-79-6
pos_3028	Phe Lys Phe	metab_3027	B(i)	73.7	0	-	M+H	C24H32N4O4	3.1424333333333	-	pos	441.24447772053	0.036066677977204	48.4	-
pos_3048	Fuscaxanthone A	metab_3047	B(ii)	0	40.3	HMDB0304627	M+ACN+H	C29H32O6	3.15315	-	pos	518.25324459952	0.015930084948228	43.4	-
pos_3059	1-Methylguanosine	metab_3058	B(i)	94.5	0	HMDB0001563	M+Na	C11H15N5O5	3.1638	C04545	pos	320.09577672982	0.061856732481628	56.4	2140-65-0
pos_3098	Hordatine A	metab_3097	B(ii)	0	50.3	HMDB0030461	M+Na	C28H38N8O5	3.1747333333333	C08307	pos	589.28892305187	0.003984917496711	44.7	7073-64-5
pos_3140	6-O-(2-Methyl)-Butyrylhelenalin	metab_3139	B(i)	37	0	-	M+Na	C20H26O5	3.1906833333333	-	pos	369.16379322768	0.091220344846259	43.8	-
pos_3142	1-(2-Methylphenyl)Sulfonyl-4-[(4-Phenyl-1,2,4-Triazol-3-Yl)Methyl]Piperidine	metab_3141	B(i)	61.6	0	-	M+H	C21H24N4O2S	3.1906833333333	-	pos	397.17077672726	0.0079945061479015	47.3	-
pos_3163	23-Hydroxyphysalolactone	metab_3162	B(ii)	0	57.1	HMDB0031388	M+NH4	C28H39ClO9	3.196	-	pos	572.26599673438	0.017531282931751	43.8	118194-16-4
pos_3166	Vinorelbine Base	metab_3165	B(ii)	0	71.7	HMDB0259819	M+Na-H2O	C45H54N4O8	3.196	-	pos	783.37250497922	0.11701848912769	49.3	-
pos_3207	Ser-Arg-Ile	metab_3206	B(i)	43.7	0	-	M+H	C15H30N6O5	3.2066333333333	-	pos	375.23425263963	0.028363512528255	45.9	-
pos_3224	Gly-Arg-Thr	metab_3223	B(i)	37.5	0	-	M+H-H2O	C12H24N6O5	3.2119666666667	-	pos	315.17673714338	0.044855728193255	44.9	-
pos_3251	Arg-Ser-Tyr	metab_3250	B(i)	88	0	-	M+H	C18H28N6O6	3.2173	-	pos	425.21202164412	0.11440420220167	55.6	-
pos_3254	Val Glu Thr Glu	metab_3253	B(i)	65.4	0	-	M+H	C19H32N4O10	3.2173	-	pos	477.21811249907	0.030026613085514	50.9	-
pos_3268	Ala Asn Arg	metab_3267	B(i)	64.3	0	-	M+H	C13H25N7O5	3.2226	-	pos	360.19788402738	0.024362642868251	47.7	-
pos_3274	Guanine	metab_3273	B(i)	79.3	0	HMDB0000132;PW_C000086	M+H	C5H5N5O	3.2279333333333	C00242	pos	152.05629288929	0.030356071541356	53.9	73-40-5
pos_3277	Ser-Ile	metab_3276	B(i)	92.9	0	HMDB0029042	M+H	C9H18N2O4	3.2279333333333	-	pos	219.13340641187	0.009984874287832	57.8	91086-51-0
pos_3284	Ile Ser Glu	metab_3283	B(i)	71.9	0	-	M+H	C14H25N3O7	3.2279333333333	-	pos	348.17570016413	0.006558238363640	52.4	-
pos_3316	N-Docosahexaenoyl Tyrosine	metab_3315	B(ii)	0	58.6	HMDB0242024	M+Na	C31H41NO4	3.2332833333333	-	pos	514.29681305747	0.038617882315836	46.7	-
pos_3372	Ms 3	metab_3371	B(ii)	0	45.7	HMDB0036557	M+CH3OH+Na	C21H24O7	3.2438666666667	C01553	pos	443.16487200627	0.027923682757069	45.4	58265-74-0
pos_3408	3-Isopropyl-6-(2-Methyl-Propyl)-2,5-Piperazinedione	metab_3407	B(i)	56.1	0	-	M+H	C11H20N2O2	3.2545	-	pos	213.15924701421	0.025718406292875	50	-
pos_3421	Gln-Pro-His	metab_3420	B(i)	52.5	0	-	M+H	C16H24N6O5	3.2545	-	pos	381.18711025515	0.017511906143355	47.8	-
pos_3482	Ile Val Gln	metab_3481	B(i)	75.1	0	-	M+H	C16H30N4O5	3.2705666666667	-	pos	359.22795479027	0.009853589766065	52.6	-
pos_3528	1-Acetylproline	metab_3527	B(i)	50.9	0	HMDB0242126	M+H	C7H11NO3	3.2811833333333	-	pos	158.08076754559	0.028428842113741	48.5	-
pos_3532	Valylarginine	metab_3531	B(ii)	0	59.9	HMDB0029121	M+H	C11H23N5O3	3.2811833333333	-	pos	274.18655286739	0.044434760427535	49.3	37682-75-0
pos_3534	Aspartame	metab_3533	B(i)	65	0	HMDB0001894	M+H	C14H18N2O5	3.2811833333333	C11045	pos	295.12814991964	0.01547481893954	51.2	22839-47-0
pos_3537	Val Tyr Asp	metab_3536	B(i)	50.9	0	-	M+H	C18H25N3O7	3.2811833333333	-	pos	396.17604269538	0.011983652372367	47.8	-
pos_3574	Glu Leu Gly	metab_3573	B(i)	50.3	0	-	M+Na	C13H23N3O6	3.2917166666667	-	pos	340.1496077	0.089230517229821	47.1	-
pos_3613	[D-Lys3]-Ghrp-6	metab_3612	B(ii)	0	73	HMDB0243762	M+H-H2O	C49H63N13O6	3.2969833333333	-	pos	912.49850586913	0.09827901679686	49.7	-
pos_3620	Leu Asn Ala	metab_3619	B(i)	85.8	0	-	M+H	C13H24N4O5	3.3022333333333	-	pos	317.18116981067	0.055390939618017	54.3	-
pos_3623	Pro Glu Val	metab_3622	B(i)	54.6	0	-	M+H	C15H25N3O6	3.3022333333333	-	pos	344.18106868446	0.021880324138123	49.5	-
pos_3626	Tyr-Thr-Leu	metab_3625	B(i)	42.9	0	-	M+H-H2O	C19H29N3O6	3.3022333333333	-	pos	378.19938814414	0.073616635477107	42.7	-
pos_3671	N-Methyl-L-Proline	metab_3670	B(i)	60.2	0	-;HMDB0094696	M+NH4	C6H11NO2	3.3128	C22223;_	pos	147.11243334616	0.010699857070136	50	-;475-11-6
pos_3678	Dyphylline	metab_3677	B(i)	38.5	0	HMDB0014789	M+NH4	C10H14N4O4	3.3128	C07819	pos	272.13445672671	0.043092896761213	45.6	479-18-5
pos_3710	Lys-Val	metab_3709	B(i)	47.9	0	HMDB0028964	M+H	C11H23N3O3	3.3180666666667	-	pos	246.18060764989	0.035166084673143	47.8	20556-11-0
pos_3741	Asn-Ser-Leu	metab_3740	B(i)	80.8	0	-	M+H	C13H24N4O6	3.3233666666667	-	pos	333.17527979012	0.047972660747816	51.9	-
pos_3760	N-Methyl-D-Aspartic Acid	metab_3759	B(i)	56.2	0	HMDB0002393	M+H	C5H9NO4	3.3286333333333	C12269	pos	148.0600691	0.065410566676156	49.4	-;6384-92-5
pos_3766	His-Gly	metab_3765	B(i)	38.8	0	HMDB0028885	M+NH4	C8H12N4O3	3.3286333333333	-	pos	230.12627331751	0.004043693819152	44.3	2578-58-7
pos_3782	Arg Leu Val Glu	metab_3781	B(i)	61.9	0	-	M+H	C22H41N7O7	3.3286333333333	-	pos	516.31261395919	0.058936863476845	49.4	-
pos_3818	Gln Ile	metab_3817	B(i)	89.4	0	-	M+H	C11H21N3O4	3.3392333333333	-	pos	260.15983962208	0.020944882469018	56.6	-
pos_3823	Senecionine N-Oxide	metab_3822	B(ii)	0	49.8	HMDB0258230	M+H-2H2O	C18H25NO6	3.3392333333333	C15612;_	pos	316.15733760318	0.089841915787665	44.5	-;_
pos_3825	Tyr Pro Gly	metab_3824	B(i)	82.9	0	-	M+H	C16H21N3O5	3.3392333333333	-	pos	336.1545113	0.021741222402798	55	-
pos_3835	Bis-Ferulamidobutane	metab_3834	B(i)	39.6	0	-	M+NH4	C24H28N2O6	3.3392333333333	-	pos	458.22433231856	0.014627983668288	44.5	-

pos_3899	Diglykokoll	metab_3898	B(i)	57.9	0	-	M+H	C4H7NO4	3.3550333333333	-	pos	134.0444766	0.011380042199267	50	-
pos_3917	Gln Tyr Gln	metab_3916	B(i)	37.4	0	-	M+H	C19H27N5O7	3.3550333333333	-	pos	438.19721806415	0.033333767762774	45.9	-
pos_3932	Phe-Ala	metab_3931	B(i)	91.5	0	HMDB0028988	M+H	C12H16N2O3	3.3603	-	pos	237.12282152942	0.006017153068092	57	3918-87-4
pos_3944	Kinetensin 1-3	metab_3943	B(ii)	0	61.4	HMDB0012983	M+H-H2O	C15H30N6O4	3.3603	-	pos	341.22874739066	0.077084137403447	49.9	138482-55-0
pos_3992	5-Aminovaleric Acid	metab_3991	B(i)	43.3	0	HMDB0003355	M+H	C5H11NO2	3.3708666666667	C00431	pos	118.08597240074	0.049321249697599	47.1	660-88-8
pos_4017	Ala Leu	metab_4016	B(i)	66.3	0	-	M+H	C9H18N2O3	3.3761333333333	-	pos	203.13850436892	0.016935093814386	52.5	-
pos_4035	Secoeremopetasitolide B	metab_4034	B(ii)	0	47.8	HMDB0041363	M+H	C21H30O7	3.3761333333333	-	pos	395.20281184212	0.045141644738313	45.7	178402-71-6
pos_4068	7,8-Dihydro-3B,6A-Dihydroxy-Alpha-Ionol 9-[Apiosyl-(1->6)-Glucoside]	metab_4067	B(ii)	0	41.7	HMDB0041579	M+	C24H42O12	3.3814	-	pos	522.2675934	0.050733337137741	45.8	177261-73-3
pos_4098	Gymnodimine	metab_4097	B(ii)	0	61.6	HMDB0041430	M+Na	C32H45NO4	3.3866833333333	C20025;_	pos	530.32827787063	0.025238063261405	46.8	173792-58-0
pos_4107	Asparagine Ethyl Ester	metab_4106	B(i)	84.5	0	-	M+H	C6H12N2O3	3.39195	-	pos	161.09168342789	0.043655097889158	54.9	-
pos_4135	Ile-Gly	metab_4134	B(i)	83.2	0	HMDB0028907	M+H-H2O	C8H16N2O3	3.3972333333333	-	pos	171.11239808699	0.018811979110805	55.1	-
pos_4136	Glycyl-L-Leucine	metab_4135	B(i)	98.9	0	HMDB0000759	M+H	C8H16N2O3	3.3972333333333	C02155	pos	189.12288706541	0.014087267273416	58.8	869-19-2
pos_4163	Lys-Met-Asn	metab_4162	B(i)	48.8	0	-	M+H	C15H29N5O5S	3.4025	-	pos	392.1969365	0.032836542670128	41	-
pos_4209	N2,N2-Dimethylguanosine	metab_4208	B(i)	89.9	0	HMDB0004824	M+H	C12H17N5O5	3.4131166666667	-	pos	312.12965494457	0.028120278020312	55.3	2140-67-2
pos_4210	Pro Arg Gly	metab_4209	B(i)	85.1	0	-	M+H	C13H24N6O4	3.4131166666667	-	pos	329.19234172983	0.01490428923662	54.7	-
pos_4220	Glu Tyr Asn Lys	metab_4219	B(i)	53.4	0	-	M+H	C24H36N6O9	3.4131166666667	-	pos	553.25918651975	0.044690372146943	48.9	-
pos_4226	Ile-Ile	metab_4225	B(i)	85.7	0	HMDB0028910	M+H-H2O	C12H24N2O3	3.41845	-	pos	227.17487846385	0.023382989684257	56.1	-
pos_4227	Ethyl N-[(E)-1-(3,5-Dimethylfuran-2-[illegible]	metab_4226	B(i)	35.6	0	-	M+CH3OH+Na	C11H16N2O3	3.41845	-	pos	279.1332545	0.016016033872302	44.8	-
pos_4235	Val Ile Glu	metab_4234	B(i)	94.6	0	-	M+H	C16H29N3O6	3.41845	-	pos	360.21199643596	0.040428204766038	57.9	-
pos_4236	Pro-Gln-His	metab_4235	B(i)	95	0	-	M+H	C16H24N6O5	3.41845	-	pos	381.18713502049	0.02800381977508	57.8	-
pos_4395	Pro-Tyr-Gln	metab_4394	B(i)	83.9	0	-	M+H	C19H26N4O6	3.4502166666667	-	pos	407.19171890941	0.042133472964781	53.1	-
pos_4426	Leu Leu Gln	metab_4425	B(i)	94.5	0	-	M+H	C17H32N4O5	3.4554833333333	-	pos	373.24345380899	0.005486748609645	57.3	-
pos_4461	Val Ile Lys	metab_4460	B(i)	61.2	0	-	M+H	C17H34N4O4	3.4607833333333	-	pos	359.26435367215	0.020557087728876	50.4	-
pos_4473	Gly-Gln-Ile	metab_4472	B(i)	59.9	0	-	M+H	C13H24N4O5	3.4660833333333	-	pos	317.18109353124	0.018054677368941	45.7	-
pos_4479	Met Ile Gln	metab_4478	B(i)	53.9	0	-	M+H	C16H30N4O5S	3.4660833333333	-	pos	391.19971804016	0.059036533292971	45.5	-
pos_4498	Hydroxyoxazolone	metab_4497	B(ii)	0	43.1	HMDB0253283	M+CH3OH+H	C3H3NO3	3.4713833333333	-	pos	134.04444975988	0.067259503990241	47	-
pos_4508	Leu Pro Gln	metab_4507	B(i)	81.6	0	-	M+H	C16H28N4O5	3.4713833333333	-	pos	357.21216050801	0.013354933837769	54.9	-
pos_4531	Leu Val	metab_4530	B(i)	73.7	0	-	M+H	C11H22N2O3	3.4766666666667	-	pos	231.16966127699	0.009376670343580	53.5	-
pos_4536	2-({2-[(Tert-Butoxycarbonyl)Amino]Propanoyl}Amino)-3-Phenylpropanoic Acid	metab_4535	B(i)	44.1	0	-	M+H-2H2O	C17H24N2O5	3.4766666666667	-	pos	301.15166707019	0.14226603934059	45.7	-
pos_4539	10-Gingerol	metab_4538	B(i)	36.4	0	-	M+	C21H34O4	3.4766666666667	-	pos	350.2465255	0.24208445017688	43	-
pos_4645	Phenylalanylarginine	metab_4644	B(ii)	0	74.3	HMDB0028989	M+H	C15H23N5O3	3.4977333333333	-	pos	322.18650096555	0.001857410272515	51.7	1238-09-1
pos_4660	His Thr Lys Lys	metab_4659	B(i)	40.8	0	-	M+H	C22H40N8O6	3.4977333333333	-	pos	513.31294698698	0.014855467638251	44.3	-
pos_4676	Leu Pro Ala	metab_4675	B(i)	95.7	0	-	M+H	C14H25N3O4	3.5030166666667	-	pos	300.19069125434	0.019752411121597	57.3	-
pos_4707	Phe Asp Glu	metab_4706	B(i)	93.6	0	-	M+H	C18H23N3O8	3.5082833333333	-	pos	410.15472491757	0.003392554036193	55.2	-
pos_4760	Doxazosin	metab_4759	B(ii)	0	54.7	HMDB0014728	M+Na	C23H25N5O5	3.5189	C06970	pos	474.17192208023	0.039754664509229	48.3	74191-85-8
pos_4800	Lysyl-Lysine	metab_4799	B(ii)	0	52.7	HMDB0304785	M+ACN+H	C12H26N4O3	3.5295166666667	-	pos	316.23354267312	0.006885136450017	49.1	-
pos_4942	Thr Val Met	metab_4941	B(i)	82	0	-	M+H	C14H27N3O5S	3.5559	-	pos	350.17314390247	0.11010523125634	51.5	-
pos_4949	Lys Asp Leu Glu	metab_4948	B(i)	40.3	0	-	M+H	C21H37N5O9	3.5559	-	pos	504.26494588497	0.074627515823004	44.7	-
pos_4951	Val Arg Ala Asp Leu	metab_4950	B(i)	47.7	0	-	M+H	C24H44N8O8	3.5559	-	pos	573.33360487809	0.026972621250825	46	-
pos_4969	Ile Pro Glu	metab_4968	B(i)	51.4	0	-	M+H	C16H27N3O6	3.5612	-	pos	358.19643444147	0.041205455482272	46.7	-
pos_4972	Gln-Gln-Leu	metab_4971	B(i)	90.5	0	-	M+H	C16H29N5O6	3.5612	-	pos	388.21781805116	0.022131533483557	55	-
pos_4982	Kodia-Pc	metab_4981	B(i)	39.1	0	-	M+H	C32H58NO11P	3.5612	-	pos	664.37583225646	0.007487939291690	44.9	-
pos_5003	Ser-Leu-Pro	metab_5002	B(i)	57.1	0	-	M+H	C14H25N3O5	3.5718166666667	-	pos	316.18567467325	0.027595275515113	48.9	-
pos_5017	Pentaethylene Glycol	metab_5016	B(i)	77.4	0	HMDB0256256	M+H	C10H22O6	3.5771166666667	-	pos	239.14830509495	0.063403975629317	54.4	-

pos_5035	Ulimorelin	metab_5034	B(ii)	0	56.3	HMDB0259396	M+	C30H39FN4O4	3.5771166666667	-	pos	538.29574537399	0.12378331603708	46.8	-
pos_5040	Formyllysine	metab_5039	B(ii)	0	40.1	HMDB0252452	M+H-H2O	C7H14N2O3	3.5824666666667	-	pos	157.09673909807	0.013139223038382	46.3	-
pos_5067	Methionine Ethyl Ester	metab_5066	B(i)	94	0	-	M+H	C7H15NO2S	3.5877333333333	-	pos	178.08918495957	0.039878453577892	56.7	-
pos_5086	Lysylproline	metab_5085	B(ii)	0	45.3	HMDB0028959	M+H	C11H21N3O3	3.5930833333333	-	pos	244.1649503	0.039619931707098	46.1	52766-27-5
pos_5155	Val Leu Asp Glu	metab_5154	B(i)	54.7	0	-	M+H	C20H34N4O9	3.6037333333333	-	pos	475.23544844852	0.11480472820603	47	-
pos_5166	Leucylproline	metab_5165	B(i)	87.7	0	HMDB0011175	M+H	C11H20N2O3	3.6090333333333	-	pos	229.15409717377	0.013032305062939	56.5	6403-35-6
pos_5183	7-O-Succinyl Macrolactin A	metab_5182	B(ii)	0	88.4	HMDB0247277	M+NH4	C28H38O8	3.6090333333333	-	pos	520.28666096381	0.009572493772906	53.8	-
pos_5231	Neryl Rhamnosyl-Glucoside	metab_5230	B(ii)	0	50.3	HMDB0029349	M+H-H2O	C22H38O10	3.61965	-	pos	445.2393654	0.015301756003461	44.8	84534-31-6
pos_5240	Myelopeptides	metab_5239	B(ii)	0	51.5	HMDB0260304	M+ACN+Na	C35H48N6O8	3.61965	-	pos	744.36556507755	0.12487382118324	47	-
pos_5273	Ala Phe Thr	metab_5272	B(i)	87	0	-	M+H	C16H23N3O5	3.6303333333333	-	pos	338.17083875952	0.011331027974182	56.1	-
pos_5302	Chapelieric Acid	metab_5301	B(i)	50	0	-	M+H	C25H26O6	3.6356333333333	-	pos	423.18079267905	0.065029219625616	45.2	-
pos_5325	Asn-Glu-Ile	metab_5324	B(i)	80.4	0	-	M+H	C15H26N4O7	3.6409333333333	-	pos	375.18650992371	0.018603245784308	54.1	-
pos_5346	Val-Ala	metab_5345	B(i)	82.4	0	HMDB0029120	M+H-H2O	C8H16N2O3	3.6462166666667	-	pos	171.11239581316	0.041080330590601	54.8	27493-61-4
pos_5352	Gln Phe	metab_5351	B(i)	94.4	0	-	M+H	C14H19N3O4	3.6462166666667	-	pos	294.1440834	0.005233143273816	56.8	-
pos_5360	Val Tyr Arg	metab_5359	B(i)	69.2	0	-	M+H	C20H32N6O5	3.6462166666667	-	pos	437.25533132055	0.041462892925382	48.3	-
pos_5422	7,8-Dihydrovomifoliol 9-[Apiosyl-(1->6)-Glucoside]	metab_5421	B(ii)	0	54.6	HMDB0029771	M+Na	C24H40O12	3.6567833333333	-	pos	543.23974965886	0.024627773276449	47.8	177261-70-0
pos_5457	Gly Glu Val	metab_5456	B(i)	51	0	-	M+H-H2O	C12H21N3O6	3.6674166666667	-	pos	286.13911831548	0.018004914062494	47.9	-
pos_5471	Neoandrographolide	metab_5470	B(ii)	0	57.4	HMDB0255516	M+NH4	C26H40O8	3.6674166666667	-	pos	498.30230148527	0.019475117931716	47.1	-
pos_5486	Ile-Tyr	metab_5485	B(i)	74.4	0	HMDB0028919	M+H	C15H22N2O4	3.6727	-	pos	295.16454627621	0.024801681798694	53.7	-
pos_5504	Asp Phe Ser Ala Thr	metab_5503	B(i)	43	0	-	M+H	C23H33N5O10	3.6727	-	pos	540.22902941344	0.060221594330558	44.3	-
pos_5511	Erythrono-1,4-Lactone	metab_5510	B(i)	72.1	0	HMDB0000349	M+NH4	C4H6O4	3.67825	-	pos	136.06142610556	0.042572525790449	51.9	17675-99-9
pos_5517	5'-Deoxy-5'-Methylthioadenosine	metab_5516	B(i)	96.3	0	HMDB0001173	M+H	C11H15N5O3S	3.67825	C00170	pos	298.09613622305	0.017736504362968	57.6	2457-80-9
pos_5541	Thr-Gly	metab_5540	B(i)	49.1	0	HMDB0029061	M+H-H2O	C6H12N2O4	3.6835833333333	-	pos	159.07601166271	0.061207100592278	48.1	686-44-2
pos_5556	20,26-Dihydroxyecdysone	metab_5555	B(ii)	0	54.2	HMDB0245570	M+Na-H2O	C27H44O8	3.6835833333333	C16500;_	pos	501.27908058402	0.13358234282401	45.8	19458-46-9;_;
pos_5578	Bonactin	metab_5577	B(i)	42.3	0	-	M+CH3OH+Na	C21H36O7	3.6888833333333	-	pos	455.25993143147	0.11597484793009	43.9	-
pos_5582	Oleuroside	metab_5581	B(ii)	0	57.1	HMDB0035352	M+NH4	C25H32O13	3.6888833333333	-	pos	558.21811826638	0.13781755241433	49.9	116383-31-4
pos_5590	N-Ethyl Trans-2-Cis-6-Nonadienamide	metab_5589	B(ii)	0	40.3	HMDB0032273	M+NH4	C11H19NO	3.6941666666667	C06551	pos	199.18003172865	0.023502500577647	46.5	608514-56-3;
pos_5603	Val Gln Met	metab_5602	B(i)	79.3	0	-	M+H	C15H28N4O5S	3.6941666666667	-	pos	377.18442806309	0.023846441685097	53.8	-
pos_5637	Cyclocalamin	metab_5636	B(ii)	0	61.7	HMDB0035863	M+ACN+H	C27H34O9	3.6995166666667	-	pos	544.24994174057	0.12181080095711	46.1	74751-39-6
pos_5652	His-Arg-Arg	metab_5651	B(i)	44.9	0	-	M+Na	C18H33N11O4	3.7047833333333	-	pos	490.26330337666	0.088295093974957	44.1	-
pos_5676	Indole-3-Carboxaldehyde	metab_5675	B(i)	88.6	0	HMDB0029737	M+H	C9H7NO	3.7154333333333	C08493	pos	146.05967578462	0.007205371461141	56.4	487-89-8
pos_5678	Trans-3-Indoleacrylic Acid	metab_5677	B(i)	86.7	0	HMDB0000734	M+H	C11H9NO2	3.7154333333333	-	pos	188.07009400874	0.004196043286971	56.4	29953-71-7
pos_5679	L-N-(3-Carboxypropyl)Glutamine	metab_5678	B(ii)	0	58.1	HMDB0029393	M+H-H2O	C9H16N2O5	3.7154333333333	-	pos	215.10213352669	0.032064070238729	49.9	3183-72-0
pos_5680	(2S)-3-Hydroxy-1-[(2S)-Pyrrolidine-2-Carbonyl]Pyrrolidine-2-Carboxylic Acid	metab_5679	B(ii)	0	40.4	HMDB0257791	M+H	C10H16N2O4	3.7154333333333	-	pos	229.11772863811	0.023254519274012	46.5	-
pos_5688	Asp Lys Ile	metab_5687	B(i)	61.9	0	-	M+H	C16H30N4O6	3.7154333333333	-	pos	375.22246599433	0.042715955254143	50.1	-
pos_5692	Ile-Asn-Tyr	metab_5691	B(i)	41	0	-	M+H	C19H28N4O6	3.7154333333333	-	pos	409.20702260189	0.013288814700546	46.4	-
pos_5697	17Beta-Estradiol-3,4-Quinone	metab_5696	B(ii)	0	56	HMDB0060085	2M+H	C18H22O3	3.7154333333333	_;C22358	pos	573.31617389486	0.092464614809469	48.9	144082-88-2;_
pos_5702	(+/-)-Tryptophan	metab_5701	B(ii)	0	73.6	HMDB0030396	M+H	C11H12N2O2	3.7207166666667	C00806	pos	205.09666076236	0.013839969359153	53.7	1954/12/6
pos_5734	2-{2-Oxo-2-[4-(1H-Pyrrol-1-Yl)Piperidino]Ethoxy}Acetic Acid	metab_5733	B(i)	53.1	0	-	M+H	C13H18N2O4	3.7260166666667	-	pos	267.13329346231	0.007617860935458	49.2	-
pos_5772	Tyr Leu Ala	metab_5771	B(i)	59	0	-	M+H	C18H27N3O5	3.73135	-	pos	366.20202154826	0.16670982743925	48.3	-
pos_5778	Val Phe Arg	metab_5777	B(i)	41.2	0	-	M+H	C20H32N6O4	3.73135	-	pos	421.25467991124	0.010560012701384	45.3	-
pos_5795	Ala Pro Leu	metab_5794	B(i)	93.5	0	-	M+H	C14H25N3O4	3.7366833333333	-	pos	300.19104160946	0.04508516666135	56.7	-
pos_5808	Milbemycin A3	metab_5807	B(ii)	0	76	HMDB0254720	M+K	C31H44O7	3.7366833333333	-	pos	567.27181275913	0.19365067103093	50.2	-
pos_5816	Trp-Gly-Thr	metab_5815	B(i)	54.9	0	-	M+H-H2O	C17H22N4O5	3.7420166666667	-	pos	345.15527511291	0.088965499155184	48.8	-
pos_5838	Gln Gln Phe	metab_5837	B(i)	96.3	0	-	M+H	C19H27N5O6	3.74735	-	pos	422.202197	0.045414660975031	57.3	-

pos_5859	Pe(22:6/0:0)	metab_5858	B(i)	47.9	0	-	M+H	C27H44NO7P	3.7526166666667	-	pos	526.29679604545	0.042868975138561	45.3	-
pos_5864	Asn Gly Phe	metab_5863	B(i)	89.5	0	-	M+H	C15H20N4O5	3.7579	-	pos	337.14989192969	0.078215923417281	53.9	-
pos_5881	Elamipretide	metab_5880	B(ii)	0	56.8	HMDB0251733	M+K	C32H49N9O5	3.7632	-	pos	678.35480004049	0.087211812636949	45	-
pos_5884	Isoindoline	metab_5883	B(ii)	0	54.7	HMDB0253648	M+	C8H9N	3.7685333333333	-	pos	119.07263643621	0.004038173390380	47.9	-
pos_5898	Glu Pro Leu	metab_5897	B(i)	89	0	-	M+H	C16H27N3O6	3.7738166666667	-	pos	358.19642729315	0.018915125651325	56	-
pos_5911	Hexaethylene Glycol	metab_5910	B(i)	53	0	HMDB0061822	M+H	C12H26O7	3.7843833333333	-	pos	283.17433555158	0.017661910768547	48.6	2615-15-8
pos_5916	Gln-Thr-Phe	metab_5915	B(i)	67.1	0	-	M+H	C18H26N4O6	3.7843833333333	-	pos	395.19125129177	0.011196598594128	51.7	-
pos_5921	(24E)-3Alpha-Acetoxy-15Alpha-Hydroxy-23-Oxo-7,9(11),24-Lanostatrien-26-Oic Acid	metab_5920	B(ii)	0	42.1	HMDB0035386	M+Na-H2O	C32H46O6	3.7843833333333	-	pos	531.31225484211	0.083445496792451	42.4	117383-37-6
pos_5925	Val Val	metab_5924	B(i)	68.6	0	-	M+H	C10H20N2O3	3.7897	-	pos	217.15418455964	0.014163938215788	52.5	-
pos_5934	Gln Val Tyr Asp	metab_5933	B(i)	42.2	0	-	M+H	C23H33N5O9	3.7897	-	pos	524.23397913382	0.058684864933213	45.6	-
pos_5957	Norfenefrine	metab_5956	B(i)	92.6	0	HMDB0255737	M+H-H2O	C8H11NO2	3.8003	-	pos	136.07534095851	0.045052096951623	57.1	-
pos_5958	5,6-Dimethoxyindan-1-One	metab_5957	B(i)	61.2	0	-	M+H	C11H12O3	3.8003	-	pos	193.08548556592	0.036285232138595	51.1	-
pos_5961	L-Aspartyl-L-Phenylalanine	metab_5960	B(i)	97.9	0	HMDB0000706	M+H	C13H16N2O5	3.8003	-	pos	281.11246203431	0.020386315726417	58.2	13433-09-5
pos_5978	Glu-Phe	metab_5977	B(i)	93.5	0	HMDB0029156	M+H	C14H18N2O5	3.8056	-	pos	295.12816185304	0.007980165458455	57.2	20556-22-3
pos_5980	Gln-Ser-Leu	metab_5979	B(i)	67.5	0	-	M+H	C14H26N4O6	3.8056	-	pos	347.19176153889	0.010930500093798	52.2	-
pos_5999	Pro Val Val	metab_5998	B(i)	82.5	0	-	M+H	C15H27N3O4	3.8109166666667	-	pos	314.20662640426	0.10438068691158	53.7	-
pos_6041	Ala-Ala-Lys	metab_6040	B(i)	59	0	-	M+H-H2O	C12H24N4O4	3.8215333333333	-	pos	271.17583140549	0.075994909108004	50	-
pos_6100	Leu-Leu-Ser	metab_6099	B(i)	64.4	0	-	M+H	C15H29N3O5	3.8321666666667	-	pos	332.21716657236	0.017292093767204	50.9	-
pos_6107	Ps(4:0/16:2)	metab_6106	B(i)	39.6	0	-	M+CH3OH+Na	C26H46NO10P	3.8321666666667	-	pos	618.2989011	0.070316298409003	44.4	-
pos_6127	Arg-Val-Phe	metab_6126	B(i)	78.9	0	-	M+H	C20H32N6O4	3.83745	-	pos	421.25459029014	0.011397000723838	54.6	-
pos_6132	28-Hydroxywithanolide E	metab_6131	B(ii)	0	77.3	HMDB0031355	M+NH4	C28H38O8	3.83745	-	pos	520.2868717	0.016201117378047	52.5	188346-67-0
pos_6155	1,2,10-Trihydroxydihydro-Trans-Linalyl Oxide 7-O-Beta-D-Glucopyranoside	metab_6154	B(ii)	0	47.9	HMDB0033237	M+ACN+Na	C16H30O10	3.84275	-	pos	446.19761056758	0.061176413948552	46.3	219814-37-6
pos_6172	Valyl-Arginine	metab_6171	B(ii)	0	62.8	HMDB0304792	M+H-H2O	C11H23N5O3	3.8480166666667	-	pos	256.17619647848	0.056102348200329	50	-
pos_6238	Val Leu	metab_6237	B(i)	66.6	0	-	M+H	C11H22N2O3	3.86395	-	pos	231.16974298855	0.009546252157525	52.3	-
pos_6243	Val Thr Val	metab_6242	B(i)	56.6	0	-	M+H	C14H27N3O5	3.86395	-	pos	318.20158704772	0.077948157652237	47.9	-
pos_6259	Hetastarch	metab_6258	B(ii)	0	52	HMDB0253113	M+Na	C29H52O21	3.86395	-	pos	759.29223534992	0.033426985168015	47.6	-
pos_6314	Arg Val Phe Asp	metab_6313	B(i)	35.8	0	-	M+H	C24H37N7O7	3.8798833333333	-	pos	536.28147283903	0.044439857640693	44.9	-
pos_6331	Pro-Tyr-His	metab_6330	B(i)	73.2	0	-	M+H	C20H25N5O5	3.8851666666667	-	pos	416.19198163277	0.007424180252935	53.4	-
pos_6361	Ala Phe Lys	metab_6360	B(i)	49.2	0	-	M+H	C18H28N4O4	3.89585	-	pos	365.2172029	0.04210189547193	47.4	-
pos_6389	Pro Ser Arg	metab_6388	B(i)	47	0	-	M+H	C14H26N6O5	3.9011333333333	-	pos	359.20097679302	0.025740186916106	41.2	-
pos_6392	Phe-Gln-Pro	metab_6391	B(i)	87.3	0	-	M+H	C19H26N4O5	3.9011333333333	-	pos	391.19665586581	0.004418101863296	55.8	-
pos_6479	Asn Glu Phe	metab_6478	B(i)	77.4	0	-	M+H	C18H24N4O7	3.9277166666667	-	pos	409.17077528364	0.055787187762721	53	-
pos_6484	Ile Thr Tyr Asp	metab_6483	B(i)	57.1	0	-	M+H	C23H34N4O9	3.9277166666667	-	pos	511.23884294283	0.026275308698576	50.3	-
pos_6532	Uracil Analogue	metab_6531	B(ii)	0	46.4	HMDB0259710	M+H-H2O	C12H16N2O4	3.9438	-	pos	235.10715231302	0.031887749570899	46.7	-
pos_6544	Arg-Ser-Asn	metab_6543	B(i)	45.1	0	-	M+CH3OH+H	C13H25N7O6	3.9438	-	pos	408.22313276918	0.037624680968974	43.9	-
pos_6596	3-Phenyl-1-(4-Propan-2-Ylanilino)Pyrrolidine-2,5-Dione	metab_6595	B(i)	39.4	0	-	M+H-2H2O	C19H20N2O2	3.9545	-	pos	273.13893804304	0.028097397929551	44.1	-
pos_6631	Pc(20:5/0:0)	metab_6630	B(i)	66.9	0	LMGP01050050	M+H	C28H48NO7P	3.95985	-	pos	542.32858545693	0.079352668249774	48.9	0
pos_6643	Ile Leu Thr	metab_6642	B(i)	51.7	0	-	M+H	C16H31N3O5	3.9652	-	pos	346.2328594	0.055633181538613	48	-
pos_6645	Arg-Leu-Leu	metab_6644	B(i)	85.1	0	-	M+H	C18H36N6O4	3.9652	-	pos	401.28603718884	0.024386770852001	54.7	-
pos_6646	Met-Trp-Ile	metab_6645	B(i)	35.9	0	-	M+H	C22H32N4O4S	3.9652	-	pos	449.2231848	0.017264281008313	44.1	-
pos_6666	Tobramycin	metab_6665	B(ii)	0	78.9	HMDB0014822	M+H-2H2O	C18H37N5O9	3.9705333333333	C00397	pos	432.24413379653	0.032925625235778	54.1	32986-56-4
pos_6669	20-Oxo-Leukotriene E4	metab_6668	B(ii)	0	52.8	HMDB0012642	M+H	C23H35NO6S	3.9705333333333	-	pos	454.22643150518	0.068968821234307	46.9	_;
pos_6740	Phe Ala Thr Asp Asp	metab_6739	B(i)	37.1	0	-	M+H	C24H33N5O11	3.9865166666667	-	pos	568.22109895185	0.0461156619234	44.1	-
pos_6765	Pro-Phe-Thr	metab_6764	B(i)	58.2	0	-	M+H	C18H25N3O5	3.9971166666667	-	pos	364.18491825514	0.0252810205218	49.6	-

pos_6826	Diacetylsongorine	metab_6825	B(i)	38.3	0	-	M+Na	C22H31NO3	4.01315	-	pos	380.21730988881	0.01899853088094	41.4	-
pos_6845	5-Nitro-2-(3-Phenylpropylamino)Benzoic Acid	metab_6844	B(i)	70.7	0	HMDB0255766	M+H	C16H16N2O4	4.0184666666667	C13705	pos	301.11855348095	0.025311410329121	52.4	107254-86-4;_
pos_6852	Ala-Thr-Phe	metab_6851	B(i)	61.4	0	-	M+CH3OH+Na	C16H23N3O5	4.0184666666667	-	pos	392.18064515441	0.026966978841878	50.7	-
pos_6855	Arg Pro Tyr	metab_6854	B(i)	47.1	0	-	M+H	C20H30N6O5	4.0184666666667	-	pos	435.23387525105	0.002578885360020	48.2	-
pos_6899	Physagulin B	metab_6898	B(ii)	0	65.5	HMDB0041048	M+ACN+H	C30H39ClO7	4.02905	-	pos	588.27586742845	0.022346857485817	46.6	146713-97-5;
pos_6913	Pro Met Tyr	metab_6912	B(i)	83.6	0	-	M+H	C19H27N3O5S	4.0344	-	pos	410.17354228888	0.057301574911092	53.3	-
pos_6920	Glycyrrhizin	metab_6919	B(ii)	0	77.1	HMDB0029843	M+H	C42H62O16	4.0344	C02284;_	pos	823.41748244612	0.083147244994712	50.3	1405-86-3;103000-77-7;1405-86-3;
pos_6923	4-[2-(1,3-Benzodioxol-5-Yl)Acetyl]-3-Methylpiperazin-2-One	metab_6922	B(i)	41.8	0	-	M+H	C14H16N2O4	4.0397333333333	-	pos	277.11758560953	0.007760982178093	46.9	-
pos_6928	Met Pro Met	metab_6927	B(i)	93	0	-	M+H	C15H27N3O4S	4.0397333333333	-	pos	378.15089455824	0.013688950319469	56	-
pos_6937	7-Glucosyl-11-Methylodeoside	metab_6936	B(i)	95	0	-	M+Na	C23H34O16	4.0397333333333	-	pos	589.17225488412	0.090507815839238	56.3	-
pos_6945	Leu Gly Val	metab_6944	B(i)	52.5	0	-	M+H	C13H25N3O4	4.0450833333333	-	pos	288.19218536386	0.054540566594951	49.4	-
pos_6951	2'-O-Methylcajanone	metab_6950	B(ii)	0	61.7	HMDB0033806	M+CH3OH+Na	C26H28O6	4.0450833333333	-	pos	491.20125670914	0.043931914454306	42.9	71765-79-2;
pos_6963	Lappaol D	metab_6962	B(ii)	0	45.6	HMDB0302070	M+NH4	C30H32O9	4.0504	C17684;C17812	pos	554.23335572822	0.046952728937711	43.9	-;_
pos_6964	L-Beta-Homothreonine	metab_6963	B(i)	42	0	-	M+H	C5H11NO3	4.0557	-	pos	134.08087130796	0.034056154631276	46.7	-
pos_6977	7-Deoxyechinosporin	metab_6976	B(i)	81.7	0	-	M+H-H2O	C10H9NO4	4.061	-	pos	190.04941811838	0.018019974610241	54.8	-
pos_7025	2-Piperidinone	metab_7024	B(ii)	0	50.9	HMDB0011749	2M+H	C5H9NO	4.0768833333333	-	pos	199.14362212592	0.093992416983902	49.2	675-20-7
pos_7034	Pro Thr Ile	metab_7033	B(i)	94.6	0	-	M+H	C15H27N3O5	4.0768833333333	-	pos	330.20148855388	0.017702801474482	57	-
pos_7085	Ser Trp	metab_7084	B(i)	69.2	0	-	M+H	C14H17N3O4	4.0929166666667	-	pos	292.12853822773	0.019971870080968	52.1	-
pos_7095	His-Asp-Arg	metab_7094	B(i)	36.3	0	-	M+H	C16H26N8O6	4.0929166666667	-	pos	427.20645907518	0.020866387105438	45.7	-
pos_7155	Caryoptosidic Acid	metab_7154	B(ii)	0	40.7	HMDB0034249	M+H-2H2O	C16H24O11	4.1088166666667	-	pos	357.11784859228	0.067698506034441	44.5	220271-92-1
pos_7169	(E,E)-4,4"-Bi(N-4-Hydroxycinnamoylserotonin)	metab_7168	B(ii)	0	52.8	HMDB0041431	M+CH3OH+H	C38H34N4O6	4.1088166666667	-	pos	675.28111931517	0.031550578565306	47.6	175702-01-9
pos_7183	Asp Glu Phe	metab_7182	B(i)	87.7	0	-	M+H	C18H23N3O8	4.1141666666667	-	pos	410.15522626771	0.024238286499212	55.6	-
pos_7221	(2S)-2-Amino-3-[4-[(2S)-2-Amino-3-Phenylpropanoyl]Oxyphenyl]Propanoic Acid	metab_7220	B(ii)	0	57.1	HMDB0257556	M+H	C18H20N2O4	4.1248166666667	-	pos	329.14869843397	0.0070915448811415	49.7	-
pos_7227	Lys-Ile-Ile	metab_7226	B(i)	79.2	0	-	M+H	C18H36N4O4	4.1248166666667	-	pos	373.28002044943	0.029159480557537	53	-
pos_7231	Gentamicin A Sulfate	metab_7230	B(ii)	0	47.4	HMDB0252686	M+Na-H2O	C18H36N4O10	4.1248166666667	-	pos	473.22395906814	0.054509620968126	45.8	-
pos_7234	Tyr Val Gln Glu	metab_7233	B(i)	37	0	-	M+H	C24H35N5O9	4.1248166666667	-	pos	538.24900247402	0.049656616037979	44.6	-
pos_7246	Ala His Trp	metab_7245	B(i)	55.7	0	-	M+H	C20H24N6O4	4.1301	-	pos	413.19208046813	0.047235684416576	49.7	-
pos_7289	(1Xi,3S)-1,2,3,4-Tetrahydro-1-Methyl-Beta-Carboline-1,3-Dicarboxylic Acid	metab_7288	B(ii)	0	41.5	HMDB0035115	M+ACN+H	C14H14N2O4	4.1460833333333	-	pos	316.12850331717	0.015610045361105	46.2	-
pos_7291	Gln Trp	metab_7290	B(i)	87.8	0	-	M+H	C16H20N4O4	4.1460833333333	-	pos	333.15457540279	0.009693656847454	55.4	-
pos_7329	Trp Gly	metab_7328	B(i)	36.2	0	-	M+H	C13H15N3O3	4.15675	-	pos	262.11800763358	0.025450838742628	45.4	-
pos_7334	Octaethylene Glycol	metab_7333	B(i)	78.2	0	HMDB0094680	M+NH4	C16H34O9	4.15675	-	pos	388.25319104161	0.016806363417458	54.3	-
pos_7337	Phe Asp Ala Glu	metab_7336	B(i)	40.7	0	-	M+H	C21H28N4O9	4.15675	-	pos	481.18949176051	0.019634395377848	45.1	-
pos_7347	2-Hydroxy-3-(4-Hydroxyphenyl)Propenoic Acid	metab_7346	B(ii)	0	49	HMDB0006915	M+H-H2O	C9H8O4	4.1620833333333	C05350	pos	163.03857448746	0.28809709679064	47.9	-
pos_7354	Marticin	metab_7353	B(i)	57	0	-	M+H	C18H16O9	4.1620833333333	-	pos	377.08345339123	0.090593399526873	48.1	-
pos_7375	Arg-Ile-Ile	metab_7374	B(i)	95.3	0	-	M+H	C18H36N6O4	4.1674166666667	-	pos	401.28619370682	0.013519441063845	57.1	-
pos_7391	Pro Leu Arg	metab_7390	B(i)	88.4	0	-	M+H	C17H32N6O4	4.1727666666667	-	pos	385.25496222722	0.024535576242741	56.1	-
pos_7404	N(6)-(1-Carboxyethyl)-L-Lysine	metab_7403	B(ii)	0	47.7	HMDB0243495	M+H-H2O	C9H18N2O4	4.1780666666667	-	pos	201.12289716276	0.008016514572225	47.7	-
pos_7419	1-Carboxyethyltyrosine	metab_7418	B(ii)	0	53	HMDB0242159	M+H	C12H15NO5	4.1833666666667	-	pos	254.10172908281	0.016772580188259	48.6	-
pos_7422	Ala-Trp	metab_7421	B(i)	86.3	0	HMDB0013209	M+H	C14H17N3O3	4.1833666666667	-	pos	276.13374888809	0.015743978254381	55.5	16305-75-2
pos_7429	Dibekacin	metab_7428	B(ii)	0	68.8	HMDB0251157	M+H-2H2O	C18H37N5O8	4.1833666666667	-	pos	416.24943616906	0.011336901542081	52.3	-
pos_7438	Dl-O-Tyrosine	metab_7437	B(i)	85.8	0	HMDB0006050	M+H	C9H11NO3	4.1887	-	pos	182.08074041921	0.007960983886699	55.9	2370-61-8
pos_7451	Perindoprilat Glucuronide	metab_7450	B(ii)	0	71.9	HMDB0060863	M+	C23H36N2O11	4.1887	-	pos	516.22861883516	0.019446449891645	50.8	-

pos_7455	Dopamine	metab_7454	B(i)	75.9	0	HMDB0000073;PW_C000052	M+H-H2O	C8H11NO2	4.1940166666667	C03758	pos	136.07536360652	0.022624702941947	53.3	51-61-6;62-31-7
pos_7468	Geldanamycin	metab_7467	B(ii)	0	83.1	HMDB0252665	M+NH4	C29H40N2O9	4.1940166666667	_;C11222	pos	578.30170350904	0.094274673470656	51.2	30562-34-6;_
pos_7504	(1R,2R,4S)-P-Menthane-1,2,8-Triol 8-Glucoside	metab_7503	B(ii)	0	56.8	HMDB0039894	M+Na	C16H30O8	4.2100166666667	-	pos	373.18233786918	0.043333562825609	49.3	378254-09-2
pos_7521	Tyr-Leu	metab_7520	B(i)	90.4	0	HMDB0029109	M+H	C15H22N2O4	4.2153666666667	-	pos	295.16472263795	0.019215597028306	57.1	-
pos_7535	Cyasterone	metab_7534	B(ii)	0	50.1	HMDB0250618	M+K	C29H44O8	4.2153666666667	_;C08816	pos	559.27138905107	0.097237059342318	44.6	17086-76-9;_;
pos_7579	Gln-Leu-Tyr	metab_7578	B(i)	88.9	0	-	M+H	C20H30N4O6	4.2259833333333	-	pos	423.22288170878	0.041911299734466	55.4	-
pos_7600	Leu Gln Met	metab_7599	B(i)	88.4	0	-	M+H	C16H30N4O5S	4.2313333333333	-	pos	391.19984182149	0.011121830111962	55.3	-
pos_7632	4-N-Ethyl-1-N-[4-(Phenylcarbamoylamino)Phenyl]Piperidine-1,4-Dicarboxamide	metab_7631	B(i)	44.2	0	-	M+H	C22H27N5O3	4.2366666666667	-	pos	410.22161793323	0.031880440746453	42.4	-
pos_7671	M-Xylylenediamine	metab_7670	B(i)	43.4	0	-	M+CH3OH+Na	C8H12N2	4.2472833333333	-	pos	191.11553913098	0.01378501855439	46.7	-
pos_7682	Nalfurafine	metab_7681	B(ii)	0	45.2	HMDB0255431	M+H	C28H32N2O5	4.2472833333333	-	pos	477.24198783706	0.036357993080239	44.1	-
pos_7699	Pro Ala Phe	metab_7698	B(i)	88	0	-	M+H	C17H23N3O4	4.2526166666667	-	pos	334.17527300659	0.011850901134252	56	-
pos_7740	N-Desmethyl Zolmitriptan	metab_7739	B(ii)	0	42.8	HMDB0255120	M+H	C15H19N3O2	4.2688833333333	-	pos	274.15748611908	0.086633839609628	43.3	-
pos_7756	8-Aminooctanoic Acid	metab_7755	B(ii)	0	54.7	HMDB0247418	M+H	C8H17NO2	4.2741833333333	-	pos	160.13280632474	0.007829281662892	50	-
pos_7760	N-(2-Cyclohexyl-2-Hydroxyethyl)-2-(2,5-Dioxopyrrolidin-1-Yl)Acetamide	metab_7759	B(i)	53.8	0	-	M+H	C14H22N2O4	4.2741833333333	-	pos	283.16218084398	0.062764741788048	46.5	-
pos_7766	Phe-Thr-Ala	metab_7765	B(i)	70.9	0	-	M+H	C16H23N3O5	4.2741833333333	-	pos	338.17018863969	0.10027075137198	51.7	-
pos_7772	Asp-Glu-Arg	metab_7771	B(i)	51.2	0	-	M+CH3OH+H	C15H26N6O8	4.2741833333333	-	pos	451.21749166702	0.029681296801817	47.5	-
pos_7801	Hv-Ncc-1	metab_7800	B(ii)	0	77.5	HMDB0039005	M+ACN+H	C35H42N4O10	4.2795166666667	-	pos	720.32943717341	0.048750442820385	50.4	135972-64-4
pos_7818	Phe Met	metab_7817	B(i)	62.9	0	-	M+H	C14H20N2O3S	4.2902	-	pos	297.12607820078	0.058491772265922	49.8	-
pos_7836	Val Tyr Val	metab_7835	B(i)	91.3	0	-	M+H	C19H29N3O5	4.2954833333333	-	pos	380.21730536393	0.029778899398471	56.4	-
pos_7881	Ethyl 3-Hydroxybutyrate	metab_7880	B(i)	85.9	0	HMDB0040409	M+H	C6H12O3	4.3113833333333	-	pos	133.08561484977	0.01840262819528	55.1	35608-64-1
pos_7882	3-Benzyl-6-Methyl-2,5-Piperazinedione	metab_7881	B(i)	75.1	0	-	M+H	C12H14N2O2	4.3113833333333	-	pos	219.11230620044	0.0146505972524	53.8	-
pos_7890	Leu-Pro-Arg	metab_7889	B(i)	48	0	-	M+H	C17H32N6O4	4.3113833333333	-	pos	385.25477233758	0.031896713712565	47.4	-
pos_7977	N-Ethyl-1-(3-Methylphenyl)-5-Oxopyrrolidine-3-Carboxamide	metab_7976	B(i)	43.9	0	-	M+H	C14H18N2O2	4.3380333333333	-	pos	247.14347219468	0.021696267731395	47.4	-
pos_7981	Niazidin	metab_7980	B(ii)	0	49.8	HMDB0031981	M+Na-H2O	C15H18N2O6S	4.3380333333333	-	pos	359.06815437642	0.10567318108102	46.5	198969-42-5
pos_7984	Beta-Casomorphin-5	metab_7983	B(ii)	0	71.1	HMDB0249107	M+H-H2O	C30H37N5O7	4.3380333333333	-	pos	562.27046109459	0.04863497337364	51.2	-
pos_7995	Val Leu Arg	metab_7994	B(i)	83.2	0	-	M+H	C17H34N6O4	4.3433333333333	-	pos	387.27091769021	0.019762115291075	54.3	-
pos_8008	(3R)-3-Hydroxy-N-[(3S)-2-Oxooxolan-3-Yl]Butanamide	metab_8007	B(ii)	0	47.1	HMDB0243607	M+ACN+H	C8H13NO4	4.34865	-	pos	229.117732	0.018469784758645	48.3	-
pos_8019	Kukoamine D	metab_8018	B(ii)	0	45.5	HMDB0060527	M+CH3OH+Na	C28H42N4O6	4.34865	C17618	pos	585.32314704419	0.064383301041763	43.9	-;_
pos_8043	Epsilon-(Carboxyethyl)Lysine	metab_8042	B(ii)	0	65.8	HMDB0251886	M+H-H2O	C9H18N2O4	4.3593166666667	-	pos	201.12291150896	0.003722584332526	51.6	-
pos_8048	Lucidenic Acid K	metab_8047	B(ii)	0	67.8	HMDB0035890	M+NH4	C27H36O7	4.3593166666667	-	pos	490.27671734863	0.063996387082169	49	-
pos_8061	Ile Ser Val	metab_8060	B(i)	82.9	0	-	M+H	C14H27N3O5	4.3646666666667	-	pos	318.20162369119	0.002886002821532	55.2	-
pos_8096	Val Ile Tyr	metab_8095	B(i)	37.1	0	-	M+H	C20H31N3O5	4.3699833333333	-	pos	394.23273804331	0.055312925184884	46.4	-
pos_8105	Betavulgaroside Vii	metab_8104	B(ii)	0	42	HMDB0033427	M+H	C41H62O16	4.3699833333333	-	pos	811.40788421028	0.019391019304265	45.1	178535-51-8
pos_8109	2-(2-Methoxyethoxy)-N-[(7-Oxoazepan-2-Yl)Methyl]Acetamide	metab_8108	B(i)	48.4	0	-	M+H	C12H22N2O4	4.3753	-	pos	259.16474406569	0.062228835971132	46.8	-
pos_8138	Tert-Butyl N-[1-(Aminocarbonyl)-3-Methylbutyl]Carbamate	metab_8137	B(i)	57.9	0	-	M+H	C11H22N2O3	4.38595	-	pos	231.16977104464	0.014934380472836	50.4	-
pos_8144	Ile Phe Thr	metab_8143	B(i)	44.9	0	-	M+H	C19H29N3O5	4.38595	-	pos	380.21725985477	0.013634784220823	47	-
pos_8184	Fruleuile	metab_8183	B(i)	88.1	0	-	M+H	C18H34N2O8	4.39655	-	pos	407.23792605133	0.033429461099486	55.8	-
pos_8257	Urofollitropin	metab_8256	B(ii)	0	83.8	HMDB0259726	M+H-H2O	C42H65N11O12S2	4.4125	-	pos	962.42231362416	0.029767225599039	53.6	-
pos_8283	Trp-Val	metab_8282	B(i)	96.6	0	HMDB0029096	M+H	C16H21N3O3	4.4231	-	pos	304.16512311951	0.026723572343451	58.1	-
pos_8298	Ala-Val-Leu	metab_8297	B(i)	76.7	0	-	M+H	C14H27N3O4	4.42865	-	pos	302.20670769008	0.017917398680532	53.7	-
pos_8324	Leu-Ile-Leu	metab_8323	B(i)	64.1	0	-	M+H-H2O	C18H35N3O4	4.4392333333333	-	pos	340.25850412398	0.018651498766099	51	-
pos_8351	2-Aminooctanedioic Acid	metab_8350	B(i)	90.2	0	-	M+H	C8H15NO4	4.4498833333333	-	pos	190.10690311107	0.21938973267255	55.6	-
pos_8354	Asp Val Leu	metab_8353	B(i)	91.7	0	-	M+H	C15H27N3O6	4.4498833333333	-	pos	346.19630501306	0.014036347789722	56.8	-

pos_8356	Pro His Tyr	metab_8355	B(i)	98.4	0	-	M+H	C20H25N5O5	4.4498833333333	-	pos	416.19171641802	0.002056458591683	58.5	-
pos_8357	Oxytetracycline	metab_8356	B(i)	47.6	0	HMDB0014733;LMPK07000005;PW_C009084	M+H	C22H24N2O9	4.4498833333333	C06624	pos	461.15169112161	0.088600701671304	45.3	79-57-2;
pos_8393	Arg-Gln-Arg	metab_8392	B(i)	80.4	0	-	M+NH4	C17H34N10O5	4.4657666666667	-	pos	476.30521425661	0.046901460807581	54.8	-
pos_8395	Deoxycholylglutamic Acid	metab_8394	B(ii)	0	52.1	HMDB0242437	M+H-H2O	C29H47NO7	4.4657666666667	-	pos	504.33632273329	0.27246838966069	43.8	-
pos_8419	Penigequinolone B	metab_8418	B(i)	36.5	0	-	M+H	C27H33NO6	4.4764	-	pos	468.24171070491	0.085254214114156	41.7	-
pos_8436	Isopentyl Gentiobioside	metab_8435	B(ii)	0	47.9	HMDB0041512	M+H-2H2O	C17H32O11	4.4817	-	pos	377.18118502936	0.013620511089236	47.9	178275-89-3
pos_8445	Harpagoside	metab_8444	B(ii)	0	61.2	HMDB0253053	M+ACN+H	C24H30O11	4.4817	C09783;_	pos	536.2078902	0.095827870979931	46.8	19210-12-9;_;
pos_8499	Curcumenol	metab_8498	B(ii)	0	55.7	HMDB0033960	M+CH3OH+Na	C15H22O2	4.4976	C16942	pos	289.17521648248	0.033506477472998	48.3	-;19431-84-6
pos_8504	Gln-Tyr-Ile	metab_8503	B(i)	59.5	0	-	M+H	C20H30N4O6	4.4976	-	pos	423.22270447151	0.026206091120295	48.8	-
pos_8523	Ile-Lys-Ile	metab_8522	B(i)	83.9	0	-	M+H	C18H36N4O4	4.5028833333333	-	pos	373.28012293471	0.069657005627263	53.9	-
pos_8591	Victoxinine-A-Glycerophosphate	metab_8590	B(i)	44.2	0	-	M+H	C20H36NO6P	4.52445	-	pos	418.23620869069	0.097011602073676	46	-
pos_8592	Ser Arg Tyr	metab_8591	B(i)	52.2	0	-	M+H	C18H28N6O6	4.52445	-	pos	425.21086780034	0.041608078290891	44.6	-
pos_8597	Dactimicin	metab_8596	B(ii)	0	72.9	HMDB0250824	M+CH3OH+Na	C18H36N6O6	4.52445	_;C17979	pos	487.28624802175	0.022819509354866	53.6	73196-97-1;_
pos_8633	Tyr Phe	metab_8632	B(i)	85.9	0	-	M+H	C18H20N2O4	4.5403666666667	-	pos	329.1487833	0.015339948201035	55.6	-
pos_8653	Val Ala Leu	metab_8652	B(i)	83.5	0	-	M+H	C14H27N3O4	4.5509666666667	-	pos	302.20673759153	0.028075995542515	54.5	-
pos_8655	Ile-Asn-Leu	metab_8654	B(i)	61	0	-	M+Na	C16H30N4O5	4.5509666666667	-	pos	381.20950130364	0.10608689424603	48.7	-
pos_8658	Cappariloside B	metab_8657	B(ii)	0	66.7	HMDB0034938	M+H-2H2O	C22H28N2O11	4.5509666666667	-	pos	461.15186015641	0.044253709126962	49.2	229483-42-5
pos_8697	Acetoacetanilide	metab_8696	B(i)	81.1	0	-	M+H	C10H11NO2	4.56695	-	pos	178.08582793695	0.032761996038701	53.4	-
pos_8702	Leu Thr Val	metab_8701	B(i)	84.7	0	-	M+H	C15H29N3O5	4.56695	-	pos	332.21691147499	0.017576727229216	54.3	-
pos_8710	3-O-Demethylfortimicin A	metab_8709	B(ii)	0	56.1	HMDB0245956	M+CH3OH+Na	C16H33N5O6	4.56695	-	pos	446.25980779078	0.018027654063357	49.8	-
pos_8732	Ethyl 2-Amino-3-Phenylpropanoate	metab_8731	B(i)	98.2	0	HMDB0252046	M+H	C11H15NO2	4.5776166666667	-	pos	194.11720268187	0.001766371049930	58.8	-
pos_8789	Arg-Gly-Asp	metab_8788	B(i)	74.4	0	-	M+H	C12H22N6O6	4.5936	-	pos	347.17076808507	0.078348391118502	52.2	-
pos_8790	Methyl (R)-9-Hydroxy-10-Undecene-5,7-Diynoate Glucoside	metab_8789	B(ii)	0	40.6	HMDB0040895	M+H	C18H24O8	4.5936	-	pos	369.15224993154	0.035123621586654	45.5	152141-44-1
pos_8808	Rcs-4 3-Methoxy Isomer	metab_8807	B(i)	46.4	0	-	M+H	C21H23NO2	4.5989	-	pos	322.17655120827	0.052044215374961	43.5	-
pos_8813	4-Hydroxyandrostenedione Glucuronide	metab_8812	B(ii)	0	51.7	HMDB0010315	M+H-2H2O	C25H34O9	4.5989	C11455;_	pos	443.20206730294	0.079304282463858	44.7	_;
pos_8842	Limonene Oxide	metab_8841	B(i)	86.6	0	HMDB0035158	M+H	C10H16O	4.6095	C07271	pos	153.12702362068	0.042946506301014	56.1	1195-92-2
pos_8847	Ala Phe Val	metab_8846	B(i)	91.2	0	-	M+H	C17H25N3O4	4.6095	-	pos	336.19036040272	0.010793346675223	54.8	-
pos_8848	Tyr Asn Leu	metab_8847	B(i)	63.8	0	-	M+H	C19H28N4O6	4.6095	-	pos	409.20692964529	0.036752747707266	49.7	-
pos_8849	Ht-2 Toxin	metab_8848	B(ii)	0	47.3	HMDB0253219	M+Na-H2O	C22H32O8	4.6095	C19952;_	pos	429.18608896542	0.027124127975045	43.4	26934-87-2;_
pos_8862	N-Methyl-A-Aminoisobutyric Acid	metab_8861	B(i)	78.8	0	HMDB0002141	M+H	C5H11NO2	4.6148666666667	-	pos	118.08597701117	0.096838850888087	54	2566-34-9
pos_8872	11-Alpha-O-Beta-D-Glucopyranosyl-16Alpha-O-	metab_8871	B(ii)	0	45.2	HMDB0039773	M+H-2H2O	C29H44O11	4.6148666666667	-	pos	533.27768659136	0.20064206930669	45.5	-
pos_8906	Pro Arg	metab_8905	B(i)	59.3	0	-	M+H	C11H21N5O3	4.6255166666667	-	pos	272.17100931729	0.018462228742144	48.5	-
pos_8909	Pro Val Ile	metab_8908	B(i)	52.6	0	-	M+H	C16H29N3O4	4.6255166666667	-	pos	328.22118531009	0.021122670465606	46.9	-
pos_8926	Gln-Ala	metab_8925	B(i)	35.5	0	HMDB0028790	M+Na	C8H15N3O4	4.6308166666667	-	pos	240.09736131384	0.020863842783436	44.8	16874-70-7
pos_8928	Clozapine N-Oxide	metab_8927	B(i)	35.4	0	HMDB0060900	M+H	C18H19ClN4O	4.6308166666667	-	pos	343.12899393858	0.011615930631563	39	-
pos_8930	Val Asn Phe	metab_8929	B(i)	62.3	0	-	M+H	C18H26N4O5	4.6308166666667	-	pos	379.19658480109	0.0401059368342	51.1	-
pos_8966	Leu Val Val	metab_8965	B(i)	88.8	0	-	M+H	C16H31N3O4	4.6414833333333	-	pos	330.23800786464	0.00786830249007	55.7	-
pos_8975	16,17-Dihydro-16Alpha,17-Dihydroxygibberellin A4 17-	metab_8974	B(ii)	0	85.1	HMDB0040647	M+ACN+H	C25H36O12	4.6414833333333	-	pos	570.25470814512	0.054816927114399	56	159858-83-0
pos_9028	Phe Trp Asn	metab_9027	B(i)	93.1	0	-	M+H	C24H27N5O5	4.6575	-	pos	466.20757964336	0.021806759590699	56.6	-
pos_9071	Val-Glu-Ile	metab_9070	B(i)	70.8	0	-	M+H	C16H29N3O6	4.6734166666667	-	pos	360.2121563	0.023213531561265	52.2	-
pos_9119	Phe-Leu	metab_9118	B(i)	91.9	0	HMDB0253025	M+H	C15H22N2O3	4.6894	-	pos	279.1696619	0.010248771835498	57	-
pos_9157	Uzarigenin 3-[Xylosyl-(1->2)-Rhamnoside]	metab_9156	B(ii)	0	50.8	HMDB0036296	M+NH4	C34H52O12	4.6946833333333	-	pos	670.37551080089	0.041186566307486	46.9	255861-29-1
pos_9168	Glu-Arg-His	metab_9167	B(i)	36.9	0	-	M+H	C17H28N8O6	4.6999833333333	-	pos	441.22206199672	0.018713106281505	45.7	-
pos_9224	Versiconol	metab_9223	B(i)	42.6	0	-	M+H	C18H16O8	4.7160166666667	C20508	pos	361.08843857522	0.069553858183792	44.7	22268-13-9
pos_9255	Myricanene A 5-[Arabinosyl-(1->6)-Glucoside]	metab_9254	B(ii)	0	47.9	HMDB0039351	M+H	C32H42O13	4.7266666666667	-	pos	635.26558988348	0.028737502440248	43.9	-
pos_9260	Ala-Ala-Ala	metab_9259	B(ii)	0	70.3	HMDB0248092	M+H-H2O	C9H17N3O4	4.7320333333333	-	pos	214.11809603864	0.053219017695812	51.6	-

pos_9277	N-Phenylanthranilic Acid	metab_9276	B(ii)	0	57	HMDB0255225	M+H	C13H11NO2	4.7373666666667	C13697	pos	214.08573403031	0.018440252918856	49.2	-
pos_9295	Melongoside O	metab_9294	B(ii)	0	83.6	HMDB0030040	M+H-2H2O	C45H74O19	4.7373666666667	-	pos	883.4717985	0.10009725801547	51.2	98464-54-1
pos_9319	Plactin D	metab_9318	B(ii)	0	84.4	HMDB0256630	M+CH3OH+Na	C32H52N8O5	4.7427166666667	-	pos	683.41798174981	0.042612139373961	53.1	-
pos_9327	7-Deacetoxy-7-Oxodeoxygedunin	metab_9326	B(i)	39.1	0	-	M+H	C26H30O5	4.74805	-	pos	423.21523288826	0.05560008593201	43.4	-
pos_9340	3-Indolebutyric Acid	metab_9339	B(ii)	0	63.8	HMDB0002096	M+NH4	C12H13NO2	4.7534	C11284	pos	221.1279054	0.018075781672344	49.8	133-32-4
pos_9350	Epsilon-(Carboxymethyl)Lysine	metab_9349	B(ii)	0	46.3	HMDB0251887	M+H-H2O	C8H16N2O4	4.759	-	pos	187.1072482	0.003202808735619	47.7	-
pos_9355	Tyr Trp	metab_9354	B(i)	86.7	0	-	M+H	C20H21N3O4	4.759	-	pos	368.16004199815	0.0194587614055	55.4	-
pos_9389	Hoechst 33342	metab_9388	B(i)	70.7	0	HMDB0253196	M+H	C27H28N6O	4.7696	C11189	pos	453.24494585199	0.013662861036748	47.3	23491-52-3;_
pos_9439	Val Ala Phe	metab_9438	B(i)	61.4	0	-	M+H	C17H25N3O4	4.7855833333333	-	pos	336.19090583313	0.013113844174451	50.2	-
pos_9473	Desglucocheirotoxin	metab_9472	B(ii)	0	51.3	HMDB0034362	M+H	C29H42O10	4.7909	-	pos	551.28703980047	0.049065906095874	48.1	5822-57-1
pos_9480	Gingerglycolipid C	metab_9479	B(ii)	0	43.3	HMDB0041095	M+CH3OH+Na	C33H60O14	4.7909	-	pos	735.4122704	0.083212218486259	45.2	35949-86-1;
pos_9488	Citreoviridin	metab_9487	B(ii)	0	65.1	HMDB0250305	M+CH3OH+Na	C23H30O6	4.79625	C16766;_	pos	457.21802878926	0.074045141340409	50.8	25425-12-1;_
pos_9511	Jubanine C	metab_9510	B(ii)	0	48.5	HMDB0037300	M+H-2H2O	C39H47N5O5	4.8015833333333	-	pos	630.34955491769	0.11513090137604	42.4	159226-00-3
pos_9533	Fevicordin B 2-Gentiobioside	metab_9532	B(ii)	0	44.6	HMDB0035296	M+H-2H2O	C43H64O18	4.8069333333333	-	pos	833.39160944895	0.081150014039441	42.3	151589-22-9
pos_9591	Vicine	metab_9590	B(ii)	0	49.8	HMDB0034073	M+H-H2O	C10H16N4O7	4.8282833333333	C08437	pos	287.09713520526	0.038588532082194	47.1	152-93-2
pos_9612	Pyroglutamylisoleucine	metab_9611	B(i)	77.9	0	-	M+H	C11H18N2O4	4.8335833333333	-	pos	243.13335659888	0.008757599350398	54.5	-
pos_9676	Leu-Phe	metab_9675	B(i)	84.8	0	HMDB0302841	M+H	C15H22N2O3	4.8548	-	pos	279.16961944381	0.023448724588894	55.7	-
pos_9745	Arg Gln Ser Lys	metab_9744	B(i)	66.9	0	-	M+H	C20H39N9O7	4.8707833333333	-	pos	518.30658353723	0.048889159849254	50.5	-
pos_9746	Isoschaftoside	metab_9745	B(i)	73.4	0	LMPK12110243	M+H	C26H28O14	4.8707833333333	-	pos	565.1537325	0.031755032259757	50.7	0
pos_9763	Prunin 6''-O-Gallate	metab_9762	B(ii)	0	65.2	HMDB0037582	M+H	C28H26O14	4.8760833333333	-	pos	587.1356714	0.054744375181838	47.3	54835-98-2;
pos_9799	Proscillaridin	metab_9798	B(ii)	0	77.8	HMDB0256851	M+NH4	C30H42O8	4.88675	C12816;_	pos	548.31754623284	0.069052498644718	51.6	466-06-8;_
pos_9821	3H-Adrenaline	metab_9820	B(ii)	0	59.7	HMDB0246263	M+CH3OH+H	C9H15NO3	4.8974333333333	-	pos	218.13874799375	0.040220447155716	50	-
pos_9825	Val Ser Trp	metab_9824	B(i)	78.3	0	-	M+H	C19H26N4O5	4.8974333333333	-	pos	391.19751080961	0.02860579664726	53.1	-
pos_9835	Corchoroside A	metab_9834	B(ii)	0	42.7	HMDB0033846	M+ACN+Na	C29H42O9	4.8974333333333	-	pos	598.29600750046	0.022570807203311	44.7	508-76-9
pos_9883	Glycinoeclepin C	metab_9882	B(ii)	0	42.4	HMDB0037036	M+H	C29H38O8	4.9134166666667	-	pos	515.25996470204	0.093220987647396	44.7	-
pos_9897	Ile-Ile-Tyr	metab_9896	B(i)	89.1	0	-	M+H	C21H33N3O5	4.9187	-	pos	408.24824554129	0.050427344580486	55.7	-
pos_9947	Thr Phe Asp Glu Ile	metab_9946	B(i)	36.5	0	-	M+H	C28H41N5O11	4.9296333333333	-	pos	624.2857615	0.11831059266962	45	-
pos_9953	Ethyl 3-Phenylglycidate	metab_9952	B(i)	54.8	0	HMDB0031623	M+NH4	C11H12O3	4.9349333333333	-	pos	210.11195690217	0.070406602696121	48.8	54276-44-7
pos_9984	Phyllanthusol A	metab_9983	B(ii)	0	46.6	HMDB0035942	M+CH3OH+H	C35H49NO19	4.94025	-	pos	820.31884618839	0.15526109907879	45.3	294864-88-3
pos_9994	Pro Trp His	metab_9993	B(i)	50.9	0	-	M+H	C22H26N6O4	4.9455333333333	-	pos	439.20774290555	0.029018154195218	46.3	-
pos_10005	Cyclo-Ile-Pro-Diketopiperazine	metab_10004	B(i)	88.6	0	-	M+H	C11H18N2O2	4.95085	-	pos	211.14359175968	0.058394323652025	55.9	-
pos_10019	Fagomine	metab_10018	B(ii)	0	43.1	HMDB0033453	M+H	C6H13NO3	4.9561833333333	C10144	pos	148.09647904685	0.15056688751998	46.8	53185-12-9
pos_10043	Ile Trp	metab_10042	B(i)	91	0	-	M+H	C17H23N3O3	4.9615333333333	-	pos	318.18047880499	0.006808137166628	56.4	-
pos_10060	Ile-Leu-Leu	metab_10059	B(i)	65.5	0	-	M+H-H2O	C18H35N3O4	4.9668666666667	-	pos	340.25854717576	0.05800282211109	49.2	-
pos_10078	Solasonine	metab_10077	B(ii)	0	58.7	HMDB0258364	M+	C45H73NO16	4.9668666666667	C10824;_	pos	883.50083772303	0.12264990498618	46.9	19121-58-5;_
pos_10083	Phe Cys Thr	metab_10082	B(i)	37.5	0	-	M+	C16H23N3O5S	4.9722166666667	-	pos	369.13461341264	0.099275320743209	42.5	-
pos_10085	Cinncassiol D4 2-Glucoside	metab_10084	B(ii)	0	45.7	HMDB0035065	M+Na	C26H42O10	4.9722166666667	-	pos	537.26610750716	0.11127997667332	45.6	-
pos_10128	Tribenzylamine	metab_10127	B(i)	85	0	HMDB0259162	M+H	C21H21N	4.9882333333333	-	pos	288.17733112207	0.06472181413517	52.3	-
pos_10131	Val-Tyr-Ile	metab_10130	B(i)	80.3	0	-	M+H	C20H31N3O5	4.9882333333333	-	pos	394.23279749405	0.019724223626352	53.9	-
pos_10142	Conophylline	metab_10141	B(ii)	0	58.4	HMDB0250438	M+H-H2O	C44H50N4O10	4.9882333333333	-	pos	777.35132420572	0.032551241482218	46.6	-
pos_10216	Hexanoyl-L-Carnitine	metab_10215	B(i)	86.6	0	-	M+H	C13H25NO4	5.02045	-	pos	260.18504913032	0.04338556377801	55.6	-
pos_10239	Palmitoyl Ara-C	metab_10238	B(ii)	0	56.7	HMDB0256087	M+K	C25H43N3O6	5.0257833333333	-	pos	520.27636324281	0.03773043791381	47.2	-
pos_10250	Val-Gly-Leu	metab_10249	B(i)	72	0	-	M+H	C13H25N3O4	5.0311166666667	-	pos	288.19108623769	0.03006940738735	52.8	-
pos_10287	Leu-Ala-Leu	metab_10286	B(i)	84.2	0	-	M+H	C15H29N3O4	5.0418166666667	-	pos	316.22239168949	0.03768698281387	55.1	-
pos_10320	Ethyl 5-Hexenoate	metab_10319	B(ii)	0	43.2	HMDB0032267	M+NH4	C8H14O2	5.0525	-	pos	160.13284445037	0.090933790431763	46.6	54653-25-7
pos_10323	Ser-Ile-Ile	metab_10322	B(i)	82.1	0	-	M+H	C15H29N3O5	5.0525	-	pos	332.2172536	0.034353903781917	54.6	-

pos_10359	Hypaphorine	metab_10358	B(i)	55	0	HMDB0061115	M+CH3OH+Na	C14H18N2O2	5.0630666666667	C09213	pos	301.15153470584	0.020742214961791	49.1	487-58-1
pos_10362	Trp Asn Val	metab_10361	B(i)	93.9	0	-	M+H	C20H27N5O5	5.0630666666667	-	pos	418.20750414828	0.023857226830382	57	-
pos_10371	Oleanolic Acid 3-[Glucosyl-(1->4)-Xyloside]	metab_10370	B(ii)	0	40	HMDB0036357	M+Na-H2O	C41H66O12	5.0630666666667	-	pos	755.42737369725	0.094396070168617	43.6	215171-28-1
pos_10396	Homoharringtonine	metab_10395	B(i)	47	0	HMDB0253207	M+H	C29H39NO9	5.0737166666667	-	pos	546.27577531261	0.036765883767492	41.4	-
pos_10429	Pro Met Phe	metab_10428	B(i)	67.4	0	-	M+H	C19H27N3O4S	5.0896666666667	-	pos	394.17864117145	0.068177350385422	49.8	-
pos_10442	Cyclo(Leu-Pro)	metab_10441	B(i)	90.9	0	HMDB0034276	M+H	C11H18N2O2	5.0951166666667	-	pos	211.14359116921	0.005404613858363	56.8	2873-36-1
pos_10467	Brevetoxin B2	metab_10466	B(ii)	0	76.1	HMDB0032069	M+H-2H2O	C53H79NO17S	5.0951166666667	C20014;_	pos	998.50317370211	0.082653187457411	48.6	-;173691-95-7
pos_10491	11-Oxo-Androsterone Glucuronide	metab_10490	B(ii)	0	42.3	HMDB0010338	M+NH4	C25H36O9	5.1058	-	pos	498.26769848494	0.10897517539644	43.5	_;
pos_10537	Val Val Phe	metab_10536	B(i)	67.3	0	-	M+H	C19H29N3O4	5.1218166666667	-	pos	364.2218104	0.050620324661726	50	-
pos_10575	[(2E,6E)-1-Oxo-8-Hydroxy-2,6-Dimethylocta-2,6-Dien-1-Yl]Beta-D-Glucopyranoside	metab_10574	B(i)	59.9	0	-	M+Na	C16H26O8	5.13245	-	pos	369.1510602	0.11691543116865	49.7	-
pos_10576	Gln Ile Phe	metab_10575	B(i)	87.5	0	-	M+H	C20H30N4O5	5.13245	-	pos	407.22773854206	0.070041843979754	56.2	-
pos_10579	Leukotriene E4	metab_10578	B(ii)	0	52.9	HMDB0002200	M+ACN+H	C23H37NO5S	5.13245	C05952;_	pos	481.27008435413	0.27712477094082	44.8	75715-89-8;_;
pos_10707	Gly-Gly-Ile	metab_10706	B(i)	59.4	0	-	M+H-H2O	C10H19N3O4	5.1752166666667	-	pos	228.1337205	0.014131404981329	49.1	-
pos_10708	Trp Ile	metab_10707	B(i)	90.8	0	-	M+H	C17H23N3O3	5.1752166666667	-	pos	318.18050828418	0.007526778146588	56.6	-
pos_10709	Zolpidem	metab_10708	B(i)	41.1	0	HMDB0005023	M+NH4	C19H21N3O	5.1752166666667	C07219	pos	325.20439368905	0.12114600920089	43	82626-48-0
pos_10722	Ethyl L-Tryptophanate	metab_10721	B(i)	92.5	0	HMDB0252047	M+H	C13H16N2O2	5.18055	-	pos	233.12784409312	0.018136144705908	57.3	-
pos_10746	Solasodine 3-O-Beta-D-Glucopyranoside	metab_10745	B(ii)	0	42.3	HMDB0304488	M+ACN+Na	C33H54NO7+	5.1858833333333	-	pos	640.40114555194	0.014510492513082	45	-
pos_10802	Momorcharaside A	metab_10801	B(ii)	0	48.6	HMDB0032942	M+H	C42H72O15	5.20195	-	pos	817.49056079642	0.030537297390467	47	135126-59-9
pos_10823	Caffeic Acid	metab_10822	B(i)	98.9	0	-;HMDB0001964;HMDB0003501;HMDB0001964	M+H	C9H8O4	5.2125833333333	C01197;C01481	pos	181.04904809505	0.020936587926109	57.9	501-16-6;331-39-5
pos_10828	Skimmin	metab_10827	B(i)	77.8	0	HMDB0258334	M+H	C15H16O8	5.2125833333333	-	pos	325.09093339387	0.016403905567245	54.3	-
pos_10833	6"-Acetylliquiritin	metab_10832	B(ii)	0	43.4	HMDB0041385	M+H	C23H24O10	5.2125833333333	-	pos	461.14288031729	0.066611482560725	45.7	166531-17-5
pos_10834	Calceolarioside A	metab_10833	B(i)	68.9	0	-	M+H	C23H26O11	5.2125833333333	-	pos	479.15343787668	0.038974879748799	52.4	-
pos_10848	Ile Gly Phe	metab_10847	B(i)	92.6	0	-	M+H	C17H25N3O4	5.2179	-	pos	336.19091522603	0.012449222149276	56.7	-
pos_10860	Ala-Thr-Trp-Leu-Pro-Pro-Arg	metab_10859	B(ii)	0	74.9	HMDB0249908	M+ACN+Na	C40H61N11O9	5.2179	-	pos	903.47572766787	0.092279730096918	50.5	-
pos_10879	Leucyl-Serine	metab_10878	B(ii)	0	55.7	HMDB0028938	M+H-H2O	C9H18N2O4	5.22855	-	pos	201.12284731634	0.008517894610903	49.4	-
pos_10896	Leu Ser Phe	metab_10895	B(i)	92	0	-	M+H	C18H27N3O5	5.2339	-	pos	366.20135566177	0.015565527687038	56.5	-
pos_10919	Cyclosquamosin F	metab_10918	B(ii)	0	92.6	HMDB0303371	M+H-2H2O	C36H54N8O11	5.2392333333333	-	pos	739.37284988457	0.10873477211241	54.9	-
pos_10923	Phe-Phe	metab_10922	B(i)	80.3	0	HMDB0013302	M+H	C18H20N2O3	5.2445666666667	-	pos	313.1539019	0.011318823008269	54.6	2577-40-4
pos_10935	Thr-Leu-Leu	metab_10934	B(i)	54.6	0	-	M+H	C16H31N3O5	5.2499166666667	-	pos	346.23278489439	0.012791656849558	49.2	-
pos_10967	[5,6Beta,16-Trihydroxygrayanotox-10(20)-En-3Beta-Yl]Beta-D-Glucopyranoside	metab_10966	B(i)	65.6	0	-	M+Na	C26H42O9	5.2605666666667	-	pos	521.27106927107	0.029103882446231	49.5	-
pos_10976	Thr Val Val	metab_10975	B(i)	35.1	0	-	M+H	C14H27N3O5	5.2658833333333	-	pos	318.2015065	0.014388884080998	44.9	-
pos_10978	Ile-Glu-Leu	metab_10977	B(i)	90.9	0	-	M+H	C17H31N3O6	5.2658833333333	-	pos	374.22776574523	0.028531346323366	55.1	-
pos_11023	Gly-Leu-Ile	metab_11022	B(i)	93.4	0	-	M+H	C14H27N3O4	5.2765833333333	-	pos	302.20666304974	0.016963940697115	57.2	-
pos_11038	Tyr-Pro-Thr	metab_11037	B(i)	45.3	0	-	M+H	C18H25N3O6	5.2819166666667	-	pos	380.17831965048	0.01988035875172	43.2	-
pos_11050	N-Methylthreonine	metab_11049	B(i)	78	0	-	M+H	C5H11NO3	5.2872166666667	-	pos	134.08083135967	0.073231344502701	53.7	-
pos_11085	Atenolol-Desisopropyl	metab_11084	B(i)	47.6	0	-	M+NH4	C11H16N2O3	5.3032166666667	-	pos	242.14932307132	0.022743671106662	47.5	-
pos_11086	Leu-Asp-Ile	metab_11085	B(i)	58.2	0	-	M+H	C16H29N3O6	5.3032166666667	-	pos	360.21215961911	0.038893015562342	49.1	-
pos_11102	Val-Asp-Ile	metab_11101	B(i)	39.6	0	-	M+H-H2O	C15H27N3O6	5.3085666666667	-	pos	328.18580391385	0.18690751932362	44.3	-
pos_11104	Thr-Tyr-Lys	metab_11103	B(i)	63.6	0	-	M+H	C19H30N4O6	5.3085666666667	-	pos	411.22040708151	0.010224221264008	49.4	-
pos_11136	Bisoprolol	metab_11135	B(ii)	0	40.2	HMDB0014750	M+ACN+Na	C18H31NO4	5.3192	C06852	pos	389.23832959057	0.089807388472295	44	66722-44-9
pos_11153	(6R)-N-Ethyl-6-Phenoxy-1,4-Oxazepane-4-Carboxamide	metab_11152	B(i)	76.5	0	-	M+H	C14H20N2O3	5.32455	-	pos	265.15400620274	0.024768766032747	54.1	-
pos_11188	Withanolide B	metab_11187	B(ii)	0	46	HMDB0030020	M+Na-H2O	C28H38O5	5.3352333333333	C00828	pos	459.25153102044	0.1718184438267	40.3	56973-41-2
pos_11246	Furazabol	metab_11245	B(ii)	0	46.4	HMDB0252534	M+CH3OH+Na	C20H30N2O2	5.3515	-	pos	385.2434395	0.023359541134331	45.8	-
pos_11247	Gln Leu Phe	metab_11246	B(i)	82.7	0	-	M+H	C20H30N4O5	5.3515	-	pos	407.22723155624	0.02525245337507	54.5	-

pos_11313	Afalanine	metab_11312	B(i)	98.1	0	HMDB0255056	M+H	C11H13NO3	5.3728166666667	-	pos	208.0962875	0.018018560913421	57.4	-
pos_11315	2-(4-Amino-1-Isopropyl-1H-Pyrazolo[3,4-D]Pyrimidin-3-Yl)-1H-Indol-5-Ol	metab_11314	B(ii)	0	43.4	HMDB0256714	M+H	C16H16N6O	5.3728166666667	-	pos	309.14371718129	0.042160320903552	45.9	-
pos_11316	Phe-Gly-Ile	metab_11315	B(i)	91.5	0	-	M+H	C17H25N3O4	5.3728166666667	-	pos	336.19083448126	0.003258660473875	56.3	-
pos_11395	Leu-Thr-Leu	metab_11394	B(i)	77.8	0	-	M+H	C16H31N3O5	5.3940666666667	-	pos	346.23281192731	0.037850320375788	53.5	-
pos_11396	Asp-Ile-His	metab_11395	B(i)	36.8	0	-	M+NH4	C16H25N5O6	5.3940666666667	-	pos	401.21730676646	0.046258740362899	43.6	-
pos_11419	Ile-Glu-Ile	metab_11418	B(i)	57.2	0	-	M+H	C17H31N3O6	5.4047166666667	-	pos	374.22770350374	0.016346387334029	49.1	-
pos_11421	H-Gly-Arg-Gly-Asp-D-Ser-Pro-Oh	metab_11420	B(ii)	0	41.9	HMDB0253013	M+H-H2O	C22H37N9O10	5.4047166666667	-	pos	570.25874032816	0.078589129542151	43.3	-
pos_11429	Cdp-Dg(Pgj2/A-13:0)	metab_11428	B(ii)	0	65.7	HMDB0292772	M+H-2H2O	C45H73N3O17P2	5.4047166666667	-	pos	954.42683994818	0.032810600515894	50.2	-
pos_11460	Ganoderenic Acid D	metab_11459	B(ii)	0	44.1	HMDB0036059	M+K	C30H40O7	5.4153666666667	-	pos	551.23941470916	0.020885769334945	46.3	100665-43-8
pos_11473	Ile Thr Phe	metab_11472	B(i)	81.1	0	-	M+H	C19H29N3O5	5.4207166666667	-	pos	380.21682251133	0.025166484611431	54.8	-
pos_11496	Urobilin	metab_11495	B(ii)	0	54.2	HMDB0004160	M+ACN+H	C33H42N4O6	5.4261	C05794	pos	632.33940051643	0.027325140705181	45	1856-98-0
pos_11517	3-Hydroxy-P-Cymene	metab_11516	B(i)	77.4	0	HMDB0001878	M+H	C10H14O	5.4367833333333	C09908	pos	151.11134612257	0.1165092018653	53.9	89-83-8
pos_11519	Clorazepate	metab_11518	B(ii)	0	41.3	HMDB0014766	M+NH4	C16H11ClN2O	5.4367833333333	C06921	pos	332.08269598167	0.05255884907441	38.9	23887-31-2
pos_11523	11B-Hydroxypristimerin	metab_11522	B(i)	36.6	0	-	M+H-H2O	C30H40O5	5.4367833333333	-	pos	463.28825739251	0.080839378581964	41.6	-
pos_11530	Isoacteoside	metab_11529	B(i)	77.8	0	HMDB0041025	M+NH4	C29H36O15	5.4367833333333	-	pos	642.23751523693	0.074216619581091	53.5	61303-13-7
pos_11531	Cilengitide	metab_11530	B(ii)	0	65.9	HMDB0250242	M+ACN+Na	C27H40N8O7	5.4367833333333	-	pos	652.31752028871	0.005061428501236	49.7	-
pos_11533	Spirolide B	metab_11532	B(ii)	0	53.1	HMDB0030492	M+K	C42H63NO7	5.4367833333333	-	pos	732.42739471527	0.17618620834578	45.5	170713-72-1
pos_11539	Nordihydroguaiaretic Acid	metab_11538	B(i)	83.2	0	HMDB0014325	M+NH4	C18H22O4	5.4421666666667	C10719	pos	320.18672788971	0.038376436029383	52.3	500-38-9
pos_11553	Periandrin V	metab_11552	B(ii)	0	56.8	HMDB0039591	M+H-H2O	C41H62O14	5.4421666666667	-	pos	761.41696008105	0.047108532936631	47.2	-
pos_11557	2-[(3S)-1-(Oxan-4-Yl)Pyrrolidin-3-Yl]-6-(Trifluoromethyl)-1H-Benzimidazole	metab_11556	B(i)	66.1	0	-	M+H	C17H20F3N3O	5.4474666666667	-	pos	340.16233634452	0.091346430572076	51.3	-
pos_11558	Glu-Phe-Ile	metab_11557	B(i)	66.5	0	-	M+H	C20H29N3O6	5.4474666666667	-	pos	408.21175324594	0.027555721958382	51	-
pos_11574	Pro-Phe	metab_11573	B(i)	92.7	0	HMDB0011179	M+H-H2O	C14H18N2O3	5.4527666666667	-	pos	245.12783185324	0.01747366053914	57.3	13589-02-1
pos_11609	Olitoriusin	metab_11608	B(ii)	0	63.1	HMDB0039542	M+H-2H2O	C41H62O19	5.4581	-	pos	823.38065711182	0.008825640068312	49.5	125708-07-8
pos_11611	Pgp(5-Iso Pgf2Vi/22:5(7Z,10Z,13Z,16Z,19Z))	metab_11610	B(ii)	0	64.1	HMDB0274103	M+K	C46H76O16P2	5.4581	-	pos	985.43293670184	0.064506841923131	48.1	-
pos_11614	Prizidilol	metab_11613	B(ii)	0	40.5	HMDB0256780	M+Na	C17H25N5O2	5.4634166666667	-	pos	354.18854545317	0.14148460419406	43.7	-
pos_11615	Ile Tyr Leu	metab_11614	B(i)	55.5	0	-	M+H	C21H33N3O5	5.4634166666667	-	pos	408.24787799379	0.034672753407304	48.3	-
pos_11662	3-Feruloyl-1-Sinapoyl Sucrose	metab_11661	B(i)	35.1	0	-	M+Na	C33H40O18	5.4794166666667	-	pos	747.20884646642	0.25928442897795	43.5	-
pos_11665	Ala Phe Leu	metab_11664	B(i)	88.8	0	-	M+H	C18H27N3O4	5.48475	-	pos	350.20515638763	0.036496514356004	53.7	-
pos_11671	Manumycin A	metab_11670	B(ii)	0	40.3	HMDB0242678	M+H	C31H38N2O7	5.48475	C12111;_	pos	551.28052877918	0.14814263580884	40.8	52665-74-4;_
pos_11672	Bipindogulomethyloside	metab_11671	B(ii)	0	48.6	HMDB0030623	M+CH3OH+H	C29H44O10	5.48475	-	pos	585.32596091411	0.19666919336717	44.7	53152-43-5
pos_11682	Phe-Pro-Ile	metab_11681	B(i)	97.1	0	HMDB0304808	M+H	C20H29N3O4	5.4901	-	pos	376.22203699284	0.009112075470464	58.3	-
pos_11683	Glu-Lys-Ile	metab_11682	B(i)	66.9	0	-	M+H	C17H32N4O6	5.4901	-	pos	389.23847751717	0.012425066818261	52.1	-
pos_11684	Ile His Glu	metab_11683	B(i)	63.2	0	-	M+H	C17H27N5O6	5.4901	-	pos	398.2040157	0.011945545600618	52	-
pos_11685	Trp-Thr-Asn	metab_11684	B(i)	46.6	0	-	M+H	C19H25N5O6	5.4901	-	pos	420.18597699387	0.048779762168195	47.1	-
pos_11690	Hydratopyrrhoxanthinol	metab_11689	B(ii)	0	60.5	HMDB0036842	M+K	C37H48O6	5.4901	-	pos	627.31000031568	0.031540810270211	48.4	120416-68-4
pos_11723	N-Cyclopentyl-2-(Diethylamino)-2-(6-Nitro-1,3-Benzodioxol-5-Yl)Acetamide	metab_11722	B(i)	63.9	0	-	M+H	C18H25N3O5	5.5060166666667	-	pos	364.18329650671	0.031126510650849	49.3	-
pos_11726	2-(Benzenesulfonyl)-N-(2-Pyridin-3-Ylethyl)-2-Azaspiro[4.5]Decane-4-Carboxamide	metab_11725	B(i)	61.5	0	-	M+H	C23H29N3O3S	5.5060166666667	-	pos	428.20060991038	0.13116392841764	46.7	-
pos_11773	Plantainoside C	metab_11772	B(i)	94.4	0	-	M+Na	C30H38O15	5.5221833333333	-	pos	661.20813569169	0.23685973526479	56	-
pos_11794	Hydroxyfluoroprednisolone Butyrate	metab_11793	B(ii)	0	50.4	HMDB0061149	M+H	C25H33FO7	5.5328333333333	-	pos	465.23104755182	0.029009066119923	45.9	1296177-38-2
pos_11796	Gentamicin C2	metab_11795	B(ii)	0	43.2	HMDB0252691	M+ACN+Na	C20H41N5O7	5.5328333333333	C02033;_	pos	527.31750207203	0.012854582873197	46.3	25876-11-3;_
pos_11807	Asn Leu Phe	metab_11806	B(i)	53.1	0	-	M+H	C19H28N4O5	5.5381833333333	-	pos	393.21157618259	0.003678552535727	45.5	-
pos_11812	Cholylarginine	metab_11811	B(ii)	0	65.5	HMDB0242375	M+Na-H2O	C30H52N4O6	5.5381833333333	-	pos	569.36402632203	0.033546236979802	48.1	-
pos_11857	Pro-Pro-Phe	metab_11856	B(ii)	0	48.9	HMDB0304811	M+H	C19H25N3O4	5.5488666666667	-	pos	360.1888041	0.060718893953562	41.9	-

pos_11944	Ile-Val-Ile	metab_11943	B(i)	90.8	0	-	M+H	C17H33N3O4	5.5754333333333	-	pos	344.25343008956	0.015471864687413	56.4	-
pos_11960	Eriojaposide B	metab_11959	B(ii)	0	82.2	HMDB0038029	M+H	C25H40O11	5.5807333333333	C17889;_	pos	517.26175594928	0.073239579498225	53.4	-;351412-97-0
pos_11981	Ps(6 Keto-Pgf1Alpha/16:0)	metab_11980	B(ii)	0	55.8	HMDB0281139	M+NH4	C42H76NO14P	5.5860833333333	-	pos	867.53834939249	0.007339319229010	48.4	-
pos_12031	Gly Ile Phe	metab_12030	B(i)	85.2	0	-	M+H	C17H25N3O4	5.6020666666667	-	pos	336.19083347373	0.026267960168903	55.2	-
pos_12034	Cgp72383	metab_12033	B(ii)	0	41.2	HMDB0013865	M+H-2H2O	C29H33N7O2	5.6020666666667	-	pos	476.25469305738	0.04424068247456	41.9	-
pos_12055	Lamiidoside	metab_12054	B(i)	64.5	0	-	M+H-2H2O	C26H32O14	5.6074166666667	-	pos	533.16136031737	0.23143868955703	48.7	-
pos_12068	Dihydrodigoxin	metab_12067	B(ii)	0	80.3	HMDB0041879	M+CH3OH+Na	C41H66O14	5.6074166666667	-	pos	837.45907073307	0.058039713512721	53	5297/10/9
pos_12079	Carvone	metab_12078	B(i)	56.7	0	HMDB0004487;HMDB0035824	M+H	C10H14O	5.6180833333333	C11383;_	pos	151.11133581258	0.11729087973341	49.1	2244-16-8;22327-39-5
pos_12082	Formyl-5-Hydroxykynurenamine	metab_12081	B(ii)	0	44.8	HMDB0012948	M+CH3OH+Na	C10H12N2O3	5.6180833333333	C05647	pos	263.10193637362	0.026990422980157	46.8	-;958733-17-0
pos_12084	Asp-Phe-Ile	metab_12083	B(i)	91.4	0	-	M+H	C19H27N3O6	5.6180833333333	-	pos	394.1961162	0.010647070173177	56.9	-
pos_12106	Jasmine Lactone	metab_12105	B(i)	83.9	0	-	M+H	C10H16O2	5.6233833333333	-	pos	169.12185840466	0.10635257202881	54.4	-
pos_12108	Trp Phe	metab_12107	B(i)	95.7	0	-	M+H	C20H21N3O3	5.6233833333333	-	pos	352.16458063889	0.010014240320795	57.6	-
pos_12139	Dg(Pgd2/A-17:0/0:0)	metab_12138	B(ii)	0	91.3	HMDB0297740	M+Na	C40H70O8	5.634	-	pos	701.49161159904	0.28687256869613	53.8	-
pos_12146	Ethyl N-[(1S)-2-Hydroxy-1-Phenylethyl]Carbamate	metab_12145	B(i)	36.8	0	-	M+H-H2O	C11H15NO3	5.6392833333333	-	pos	192.10139590037	0.007410956472207	45.9	-
pos_12162	7,8-Dimethoxy-1,3-Dihydro-2H-3-Benzazepin-2-One	metab_12161	B(i)	58.3	0	-	M+H	C12H13NO3	5.64465	-	pos	220.09627014112	0.079835247530481	49.3	-
pos_12164	Leu-Ile-Ile	metab_12163	B(i)	58.7	0	-	M+H	C18H35N3O4	5.64465	-	pos	358.26905628006	0.054720568104625	49.3	-
pos_12165	Ser Phe Phe	metab_12164	B(i)	86	0	-	M+H	C21H25N3O5	5.64465	-	pos	400.18557738686	0.014480976934895	54.4	-
pos_12166	Trp Asn Leu	metab_12165	B(i)	93.5	0	-	M+H	C21H29N5O5	5.64465	-	pos	432.22275762768	0.012271514187012	57.3	-
pos_12167	Morphiceptin	metab_12166	B(ii)	0	50.5	HMDB0005777	M+H-2H2O	C28H35N5O5	5.64465	-	pos	486.24595854353	0.052447546106558	46.6	74135-04-9
pos_12169	Gliadins	metab_12168	B(ii)	0	74.2	HMDB0252744	M+H-2H2O	C29H41N7O9	5.64465	-	pos	596.27865371629	0.024764720641721	51.7	-
pos_12195	4'-O-Beta-D-Glucosyl-5-O-Methylvisamminol	metab_12194	B(i)	87	0	-	M+H	C22H28O10	5.6553	-	pos	453.17195547185	0.034909430617898	53.5	-
pos_12199	Rhizoxin	metab_12198	B(ii)	0	42.3	HMDB0257197	M+	C35H47NO9	5.6553	-	pos	625.33019978633	0.16110359076988	42.9	-
pos_12272	3-Hydroxytetradecanedioic Acid	metab_12271	B(ii)	0	42.1	HMDB0000394	M+H-2H2O	C14H26O5	5.68755	-	pos	239.16362549113	0.012915143370744	46.3	73179-89-2
pos_12276	Arginyl-Glycyl-Aspartyl-Valine	metab_12275	B(ii)	0	42.4	HMDB0248574	M+H	C17H31N7O7	5.68755	-	pos	446.23457359508	0.094912290089107	45.8	-
pos_12287	Pro Ile Trp	metab_12286	B(i)	76.2	0	-	M+H	C22H30N4O4	5.6928833333333	-	pos	415.23305847998	0.078936103173579	52.4	-
pos_12288	Tyr Leu Phe	metab_12287	B(i)	76.2	0	-	M+H	C24H31N3O5	5.6928833333333	-	pos	442.23241569616	0.078164910319076	51.3	-
pos_12317	Dehydrocarpaine Ii	metab_12316	B(ii)	0	53.8	HMDB0030273	M+ACN+Na	C28H46N2O4	5.7035	-	pos	538.35856589055	0.049393325422248	47.5	72362-03-9
pos_12321	Trp Trp	metab_12320	B(i)	86.3	0	-	M+H	C22H22N4O3	5.7088333333333	-	pos	391.17561030513	0.086866398598673	54.9	-
pos_12351	Salicylanilide	metab_12350	B(i)	57.2	0	HMDB0257459	M+NH4	C13H11NO2	5.7194333333333	C18915	pos	231.11228032607	0.055948663769739	50.1	87-17-2;_
pos_12354	Pro Asp Ala Lys Ser	metab_12353	B(i)	48.1	0	-	M+H	C21H36N6O9	5.7194333333333	-	pos	517.26783904136	0.013233767086872	43.8	-
pos_12368	Riboprine	metab_12367	B(i)	91.6	0	HMDB0257214	M+H	C15H21N5O4	5.7300333333333	-	pos	336.16580251321	0.078153583893651	55.3	-
pos_12373	Hydroxyhomodestruxin B	metab_12372	B(ii)	0	42.2	HMDB0039897	M+Na-H2O	C31H53N5O8	5.7300333333333	-	pos	628.3650185	0.091214245779349	44.3	-
pos_12381	4-[[2-(1-Hydroxybut-2-Enyl)-5-Oxooxolan-3-Yl]Amino]Benzoic	metab_12380	B(i)	36.7	0	-	M+H	C15H17NO5	5.7353833333333	-	pos	292.12061769531	0.033641270513283	42.9	-
pos_12396	Pengitoxin	metab_12395	B(ii)	0	41.5	HMDB0256225	M+K	C51H74O19	5.7353833333333	-	pos	1029.4418861773	0.055868031949146	44.3	-
pos_12399	Phe Ala Trp	metab_12398	B(i)	89.9	0	-	M+H	C23H26N4O4	5.7406833333333	-	pos	423.20155194656	0.018412086534389	56.3	-
pos_12406	Tyr-Pro-Ile	metab_12405	B(i)	61.3	0	-	M+ACN+H	C20H29N3O5	5.7460333333333	-	pos	433.2433947	0.055838600312376	49.4	-
pos_12426	Val Ile Trp	metab_12425	B(i)	82.3	0	-	M+H	C22H32N4O4	5.7513166666667	-	pos	417.24879553577	0.056623451847466	53	-
pos_12457	2-Norbornene-5,6-Dicarboxylic Anhydride	metab_12456	B(i)	46.8	0	-	M+NH4	C9H8O3	5.7566166666667	-	pos	182.08066528255	0.075772322961002	47.1	-
pos_12482	Val Pro Trp	metab_12481	B(i)	61.8	0	-	M+ACN+Na	C21H28N4O4	5.7622166666667	-	pos	464.22513284277	0.15546063771967	47.6	-
pos_12503	2'-(E)-Feruloyl-3-(Arabinosylxylose)	metab_12502	B(ii)	0	74.8	HMDB0030230	M+H	C20H26O12	5.7675166666667	-	pos	459.14975754432	0.1846900958253	53.8	-
pos_12517	Isobyakangelicin	metab_12516	B(i)	53.2	0	-	M+H	C17H18O7	5.7728166666667	-	pos	335.11168136456	0.12864875940563	48.7	-
pos_12532	Homovanillic Acid	metab_12531	B(i)	66.8	0	HMDB0000118;PW_C000074	M+H-H2O	C9H10O4	5.7781333333333	C05582	pos	165.05418176985	0.080619165325057	52.1	306-08-1
pos_12534	1-O-Caffeoylglucose	metab_12533	B(ii)	0	65.8	HMDB0036937	M+H-H2O	C15H18O9	5.7781333333333	C10433	pos	325.09100273223	0.12586591812868	51.3	14364-08-0
pos_12537	Robustaside B	metab_12536	B(i)	68.6	0	-	M+H	C21H22O10	5.7781333333333	-	pos	435.12738668052	0.2458989467474	51.2	-

pos_12569	Flunisolide	metab_12568	B(i)	51.5	0	HMDB0014326	M+NH4	C24H31FO6	5.7834166666667	C07005	pos	452.24661447777	0.14233863626237	46.4	3385/3/3;3385-03-3
pos_12588	Periplocin	metab_12587	B(ii)	0	71.7	HMDB0256343	M+ACN+H	C36H56O13	5.7834166666667	-	pos	738.40586376644	0.017566660597584	50.5	-
pos_12617	4-Formyl Indole	metab_12616	B(i)	93.9	0	-	M+H	C9H7NO	5.7940333333333	-	pos	146.05964688604	0.017456546663977	56.2	-
pos_12634	Phe-Val	metab_12633	B(i)	87.6	0	HMDB0029008	M+H-H2O	C14H20N2O3	5.7993833333333	-	pos	247.14352898562	0.15618575352187	55.3	3918-90-9
pos_12662	5-(6-Methyl-6-Hydroxyoctyl)Furan-2(5H)-One	metab_12661	B(i)	63.5	0	-	M+H-H2O	C13H22O3	5.8046833333333	-	pos	209.15313593052	0.1284255382838	51	-
pos_12693	Tricyclazole	metab_12692	B(i)	93.4	0	HMDB0031809	M+H	C9H7N3S	5.8099833333333	C18492	pos	190.04288701768	0.037256084235199	55.4	41814-78-2
pos_12752	Lys-Thr-Asn	metab_12751	B(i)	60.1	0	-	M+H-H2O	C14H27N5O6	5.8153	-	pos	344.19603797603	0.003717242311683	49.2	-
pos_12757	Lys Asp Leu Ser Leu	metab_12756	B(i)	57.1	0	-	M+H	C25H46N6O9	5.8153	-	pos	575.3383305	0.21990741	45.3	-
pos_12823	Dihydrovaltrate	metab_12822	B(ii)	0	48.2	HMDB0034492	M+H	C22H32O8	5.8206166666667	-	pos	425.21533079068	0.17288896117984	47.4	18296-45-2
pos_12912	Eutypine	metab_12911	B(ii)	0	48	HMDB0301834	M+CH3OH+H	C12H10O2	5.8312333333333	C08448	pos	219.10093380905	0.038700923619618	48	121007-17-8;_
pos_12915	Hypochoeroside A	metab_12914	B(ii)	0	63.6	HMDB0303759	M+H	C21H32O9	5.8312333333333	-	pos	429.2096798	0.044484483412277	49.8	-
pos_12916	Ile-Ile-Ile-Pro	metab_12915	B(ii)	0	56.1	HMDB0304800	M+H	C23H42N4O5	5.8312333333333	-	pos	455.32156684659	0.040714998506726	49.4	-
pos_12920	Asterinin D	metab_12919	B(i)	41.6	0	-	M+H	C25H33N5O7	5.8312333333333	-	pos	516.24204781739	0.024245004905301	44.4	-
pos_12968	Thr Arg Glu Glu Lys	metab_12967	B(i)	74.2	0	-	M+H	C26H47N9O11	5.83655	-	pos	662.35335601449	0.071918072811123	49.1	-
pos_12990	Perillyl Alcohol	metab_12989	B(i)	80.4	0	HMDB0003634	M+H	C10H16O	5.8418333333333	C02452	pos	153.12703034433	0.081155740361357	54.6	18457-55-1;536-59-4
pos_13038	Ponasteroside A	metab_13037	B(ii)	0	63.4	HMDB0034091	M+ACN+Na	C33H54O11	5.8472	-	pos	690.38079275917	0.16163014721701	47.4	20117-33-3
pos_13060	Phe-Val-Phe	metab_13059	B(i)	83.3	0	-	M+H	C23H29N3O4	5.8525333333333	-	pos	412.22187494828	0.042654749684665	54.1	-
pos_13100	N-Docosahexaenoyl Histidine	metab_13099	B(ii)	0	40.8	HMDB0242015	M+CH3OH+Na	C28H39N3O3	5.8578833333333	-	pos	520.31211057351	0.027396182412541	43.4	_;
pos_13147	Ile Phe Leu	metab_13146	B(i)	86.1	0	-	M+H	C21H33N3O4	5.8685	-	pos	392.25317831274	0.014212454656979	55.4	-
pos_13179	Ser-Tyr-Leu	metab_13178	B(i)	36.6	0	-	M+NH4	C18H27N3O6	5.8737833333333	-	pos	399.22268514349	0.022249687006997	45.7	-
pos_13192	3-Feruloyl-1,5-Quinolactone	metab_13191	B(ii)	0	63.2	HMDB0029289	2M+H	C17H18O8	5.8737833333333	-	pos	701.20345429675	0.16901014294022	49.4	-
pos_13217	Iodamide	metab_13216	B(ii)	0	41.1	HMDB0253510	M+H-2H2O	C12H11I3N2O	5.8790833333333	-	pos	592.76864550104	0.064355706285133	46.2	-
pos_13230	Vinfosiltine	metab_13229	B(ii)	0	49.3	HMDB0259816	M+Na	C51H72N5O10	5.8790833333333	-	pos	968.49131600996	0.11734651825598	47.2	-
pos_13318	Damascenone	metab_13317	B(i)	46.9	0	HMDB0013804	M+H	C13H18O	5.8949666666667	-	pos	191.14255998534	0.041789375962211	47.6	23696-85-7
pos_13319	L-Phenylalanine, Butyl Ester	metab_13318	B(i)	89.4	0	-	M+H	C13H19NO2	5.8949666666667	-	pos	222.14834082183	0.090800886651712	55.6	-
pos_13356	Tryptophol	metab_13355	B(i)	98.9	0	HMDB0003447	M+H	C10H11NO	5.9002666666667	C00955	pos	162.09090065258	0.009124364514025	58.7	526-55-6
pos_13399	Delta-Cehc	metab_13398	B(i)	36.8	0	HMDB0242106	M+H	C14H18O4	5.9056	-	pos	251.1250415	0.045947401036994	42.9	-
pos_13417	Cyclo(Pro-Phe-D-Trp-Lys-Thr-Phe)	metab_13416	B(ii)	0	63.5	HMDB0250003	M+Na-H2O	C44H54N8O7	5.9056	-	pos	811.38436001397	0.059536742309091	45.4	-
pos_13442	Thalicarpine	metab_13441	B(i)	35.6	0	-	M+H	C41H48N2O8	5.9109	C09655	pos	697.35077798735	0.14084383949786	43.3	5373-42-2
pos_13459	Flavonol 3-O-D-Glucoside	metab_13458	B(ii)	0	59	HMDB0252295	M+CH3OH+H	C21H20O8	5.9161833333333	C03946;_	pos	433.14575592785	0.045959211849909	48.9	-;_
pos_13488	Ile-Tyr-Lys	metab_13487	B(i)	57.2	0	-	M+H	C21H34N4O5	5.9215	-	pos	423.25641243642	0.033928326701483	47.7	-
pos_13514	Eriojaposide A	metab_13513	B(ii)	0	72.3	HMDB0038028	M+H	C24H38O11	5.92705	C17888;_	pos	503.24643316505	0.09075856833972	51.3	-;290308-51-9
pos_13548	Glu-Leu-Leu	metab_13547	B(i)	87.3	0	-	M+H-H2O	C17H31N3O6	5.9376333333333	-	pos	356.21717916988	0.014972877048947	55.8	-
pos_13571	4-Piperidinone, 1-Hydroxy-2,2,6,6-Tetramethyl-	metab_13570	B(i)	52.7	0	-	M+H	C9H17NO2	5.9429333333333	-	pos	172.13277042058	0.027419854194124	48.5	-
pos_13576	5Z-7-Oxozeaenol	metab_13575	B(ii)	0	45.4	HMDB0247027	M+H-H2O	C19H22O7	5.9429333333333	-	pos	345.13223230775	0.045477557948169	47.6	-
pos_13577	N-(Furan-2-Ylmethyl)-1-[(4-Methoxyphenyl)Methyl]Benzotriazole-5-Carboxamide	metab_13576	B(i)	41.4	0	-	M+H	C20H18N4O3	5.9429333333333	-	pos	363.14284527631	0.099756683961993	45.3	-
pos_13585	Gmp-N-Epsilon-(N-Alpha-Acetyl Lysine Methyl Ester) 5'-Phosphoramidate	metab_13584	B(ii)	0	75.5	HMDB0304375	M+H	C19H29N7O10P-	5.9429333333333	-	pos	547.1770986	0.024907358081881	53.8	-
pos_13596	Lacto-N-Tetraose	metab_13595	B(ii)	0	66.8	HMDB0006566	M+H	C26H45NO21	5.9429333333333	_;C06371	pos	708.25813288504	0.2485028184903	50.4	-;7578-24-7
pos_13608	Glycinoeclepin A	metab_13607	B(ii)	0	47.6	HMDB0037037	M+ACN+H	C25H34O7	5.9483	C08765	pos	488.26739590105	0.067356084488047	44.7	83216-10-8
pos_13618	Indolophenanthridine	metab_13617	B(ii)	0	65.7	HMDB0253475	M+NH4	C19H18N2	5.9535833333333	-	pos	292.18005254468	0.048537713843183	50.9	-
pos_13620	Dacinostat	metab_13619	B(ii)	0	46.7	HMDB0250821	M+H	C22H25N3O3	5.9535833333333	-	pos	380.19578968416	0.00988346898514	47.6	-
pos_13637	Fasoracetam	metab_13636	B(ii)	0	50	HMDB0252174	M+H	C10H16N2O2	5.9588833333333	C13311;_	pos	197.12794052312	0.085274456236095	48.2	110958-19-5;_
pos_13657	Prenyl Caproate	metab_13656	B(ii)	0	54.7	HMDB0032489	M+NH4	C11H20O2	5.9641833333333	C13422	pos	202.17964163323	0.052465267959184	49.7	76649-22-4

pos_13688	1-Phenylpropane-1,2-Dione	metab_13687	B(i)	90	0	HMDB0035243	M+NH4	C9H8O2	5.9747666666667	C17268	pos	166.08584097244	0.050401821941595	55.4	579-07-7
pos_13701	Ganosporelactone A	metab_13700	B(ii)	0	40.4	HMDB0036406	M+H-H2O	C30H40O7	5.9747666666667	-	pos	495.27515788019	0.031645025389112	43.7	138008-04-5
pos_13710	Pgp(Pgd1/I-22:0)	metab_13709	B(ii)	0	78.4	HMDB0275917	M+Na-H2O	C48H90O16P2	5.9747666666667	-	pos	989.5425893	0.022341904758097	53	-
pos_13712	1-[4-(Ethylsulfonylamino)Phenyl]-N-(2-Methylpropyl)Cyclohexane-1-Carboxamide	metab_13711	B(i)	58.3	0	-	M+H	C19H30N2O3S	5.98005	-	pos	367.20809290912	0.021653350972198	47.2	-
pos_13734	3-Hydroxy-4-[3-(4-Methoxyphenyl)Propanoyl]Phenyl Beta-D-Mannopyranoside	metab_13733	B(i)	84.9	0	-	M+H	C22H26O9	5.9853666666667	-	pos	435.16133285056	0.011139602550487	53.8	-
pos_13751	Caldiamide	metab_13750	B(ii)	0	45.1	HMDB0251634	M+H	C16H29N5O8	5.9906666666667	-	pos	420.21168426562	0.031764280461445	46.8	-
pos_13774	Koninginin A	metab_13773	B(i)	77.5	0	-	M+H	C16H28O4	6.0013166666667	-	pos	285.20529083076	0.034305314069141	53.8	-
pos_13802	Lonfuranacid A	metab_13801	B(i)	36.1	0	-	M+Na-H2O	C12H20O5	6.0120166666667	-	pos	249.10931114784	0.10612229309713	44.2	-
pos_13806	Ptesculentoside	metab_13805	B(i)	87.8	0	-	M+Na-H2O	C20H30O9	6.0120166666667	-	pos	419.16646871786	0.087615525433408	54.9	-
pos_13809	4-Methylphthalic Anhydride	metab_13808	B(i)	87	0	-	M+H	C9H6O3	6.0173666666667	-	pos	163.03851055856	0.05900605257518	55.7	-
pos_13824	4-Methyl-1H-Benzotriazole	metab_13823	B(i)	86.9	0	-	M+H	C7H7N3	6.0227166666667	-	pos	134.07091854756	0.043311960359193	55.2	-
pos_13832	Cytochalasin B	metab_13831	B(i)	57	0	-;HMDB0250718;LMPK11000002	M+H	C29H37NO5	6.0227166666667	C19954;_	pos	480.27641253699	0.061682784573051	48.8	14930-96-2;_;
pos_13834	Asn Tyr Phe Glu	metab_13833	B(i)	37.3	0	-	M+H	C27H33N5O9	6.0227166666667	-	pos	572.23339184302	0.12729240376944	44.3	-
pos_13841	(S)-11,12,13-Trinor-7-Calamenone	metab_13840	B(ii)	0	57.5	HMDB0040823	M+H	C12H14O	6.0280333333333	-	pos	175.11122149349	0.084800573629519	49.2	155748-76-8
pos_13842	Thymyl Acetate	metab_13841	B(i)	40.6	0	HMDB0303499	M+H	C12H16O2	6.0280333333333	C09909	pos	193.12181714393	0.029498747427722	46.1	528-79-0;_
pos_13843	Jasmonic Acid	metab_13842	B(i)	70.8	0	HMDB0032797;LMFA02020001	M+H	C12H18O3	6.0280333333333	C08491	pos	211.13233334364	0.027638202242934	52.4	6894-38-8;59366-47-1;
pos_13852	Menthofuran	metab_13851	B(i)	70.2	0	HMDB0036089;LMPR0102090020	M+H-H2O	C10H14O	6.0333333333333	C09868;C18025	pos	133.100823	0.067116118943782	52	494-90-6;
pos_13861	Delta17-6-Keto Prostaglandin F1Alpha	metab_13860	B(i)	83.5	0	-	M+H	C20H32O6	6.0333333333333	-	pos	369.22361505982	0.006680219284904	53.6	-
pos_13872	9-Oxootre	metab_13871	B(i)	71.3	0	-	M+H	C18H28O3	6.0386666666667	-	pos	293.21016599461	0.007833217593308	53.3	-
pos_13873	Sterebin A	metab_13872	B(i)	76.8	0	HMDB0035337	M+H	C18H30O4	6.0386666666667	-	pos	311.22028116325	0.006286304919092	53.2	107647-14-3
pos_13874	Tricin	metab_13873	B(i)	92.5	0	-;LMPK12110873	M+H	C17H14O7	6.0386666666667	C10193	pos	331.08028573835	0.077859238744756	56.1	520-32-1;
pos_13886	Dl-Ornithino-L-Alanine	metab_13885	B(ii)	0	62.9	HMDB0029448	M+CH3OH+H	C8H17N3O4	6.0440166666667	-	pos	252.15634192627	0.031976147997825	51.3	25693-39-4
pos_13888	Caryophyllene Oxide	metab_13887	B(i)	38	0	HMDB0036789	M+CH3OH+Na	C15H24O	6.0440166666667	C16908	pos	275.19971800621	0.031048913315802	45	1139-30-6
pos_13895	Rifametane	metab_13894	B(ii)	0	41.3	HMDB0257224	M+ACN+H	C44H60N4O12	6.0440166666667	-	pos	878.46138557848	0.17343980792904	43.8	-
pos_13900	Decylamine Oxide	metab_13899	B(i)	76	0	-	M+H	C12H27NO	6.0493166666667	-	pos	202.21600237614	0.071699414089915	53.1	-
pos_13902	3-(6-Methylheptyl)-3,6-Dihydro-1H-Furo[3,4-C]Furan-4-One	metab_13901	B(i)	68.8	0	-	M+H	C14H22O3	6.0493166666667	-	pos	239.16354704043	0.015502198943526	52.3	-
pos_13907	(+)-15,16-Dihydroxyoctadecanoic Acid	metab_13906	B(ii)	0	45.3	HMDB0031008	M+NH4	C18H36O4	6.0493166666667	-	pos	334.29425866846	0.062900904278236	46.8	-
pos_13908	1,8,10-Trihydroxy-3-Methoxy-6-Methyl-10-(3-Methylbut-2-Enyl)Anthracen-9-One	metab_13907	B(i)	44.6	0	-	M+H	C21H22O5	6.0493166666667	-	pos	355.15296755686	0.028121646521915	47.3	-
pos_13915	L-Fucose	metab_13914	B(ii)	0	65.7	HMDB0000174	M+H-2H2O	C6H12O5	6.0546166666667	C01019	pos	129.05427149912	0.18348848184127	51.2	2438-80-4;6696-41-9
pos_13921	2,6-Di-Tert-Butyl-P-Benzoquinone	metab_13920	B(i)	45	0	HMDB0013817	M+H	C14H20O2	6.0546166666667	-	pos	221.15302571956	0.011545403590935	47.1	719-22-2
pos_13926	Decanoyl-L-Carnitine	metab_13925	B(i)	72.1	0	HMDB0000651	M+H	C17H33NO4	6.0546166666667	C03299	pos	316.24734449346	0.12587225127245	51.5	1492-27-9
pos_13976	(2S)-2-(2,6-Dihydroxyphenyl)-5,7-Dihydroxy-2,3-Dihydrochromen-4-One	metab_13975	B(i)	48.5	0	-	M+H-H2O	C15H12O6	6.07585	-	pos	271.05929978757	0.035089957224314	47.5	-
pos_13980	Teumarin	metab_13979	B(i)	67.3	0	-	M+H-H2O	C22H28O8	6.07585	-	pos	403.17197691272	0.034520220833475	49.3	-
pos_13989	(Z)-6,9,10-Trihydroxyoctadec-7-Enoic Acid	metab_13988	B(i)	64.2	0	-	M+H	C18H34O5	6.0811833333333	-	pos	331.24694702208	0.03523107180505	50.9	-
pos_13993	Prostaglandin D2-1-Glyceryl Ester	metab_13992	B(ii)	0	68.5	HMDB0013653	M+H	C23H38O7	6.0811833333333	-	pos	427.26528668576	0.01784651466176	50.1	-
pos_14021	2-(2-Butoxyethoxy)Ethyl Acetate	metab_14020	B(i)	49.2	0	-	M+H	C10H20O4	6.1026166666667	-	pos	205.14283014717	0.059517195685641	47	-
pos_14022	Cer(8:1_2O/10:0)	metab_14021	B(i)	46.6	0	-	M+H	C18H35NO3	6.1026166666667	-	pos	314.26793379652	0.036538834630047	45.5	-
pos_14035	4-Dodecylmorpholine	metab_14034	B(i)	40.2	0	-	M+H	C16H33NO	6.1132833333333	-	pos	256.26282564285	0.034422701583747	45.6	-
pos_14036	Spb(16:1_2O)	metab_14035	B(i)	78	0	-	M+H	C16H33NO2	6.1132833333333	-	pos	272.2576423	0.02053563381114	54.5	-
pos_14038	Monic Acid	metab_14037	B(ii)	0	76.7	HMDB0061154	M+Na-H2O	C18H30O6	6.1132833333333	-	pos	347.18010521945	0.033858545727405	50.1	-
pos_14044	Spb(14:0_2O)	metab_14043	B(i)	84.5	0	-	M+H	C14H31NO2	6.1185833333333	-	pos	246.24206340114	0.10585407805218	54.8	-
pos_14061	Heptadecasphinganine	metab_14060	B(i)	62	0	LMSP01040003	M+H	C17H37NO2	6.1344666666667	-	pos	288.28886284945	0.027294165900466	50	0

pos_14066	1,5-Dimethyl-1-Vinyl-4-Hexenyl Acetate	metab_14065	B(i)	35.6	0	HMDB0039522	M+H-2H2O	C12H20O2	6.1397666666667	C09863	pos	161.13198208931	0.034643135745953	44.7	115-95-7
pos_14068	Dodecatrienoic Acid	metab_14067	B(ii)	0	44.7	HMDB0302622	M+H	C12H18O2	6.1397666666667	-	pos	195.13739543931	0.011634511642156	47.3	-
pos_14069	7-Oxo-11-Dodecenoic Acid	metab_14068	B(i)	56.1	0	LMFA01060182	M+H	C12H20O3	6.1397666666667	-	pos	213.14791972183	0.009821916058695	49.6	0
pos_14092	(Z)-9,10,11-Trihydroxyoctadec-12-Enoic Acid	metab_14091	B(i)	69.4	0	-	M+NH4	C18H34O5	6.1451166666667	-	pos	348.27343200796	0.077041977644586	52.2	-
pos_14093	Kanzonol N	metab_14092	B(ii)	0	51	HMDB0041100	M+H	C22H24O6	6.1451166666667	-	pos	385.16582530387	0.18891776150961	47.3	156250-71-4
pos_14105	(-)-Trans-C75	metab_14104	B(i)	40.8	0	-	M+H	C14H22O4	6.1504	-	pos	255.15628242795	0.049889398089512	43	-
pos_14117	Beta-Ionone	metab_14116	B(i)	42.6	0	HMDB0036565	M+H	C13H20O	6.1556666666667	C12287	pos	193.15816253411	0.02064538118102	46.4	79-77-6
pos_14118	Kobusone	metab_14117	B(i)	70.2	0	-;HMDB0036790	M+H	C14H22O2	6.1556666666667	_;C16983	pos	223.16864663201	0.052010263020554	52	-;24173-71-5
pos_14142	Styrene	metab_14141	B(i)	38	0	-;HMDB0034240	M+H	C8H8	6.16635	C07083;C19506	pos	105.06958264051	0.057338472156482	46.3	100-42-5
pos_14151	Benzoyl-Dl-Arginine-Naphthylamide	metab_14150	B(ii)	0	49.4	HMDB0257846	M+H	C23H25N5O2	6.16635	-	pos	404.20563732929	0.014698146931918	47.3	-
pos_14164	(2-Dodecenyl)Succinic Anhydride	metab_14163	B(i)	80.2	0	-	M+H	C16H26O3	6.1717	-	pos	267.19472099371	0.01496496897941	54.7	-
pos_14189	N,N-Dimethyltetradecylamine-N-Oxide	metab_14188	B(i)	82.7	0	-	M+H	C16H35NO	6.1823333333333	-	pos	258.27837074194	0.10095084256205	54.5	-
pos_14218	Tetranor-12R-Hete	metab_14217	B(i)	87.4	0	LMFA01050143	M+H-H2O	C16H26O3	6.1983	-	pos	249.18415712782	0.006392152435864	55.2	0
pos_14226	Cer(8:1_2O/14:1)	metab_14225	B(i)	82.8	0	-	M+H	C22H41NO3	6.1983	-	pos	368.31378288876	0.17801651194323	52.5	-
pos_14237	7-Hydroxy-5-(2-Hydroxypentyl)-2,2-Dimethyl-4,11-Dioxabicyclo[6.2.1]Undecan-3-One	metab_14236	B(i)	50.8	0	-	M+H-H2O	C16H28O5	6.2035833333333	-	pos	283.18878113397	0.023850511662228	46.9	-
pos_14264	Octadecenedioic Acid	metab_14263	B(i)	56.5	0	HMDB0304442	M+H-2H2O	C18H32O4	6.2142166666667	-	pos	277.21542776331	0.011211045925436	49.9	-
pos_14265	9-Oxoode	metab_14264	B(i)	86.3	0	HMDB0004669;LMFA02000251;LMFA02000274	M+H	C18H30O3	6.2142166666667	C14766;_	pos	295.22566171257	0.037636528907498	55.8	-;_;
pos_14279	Dihomo-Gamma-Linolenic Acid	metab_14278	B(i)	69	0	HMDB0002925;HMDB0247333	M+NH4	C20H34O2	6.2195	C03242;_	pos	324.28789416686	0.04896642698336	50.4	1783-84-2;_
pos_14284	4-Methylstyrene	metab_14283	B(i)	58.2	0	HMDB0246518	M+H	C9H10	6.2247833333333	-	pos	119.0851867	0.029357662963051	49.4	-
pos_14290	Trp Ala Arg	metab_14289	B(i)	40.5	0	-	M+H	C20H29N7O4	6.2247833333333	-	pos	432.23663629513	0.021602735217979	46.7	-
pos_14296	Sclareolide	metab_14295	B(i)	66.2	0	HMDB0035293	M+H	C16H26O2	6.2301166666667	-	pos	251.19979601242	0.019394915293204	50.6	564-20-5
pos_14312	1-Hexadecylamine	metab_14311	B(i)	38.1	0	-	M+H	C16H35N	6.2407666666667	-	pos	242.2836125	0.018780594897572	45.3	-
pos_14317	9-Hexadecenoic Acid	metab_14316	B(i)	83	0	HMDB0247596	M+H	C16H30O2	6.2461166666667	-	pos	255.23111963907	0.11767665549645	53.6	-
pos_14319	Dhv-Pge2	metab_14318	B(ii)	0	46.5	HMDB0251112	M+H	C22H34O5	6.2461166666667	-	pos	379.24445482009	0.021790362051054	45.8	-
pos_14322	12-Hydroxydodecanoic Acid	metab_14321	B(ii)	0	75.2	HMDB0002059	M+Na-H2O	C12H24O3	6.25145	C08317	pos	221.15298355307	0.075427467438559	52.2	505-95-3
pos_14325	Dihydroceramide C2	metab_14324	B(i)	42.5	0	-	M+H	C20H41NO3	6.25145	-	pos	344.31483335956	0.072881217874642	46.7	-
pos_14360	(3S)-3-Hydroxycyclocitral	metab_14359	B(ii)	0	56.9	HMDB0303972	M+CH3OH+H	C10H16O2	6.2674833333333	C19731	pos	201.1479581	0.004869785667929	50	-;_
pos_14365	15(S)-Hydroxyeicosatrienoic Acid	metab_14364	B(ii)	0	54.9	HMDB0005045	M+H	C20H34O3	6.2674833333333	-	pos	323.25690568395	0.022087467425205	49.1	13-16-1
pos_14368	2-(7,8-Dihydroxypentadecyl)-6-Hydroxybenzoic Acid	metab_14367	B(i)	52.6	0	-	M+H	C22H36O5	6.2674833333333	-	pos	381.26000357988	0.003925510345862	47.8	-
pos_14378	Isokobusone	metab_14377	B(i)	54.7	0	-;HMDB0036791	M+H	C14H22O2	6.2728333333333	C16977;_	pos	223.16860646649	0.053100061060504	48.4	-;24173-72-6
pos_14381	15-Oxoede	metab_14380	B(i)	60.9	0	-	M+H-H2O	C20H34O3	6.2728333333333	-	pos	305.2466157	0.031029181618743	49.8	-
pos_14384	Grayanotoxin	metab_14383	B(ii)	0	41.1	HMDB0246239	M+H	C20H34O5	6.2728333333333	-	pos	355.24443642844	0.012584785729028	44.3	-
pos_14393	1-(4-Hydroxy-3-Methoxyphenyl)-5-Methoxydecan-3-One	metab_14392	B(i)	52	0	-	M+H	C18H28O4	6.2781666666667	-	pos	309.2030383	0.019390551607357	47	-
pos_14395	L-Norleucine	metab_14394	B(i)	86.7	0	HMDB0001645	M+H	C6H13NO2	6.2835166666667	C01933	pos	132.10152671363	0.080880748351452	55.3	327-57-1
pos_14418	Choldienic Acid	metab_14417	B(i)	91.5	0	-	M+H	C24H36O3	6.2888166666667	-	pos	373.27205874951	0.071184482249074	54.7	-
pos_14429	1-Octadecanamine	metab_14428	B(i)	36.8	0	HMDB0029586	M+H	C18H39N	6.29415	-	pos	270.31474684702	0.23728245451609	45.5	124-30-1
pos_14430	(4E,14Z)-2-Aminooctadeca-4,14-Diene-1,3-Diol	metab_14429	B(ii)	0	42	HMDB0242112	M+H	C18H35NO2	6.29415	-	pos	298.27317375437	0.092384218087849	46.2	-
pos_14437	4-Methylbenzophenone	metab_14436	B(i)	89.7	0	-	M+H	C14H12O	6.2994333333333	-	pos	197.09554772453	0.12535220079093	55.7	-
pos_14444	4-(4-Methylcyclohexyl)-4-Oxobutanoic Acid	metab_14443	B(ii)	0	43.3	HMDB0253742	M+H	C11H18O3	6.3047833333333	-	pos	199.13238634176	0.042492878238091	46.6	-
pos_14448	(8E,12Z)-10-Hydroxyoctadeca-8,12-Dienoic Acid	metab_14447	B(i)	41.8	0	-	M+H	C18H32O3	6.3047833333333	-	pos	297.24151526744	0.012125481335634	46.6	-
pos_14449	12,13-Dihome	metab_14448	B(i)	52.7	0	LMFA02000230	M+H	C18H34O4	6.3047833333333	C14829	pos	315.25210517637	0.004102773303302	48.4	0
pos_14454	Diethyl Azelate	metab_14453	B(i)	91.2	0	-	M+H	C13H24O4	6.3100833333333	-	pos	245.17407059536	0.008920456276864	56.5	-
pos_14456	Nandrolone	metab_14455	B(i)	58.3	0	HMDB0002725;LMST02010044	M+H	C18H26O2	6.3100833333333	C07254	pos	275.19977833605	0.012374891056976	49.1	434-22-0;
pos_14457	12-Opda	metab_14456	B(i)	61.6	0	HMDB0301804	M+H	C18H28O3	6.3100833333333	C01226	pos	293.20997980926	0.009179615545493	50.3	-
pos_14458	7(14)-Bisabolene-2,3,10,11-Tetrol	metab_14457	B(ii)	0	74.1	HMDB0035918	M+CH3OH+Na	C15H28O4	6.3100833333333	-	pos	327.21346023392	0.015487005011625	52.7	122470-42-2

pos_14496	Dg(5-Iso Pgf2Vi/0:0/A-15:0)	metab_14495	B(ii)	0	48.8	HMDB0297570	M+Na	C36H64O8	6.3366833333333	-	pos	647.45544435303	0.12874086590797	47.1	-
pos_14498	N-Acetyl-B-Glucosaminylamine	metab_14497	B(ii)	0	86.3	HMDB0001104	M+CH3OH+H	C8H16N2O5	6.3419833333333	C01239	pos	253.14009825037	0.1019593136468	54.6	-
pos_14500	Tsangane L 3-Glucoside	metab_14499	B(ii)	0	49.9	HMDB0040824	M+H-H2O	C19H34O7	6.3419833333333	-	pos	357.22349725481	0.02277869569605	45.5	143775-68-2
pos_14534	T2 Triol	metab_14533	B(ii)	0	59.6	HMDB0035396	M+H	C20H30O7	6.3632166666667	-	pos	383.20328366105	0.085767947833543	47.9	34114-98-2
pos_14553	Lpc(18:3-Sn1)	metab_14552	B(i)	84.4	0	-	M+H	C26H48NO7P	6.3685	-	pos	518.32271154519	0.02758571705087	55.3	-
pos_14566	Phthalic Anhydride	metab_14565	B(i)	87.6	0	HMDB0256501	M+H	C8H4O3	6.3791666666667	-	pos	149.0228695	0.017580043152869	55.9	-
pos_14567	Diethyl Phthalate	metab_14566	B(i)	85.6	0	HMDB0094660	M+H-H2O	C12H14O4	6.3791666666667	C14175	pos	205.08533754924	0.034282676149827	55	84-66-2;_
pos_14580	1-Monolinolenoyl-Rac-Glycerol	metab_14579	B(ii)	0	47.6	HMDB0252853	M+H	C21H36O4	6.3845333333333	-	pos	353.2674715	0.04142685844081	47.2	-
pos_14592	N-(Cyclohexylmethyl)-3-[1-(2-Methylphenyl)Sulfonylpiperidin-3-Yl]Propanamide	metab_14591	B(i)	60.1	0	-	M+H	C22H34N2O3S	6.3898333333333	-	pos	407.23913877318	0.024399920154029	46.5	-
pos_14595	Senegenin	metab_14594	B(ii)	0	76.5	HMDB0258232	M+H	C30H45ClO6	6.3898333333333	-	pos	537.30169989275	0.049605978030936	49.3	-
pos_14598	Notoginsenoside R2	metab_14597	B(i)	53.4	0	HMDB0035362	M+H	C41H70O13	6.3898333333333	-	pos	771.48383653663	0.085805132257821	46.6	80418-25-3
pos_14602	13-Hotre	metab_14601	B(i)	87.9	0	LMFA02000051	M+H	C18H30O3	6.3951166666667	C16316	pos	295.22588736064	0.003923682864281	55.8	0
pos_14604	Tetrahydrodeoxycortisol	metab_14603	B(ii)	0	53.9	HMDB0005972	M+H	C21H34O4	6.3951166666667	C14594	pos	351.24959017174	0.032143128217125	47.3	68-60-0;
pos_14605	Buprenorphine	metab_14604	B(i)	66.2	0	HMDB0015057;PW_C009368	M+H	C29H41NO4	6.3951166666667	C08007	pos	468.3071734	0.015275037917754	49.6	52485-79-7
pos_14614	8-(3-Hydroxybutyl)-1,5-Dimethyl-6-Oxabicyclo[3.2.1]Octan-3-Ol	metab_14613	B(i)	62.7	0	-	M+H-2H2O	C13H24O3	6.4004166666667	-	pos	193.15818408095	0.15536504340659	50.7	-
pos_14629	(Ent-2Alpha,3Beta,15Beta)-16-Kaurene-2,3,15-Triol	metab_14628	B(ii)	0	41.6	HMDB0038689	M+H	C20H32O3	6.4057	-	pos	321.24131514775	0.061592290051039	45	140636-09-5
pos_14659	Norbinaltorphimine	metab_14658	B(ii)	0	44.9	HMDB0255724	M+H	C40H43N3O6	6.4272	_;C18130	pos	662.32534717204	0.056826094518353	44.5	105618-26-6;_
pos_14662	13(S)-Hydroperoxylinolenic Acid	metab_14661	B(ii)	0	40.1	HMDB0301803	M+H-H2O	C18H30O4	6.4324833333333	C04785	pos	293.20833418995	0.025953220190272	43.5	67597-26-6
pos_14668	Pc(18:2/0:0)	metab_14667	B(i)	71.4	0	LMGP01050034	M+H	C26H50NO7P	6.4324833333333	-	pos	520.33808507246	0.041386633533961	53.2	0
pos_14673	Prostaglandin A1	metab_14672	B(i)	36.5	0	HMDB0002656	M+H-H2O	C20H32O4	6.4377666666667	C04685	pos	319.22354561657	0.011046675026905	43.8	14152-28-4
pos_14679	25(R)-Hydroxyprotopanaxadiol	metab_14678	B(ii)	0	40.7	HMDB0245691	M+K	C30H54O4	6.4377666666667	-	pos	517.36915445587	0.12344025036212	43.6	-
pos_14698	Pe(O-16:2/2:0)	metab_14697	B(i)	95.2	0	-	M+H	C23H44NO7P	6.4536666666667	-	pos	478.2914741	0.037578209068912	57.8	-
pos_14700	4-Deoxyphysalolactone	metab_14699	B(ii)	0	55.9	HMDB0034346	M+H	C28H39ClO7	6.4536666666667	-	pos	523.2431751	0.056167879167367	44.1	78286-00-7
pos_14707	(9E,11Z)-8-Hydroxyoctadeca-9,11-Dienoic Acid	metab_14706	B(i)	54.4	0	-	M+H	C18H32O3	6.45895	-	pos	297.24153918871	0.010113619930311	49	-
pos_14738	Methyl Alpha-Eleostearate	metab_14737	B(i)	85.6	0	-	M+H	C19H32O2	6.4802833333333	-	pos	293.24674844919	0.096652783946661	55.4	-
pos_14758	Monoketocholic Acid	metab_14757	B(ii)	0	46.6	HMDB0254862	M+H	C24H38O6	6.4908666666667	-	pos	423.27053511599	0.077062256644303	45.3	-
pos_14782	Sphinganine 1-Phosphate	metab_14781	B(i)	83.7	0	HMDB0001383;PW_C001070	M+H	C18H40NO5P	6.5067333333333	C01120	pos	382.27068170341	0.14479272791202	54.4	19794-97-9
pos_14795	Pc(O-16:0)	metab_14794	B(i)	58.5	0	LMGP01020019	M+H	C24H50NO7P	6.5173666666667	-	pos	496.337988	0.017732134208348	50.4	0
pos_14801	Ethyl Linolenate	metab_14800	B(i)	80.5	0	HMDB0302158;HMDB0302813	M+H	C20H34O2	6.5227	-	pos	307.26225510294	0.040511672070612	54.6	-
pos_14809	Pc(18:1/0:0)	metab_14808	B(i)	77.3	0	LMGP01050029	M+H	C26H52NO7P	6.5280333333333	-	pos	522.35382133775	0.008844032336611	54.3	0
pos_14816	Austroinulin	metab_14815	B(ii)	0	65.1	HMDB0036802	M+H	C20H34O3	6.5333333333333	-	pos	323.25709892383	0.040083032698923	51.4	62868-75-1
pos_14820	Anisperimus	metab_14819	B(ii)	0	50.3	HMDB0248445	M+H	C18H39N7O3	6.5333333333333	-	pos	402.32019412407	0.077057384587155	47.2	-
pos_14836	Velvione	metab_14835	B(i)	60	0	-	M+NH4	C16H28O	6.5491833333333	-	pos	254.24714376703	0.038403281648667	50.6	-
pos_14847	Pe(O-15:1/3:0)	metab_14846	B(i)	85.8	0	-	M+H	C23H46NO7P	6.55445	-	pos	480.30729939001	0.006264807349765	55.6	-
pos_14851	Pe(P-16:0/2:0)	metab_14850	B(i)	90.2	0	-	M+Na	C23H46NO7P	6.5597166666667	-	pos	502.28891346253	0.032433953953258	55.8	-
pos_14855	S-Methoprene	metab_14854	B(i)	52	0	-	M+H	C19H34O3	6.565	-	pos	311.25718017201	0.024454735061787	48.8	-
pos_14890	Cp 47,497-C8-Homolog C-8-Hydroxy Metabolite	metab_14889	B(i)	38.3	0	-	M+H	C22H36O3	6.5863666666667	-	pos	349.26978746186	0.02238976215585	42.5	-
pos_14894	4Alpha-Carboxy-5Alpha-Cholesta-8,24-Dien-3Beta-Ol	metab_14893	B(ii)	0	44.7	HMDB0062385	M+K	C28H44O3	6.59175	_;C22112	pos	467.29620246929	0.082892599237265	43.9	-;_;
pos_14909	7Alpha-Hydroxycholesterol	metab_14908	B(ii)	0	43.9	HMDB0001496	M+K	C27H46O2	6.60775	C03594	pos	441.31685074321	0.037008306332759	43.8	566-26-7
pos_14910	Garcinoic Acid	metab_14909	B(i)	69.6	0	LMPR02020069	M+CH3OH+H	C27H38O4	6.60775	-	pos	459.30685432073	0.031590985845474	49.5	0
pos_14914	(Z)-9-Cycloheptadecen-1-One	metab_14913	B(ii)	0	60.2	HMDB0031336	M+NH4	C17H30O	6.6130833333333	-	pos	268.26273765574	0.088567488852318	49.1	542-46-1
pos_14918	Notoginsenoside T2	metab_14917	B(ii)	0	70.7	HMDB0039050	M+K	C37H62O10	6.6130833333333	-	pos	705.40079110329	0.12898818067644	49.9	343962-54-9
pos_14919	2-Hexylpentanedioic Acid	metab_14918	B(i)	94.5	0	-	M+H	C11H20O4	6.61835	-	pos	217.14283751159	0.060541001754815	56.5	-
pos_14921	Pinolenic Acid	metab_14920	B(i)	79.7	0	HMDB0256562;LMFA01030344	M+H	C18H30O2	6.61835	-	pos	279.23105510601	0.033701562509953	53.9	_;
pos_14922	Pinolenic Acid Methyl Ester	metab_14921	B(i)	44.3	0	-	M+CH3OH+H	C19H32O2	6.61835	-	pos	325.27277775204	0.013894780124084	47.6	-

pos_14935	3-Ethyl-4-(9-Hydroxy-4,6,8,10-Tetramethyl-7-Oxododec-4-En-2-Yl)Oxetan-2-One	metab_14934	B(ii)	0	46.1	HMDB0251673	M+H	C21H36O4	6.6289833333333	-	pos	353.26528285773	0.070874242432234	44.6	-
pos_14958	Methyl Linoleate	metab_14957	B(i)	79.7	0	HMDB0034381	M+H	C19H34O2	6.6449166666667	-	pos	295.26222917402	0.088089633965487	52.8	112-63-0
pos_14964	Palmitoyl Ethanolamide	metab_14963	B(i)	97.1	0	HMDB0002100	M+H	C18H37NO2	6.65025	C16512	pos	300.28884299603	0.1015401803593	57.4	544-31-0
pos_14974	Annonacin	metab_14973	B(ii)	0	63.8	HMDB0033327	M+Na-H2O	C35H64O7	6.6556	C20213;_	pos	601.44194000315	0.072253032987311	49	111035-65-5;_
pos_14975	Dg(11M3/9D3/0:0)	metab_14974	B(ii)	0	84.7	HMDB0116393	M+H	C40H66O7	6.6556	C00165;_	pos	659.48364167576	0.031438519416438	52.7	-
pos_14982	Hexadecanamide	metab_14981	B(i)	78.2	0	HMDB0012273;LMFA08010009	M+H	C16H33NO	6.66625	-	pos	256.26275292819	0.02276542897511	54.5	629-54-9;
pos_14986	N-Cyclopentyl-1-(6-Pyrrolidin-1-Ylsulfonylquinolin-2-Yl)Piperidine-3-Carboxamide	metab_14985	B(i)	56	0	-	M+H	C24H32N4O3S	6.66625	-	pos	457.23113762094	0.030291651786661	46.5	-
pos_15003	N-Stearoyl Valine	metab_15002	B(ii)	0	80.9	HMDB0241952	M+Na	C23H45NO3	6.68225	-	pos	406.32799068048	0.046496821162785	54.2	_;
pos_15004	Pe(O-12:0/6:0)	metab_15003	B(i)	89.9	0	-	M+H	C23H48NO7P	6.68225	-	pos	482.32323959138	0.092368209912805	54.8	-
pos_15008	(+/-)-(Z)-2-(5-Tetradecenyl)Cyclobutanone	metab_15007	B(ii)	0	56.7	HMDB0037543	M+H	C18H32O	6.68755	-	pos	265.2519027236	0.005356697832455	50.1	173074-85-6
pos_15010	Isolinderanolide	metab_15009	B(ii)	0	49.9	HMDB0038105	M+CH3OH+H	C21H36O3	6.68755	-	pos	369.29716012142	0.20318778252078	44.1	139559-06-1
pos_15011	Dodemorph	metab_15010	B(i)	62.5	0	-	2M+H	C18H35NO	6.68755	C18786	pos	563.54920271162	0.049416816955262	48.3	1593-77-7
pos_15013	Leupeptin	metab_15012	B(ii)	0	50.6	HMDB0254042	M+H-2H2O	C20H38N6O4	6.6928333333333	_;C01591	pos	391.28107140127	0.028861737267841	47.6	24365-47-7;_
pos_15019	Ruscogenin	metab_15018	B(ii)	0	41.1	HMDB0257364	M+CH3OH+H	C27H42O4	6.6981833333333	_;C08909	pos	463.33799370275	0.015768797991357	44.9	472-11-7;_;
pos_15025	4,4-Dimethyl-2-[3-Carboxylatopropyl]-2-Tridecyloxazolidine 3-	metab_15024	B(ii)	0	65.5	HMDB0246830	M+H-H2O	C22H43NO4	6.7035166666667	-	pos	368.31477896159	0.069165968103744	49.7	-
pos_15041	Dieporeticenin	metab_15040	B(ii)	0	70	HMDB0029792	M+CH3OH+Na	C37H64O4	6.7194	-	pos	627.49534778666	0.06279801375365	49.3	160544-67-2
pos_15047	N-Arachidonylethanolamine	metab_15046	B(ii)	0	52.4	HMDB0255084	M+H	C22H39NO	6.73	-	pos	334.30950792109	0.030624370309162	48.3	-
pos_15050	Docosanamide	metab_15049	B(ii)	0	51.7	HMDB0000583	M+H	C22H45NO	6.7406333333333	-	pos	340.35722023967	0.10327217258546	48.1	3061-75-4;
pos_15056	Dg(Pgd2/A-15:0/0:0)	metab_15055	B(ii)	0	74.9	HMDB0297532	M+H-2H2O	C38H66O8	6.7459333333333	-	pos	615.4574457	0.047753540777317	51.6	-
pos_15063	Tulathromycin A	metab_15062	B(ii)	0	80.6	HMDB0259324	M+ACN+Na	C41H79N3O12	6.7621833333333	C21788;_	pos	869.59015279208	0.071590808779254	48.6	217500-96-4;_
pos_15077	Bis(2-Ethylhexyl) Phthalate	metab_15076	B(i)	93.3	0	HMDB0249243	M+H	C24H38O4	6.7940333333333	C03690	pos	391.28311792843	0.028068483916508	56.2	-
pos_15085	His-Phe-Arg	metab_15084	B(i)	84	0	-	M+Na	C21H30N8O4	6.8046833333333	-	pos	481.22871640114	0.032746524652439	53.2	-
pos_15102	Paullinic Acid	metab_15101	B(ii)	0	61.7	HMDB0035159	M+H-H2O	C20H38O2	6.826	C21946;_	pos	293.28307199361	0.029813307770474	48.9	-;17735-94-3;
pos_15116	Octadecanamide	metab_15115	B(i)	59.8	0	HMDB0034146;LMFA08010003	M+H	C18H37NO	6.8313333333333	C13846	pos	284.29396411783	0.088627878080538	50.6	-;124-26-5;
pos_15125	Dgdg(18:3/18:3)	metab_15124	B(i)	86.7	0	-	M+Na	C51H84O15	6.8313333333333	-	pos	959.56699759268	0.13576013808464	53.8	-
pos_15127	Phytol	metab_15126	B(ii)	0	58.1	HMDB0002019	M+Na	C20H40O	6.8367	C01389	pos	319.29861975458	0.038677461424468	49	7541-49-3;150-86-
pos_15142	Neoannonin B	metab_15141	B(ii)	0	54	HMDB0037117	M+Na	C37H66O6	6.8473666666667	-	pos	629.47320250211	0.033499627898107	47.7	170312-94-4
pos_15173	Ivabradine	metab_15172	B(ii)	0	52.4	HMDB0253723	M+H	C27H36N2O5	6.9272166666667	-	pos	469.27306259936	0.005070447538266	44.4	-
pos_15175	Gilteritinib	metab_15174	B(ii)	0	47	HMDB0252717	M+H	C29H44N8O3	6.9272166666667	-	pos	553.36640254151	0.095337911379146	43.2	-
pos_15179	Dg(18:2/18:3)	metab_15178	B(i)	71.5	0	HMDB0007249	M+H	C39H66O5	6.93255	-	pos	615.49592464787	0.13390974255009	49.2	-
pos_15180	N,N-Diisopropylethylamine	metab_15179	B(i)	75.9	0	HMDB0247306	M+H	C8H19N	6.9379166666667	-	pos	130.15866955237	0.005509651645699	54	-
pos_15183	Dg(Pgf1Alpha/21:0/0:0)	metab_15182	B(ii)	0	52.8	HMDB0296632	M+Na	C44H82O8	6.9379166666667	-	pos	761.58775041019	0.07655513335246	46	-
pos_15185	Cyclohexane	metab_15184	B(ii)	0	47.9	HMDB0029597	M+NH4	C6H12	6.9432666666667	C11249	pos	102.1273267	0.005807418307291	47.3	110-82-7
pos_15193	Lubiminol	metab_15192	B(ii)	0	62.6	HMDB0029604	M+CH3OH+Na	C15H26O3	6.9805833333333	-	pos	309.20253428884	0.1302825469417	48.5	55784-92-4
pos_15207	Lefamulin	metab_15206	B(ii)	0	56.8	HMDB0254002	M+NH4	C28H45NO5S	7.0230666666667	-	pos	525.33542008264	0.14208924450457	46.4	-
pos_15210	(3Beta,5Alpha,9Alpha,22E,24R)-3,5,9-Trihydroxy-23-Methylergosta-7,22-Dien-6-One	metab_15209	B(ii)	0	40.1	HMDB0032669	M+K	C29H46O4	7.0283666666667	-	pos	497.30428204553	0.11538011120241	42.3	211486-12-3
pos_15212	Glabranin	metab_15211	B(ii)	0	40.9	HMDB0032104	M+H-H2O	C37H66O7	7.0283666666667	C09752	pos	605.47338900698	0.23729795266709	41.9	41983-91-9;_;
pos_15219	N-(2R-Hydroxyhexadecanoyl)-2S-Amino-9-Methyl-4E,8E-Octadecadiene-1,3R-Diol	metab_15218	B(ii)	0	72.5	HMDB0040132	M+H-H2O	C35H67NO4	7.04975	-	pos	548.50197539223	0.12528834916178	48.1	-
pos_15228	Dg(Pgf2Alpha/0:0/A-17:0)	metab_15227	B(ii)	0	41	HMDB0297750	M+H-2H2O	C40H72O8	7.0709833333333	-	pos	645.50398657168	0.079253727990994	43.1	-
pos_15231	Soblidotin	metab_15230	B(ii)	0	48.8	HMDB0258356	M+Na-H2O	C39H67N5O6	7.0763	-	pos	706.49358910234	0.062914367469239	44.9	-
pos_15236	Squalamine	metab_15235	B(ii)	0	73.5	HMDB0258449	M+CH3OH+Na	C34H65N3O5S	7.10315	C16841;_	pos	682.48444449286	0.045901145762837	45.9	148717-90-
pos_15240	Bis(2-Ethylhexyl) Sebacate	metab_15239	B(i)	83.5	0	-	M+H	C26H50O4	7.1243833333333	-	pos	427.37685845088	0.012884230325686	55.8	-
pos_15261	Triethanolamine	metab_15260	B(i)	94.6	0	HMDB0032538	M+H	C6H15NO3	7.24155	C06771	pos	150.11205057825	0.002220170548307	56.8	102-71-6

pos_15262	Soyacerebroside I	metab_15261	B(ii)	0	54.4	HMDB0032677	M+Na	C40H75NO9	7.2469	-	pos	736.53621308018	0.1042960389097	46.6	114297-20-0
pos_15266	Annohexocin	metab_15265	B(ii)	0	87.7	HMDB0041362	M+K	C35H64O9	7.2574833333333	-	pos	667.41343517425	0.22535413939694	54.2	167696-97-1
pos_15290	Cerebroside B	metab_15289	B(ii)	0	50.3	HMDB0035990	M+Na	C41H77NO9	7.3533833333333	-	pos	750.5556631	0.014622356428027	46.9	88642-46-0;
pos_15294	2,3-Bis(Palmitoyloxy)Propyl Dihydrogen Phosphate	metab_15293	B(ii)	0	45	HMDB0244852	M+K	C35H69O8P	7.36935	-	pos	687.44009763046	0.020933724557471	42.1	-
pos_15296	Tris(2,4-Di-Tert-Butylphenyl) Phosphite	metab_15295	B(i)	54.1	0	-	M+H	C42H63O3P	7.3747	-	pos	647.45662622992	0.020604478557452	49.1	-
pos_15306	Melamine	metab_15305	B(i)	56.6	0	HMDB0041922	M+H	C3H6N6	7.4012	C08737	pos	127.07235240712	0.025799342739791	49.7	108-78-1
pos_15315	Zeaxanthin	metab_15314	B(i)	89.1	0	HMDB0002789;LMPR01070261	M+H	C40H56O2	7.4224833333333	C06098	pos	569.43118235825	0.028015632240271	54.8	144-68-3;
pos_15324	Cis-Neoxanthin	metab_15323	B(ii)	0	50.1	HMDB0302969	M+H	C40H56O4	7.4280833333333	C13431	pos	601.41992248251	0.012770376116906	45.8	-
pos_15332	(Z)-2-Methyl-2-Butene-1,4-Diol 4-O-Beta-D-Glucopyranoside	metab_15331	B(ii)	0	75	HMDB0041187	2M+H	C11H20O7	7.4387333333333	-	pos	529.24981218672	0.015389300311412	53.1	-
pos_15434	Acetylphosphate	metab_15433	B(i)	38.1	0	HMDB0001494;PW_C001151	M+H	C2H5O5P	7.5184166666667	C00227	pos	140.99536971643	0.027195329870609	46.3	590-54-5
pos_15494	Tetradecyldiethanolamine	metab_15493	B(i)	85.5	0	-	M+H	C18H39NO2	7.5291	-	pos	302.30464772397	0.18342301444561	54	-
pos_15570	N-(2,6-Dimethylphenyl)-2-Hydroxyacetamide	metab_15569	B(i)	45.3	0	-	M+H	C10H13NO2	7.9671	-	pos	180.10161677066	0.27682879977877	42.7	-
pos_15748	7A,12A-Dihydroxy-3-Oxo-4-Cholenoic Acid	metab_15747	B(ii)	0	81.2	HMDB0000447	M+NH4	C24H36O5	7.5025	C15568	pos	422.29301313024	0.045961579065017	51.2	13587-11-6
pos_15759	Aldosterone 18-Glucuronide	metab_15758	B(ii)	0	43.3	HMDB0010345	M+Na-H2O	C27H36O11	7.4971833333333	C03033;_	pos	541.20553211286	0.10781953338749	43.6	3604-86-2;
pos_15764	Perphenazine Decanoate	metab_15763	B(ii)	0	57.5	HMDB0256350	M+ACN+Na	C31H44ClN3O2S	7.4918833333333	-	pos	621.29915648002	0.13000723712357	42.5	-
pos_15813	4-Hydroxyatorvastatin	metab_15812	B(ii)	0	76.5	HMDB0246450	M+Na	C33H35FN2O6	7.4387333333333	-	pos	597.23696931918	0.012871500434972	51.5	-
pos_15837	Calcimycin	metab_15836	B(i)	50.3	0	-;HMDB0249542	M+H	C29H37N3O6	7.4012	_;C11309	pos	524.27068319422	0.019319993866216	42.6	52665-69-7;_
pos_15863	Coronin	metab_15862	B(ii)	0	73.2	HMDB0303580	M+CH3OH+Na	C37H64O4	7.33735	-	pos	627.49529597342	0.12074853761769	51.6	-
pos_15873	As 1-1	metab_15872	B(ii)	0	57.1	HMDB0032794	M+Na	C38H71NO9	7.3054666666667	-	pos	708.50874423586	0.020795812777823	46.1	-
pos_15894	4,4'-Dioctyldiphenylamine	metab_15893	B(i)	48.3	0	-	M+H	C28H43N	7.1457	-	pos	394.34552060669	0.011736403727974	45	-
pos_15903	Dibutyl Sebacate	metab_15902	B(i)	86.2	0	HMDB0041220	M+H	C18H34O4	7.1243833333333	-	pos	315.25212411925	0.013740296514809	55.8	109-43-3
pos_15930	4-Deoxyannoreticuin	metab_15929	B(ii)	0	70.5	HMDB0033165	M+Na-H2O	C35H64O6	7.0337	-	pos	585.44716497701	0.026345971388745	50.6	206192-79-2
pos_15934	1-(8Z,11Z,14Z-Eicosatrienoyl)-Glycero-3-Phosphate	metab_15933	B(ii)	0	71.8	HMDB0062313	M+NH4	C23H41O7P	7.0283666666667	-	pos	478.29162650009	0.049761944050336	51.2	_;
pos_15938	24-Oxo-1Alpha,23,25-Trihydroxyvitamin D3	metab_15937	B(ii)	0	41.1	HMDB0060129	M+K	C27H42O5	7.0230666666667	-	pos	485.26245885539	0.049818737721483	42.6	-
pos_15944	Erucamide	metab_15943	B(i)	93	0	LMFA08010028	M+H	C22H43NO	7.0124666666667	-	pos	338.34076895953	0.014144976998247	57.2	0
pos_15952	2-[4-(Carboxymethyl)-1,4,8,11-Tetrazabicyclo[6.6.2]Hexadecan-11-Yl]Acetic Acid	metab_15951	B(ii)	0	40.5	HMDB0247867	M+CH3OH+H	C16H30N4O4	6.9859333333333	-	pos	375.26012231713	0.069322415354685	44.4	-
pos_15962	3-Methyl-1-Butylamine	metab_15961	B(ii)	0	41.4	HMDB0031659	M+H	C5H13N	6.9432666666667	C02640	pos	88.111819529712	0.016226715168442	46.5	107-85-7
pos_15972	Hydroxybuprenorphine	metab_15971	B(ii)	0	53.5	HMDB0060547	M+ACN+H	C29H41NO5	6.9272166666667	-	pos	525.33559181228	0.062072446521096	45.3	-
pos_15973	Psychotrin	metab_15972	B(i)	56.7	0	-	M+CH3OH+H	C28H36N2O4	6.9272166666667	-	pos	497.30441478326	0.096911170054841	45.5	-
pos_15978	2-Heptadecylfuran	metab_15977	B(ii)	0	63	HMDB0033608	M+NH4	C21H38O	6.9165666666667	-	pos	324.32521070499	0.067268140309352	50.2	208329-97-9
pos_16019	N-Isobutyloctadeca-Trans-2-Trans-4-Dienamide	metab_16018	B(ii)	0	60.1	HMDB0302880	M+H	C22H41NO	6.8367	-	pos	336.32515174569	0.006403671500186	50.4	-
pos_16027	7A,12A-Dihydroxy-Cholestene-3-One	metab_16026	B(ii)	0	46.1	HMDB0002197	M+CH3OH+H	C27H44O3	6.8313333333333	C05457	pos	449.35863644938	0.040200812384856	46.7	-
pos_16034	4-O-Methyl-12-O-Tetradecanoylphorbol 13-Acetate	metab_16033	B(ii)	0	40.5	HMDB0246554	M+CH3OH+Na	C37H58O8	6.826	-	pos	685.43321353779	0.024083731540621	45.6	-
pos_16045	N-Hexadecanoylpyrrolidine	metab_16044	B(ii)	0	49.1	HMDB0032740	M+H	C20H39NO	6.826	-	pos	310.30951618683	0.028642004932263	48.4	70974-48-0
pos_16050	Val Leu Ser Asp	metab_16049	B(i)	36.8	0	-	M+H	C18H32N4O8	6.8206666666667	-	pos	433.23131284198	0.023300708051497	44.2	-
pos_16052	Bis(2-Ethylhexyl)Adipate	metab_16051	B(i)	67.3	0	-	M+H	C22H42O4	6.8206666666667	-	pos	371.3144742	0.010990356427069	50.9	-
pos_16066	Beta-Funaltrexamine	metab_16065	B(ii)	0	73.8	HMDB0249128	M+H	C25H30N2O6	6.8046833333333	C18127;_	pos	455.21317116108	0.17343756788284	48.1	72782-05-9;_
pos_16079	Sm(10:1_2O/22:6)	metab_16078	B(i)	64	0	-	M+NH4	C37H63N2O6P	6.7781333333333	-	pos	680.47792510138	0.031558679490994	51.4	-
pos_16126	Ethyl Linoleate	metab_16125	B(i)	84.1	0	HMDB0252048	M+H	C20H36O2	6.7035166666667	-	pos	309.27786822119	0.055658419208748	54.7	-
pos_16129	Robustocin	metab_16128	B(ii)	0	80.4	HMDB0035898	M+Na	C35H62O5	6.6981833333333	-	pos	585.44721341034	0.034754308884293	53.4	-
pos_16132	Geranylgeraniol	metab_16131	B(i)	88.5	0	-;LMPR0104010009	M+NH4	C20H34O	6.6981833333333	C09094	pos	308.29383119585	0.019474655627382	53.4	24034-73-9;
pos_16141	Oleamide	metab_16140	B(i)	92.8	0	HMDB0002117;LMFA08010004	M+H	C18H35NO	6.68755	_;C19670	pos	282.27823740006	0.010913866955635	57.3	301-02-0;
pos_16142	Estrane	metab_16141	B(ii)	0	55.6	HMDB0251974	M+H	C18H30	6.68755	_;C19641	pos	247.24130649872	0.030537420772145	49.4	-;_
pos_16153	Phosphatidylcholine Lyso 18:0	metab_16152	B(i)	94.6	0	-	M+H	C26H54NO7P	6.6769166666667	-	pos	524.36943831207	0.018383935908336	56.8	-

pos_16159	Dg(Pge1/I-16:0/0:0)	metab_16158	B(ii)	0	61.3	HMDB0299325	M+Na	C39H70O8	6.66625	-	pos	689.49387653515	0.051388022312359	49.6	-
pos_16162	3,7-Dihydroxy-25-Methoxycucurbita-5,23-Dien-19-Al	metab_16161	B(ii)	0	40	HMDB0039362	M+K	C31H50O4	6.66625	-	pos	525.3358818	0.085864789431108	42.9	-
pos_16175	Oleoyl Ethanolamide	metab_16174	B(i)	94.6	0	HMDB0002088	M+H	C20H39NO2	6.66095	-	pos	326.30433905464	0.017750928454255	56.4	111-58-0
pos_16185	3,17,20-Trihydroxypregnane	metab_16184	B(ii)	0	47.5	HMDB0256756	M+NH4	C21H36O3	6.65025	-	pos	354.29923745975	0.091780155968778	46.5	-
pos_16186	Methyl Ricinoleate	metab_16185	B(i)	68.7	0	-	M+H	C19H36O3	6.65025	-	pos	313.27285314174	0.006402695896623	50.9	-
pos_16189	Dgdg(O-9:0/2:0)	metab_16188	B(i)	55.4	0	-	M+Na	C26H48O14	6.6449166666667	-	pos	607.28954530578	0.17807998590043	48.2	-
pos_16216	Hexylamine	metab_16215	B(ii)	0	61.9	HMDB0032323	M+H	C6H15N	6.6289833333333	C08306	pos	102.12732725183	0.012522923484259	50.2	111-26-2
pos_16219	3A,6B,7A,12A-Tetrahydroxy-5B-Cholanoic Acid	metab_16218	B(ii)	0	79.8	HMDB0000399	M+H-2H2O	C24H40O6	6.62365	C01094	pos	389.265215	0.03613374147253	52.2	80875-93-0
pos_16229	Ethyl 9,11,13-Octadecatrienoate	metab_16228	B(i)	88.5	0	-	M+H	C20H34O2	6.61835	-	pos	307.26231916438	0.014934874987846	56.1	-
pos_16242	Phoenicoxanthin	metab_16241	B(ii)	0	54.1	HMDB0248021	M+H	C40H52O3	6.6024166666667	C15967;_	pos	581.39779405007	0.017189286669522	47.1	-;_
pos_16250	(2-Acetyloxy-3-Hydroxypropyl) (E)-Octadec-9-Enoate	metab_16249	B(ii)	0	50.3	HMDB0242459	M+Na	C23H42O5	6.5970833333333	-	pos	421.29107484035	0.085169924290015	43.5	-
pos_16257	Deferoxamine	metab_16256	B(ii)	0	49	HMDB0014884	M+H-2H2O	C25H48N6O8	6.5863666666667	C06940	pos	525.33570752528	0.031510277653859	44.8	70-51-9;
pos_16266	Sclareol	metab_16265	B(ii)	0	48.8	HMDB0036827	M+H	C20H36O2	6.5810166666667	C09183	pos	309.27796512745	0.02426634657436	47.8	515-03-7;
pos_16267	3-Ketosphingosine	metab_16266	B(ii)	0	58.3	HMDB0245914	M+H-H2O	C18H35NO2	6.5810166666667	_;C06121	pos	280.26258318838	0.016610171757632	50.3	-;_;
pos_16268	Farnesyl Acetone	metab_16267	B(i)	84.2	0	-	M+H	C18H30O	6.5810166666667	-	pos	263.23620903107	0.016411437152436	55.5	-
pos_16275	Dg(9M5/9D3/0:0)	metab_16274	B(ii)	0	71.1	HMDB0116446	M+H	C40H66O7	6.5756833333333	C00165;_	pos	659.48398601479	0.038485610194698	50.8	-
pos_16290	Glyceryl Palmitate	metab_16289	B(i)	75.2	0	HMDB0011564	M+H	C19H38O4	6.565	-	pos	331.28327379353	0.099822082591941	51.3	32899-41-5
pos_16291	Methyl Linolenate	metab_16290	B(i)	75.9	0	HMDB0254600	M+H	C19H32O2	6.565	-	pos	293.24679353779	0.024272272579747	52.4	-
pos_16294	Cymegesolate	metab_16293	B(ii)	0	70.6	HMDB0250691	M+Na-H2O	C32H46O5	6.5597166666667	-	pos	515.31744848799	0.015298767994638	50.7	-
pos_16303	Pe(O-8:0/8:0)	metab_16302	B(i)	86.7	0	-	M+H	C21H44NO7P	6.5491833333333	-	pos	454.29135534359	0.013769811852203	56	-
pos_16304	Carboprost Methyl	metab_16303	B(ii)	0	50	HMDB0249645	M+CH3OH+Na	C22H38O5	6.5491833333333	-	pos	437.28475381953	0.088670193250246	45.7	-
pos_16307	Linoleoyl Ethanolamide	metab_16306	B(i)	85.9	0	HMDB0012252	M+H	C20H37NO2	6.5491833333333	-	pos	324.28873844444	0.0172317752662	55.5	68171-52-8
pos_16328	9,12-Octadecadiynoic Acid	metab_16327	B(i)	80.2	0	HMDB0247623;LMFA01030540	M+H	C18H28O2	6.5333333333333	-	pos	277.21543705606	0.016668017827129	53.7	_;
pos_16332	Nigericin	metab_16331	B(ii)	0	48.8	HMDB0255599	M+CH3OH+Na	C40H68O11	6.5280333333333	C11609;_	pos	779.49475940352	0.28272254728413	41.9	28380-24-7;_
pos_16349	Mg(Pgf1Alpha/0:0/0:0)	metab_16348	B(ii)	0	57.1	HMDB0260511	M+H-H2O	C23H42O7	6.5173666666667	-	pos	413.28566183968	0.021022543742563	47	-
pos_16355	Pe(Pgf2Alpha/P-18:1(9Z))	metab_16354	B(ii)	0	52.3	HMDB0285510	M+CH3OH+H	C43H78NO10P	6.5067333333333	-	pos	832.56447360078	0.1364654370657	45.2	-
pos_16367	3-Gonal	metab_16366	B(ii)	0	64.4	HMDB0245878	M+NH4	C18H28O	6.5014666666667	-	pos	278.24701537778	0.011901703300384	51	-
pos_16386	Methyl (10E,12Z)-9-Oxooctadeca-10,12-Dienoate	metab_16385	B(i)	59.7	0	-	M+H	C19H32O3	6.4908666666667	-	pos	309.24151326839	0.074556154168883	50.4	-
pos_16394	Darutoside	metab_16393	B(i)	52.5	0	-	M+CH3OH+Na	C26H44O8	6.4802833333333	-	pos	539.31746041826	0.022559711608219	49.2	-
pos_16415	Urea, N'-(2,4-Difluorophenyl)-N-((4-(2,2-Dimethylpropyl)Phenyl)Methyl)-N-Heptyl-	metab_16414	B(ii)	0	59	HMDB0250309	M+H	C26H36F2N2O	6.46965	-	pos	431.28702715748	0.16417987826374	48.6	-
pos_16418	Phytosphingosine-1-P	metab_16417	B(i)	96.4	0	HMDB0012280	M+H	C18H40NO6P	6.46965	-	pos	398.26542409069	0.053350759572008	54.7	-
pos_16423	Stearidonic Acid Ethyl Ester	metab_16422	B(i)	85.5	0	-	M+H	C20H32O2	6.4642833333333	-	pos	305.24657006767	0.12919723675924	54.3	-
pos_16444	P-Mentha-1,3,5,8-Tetraene	metab_16443	B(i)	61.8	0	HMDB0029641	M+H	C10H12	6.4536666666667	-	pos	133.10082400323	0.013073730452232	50.2	1195-32-0
pos_16448	Myristoleic Acid	metab_16447	B(ii)	0	75.9	HMDB0002000	M+Na	C14H26O2	6.4483333333333	C08322	pos	249.18420158286	0.092937546016552	51.2	544-64-9;
pos_16457	Lithocholyltaurine	metab_16456	B(ii)	0	53.4	HMDB0000722	M+ACN+H	C26H45NO5S	6.4377666666667	C02592	pos	525.33551823635	0.10340906376522	47.9	516-90-5
pos_16461	Car(14:3)	metab_16460	B(i)	44.4	0	-	M+H	C21H36NO4	6.4377666666667	-	pos	367.27098325772	0.026544419394562	44.3	-
pos_16463	(13R,14R)-8-Labdene-13,14,15-Triol	metab_16462	B(ii)	0	41.6	HMDB0034957	M+H	C20H36O3	6.4377666666667	-	pos	325.27274779538	0.029105361424533	46.2	-
pos_16465	Vernolic Acid	metab_16464	B(i)	74.7	0	-;HMDB0004702	M+H-H2O	C18H32O3	6.4377666666667	C14826;C08368	pos	279.23102740264	0.036793724714731	53.3	503-07-1;_
pos_16473	9,10,13-Trihome	metab_16472	B(ii)	0	61.9	HMDB0004710	M+CH3OH+Na	C18H34O5	6.4324833333333	_;C14835	pos	385.25465966424	0.041501714379462	49.9	-;29907-57-1
pos_16481	Rphdhd	metab_16480	B(ii)	0	59.2	HMDB0257327	M+H-H2O	C26H48O9	6.4272	-	pos	487.32271230389	0.077985995528305	46.5	-
pos_16495	Deoxyspergualin	metab_16494	B(ii)	0	44.8	HMDB0243514	M+H-2H2O	C17H37N7O3	6.41635	-	pos	352.28119947532	0.027208286493165	46.2	-
pos_16509	Dodecanamide	metab_16508	B(ii)	0	46.7	HMDB0251566	M+H	C12H25NO	6.4057	C13831	pos	200.20034596399	0.032009032933728	47.4	-;_;
pos_16515	7-Methyl-Cholic Acid	metab_16514	B(ii)	0	44.2	HMDB0244296	M+H	C25H42O5	6.4004166666667	-	pos	423.30679997362	0.07657840201228	44.8	-
pos_16516	Acetyltributyl Citrate	metab_16515	B(i)	88.6	0	HMDB0034159	M+H	C20H34O8	6.4004166666667	-	pos	403.2314453	0.026263172695588	56.3	77-90-7
pos_16522	Stearidonic Acid	metab_16521	B(i)	83.8	0	HMDB0006547;LMFA01030357;PW_C002804	M+H	C18H28O2	6.4004166666667	C16300	pos	277.21542117551	0.031478273372682	55.2	20290-75-9;

pos_16528	Pa(Pgj2/I-16:0)	metab_16527	B(ii)	0	50.5	HMDB0267760	M+H	C39H67O10P	6.3951166666667	-	pos	727.4580645	0.058111099530826	46.5	-
pos_16534	Pe(O-16:3/2:0)	metab_16533	B(i)	87.8	0	-	M+H	C23H42NO7P	6.3951166666667	-	pos	476.27580037953	0.023176422415357	56.1	-
pos_16537	Methyl 13-Hydroxyoctadeca-9,11-Dienoate	metab_16536	B(i)	45.1	0	-	M+H	C19H34O3	6.3951166666667	-	pos	311.25716619217	0.090487532247531	46.5	-
pos_16548	17-Acetoxygrindelic Acid	metab_16547	B(i)	61.9	0	-	M+H	C22H34O5	6.3898333333333	-	pos	379.24432656112	0.024039376738882	48.3	-
pos_16571	Riesling Acetal	metab_16570	B(ii)	0	41.2	HMDB0037562	M+CH3OH+Na	C13H22O3	6.3791666666667	-	pos	281.17155560814	0.05172603466644	46.6	-
pos_16580	Cer(14:3_2O/4:0)	metab_16579	B(i)	40.3	0	-	M+H	C18H31NO3	6.3738333333333	-	pos	310.23644029221	0.064884395611516	44.6	-
pos_16586	Milbemycin Alpha9	metab_16585	B(ii)	0	74.3	HMDB0254726	M+H	C36H47NO9	6.3685	-	pos	638.32778874809	0.064591459723771	50.2	-
pos_16617	(11R,16S)-Misoprostol	metab_16616	B(ii)	0	65.3	HMDB0242321	M+H	C22H38O5	6.3526333333333	-	pos	383.27596082168	0.026719273784868	49.6	-
pos_16630	(15A,20R)-Dihydroxypregn-4-En-3-One 20-[Glucosyl-(1->4)-6-Acetyl-Glucoside]	metab_16629	B(ii)	0	69.2	HMDB0033168	M+H	C35H54O14	6.3419833333333	-	pos	699.35396987021	0.014124796552079	50.5	-
pos_16637	11-Hydroxyeicosatetraenoate Glyceryl Ester	metab_16636	B(ii)	0	61.1	HMDB0012530	M+H	C23H38O5	6.3419833333333	-	pos	395.27603585109	0.073594201652239	47.8	-
pos_16650	Lovastatin Acid	metab_16649	B(ii)	0	57.3	HMDB0254177	M+H	C24H38O6	6.33135	_;C21130	pos	423.27063775609	0.018817644418433	47.8	75225-51-3;_
pos_16651	12(R)-Hpete	metab_16650	B(ii)	0	72.4	HMDB0004692	M+Na	C20H32O4	6.33135	C14812	pos	359.2159412	0.038510968157833	50.5	-;126873-49-2
pos_16671	Dihydrozeatin	metab_16670	B(ii)	0	54.1	HMDB0012215	M+NH4	C10H15N5O	6.3153833333333	_;C02029	pos	239.16094786754	0.074639509259935	48.1	23599-75-9;37789-32-5
pos_16688	Trans-Ekode-(E)-Ib	metab_16687	B(i)	65	0	-	M+H	C18H30O4	6.3047833333333	-	pos	311.2202469	0.046086207702883	50.4	-
pos_16690	Alpha-Linolenic Acid	metab_16689	B(i)	68.7	0	HMDB0001388;LMFA01030152;PW_C001073	M+H	C18H30O2	6.3047833333333	C06427	pos	279.23103460453	0.01472190201288	51.9	463-40-1;
pos_16692	Homononactic Acid	metab_16691	B(i)	75.5	0	-	M+H	C11H20O4	6.3047833333333	-	pos	217.14281872613	0.041697907413102	53.7	-
pos_16736	Spb(20:0_2O)	metab_16735	B(i)	81.5	0	-	M+H	C20H43NO2	6.2835166666667	-	pos	330.33564015736	0.16359348640469	54.7	-
pos_16742	N,N-Dimethyloctanamide	metab_16741	B(i)	40	0	-	M+H	C10H21NO	6.2835166666667	-	pos	172.16909155237	0.029068916384497	46.7	-
pos_16770	Prostaglandin E1	metab_16769	B(ii)	0	70.5	HMDB0001442	M+Na	C20H34O5	6.2674833333333	C04741;_	pos	377.228358	0.038933175884327	50.8	745-65-3;_
pos_16771	Spb(20:0_3O)	metab_16770	B(i)	59.3	0	-	M+H	C20H43NO3	6.2674833333333	-	pos	346.330742	0.11618636970284	50.1	-
pos_16772	Prostaglandin J2	metab_16771	B(i)	55.3	0	HMDB0002710;PW_C001687	M+H	C20H30O4	6.2674833333333	C05957	pos	335.2183757	0.002213695241771	47.7	60203-57-8
pos_16778	Neocnidilide	metab_16777	B(i)	70	0	HMDB0034450;HMDB0302242;HMDB0034450	M+H	C12H18O2	6.2674833333333	C17002	pos	195.13739782666	0.022377994094195	52.3	4567-33-3
pos_16804	Acoric Acid	metab_16803	B(i)	47.9	0	HMDB0038165	M+Na	C15H24O4	6.2567833333333	-	pos	291.15587085875	0.071740442284789	47.5	5956/6/9
pos_16816	9-Octadecen-1-Ol, (9Z)-	metab_16815	B(ii)	0	70.8	HMDB0247606	M+NH4	C18H36O	6.25145	-	pos	286.30957869334	0.088254996721626	52.2	-
pos_16822	3-Hydroxy-Hexadecanoic Acid	metab_16821	B(i)	79.8	0	LMFA01050188	M+H	C16H32O3	6.2461166666667	-	pos	273.24165918853	0.069358744859242	53.6	0
pos_16825	Cer(8:0_2O/14:0)	metab_16824	B(i)	82.3	0	-	M+H	C22H45NO3	6.2407666666667	-	pos	372.34594374585	0.021164849430772	54.2	-
pos_16834	Sphinganine	metab_16833	B(i)	99.5	0	HMDB0000269;LMSP01020001;PW_C000184	M+H	C18H39NO2	6.2354666666667	C00836	pos	302.30442698779	0.036102814574331	58.6	764-22-7;
pos_16841	Arachidoyl Ethanolamide	metab_16840	B(i)	61.6	0	HMDB0248559	M+H	C22H45NO2	6.2301166666667	-	pos	356.35112019248	0.055800793071793	49.9	-
pos_16842	3-Ketosphinganine	metab_16841	B(i)	90.1	0	HMDB0001480;LMSP01020002	M+H	C18H37NO2	6.2301166666667	C02934	pos	300.28885058642	0.036024624688436	56.8	16105-69-4;
pos_16849	Austinoneol	metab_16848	B(i)	83.5	0	-	M+H	C24H30O6	6.2247833333333	-	pos	415.210223	0.017629146148416	55.7	-
pos_16861	20-Hydroxy-6Z,15Z-Eicosadienoic Acid	metab_16860	B(ii)	0	84.4	HMDB0245564	M+NH4	C20H36O3	6.2195	-	pos	342.29910295371	0.076695462013014	54.1	_;
pos_16874	9,12,13-Trihydroxyoctadec-15-Enoic Acid	metab_16873	B(i)	43	0	-	M+H	C18H34O5	6.2142166666667	-	pos	331.24695520963	0.006823670225775	47.1	-
pos_16875	Phytosphingosine	metab_16874	B(i)	88.5	0	HMDB0004610;HMDB0004628;HMDB0004610;LMSP01030001	M+H	C18H39NO3	6.2142166666667	C12144	pos	318.29922375793	0.03924568211409	56.3	554-62-1;
pos_16876	(5Z,8Z)-16,17-Dihydroxyoctadeca-5,8-Dienoic Acid	metab_16875	B(i)	75.7	0	-	M+H	C18H32O4	6.2142166666667	-	pos	313.23638640314	0.020288082520519	53.4	-
pos_16883	N,N-Dibutylformamide	metab_16882	B(i)	47.4	0	-	M+H	C9H19NO	6.2142166666667	-	pos	158.15346621395	0.045172567760856	47.1	-
pos_16902	12-Hydroxy-13-(Hydroxymethyl)-3,5,7-Trimethyl-2,4-Tetradecadienedioic Acid	metab_16901	B(i)	44.2	0	-	M+H-2H2O	C18H30O6	6.2035833333333	-	pos	307.18712097887	0.06560381598491	45.4	-
pos_16913	Cervonoyl Ethanolamide	metab_16912	B(ii)	0	61.3	HMDB0013627	M+H	C24H36O3	6.1983	C13828	pos	373.27244588695	0.030037009515151	48.4	-
pos_16923	Alpha-Irone	metab_16922	B(ii)	0	58	HMDB0035631	M+H-H2O	C14H22O	6.1983	C09690	pos	189.16320471925	0.077990201647031	49.2	35124-13-1;79-69-6
pos_16937	Mg(15:0)	metab_16936	B(i)	43	0	HMDB0011532	M+H	C18H36O4	6.1876833333333	-	pos	317.26816866599	0.091597318131406	45.1	-
pos_16938	2-Aminooctadec-4-Yne-1,3-Diol	metab_16937	B(i)	95.8	0	-	M+H	C18H35NO2	6.1876833333333	-	pos	298.27317751072	0.046239997270664	57.8	-
pos_16947	Cer(8:0_2O/10:0)	metab_16946	B(i)	80.9	0	-	M+H	C18H37NO3	6.1823333333333	-	pos	316.28363290894	0.014986075244517	54.8	-
pos_16949	Spb(16:0_2O)	metab_16948	B(i)	90.3	0	-	M+H-H2O	C16H35NO2	6.1823333333333	-	pos	256.26273963433	0.024909790661602	56.5	-
pos_16963	Hexosylsphingosine	metab_16962	B(ii)	0	77.3	HMDB0253164	M+H	C24H47NO8	6.1717	-	pos	478.33601271659	0.003396820309969	53.7	-

pos_16971	(3E,11E)-2-Hydroxy-7-Methyl-6-Oxabicyclo[11.3.0]Hexadeca-3,11-Dien-5-One	metab_16970	B(i)	74.2	0	-	M+H	C16H24O3	6.1717	-	pos	265.17904935391	0.037759668307013	52.9	-
pos_16983	Blumealactone A	metab_16982	B(ii)	0	52.4	HMDB0036665	M+Na	C20H28O6	6.16635	-	pos	387.17912997094	0.052124486582347	49.3	111545-46-1
pos_16984	Polyoxyethylene 40 Monostearate	metab_16983	B(ii)	0	69.9	HMDB0032477	M+NH4	C20H40O3	6.16635	-	pos	346.33060658635	0.027451433764285	52.2	9004-99-3
pos_16991	P-Tolualdehyde	metab_16990	B(i)	88.9	0	HMDB0029638;HMDB0303310	M+H	C8H8O	6.16635	C06758;_	pos	121.06445119616	0.087941213610341	55.7	104-87-0;_
pos_17015	4-[(1E,3E)-Hepta-1,3-Dienyl]-3-(Hydroxymethyl)Cyclohexane-1,2-Diol	metab_17014	B(i)	68.9	0	-	M+H	C14H24O3	6.1556666666667	-	pos	241.17914849215	0.035089151720095	51.3	-
pos_17025	(9Z,12Z)-7,8,17-Trihydroxyoctadeca-9,12-Dienoic Acid	metab_17024	B(i)	58.1	0	-	M+H	C18H32O5	6.1504	-	pos	329.23127468621	0.057698366921057	48.7	-
pos_17058	Penbutolol	metab_17057	B(ii)	0	63.3	HMDB0015447	M+ACN+Na	C18H29NO2	6.1397666666667	C07416	pos	355.23427389715	0.026099932028598	48.3	38363-40-5;36507-48-9
pos_17060	13-Oxoode	metab_17059	B(i)	78.7	0	HMDB0004668;LMFA02000252	M+H	C18H30O3	6.1397666666667	_;C14765	pos	295.2258696	0.066561546429519	54.1	-;_;
pos_17061	Bolandiol	metab_17060	B(ii)	0	65.8	HMDB0249338	M+H	C18H28O2	6.1397666666667	-	pos	277.21539956602	0.064457551373287	51.5	-
pos_17064	13-Nor-6-Eremophilene-8,11-Dione	metab_17063	B(ii)	0	51.7	HMDB0037606	M+H	C14H20O2	6.1397666666667	-	pos	221.15299878014	0.040037058172374	48.5	348119-85-7
pos_17071	3,4-Dihydro-6,8-Dihydroxy-3-(10-Hydroxyundecyl)Isocoumarin	metab_17070	B(i)	96.4	0	-	M+H	C20H30O5	6.1344666666667	-	pos	351.2131682	0.027760248939666	55.9	-
pos_17072	(6E,8E)-5,10-Dioxooctadeca-6,8-Dienoic Acid	metab_17071	B(i)	65	0	-	M+H-H2O	C18H28O4	6.1344666666667	-	pos	291.19328215873	0.078054013351163	48.6	-
pos_17078	Palitantin	metab_17077	B(i)	71	0	-	M+H-H2O	C14H22O4	6.12915	-	pos	237.14783855999	0.079146986083018	51.5	-
pos_17080	Dodecylamine	metab_17079	B(i)	36.9	0	HMDB0251571	M+H	C12H27N	6.12915	-	pos	186.22107152461	0.028473675078507	45.3	-
pos_17083	N-Dodecylsarcosinate	metab_17082	B(ii)	0	76.1	HMDB0255129	M+CH3OH+H	C15H31NO2	6.1238666666667	-	pos	290.26812424274	0.016157796376864	53.8	-
pos_17086	Pterosin B	metab_17085	B(i)	66.3	0	HMDB0030759	M+H	C14H18O2	6.1238666666667	-	pos	219.13732734331	0.0886367389499	50.8	34175-96-7
pos_17091	Lauramine Oxide	metab_17090	B(i)	87.5	0	-	M+H	C14H31NO	6.1185833333333	-	pos	230.24718052614	0.034421871588008	56.4	-
pos_17097	10-Hexyl-11,12-Dioxatricyclo[7.2.1.01,6]Dodecane-2,3-Diol	metab_17096	B(i)	66.8	0	-	M+H	C16H28O4	6.1132833333333	-	pos	285.20516177506	0.019121638815877	51.4	-
pos_17098	3-(Dodecenyl)Dihydro-2,5-Furandione	metab_17097	B(i)	65.2	0	-	M+H	C16H26O3	6.1132833333333	-	pos	267.19472951363	0.029012639698596	51.5	-
pos_17101	Glu-Phe-Tyr	metab_17100	B(i)	70.2	0	-	M+H	C23H27N3O7	6.1079666666667	-	pos	458.19062785363	0.17497546781597	51.8	-
pos_17103	Lauryldiethanolamine	metab_17102	B(i)	96.9	0	-	M+H	C16H35NO2	6.1079666666667	-	pos	274.27318511319	0.011874239662023	58.1	-
pos_17104	Eucalyptol	metab_17103	B(i)	53.4	0	HMDB0004472	M+H	C10H18O	6.1079666666667	C09844	pos	155.14258300352	0.08735980854612	48	470-82-6
pos_17136	Tyr-Glu-Arg	metab_17135	B(i)	46.4	0	-	M+NH4	C20H30N6O7	6.0811833333333	-	pos	484.25402141316	0.10841610168045	45.8	-
pos_17186	Epinepetalactone	metab_17185	B(i)	61.3	0	HMDB0035063	M+H	C10H14O2	6.0652666666667	-	pos	167.10620259542	0.010465531063908	49.6	17257-15-7
pos_17191	Gibberellin A50	metab_17190	B(ii)	0	42.1	HMDB0035038	M+Na	C19H24O7	6.0599333333333	-	pos	387.14038753665	0.066479130107076	46.7	68062-25-9
pos_17205	(9S,10S)-10-Hydroxy-9-(Phosphonooxy)Octadecanoate	metab_17204	B(ii)	0	52.9	HMDB0059632	M+NH4	C18H37O7P	6.0546166666667	C15989	pos	414.26032764151	0.18218938880904	48	-;_
pos_17221	Cowagarcinone D	metab_17220	B(ii)	0	69.6	HMDB0304628	M+Na	C28H30O6	6.0493166666667	-	pos	485.19789124181	0.025364837751335	49.7	-
pos_17228	Gly Thr Lys	metab_17227	B(i)	46.2	0	-	M+H	C12H24N4O5	6.0493166666667	-	pos	305.18458746579	0.13297907035216	46.2	-
pos_17242	Rhodamine 6G	metab_17241	B(i)	88.8	0	-;HMDB0257199	M+H	C28H30N2O3	6.0440166666667	C11177;_	pos	443.22918295503	0.27828239067528	52.6	989-38-8;_
pos_17255	1-Deoxypebrolide	metab_17254	B(i)	36.6	0	-	M+NH4	C24H30O6	6.0386666666667	-	pos	432.24023967664	0.04187708967268	43.3	-
pos_17262	Acremin F	metab_17261	B(i)	68.6	0	-	M+H	C12H20O4	6.0386666666667	-	pos	229.1427741	0.050183902215957	50.4	-
pos_17272	His-Gly-Lys	metab_17271	B(i)	80.2	0	-	M+H-H2O	C14H24N6O4	6.0333333333333	-	pos	323.18201082288	0.008433263625509	54.6	-
pos_17283	Vapiprost	metab_17282	B(ii)	0	77.7	HMDB0259765	M+H-H2O	C30H39NO4	6.0280333333333	-	pos	460.2890462	0.056811448519692	50.6	-
pos_17284	Meteneprost	metab_17283	B(ii)	0	40.2	HMDB0254509	M+H-H2O	C23H38O4	6.0280333333333	-	pos	361.27313722629	0.017877282050501	45.5	-
pos_17285	Cyclo(Phe-Leu)	metab_17284	B(i)	40.8	0	HMDB0094673	M+H	C15H20N2O2	6.0280333333333	C20519	pos	261.15898508499	0.009375752358699	44.2	-
pos_17315	Medicocarpin	metab_17314	B(ii)	0	46.9	HMDB0033855	M+H	C22H24O9	6.0173666666667	C16223	pos	433.14570099912	0.04588177286777	46.1	52766-70-8
pos_17321	(3S,5S)-5-(Hydroxymethyl)-3-[(1R)-1-Hydroxy-6-Methylheptyl]Oxolan-2-One	metab_17320	B(i)	60.5	0	-	M+H-2H2O	C13H24O4	6.0173666666667	-	pos	209.15306119433	0.018257926774484	50.2	-
pos_17356	3-Benzyl-6-(1H-Indol-3-Ylmethyl)Piperazine-2,5-Dione	metab_17355	B(i)	71	0	-	M+H	C20H19N3O2	6.0013166666667	-	pos	334.15408242424	0.043487736837432	52.3	-
pos_17358	Xi-7-Hydroxyhexadecanedioic Acid	metab_17357	B(ii)	0	60.1	HMDB0037830	M+H-2H2O	C16H30O5	6.0013166666667	-	pos	267.19475677159	0.037857319980477	50.4	-
pos_17373	Fraxinellone	metab_17372	B(i)	63.1	0	-	M+H	C14H16O3	5.9959833333333	-	pos	233.11662775425	0.043770870465304	50.5	-
pos_17383	5,6-Dihydroxyprostaglandin F1A	metab_17382	B(ii)	0	46.9	HMDB0012109	M+H-H2O	C20H36O7	5.9906666666667	C06475	pos	371.23962902311	0.032589211581015	46.3	11000-26-3
pos_17385	Pro-Ala-Arg	metab_17384	B(i)	44.2	0	-	M+H	C14H26N6O4	5.9906666666667	-	pos	343.20837694863	0.027668827183793	47.2	-
pos_17400	Koninginin E	metab_17399	B(i)	64.7	0	-	M+H	C16H26O4	5.9853666666667	-	pos	283.18931594425	0.012603505896124	50.7	-

pos_17430	Ala Ser Ser Thr Lys	metab_17429	B(i)	56.4	0	-	M+H	C19H36N6O9	5.9747666666667	-	pos	493.26195961066	0.068929035458544	49.6	-
pos_17431	Isoflupredone Acetate	metab_17430	B(ii)	0	70.6	HMDB0253640	M+CH3OH+Na	C23H29FO6	5.9747666666667	_;C14636	pos	475.2061888	0.1311929304839	50.8	338-98-7;_
pos_17440	Ile Glu Phe	metab_17439	B(i)	66.6	0	-	M+H-H2O	C20H29N3O6	5.9747666666667	-	pos	390.20138813182	0.013721485151778	51.7	-
pos_17443	Isoamyl Cinnamate	metab_17442	B(ii)	0	59.4	HMDB0037704	M+NH4	C14H18O2	5.9747666666667	-	pos	236.16388095745	0.007487504301747	50.1	7779-65-9
pos_17467	Methylprednisolone Acetate	metab_17466	B(ii)	0	60.6	HMDB0254661	M+ACN+Na	C24H32O6	5.9641833333333	C08179;_	pos	480.23320305029	0.10076604574192	44	53-36-1;_
pos_17468	Gly-His-Lys	metab_17467	B(i)	62.9	0	-	M+H	C14H24N6O4	5.9641833333333	-	pos	341.19275182337	0.008765761662965	51.4	-
pos_17471	Octanoyl-L-Carnitine	metab_17470	B(i)	97	0	-	M+H	C15H29NO4	5.9641833333333	-	pos	288.21619959476	0.016862052397512	58.1	-
pos_17487	Gln-Ala-Lys	metab_17486	B(i)	59	0	-	M+H	C14H27N5O5	5.9588833333333	-	pos	346.21159751749	0.032649661979228	49.3	-
pos_17490	5,7-Dihydroxy-11-Ketotetranorprostanoic Acid	metab_17489	B(i)	73.6	0	-	M+H-2H2O	C16H28O5	5.9588833333333	-	pos	265.17919877126	0.01451758017911	52.9	-
pos_17492	Leu-Glu	metab_17491	B(i)	91.3	0	HMDB0028928	M+H-2H2O	C11H20N2O5	5.9588833333333	-	pos	225.12280462095	0.036530130575958	56.4	-
pos_17493	(5E,7E)-3-Aminoundeca-5,7-Dienoic Acid	metab_17492	B(i)	48.9	0	-	M+H	C11H19NO2	5.9588833333333	-	pos	198.1483496	0.029414956801132	47.2	-
pos_17495	Ps(Pgf2Alpha/18:3(9Z,12Z,15Z))	metab_17494	B(ii)	0	79.8	HMDB0281869	M+Na	C44H74NO13P	5.9535833333333	-	pos	878.48522078195	0.060733253787047	52.1	-
pos_17510	Phe Leu Phe	metab_17509	B(i)	37.9	0	-	M+H	C24H31N3O4	5.9535833333333	-	pos	426.23591757604	0.018476262641663	44.7	-
pos_17552	Leu Trp Phe	metab_17551	B(i)	55.5	0	-	M+H	C26H32N4O4	5.9429333333333	-	pos	465.24795883951	0.053322142758854	47.5	-
pos_17587	Anandamide	metab_17586	B(ii)	0	50.4	HMDB0004080	M+Na	C22H37NO2	5.9376333333333	C11695	pos	370.26897274711	0.002521494454989	46.8	94421-68-8
pos_17590	Isohomovanillic Acid	metab_17589	B(i)	61.3	0	HMDB0000333	M+H-H2O	C9H10O4	5.9376333333333	-	pos	165.0541767	0.20178242106469	50.5	1131-94-8
pos_17591	Acetophenone	metab_17590	B(i)	74.4	0	HMDB0033910	M+H	C8H8O	5.9376333333333	C07113	pos	121.06446421326	0.034635156392082	54	98-86-2
pos_17620	Almorexant	metab_17619	B(ii)	0	58.6	HMDB0248172	M+NH4	C29H31F3N2O	5.92705	-	pos	530.25763472602	0.040355725911778	45.8	-
pos_17730	Cichorioside J	metab_17729	B(ii)	0	59.3	HMDB0302056	M+H	C22H28O10	5.9056	-	pos	453.17186933766	0.022363280937065	49	-
pos_17736	7,8 Dihydrokawain	metab_17735	B(i)	49.9	0	-	M+H	C14H16O3	5.9056	-	pos	233.11656675667	0.14475403200556	48.2	-
pos_17743	Araloside A	metab_17742	B(ii)	0	46.9	HMDB0034535	M+ACN+Na	C47H74O18	5.9002666666667	C17540;_	pos	990.50381077601	0.059802691628921	48	7518-22-1
pos_17764	Propafenone Glucuronide	metab_17763	B(ii)	0	52	HMDB0247645	M+CH3OH+Na	C27H35NO9	5.9002666666667	-	pos	572.24854966392	0.11494730512584	47.9	-
pos_17778	2-Naphthylamine	metab_17777	B(i)	64.3	0	HMDB0041802	M+H	C10H9N	5.9002666666667	C02227	pos	144.08039646254	0.044234594691278	51.8	91-59-8
pos_17854	Cdp-Dg(5-Iso Pgf2Vi/I-20:0)	metab_17853	B(ii)	0	43.2	HMDB0294135	M+H-2H2O	C50H87N3O18 P2	5.8843833333333	-	pos	1044.5388223306	0.12716842926893	39.6	-
pos_17859	Capsianoside Vi	metab_17858	B(ii)	0	56.4	HMDB0039165	M+H-2H2O	C44H74O20	5.8843833333333	-	pos	887.46680053681	0.064727807461944	48.4	-
pos_17904	Atractysucrose Iiia	metab_17903	B(i)	68.6	0	-	M+K	C32H54O15	5.8790833333333	-	pos	717.30550650632	0.10924125781029	49.3	-
pos_17919	Trp-Gln-Leu	metab_17918	B(i)	55.4	0	-	M+H	C22H31N5O5	5.8790833333333	-	pos	446.23844871761	0.19542587764199	47.6	-
pos_17930	6'-Hmg Sdg	metab_17929	B(ii)	0	63.6	HMDB0040282	M+H	C38H54O20	5.8737833333333	-	pos	831.32605775908	0.064574368562315	50.6	-
pos_17935	Torvoside D	metab_17934	B(ii)	0	44.2	HMDB0029624	M+H	C38H62O13	5.8737833333333	-	pos	727.43264774729	0.02127982020541	45.5	-
pos_17942	Styraxlignolide F	metab_17941	B(i)	35	0	-	M+Na	C27H34O11	5.8737833333333	-	pos	557.19795258925	0.04395169472953	45.8	-
pos_17983	4-[(1R,2S)-3-(4-Benzylpiperidin-1-Yl)-1-Hydroxy-2-Methylpropyl]Phenol	metab_17982	B(ii)	0	49.8	HMDB0257258	M+CH3OH+Na	C22H29NO2	5.8685	-	pos	394.23263861041	0.02020898514216	47.1	-
pos_17985	Piperanine	metab_17984	B(ii)	0	46.3	HMDB0033874	M+NH4	C17H21NO3	5.8685	-	pos	305.18513309538	0.016491527698133	47.4	23512-46-1
pos_17987	4-Chlorobenzamide	metab_17986	B(i)	50.9	0	-	M+H	C7H6ClNO	5.8685	-	pos	156.02067254023	0.009703952756724	44	-
pos_18031	Nefazodone	metab_18030	B(ii)	0	50.2	HMDB0015280	M+ACN+Na	C25H32ClN5O	5.8578833333333	C07256	pos	533.23578606387	0.080326607341026	42	83366-66-9
pos_18038	Phe Pro Phe	metab_18037	B(i)	97.5	0	-	M+H	C23H27N3O4	5.8578833333333	-	pos	410.20661969128	0.013623834201482	57.2	-
pos_18042	Butyl 2-Amino-4-Methyl-Pentanoate	metab_18041	B(i)	76.2	0	-	M+H	C10H21NO2	5.8578833333333	-	pos	188.16404593446	0.065273123987825	53.5	-
pos_18049	Rifampicin	metab_18048	B(ii)	0	86.8	HMDB0257225	M+CH3OH+Na	C43H58N4O12	5.8525333333333	_;C06688	pos	877.41678417406	0.042001689741789	55.5	13292-46-1;_
pos_18069	Dide-O-Methyl-4-O-Alpha-D-Glucopyranosylsimmondsin	metab_18068	B(ii)	0	62.9	HMDB0038329	M+ACN+H	C20H31NO14	5.8525333333333	-	pos	551.20866706889	0.10551289210256	51.7	370068-00-1
pos_18075	Ile Ile Leu	metab_18074	B(i)	94.4	0	-	M+H	C18H35N3O4	5.8525333333333	-	pos	358.2691161	0.033336248446724	57.1	-
pos_18080	Diethylene Glycol Monobutyl Ether	metab_18079	B(i)	55.8	0	HMDB0244918	M+H	C8H18O3	5.8525333333333	-	pos	163.13244533261	0.034900018385553	48.9	-
pos_18139	Valganciclovir, (S)-	metab_18138	B(ii)	0	48.3	HMDB0259748	M+H	C14H22N6O5	5.8418333333333	-	pos	355.17211843004	0.017791943672888	48.8	-
pos_18186	Pa(Lte4/I-14:0)	metab_18185	B(ii)	0	44.1	HMDB0267554	M+Na-H2O	C40H70NO11P	5.8312333333333	-	pos	808.42577604094	0.035423368946938	45.2	-
pos_18212	Pro-Tyr-Lys	metab_18211	B(i)	37	0	-	M+H	C20H30N4O5	5.8312333333333	-	pos	407.22792646493	0.072765686632351	44.6	-
pos_18366	Tyr-Ala-Ser	metab_18365	B(i)	38.6	0	-	M+NH4	C15H21N3O6	5.8153	-	pos	357.17875782975	0.13173272191577	44.9	-

pos_18456	Pterosin G	metab_18455	B(i)	42	0	-	M+NH4	C14H18O3	5.8046833333333	-	pos	252.15882237169	0.012807776010637	46.9	-
pos_18481	Pro Phe Trp	metab_18480	B(i)	88.4	0	-	M+H	C25H28N4O4	5.7993833333333	-	pos	449.21726457239	0.04566131453634	55.5	-
pos_18483	Phe-Val-Leu	metab_18482	B(i)	93.5	0	-	M+H	C20H31N3O4	5.7993833333333	-	pos	378.2374018	0.054755204781844	56.4	-
pos_18502	Cdp-Dg(Pgj2/I-15:0)	metab_18501	B(ii)	0	66.4	HMDB0293603	M+Na-H2O	C47H77N3O17P2	5.7887333333333	-	pos	1022.4522607396	0.12468651047279	50	-
pos_18517	Trp-Phe-Tyr	metab_18516	B(i)	95.8	0	-	M+H	C29H30N4O5	5.7887333333333	-	pos	515.22713592449	0.005696434609891	56.9	-
pos_18550	Trp-Asp-Ile	metab_18549	B(i)	92.1	0	-	M+H	C21H28N4O6	5.7834166666667	-	pos	433.2072173	0.049841556512161	56.3	-
pos_18552	Ile-Ile-Ile	metab_18551	B(i)	90.7	0	-	M+H	C18H35N3O4	5.7834166666667	-	pos	358.26908454324	0.031119914357599	56.5	-
pos_18582	Asp Phe Phe	metab_18581	B(i)	73.2	0	-	M+H	C22H25N3O6	5.7781333333333	-	pos	428.18051032172	0.022535336412378	52.9	-
pos_18586	L-Beta-Aspartyl-L-Phenylalanine	metab_18585	B(ii)	0	53	HMDB0011167	M+H-H2O	C13H16N2O5	5.7781333333333	-	pos	263.10195617473	0.014926855953543	47.6	-
pos_18587	4-Hydroxyphenylpyruvic Acid	metab_18586	B(ii)	0	61.8	HMDB0000707	M+H-H2O	C9H8O4	5.7781333333333	C01179	pos	163.03854532569	0.14050893340882	51.1	156-39-8
pos_18601	Gln Trp Phe	metab_18600	B(i)	78.6	0	-	M+H	C25H29N5O5	5.7728166666667	-	pos	480.2229926	0.10023980309459	51	-
pos_18619	(+/-)-Win 55,212	metab_18618	B(i)	44.2	0	-	M+H	C27H26N2O3	5.7675166666667	-	pos	427.20662677393	0.023630618417362	42.9	-
pos_18638	4'-O-Methyl-(-)-Epicatechin 3'-O-Glucuronide	metab_18637	B(ii)	0	56.8	HMDB0041674	M+H	C22H24O12	5.7622166666667	-	pos	481.13179389793	0.11361071063486	48.5	-
pos_18734	Lys Ile Tyr	metab_18733	B(i)	52.2	0	-	M+H	C21H34N4O5	5.7353833333333	-	pos	423.26471628471	0.088888861143315	44.5	-
pos_18776	Agavoside A	metab_18775	B(ii)	0	54.4	HMDB0034391	M+Na	C33H52O9	5.7141333333333	C08885;_	pos	615.34822100953	0.035973729789451	48.5	56857-65-9;
pos_18785	Ps(5-Iso Pgf2Vi/22:6(4Z,7Z,10Z,13Z,16Z,19Z))	metab_18784	B(ii)	0	68.2	HMDB0283545	M+CH3OH+Na	C46H72NO13P	5.7088333333333	-	pos	932.48177382166	0.10131371319427	47.7	-
pos_18801	Val-Tyr-Lys	metab_18800	B(i)	39.9	0	-	M+H	C20H32N4O5	5.7035	-	pos	409.24094851061	0.012871395189015	44.6	-
pos_18832	11-Dodecenoic Acid	metab_18831	B(ii)	0	52.6	HMDB0032248	M+Na	C12H22O2	5.6928833333333	-	pos	221.15312027682	0.061686505706387	47.2	65423-25-8;
pos_18841	Val Leu Phe	metab_18840	B(i)	78.5	0	-	M+H	C20H31N3O4	5.68755	-	pos	378.23754590062	0.023030513265247	53.6	-
pos_18844	[3-(2-Aminopropyl)-6-Methylidenecyclohexa-1,3-Dien-1-Yl]Methanediol	metab_18843	B(ii)	0	49.5	HMDB0257571	M+ACN+Na	C11H17NO2	5.68755	-	pos	259.14348809277	0.055217978588637	47.4	-
pos_18857	Thr Phe Phe	metab_18856	B(i)	63.6	0	-	M+H	C22H27N3O5	5.6822166666667	-	pos	414.20114347753	0.037462301021603	49.5	-
pos_18868	Gly Phe Phe	metab_18867	B(i)	88.5	0	-	M+H	C20H23N3O4	5.67685	-	pos	370.17537589257	0.081526238817814	55.4	-
pos_18876	Ile His Asn Glu	metab_18875	B(i)	52	0	-	M+H	C21H33N7O8	5.67125	-	pos	512.24666012059	0.03363352822122	49.2	-
pos_18936	Narirutin	metab_18935	B(ii)	0	53.7	HMDB0033740	M+H-H2O	C27H32O14	5.6499666666667	C09793	pos	563.17201830893	0.038550287153891	46.8	14259-46-2;
pos_18951	Leu Tyr Ile	metab_18950	B(i)	70.3	0	-	M+H	C21H33N3O5	5.64465	-	pos	408.2473858	0.020032489522025	49.1	-
pos_18953	4-{Methyl[3-Phenyl-3-(Pyridin-2-Yl)Propyl]Amino}-4-Oxobutanoic Acid	metab_18952	B(i)	57.3	0	-	M+NH4	C19H22N2O3	5.64465	-	pos	344.19577826798	0.049814468636808	47.7	-
pos_18956	Mabioside E	metab_18955	B(ii)	0	42.8	HMDB0040654	M+H-2H2O	C48H78O22S	5.6392833333333	-	pos	1003.4662734603	0.072747166452907	44.1	156980-56-2
pos_18970	N-Lactoyl-Phenylalanine	metab_18969	B(i)	96	0	HMDB0062175	M+H	C12H15NO4	5.6392833333333	-	pos	238.10679061011	0.009066458079345	57.8	183241-73-8
pos_18989	Cis-3-Hexenyl Acetate	metab_18988	B(ii)	0	54.5	HMDB0040215	M+NH4	C8H14O2	5.634	C19757	pos	160.13277231578	0.014800510615042	49.6	3681-71-8
pos_19008	Moschamine	metab_19007	B(ii)	0	45.7	HMDB0032759	M+H-H2O	C20H20N2O4	5.6233833333333	-	pos	335.13805217541	0.028912569857711	47.4	68573-23-9
pos_19030	Plicatic Acid	metab_19029	B(ii)	0	40.9	HMDB0256643	M+H	C20H22O10	5.6180833333333	C10873;_	pos	423.12502935849	0.010574248769054	44.6	16462-65-0;_
pos_19032	N-[[1-(4-Carboxybutyl)-1H-Indazol-3-Yl]Carbonyl]-L-Valine,1-Methylester	metab_19031	B(i)	38.5	0	-	M+H	C19H25N3O5	5.6180833333333	-	pos	376.18591858607	0.084531700856117	45.2	-
pos_19034	Phenaceturic Acid	metab_19033	B(i)	36.8	0	HMDB0000821	M+ACN+H	C10H11NO3	5.6180833333333	C05598	pos	235.10707810499	0.039621161350842	45.7	500-98-1
pos_19036	10-Hydroxy-2E-Decenoic Acid	metab_19035	B(i)	82.8	0	LMFA01050157	M+H	C10H18O3	5.6180833333333	-	pos	187.13233518465	0.05342633179105	54.5	0
pos_19063	Glu Phe Phe	metab_19062	B(i)	82.1	0	-	M+H	C23H27N3O6	5.6074166666667	-	pos	442.1963434	0.01863942048519	53.7	-
pos_19066	Phe Ala Phe	metab_19065	B(i)	54.6	0	-	M+H	C21H25N3O4	5.6074166666667	-	pos	384.18897420729	0.010786917742125	47.7	-
pos_19098	Ala Leu Phe	metab_19097	B(i)	76.7	0	-	M+H	C18H27N3O4	5.5967333333333	-	pos	350.20639696074	0.050351082323576	52.7	-
pos_19156	Leu Pro Trp	metab_19155	B(i)	62.8	0	-	M+H	C22H30N4O4	5.5807333333333	-	pos	415.23285448112	0.045040036920767	50.4	-
pos_19160	Karanjachromene	metab_19159	B(i)	36.9	0	LMPK12111544	M+H-2H2O	C21H18O4	5.5807333333333	-	pos	299.1092712	0.10191470834576	42.6	0
pos_19166	Leukotriene C5	metab_19165	B(ii)	0	73.7	HMDB0012993	M+H-2H2O	C30H45N3O9S	5.5754333333333	-	pos	588.27331847656	0.015533183034843	49.8	75207-09-9
pos_19220	Ser-Phe-Ile	metab_19219	B(i)	52.1	0	-	M+H	C18H27N3O5	5.5595166666667	-	pos	366.20124976779	0.022247538474161	48.6	-
pos_19244	Elaterinide	metab_19243	B(ii)	0	45.9	HMDB0035893	M+H	C38H54O13	5.5488666666667	-	pos	719.36977095602	0.035582935479298	40.1	1398-78-3
pos_19254	2-[[3-Methyl-2-(Pyridine-3-Carbonylamino)Butanoyl]Amino]-3-Phenylpropanoic Acid	metab_19253	B(i)	61.2	0	-	M+H	C20H23N3O4	5.5488666666667	-	pos	370.17489886428	0.068813165582976	47.2	-

pos_19276	Allotetrahydrocortisol	metab_19275	B(i)	81.4	0	HMDB0000526	M+Na-H2O	C21H34O5	5.5435166666667	-	pos	371.2187397	0.018307292122584	52	302-91-0
pos_19339	Tyr Phe Glu Lys	metab_19338	B(i)	75.8	0	-	M+H	C29H39N5O8	5.5221833333333	-	pos	586.29295943074	0.12546469916966	47.1	-
pos_19342	Eleutherazineb	metab_19341	B(i)	92.8	0	-	M+H	C22H36N4O6	5.5221833333333	-	pos	453.26702326856	0.048914334412742	54.6	-
pos_19363	3-N-Methylspiperone	metab_19362	B(ii)	0	49.3	HMDB0245942	M+CH3OH+H	C24H28FN3O2	5.5115333333333	-	pos	442.25059625655	0.042976417677804	47	-
pos_19367	Caffeic Acid 3-Glucoside	metab_19366	B(ii)	0	46.7	HMDB0303040	M+H-H2O	C15H18O9	5.5115333333333	C10431	pos	325.09098486144	0.069361216946892	47.3	24959-81-7;_
pos_19379	Sisomicin Sulfate	metab_19378	B(ii)	0	79.8	HMDB0242632	M+H-H2O	C19H37N5O7	5.5060166666667	-	pos	430.26489739145	0.026273289717927	54.6	-
pos_19382	Beta,Beta-Dimethylacrylshikonin	metab_19381	B(i)	80.8	0	-;HMDB0245673	M+H	C21H22O6	5.5060166666667	C17415;_	pos	371.14566067742	0.035606519343912	49.9	24502-79-2;_
pos_19395	Gentamicin B	metab_19394	B(ii)	0	56.7	HMDB0252687	M+H-H2O	C19H38N4O10	5.5007166666667	-	pos	465.25653256048	0.17470632001218	47.3	-
pos_19398	Tert-Butyl (3S)-3-Hydroxypyrrolidine-1-Carboxylate	metab_19397	B(i)	36.9	0	-	M+ACN+H	C9H17NO3	5.5007166666667	-	pos	229.1540603	0.002017107570161	45.6	-
pos_19448	Cis-Mulberroside A	metab_19447	B(i)	82.9	0	HMDB0031726	M+Na	C26H32O14	5.4794166666667	-	pos	591.16679469598	0.056648728279434	54.1	166734-06-1
pos_19455	L-Acetylcarnitine	metab_19454	B(ii)	0	42.9	HMDB0000201	M+H	C9H17NO4	5.4794166666667	C02571;_	pos	204.1225177	0.031298541202815	46.9	3040-38-8
pos_19464	Arthrobactin	metab_19463	B(i)	42.3	0	-	M+H	C20H36N4O9	5.47405	-	pos	477.25357163747	0.012607952883696	46.5	-
pos_19466	Statine	metab_19465	B(ii)	0	49.7	HMDB0258487	M+H-H2O	C8H17NO3	5.47405	-	pos	158.11712903494	0.016411377305532	48.5	-
pos_19477	Withaferin A	metab_19476	B(ii)	0	44.9	HMDB0259897	M+K	C28H38O6	5.4687166666667	C08841;_	pos	509.22729520134	0.095485057438554	43.1	5119-48-2;_;
pos_19511	Tyr-Tyr-Lys	metab_19510	B(i)	61.2	0	-	M+H	C24H32N4O6	5.4581	-	pos	473.23581863788	0.056166052749317	48.5	-
pos_19550	Trp Leu	metab_19549	B(i)	96.5	0	-	M+H	C17H23N3O3	5.4474666666667	-	pos	318.1804195	0.027273564726373	57.9	-
pos_19566	Nefiracetam	metab_19565	B(ii)	0	52.4	HMDB0255503	M+CH3OH+Na	C14H18N2O2	5.4421666666667	-	pos	301.15388538467	0.008193552978334	48.4	-
pos_19573	Forsythoside H	metab_19572	B(i)	92.6	0	-	M+Na	C29H36O15	5.4367833333333	-	pos	647.19305878528	0.02593654815155	57.3	-
pos_19586	7-Hydroxycoumarine	metab_19585	B(i)	35.6	0	-	M+H	C9H6O3	5.4367833333333	-	pos	163.03855304078	0.015511091308838	45.8	-
pos_19593	22-Deoxoisocucurbitacin D	metab_19592	B(ii)	0	74.1	HMDB0034704	M+K	C30H46O6	5.43145	-	pos	541.2972093	0.019739615846167	50	15371-77-4
pos_19650	Hovenidulcioside B1	metab_19649	B(ii)	0	53	HMDB0041548	M+	C44H70O16	5.4100666666667	-	pos	854.47091032634	0.07411396754518	45.4	174902-16-0
pos_19672	Leu-Asp-Leu	metab_19671	B(i)	45.4	0	-	M+H	C16H29N3O6	5.4100666666667	-	pos	360.21220562006	0.031796395932767	46.9	-
pos_19686	Ile-Val-Leu	metab_19685	B(i)	58	0	-	M+H	C17H33N3O4	5.4047166666667	-	pos	344.25351655899	0.017286025356072	49.9	-
pos_19707	(3B,9R)-5-Megastigmene-3,9-Diol 9-[Apiosyl-(1->6)-Glucoside]	metab_19706	B(ii)	0	52	HMDB0038327	M+ACN+H	C24H42O11	5.3940666666667	-	pos	548.30672516514	0.037505191719646	48.9	347852-04-4;
pos_19728	N-Demethyl Mifepristone	metab_19727	B(ii)	0	40.4	HMDB0255109	M+CH3OH+H	C28H33NO2	5.3887333333333	-	pos	448.28860217108	0.039750654696916	40.9	-
pos_19730	2-O-Acetyl-Trans-Coutaric Acid	metab_19729	B(ii)	0	49.2	HMDB0034621	M+H-2H2O	C15H14O9	5.3887333333333	-	pos	303.04678755573	0.013170490843738	46.3	106928-35-2
pos_19751	Phenylalanyltryptophan	metab_19750	B(i)	94.3	0	HMDB0029006	M+H	C20H21N3O3	5.3834333333333	-	pos	352.16465856711	0.015042138051715	57.4	24587-41-5
pos_19763	Ile Phe Thr Asp Gln	metab_19762	B(i)	38.8	0	-	M+H	C28H42N6O10	5.3781166666667	-	pos	623.30184920535	0.057700785455245	45.8	-
pos_19789	Ile Ser Phe	metab_19788	B(i)	38.4	0	-	M+H	C18H27N3O5	5.3728166666667	-	pos	366.20099371932	0.012001585590643	46.2	-
pos_19791	5-(Furan-2-Carbonyl)-9-(Hydroxymethyl)-1,5-Diazacycloundecan-2-One	metab_19790	B(i)	50.9	0	-	M+H	C15H22N2O4	5.3728166666667	-	pos	295.16437825265	0.054232220156096	48.3	-
pos_19794	3-Ethyl-5-Hydroxy-4,5-Dimethyl-Pyrrolin-2-One	metab_19793	B(ii)	0	56.7	HMDB0253169	M+NH4	C8H13NO2	5.3728166666667	-	pos	173.12796405547	0.093486916751039	49.2	-
pos_19807	Ala-Phe-Ile	metab_19806	B(i)	72.6	0	-	M+H	C18H27N3O4	5.3674833333333	-	pos	350.20635423516	0.035801823606551	52.3	-
pos_19809	Methyl 2-Amino-3-Phenylpropanoate	metab_19808	B(i)	66.5	0	HMDB0245704	M+H-H2O	C10H13NO2	5.3674833333333	-	pos	162.09090348182	0.057230179536124	51.3	-
pos_19856	Ala Thr Tyr Lys	metab_19855	B(i)	43.2	0	-	M+H	C22H35N5O7	5.3515	-	pos	482.25593501947	0.071050187799056	43.8	-
pos_19876	Vertilmicin	metab_19875	B(ii)	0	42.6	HMDB0242753	M+H-H2O	C22H43N5O7	5.34615	-	pos	472.31167467757	0.032044593497585	46.2	-
pos_19881	Asn Ile Phe	metab_19880	B(i)	81.3	0	-	M+H	C19H28N4O5	5.34615	-	pos	393.21209622172	0.029609752197423	54.6	-
pos_19883	Gly-Gly-Leu	metab_19882	B(i)	49.7	0	-	M+H-H2O	C10H19N3O4	5.34615	-	pos	228.1337587	0.051941385127586	48.1	-
pos_19886	Bms-275183	metab_19885	B(ii)	0	57.9	HMDB0249316	M+ACN+H	C43H59NO16	5.3408166666667	-	pos	887.41215663193	0.17522794808068	47.4	-
pos_19948	Leu Ser Ile	metab_19947	B(i)	94.3	0	-	M+H	C15H29N3O5	5.32455	-	pos	332.21718822447	0.033433441963931	57.1	-
pos_20013	Ile-Gln-Leu	metab_20012	B(i)	96	0	-	M+H	C17H32N4O5	5.3032166666667	-	pos	373.24360041394	0.023349608278269	58	-
pos_20021	Sialorphin	metab_20020	B(ii)	0	59	HMDB0258277	M+H-H2O	C26H40N10O1	5.2979	-	pos	635.28373184346	0.18942306489196	45.5	-
pos_20025	Tyr-Lys-Ser	metab_20024	B(i)	66.5	0	-	M+H	C18H28N4O6	5.2979	-	pos	397.20472524197	0.017481772727223	49.7	-
pos_20060	Plantaricin Bn	metab_20059	B(ii)	0	45.1	HMDB0038238	M+H	C24H36O10	5.2819166666667	-	pos	485.23571658313	0.022303569925601	46.3	144377-77-5
pos_20065	Glu Pro Ile	metab_20064	B(i)	69.4	0	-	M+H	C16H27N3O6	5.2819166666667	-	pos	358.196306	0.020327954644042	49.5	-
pos_20066	Pro Phe	metab_20065	B(i)	49.8	0	-	M+H	C14H18N2O3	5.2819166666667	-	pos	263.1383727	0.043769230677506	47.8	-

pos_20080	25-Desacetyl Rifapentine	metab_20079	B(ii)	0	62.8	HMDB0245684	M+ACN+H	C45H62N4O11	5.2712166666667	-	pos	876.48000259631	0.025356019378486	47.5	-
pos_20118	Encaleret	metab_20117	B(ii)	0	43.7	HMDB0251787	M+H	C29H33ClFNO	5.2658833333333	-	pos	514.2170625	0.007830925248053	42.4	-
pos_20135	Phe Gly Phe Gly	metab_20134	B(i)	46.7	0	-	M+H	C22H26N4O5	5.2605666666667	-	pos	427.19647125579	0.012613064611028	47	-
pos_20137	Acetovanillone	metab_20136	B(i)	66.1	0	HMDB0247918	M+H	C9H10O3	5.2605666666667	C11380	pos	167.06983978309	0.10876170460075	51.3	498-02-2;_
pos_20151	Leu Ala Phe	metab_20150	B(i)	64.6	0	-	M+H	C18H27N3O4	5.2552	-	pos	350.20598171001	0.025715630663006	50.3	-
pos_20192	Ala Leu Leu	metab_20191	B(i)	73.2	0	-	M+H	C15H29N3O4	5.2392333333333	-	pos	316.22234909379	0.019213202765456	52.7	-
pos_20193	Neuromedin B (4-10)	metab_20192	B(ii)	0	41.7	HMDB0013017	M+ACN+Na	C40H53N11O8	5.2339	-	pos	911.40157150865	0.080104579861994	40.9	-
pos_20201	6-(4-O-Beta-D-Glucosyl-3-Methyl-Trans-But-2-Enyl-Amino)-	metab_20200	B(ii)	0	40	HMDB0302640	M+CH3OH+H	C16H23N5O6	5.2339	C03423	pos	414.19904281086	0.041146241814491	45.8	-
pos_20203	N-(5-(2,5-Dioxopyrrolidin-1-Yl)Pentyl)-N-Hydroxyacetamide	metab_20202	B(i)	62.3	0	-	M+CH3OH+H	C11H18N2O4	5.2339	-	pos	275.15952929516	0.049302792102045	50.7	-
pos_20236	Ser-Leu-Leu	metab_20235	B(i)	88.9	0	-	M+H	C15H29N3O5	5.22325	-	pos	332.21721121825	0.016993045237717	56.3	-
pos_20259	Hesperidin Methylchalcone	metab_20258	B(ii)	0	49.7	HMDB0253112	M+H	C29H36O15	5.2125833333333	-	pos	625.2108878	0.036069085719378	48.5	-
pos_20266	Umbelliferone 7-O-Rutinoside	metab_20265	B(i)	77.7	0	-	M+H	C21H26O12	5.2125833333333	-	pos	471.14832776248	0.016291273533332	54.4	-
pos_20273	2-Ethoxy-6(5-Tetrazolyl)Xanthone	metab_20272	B(ii)	0	46.2	HMDB0247563	M+H	C16H12N4O3	5.2125833333333	-	pos	309.09605206643	0.033816221255155	46.4	-
pos_20277	4-Hydroxycoumarin	metab_20276	B(i)	49.5	0	-	M+H	C9H6O3	5.2125833333333	C20414	pos	163.03849753672	0.007931669140477	49	1076-38-6
pos_20278	Phthalide	metab_20277	B(ii)	0	42.9	HMDB0032469	M+H	C8H6O2	5.2125833333333	_;C18611	pos	135.04370309963	0.053444284951902	46.5	27355-22-2;87-41-2
pos_20313	Gly-Ile-Ile	metab_20312	B(i)	64.4	0	-	M+H-H2O	C14H27N3O4	5.20195	-	pos	284.19615626067	0.029116827093924	50.4	-
pos_20319	Crenolanib	metab_20318	B(ii)	0	40.8	HMDB0250534	M+CH3OH+Na	C26H29N5O2	5.1965833333333	-	pos	498.24343529314	0.1520348576947	44.7	-
pos_20321	Pro Arg Tyr	metab_20320	B(i)	84.2	0	-	M+H	C20H30N6O5	5.1965833333333	-	pos	435.23343741901	0.02433806687157	54.3	-
pos_20325	Leu Asn Leu	metab_20324	B(i)	81.8	0	-	M+H	C16H30N4O5	5.1965833333333	-	pos	359.22792803885	0.014026387866376	54.8	-
pos_20373	Val Ile Leu	metab_20372	B(i)	47.4	0	-	M+H	C17H33N3O4	5.18055	-	pos	344.25356391245	0.005851869870757	47.5	-
pos_20375	6-(4-Methoxyphenyl)Pyrimidine-2,4-Diamine	metab_20374	B(i)	46.1	0	-	M+	C11H12N4O	5.18055	-	pos	216.10140221982	0.017930195741556	48.5	-
pos_20447	Leu Ala Ile	metab_20446	B(i)	92.8	0	-	M+H	C15H29N3O4	5.1592	-	pos	316.22231871813	0.01137430553962	56.9	-
pos_20449	Balagyptin	metab_20448	B(ii)	0	40.2	HMDB0041332	M+K	C39H64O16	5.1538333333333	-	pos	827.37595717508	0.28195940189893	43.3	172046-43-4
pos_20461	Ile-Thr-Ile	metab_20460	B(i)	89.4	0	-	M+H	C16H31N3O5	5.1538333333333	-	pos	346.23267476591	0.020194721287506	55.8	-
pos_20493	Taraxinic Acid Glucosyl Ester	metab_20492	B(ii)	0	51.7	HMDB0035249	M+CH3OH+H	C21H28O9	5.1431333333333	-	pos	457.20958693604	0.012828315650514	47.5	75911-14-7
pos_20500	2-(3-Methyloxan-4-Yl)Ethanol	metab_20499	B(i)	48.1	0	-	M+H-2H2O	C8H16O2	5.1431333333333	-	pos	109.10090449909	0.032332178767009	48.3	-
pos_20507	Leu-Pro-Leu	metab_20506	B(i)	91.6	0	-	M+H	C17H31N3O4	5.1378	-	pos	342.2377953	0.010935303587858	56.7	-
pos_20529	3-Aminonon-5-Enoic Acid	metab_20528	B(i)	61.2	0	-	M+H	C9H17NO2	5.13245	-	pos	172.13278362434	0.032400659031425	51	-
pos_20530	4-Oxo-2-Nonenal	metab_20529	B(i)	61.4	0	HMDB0060285;HMDB0245270	M+H	C9H14O2	5.13245	-	pos	155.10627418237	0.008424853478577	51	-
pos_20549	Mesotocin	metab_20548	B(ii)	0	92.8	HMDB0254480	M+H-H2O	C43H66N12O12S2	5.1218166666667	-	pos	989.44052679378	0.06374469417933	52.5	-
pos_20570	Digitoxigenin Bisdigitoxide	metab_20569	B(ii)	0	40.2	HMDB0251275	M+H	C35H54O10	5.1164666666667	-	pos	635.37342612904	0.030580873753588	43.5	-
pos_20578	Leu-Gly-Leu	metab_20577	B(i)	80	0	-	M+H	C14H27N3O4	5.1164666666667	-	pos	302.20670129386	0.007127836778532	54.2	-
pos_20628	3-N-Benzyl-3-Methyl-1-N-[2-(Trifluoromethyl)Phenyl]Piperidine-1,3-Dicarboxamide	metab_20627	B(i)	49.4	0	-	M+H	C22H24F3N3O2	5.10045	-	pos	420.19261154668	0.12784809205842	44.1	-
pos_20653	Asp-Leu-Leu	metab_20652	B(i)	80.9	0	-	M+H	C16H29N3O6	5.0951166666667	-	pos	360.21232917863	0.072785514492378	54.4	-
pos_20655	Delphinidin	metab_20654	B(ii)	0	57.1	HMDB0003074	M+NH4	C15H11O7+	5.0951166666667	C05908	pos	321.08153205887	0.032841343378036	46.6	528-53-0;13270-61-6;
pos_20715	Gln Leu Leu	metab_20714	B(i)	90	0	-	M+H	C17H32N4O5	5.0737166666667	-	pos	373.24366787512	0.01740337104816	56.2	-
pos_20731	Lys-Gly-Tyr	metab_20730	B(i)	53	0	-	M+H	C17H26N4O5	5.0683666666667	-	pos	367.19425639564	0.040578321860063	47.4	-
pos_20782	1-Ethyl 4-(2-Oxo-1,2-Diphenylethyl) Succinate	metab_20781	B(i)	50.4	0	-	M+H	C20H20O5	5.0525	-	pos	341.13758618756	0.086337644675887	48.2	-
pos_20792	Azelnidipine	metab_20791	B(i)	52.1	0	HMDB0248792	M+H	C33H34N4O6	5.04715	-	pos	583.2535673	0.044648716068616	45.9	-
pos_20796	17-O-Deacetylvindoline	metab_20795	B(ii)	0	42.1	HMDB0304032	M+ACN+Na	C23H31N2O5+	5.04715	_;C01091	pos	479.23610939626	0.064158446295725	37.9	-;_
pos_20838	Ile-Leu-Tyr	metab_20837	B(i)	82.8	0	-	M+H	C21H33N3O5	5.0364666666667	-	pos	408.2482241	0.079022355079761	53	-
pos_20859	(4E,7E,10E,13E)-Hexadeca-4,7,10,13-Tetraenoic Acid	metab_20858	B(ii)	0	50.3	HMDB0253125	M+Na	C16H24O2	5.0311166666667	-	pos	271.16465684327	0.10874780283749	47	-
pos_20862	Leucyl-Alanine	metab_20861	B(ii)	0	40.6	HMDB0304783	M+H-H2O	C9H18N2O3	5.0311166666667	-	pos	185.12800382971	0.031098256092343	46.2	-

pos_20875	Licoricesaponin G2	metab_20874	B(ii)	0	56.1	HMDB0039318	M+ACN+Na	C42H62O17	5.02045	-	pos	902.40905536782	0.030922082067615	47.4	-
pos_20916	Ser-Trp-Leu	metab_20915	B(i)	37.1	0	-	M+H	C20H28N4O5	5.0098333333333	-	pos	405.21326959064	0.004539786968107	45.8	-
pos_20929	2-(4-Methoxyphenyl)Ethyl 6-O-A-L-Arabinopyranosyl-B-D-Glucopyranoside	metab_20928	B(i)	35.4	0	-	M+Na	C20H30O11	5.0042166666667	-	pos	469.16681204132	0.069563974447541	43.9	-
pos_20930	Mitoxantrone	metab_20929	B(ii)	0	48	HMDB0015335	M+H	C22H28N4O6	5.0042166666667	C11195	pos	445.20458475907	0.002221856034973	46.8	65271-80-9
pos_20931	Phe-Glu-Lys	metab_20930	B(i)	73.7	0	-	M+H	C20H30N4O6	5.0042166666667	-	pos	423.22286697969	0.015297981671459	53.2	-
pos_20935	Petasinine	metab_20934	B(ii)	0	48.6	HMDB0030516	M+NH4	C13H21NO3	5.0042166666667	-	pos	257.18539942679	0.026838647426673	47.5	70474-33-8
pos_20975	Geranyl Arabinopyranosyl-Glucoside	metab_20974	B(ii)	0	41.1	HMDB0039573	M+H	C21H36O10	4.9882333333333	-	pos	449.23846403111	0.010603640514974	47	150995-11-2
pos_21033	Maytansine	metab_21032	B(i)	39.2	0	-;LMPK04000017	M+NH4	C34H46ClN3O10	4.9668666666667	C10606	pos	709.32690559884	0.058797657340042	39	35846-53-8;
pos_21063	Phe Thr Val	metab_21062	B(i)	79.4	0	-	M+H	C18H27N3O5	4.9615333333333	-	pos	366.20127922799	0.019983960473499	53.8	-
pos_21070	Erythromycin A Enol Ether	metab_21069	B(ii)	0	73.2	HMDB0251934	M+K	C37H65NO12	4.9561833333333	-	pos	754.40785565115	0.034493403372592	50.5	-
pos_21083	2-(1-(Cyclohexylmethyl)-1H-Indazole-3-Carboxamido)-3-Methylsuccinicacid	metab_21082	B(i)	52.3	0	-	M+H	C20H25N3O5	4.9561833333333	-	pos	388.18332849076	0.025092104414148	47.4	-
pos_21178	(E)-10-Hydroxy-8-Decenoic Acid	metab_21177	B(ii)	0	44.6	HMDB0039533	M+H	C10H18O3	4.9296333333333	-	pos	187.13233012566	0.23523311621844	46	106541-97-3
pos_21232	Pisumionoside	metab_21231	B(ii)	0	56.4	HMDB0039947	M+CH3OH+H	C19H32O9	4.9134166666667	-	pos	437.23780483309	0.08087451239456	48.9	371113-06-3
pos_21308	Ile-Pro-Ile	metab_21307	B(i)	94.1	0	-	M+H	C17H31N3O4	4.8920833333333	-	pos	342.23772179804	0.035561640414918	57.2	-
pos_21345	Imazapyr	metab_21344	B(i)	52.1	0	HMDB0253405	M+H	C13H15N3O3	4.8814333333333	C18864	pos	262.11796727019	0.041913209208688	48.2	81334-34-1;_
pos_21353	Dynorphin B (10-13)	metab_21352	B(ii)	0	55.6	HMDB0012936	M+CH3OH+Na	C20H39N5O6	4.8760833333333	-	pos	500.3068761	0.031983764101784	48.3	-
pos_21355	Phe Gln Val	metab_21354	B(i)	90.2	0	-	M+H	C19H28N4O5	4.8760833333333	-	pos	393.21213412825	0.063818999129679	55	-
pos_21357	Ethyl Butylacetylaminopropionate	metab_21356	B(ii)	0	43.6	HMDB0246904	M+ACN+H	C11H21NO3	4.8760833333333	C18830	pos	257.18545641923	0.088898449854077	47.6	52304-36-6;_
pos_21377	Phe Pro Val	metab_21376	B(i)	72.4	0	-	M+H	C19H27N3O4	4.8707833333333	-	pos	362.20676627798	0.040808761725576	52.4	-
pos_21417	Mmda	metab_21416	B(i)	52.3	0	HMDB0254817	M+H	C11H15NO3	4.86015	-	pos	210.11200720418	0.042692118389244	48.4	-
pos_21427	Phe-Val-Tyr	metab_21426	B(i)	83	0	-	M+H	C23H29N3O5	4.8548	-	pos	428.21708843043	0.062660190496177	53	-
pos_21446	Glu Asp Arg Arg	metab_21445	B(i)	39.3	0	-	M+H	C21H38N10O9	4.8494833333333	-	pos	575.2840471	0.023037665687536	44.4	-
pos_21502	Ile Thr	metab_21501	B(i)	42.8	0	-	M+H-2H2O	C10H20N2O4	4.8335833333333	-	pos	197.12799475937	0.013473784283482	47.3	-
pos_21505	Schidigerasaponin E1	metab_21504	B(ii)	0	61.7	HMDB0036297	M+CH3OH+H	C44H70O18	4.8282833333333	-	pos	919.48666958911	0.17700212130858	47.3	266998-23-6
pos_21543	Ile Tyr Val	metab_21542	B(i)	84.2	0	-	M+H	C20H31N3O5	4.8229833333333	-	pos	394.23257937285	0.0319585829992	55	-
pos_21546	Val-Val-Ile	metab_21545	B(i)	79.1	0	-	M+H	C16H31N3O4	4.8229833333333	-	pos	330.23799467886	0.009459818482366	54.2	-
pos_21565	1-(3,5-Dimethylbenzoyl)-N-(3-Methylsulfanylphenyl)Piperidine-3-Carboxamide	metab_21564	B(i)	43.2	0	-	M+H	C22H26N2O2S	4.8176333333333	-	pos	383.17968776985	0.049980487965919	43	-
pos_21594	Trp Leu Gly	metab_21593	B(i)	61.8	0	-	M+H	C19H26N4O4	4.8069333333333	-	pos	375.20197206459	0.01394601346489	49.7	-
pos_21643	Arg Asn Gln Glu	metab_21642	B(i)	41.8	0	-	M+H	C20H35N9O9	4.7909	-	pos	546.26567405416	0.014698076349114	46.5	-
pos_21677	Gly Val Phe	metab_21676	B(i)	74.2	0	-	M+H	C16H23N3O4	4.7855833333333	-	pos	322.17548574838	0.028707291444728	53.4	-
pos_21736	Glu Leu Gly Lys Gln	metab_21735	B(i)	36.4	0	-	M+H	C24H43N7O9	4.7643	-	pos	574.31777420847	0.1037842370206	44.2	-
pos_21759	Met-Pro-Leu	metab_21758	B(i)	81.8	0	-	M+H	C16H29N3O4S	4.759	-	pos	360.19389406492	0.073774842357963	54.1	-
pos_21760	Val Ser Phe	metab_21759	B(i)	73.6	0	-	M+H	C17H25N3O5	4.759	-	pos	352.18576981878	0.004436358512710	52.9	-
pos_21775	Physangulide	metab_21774	B(ii)	0	41.2	HMDB0037381	M+CH3OH+H	C28H42O9	4.74805	-	pos	555.31234454953	0.007277499068038	43.6	131749-58-1
pos_21790	Pa(6 Keto-Pgf1Alpha/22:4(7Z,10Z,13Z,16Z))	metab_21789	B(ii)	0	44.4	HMDB0265891	M+H	C45H75O12P	4.7427166666667	-	pos	839.50709633712	0.05253223300268	47	-
pos_21808	Tyr-Ser-Lys	metab_21807	B(i)	39.1	0	-	M+NH4	C18H28N4O6	4.7427166666667	-	pos	414.23360692241	0.029170684459836	44.2	-
pos_21834	1-(3-Trifluoromethylphenyl)Piperazine	metab_21833	B(i)	47.9	0	HMDB0244703	M+H	C11H13F3N2	4.7373666666667	-	pos	231.11223807626	0.014982958777815	47.6	-
pos_21853	Neolinustatin	metab_21852	B(ii)	0	49.5	HMDB0038482	2M+H	C17H29NO11	4.7266666666667	C08336	pos	847.35932462607	0.069859550450992	44.7	72229-42-6
pos_21876	3,5-Dimethyl-3'-Isopropyl-L-Thyronine	metab_21875	B(ii)	0	54	HMDB0246061	2M+H	C20H25NO4	4.7160166666667	-	pos	687.36578145461	0.1222861675424	48.8	-
pos_21912	Equilenin	metab_21911	B(ii)	0	41.6	HMDB0251896	M+CH3OH+Na	C18H18O2	4.7106666666667	C14303;_	pos	321.14378937105	0.051607556416246	44.2	517-09-9;_
pos_21944	Quercetin 7-Glucoside	metab_21943	B(ii)	0	54.9	HMDB0302151	M+ACN+Na	C21H20O12	4.6999833333333	C12639	pos	528.1100873	0.061380140860318	48.3	-
pos_21980	Cyanidin 3-O-(2-Xylosyl-6'-Glucosyl-Galactoside)	metab_21979	B(ii)	0	55.1	HMDB0301881	M+H	C32H39O20+	4.6894	-	pos	744.21498808708	0.036653554977902	48.4	-
pos_21985	Physcion 8-Gentiobioside	metab_21984	B(ii)	0	62.9	HMDB0036009	M+CH3OH+Na	C28H32O15	4.6894	C10382	pos	663.18788584255	0.17352667256507	49.7	84268-38-2;

pos_21994	Ser Ile Phe	metab_21993	B(i)	87.1	0	-	M+H	C18H27N3O5	4.6894	-	pos	366.20141981017	0.010414936123983	56	-
pos_21995	Trp-Met	metab_21994	B(i)	86.8	0	HMDB0029089	M+H	C16H21N3O3S	4.6894	-	pos	336.1369871	0.13014127307837	54.3	-
pos_21998	Aminocaproic Acid	metab_21997	B(ii)	0	51.5	HMDB0001901	M+ACN+H	C6H13NO2	4.6894	C02378	pos	173.12801750253	0.01305144378147	48.9	60-32-2
pos_22012	Arg Ile Phe	metab_22011	B(i)	95.9	0	-	M+H	C21H34N6O4	4.6840833333333	-	pos	435.27017101553	0.016421958285238	56.1	-
pos_22052	Ergosine	metab_22051	B(i)	37.3	0	-;HMDB0251913	M+H	C30H37N5O5	4.6681166666667	C09167;_	pos	548.29067703782	0.038990062255394	43.2	561-94-4;_
pos_22057	[2-(4-Propylphenyl)Quinolin-4-Yl]-(4-Pyridin-2-Ylpiperazin-1-Yl)Methanone	metab_22056	B(i)	41.9	0	-	M+H	C28H28N4O	4.6681166666667	-	pos	437.2351377	0.034157539316449	44.5	-
pos_22080	Pro-Asn-Ser	metab_22079	B(i)	51	0	-	M+H	C12H20N4O6	4.6628	-	pos	317.14645696913	0.002626033970369	48.9	-
pos_22090	N-Acetyl-Leukotriene E4	metab_22089	B(ii)	0	41.2	HMDB0005084	M+H	C25H39NO6S	4.6575	C11361	pos	482.25919462494	0.084422886884837	42.6	80115-95-3;
pos_22093	N-Cyclopropyl-4-[[1-(3,4-Dimethylbenzoyl)Piperidin-4-Yl]Methoxy]Benzamide	metab_22092	B(i)	39.5	0	-	M+H	C25H30N2O3	4.6575	-	pos	407.23759048634	0.0078228105942001	44.7	-
pos_22095	Met Phe	metab_22094	B(i)	78.3	0	-	M+H	C14H20N2O3S	4.6575	-	pos	297.1258969	0.016761339032386	52.8	-
pos_22110	Pro His Trp	metab_22109	B(i)	73.3	0	-	M+H	C22H26N6O4	4.6521666666667	-	pos	439.20779999969	0.0238683480507	52.6	-
pos_22123	Proctolin	metab_22122	B(ii)	0	61.8	HMDB0256789	M+Na-H2O	C30H48N8O8	4.6468333333333	-	pos	653.33934414583	0.03831150033055	50.7	-
pos_22127	Val His Phe Glu	metab_22126	B(i)	64.1	0	-	M+H	C25H34N6O7	4.6468333333333	-	pos	531.25648873017	0.05767323812994	46.5	-
pos_22133	[4-(1-Ethylimidazol-2-Yl)Piperazin-1-Yl]-(5-Methyl-1,2-Oxazol-3-Yl)Methanone	metab_22132	B(i)	46.9	0	-	M+H	C14H19N5O2	4.6468333333333	-	pos	290.15994527508	0.099797362091249	45.2	-
pos_22186	Thr-Tyr-Leu	metab_22185	B(i)	56.8	0	-	M+H	C19H29N3O6	4.6308166666667	-	pos	396.21211200987	0.039902326533668	49.1	-
pos_22187	N-[(4-Fluorophenyl)Methyl]-4-(6-Oxo-3-Pyrrolidin-1-Ylpyridazin-1-Yl)Benzamide	metab_22186	B(i)	57.2	0	-	M+H	C22H21FN4O2	4.6308166666667	-	pos	393.17354649588	0.011932915121621	49.1	-
pos_22190	Glu Leu Ala	metab_22189	B(i)	35.2	0	-	M+Na	C14H25N3O6	4.6308166666667	-	pos	354.16504991738	0.038833595016627	42.3	-
pos_22268	Astromicin	metab_22267	B(ii)	0	64.2	HMDB0248678	M+CH3OH+Na	C17H35N5O6	4.6095	C17708;_	pos	460.27555121661	0.021195520143733	51.5	55779-06-1;_
pos_22289	Bml-281	metab_22288	B(i)	37.5	0	-	M+H	C22H30N4O6	4.6041833333333	-	pos	447.22689749239	0.030032161563073	43.5	-
pos_22311	[(1R,5R)-5-(6-Aminopurin-9-Yl)Cyclohex-3-En-1-Yl]Methanol	metab_22310	B(ii)	0	47.1	HMDB0243753	M+ACN+Na	C12H15N5O	4.5989	-	pos	309.14388785313	0.035147138137844	48.5	-
pos_22317	Capsianoside I	metab_22316	B(ii)	0	58.5	HMDB0002318	M+H	C32H52O14	4.5936	-	pos	661.34943894257	0.074484695681033	46.3	121924-04-7
pos_22325	3,6,9,12,15,18,21,24,27,30-Decaoxadotriacontane-1,32-Diol	metab_22324	B(i)	42.3	0	-	M+H	C22H46O12	4.5936	-	pos	503.30507683505	0.018085078822401	46.3	-
pos_22337	Taxine	metab_22336	B(ii)	0	42.6	HMDB0258749	M+H-H2O	C35H47NO10	4.58825	-	pos	624.32216082114	0.20253259833507	44	-
pos_22356	17-Dimethylaminogeldanamycin	metab_22355	B(ii)	0	80.9	HMDB0244766	M+Na-H2O	C32H48N4O8	4.5829166666667	-	pos	621.32320433402	0.053184675080371	54.2	-
pos_22364	Val-Ser-Ile	metab_22363	B(i)	82.1	0	-	M+H	C14H27N3O5	4.5829166666667	-	pos	318.20170042587	0.026319581572303	53.8	-
pos_22369	Kaempferide 3-[Rhamnopyranosyl-(1->6)-Glucoside] 7-[illegible]	metab_22368	B(ii)	0	55.9	HMDB0040804	M+CH3OH+Na	C34H42O19	4.5776166666667	-	pos	809.2450917	0.26352409903611	48	150164-07-1
pos_22383	Ile-Val-Tyr	metab_22382	B(i)	56.8	0	-	M+H	C20H31N3O5	4.5776166666667	-	pos	394.23260103074	0.034072897102585	50	-
pos_22384	Leu-Ile-Asp	metab_22383	B(i)	54.4	0	-	M+H	C16H29N3O6	4.5776166666667	-	pos	360.21238862105	0.088299612264913	46.2	-
pos_22385	Ser Asn Thr	metab_22384	B(i)	36.4	0	-	M+H	C11H20N4O7	4.5776166666667	-	pos	321.14387296233	0.1186202486256	44.5	-
pos_22388	Ethyl Cinnamate	metab_22387	B(i)	37	0	-;HMDB0033834	M+H	C11H12O2	4.5776166666667	C06359;_	pos	177.09062286382	0.034840306548005	45.6	103-36-6;4192-77-2
pos_22403	Chavicol	metab_22402	B(i)	67.1	0	HMDB0034107	M+H	C9H10O	4.5723	C16930	pos	135.0801732	0.088161559365186	49.7	501-92-8
pos_22424	Ecgonine	metab_22423	B(ii)	0	47.5	HMDB0006548	M+CH3OH+H	C9H15NO3	4.56695	C10858	pos	218.13870943914	0.052639373026114	47.5	481-37-8
pos_22435	Gln Phe Tyr	metab_22434	B(i)	51.3	0	-	M+H	C23H28N4O6	4.5616	-	pos	457.20529703311	0.06625560568557	46.8	-
pos_22479	Phe-Pro	metab_22478	B(i)	91	0	HMDB0011177	M+H	C14H18N2O3	4.5456666666667	-	pos	263.13837556605	0.014100946242892	56.8	7669-65-0
pos_22483	Acetildenafil	metab_22482	B(ii)	0	46.1	HMDB0247908	M+CH3OH+Na	C25H34N6O3	4.5403666666667	-	pos	521.28022216177	0.10265950047771	45	-
pos_22489	1-(6-Hydroxy-2-Azabicyclo[2.2.1]Heptane-3-Carbonyl)Pyrrolidine-2-Carbonitrile	metab_22488	B(ii)	0	40.5	HMDB0260235	M+NH4	C12H17N3O2	4.5403666666667	-	pos	253.16527948836	0.029216436663155	45.4	-
pos_22501	Phe Gly Val	metab_22500	B(i)	35	0	-	M+H-H2O	C16H23N3O4	4.53505	-	pos	304.16498447601	0.053608294636284	45.2	-
pos_22515	Dinophysistoxin 1	metab_22514	B(ii)	0	41.4	HMDB0030442	M+H-2H2O	C45H70O13	4.52445	C16870;_	pos	783.46858716648	0.005131795658765	45.1	81720-10-7
pos_22521	Cyclo(Glycylleucylvalylleucylprolylseryl)	metab_22520	B(ii)	0	85.8	HMDB0303709	M+Na-H2O	C27H46N6O7	4.52445	-	pos	571.31823298204	0.023772664110735	54.5	-
pos_22531	Ingenol-5,20-Acetonide	metab_22530	B(i)	35.6	0	-	M+Na	C23H32O5	4.52445	-	pos	411.21255230486	0.094228986505458	42.2	-
pos_22536	Brivaracetam	metab_22535	B(ii)	0	42.4	HMDB0249388	M+H	C11H20N2O2	4.52445	-	pos	213.15927106531	0.045014724300087	46.8	-
pos_22550	Ps(5-Iso Pgf2Vi/16:1(9Z))	metab_22549	B(ii)	0	53.3	HMDB0281259	M+H	C40H70NO13P	4.5138166666667	-	pos	804.46725146344	0.051715524567632	47.1	-

pos_22596	Ala Glu Trp	metab_22595	B(i)	86.8	0	-	M+H	C19H24N4O6	4.5028833333333	-	pos	405.17781244093	0.032958004460053	54.6	-
pos_22601	Lys-Ile	metab_22600	B(i)	78	0	HMDB0028954	M+H	C12H25N3O3	4.5028833333333	-	pos	260.19623067025	0.029996496510309	53.3	20556-13-2
pos_22627	Pulegone	metab_22626	B(i)	85.7	0	HMDB0035602;HMDB0035604;HMDB0035604	M+H	C10H16O	4.4976	C09893	pos	153.12701189767	0.075629119321323	55.2	89-82-7
pos_22647	2-[[(2E)-2-Hydroxyimino-4-Methylpentanoyl]Amino]-4-Methylpentanoic Acid	metab_22646	B(i)	42.2	0	-	M+H	C12H22N2O4	4.4923	-	pos	259.16459302697	0.016314948279365	45.5	-
pos_22649	1-(4-Trifluoromethylphenyl)Piperazine	metab_22648	B(i)	48	0	-	M+H	C11H13F3N2	4.4923	-	pos	231.11224108874	0.006714860910909	47.5	-
pos_22665	Val-Asn-Leu	metab_22664	B(i)	92.3	0	-	M+H	C15H28N4O5	4.487	-	pos	345.21235332135	0.019513114665535	56.3	-
pos_22718	Nomilinic Acid	metab_22717	B(ii)	0	46.5	HMDB0035771	M+H	C28H36O10	4.4711166666667	-	pos	533.2339693	0.11243733597066	44	35930-20-2
pos_22743	Pc(Pgf2Alpha/2:0)	metab_22742	B(ii)	0	49.9	HMDB0288833	M+Na	C30H54NO11P	4.4605	-	pos	658.33873634504	0.034294050102787	44.9	-
pos_22754	Ile Thr Val	metab_22753	B(i)	77	0	-	M+H	C15H29N3O5	4.4605	-	pos	332.2172297	0.064097164741232	53.3	-
pos_22776	Gamma-Glutamyl-2-Aminobutyric Acid	metab_22775	B(ii)	0	55.3	HMDB0240645	M+H-H2O	C9H16N2O5	4.4498833333333	-	pos	215.1021232	0.012928973508908	49.8	16869-42-4
pos_22777	Allysine	metab_22776	B(ii)	0	60.8	HMDB0001263	M+ACN+H	C6H11NO3	4.4498833333333	C04076;C01475;_	pos	187.10724799134	0.048176472265919	50.7	-;6665-12-9;_;1962-83-0
pos_22788	(3S,6S)-3,6-Di-Sec-Butylpiperazine-2,5-Dione	metab_22787	B(i)	37	0	-	M+H	C12H22N2O2	4.4445333333333	-	pos	227.17487285736	0.006438336965679	46.3	-
pos_22802	Pro His Gln	metab_22801	B(i)	47	0	-	M+H	C16H24N6O5	4.4392333333333	-	pos	381.18721957168	0.01140583403237	47.7	-
pos_22819	Madlongiside D	metab_22818	B(ii)	0	68.9	HMDB0037905	M+K	C41H66O14	4.42865	-	pos	821.41308268621	0.097334908206876	48.6	58019-21-9
pos_22845	Gly Val Leu	metab_22844	B(i)	60.4	0	-	M+H	C13H25N3O4	4.4231	-	pos	288.19164591622	0.037857798159379	50.7	-
pos_22858	Micronomicin	metab_22857	B(ii)	0	45	HMDB0254707	M+ACN+Na	C20H41N5O7	4.4178	-	pos	527.31724102367	0.16399161554945	47	-
pos_22870	Galnon	metab_22869	B(ii)	0	51.4	HMDB0252602	M+H	C40H46N4O6	4.4125	-	pos	679.34890953503	0.17236746141299	46.6	-
pos_22904	Heliotron	metab_22903	B(ii)	0	44.7	HMDB0253072	M+ACN+H	C16H27NO5	4.4072	-	pos	355.22053318061	0.074099155945815	44.4	-
pos_22917	Gln Lys Leu Glu	metab_22916	B(i)	59.4	0	-	M+H	C22H40N6O8	4.4019	-	pos	517.29673001098	0.012591637127036	50.4	-
pos_22922	Arg Leu Phe	metab_22921	B(i)	80	0	-	M+H	C21H34N6O4	4.4019	-	pos	435.27054690863	0.031211365922831	54.1	-
pos_22999	Bis-(Acetyl)Diaminopentane	metab_22998	B(i)	47.7	0	-	M+H	C9H18N2O2	4.3806	-	pos	187.1436328	0.008107016421460	46.9	-
pos_23013	Ile Pro Val	metab_23012	B(i)	93.4	0	-	M+H	C16H29N3O4	4.3753	-	pos	328.22231639355	0.009851648688329	57.1	-
pos_23015	Gly Leu Val	metab_23014	B(i)	59.5	0	-	M+H	C13H25N3O4	4.3753	-	pos	288.19162809593	0.010655495870661	50.6	-
pos_23017	Lys-Leu	metab_23016	B(i)	88.9	0	HMDB0028955	M+H	C12H25N3O3	4.3753	-	pos	260.1962636	0.022182234011787	55.4	7369-79-1
pos_23045	Ethyl N,O-Bis(Tert-Butoxycarbonyl)Tyrosyl-L-	metab_23044	B(i)	50	0	-	M+H	C28H42N4O10	4.3646666666667	-	pos	595.2999375	0.038009745212872	45.3	-
pos_23098	Ser Pro Phe	metab_23097	B(i)	95.9	0	-	M+H	C17H23N3O5	4.3539833333333	-	pos	350.17030970197	0.010465695364031	57.9	-
pos_23101	(3Beta,6Beta)-Furanoeremophilane-3,6-Diol 6-Acetate	metab_23100	B(ii)	0	42.4	HMDB0034930	M+H-2H2O	C17H24O4	4.3539833333333	-	pos	257.15470532572	0.040213427967898	44.7	34335-95-0
pos_23114	4B-Hydroxystanozolol	metab_23113	B(ii)	0	44.9	HMDB0003318	M+CH3OH+Na	C21H32N2O2	4.34865	-	pos	399.2593499	0.015153627198826	46	125636-92-2
pos_23115	Asp Ile Val	metab_23114	B(i)	92.7	0	-	M+H	C15H27N3O6	4.34865	-	pos	346.19659810644	0.003325464189223	57.4	-
pos_23131	Ser-Leu-Tyr	metab_23130	B(i)	85.1	0	-	M+H	C18H27N3O6	4.3433333333333	-	pos	382.19644702539	0.083153017051545	53.7	-
pos_23133	2-Undecyl-Thiazolidine-4-Carboxylic Acid	metab_23132	B(i)	41.7	0	-	M+H	C15H29NO2S	4.3433333333333	-	pos	288.20197830171	0.013458465904362	43.5	-
pos_23136	Levodropropizine	metab_23135	B(i)	47.5	0	HMDB0251622	M+H-H2O	C13H20N2O2	4.3433333333333	-	pos	219.14867687505	0.019390927517902	48.1	-
pos_23152	L-Rhamnofuranose	metab_23151	B(ii)	0	73.2	HMDB0304405	M+H-2H2O	C6H12O5	4.3380333333333	C02431	pos	129.05432209624	0.15108462164573	52.9	-;_
pos_23178	Taxine B	metab_23177	B(ii)	0	50	HMDB0258750	M+Na-H2O	C33H45NO8	4.3273666666667	C19989	pos	588.29755896044	0.005482964662975	45.4	1361-51-9;_
pos_23227	3-Hydroxybutyric Acid	metab_23226	B(i)	89.4	0	HMDB0000011;PW_C000258	M+H-H2O	C4H8O3	4.3167	C01089	pos	87.043860515319	0.010128993143618	57	625-72-9;300-85-6
pos_23245	Phe Ala Gly	metab_23244	B(i)	52.9	0	-	M+CH3OH+Na	C14H19N3O4	4.3113833333333	-	pos	348.15457435863	0.031535767737801	48.6	-
pos_23251	Epsilon-Caprolactone	metab_23250	B(ii)	0	61.9	HMDB0060476	M+H	C6H10O2	4.3113833333333	C01880	pos	115.07509876546	0.026611443942283	51.3	502-44-3;_
pos_23282	Asparaginylarginine	metab_23281	B(ii)	0	47.8	HMDB0028725	M+Na	C10H20N6O4	4.3007833333333	-	pos	311.14192800991	0.052732305294819	45.8	2478/1/5
pos_23293	Leu-Pro-Tyr	metab_23292	B(i)	57.6	0	-	M+H	C20H29N3O5	4.2954833333333	-	pos	392.21702586657	0.16462650063567	47.7	-
pos_23296	Aucubin	metab_23295	B(ii)	0	61.1	HMDB0036562	M+H	C15H22O9	4.2954833333333	C09771	pos	347.13058398105	0.026135737188037	47.1	479-98-1;
pos_23305	Thr Val Phe Asp	metab_23304	B(i)	63.9	0	-	M+H	C22H32N4O8	4.2902	-	pos	481.22812282997	0.021878590776344	50.8	-
pos_23318	Trp-His-Arg	metab_23317	B(i)	42.6	0	-	M+Na-H2O	C23H31N9O4	4.28485	-	pos	502.23081417859	0.13690029657385	45.5	-
pos_23338	Phe-His-Ser	metab_23337	B(i)	71	0	-	M+H	C18H23N5O5	4.2795166666667	-	pos	390.1766397	0.03974636293811	50.8	-
pos_23349	Met Glu Leu Ser Glu	metab_23348	B(i)	35.3	0	-	M+H	C24H41N5O11	4.2741833333333	-	pos	608.26590343895	0.032814921815348	44.3	-

pos_23352	Glu Phe Thr Asp	metab_23351	B(i)	59.8	0	-	M+H	C22H30N4O10	4.2741833333333	-	pos	511.20235948048	0.022288182397638	49.3	-
pos_23357	Glu Leu Lys	metab_23356	B(i)	47.7	0	-	M+H	C17H32N4O6	4.2741833333333	-	pos	389.23847967175	0.0291001656238	48.1	-
pos_23362	Ile-Ser-Ile	metab_23361	B(i)	54.1	0	-	M+H	C15H29N3O5	4.2741833333333	-	pos	332.21726229641	0.012604644879803	48.9	-
pos_23368	[1-(Tert-Butylcarbamoyl)Piperidin-4-Yl] Acetate	metab_23367	B(i)	43.2	0	-	M+H	C12H22N2O3	4.2741833333333	-	pos	243.16973846914	0.056137779	47.4	-
pos_23371	Ttatpn	metab_23370	B(ii)	0	76.9	HMDB0259311	M+Na	C43H46N8O6	4.2688833333333	-	pos	793.3455397	0.015041081503902	51.5	-
pos_23394	Coagulin R 3-Glucoside	metab_23393	B(ii)	0	54.8	HMDB0033207	M+H-2H2O	C34H48O11	4.26355	-	pos	597.3120146	0.097684312779015	43.5	221695-66-5
pos_23398	Thr Phe Arg	metab_23397	B(i)	39	0	-	M+H	C19H30N6O5	4.26355	-	pos	423.2381935	0.057286735059905	42.8	-
pos_23412	Thr Pro Phe	metab_23411	B(i)	36.9	0	-	M+H	C18H25N3O5	4.2582	-	pos	364.18575339978	0.011322662483693	45.9	-
pos_23452	Gln-Val-Leu	metab_23451	B(i)	94.8	0	-	M+H	C16H30N4O5	4.2472833333333	-	pos	359.22806136513	0.004441847839302	57.6	-
pos_23631	Cucurbitacin I	metab_23630	B(ii)	0	60.2	HMDB0250582	M+K	C30H42O7	4.2046833333333	_;C08800	pos	553.25295447064	0.097983442873341	48.6	2222/7/3;_;
pos_23661	Trp-Ser-Gln	metab_23660	B(i)	36.3	0	-	M+H	C19H25N5O6	4.1940166666667	-	pos	420.18699502199	0.020986018824638	44.5	-
pos_23675	Xi-Linalool 3-[Rhamnosyl-(1->6)-Glucoside]	metab_23674	B(ii)	0	41	HMDB0030422	M+CH3OH+H	C22H38O10	4.1887	-	pos	495.27983140884	0.048960091040804	46.3	84534-33-8
pos_23686	Met-Leu	metab_23685	B(i)	68.4	0	HMDB0028977	M+H	C11H22N2O3S	4.1887	-	pos	263.14030952056	0.017445396469686	50.6	-
pos_23714	Ile-Thr-Tyr	metab_23713	B(i)	96.4	0	-	M+H	C19H29N3O6	4.1780666666667	-	pos	396.2125456	0.030079481899801	58.2	-
pos_23716	Ser Leu Val	metab_23715	B(i)	92.5	0	-	M+H	C14H27N3O5	4.1780666666667	-	pos	318.20157533286	0.002438050819541	56.7	-
pos_23741	Caffeine	metab_23740	B(i)	99	0	HMDB0001847;PW_C001222	M+H	C8H10N4O2	4.1727666666667	C07481	pos	195.08719715281	0.01773523972378	57.2	1958/8/2;58-08-2
pos_23742	Diglycidyl Ether	metab_23741	B(i)	38.5	0	HMDB0251282	M+NH4	C6H10O3	4.1727666666667	-	pos	148.09650142249	0.069291923011562	45.8	-
pos_23749	Val Leu Thr Gly Ser	metab_23748	B(i)	41.8	0	-	M+H	C20H37N5O8	4.1674166666667	-	pos	476.27044616908	0.015191674900029	42.2	-
pos_23755	Astragaloside Iii	metab_23754	B(ii)	0	79	HMDB0248674	M+K	C41H68O14	4.1620833333333	C08924;_	pos	823.41781787021	0.096539483046205	50.6	84687-42-3;_
pos_23767	Gpcho(7:0/2:0)	metab_23766	B(i)	37.6	0	-	M+H	C17H34NO8P	4.1620833333333	-	pos	412.21244925241	0.035661971042658	43.8	-
pos_23783	Ergocrystine	metab_23782	B(i)	41.9	0	-	M+H	C35H39N5O5	4.15675	-	pos	610.30684346762	0.026416769472349	44.7	-
pos_23823	Lymecycline	metab_23822	B(ii)	0	69.2	HMDB0014401	M+ACN+H	C29H38N4O10	4.1460833333333	-	pos	644.29780859167	0.061244975876681	50.3	992-21-2
pos_23834	Methyl Helianthenoate F Glucoside	metab_23833	B(ii)	0	47.6	HMDB0040797	M+H	C17H22O8	4.1460833333333	-	pos	355.13686021386	0.02094337177361	46	-
pos_23848	Galanganone A	metab_23847	B(i)	40.8	0	-	M+H	C32H36O6	4.1407333333333	-	pos	517.26160759143	0.070187059014817	43.8	-
pos_23870	Chebi:69439	metab_23869	B(i)	77.4	0	-	M+H	C10H16N2O2	4.1354333333333	-	pos	197.12798879592	0.09413423359842	52.7	-
pos_23906	Ile Gly Val	metab_23905	B(i)	42.4	0	-	M+H	C13H25N3O4	4.1248166666667	-	pos	288.19177063303	0.01819940409721	47.7	-
pos_23947	Asn Ile Val	metab_23946	B(i)	45.2	0	-	M+H	C15H28N4O5	4.1141666666667	-	pos	345.21274262886	0.092417576529193	46	-
pos_23949	Pro Gly Phe	metab_23948	B(i)	70.7	0	-	M+H	C16H21N3O4	4.1141666666667	-	pos	320.16054180006	0.046910787667949	52.4	-
pos_23961	Ile Asp Ala Ser Lys	metab_23960	B(i)	35.5	0	-	M+H	C22H40N6O9	4.1088166666667	-	pos	533.29221055508	0.046663828276201	45.3	-
pos_23967	Sophoraflavanone G	metab_23966	B(ii)	0	42.6	HMDB0258381	M+H	C25H28O6	4.1088166666667	C18053;_	pos	425.19421982957	0.053033460268993	44.3	-;_;
pos_24056	Altiloxin B	metab_24055	B(i)	38.5	0	-	M+CH3OH+H	C15H23ClO4	4.0875333333333	-	pos	335.16281338863	0.10737308071289	40.3	-
pos_24065	Glycocholenate Sulfate	metab_24064	B(ii)	0	78	HMDB0242178	M+H-H2O	C26H41NO9S	4.0822166666667	-	pos	526.24777866392	0.12272674950124	52.2	-
pos_24085	Fluocortolone Pivalate	metab_24084	B(ii)	0	48.7	HMDB0252353	M+CH3OH+Na	C27H37FO5	4.0768833333333	-	pos	515.28091832377	0.049990537065882	46.6	-
pos_24095	Leu Leu Ala	metab_24094	B(i)	49.9	0	-	M+H	C15H29N3O4	4.0768833333333	-	pos	316.22261048275	0.034049804183033	48.2	-
pos_24099	Tyr-Val	metab_24098	B(i)	78.5	0	HMDB0029118	M+H	C14H20N2O4	4.0768833333333	-	pos	281.14900381133	0.13295139996872	53.7	-
pos_24105	Vinflunine	metab_24104	B(ii)	0	53.3	HMDB0259815	M+CH3OH+Na	C45H54F2N4O	4.0715833333333	-	pos	871.41280495712	0.080070585660521	43.3	-
pos_24110	Val Asp Gly Thr Ile	metab_24109	B(i)	47.3	0	-	M+H	C21H37N5O9	4.0715833333333	-	pos	504.26508334405	0.07460916174608	47	-
pos_24140	4-Megastigmene-6A,9R-Diol 9-[Apiosyl-(1->6)-Glucoside]	metab_24139	B(ii)	0	45.8	HMDB0041580	M+CH3OH+Na	C24H42O11	4.061	-	pos	561.28656588865	0.04095406698552	46	177261-74-4
pos_24143	Pro-Ile-His	metab_24142	B(i)	51.8	0	-	M+ACN+H	C17H27N5O4	4.061	-	pos	407.2382064	0.054453165749111	48	-
pos_24151	3-(N-Maleimidopropionyl)Biocytin	metab_24150	B(ii)	0	43.1	HMDB0245790	M+H	C23H33N5O7S	4.0557	-	pos	524.21598844808	0.031212061875286	45.6	-
pos_24185	7,8-Dihydrobuddlenol B	metab_24184	B(i)	46.4	0	-	M+H	C31H38O11	4.0397333333333	-	pos	587.24478507891	0.051461248893157	42.2	-
pos_24186	Canescein	metab_24185	B(ii)	0	46.4	HMDB0029313	M+H-H2O	C29H42O11	4.0397333333333	-	pos	549.26893671959	0.012289400789124	48	22333-73-9
pos_24193	Pro Ile Arg	metab_24192	B(i)	94.2	0	-	M+H	C17H32N6O4	4.0397333333333	-	pos	385.2550501	0.017721022695656	57.8	-
pos_24196	Ile-Gly-Tyr	metab_24195	B(i)	64.3	0	-	M+H	C17H25N3O5	4.0397333333333	-	pos	352.18536875078	0.016386765043636	50.3	-
pos_24198	Leu Ile Val	metab_24197	B(i)	51.5	0	-	M+H-H2O	C17H33N3O4	4.0397333333333	-	pos	326.24297188597	0.043252767098668	47.6	-

pos_24208	Gpcho(2:0/7:0)	metab_24207	B(i)	37.5	0	-	M+H	C17H34NO8P	4.0344	-	pos	412.21248866842	0.024049432436934	44.1	-
pos_24223	Anamorelin	metab_24222	B(ii)	0	45	HMDB0248383	M+Na-H2O	C31H42N6O3	4.02905	-	pos	551.31594511718	0.1052490591992	42.7	-
pos_24229	His Ala Val Asp	metab_24228	B(i)	40.4	0	-	M+H	C18H28N6O7	4.02905	-	pos	441.20794655746	0.045030877486972	46.2	-
pos_24250	2-Hydroxy-Imipramine Glucuronide	metab_24249	B(ii)	0	46.2	HMDB0060717	M+Na	C25H32N2O7	4.02375	-	pos	495.20742145269	0.050814712082684	46.2	-
pos_24256	Pro Ala Leu	metab_24255	B(i)	45.4	0	-	M+H	C14H25N3O4	4.02375	-	pos	300.19106434996	0.01198259947794	47.1	-
pos_24296	Glu-Val-Lys	metab_24295	B(i)	63.2	0	-	M+H	C16H30N4O6	4.01315	-	pos	375.22235169305	0.018374722728075	50.2	-
pos_24308	Remikiren	metab_24307	B(ii)	0	47.4	HMDB0014357	M+Na-H2O	C33H50N4O6S	4.0078666666667	C07465	pos	635.32274369912	0.009924961018641	47.6	126222-34-2
pos_24335	Ile Asp Trp	metab_24334	B(i)	38.8	0	-	M+NH4	C21H28N4O6	4.0024666666667	-	pos	450.23306614558	0.03851335301884	41.1	-
pos_24410	N-Eicosapentaenoyl Glutamine	metab_24409	B(ii)	0	64.1	HMDB0242069	M+ACN+Na	C25H38N2O4	3.9812166666667	-	pos	494.29621665258	0.037588842576174	49.4	-
pos_24418	Met Pro Val	metab_24417	B(i)	83	0	-	M+H	C15H27N3O4S	3.9812166666667	-	pos	346.178631	0.018568776291072	54.5	-
pos_24429	[D-Met2, Pro5]-Enkephalinamide	metab_24428	B(ii)	0	42.2	HMDB0247158	M+ACN+Na	C30H40N6O6S	3.9758833333333	-	pos	676.28307585079	0.057654846596074	40.3	-
pos_24433	Argatroban	metab_24432	B(ii)	0	51.9	HMDB0014423	M+ACN+Na	C23H36N6O5S	3.9758833333333	C04931	pos	572.26572273182	0.048509541963339	46	74863-84-6
pos_24440	Peg N7	metab_24439	B(i)	77.9	0	-	M+H	C14H30O8	3.9758833333333	-	pos	327.20057441755	0.041592882601052	54	-
pos_24459	Leu Ile Ser Glu	metab_24458	B(i)	65.1	0	-	M+H	C20H36N4O8	3.9705333333333	-	pos	461.2582104	0.045621375150773	49.2	-
pos_24474	Hordatine B	metab_24473	B(ii)	0	59.6	HMDB0030459	M+ACN+Na	C29H40N8O5	3.9652	C08308	pos	644.32342484132	0.017152338979231	46.8	10502-21-3
pos_24534	Tyrosyl-Leucine	metab_24533	B(ii)	0	68.2	HMDB0029109	M+H	C15H22N2O4	3.9545	-	pos	295.16457989432	0.014816450402804	52.3	-
pos_24592	Val-Asp-Lys	metab_24591	B(i)	66	0	-	M+H	C15H28N4O6	3.9438	-	pos	361.20745076093	0.00695692573511	51.8	-
pos_24616	Gly Ala Phe	metab_24615	B(i)	68.9	0	-	M+H	C14H19N3O4	3.9384166666667	-	pos	294.14427016736	0.040201343872559	51.4	-
pos_24617	2-Methylbutyroylcarnitine	metab_24616	B(i)	86.1	0	HMDB0000378;LMFA07070034	M+H	C12H23NO4	3.9384166666667	-	pos	246.1694335	0.055902048817766	54.6	31023-25-3;
pos_24628	Phe-Glu-Gln	metab_24627	B(i)	63.7	0	-	M+H	C19H26N4O7	3.9330666666667	-	pos	423.18613043517	0.028000510035969	50.2	-
pos_24631	Tyr Gly Tyr	metab_24630	B(i)	53	0	-	M+H	C20H23N3O6	3.9330666666667	-	pos	402.1650365	0.001226736252166	49.5	-
pos_24639	Cyclo(Ile-Ala)	metab_24638	B(i)	52.2	0	-	M+CH3OH+H	C9H16N2O2	3.9330666666667	-	pos	217.15418298421	0.046472897775944	48.8	-
pos_24640	1-(2-(Pyridin-3-Yl)Pyrrolidin-1-Yl)Ethanone	metab_24639	B(i)	40.6	0	-	M+H	C11H14N2O	3.9330666666667	-	pos	191.11741804966	0.008609028416070	45.4	-
pos_24644	Murabutide	metab_24643	B(ii)	0	57.9	HMDB0254948	M+Na-H2O	C23H40N4O11	3.9277166666667	-	pos	553.24977658001	0.12016658356608	46.1	-
pos_24687	Phe Lys	metab_24686	B(i)	44.5	0	-	M+H	C15H23N3O3	3.9170833333333	-	pos	294.18013292116	0.011909964376887	45.5	-
pos_24724	Ziritaxestat	metab_24723	B(ii)	0	53	HMDB0252755	M+ACN+H	C30H33FN8O2	3.9011333333333	-	pos	630.28171207546	0.091644908490295	45.6	-
pos_24737	Thr-Glu-Leu	metab_24736	B(i)	91.6	0	-	M+H	C15H27N3O7	3.9011333333333	-	pos	362.19149387985	0.012088656947912	57.1	-
pos_24784	Ser Pro Leu	metab_24783	B(i)	85.8	0	-	M+H	C14H25N3O5	3.8905	-	pos	316.18587465229	0.017347032899233	55.6	-
pos_24789	Bellericagenin B	metab_24788	B(i)	51.7	0	-	M+K	C30H48O7	3.8851666666667	-	pos	559.30293313245	0.12267144754127	46.3	-
pos_24835	Leukotriene F4	metab_24834	B(ii)	0	48.1	HMDB0006465	M+H-H2O	C28H44N2O8S	3.8746166666667	C06462;_	pos	551.27967939931	0.075486585530571	44.6	-;_;
pos_24843	Pro Gln Leu	metab_24842	B(i)	54.9	0	-	M+H	C16H28N4O5	3.8746166666667	-	pos	357.21237187429	0.018950796102818	48.9	-
pos_24896	Ly293111	metab_24895	B(i)	46.4	0	-	M+H	C33H33FO6	3.8586	-	pos	545.23182423544	0.065824522792946	46.4	-
pos_24949	Val-Arg-Ile	metab_24948	B(i)	92.2	0	-	M+H	C17H34N6O4	3.8480166666667	-	pos	387.27071247456	0.041449725796666	55.4	-
pos_24993	Asi-222	metab_24992	B(ii)	0	54.4	HMDB0260432	M+NH4	C29H45NO7	3.83745	-	pos	537.34931525995	0.038335095858917	47.1	-
pos_24995	Ala Ala Gln Asn Leu	metab_24994	B(i)	44.1	0	-	M+H	C21H37N7O8	3.83745	-	pos	516.27676365731	0.041412235217935	46.7	-
pos_25001	Thr-Arg-Ile	metab_25000	B(i)	92.4	0	-	M+H	C16H32N6O5	3.83745	-	pos	389.25007600934	0.048148339110716	56.1	-
pos_25033	Gly Asn Gly Thr Ile	metab_25032	B(i)	59.1	0	-	M+H	C18H32N6O8	3.8268833333333	-	pos	461.23445527885	0.066401257315642	50	-
pos_25055	Strophanthin	metab_25054	B(ii)	0	77.9	HMDB0242744	M+H-2H2O	C36H54O14	3.8215333333333	-	pos	675.34419572232	0.013891070098509	52	-
pos_25057	Paraherquamide A	metab_25056	B(i)	59.6	0	-	M+H	C28H35N3O5	3.8215333333333	-	pos	494.27068400556	0.027503884612892	46.7	-
pos_25070	Leu-Arg-Pro	metab_25069	B(i)	82.2	0	-	M+H	C17H32N6O4	3.8215333333333	-	pos	385.25478661528	0.022894708553137	54	-
pos_25074	Lys-Ala-Ile	metab_25073	B(i)	40.3	0	-	M+Na	C15H30N4O4	3.8215333333333	-	pos	353.21561793971	0.058141139358551	44.4	-
pos_25085	3-Methyl-3,4-Dihydro-2H-1,4-Benzoxazine	metab_25084	B(i)	52.7	0	-	M+ACN+H	C9H11NO	3.8215333333333	-	pos	191.11738718021	0.077275173918205	48.1	-
pos_25104	Ser Gly Phe	metab_25103	B(i)	84.1	0	-	M+H	C14H19N3O5	3.81625	-	pos	310.13898764086	0.015700837070697	55.3	-
pos_25124	Asp-Val-Lys	metab_25123	B(i)	48.8	0	-	M+H	C15H28N4O6	3.8109166666667	-	pos	361.2070907	0.091552924453284	47	-
pos_25138	poststatin	metab_25137	B(ii)	0	44.4	HMDB0256711	M+	C26H47N5O7	3.8056	-	pos	541.34438418756	0.039678622994237	44.7	-
pos_25146	Lys-Trp	metab_25145	B(i)	59.4	0	HMDB0028962	M+NH4	C17H24N4O3	3.8056	-	pos	350.21778820255	0.093209321318541	49	50674-18-5

pos_25151	Pyr-Phe-Oh	metab_25150	B(i)	65.7	0	-	M+H	C14H16N2O4	3.8056	-	pos	277.11757795267	0.015491614634484	51.7	-
pos_25166	Ethyl 2-Amino-3-(4-Hydroxyphenyl)Propanoate	metab_25165	B(i)	94.6	0	HMDB0253924	M+H	C11H15NO3	3.8003	-	pos	210.11197213592	0.022414016253931	57.8	-
pos_25192	Phylanthoside	metab_25191	B(ii)	0	78.7	HMDB0256512	M+H-H2O	C40H52O17	3.7897	-	pos	787.32322091056	0.028712812742238	51.3	-
pos_25202	Thr Pro Leu	metab_25201	B(i)	92.3	0	-	M+H	C15H27N3O5	3.7897	-	pos	330.20136681364	0.061620839413763	55.2	-
pos_25237	Gly Gly Phe	metab_25236	B(i)	71.1	0	-	M+H	C13H17N3O4	3.7738166666667	-	pos	280.12852523987	0.090701720658182	51.3	-
pos_25249	Chlorobenzene	metab_25248	B(ii)	0	64	HMDB0041855	M+ACN+H	C6H5Cl	3.7685333333333	C06990	pos	154.0413563	0.009973621906215	46.8	108-90-7
pos_25251	Indole	metab_25250	B(i)	48	0	HMDB0000738	M+H	C8H7N	3.7685333333333	C00463	pos	118.06483329556	0.028350354468353	47.7	120-72-9
pos_25262	Phe Tyr Asn Glu	metab_25261	B(i)	75.3	0	-	M+H	C27H33N5O9	3.7579	-	pos	572.23558863015	0.27786511885537	50.3	-
pos_25267	Ile Leu Gln	metab_25266	B(i)	55.8	0	-	M+H	C17H32N4O5	3.7579	-	pos	373.24330724465	0.005625336428986	47.4	-
pos_25279	1-(2,6-Dimethoxyphenoxy)-3-[4-(4-Fluorophenyl)Piperazin-1-Yl]Propan-2-Ol	metab_25278	B(i)	40.6	0	-	M+H	C21H27FN2O4	3.7526166666667	-	pos	391.20708753057	0.04837424668181	43.3	-
pos_25286	Betaine	metab_25285	B(i)	75.7	0	-;HMDB0000043;PW_C000030	M+H	C5H11NO2	3.7526166666667	_;C00719	pos	118.08596517666	0.03797597035346	53.4	107-43-7;6915-17-9
pos_25287	Pgp(Txb2/A-17:0)	metab_25286	B(ii)	0	62.2	HMDB0274512	M+H-2H2O	C43H80O17P2	3.74735	-	pos	895.47214567121	0.032712609380708	48	-
pos_25308	Val-Glu-Pro-Ile-Pro-Tyr	metab_25307	B(ii)	0	60.2	HMDB0259739	M+K	C35H52N6O10	3.7420166666667	-	pos	755.33580292568	0.082426773995752	45.7	-
pos_25366	Dehydrocorydaline	metab_25365	B(i)	37.2	0	-	M+H	C22H24NO4+	3.73135	C17790	pos	367.18031342397	0.037522850101807	36.3	30045-16-0
pos_25391	Phe-Gln-His	metab_25390	B(i)	44.4	0	-	M+H	C20H26N6O5	3.7260166666667	-	pos	431.2028607	0.043694682793219	47	-
pos_25418	Ile Gln Asn Glu	metab_25417	B(i)	49.5	0	-	M+H	C20H34N6O9	3.7207166666667	-	pos	503.24484155549	0.009134887896647	45.9	-
pos_25447	3-(Benzenesulfonyl)-N-[(1-Cyclopentylpiperidin-4-Yl)Methyl]Propanamide	metab_25446	B(i)	51.9	0	-	M+H	C20H30N2O3S	3.7154333333333	-	pos	379.20714744998	0.01246455284825	45.9	-
pos_25448	Asp Val Val	metab_25447	B(i)	81.1	0	-	M+H	C14H25N3O6	3.7154333333333	-	pos	332.18101792853	0.02410853019823	55	-
pos_25455	Ala-Phe	metab_25454	B(i)	93.5	0	HMDB0028694	M+H	C12H16N2O3	3.7154333333333	-	pos	237.12281267569	0.013740985071737	57.5	3061-90-3
pos_25458	1,5-Naphthalenediamine	metab_25457	B(i)	58.5	0	HMDB0244231	M+H	C10H10N2	3.7154333333333	C19463	pos	159.09127860882	0.06490540461566	49.9	2243-62-1;_
pos_25501	Arg-Ile-Tyr	metab_25500	B(i)	77.3	0	-	M+H	C21H34N6O5	3.6995166666667	-	pos	451.26554279508	0.072262897362117	51.8	-
pos_25531	Pro His Leu	metab_25530	B(i)	48.7	0	-	M+H	C17H27N5O4	3.6941666666667	-	pos	366.21276327299	0.009093170779194	46.8	-
pos_25535	Val Pro Val	metab_25534	B(i)	85	0	-	M+H	C15H27N3O4	3.6941666666667	-	pos	314.20660167186	0.042436070586446	54.9	-
pos_25539	His-Pro	metab_25538	B(i)	83.2	0	HMDB0028893	M+H	C11H16N4O3	3.6941666666667	-	pos	253.12891272443	0.034218862253247	55.1	20930-58-9
pos_25581	Thromboxane B2	metab_25580	B(i)	62.5	0	HMDB0003252;PW_C001820	M+Na	C20H34O6	3.6835833333333	C05963	pos	393.2225275	0.019411918667119	49.9	54397-85-2
pos_25584	Ala-Gln-Leu	metab_25583	B(i)	79.7	0	-	M+H	C14H26N4O5	3.6835833333333	-	pos	331.19642461342	0.047269008455092	53.4	-
pos_25585	Ala-Ser-Ile	metab_25584	B(i)	93.5	0	-	M+H	C12H23N3O5	3.6835833333333	-	pos	290.170388	0.016052119228508	57.6	-
pos_25613	Ile-Met	metab_25612	B(i)	65.1	0	HMDB0028913	M+H	C11H22N2O3S	3.67825	-	pos	263.14139401262	0.010403153606927	49.7	-
pos_25641	Gly Glu Leu	metab_25640	B(i)	46.6	0	-	M+H	C13H23N3O6	3.6727	-	pos	318.16509828386	0.069647002154392	47.6	-
pos_25649	Corchorusoside D	metab_25648	B(ii)	0	60.8	HMDB0033085	M+K	C35H54O14	3.6674166666667	-	pos	737.3196589	0.034253697909599	47.8	210637-18-6
pos_25660	Strophanthidin Semicarbazide	metab_25659	B(i)	37.6	0	-	M+H	C24H35N3O6	3.6674166666667	-	pos	462.25473545302	0.024301148568682	43.8	-
pos_25676	Galactosylpyridinoline	metab_25675	B(ii)	0	70.5	HMDB0252583	M+CH3OH+H	C24H38N4O13	3.6620833333333	-	pos	623.27684809819	0.091224285390385	49.1	-
pos_25695	Arginyllysine	metab_25694	B(ii)	0	44.6	HMDB0028714	M+Na-H2O	C12H26N6O3	3.6620833333333	-	pos	307.18587709794	0.073594870902089	46.2	40968-46-5
pos_25700	Phenylalanyl-Glycine	metab_25699	B(ii)	0	40.1	HMDB0304788	M+H	C11H14N2O3	3.6620833333333	-	pos	223.10719977232	0.013106077165779	46.9	-
pos_25701	6-Hydroxypseudooxynicotine	metab_25700	B(ii)	0	45.6	HMDB0240264	M+H-H2O	C10H14N2O2	3.6620833333333	C01297	pos	177.10180489365	0.049409265127737	47.7	-;7424-35-3
pos_25740	1-O-All-Trans-Retinoyl-Beta-Glucuronic Acid	metab_25739	B(ii)	0	42.4	HMDB0062632	M+NH4	C26H36O8	3.6515	C11061;_	pos	494.27056665317	0.080349634775945	43.2	_;
pos_25750	Pro-Pro-Pro	metab_25749	B(ii)	0	46.2	HMDB0256689	M+H	C15H23N3O4	3.6515	-	pos	310.1741075	0.036057409334286	44.4	-
pos_25757	(R)-Alpha-Amino-3-Hydroxy-5-Methyl-4-Isoxazolepropionic	metab_25756	B(i)	40	0	-	M+H	C7H10N2O4	3.6515	-	pos	187.07086047103	0.011931673209873	45.6	-
pos_25773	Ile His Ile	metab_25772	B(i)	41.5	0	-	M+H	C18H31N5O4	3.6462166666667	-	pos	382.24394341209	0.032304531732457	46.2	-
pos_25777	Ala Val Val	metab_25776	B(i)	60.1	0	-	M+H	C13H25N3O4	3.6462166666667	-	pos	288.19169308275	0.028338541560143	50.7	-
pos_25803	Gly-Pro-Arg	metab_25802	B(i)	61.4	0	-	M+K	C13H24N6O4	3.6409333333333	-	pos	367.14951243591	0.072218039934122	50.2	-
pos_25853	Fumagillol	metab_25852	B(ii)	0	41.4	HMDB0252515	M+ACN+Na	C16H26O4	3.6303333333333	C22618;_	pos	346.19622360859	0.051656442346817	30.3	108102-51-8;_
pos_25860	Val-Leu	metab_25859	B(i)	80.4	0	HMDB0029131	M+H	C11H22N2O3	3.6303333333333	-	pos	231.16971245474	0.016645825960735	55	3989-97-7
pos_25954	Phe Asn	metab_25953	B(i)	53.4	0	-	M+H	C13H17N3O4	3.6090333333333	-	pos	280.12849926006	0.025211003220182	49	-

pos_25978	Ala-Leu-Lys	metab_25977	B(i)	91.3	0	-	M+H	C15H30N4O4	3.6037333333333	-	pos	331.23308130955	0.039709522698628	56.5	-
pos_25979	Leu Pro Thr	metab_25978	B(i)	94.6	0	-	M+H	C15H27N3O5	3.6037333333333	-	pos	330.20138977446	0.012109821290456	56.3	-
pos_26005	Pyroglutamylisoleucylarginine	metab_26004	B(i)	84.6	0	-	M+H	C17H30N6O5	3.5984333333333	-	pos	399.23407604864	0.026402630865384	54	-
pos_26012	N-[(2E)-3-(4-Hydroxyphenyl)-2-Propenoyl]-L-Tyrosine	metab_26011	B(i)	47.3	0	-	M+NH4	C18H17NO5	3.5984333333333	-	pos	345.14340038793	0.04039506996546	47.6	-
pos_26014	Ser Val Val	metab_26013	B(i)	77.1	0	-	M+H	C13H25N3O5	3.5984333333333	-	pos	304.18586206552	0.027192319845826	53.4	-
pos_26033	Methyldopa	metab_26032	B(i)	52.5	0	HMDB0011754	M+ACN+H	C10H13NO4	3.5930833333333	C07194	pos	253.11769341843	0.015229308066674	49.4	555-30-6
pos_26092	Val Ala His Glu	metab_26091	B(i)	46.3	0	-	M+H	C19H30N6O7	3.5771166666667	-	pos	455.22135972804	0.04723997131783	45.6	-
pos_26101	Ala Phe Glu	metab_26100	B(i)	62.9	0	-	M+H	C17H23N3O6	3.5771166666667	-	pos	366.16544162552	0.02560092273832	51.2	-
pos_26109	Naloxegol	metab_26108	B(ii)	0	48.5	HMDB0255436	M+K	C34H53NO11	3.5718166666667	-	pos	690.32799742471	0.077910640543879	45.4	-
pos_26141	Pro Gln Ile	metab_26140	B(i)	42.8	0	-	M+H	C16H28N4O5	3.5664833333333	-	pos	357.21226787141	0.013009989301869	47.3	-
pos_26145	Formacidine	metab_26144	B(ii)	0	67.7	HMDB0252438	M+K	C43H74N2O14	3.5612	-	pos	881.48141876432	0.063096420988913	49	-
pos_26147	S-(Pga2)-Glutathione	metab_26146	B(ii)	0	64.5	HMDB0013062	M+NH4	C30H47N3O10	3.5612	-	pos	659.33442706175	0.063603384836568	47.9	-
pos_26153	N-Docosahexaenoyl Tryptophan	metab_26152	B(ii)	0	46.9	HMDB0242023	M+H-H2O	C33H42N2O3	3.5612	-	pos	497.31823796951	0.014819267080788	45.2	-
pos_26167	Met-Tyr	metab_26166	B(i)	50	0	HMDB0028985	M+H	C14H20N2O4S	3.5612	-	pos	313.12084652692	0.021680553829335	47.1	-
pos_26173	4-(3-Amino-2-Hydroxypropoxy)Phenylacetamide	metab_26172	B(i)	43.8	0	-	M+NH4	C11H16N2O3	3.5612	-	pos	242.14928128242	0.060071714773304	46.3	-
pos_26195	Val Gly Val	metab_26194	B(i)	59.9	0	-	M+H	C12H23N3O4	3.5559	-	pos	274.17587596163	0.020432769125556	50.9	-
pos_26222	Beclometasone Dipropionate	metab_26221	B(ii)	0	62.4	HMDB0014538	M+H	C28H37ClO7	3.5453666666667	C07813	pos	521.23410430112	0.037989730824733	43	5534/9/8;5534-09-8
pos_26245	Asp Gln Leu Thr Glu	metab_26244	B(i)	40	0	-	M+H	C24H40N6O12	3.5400833333333	-	pos	605.27563455704	0.16391783290569	42.8	-
pos_26259	Dimethylarginine	metab_26258	B(ii)	0	42.8	HMDB0251395	M+H	C8H18N4O2	3.5400833333333	-	pos	203.14976414959	0.042492625655581	47.1	-
pos_26325	Val Ile Ala	metab_26324	B(i)	80.3	0	-	M+H	C14H27N3O4	3.5295166666667	-	pos	302.20671833531	0.005170096784395	54.6	-
pos_26330	Ile-Val	metab_26329	B(i)	88.5	0	HMDB0028920	M+H-H2O	C11H22N2O3	3.5295166666667	-	pos	213.15923079604	0.019106052944759	56.3	-
pos_26353	Hexazinone	metab_26352	B(i)	36.4	0	HMDB0253153	M+NH4	C12H20N4O2	3.5242166666667	C10926	pos	270.19175429939	0.078497029607842	44.2	51235-04-2;_
pos_26375	Torachrysone 8-Beta-Gentiobioside	metab_26374	B(ii)	0	51.2	HMDB0034613	M+NH4	C26H34O14	3.5135833333333	-	pos	588.22830548292	0.040968388913127	48.5	245724-09-8
pos_26383	Dilept	metab_26382	B(ii)	0	62.1	HMDB0251364	M+CH3OH+Na	C21H30N2O5	3.5135833333333	-	pos	445.2280346	0.011445348107321	49.3	-
pos_26407	Pro-Tyr-Arg	metab_26406	B(i)	40.4	0	-	M+H	C20H30N6O5	3.5082833333333	-	pos	435.2340833	0.013241652141744	46.7	-
pos_26421	Ethyl Tiglate	metab_26420	B(ii)	0	48.1	HMDB0038962	M+NH4	C7H12O2	3.5082833333333	C08487	pos	146.11719281876	0.029150003328972	47.8	5837-78-5;
pos_26454	Tryptophyl-Glutamate	metab_26453	B(ii)	0	55	HMDB0029082	2M+H	C16H19N3O5	3.4977333333333	-	pos	667.26634948444	0.032533295783328	47.6	-
pos_26462	Ile Phe Arg	metab_26461	B(i)	66.1	0	-	M+H	C21H34N6O4	3.4977333333333	-	pos	435.27013352345	0.029098603820197	51.1	-
pos_26534	Leu Tyr Phe Lys	metab_26533	B(i)	43.8	0	-	M+H	C30H43N5O6	3.4819333333333	-	pos	570.33420168902	0.097177794334292	44.3	-
pos_26548	Asp-Leu	metab_26547	B(i)	92.8	0	HMDB0028757	M+H	C10H18N2O5	3.4819333333333	-	pos	247.1282471	0.027657668556773	57.2	-
pos_26569	Fenvalerate	metab_26568	B(ii)	0	48.2	HMDB0031791	M+H-2H2O	C25H22ClNO3	3.4766666666667	C10988	pos	384.11406082091	0.002233812511226	42	51630-58-1
pos_26613	Homostachydrine	metab_26612	B(i)	65.1	0	HMDB0033433;HMDB0304552;HMDB0033433	M+NH4	C8H15NO2	3.4713833333333	C08283	pos	175.14370766334	0.055937404543243	51.1	1195-94-4;472-22-0
pos_26620	Lys Asn Gln Glu	metab_26619	B(i)	46	0	-	M+H	C20H35N7O9	3.4660833333333	-	pos	518.25562658021	0.019883896233392	46.1	-
pos_26672	Enkephalin L	metab_26671	B(ii)	0	47.6	HMDB0001045	M+Na-H2O	C28H37N5O7	3.4554833333333	-	pos	560.24837594403	0.077552332014279	46.7	14-18-6
pos_26687	Gln-Asn-Leu	metab_26686	B(i)	39.2	0	-	M+H	C15H27N5O6	3.4554833333333	-	pos	374.20090372034	0.016434079089427	43.8	-
pos_26727	4-(Dimethylamino)Benzaldehyde	metab_26726	B(i)	60.2	0	HMDB0246303	M+ACN+H	C9H11NO	3.4502166666667	-	pos	191.11732058496	0.034137850718069	50.2	-
pos_26784	Arginylisoleucine	metab_26783	B(ii)	0	51.9	HMDB0028712	M+H	C12H25N5O3	3.4396166666667	-	pos	288.20225136216	0.005209810074121	48.7	62632-70-6
pos_26785	Val-Gly	metab_26784	B(i)	81.9	0	HMDB0259740	M+H-H2O	C7H14N2O3	3.4396166666667	-	pos	157.09674378863	0.030150668293196	54.2	-
pos_26857	Tenuifolin	metab_26856	B(ii)	0	52.1	HMDB0258821	M+Na	C36H56O12	3.41845	-	pos	703.3708243	0.059358364309682	45.1	-
pos_26880	7-Ethylguanine	metab_26879	B(i)	44.3	0	-	M+H	C7H9N5O	3.41845	-	pos	180.0874938	0.047365550109273	46.4	-
pos_26895	Leu Gln Ala	metab_26894	B(i)	96.5	0	-	M+H	C14H26N4O5	3.4131166666667	-	pos	331.19680572881	0.033730952739834	57.7	-
pos_26898	Ile Ser Ala	metab_26897	B(i)	71.3	0	-	M+H	C12H23N3O5	3.4131166666667	-	pos	290.17039114672	0.061994233830237	52.7	-
pos_26925	Spermic Acid 2	metab_26924	B(ii)	0	45.7	HMDB0013075	M+H	C10H20N2O4	3.4077833333333	-	pos	233.14905903207	0.021533133374593	48.2	14209-33-7
pos_27000	L-Leucine	metab_26999	B(i)	91.9	0	HMDB0000687;PW_C000540	M+H	C6H13NO2	3.39195	C00123	pos	132.10157955645	0.012893526074403	57.3	61-90-5

pos_27015	Glu Pro Arg	metab_27014	B(i)	73.4	0	-	M+H	C16H28N6O6	3.3866833333333	-	pos	401.21328326731	0.090009290107846	52.6	-
pos_27029	Met-Val	metab_27028	B(i)	74.1	0	HMDB0028986	M+H	C10H20N2O3S	3.3866833333333	-	pos	249.12614074894	0.031170189881846	52	-
pos_27030	Pro-Ile	metab_27029	B(i)	51.2	0	HMDB0304810	M+H	C11H20N2O3	3.3866833333333	-	pos	229.15413363325	0.036033371975774	49.2	-
pos_27047	Terbogrel	metab_27046	B(ii)	0	49.6	HMDB0258827	M+H-H2O	C23H27N5O2	3.3814	-	pos	388.21269389897	0.02269802184121	48.8	-
pos_27063	His Lys Ile Asp	metab_27062	B(i)	40.4	0	-	M+H	C22H37N7O7	3.3761333333333	-	pos	512.28148168729	0.064271675180253	46.2	-
pos_27078	Trp Ser	metab_27077	B(i)	86	0	-	M+H	C14H17N3O4	3.3761333333333	-	pos	292.12873134846	0.12207722809715	54.8	-
pos_27087	Phe Gly	metab_27086	B(i)	81.4	0	-	M+H	C11H14N2O3	3.3761333333333	-	pos	223.10715972326	0.010266002385196	55	-
pos_27110	Pe(6 Keto-Pgf1Alpha/14:0)	metab_27109	B(ii)	0	68.7	HMDB0260599	M+H-2H2O	C39H72NO12P	3.3655833333333	-	pos	742.46651956898	0.10154657729105	49.4	-
pos_27196	Tyr Val	metab_27195	B(i)	64.9	0	-	M+H	C14H20N2O4	3.3550333333333	-	pos	281.14880564542	0.012288293527727	52	-
pos_27220	Prolyl-Arginine	metab_27219	B(ii)	0	46.1	HMDB0029011	M+H	C11H21N5O3	3.3497666666667	-	pos	272.1711341	0.010849318476168	42.1	-
pos_27229	3-Amino-3-(4-Hydroxyphenyl)Propanoate	metab_27228	B(i)	72.4	0	HMDB0003831	M+H	C9H11NO3	3.3497666666667	C04368	pos	182.08071411057	0.03219301357881	52.8	-
pos_27248	Pro Met	metab_27247	B(i)	46.1	0	-	M+H	C10H18N2O3S	3.3445	-	pos	247.1105023	0.046258365701848	47	-
pos_27258	Tyr Gln Asn Glu	metab_27257	B(i)	60.3	0	-	M+H	C23H32N6O10	3.3392333333333	-	pos	553.22342916418	0.088922247184193	49	-
pos_27260	Blumenol C O-[Apiosyl-(1->6)-Glucoside]	metab_27259	B(ii)	0	50.1	HMDB0031936	M+Na-H2O	C24H40O11	3.3392333333333	-	pos	509.23415421837	0.033283830259361	48.8	177261-68-6
pos_27286	2-Phenylacetamide	metab_27285	B(i)	58	0	HMDB0010715	M+H	C8H9NO	3.3392333333333	C02505	pos	136.07534944159	0.046981058868035	49.6	103-81-1
pos_27309	Asp Leu	metab_27308	B(i)	63.4	0	-	M+H	C10H18N2O5	3.3339	-	pos	247.12827168616	0.009778862668224	51	-
pos_27385	Ne,Ne Dimethyllysine	metab_27384	B(ii)	0	49	HMDB0013287	M+H-H2O	C8H18N2O2	3.3180666666667	C05545	pos	157.13314160582	0.012484400876195	48.2	-
pos_27435	Ala-Ile-Lys	metab_27434	B(i)	87.9	0	-	M+H	C15H30N4O4	3.3075	-	pos	331.23309094627	0.053191402055183	54.3	-
pos_27450	Arg Glu Trp	metab_27449	B(i)	49.9	0	-	M+H	C22H31N7O6	3.3022333333333	-	pos	490.2462991	0.080464633817135	44.6	-
pos_27487	Trp Asn	metab_27486	B(i)	91.7	0	-	M+H	C15H18N4O4	3.2969833333333	-	pos	319.13935578882	0.024141166247782	56.7	-
pos_27545	N,N'-Diacetylchitobiose	metab_27544	B(ii)	0	75.6	HMDB0062702	M+NH4	C16H28N2O11	3.2811833333333	C01674	pos	442.20150284694	0.026200781245827	51.7	35061-50-8
pos_27552	Gly Pro Arg	metab_27551	B(i)	41.6	0	-	M+H	C13H24N6O4	3.2811833333333	-	pos	329.19199416437	0.068608609298936	44.4	-
pos_27557	Asn Leu	metab_27556	B(i)	94.9	0	-	M+H	C10H19N3O4	3.2811833333333	-	pos	246.14429563188	0.016535666141768	57.6	-
pos_27595	Arg-Val-Tyr	metab_27594	B(i)	85	0	-	M+H	C20H32N6O5	3.2705666666667	-	pos	437.2496042	0.06284507600054	52.3	-
pos_27604	(2S)-2-(2-Benzamidoethylamino)-5-(Diaminomethylideneamino)Pentanoic Acid	metab_27603	B(ii)	0	61.4	HMDB0257707	M+H	C15H23N5O3	3.2705666666667	-	pos	322.18674182464	0.03806254908053	50	-
pos_27630	5-Formiminotetrahydrofolic Acid	metab_27629	B(ii)	0	46.8	HMDB0001534	M+CH3OH+Na	C20H24N8O6	3.2600333333333	C00664	pos	527.19707918627	0.17734739583138	46.9	2311-81-1
pos_27642	Ile-Pro-Asn	metab_27641	B(i)	83.9	0	-	M+H	C15H26N4O5	3.2600333333333	-	pos	343.19666538925	0.16992512868857	52.9	-
pos_27662	Ser Ile Gln Glu	metab_27661	B(i)	42.3	0	-	M+H	C19H33N5O9	3.2545	-	pos	476.23377265414	0.030965455322265	47.6	-
pos_27663	Gln Ile Glu	metab_27662	B(i)	77.9	0	-	M+H	C16H28N4O7	3.2545	-	pos	389.20200639931	0.013058671674704	44.3	-
pos_27665	Met-Asp-Lys	metab_27664	B(i)	71.1	0	-	M+H-H2O	C15H28N4O6S	3.2545	-	pos	375.16898981839	0.051915517928294	48.7	-
pos_27676	Arginine Ethyl Ester	metab_27675	B(i)	74.9	0	-	M+H	C8H18N4O2	3.2545	-	pos	203.149777	0.041342749583447	53.3	-
pos_27716	His Gln Phe	metab_27715	B(i)	83.1	0	-	M+H	C20H26N6O5	3.2438666666667	-	pos	431.20245263583	0.10027189444929	52.2	-
pos_27717	Phe Gln Gln	metab_27716	B(i)	86.2	0	-	M+H	C19H27N5O6	3.2438666666667	-	pos	422.20336886174	0.038659199143994	55.7	-
pos_27760	Pro-Lys	metab_27759	B(i)	40.7	0	HMDB0029022	M+H	C11H21N3O3	3.2385833333333	-	pos	244.16485050876	0.059433358896894	46.2	-
pos_27797	Alpha-Crocetin Glucosyl Ester	metab_27796	B(ii)	0	51.4	HMDB0039120	M+H-2H2O	C26H34O9	3.2279333333333	-	pos	455.20546673839	0.068800510287001	45.1	58050-17-2
pos_27800	N-[2-(4-Methoxyphenyl)-7-Methylimidazo[1,2-A]Pyridin-3-Yl]-2-Phenylbutanamide	metab_27799	B(i)	45.8	0	-	M+H	C25H25N3O2	3.2279333333333	-	pos	400.20587111102	0.16225821876047	43	-
pos_27815	N-Acetylcaprolactam	metab_27814	B(i)	67.4	0	-	M+NH4	C8H13NO2	3.2279333333333	-	pos	173.12800588524	0.002488723645553	51.6	-
pos_27858	Ser Tyr Lys Asp	metab_27857	B(i)	35.9	0	-	M+H	C22H33N5O9	3.2119666666667	-	pos	512.23361241818	0.013017206752763	45.7	-
pos_27870	Ala Pro Val	metab_27869	B(i)	54	0	-	M+H	C13H23N3O4	3.2119666666667	-	pos	286.17510738792	0.014252062267848	48.2	-
pos_27880	Dorrigocin A	metab_27879	B(i)	47.8	0	-	M+H	C27H41NO8	3.2066333333333	-	pos	508.28656043715	0.15356540858991	45.9	-
pos_27883	Lasofoxifene	metab_27882	B(ii)	0	47.6	HMDB0253987	M+CH3OH+H	C28H31NO2	3.2066333333333	-	pos	446.27111184668	0.031379529714059	45.2	-
pos_27884	Met Ile Phe	metab_27883	B(i)	53.7	0	-	M+H	C20H31N3O4S	3.2066333333333	-	pos	410.2096179	0.24328109527373	45.9	-
pos_27888	1,1'-(2,6-Dimethyl-4-(3-Nitrophenyl)-1,4-Dihydropyridine-3,5-Diyl)Diethanone	metab_27887	B(i)	48.3	0	-	M+NH4	C17H18N2O4	3.2066333333333	-	pos	332.16333848515	0.028886959902396	44.6	-
pos_27889	Dienogest	metab_27888	B(ii)	0	43.9	HMDB0251234	M+NH4	C20H25NO2	3.2066333333333	-	pos	329.22300934308	0.032534754202464	44.8	-

pos_27965	Homo-L-Arginine	metab_27964	B(ii)	0	41.9	HMDB0000670	M+Na	C7H16N4O2	3.1906833333333	C01924	pos	211.11840507159	0.033449024665231	42.7	156-86-5
pos_27967	Neuac(Alpha->6)Galnac(Alpha1->O)Ser	metab_27966	B(ii)	0	80.6	HMDB0255554	M+CH3OH+Na	C22H37N3O16	3.1853833333333	-	pos	654.23510928705	0.089153325874996	52.4	-
pos_27991	2",4",6"-Triacetylglycitin	metab_27990	B(ii)	0	66	HMDB0034694	M+CH3OH+Na	C28H28O13	3.1747333333333	-	pos	627.16555919347	0.21599861861626	47.6	-
pos_28005	1,3-Diazaspiro[4.5]Decane-2,4-Dione	metab_28004	B(i)	37.3	0	-	M+H	C8H12N2O2	3.1747333333333	-	pos	169.09673542299	0.035987943000265	45	-
pos_28010	1'-[2-(3,4-Diethoxyphenyl)Acetyl]-1-Methylspiro[Indole-3,4'-Piperidine]-2-One	metab_28009	B(i)	67.9	0	-	M+H	C25H30N2O4	3.1691666666667	-	pos	423.23216040878	0.016525895172758	47.3	-
pos_28027	Arg-Asn-Glu	metab_28026	B(i)	46	0	-	M+H-H2O	C15H27N7O7	3.1638	-	pos	400.19294571336	0.044164254864705	45.8	-
pos_28058	Sagopilone	metab_28057	B(ii)	0	47.1	HMDB0257445	M+Na	C30H41NO6S	3.1424333333333	-	pos	566.25526961912	0.022554412574921	45.4	-
pos_28073	L-Furosine	metab_28072	B(ii)	0	47	HMDB0029390	M+H-2H2O	C12H18N2O4	3.1424333333333	-	pos	219.11038327561	0.038176101404731	44.6	19746-33-9
pos_28086	N-(3,5-Dimethylphenyl)-2-(5-Pyrazin-2-Yl-1,3,4-Oxadiazol-2-Yl)Acetamide	metab_28085	B(i)	71	0	-	M+H	C16H15N5O2	3.1317833333333	-	pos	310.12803139684	0.015552688342767	51.6	-
pos_28103	Adomeglivant	metab_28102	B(ii)	0	53.6	HMDB0248020	M+H	C32H36F3NO4	3.1210833333333	-	pos	556.26785070393	0.2469155451479	44.2	-
pos_28109	Val Val Lys	metab_28108	B(i)	74.2	0	-	M+H	C16H32N4O4	3.1210833333333	-	pos	345.24873546931	0.017469034297581	53.2	-
pos_28110	Arginylleucine	metab_28109	B(ii)	0	54	HMDB0028713	M+H	C12H25N5O3	3.1210833333333	-	pos	288.20211663485	0.009715968114079	48.9	1188-24-5
pos_28129	N-Fructosyl Phenylalanine	metab_28128	B(i)	75.1	0	-	M+H	C15H21NO7	3.1157666666667	-	pos	328.13825819203	0.007899222195756	53.4	-
pos_28151	Thr Pro Lys	metab_28150	B(i)	75.3	0	-	M+H	C15H28N4O5	3.0945166666667	-	pos	345.21293342661	0.013033796173032	52.1	-
pos_28152	Gln-Ile	metab_28151	B(i)	71.7	0	HMDB0028800	M+H	C11H21N3O4	3.0945166666667	-	pos	260.15992280726	0.008656266683842	51.2	86416-45-7
pos_28182	Coproporphyrin Iii Tetramethyl Ester	metab_28181	B(i)	35.4	0	-	M+NH4	C40H46N4O8	3.0569333333333	-	pos	728.36647436729	0.006422027907718	43.2	-
pos_28185	Dn-Isobutylamide	metab_28184	B(ii)	0	54.4	HMDB0251551	M+ACN+Na	C20H32N6O5	3.0462166666667	-	pos	500.25496468901	0.15727829867513	44.8	-
pos_28211	Arg Pro Gly	metab_28210	B(i)	62.4	0	-	M+H	C13H24N6O4	2.9875666666667	-	pos	329.19231863392	0.014465896772153	50.1	-
pos_28213	3-(1-Pyrazolyl)-Alanine	metab_28212	B(i)	68.5	0	-	M+H	C6H9N3O2	2.9875666666667	-	pos	156.07634947151	0.05897812332565	52	-
pos_28218	Ile-Gly-Ser	metab_28217	B(i)	35.2	0	-	M+Na	C11H21N3O5	2.9662333333333	-	pos	298.13692174567	0.012051974931443	44	-
pos_28224	His-Ser-Leu	metab_28223	B(i)	94.3	0	-	M+H	C15H25N5O5	2.9343166666667	-	pos	356.19195382843	0.02127557952303	55.9	-
pos_28231	Carbidopa	metab_28230	B(i)	54.3	0	HMDB0014336;HMDB0246122	M+H-H2O	C10H14N2O4	2.9182666666667	-	pos	209.09162278017	0.18754144383052	48.5	28860-95-9;_
pos_28236	Lpc(18:5-Sn2)	metab_28235	B(i)	69.7	0	-	M+H	C26H44NO7P	2.8971	-	pos	514.296997	0.015114616904008	50.3	-
pos_28246	Ile-Ile-His	metab_28245	B(i)	95.7	0	-	M+H	C18H31N5O4	2.8330166666667	-	pos	382.24387349856	0.009776597172312	57	-
pos_28248	6-Epi-7-Isocucurbic Acid Glucoside	metab_28247	B(ii)	0	41.8	HMDB0029782	M+Na-H2O	C18H30O8	2.8064	-	pos	379.17629737485	0.28192249803467	43.6	176486-13-8
pos_28249	Arg-Gly-Ile	metab_28248	B(i)	85	0	-	M+H	C14H28N6O4	2.8011	-	pos	345.2232094	0.055265420060147	54	-
pos_28250	Tyr-Gly-Gly-Phe-Gly-Oh	metab_28249	B(ii)	0	68.8	HMDB0259341	M+NH4	C24H29N5O7	2.7904	-	pos	517.24533224533	0.028242827013451	49.8	-
pos_28252	5-(3-Pyridyl)-2-Hydroxytetrahydrofuran	metab_28251	B(ii)	0	43.9	HMDB0062407	M+H	C9H11NO2	2.7904	C19578	pos	166.08574074187	0.000628556635456	46.1	53798-73-5
pos_28253	Glu Tyr	metab_28252	B(i)	86.1	0	-	M+H	C14H18N2O6	2.7850833333333	-	pos	311.12301085177	0.015069080766118	54.6	-
pos_28255	3-Acetyl-2,5-Dimethylfuran	metab_28254	B(i)	45.7	0	HMDB0029563	M+H-2H2O	C8H10O2	2.7850833333333	-	pos	103.05396227384	0.020285296106416	47.8	10599-70-9
pos_28261	Imidazoleacetic Acid Riboside	metab_28260	B(ii)	0	70.9	HMDB0002331	M+CH3OH+H	C10H14N2O6	2.7479333333333	C05131	pos	291.11802216115	0.2027068525583	51.3	29605-99-0
pos_28262	Nicomol	metab_28261	B(ii)	0	47.3	HMDB0255582	M+NH4	C34H32N4O9	2.7426	-	pos	658.2529385	0.044437984971991	43.8	-
pos_28265	N(Epsilon)-(Carboxyethyl)Lysine	metab_28264	B(ii)	0	65.4	HMDB0255289	M+H	C9H18N2O4	2.7426	-	pos	219.13336802008	0.012900694223125	51.2	-
pos_28276	Gln Leu Asp	metab_28275	B(i)	91.4	0	-	M+H	C15H26N4O7	2.7160666666667	-	pos	375.18636847703	0.023815807196996	56.2	-
pos_28278	2-Octenedioic Acid	metab_28277	B(ii)	0	40.2	HMDB0000341	M+NH4	C8H12O4	2.6787	-	pos	190.10695825453	0.070799029422008	45.6	5698-50-0;
pos_28280	Tyr Gly Glu	metab_28279	B(i)	92.7	0	-	M+H	C16H21N3O7	2.6520166666667	-	pos	368.14400934403	0.020471931940167	54.1	-
pos_28282	Phe Asp	metab_28281	B(i)	58.9	0	-	M+H	C13H16N2O5	2.6413666666667	-	pos	281.11238714008	0.007411030272464	50.4	-
pos_28285	Retosiban	metab_28284	B(ii)	0	53.7	HMDB0257182	M+H	C27H34N4O5	2.6201166666667	-	pos	495.26354438087	0.014496539949506	46.8	-
pos_28286	Semilepidinoside B	metab_28285	B(ii)	0	71	HMDB0033108	M+ACN+Na	C17H22N2O7	2.6147666666667	-	pos	430.15561731539	0.02702492051617	50.3	-
pos_28289	Pro Met Ala	metab_28288	B(i)	61.9	0	-	M+H	C13H23N3O4S	2.6094166666667	-	pos	318.14749496298	0.041690602415401	48	-
pos_28291	Montirelin	metab_28290	B(ii)	0	71.3	HMDB0254874	M+NH4	C17H24N6O4S	2.5828166666667	-	pos	426.19489898233	0.16854791303595	49.5	-
pos_28295	Tubocurarine	metab_28294	B(ii)	0	64.2	HMDB0015330	M+H-H2O	C37H41N2O6+	2.5614666666667	C07547	pos	592.29179365858	0.27559672621892	48.3	57-95-4;6989-98-6
pos_28297	3,4,5-Trimethoxy-N-Pyridin-2-Ylbenzamide	metab_28296	B(i)	35.6	0	-	M+H	C15H16N2O4	2.5083666666667	-	pos	289.11561571689	0.036644073599674	42.8	-
pos_28298	H-Asp-Ala-His-Lys-Oh	metab_28297	B(ii)	0	66.6	HMDB0253006	M+CH3OH+Na	C19H31N7O7	2.49745	-	pos	524.24260522526	0.062840244317287	51.2	-

pos_28300	Butyrolactone Iii	metab_28299	B(i)	38.9	0	-	M+ACN+H	C24H24O8	2.4815	-	pos	482.18484893901	0.14904129791337	42	-
pos_28302	Asp-Glu-Lys	metab_28301	B(i)	77.3	0	-	M+H	C15H26N4O8	2.4549166666667	-	pos	391.18131909786	0.019216534509198	54.2	-
pos_28306	Hydromorphone-3-Glucoside	metab_28305	B(ii)	0	61.6	HMDB0061144	M+H-2H2O	C23H29NO8	2.4282333333333	-	pos	412.17905056175	0.07613636403842	47.1	-
pos_28310	Kynurenine	metab_28309	B(i)	86	0	HMDB0250769	M+H	C10H12N2O3	2.3910166666667	C01718	pos	209.09152847356	0.054234484730012	55.1	343-65-7
pos_28314	Arg-Trp	metab_28313	B(i)	59.6	0	HMDB0028720	M+H	C17H24N6O3	2.3644166666667	-	pos	361.19707411533	0.058760679948031	49.9	25615-38-7
pos_28316	Gln Tyr	metab_28315	B(i)	91.8	0	-	M+H	C14H19N3O5	2.34315	-	pos	310.1389938	0.005369212017661	56.9	-
pos_28317	Hypoglycin B	metab_28316	B(ii)	0	42	HMDB0029428	M+Na	C12H18N2O5	2.3375333333333	C08280	pos	293.11250158653	0.017892889920939	46.3	502-37-4
pos_28320	Val Arg Val	metab_28319	B(i)	44.4	0	-	M+H	C16H32N6O4	2.3108833333333	-	pos	373.25484136872	0.001711828253950	46.9	-
pos_28323	Thr-Asp-Met	metab_28322	B(i)	64.2	0	-	M+H	C13H23N3O7S	2.2788833333333	-	pos	366.13201923242	0.011199941324168	49.8	-
pos_28324	Arginylphenylalanine	metab_28323	B(i)	84.5	0	HMDB0028716	M+H	C15H23N5O3	2.2788833333333	-	pos	322.18652563558	0.007567329967747	54.5	2047-13-4
pos_28328	Thr-Gly-Lys	metab_28327	B(i)	72.9	0	-	M+H	C12H24N4O5	2.2412833333333	-	pos	305.18130691387	0.0747179134447	52.4	-
pos_28337	Ser Phe Arg	metab_28336	B(i)	67.9	0	-	M+H	C18H28N6O5	2.1719833333333	-	pos	409.21968122343	0.10338150426812	50.5	-
pos_28341	Glu-Glu-Lys	metab_28340	B(i)	58.1	0	-	M+H	C16H28N4O8	2.1666333333333	-	pos	405.19742426494	0.006853208439293	50	-
pos_28342	2-(3-Indolyl)Propionic Acid	metab_28341	B(i)	50.2	0	-	M+NH4	C11H11NO2	2.1666333333333	-	pos	207.11223430485	0.040717361180782	48.7	-
pos_28346	Ala-Tyr	metab_28345	B(i)	90.7	0	HMDB0028699	M+H	C12H16N2O4	2.15065	-	pos	253.11764404736	0.009365188459223	57	3061-88-9
pos_28350	Asn Leu Asn	metab_28349	B(i)	37.6	0	-	M+H	C14H25N5O6	2.14005	-	pos	360.18679099694	0.015785312454388	46.1	-
pos_28353	Ryanodine	metab_28352	B(ii)	0	53.6	HMDB0257371	M+ACN+Na	C25H35NO9	2.1347	C08705;_	pos	557.24377586746	0.013109965456152	47.3	15662-33-6;_
pos_28355	Gln-Gly-His	metab_28354	B(i)	37.2	0	-	M+NH4	C13H20N6O5	2.1293333333333	-	pos	358.18365897938	0.20071372828261	44.5	-
pos_28360	Gly-Tyr	metab_28359	B(i)	97.1	0	HMDB0028853	M+H	C11H14N2O4	2.1239833333333	-	pos	239.10203169106	0.020290169017947	58.2	658-79-7
pos_28364	Pro-Arg	metab_28363	B(i)	93.7	0	HMDB0029011	M+H-H2O	C11H21N5O3	2.1132666666667	-	pos	254.16052383937	0.0152880683983	57.3	-
pos_28366	Glycyl-Tyrosyl-Glycine	metab_28365	B(ii)	0	41.1	HMDB0252836	M+H	C13H17N3O5	2.0973166666667	-	pos	296.12331208168	0.010152506022613	46.5	-
pos_28373	Leu-Gly-Ser	metab_28372	B(i)	63.6	0	-	M+Na	C11H21N3O5	2.0703666666667	-	pos	298.13667653604	0.016685835959025	50.7	-
pos_28376	Ab-Fubinaca	metab_28375	B(i)	35.7	0	HMDB0247739	M+H	C20H21FN4O2	2.0544666666667	-	pos	369.17313098862	0.082883857896015	43.4	-
pos_28378	Gln Leu Gln	metab_28377	B(i)	72.5	0	-	M+H	C16H29N5O6	2.0491666666667	-	pos	388.2181951	0.009255834754762	52.4	-
pos_28394	Val Arg Asn	metab_28393	B(i)	43.5	0	-	M+H	C15H29N7O5	2.0062833333333	-	pos	388.22901174963	0.07712340490465	45.3	-
pos_28398	Arg-Tyr	metab_28397	B(i)	93.4	0	HMDB0028721	M+H-H2O	C15H23N5O4	2.0009333333333	-	pos	320.17099097899	0.008152043380339	56.4	74863-12-0
pos_28403	Gly-Ile	metab_28402	B(i)	77.9	0	HMDB0028844	M+H	C8H16N2O3	2.0009333333333	-	pos	189.12287007453	0.018051699978081	54.4	-
pos_28425	Cromakalim	metab_28424	B(ii)	0	40.8	HMDB0250542	2M+H	C16H18N2O3	1.9690166666667	C11819	pos	573.2741052	0.069514249503337	43.2	94470-67-4;_
pos_28426	Arg Asp Phe	metab_28425	B(i)	59.8	0	-	M+H	C19H28N6O6	1.9690166666667	-	pos	437.213087	0.017931794036256	50.3	-
pos_28432	Lys Ser Thr Leu Thr	metab_28431	B(i)	46.6	0	-	M+H	C23H44N6O9	1.9636833333333	-	pos	549.32272847409	0.10151167261455	46.1	-
pos_28440	Aminoadipic Acid	metab_28439	B(ii)	0	40.9	HMDB0000510	M+H	C6H11NO4	1.9583666666667	_;C00956	pos	162.07569807737	0.051718886108172	46.1	1118-90-7;_;;542-32-5
pos_28448	L-Fuculose	metab_28447	B(ii)	0	40.3	HMDB0060267	M+Na-H2O	C6H12O5	1.9530666666667	C01721	pos	169.04662737101	0.041034713002271	45.2	-;_
pos_28458	O6-Carboxymethyl-2'-Deoxy-Guanosine	metab_28457	B(i)	83.6	0	HMDB0255879	M+K	C12H15N5O6	1.9477333333333	-	pos	364.06424968582	0.037474886435935	54.3	-
pos_28467	6-Hydroxy-2-Aminopurine	metab_28466	B(i)	98.7	0	HMDB0000132	M+H	C5H5N5O	1.9477333333333	C00242	pos	152.05624768639	0.017285191721151	57.8	73-40-5
pos_28489	Asp-Asp-Lys	metab_28488	B(i)	50.2	0	-	M+H-H2O	C14H24N4O8	1.9423833333333	-	pos	359.15520094662	0.046178071823895	47.8	-
pos_28490	Gly Lys Phe	metab_28489	B(i)	48.3	0	-	M+H	C17H26N4O4	1.9423833333333	-	pos	351.20172841724	0.030017344869891	47.9	-
pos_28492	Val Pro Gln	metab_28491	B(i)	67.9	0	-	M+H	C15H26N4O5	1.9423833333333	-	pos	343.19649713598	0.064715449362652	49.9	-
pos_28494	Ile Gln Gly	metab_28493	B(i)	58.4	0	-	M+H	C13H24N4O5	1.9423833333333	-	pos	317.181284	0.034328692747325	49.4	-
pos_28522	His Pro Gln	metab_28521	B(i)	49.2	0	-	M+H	C16H24N6O5	1.9370833333333	-	pos	381.18668250442	0.021687055984688	48.1	-
pos_28545	Ile-Ala	metab_28544	B(i)	57.6	0	HMDB0028900	M+H	C9H18N2O3	1.9370833333333	-	pos	203.13845689101	0.021227432878091	50.5	-
pos_28584	N-Fructosyl Isoleucine	metab_28583	B(i)	85.7	0	-	M+H	C12H23NO7	1.9317333333333	-	pos	294.15394262701	0.020336024796374	55.9	-
pos_28587	Leu-Arg	metab_28586	B(i)	88.3	0	HMDB0028923	M+H	C12H25N5O3	1.9317333333333	-	pos	288.20221227244	0.009773787547114	56.1	-
pos_28592	Ile Glu	metab_28591	B(i)	42	0	-	M+H	C11H20N2O5	1.9317333333333	-	pos	261.14388587998	0.007545468459716	46	-
pos_28609	Ketone Ester	metab_28608	B(ii)	0	49.9	HMDB0253792	M+NH4	C8H16O4	1.9317333333333	-	pos	194.13883403094	0.005416893258447	48.3	-
pos_28618	2-Aminoacetophenone	metab_28617	B(i)	37.2	0	HMDB0032628	M+H	C8H9NO	1.9317333333333	-	pos	136.07531669802	0.016978697396417	45.5	613-89-8

pos_28619	Tyramine	metab_28618	B(i)	62.5	0	HMDB0000306;PW_C000210	M+H-H2O	C8H11NO	1.9317333333333	C00483	pos	120.08044283905	0.011031524875629	51.5	51-67-2
pos_28639	Arg Ile Ala	metab_28638	B(i)	83.7	0	-	M+H	C15H30N6O4	1.9264333333333	-	pos	359.23934899292	0.036345407457853	53.2	-
pos_28640	Arg Leu Gly	metab_28639	B(i)	58.4	0	-	M+H	C14H28N6O4	1.9264333333333	-	pos	345.22373524339	0.043956000617426	48.7	-
pos_28676	Hemorphin-4	metab_28675	B(ii)	0	72.3	HMDB0059788	M+H	C29H35N5O7	1.9210666666667	-	pos	566.25532639352	0.070982593707397	48.7	-
pos_28683	Glycylprolylhydroxyproline	metab_28682	B(ii)	0	41.8	HMDB0002171	M+CH3OH+Na	C12H19N3O5	1.9210666666667	-	pos	340.14908989156	0.13783611948735	32.7	2239-67-0
pos_28685	Pro Val Asp	metab_28684	B(i)	56.8	0	-	M+H	C14H23N3O6	1.9210666666667	-	pos	330.16509624888	0.12307785406226	48.7	-
pos_28686	Noroxycodone-D3	metab_28685	B(i)	66.7	0	-	M+NH4	C17H[2]3H[1]16NO4	1.9210666666667	-	pos	322.18650783133	0.047226964254435	50.8	-
pos_28713	Gly-Pro-Asn	metab_28712	B(i)	42.3	0	-	M+NH4	C11H18N4O5	1.9157833333333	-	pos	304.16033278261	0.017315059720288	45.6	-
pos_28743	Asn Ile Asn	metab_28742	B(i)	66.2	0	-	M+H	C14H25N5O6	1.8842	-	pos	360.18745222903	0.14109482531379	49.9	-
pos_28748	Gly-Val-His	metab_28747	B(i)	41.8	0	-	M+H-2H2O	C13H21N5O4	1.8789	-	pos	276.14334185162	0.19693750593967	45.4	-
pos_28752	Glu-Ala	metab_28751	B(i)	35.1	0	HMDB0003764	M+H-H2O	C8H14N2O5	1.8629166666667	-	pos	201.08645047786	0.01723397789849	44.4	21064-18-6
pos_28754	Neosaxitoxin	metab_28753	B(i)	36.8	0	HMDB0029369	M+H	C10H17N7O5	1.8576	C17208	pos	316.1358229	0.050697176710587	46	64296-20-4
pos_28761	Indole-3-Carboxylic Acid	metab_28760	B(i)	60.6	0	HMDB0003320	M+H	C9H7NO2	1.8257	C19837	pos	162.05450721744	0.010377618278236	49.4	771-50-6
pos_28763	Gly Gln Arg	metab_28762	B(i)	38.9	0	-	M+H	C13H25N7O5	1.8150666666667	-	pos	360.19738127784	0.032651209894432	44.6	-
pos_28767	Gln Arg Asp	metab_28766	B(i)	39.7	0	-	M+CH3OH+Na	C15H27N7O7	1.783	-	pos	472.21365601049	0.015877368621161	46.7	-
pos_28773	Ps(3:0/18:5)	metab_28772	B(i)	35.3	0	-	M+NH4	C27H42NO10P	1.7617166666667	-	pos	589.29253445574	0.17707322451913	42.2	-
pos_28776	Acipimox	metab_28775	B(i)	39.1	0	HMDB0247941	M+H	C6H6N2O3	1.7564	-	pos	155.04605774498	0.04544650339135	44.9	-
pos_28781	Glu-Glu-Gln	metab_28780	B(i)	69.6	0	-	M+H-H2O	C15H24N4O9	1.74575	-	pos	387.15011314742	0.091508669885911	50.9	-
pos_28803	L-Isoleucine	metab_28802	B(i)	97.7	0	HMDB0000172;PW_C000112	M+H	C6H13NO2	1.62855	C00407	pos	132.10148913926	0.003832254595164	57.5	73-32-5
pos_28804	Piperidine	metab_28803	B(i)	64.4	0	HMDB0034301	M+H	C5H11N	1.62855	C01746	pos	86.096161348553	0.002915853026567	51.1	110-89-4
pos_28808	Gly Cys Val	metab_28807	B(i)	54.4	0	-	M+H	C10H19N3O4S	1.5857166666667	-	pos	278.11623097651	0.10118623595418	48.3	-
pos_28809	Asn Ser Val	metab_28808	B(i)	93.1	0	-	M+H	C12H22N4O6	1.5803666666667	-	pos	319.16037153003	0.07368597081114	56.5	-
pos_28814	Zinostatin	metab_28813	B(ii)	0	54.4	HMDB0260026	M+CH3OH+H	C35H35NO12	1.5536833333333	-	pos	694.25046821586	0.21783434771217	47	-
pos_28817	4-(4-Tert-Butylphenoxy)-N-(4-Phenyl-1,3-Thiazol-2- [illegible]	metab_28816	B(i)	45.5	0	-	M+H	C23H26N2O2S	1.5430166666667	-	pos	395.1820371	0.075913702768009	40.5	-
pos_28820	Arg-Ile	metab_28819	B(i)	88.1	0	HMDB0028712	M+H	C12H25N5O3	1.5376833333333	-	pos	288.20222573244	0.003825225015622	56.1	62632-70-6
pos_28826	Lipoamide	metab_28825	B(ii)	0	46	HMDB0000962	M+Na-H2O	C8H15NOS2	1.5216833333333	C00248;_	pos	210.03669015782	0.16738840344857	43.5	940-69-2;_;
pos_28835	Ala Ile Arg	metab_28834	B(i)	86.8	0	-	M+H	C15H30N6O4	1.50565	-	pos	359.23918210517	0.0234503266122	55	-
pos_28839	Ceftibuten	metab_28838	B(ii)	0	55	HMDB0015485	M+H-H2O	C15H14N4O6S	1.5003333333333	C08117	pos	393.03300104013	0.02453453699518	46.6	97519-39-6
pos_28841	Taxiphyllin	metab_28840	B(ii)	0	65.8	HMDB0030704	M+CH3OH+Na	C14H17NO7	1.495	C01855	pos	366.11507118473	0.03695627839295	50.8	21401-21-8
pos_28852	Met Asn	metab_28851	B(i)	45.6	0	-	M+H	C9H17N3O4S	1.4737	-	pos	264.10058211437	0.034937035950718	45.4	-
pos_28853	Gly Val	metab_28852	B(i)	78	0	-	M+H	C7H14N2O3	1.46835	-	pos	175.10727190033	0.019813353345376	54.1	-
pos_28855	Leu Glu Asn	metab_28854	B(i)	51.1	0	-	M+H	C15H26N4O7	1.4577166666667	-	pos	375.18638185285	0.22880981298216	46	-
pos_28869	Leu Ala Gly	metab_28868	B(i)	39.1	0	-	M+H	C11H21N3O4	1.3883	-	pos	260.15978449017	0.010217648082705	46.5	-
pos_28872	Val Thr Asp	metab_28871	B(i)	93.5	0	-	M+H	C13H23N3O7	1.3723166666667	-	pos	334.16001358677	0.015186455293482	56.7	-
pos_28880	L-Tyrosine	metab_28879	B(i)	99.6	0	HMDB0000158;PW_C000103	M+H	C9H11NO3	1.3510166666667	C00082	pos	182.08060812605	0.007876277916443	59	60-18-4
pos_28881	Coumarin	metab_28880	B(i)	40.4	0	HMDB0001218	M+H	C9H6O2	1.3510166666667	C05851	pos	147.04366107739	0.013507679458306	47.1	91-64-5
pos_28884	4-Hydroxybenzaldehyde	metab_28883	B(i)	83.3	0	HMDB0011718	M+H	C7H6O2	1.3457333333333	C00633	pos	123.04375442158	0.011333272360236	55.7	123-08-0
pos_28888	Tyr Gln	metab_28887	B(i)	53.7	0	-	M+H	C14H19N3O5	1.3404	-	pos	310.13889282093	0.017202211849173	48.9	-
pos_28896	Leu-Asp	metab_28895	B(i)	64.2	0	HMDB0028925	M+H	C10H18N2O5	1.3348833333333	-	pos	247.12819782965	0.001936667309714	51.5	-
pos_28898	Morroniside	metab_28897	B(ii)	0	41.2	HMDB0254897	M+CH3OH+H	C17H26O11	1.3295333333333	C17000;_	pos	439.17729383572	0.013923476925439	45.5	25406-64-8;_
pos_28901	Coelenterazine	metab_28900	B(ii)	0	51.2	HMDB0250397	M+Na	C26H21N3O3	1.3241833333333	C15037;_	pos	446.15034690664	0.006839078525756	46.1	55779-48-1;_
pos_28905	Arg Ile Gln	metab_28904	B(i)	86.6	0	-	M+H	C17H33N7O5	1.3188833333333	-	pos	416.26056492678	0.032123348930404	54.9	-
pos_28909	Guanidylic Acid (Guanosine Monophosphate)	metab_28908	B(i)	89	0	-	M+H	C10H14N5O8P	1.31355	-	pos	364.06423465402	0.019905263810092	55.4	-
pos_28913	6'-Apiosyllotaustralin	metab_28912	B(ii)	0	59.7	HMDB0034207	M+ACN+H	C16H27NO10	1.3029	-	pos	435.19486307556	0.019322162686585	48.2	170033-25-7
pos_28922	Bicine	metab_28921	B(i)	39.4	0	HMDB0011727	M+ACN+H	C6H13NO4	1.2923	-	pos	205.11767799337	0.008741192520459	46.6	150-25-4

pos_28925	Val-Pro-Arg	metab_28924	B(i)	75.5	0	-	M+H	C16H30N6O4	1.28695	-	pos	371.23917503914	0.050160622226979	52.1	-
pos_28935	Uracil	metab_28934	B(i)	77.4	0	HMDB0000300;PW_C000204	M+H	C4H4N2O2	1.2815833333333	C00106	pos	113.03425195309	0.005764826208246	54.2	66-22-8
pos_28942	Arg-Pro	metab_28941	B(i)	80	0	HMDB0028717	M+H	C11H21N5O3	1.2708833333333	-	pos	272.17103643166	0.051329174619922	53.5	2418-69-1
pos_28944	Leu-His-Ser	metab_28943	B(i)	59.3	0	-	M+H-H2O	C15H25N5O5	1.2655333333333	-	pos	338.18169682309	0.013727439494621	49.7	-
pos_28949	Tyr Thr Asn	metab_28948	B(i)	59.5	0	-	M+H	C17H24N4O7	1.2601833333333	-	pos	397.17006702083	0.02041580044086	48.1	-
pos_28960	Thr-Asn-Pro	metab_28959	B(i)	58.5	0	-	M+H	C13H22N4O6	1.2546833333333	-	pos	331.16029986232	0.04850849312758	48.3	-
pos_28969	Gln-Gly-Arg	metab_28968	B(i)	75.3	0	-	M+H	C13H25N7O5	1.24935	-	pos	360.1980979	0.044423981256898	51.3	-
pos_28974	Biflavone	metab_28973	B(ii)	0	52.1	HMDB0249199	M+H	C30H18O4	1.2386666666667	-	pos	443.12589783034	0.21486032974257	44.5	-
pos_28976	Val-Asn-His	metab_28975	B(i)	41.7	0	-	M+H-H2O	C15H24N6O5	1.2386666666667	-	pos	351.17521231024	0.040055916703869	44.6	-
pos_28977	Pro Gln Ala	metab_28976	B(i)	79.6	0	-	M+H	C13H22N4O5	1.2386666666667	-	pos	315.16540173318	0.06153047759295	54.7	-
pos_28979	Arg-Phe-Tyr	metab_28978	B(i)	83.4	0	-	M+Na	C24H32N6O5	1.2333166666667	-	pos	507.23298832708	0.038122453284811	52.2	-
pos_28983	Threonylarginine	metab_28982	B(ii)	0	44.7	HMDB0029055	M+H	C10H21N5O4	1.228	-	pos	276.16593028662	0.007861982092979	47.1	13448-26-5
pos_28984	2-(1,5-Dimethyl-4-Nitropyrazol-3-Yl)-N,N-Dimethylethenamine	metab_28983	B(i)	54.9	0	-	M+NH4	C9H14N4O2	1.228	-	pos	228.14486001146	0.17974755733576	48.4	-
pos_28994	Dihydrostreptomycin	metab_28993	B(ii)	0	63	HMDB0251330	M+ACN+Na	C21H41N7O12	1.2067	C01023;_	pos	647.29754802875	0.16095406470444	50.9	128-46-1;_
pos_29000	Ile-Gln-Lys	metab_28999	B(i)	85.7	0	-	M+Na	C17H33N5O5	1.2013666666667	-	pos	410.23617651441	0.081063309154729	54.8	-
pos_29014	S-Allyl-L-Cysteine	metab_29013	B(i)	47.2	0	HMDB0034323	M+H	C6H11NO2S	1.1906833333333	C16759	pos	162.0578589	0.004569079430357	46.4	21593-77-1
pos_29019	Gly-Phe-Lys	metab_29018	B(i)	48.3	0	-	M+CH3OH+Na	C17H26N4O4	1.18535	-	pos	405.20815945116	0.070838165192611	45.2	-
pos_29020	Uridine Monophosphate (Ump)	metab_29019	B(i)	51.6	0	-	M+H	C9H13N2O9P	1.18535	-	pos	325.04217139395	0.03518250010498	48	-
pos_29023	Pyridoxine	metab_29022	B(i)	92.5	0	HMDB0000239;PW_C000161	M+H	C8H11NO3	1.18535	C00314	pos	170.08056449875	0.17208820715233	55.8	65-23-6
pos_29045	7-Methylguanine	metab_29044	B(i)	83.7	0	HMDB0000897	M+H	C6H7N5O	1.164	C02242	pos	166.07187938629	0.025646770990701	55.1	578-76-7
pos_29046	Hypoxanthine	metab_29045	B(i)	72.2	0	HMDB0000157;PW_C000102	M+H	C5H4N4O	1.164	C00262	pos	137.04542124611	0.02242967123495	52.6	68-94-0
pos_29051	Ala Lys Gly	metab_29050	B(i)	52.2	0	-	M+H	C11H22N4O4	1.15865	-	pos	275.17066995863	0.007673692944005	48.7	-
pos_29052	Pro-Glu	metab_29051	B(i)	82.3	0	HMDB0029016	M+H	C10H16N2O5	1.15865	-	pos	245.11253469815	0.072043542848968	53.8	-
pos_29057	Gln-Ile-Lys	metab_29056	B(i)	59.3	0	-	M+H	C17H33N5O5	1.1533	-	pos	388.25434833712	0.029084161537704	49.5	-
pos_29065	Glu Lys Ala Lys Ser	metab_29064	B(i)	45.9	0	-	M+H	C23H43N7O9	1.1479666666667	-	pos	562.3177941	0.044713724030959	47	-
pos_29067	Met Glu Arg	metab_29066	B(i)	50.1	0	-	M+H	C16H30N6O6S	1.1479666666667	-	pos	435.20084005463	0.054696810473694	47.1	-
pos_29069	Gln Val Gln	metab_29068	B(i)	92.3	0	-	M+H	C15H27N5O6	1.1479666666667	-	pos	374.20235892074	0.023431858073923	56.6	-
pos_29096	Arg Leu Asn	metab_29095	B(i)	85.8	0	-	M+H	C16H31N7O5	1.1372666666667	-	pos	402.24502572978	0.006554702274607	54.2	-
pos_29098	Hexaconazole	metab_29097	B(i)	35.2	0	-	M+H	C14H17Cl2N3	1.1372666666667	C18466	pos	314.08377420075	0.005570517830513	36	79983-71-4
pos_29100	Islatravir	metab_29099	B(ii)	0	56.2	HMDB0253596	M+H	C12H12FN5O3	1.1372666666667	-	pos	294.09981477693	0.045662104069048	49.1	-
pos_29103	Telmisartan	metab_29102	B(i)	35	0	HMDB0015101;PW_C009406	M+NH4	C33H30N4O2	1.1319666666667	C07710	pos	532.27081419806	0.11131739397239	42.6	144701-48-4
pos_29120	Leu-Asn-Asn	metab_29119	B(i)	64.8	0	-	M+H	C14H25N5O6	1.11595	-	pos	360.18669132772	0.043601374927092	49.4	-
pos_29121	N-(1,3-Benzodioxol-5-Ylmethyl)-1-(2,2-Dimethylpropanoyl)Piperidine-4-Carboxamide	metab_29120	B(i)	54.6	0	-	M+H	C19H26N2O4	1.11595	-	pos	347.19725947572	0.028459369970767	47.1	-
pos_29125	His Tyr	metab_29124	B(i)	95.8	0	-	M+H	C15H18N4O4	1.11595	-	pos	319.13927746429	0.038178876208698	54.5	-
pos_29133	Isonicotinamide	metab_29132	B(i)	49.9	0	HMDB0253663	M+H	C6H6N2O	1.11595	C02421	pos	123.05494599818	0.041930354842765	48.5	1453-82-3;_
pos_29138	Ile His Gln	metab_29137	B(i)	95.7	0	-	M+H	C17H28N6O5	1.1106333333333	-	pos	397.21852555172	0.042432076164923	56.5	-
pos_29149	Pro-Gln	metab_29148	B(i)	68.3	0	HMDB0029015	M+H	C10H17N3O4	1.1053166666667	-	pos	244.12851142216	0.017759693407032	51.8	-
pos_29160	6'-Hydroxybuspirone	metab_29159	B(ii)	0	50.8	HMDB0061110	M+CH3OH+Na	C21H31N5O3	1.1000333333333	-	pos	456.25513612478	0.026004738853916	45.3	-
pos_29196	Ser-Leu-Lys	metab_29195	B(i)	40.6	0	-	M+H	C15H30N4O5	1.0893	-	pos	347.22779157633	0.018225722434807	45.8	-
pos_29208	Alpha-Cyclodextrin	metab_29207	B(i)	83.3	0	HMDB0242514	M+H	C36H60O30	1.0839666666667	-	pos	973.32123106394	0.044392797558718	54.5	-
pos_29225	N-Hydroxycadaverine	metab_29224	B(ii)	0	41.2	HMDB0304426	M+CH3OH+Na	C5H15N2O+	1.0839666666667	-	pos	174.13446357223	0.048376123345181	46	-
pos_29228	Prazepam	metab_29227	B(i)	82	0	HMDB0015527	2M+H	C19H17ClN2O	1.0786666666667	C07366	pos	649.2164608	0.016819227761905	50.3	2955-38-6
pos_29239	Glycogen	metab_29238	B(ii)	0	58.5	HMDB0000757	M+Na-H2O	C24H42O21	1.0733666666667	C00182;C00369	pos	671.19849967376	0.03296140986831	48.8	9005-79-2;9005-25-8
pos_29243	8-Methylthiooctyl Glucosinolate	metab_29242	B(ii)	0	59.9	HMDB0304248	M+CH3OH+H	C16H30NO9S3	1.0733666666667	C17254;_	pos	509.14599807656	0.02203604721088	46.2	-;_

pos_29251	N-Alpha-Acetyllysine	metab_29250	B(i)	60.5	0	HMDB0000446	M+H	C8H16N2O3	1.0733666666667	C12989	pos	189.12283236718	0.007578024482044	51	1946-82-3
pos_29272	Gamma-Delta-Dioxovaleric Acid	metab_29271	B(ii)	0	51.3	HMDB0013233	M+H	C5H6O4	1.0521	C02800	pos	131.03351905051	0.033226351981638	48.6	-
pos_29284	O-Acetyl-L-Homoserine	metab_29283	B(i)	35.5	0	-	M+H	C6H11NO4	1.0415	C01077	pos	162.07560960787	0.0276113714954	45.3	7540-67-2
pos_29301	Glutathionyl-3-Hydroxykynurenine Glucoside	metab_29300	B(ii)	0	40.8	HMDB0062471	M+H	C26H36N4O15	1.0255166666667	-	pos	677.1951677	0.010233332408702	43.7	244286-41-7
pos_29302	Rose Oxide (Cis)	metab_29301	B(ii)	0	57.3	HMDB0304731	M+H-2H2O	C21H24O9	1.0255166666667	C10288	pos	385.126481	0.029015655995564	47.8	-
pos_29304	S-Ethyl-L-Cysteine	metab_29303	B(ii)	0	70.5	HMDB0257396	M+H	C5H11NO2S	1.0255166666667	-	pos	150.05784681129	0.013226472869223	51.9	-
pos_29310	Pinobanksin 5-[Galactosyl-(1->4)-Glucoside]	metab_29309	B(ii)	0	47.1	HMDB0037506	M+	C27H32O15	1.0202166666667	C09826	pos	596.16895778785	0.059692969740944	44.2	91925-95-0
pos_29311	L-Glutathione	metab_29310	B(i)	99	0	HMDB0000125	M+H	C10H17N3O6S	1.0202166666667	C00051	pos	308.0901971	0.024448805487902	57.9	70-18-8
pos_29315	Methionine Sulfoxide	metab_29314	B(i)	35.2	0	HMDB0002005;PW_C001342	M+H-H2O	C5H11NO3S	1.0202166666667	C02989;_	pos	148.04225045529	0.022238527141966	45.4	3226-65-1;62697-73-8
pos_29331	3'-Deoxythymidine	metab_29330	B(ii)	0	41.5	HMDB0246094	M+H	C10H14N2O4	1.0148833333333	-	pos	227.10197840316	0.002619114203376	46.8	-
pos_29357	N(6)-Hydroxymethyladenosine	metab_29356	B(i)	54.5	0	-	M+H	C11H15N5O5	1.00955	-	pos	298.11374880751	0.10348826233101	47.9	-
pos_29359	(3-Ethyl-3-Hydroxy-7-Azaspiro[3.5]Nonan-7-Yl)-Thiophen-2-Ylmethanone	metab_29358	B(i)	73.8	0	-	M+H	C15H21NO2S	1.00955	-	pos	280.1383269	0.02339498923718	51.1	-
pos_29364	His Ala	metab_29363	B(i)	46.7	0	-	M+H	C9H14N4O3	1.00955	-	pos	227.11325106721	0.008333740170083	47.4	-
pos_29372	L-Theanine	metab_29371	B(i)	91	0	HMDB0034365	M+H	C7H14N2O3	1.00955	C01047	pos	175.10753851623	0.006720145805582	56.7	3081-61-6
pos_29375	Glabrin A	metab_29374	B(ii)	0	55	HMDB0301777	M+H-H2O	C7H13NO4	1.00955	-	pos	158.08071568814	0.007758453073718	49.5	162157-00-8
pos_29380	Nicotinic Acid	metab_29379	B(i)	57.6	0	HMDB0001488;PW_C001145	M+H	C6H5NO2	1.00955	C00253	pos	124.038946	0.039133182115462	50	59-67-6
pos_29394	Val-Gln-His	metab_29393	B(i)	54.6	0	-	M+H	C16H26N6O5	1.0041166666667	-	pos	383.20262013556	0.053251864140793	48.5	-
pos_29403	Arg Met	metab_29402	B(i)	52.2	0	-	M+H	C11H23N5O3S	1.0041166666667	-	pos	306.15851721156	0.040151170861104	47.5	-
pos_29418	Val Thr	metab_29417	B(i)	47.2	0	-	M+H	C9H18N2O4	1.0041166666667	-	pos	219.13336095193	0.048465965245098	41.1	-
pos_29442	Val Glu Arg	metab_29441	B(i)	88.3	0	-	M+H	C16H30N6O6	0.99883333333333	-	pos	403.22881472706	0.02309158292397	53.3	-
pos_29448	Gly-Asn-Thr	metab_29447	B(i)	38.9	0	-	M+H	C10H18N4O6	0.99883333333333	-	pos	291.12900994347	0.031801148831487	44.8	-
pos_29466	Glu-Gln-Lys	metab_29465	B(i)	68.1	0	-	M+H-H2O	C16H29N5O7	0.99356666666667	-	pos	386.20213849128	0.1110984121982	51	-
pos_29483	Pgg-Glucan	metab_29482	B(ii)	0	67.8	HMDB0256373	M+H	C36H62O31	0.98828333333333	-	pos	991.33156350992	0.033846602232742	51.8	-
pos_29493	Thr-Pro-Arg	metab_29492	B(i)	79.8	0	-	M+H	C15H28N6O5	0.98828333333333	-	pos	373.21819610591	0.079372752037241	51.6	-
pos_29499	Cytidine	metab_29498	B(i)	92.1	0	HMDB0000089;HMDB0248553;PW_C000061	M+H	C9H13N3O5	0.98828333333333	C00475;_	pos	244.09211538262	0.006187196790170	56.4	65-46-3;_
pos_29518	Gly Leu Arg	metab_29517	B(i)	66.6	0	-	M+H	C14H28N6O4	0.98298333333333	-	pos	345.22355480411	0.040778754176769	47.6	-
pos_29528	Cycloleucine	metab_29527	B(i)	74.2	0	HMDB0062225	M+H	C6H11NO2	0.98298333333333	C03969	pos	130.08587741718	0.055725741941014	53.3	52-52-8
pos_29570	Tyr-Lys	metab_29569	B(i)	65.1	0	HMDB0029110	M+H	C15H23N3O4	0.96705	-	pos	310.17517247219	0.028256326325053	49.9	-
pos_29584	L-Valine	metab_29583	B(i)	99.3	0	HMDB0000883;PW_C000704	M+H	C5H11NO2	0.9617	C00183	pos	118.08590021121	0.002878160747728	57.9	72-18-4
pos_29594	His-Ile	metab_29593	B(i)	40.9	0	HMDB0028888	M+Na	C12H20N4O3	0.9564	-	pos	291.14187399594	0.091734816627115	45.2	129050-48-2
pos_29599	3-Methylbut-2-Enoyl-Coa	metab_29598	B(ii)	0	60.1	HMDB0301419	M+CH3OH+H	C26H42N7O17P3S	0.95105	C03069;_	pos	882.19041326554	0.12164703893277	48.8	-;_;
pos_29610	Cay10415	metab_29609	B(i)	49.2	0	-	M+ACN+Na	C19H18N2O4S	0.94571666666667	-	pos	434.11680368356	0.068528944834808	45	-
pos_29633	Gln Asn Ala	metab_29632	B(i)	93.9	0	-	M+H	C12H21N5O6	0.92971666666667	-	pos	332.15555310139	0.16665304414866	55.1	-
pos_29641	Trp-Leu-Oh	metab_29640	B(i)	41.7	0	-	M+H	C23H25N3O6	0.92438333333333	-	pos	440.17934861322	0.13122516571921	42.5	-
pos_29648	Arctignan C	metab_29647	B(ii)	0	65.4	HMDB0302299	M+ACN+H	C30H32O10	0.91378333333333	-	pos	594.23469456326	0.04271725883128	47.7	-
pos_29650	Pro Gly Gln	metab_29649	B(i)	45	0	-	M+H	C12H20N4O5	0.91378333333333	-	pos	301.1499069	0.091024228373305	46	-
pos_29658	Ser Thr Glu	metab_29657	B(i)	78.8	0	-	M+H	C12H21N3O8	0.9031	-	pos	336.13915383217	0.060350515953875	52.4	-
pos_29662	Docarpamine	metab_29661	B(ii)	0	57	HMDB0251553	M+ACN+H	C21H30N2O8S	0.89778333333333	-	pos	512.20609903636	0.122490409	46.1	-
pos_29663	Adimolol	metab_29662	B(ii)	0	60.9	HMDB0248012	M+Na-H2O	C25H29N3O3	0.89778333333333	-	pos	424.20278782251	0.22126059743265	45.7	-
pos_29680	Gladiatoside A2	metab_29679	B(ii)	0	60.8	HMDB0036260	M+H-H2O	C47H56O26	0.87651666666667	-	pos	1019.3099484017	0.14869777688875	44.7	-
pos_29682	Trp-Met-His	metab_29681	B(i)	42	0	-	M+NH4	C22H28N6O4S	0.87651666666667	-	pos	490.22420918127	0.096278999143715	42.7	-
pos_29690	S-Lactoylglutathione	metab_29689	B(ii)	0	41.6	HMDB0001066	M+ACN+Na	C13H21N3O8S	0.85523333333333	C03451	pos	443.12201888796	0.038653790099356	44.3	25138-66-3
pos_29699	Ala Asn Glu Arg	metab_29698	B(i)	42	0	-	M+H	C18H32N8O8	0.83398333333333	-	pos	489.2399116	0.085823950434017	44	-
pos_29733	Thiomorpholine 3-Carboxylate	metab_29732	B(ii)	0	56.9	HMDB0059611	M+H	C5H9NO2S	0.78623333333333	C03901	pos	148.04224798859	0.024748904719305	48.9	20960-92-3;_

pos_29761	D-(+)-Turanose	metab_29760	B(i)	64.5	0	HMDB0011740	M+NH4	C12H22O11	0.7649	C19636	pos	360.1489699	0.064707794721773	49.4	547-25-1
pos_29808	Val Ala	metab_29807	B(i)	54	0	-	M+H	C8H16N2O3	0.73833333333333	-	pos	189.12285302657	0.0217443061757	49.4	-
pos_29809	Norvaline	metab_29808	B(i)	97.3	0	HMDB0013716	M+H	C5H11NO2	0.73833333333333	C01799	pos	118.08590976063	0.01482856919288	57.6	760-78-1
pos_29815	N-(1-Deoxy-1-Fructosyl)Valine	metab_29814	B(ii)	0	66	HMDB0037844	M+H	C11H21NO7	0.73305	-	pos	280.13826680919	0.030956969951059	50.8	-
pos_29861	Cascarillin	metab_29860	B(ii)	0	42.2	HMDB0036836	M+H	C22H32O7	0.71718333333333	C09071	pos	409.21811864907	0.021448203288657	43.2	10118-56-6
pos_29866	Ala Leu Arg	metab_29865	B(i)	78.5	0	-	M+H	C15H30N6O4	0.71718333333333	-	pos	359.23917603052	0.038354409850767	52.7	-
pos_29869	D[-Arg-2]Kyotorphan	metab_29868	B(i)	75.9	0	-	M+H	C15H23N5O4	0.71718333333333	-	pos	338.18164791538	0.009255288892678	53.2	-
pos_29877	Isoleucyl-Arginine	metab_29876	B(ii)	0	71.3	HMDB0028901	M+H	C12H25N5O3	0.71718333333333	-	pos	288.2021916	0.011858362987574	52.7	-
pos_29890	Pyroglutamylproline	metab_29889	B(i)	66.8	0	-	M+H	C10H14N2O4	0.71718333333333	-	pos	227.10216593615	0.007700470635479	52	-
pos_29903	Glu Phe Thr Pro Ala	metab_29902	B(i)	35.5	0	-	M+H	C26H37N5O9	0.71191666666667	-	pos	564.2603312	0.048492190596979	41.8	-
pos_29909	Val Gln Ala Arg	metab_29908	B(i)	53.7	0	-	M+H	C19H36N8O6	0.71191666666667	-	pos	473.28171488967	0.030996729265143	48.9	-
pos_29923	Ile Lys Gln	metab_29922	B(i)	50.5	0	-	M+H	C17H33N5O5	0.71191666666667	-	pos	388.25441852827	0.008025094033897	47	-
pos_29925	Leu-Asn-His	metab_29924	B(i)	60.6	0	-	M+H	C16H26N6O5	0.71191666666667	-	pos	383.20267249569	0.051945534715529	49.7	-
pos_29927	Leu Ser Arg	metab_29926	B(i)	84.8	0	-	M+H	C15H30N6O5	0.71191666666667	-	pos	375.23397020982	0.015540848462928	55.2	-
pos_29941	Pro Asn Gly	metab_29940	B(i)	38.8	0	-	M+NH4	C11H18N4O5	0.71191666666667	-	pos	304.16045519583	0.035011957379369	44.5	-
pos_29955	His-Leu	metab_29954	B(i)	70.2	0	HMDB0028889;HMDB0253179;HMDB0028889	M+H	C12H20N4O3	0.71191666666667	C05010	pos	269.16015924168	0.027341413809846	52.4	7763-65-7
pos_29976	N-Acetyl-L-Arginine	metab_29975	B(i)	57.5	0	HMDB0004620	M+H	C8H16N4O3	0.71191666666667	-	pos	217.12859554314	0.045106717940963	48.9	155-84-0
pos_29990	L-Lysine	metab_29989	B(i)	84.1	0	HMDB0000182;PW_C000118	M+H	C6H14N2O2	0.71191666666667	C00047	pos	147.11237917926	0.026704571660874	54.8	56-87-1
pos_29994	Piperidine-4-Carboxamide	metab_29993	B(i)	43	0	-	M+H	C6H12N2O	0.71191666666667	-	pos	129.10187115862	0.008536912876813	46.7	-
pos_29995	Isocytosine	metab_29994	B(i)	43.6	0	-	M+H	C4H5N3O	0.71191666666667	-	pos	112.05022516379	0.037025386035005	47.4	-
pos_29996	2-Amino-3-Methyl-1-Butanol	metab_29995	B(i)	37.4	0	HMDB0244984	M+H-H2O	C5H13NO	0.71191666666667	-	pos	86.09618823	0.018111976114767	46.6	-
pos_30002	Asn Thr Val Arg	metab_30001	B(i)	46.5	0	-	M+H	C19H36N8O7	0.70665	-	pos	489.27712477808	0.058311720210133	46	-
pos_30012	Gln-Val-Lys	metab_30011	B(i)	63.6	0	-	M+H	C16H31N5O5	0.70665	-	pos	374.23862174376	0.072333942527738	48.3	-
pos_30014	Ser Val Arg	metab_30013	B(i)	90.9	0	-	M+H	C14H28N6O5	0.70665	-	pos	361.2183189	0.020424169886789	56.3	-
pos_30054	Leu Lys	metab_30053	B(i)	85.8	0	-	M+H	C12H25N3O3	0.70138333333333	-	pos	260.19614133909	0.019298798341943	55.7	-
pos_30061	Arg Val Gln	metab_30060	B(i)	77.6	0	-	M+H	C16H31N7O5	0.69613333333333	-	pos	402.24491300786	0.065575661409086	52.3	-
pos_30081	Arg-His-Ile	metab_30080	B(i)	64.9	0	-	M+H	C18H32N8O4	0.69086666666667	-	pos	425.26079632617	0.020167870403835	50.3	-
pos_30087	His Arg	metab_30086	B(i)	51.8	0	-	M+H	C12H21N7O3	0.69086666666667	-	pos	312.17669270543	0.021795831540608	46.9	-
pos_30093	Pro-Thr	metab_30092	B(i)	51.5	0	HMDB0029027	M+H	C9H16N2O4	0.69086666666667	-	pos	217.11774064619	0.045973749035087	48.5	-
pos_30115	Deoxyloganic Acid	metab_30114	B(ii)	0	44.8	HMDB0037028	M+NH4	C16H24O9	0.68558333333333	C11647	pos	378.17403999681	0.11036770575682	44.9	92842-56-3
pos_30123	Thr Glu	metab_30122	B(i)	84.1	0	-	M+H	C9H16N2O6	0.68558333333333	-	pos	249.10736606673	0.049208372485151	55.1	-
pos_30141	14Alpha-Hydroxypaxilline	metab_30140	B(ii)	0	47.3	HMDB0040978	M+Na	C27H33NO5	0.68025	-	pos	474.22925707621	0.079928901899937	43.8	157530-30-8
pos_30143	Niazicinin	metab_30142	B(ii)	0	46.4	HMDB0031947	M+CH3OH+Na	C17H23NO8	0.68025	-	pos	424.15491040804	0.066975729130795	44.7	163812-19-9
pos_30151	Val Ala His	metab_30150	B(i)	95.8	0	-	M+H	C14H23N5O4	0.68025	-	pos	326.18126180956	0.04571015717393	57.7	-
pos_30152	Val-Gly-Lys	metab_30151	B(i)	38.3	0	-	M+H	C13H26N4O4	0.68025	-	pos	303.20211303083	0.016283977585043	45.1	-
pos_30163	Glycerol	metab_30162	B(i)	94.7	0	HMDB0000131;PW_C000085	M+H	C3H8O3	0.68025	C00116	pos	93.054367214128	0.02102995090759	57.5	56-81-5
pos_30171	Thr Gln Gln	metab_30170	B(i)	94.9	0	-	M+H	C14H25N5O7	0.67498333333333	-	pos	376.18148583291	0.073538022772761	56.1	-
pos_30185	3-Hydroxypropanal	metab_30184	B(ii)	0	86.1	HMDB0003453	M+H	C3H6O2	0.67498333333333	C00969	pos	75.043827551559	0.040350811633054	55.8	2134-29-4
pos_30192	Val-Ala-Lys	metab_30191	B(i)	73.5	0	-	M+H	C14H28N4O4	0.66965	-	pos	317.21740591517	0.03870805775933	52.2	-
pos_30196	Imidazole Lactic Acid	metab_30195	B(i)	98.8	0	HMDB0253418	M+H	C6H8N2O3	0.66965	C05568	pos	157.06026583794	0.067498525717848	58.2	-
pos_30198	L-Proline	metab_30197	B(i)	95.8	0	HMDB0000162;PW_C000106	M+H	C5H9NO2	0.66965	C00148	pos	116.07023756128	0.024969138272797	57.3	147-85-3
pos_30201	Fagopyritol B3	metab_30200	B(ii)	0	44.2	HMDB0035323	M+H	C24H42O21	0.66438333333333	-	pos	667.22684785898	0.029260194298193	47.1	208708-11-6
pos_30207	Leu-Lys-His	metab_30206	B(i)	85	0	-	M+H	C18H32N6O4	0.66438333333333	-	pos	397.25447696099	0.053344066664122	54.3	-
pos_30213	Ser His	metab_30212	B(i)	87.9	0	-	M+H	C9H14N4O4	0.66438333333333	-	pos	243.10809981696	0.033323225498752	55.8	-
pos_30226	Ala Glu Asn Arg	metab_30225	B(i)	68.9	0	-	M+H	C18H32N8O8	0.6591	-	pos	489.23935071881	0.055118486638808	49.9	-
pos_30229	Asp Arg Glu	metab_30228	B(i)	69.4	0	-	M+H	C15H26N6O8	0.6591	-	pos	419.18729628646	0.034842526946481	51	-

pos_30243	Ala-Gln	metab_30242	B(i)	84.2	0	HMDB0028685	M+H	C8H15N3O4	0.6591	-	pos	218.11291580227	0.04001100867968	55.3	39537-23-0
pos_30259	N6-Methyl-2'-Deoxyadenosine	metab_30258	B(ii)	0	62.9	HMDB0255300	M+H	C11H15N5O3	0.65381666666667	_;C03795	pos	266.12272119718	0.01380051667911	49.6	2002-35-9;_
pos_30263	Ser Glu	metab_30262	B(i)	84.3	0	-	M+H	C8H14N2O6	0.65381666666667	-	pos	235.09178958922	0.022679511952276	55.7	-
pos_30283	2-Nitrophenyl A-D-Galactopyranoside	metab_30282	B(ii)	0	49.7	HMDB0244855	M+H-H2O	C12H15NO8	0.64855	-	pos	284.07889087756	0.10815079798736	45.9	-
pos_30284	Asn Glu	metab_30283	B(i)	82.6	0	-	M+H	C9H15N3O6	0.64855	-	pos	262.10262153273	0.03018284740419	54.5	-
pos_30286	N-(2-Furoyl)Glycine	metab_30285	B(i)	49.1	0	HMDB0000439	M+NH4	C7H7NO4	0.64855	-	pos	187.07072546339	0.091224444911066	48.4	5657-19-2
pos_30299	Thr Gln	metab_30298	B(i)	82.8	0	-	M+H	C9H17N3O5	0.64326666666667	-	pos	248.12329613844	0.13240475644379	54.2	-
pos_30305	D-Sorbitol	metab_30304	B(i)	90.7	0	HMDB0000247	M+H	C6H14O6	0.64326666666667	C00794	pos	183.08568891977	0.21877849232945	55.9	50-70-4
pos_30312	Neuraminic Acid	metab_30311	B(ii)	0	59.7	HMDB0000830	M+ACN+H	C9H17NO8	0.63793333333333	C06469	pos	309.12832629822	0.031430536859348	48.8	114-04-5
pos_30313	(2S,3R,4S,5S,6R)-2-(3,5-Dihydroxyphenoxy)-6-(Hydroxymethyl)Oxane-3,4,5-Triol	metab_30312	B(i)	39.2	0	-	M+H	C12H16O8	0.63793333333333	-	pos	289.09092741419	0.052208916362398	46	-
pos_30315	Phenylglucuronide	metab_30314	B(ii)	0	60.8	HMDB0059806	M+H	C12H14O7	0.63793333333333	-	pos	271.08048883477	0.044701473317319	50	-
pos_30321	N5-Formyl-N5-Hydroxy-L-Ornithine	metab_30320	B(ii)	0	43.1	HMDB0304431	M+H-H2O	C6H12N2O4	0.63793333333333	-	pos	159.07591590039	0.024121782841869	46.4	-
pos_30322	Phloroglucinol	metab_30321	B(i)	79.5	0	HMDB0013675	M+H	C6H6O3	0.63793333333333	C02183	pos	127.03858963363	0.14441658393327	54.9	108-73-6
pos_30324	2-Oxobutanoic Acid	metab_30323	B(i)	58.9	0	HMDB0000005	M+H-H2O	C4H6O3	0.63793333333333	C00109	pos	85.028150274541	0.091996558413383	50.9	600-18-0
pos_30326	Muramic Acid	metab_30325	B(i)	75.3	0	HMDB0003254;HMDB0248237	M+H	C9H17NO7	0.63263333333333	C06470;_	pos	252.10702368542	0.012337396397248	52.1	1114-41-6;_
pos_30336	Gln Gln	metab_30335	B(i)	96.2	0	-	M+H	C10H18N4O5	0.62203333333333	-	pos	275.13413512431	0.015632427484197	57.5	-
pos_30347	Scopoletin	metab_30346	B(i)	37.6	0	HMDB0034344	M+CH3OH+Na	C10H8O4	0.61676666666667	C01752	pos	247.0569451	0.02262154016528	44.7	92-61-5
pos_30356	T-4-Aminocrotonic Acid	metab_30355	B(i)	75.2	0	-	M+H	C4H7NO2	0.6115	-	pos	102.05465751542	0.019370038015452	53.4	-
pos_30357	2-Pyrrolidinone	metab_30356	B(i)	66.5	0	HMDB0002039	M+H	C4H7NO	0.6115	C11118	pos	86.059792533482	0.051460370287321	51.9	616-45-5
pos_30358	5,6-Dihydro-1,2,9,10-Tetramethoxy-6-Methyl-7-Benzoyl-4H-Dibenzo[De,G]Quinoline	metab_30357	B(i)	64	0	-	M+NH4	C28H27NO5	0.60623333333333	-	pos	475.22439524202	0.02008814510497	47.9	-
pos_30363	Gly Asn Lys	metab_30362	B(i)	54.9	0	-	M+H	C12H23N5O5	0.60623333333333	-	pos	318.17638876286	0.044205879363403	47.8	-
pos_30377	L-Threonine	metab_30376	B(i)	98.6	0	HMDB0000167;PW_C000109	M+H	C4H9NO3	0.60623333333333	C00188	pos	120.06515686253	0.037937845583247	58	72-19-5
pos_30383	Liquiritin	metab_30382	B(ii)	0	50.7	HMDB0029520	M+H-2H2O	C21H22O9	0.60096666666667	C16989;_	pos	383.11481152076	0.038185609480093	46.4	551-15-5;
pos_30392	N4-Acetylcytidine	metab_30391	B(ii)	0	42	HMDB0005923	M+Na-H2O	C11H15N3O6	0.60096666666667	C22293;_	pos	290.07487832787	0.060381276464583	45.6	3768-18-1
pos_30394	Choline Glycerophosphate	metab_30393	B(i)	98.1	0	HMDB0000086	M+H	C8H20NO6P	0.60096666666667	C00670	pos	258.10918978723	0.032135723584348	58.5	28319-77-9
pos_30397	Thr-Asn	metab_30396	B(i)	73.8	0	HMDB0029056	M+H	C8H15N3O5	0.60096666666667	-	pos	234.10771409198	0.046633763079576	53	330601-56-4
pos_30408	Aspartic Acid Methyl Ester	metab_30407	B(i)	49.8	0	-	M+H	C5H10N2O3	0.60096666666667	-	pos	147.07596152005	0.006154483104499	48.7	-
pos_30409	D-Pyroglutamic Acid	metab_30408	B(i)	95.8	0	-	M+H	C5H7NO3	0.60096666666667	C02237	pos	130.04946035173	0.012739058081121	58	4042-36-8
pos_30411	L-Alanine	metab_30410	B(i)	82.2	0	HMDB0000161;PW_C000105	M+H	C3H7NO2	0.60096666666667	C00041	pos	90.054690605334	0.037415053627819	55.3	56-41-7
pos_30412	Dimethylformamide	metab_30411	B(i)	48.1	0	HMDB0001888	M+H	C3H7NO	0.60096666666667	C03134	pos	74.059826936316	0.029772823990204	48.2	1968/12/2
pos_30421	Ethyl 4-[[1-(4-Methoxyphenyl)Cyclopentyl]Methylamino]-4-Oxobutanoate	metab_30420	B(i)	35	0	-	M+H	C19H27NO4	0.5957	-	pos	334.20220151558	0.13165756572301	43	-
pos_30422	Ala His Ser	metab_30421	B(i)	77.2	0	-	M+H	C12H19N5O5	0.5957	-	pos	314.14496591848	0.061460365260752	52.1	-
pos_30429	L-Erythro-4-Hydroxyarginine	metab_30428	B(ii)	0	50.9	HMDB0034326	M+ACN+H	C6H14N4O3	0.5957	C08284	pos	232.13972711589	0.016437071564114	45.8	61370-10-3
pos_30437	Glyoxylic Acid	metab_30436	B(i)	38.6	0	HMDB0000119;PW_C000075	M+ACN+H	C2H2O3	0.5957	C00048	pos	116.03387393378	0.055358716335151	46.1	298-12-4
pos_30439	L-Glycine	metab_30438	B(i)	35.1	0	-	M+H	C2H5NO2	0.5957	C00037	pos	76.03907002	0.036721320927617	45.7	-
pos_30443	S-Adenosylmethionine	metab_30442	B(i)	99.6	0	HMDB0001185;PW_C000921	M+H	C15H22N6O5S	0.59043333333333	C00019	pos	399.14314613207	0.054041476043369	57.9	29908-03-0;485-80-3
pos_30444	Gln Arg Ala	metab_30443	B(i)	64.6	0	-	M+H	C14H27N7O5	0.59043333333333	-	pos	374.21347790148	0.04478967788519	50.9	-
pos_30446	N2-Fructopyranosylarginine	metab_30445	B(ii)	0	56.2	HMDB0041541	M+H	C12H24N4O7	0.59043333333333	-	pos	337.170676	0.06696249900139	49.4	162966-07-6
pos_30449	Asp Arg	metab_30448	B(i)	86.9	0	-	M+H	C10H19N5O5	0.59043333333333	-	pos	290.14496560244	0.04362741695313	55.6	-
pos_30473	Ser Asn Thr Arg	metab_30472	B(i)	70.6	0	-	M+H	C17H32N8O8	0.58515	-	pos	477.24017473871	0.041660343475555	51.7	-
pos_30475	Arg Ser Gln	metab_30474	B(i)	64.8	0	-	M+H	C14H27N7O6	0.58515	-	pos	390.20869632792	0.056812054912401	49.2	-
pos_30481	Pro-Asp-Ser	metab_30480	B(i)	55.3	0	-	M+H	C12H19N3O7	0.58515	-	pos	318.12863521709	0.081077769217041	48.6	-
pos_30482	Glu Arg	metab_30481	B(i)	90.8	0	-	M+H	C11H21N5O5	0.58515	-	pos	304.16040719763	0.033259712436112	56	-

pos_30483	Arg Gln	metab_30482	B(i)	70.3	0	-	M+H	C11H22N6O4	0.58515	-	pos	303.17664825531	0.031954524735113	51	-
pos_30484	Olpadronic Acid	metab_30483	B(ii)	0	41.3	HMDB0255958	M+CH3OH+H	C5H15NO7P2	0.58515	-	pos	296.0650918	0.038598911072076	46.9	-
pos_30487	Beta-Alanyl-L-Arginine	metab_30486	B(ii)	0	62.7	HMDB0060441	M+H	C9H19N5O3	0.58515	C05340	pos	246.15535615074	0.024527300968367	50.7	-;_
pos_30521	2-Amino-4-[Carbamimidoyl(Methyl)Amino]Butanoic Acid	metab_30520	B(ii)	0	55.5	HMDB0255374	M+H	C6H14N4O2	0.579866666666667	-	pos	175.11834706031	0.015385943090165	48.9	-
pos_30522	(E)-Ethenoxyimino-Oxido-Pyrrolidin-1-Ylazanium	metab_30521	B(i)	35.8	0	-	M+H	C6H11N3O2	0.579866666666667	-	pos	158.09191005454	0.03291082909714	45.5	-
pos_30523	L-Histidine	metab_30522	B(i)	99.2	0	HMDB0000177;PW_C000116	M+H	C6H9N3O2	0.579866666666667	C00135	pos	156.07627115415	0.014702111742945	58.6	71-00-1
pos_30538	N-Cyclopentyl-6-Imidazol-1-Ylpyrimidin-4-Amine	metab_30537	B(i)	60.1	0	-	M+H	C12H15N5	0.574616666666667	-	pos	230.14232568713	0.040362528115716	48.6	-
pos_30564	L-Histidine Trimethylbetaine	metab_30563	B(ii)	0	50.6	HMDB0029422	M+H	C9H15N3O2	0.569316666666667	C05575	pos	198.12314424789	0.009376195596978	48.7	534-30-5
pos_30636	S-Adenosylmethioninamine	metab_30635	B(ii)	0	65.9	HMDB0000988	M+	C14H23N6O3S +	0.558766666666667	C01137	pos	355.15374393619	0.14531869709779	49.9	-;22365-13-5
pos_30648	Asp His	metab_30647	B(i)	77.6	0	-	M+	C10H14N4O5	0.558766666666667	-	pos	270.09555417038	0.11531245402164	52.3	-
pos_30678	His-Ala	metab_30677	B(i)	83.1	0	HMDB0028878	M+H	C9H14N4O3	0.553416666666667	-	pos	227.11335954117	0.026224097629111	54.6	16874-75-2
pos_30686	Arg Ala Gln Lys	metab_30685	B(i)	53.8	0	-	M+H	C20H39N9O6	0.548083333333333	-	pos	502.30813488485	0.1269632313687	46.7	-
pos_30693	His Ser	metab_30692	B(i)	74.7	0	-	M+H	C9H14N4O4	0.548083333333333	-	pos	243.10806252711	0.058749979602873	52.9	-
pos_30714	Glu-Lys	metab_30713	B(i)	38.9	0	HMDB0004207	M+H-H2O	C11H21N3O5	0.5428	-	pos	258.14411968697	0.037638960781297	44.5	5891-53-2
pos_30719	Lys Ala	metab_30718	B(i)	82.7	0	-	M+H	C9H19N3O3	0.5428	-	pos	218.14930062633	0.03465774485495	54.9	-
pos_30727	Pipecolic Acid	metab_30726	B(i)	75.6	0	HMDB0000070;HMDB0000716;HMDB0000070	M+H	C6H11NO2	0.5428	C00408	pos	130.08587114919	0.016126154505761	53.8	3105-95-1;535-75-1
pos_30728	1-Aminocyclobutane Carboxylic Acid	metab_30727	B(i)	84.4	0	-	M+H	C5H9NO2	0.5428	-	pos	116.07027586087	0.031986425543988	55.7	-
pos_30732	Lys Ala Arg	metab_30731	B(i)	76.5	0	-	M+H	C15H31N7O4	0.537483333333333	-	pos	374.24996250016	0.23436003070368	51.4	-
pos_30959	Spermidine	metab_30958	B(i)	93.1	0	HMDB0001257;PW_C000971	M+H	C7H19N3	0.484166666666667	C00315	pos	146.16468073434	0.017996670604805	56.2	124-20-9
pos_31077	Erucic Acid	metab_31076	B(ii)	0	60.7	HMDB0002068	M+H-H2O, M+, M+H-2H2O	C22H42O2	7.0124666666667	C08316	pos	321.31439052727	0.029276617686728	49.8	112-86-7
pos_31115	Rebaudioside B	metab_31114	B(ii)	0	44.9	HMDB0034949	M+H-H2O, M+Na-H2O	C38H60O18	4.56695	-	pos	787.38096275365	0.012581388254496	45.5	58543-17-2;
pos_31135	Pipercyclobutanamide B	metab_31134	B(ii)	0	42.3	HMDB0036361	M+, M+Na	C36H40N2O6	5.7728166666667	-	pos	619.28354362812	0.044553949785604	42.4	-
pos_31163	Rifapentina	metab_31162	B(ii)	0	46.6	HMDB0257227	M+, M+Na	C47H64N4O12	5.9161833333333	-	pos	876.45857207007	0.012467357230838	45.8	-
pos_31221	Cassiaside B2	metab_31220	B(ii)	0	60	HMDB0032833	M+H-H2O, M+	C39H52O25	1.0893	C17674;_	pos	903.28557009736	0.045083275804023	46	-;_
neg_37	Mycophenolate Mofetil	metab_31264	B(i)	80.8	0	HMDB0014826;HMDB0255488;PW_C009165	M+FA-H	C23H31NO7	0.4573	C07908;_	neg	478.20418076986	0.063706199063131	51.7	128794-94-5;_
neg_46	Lys-Ala-Lys	metab_31273	B(i)	77.8	0	-	M-H	C15H31N5O4	0.4626	-	neg	344.23037862629	0.06370915535777	53.6	-
neg_90	Furaquinocin C	metab_31317	B(i)	55.6	0	-	M+FA-H	C22H26O5	0.483816666666667	-	neg	415.17985108058	0.19042748263754	48.3	-
neg_91	Fipronil	metab_31318	B(ii)	0	48.5	HMDB0252265	M+Cl	C12H4Cl2F6N4OS	0.483816666666667	C11099	neg	470.90435082251	0.02975568612392	41.1	120068-37-3;_
neg_119	Glycyl-Histidyl-Arginyl-Proline	metab_31346	B(ii)	0	66.8	HMDB0252877	M+Na-2H	C19H31N9O5	0.49445	-	neg	486.21742063465	0.075792405914878	51.5	-
neg_165	Asp-Lys-His	metab_31392	B(i)	39.6	0	-	M+FA-H	C16H26N6O6	0.50505	-	neg	443.18599645572	0.078003927083972	45.7	-
neg_180	Arg-Ala-Lys	metab_31407	B(i)	72	0	-	M-H	C15H31N7O4	0.51035	-	neg	372.23667107227	0.071617038149549	52.8	-
neg_214	Methyl 7-Deshydroxypyrogallin-4-Carboxylate	metab_31441	B(i)	45.9	0	-	M-H	C13H10O6	0.526283333333333	-	neg	261.04065403594	0.015882099176693	46.4	-
neg_216	Lys-Asn-Asn	metab_31443	B(i)	71.1	0	-	M-H	C14H26N6O6	0.526283333333333	-	neg	373.18426071176	0.026004941058271	53	-
neg_237	Asn-Gly-Lys	metab_31464	B(i)	48.4	0	-	M+Hac-H	C12H23N5O5	0.531583333333333	-	neg	376.18393577864	0.10353735881474	48.4	-
neg_254	Ala-Gly-Lys	metab_31481	B(i)	81.3	0	-	M-H	C11H22N4O4	0.536883333333333	-	neg	273.15704471673	0.022987316322632	55.2	-
neg_273	Gly-His	metab_31500	B(i)	90.2	0	HMDB0028843	M-H	C8H12N4O3	0.5422	-	neg	211.08376875821	0.027249707037572	56.8	-
neg_298	Arginylserine	metab_31525	B(ii)	0	51.2	HMDB0028718	M-H	C9H19N5O4	0.547516666666667	-	neg	260.13651711562	0.025939503064355	49.2	70921-62-9
neg_329	Creatinine Lactate	metab_31556	B(ii)	0	48.3	HMDB0250531	M+Na-2H	C7H11N3O4	0.5528	-	neg	222.04973224861	0.016833623668364	48.7	-
neg_418	Asn-Gln-Lys	metab_31645	B(i)	49.1	0	-	M+Hac-H	C15H28N6O6	0.558116666666667	-	neg	447.22106714615	0.032394942202445	49.3	-
neg_440	2-Oxoarginine	metab_31667	B(ii)	0	55.5	HMDB0004225	M-H2O-H	C6H11N3O3	0.563433333333333	C03771	neg	154.06233462626	0.016968801448719	49.3	3715/10/4;3715-10-4
neg_442	Carnosine	metab_31669	B(i)	78.3	0	HMDB0000033;PW_C000023	M-H	C9H14N4O3	0.563433333333333	C00386	neg	225.09939328184	0.035352083089721	54.9	305-84-0
neg_448	Arg-Gly-Gln	metab_31675	B(i)	59.9	0	-	M-H	C13H25N7O5	0.563433333333333	-	neg	358.18459756162	0.052017658708656	50.3	-

neg_477	Arg-Asp	metab_31704	B(i)	84.8	0	HMDB0028705	M-H	C10H19N5O5	0.5687	-	neg	288.13153037522	0.05947277456438	55.1	15706-88-4
neg_508	Cystathionine	metab_31735	B(i)	90.8	0	HMDB0033142;HMDB0303077;HMDB0303077	M-H	C7H14N2O4S	0.574	C00542	neg	221.06030948873	0.021504010952172	56.1	535-34-2;_
neg_552	Glycylglycine	metab_31779	B(i)	58.7	0	HMDB0011733	M+Hac-H	C4H8N2O3	0.5793	C02037	neg	191.06745323378	0.035440594750459	51.5	556-50-3
neg_555	Desglymidodrine	metab_31782	B(ii)	0	48.7	HMDB0060558	M+Na-2H	C10H15NO3	0.5793	C21521;_	neg	218.07832923793	0.046619234502119	45.8	3600-87-1;_
neg_568	3-Feruloylquinic Acid	metab_31795	B(ii)	0	78.3	HMDB0030669	M-H	C17H20O9	0.5793	C02572	neg	367.10523485193	0.046525577222036	52.6	62929-69-5
neg_584	Glucose 1-Phosphate	metab_31811	B(i)	88.8	0	HMDB0001586;PW_C001207	2M-H	C6H13O9P	0.5793	C00103	neg	519.0523177	0.05467409953167	56.7	59-56-3
neg_595	Taurine	metab_31822	B(i)	97.8	0	HMDB0000251;PW_C000171	M-H	C2H7NO3S	0.58456666666667	C00245	neg	124.0074815	0.057823171086422	57.8	107-35-7
neg_596	Succinamic Acid	metab_31823	B(ii)	0	44.3	HMDB0258538	M+FA-H	C4H7NO3	0.58456666666667	-	neg	162.04091598721	0.1344135158367	47.6	-
neg_597	Glycerol 3-Phosphate	metab_31824	B(i)	89.7	0	HMDB0000126;PW_C000081	M-H	C3H9O6P	0.58456666666667	C00093	neg	171.00650447821	0.026401203758432	57.1	57-03-4;17989-41-2
neg_598	Ala-Ser	metab_31825	B(i)	98.7	0	HMDB0028696	M-H	C6H12N2O4	0.58456666666667	-	neg	175.07252159552	0.028840123240908	58.8	3303-41-1
neg_599	Sn-Glycero-3-Phosphoethanolamine	metab_31826	B(ii)	0	75	HMDB0059660	M-H	C5H14NO6P	0.58456666666667	C01233;_	neg	214.04863161375	0.002835616073373	54	-;_
neg_607	Sedoheptulose 7-Phosphate	metab_31834	B(i)	71.8	0	HMDB0001068;HMDB0258206;HMDB0258206	M-H	C7H15O10P	0.58456666666667	C05382	neg	289.03320202602	0.19888765589956	52.7	-;_
neg_615	Digalacturonate	metab_31842	B(i)	78.6	0	HMDB0039721	M-H	C12H18O13	0.58456666666667	C02273	neg	369.06769221684	0.022462210154418	54.7	5894-59-7
neg_625	Neomangiferin	metab_31852	B(ii)	0	75.7	HMDB0255526	M-H	C25H28O16	0.58456666666667	-	neg	583.12817677414	0.022177459736902	51.3	-
neg_631	Gly-Asp	metab_31858	B(i)	95.3	0	HMDB0028837	M-H	C6H10N2O5	0.58985	-	neg	189.05177655828	0.01894208504383	57.7	-
neg_632	Gly-Gln	metab_31859	B(i)	78.8	0	HMDB0028839	M-H	C7H13N3O4	0.58985	-	neg	202.08345397989	0.029332786447863	54.8	-
neg_636	D-Manno-2-Heptulose	metab_31863	B(ii)	0	56.8	HMDB0029935	M+Cl	C7H14O7	0.58985	C08236	neg	245.04324100216	0.019748369562903	50.3	60426-75-7
neg_638	Glutaminylasparagine	metab_31865	B(ii)	0	62.7	HMDB0028792	M-H	C9H16N4O5	0.58985	-	neg	259.10486420666	0.012304231583425	51.3	14608-79-8
neg_640	Polyribosylribitolphosphate	metab_31867	B(ii)	0	43.6	HMDB0249913	M+FA-H	C5H13O8P	0.58985	-	neg	277.03299674791	0.010311962818171	48.5	-
neg_644	5-Aminoimidazole Ribonucleotide	metab_31871	B(ii)	0	66.7	HMDB0001235	M+Hac-H	C8H14N3O7P	0.58985	C03373	neg	354.0725174	0.043595746006298	51.5	25635-88-5
neg_646	Serylvalylglycylglutamic Acid	metab_31873	B(ii)	0	71.8	HMDB0302163	M-H	C15H26N4O8	0.58985	-	neg	389.16775353397	0.13856388110465	51.3	-
neg_651	L-Glutamine	metab_31878	B(i)	96.8	0	HMDB0000641;PW_C000500	M-H	C5H10N2O3	0.59526666666667	C00064	neg	145.06198263137	0.013145568574418	58.6	56-85-9
neg_652	Aminophylline	metab_31879	B(i)	51	0	HMDB0001889	M+Cl	C7H8N4O2	0.59526666666667	C07130	neg	215.03280357182	0.013697952070425	47.9	58-55-9
neg_653	Val-Lys	metab_31880	B(i)	80	0	HMDB0029132	M-H	C11H23N3O3	0.59526666666667	-	neg	244.16679862155	0.083674807469303	54.2	22677-62-9
neg_654	Homomethionine	metab_31881	B(ii)	0	59.5	HMDB0030406	2M-H	C6H13NO2S	0.59526666666667	C17213	neg	325.12533494481	0.018710187342108	50.3	-;25148-30-5
neg_656	Glutamic Acid	metab_31883	B(i)	99.3	0	HMDB0000148	M-H	C5H9NO4	0.60061666666667	C00025	neg	146.0459919	0.016252513850135	59.2	56-86-0
neg_657	N-Gluconyl Ethanolamine	metab_31884	B(ii)	0	59.8	HMDB0032293	M+Hac-H	C8H17NO7	0.60061666666667	-	neg	298.11443612924	0.035021052617594	50.6	686298-93-1
neg_660	4-Oxoproline	metab_31887	B(i)	46.2	0	-;HMDB0246561;HMDB0304793	M-H	C5H7NO3	0.60596666666667	_;C01877	neg	128.035423	0.060627495707023	47.8	4347-18-6;_
neg_661	Ala-Asn-Ser	metab_31888	B(i)	56	0	-	M-H	C10H18N4O6	0.6113	-	neg	289.11546797119	0.083468743766354	48.5	-
neg_666	Inositol	metab_31893	B(i)	59.6	0	HMDB0000211	2M-H	C6H12O6	0.6166	C00137	neg	359.11952979432	0.020796571133861	51.3	87-89-8
neg_671	Asp-Asp	metab_31898	B(i)	70.3	0	HMDB0028749	M-H2O-H	C8H12N2O7	0.62195	-	neg	229.04662335349	0.022951353100571	51.9	-
neg_672	Gly-Ala-Asp	metab_31899	B(i)	65.9	0	-	M-H	C9H15N3O6	0.62195	-	neg	260.08889670794	0.068703732105945	51.7	-
neg_674	Beta-Propiolactone	metab_31901	B(ii)	0	48.7	HMDB0249149	M-H	C3H4O2	0.6273	C19297	neg	71.013908807831	0.0364349785294	49.4	57-57-8;_
neg_676	Glutaric Acid	metab_31903	B(ii)	0	49	HMDB0000661	M-H2O-H	C5H8O4	0.6273	C00489	neg	113.02448973343	0.035213326254369	49.3	110-94-1
neg_677	D-Lyxose	metab_31904	B(i)	92.7	0	-	M-H	C5H10O5	0.6273	C00476	neg	149.04566368664	0.051003284396623	57.5	1114-34-7
neg_678	Alpha-D-Glucose	metab_31905	B(i)	95.8	0	HMDB0003345;PW_C001851	M-H	C6H12O6	0.6273	C00267	neg	179.05610076747	0.035980854258939	59	492-62-6
neg_679	Glucoheptonic Acid	metab_31906	B(ii)	0	64.8	HMDB0252766	M-H	C7H14O8	0.6273	-	neg	225.06165556633	0.030525563180883	52.5	-
neg_684	D-Xylonic Acid	metab_31911	B(ii)	0	68.5	HMDB0059750	M-H	C5H10O6	0.63258333333333	C00502	neg	165.04053815804	0.003710181210408	52.3	526-91-0
neg_685	Gulonic Acid	metab_31912	B(i)	90.8	0	HMDB0003290	M-H	C6H12O7	0.63258333333333	C00800	neg	195.05107773956	0.014285515822056	57.7	526-97-6;20246-53-1
neg_686	Gly-Ala-Thr	metab_31913	B(i)	64.9	0	-	M-H	C9H17N3O5	0.63258333333333	-	neg	246.1096349	0.10130917831107	50.9	-
neg_697	Thr-Asp	metab_31924	B(i)	73.9	0	HMDB0029057	M-H	C8H14N2O6	0.6432	-	neg	233.07787418646	0.020965951967931	53.9	108320-97-4
neg_703	Threonic Acid	metab_31930	B(i)	94.6	0	HMDB0000943	M-H	C4H8O5	0.64848333333333	C01620	neg	135.02998424785	0.025248451759018	57.7	7306-96-9
neg_705	Pinostrobin 5-Glucoside	metab_31932	B(ii)	0	43.4	HMDB0040503	M+Cl	C22H24O9	0.64848333333333	-	neg	467.10781607203	0.048712046445955	43.7	115799-14-9
neg_708	Cellobiose	metab_31935	B(i)	82.8	0	-;HMDB0000055;HMDB0242553;HMDB0246552	2M+FA-H	C12H22O11	0.64848333333333	C00185;C06422;_	neg	729.23004481244	0.022560487760048	54.3	16462-44-5;528-50-7;_

neg_711	Levoglucosan	metab_31938	B(i)	79	0	-;HMDB0000640	M+Hac-H	C6H10O5	0.65378333333333	_;C22350	neg	221.06669938587	0.03757615742873	55.3	498-07-7
neg_714	Melibiose	metab_31941	B(i)	91.6	0	-;HMDB0000048;HMDB0259486;PW_C000033	M-H	C12H22O11	0.65378333333333	C05402;_	neg	341.10883284438	0.02208403541679	58.1	585-99-9;13299-20-
neg_716	Lactobionic Acid	metab_31943	B(ii)	0	73.5	HMDB0253945	M+Na-2H	C12H22O12	0.65378333333333	-	neg	379.08231293261	0.008334121525892	51.7	-
neg_723	Laevuflex	metab_31950	B(i)	82.2	0	-	M-H2O-H	C6H12O6	0.65911666666667	-	neg	161.04554994875	0.01603695226999	56	-
neg_724	Lactulose	metab_31951	B(i)	82.1	0	HMDB0000740	M-H2O-H	C12H22O11	0.65911666666667	C07064	neg	323.09843655108	0.027029677250976	54.6	4618-18-2
neg_728	D-(-)-Ribose	metab_31955	B(i)	81.5	0	-	M-H2O-H	C5H10O5	0.66441666666667	-	neg	131.03507340987	0.024357271715557	55.4	-
neg_729	Fructose Lactate	metab_31956	B(ii)	0	54.5	HMDB0252491	M-H	C9H16O9	0.66441666666667	-	neg	267.07220121619	0.046323764540664	49.8	-
neg_735	Xylobiose	metab_31962	B(ii)	0	68.9	HMDB0029894	2M+FA-H	C10H18O9	0.66441666666667	C01630	neg	609.1883636	0.11130503185748	52.8	6860-47-5
neg_738	Dihydrolipoate	metab_31965	B(ii)	0	67.7	HMDB0012210	M-H	C8H16O2S2	0.66975	C02147	neg	207.05107498103	0.040515450663099	49.9	462-20-4
neg_739	2-[[2-[(2,4-Diamino-4-Oxobutanoyl)Amino]Acetyl]Amino]Pentanedioic Acid	metab_31966	B(i)	74.2	0	-	M-H	C11H18N4O7	0.66975	-	neg	317.110369	0.10728020788278	53.2	-
neg_749	D-Altro-D-Manno-Heptose	metab_31976	B(ii)	0	44.8	HMDB0029952	M-H2O-H	C7H14O7	0.67503333333333	C06397	neg	191.05615547121	0.025651340295975	48.3	1961-73-5
neg_750	Glu-Thr	metab_31977	B(i)	82.6	0	HMDB0028829	M-H	C9H16N2O6	0.67503333333333	-	neg	247.0935959	0.019927022691314	55.8	6875-80-5
neg_751	Rutinose	metab_31978	B(ii)	0	66.4	HMDB0302439	M+FA-H	C12H22O10	0.67503333333333	_;C08247	neg	371.11957130178	0.025103298133968	52.6	90-74-4;_
neg_755	Vincosamide	metab_31982	B(ii)	0	65.9	HMDB0259813	M+Cl	C26H30N2O8	0.67503333333333	-	neg	533.17241197682	0.086159525862882	49.4	-
neg_768	Ethylphosphate	metab_31995	B(ii)	0	43.4	HMDB0012228	M-H	C2H7O4P	0.68558333333333	-	neg	125.00100485801	0.068423172946729	47.9	78-40-0
neg_770	Galactosylglycerol	metab_31997	B(ii)	0	61	HMDB0006790	M-H	C9H18O8	0.68558333333333	C05401	neg	253.09300680222	0.031012670134302	51.2	-;_
neg_779	Val-Ser	metab_32006	B(i)	91.1	0	HMDB0029136	M-H	C8H16N2O4	0.69085	-	neg	203.10375992811	0.15596507746735	56.8	13588-94-8
neg_780	Ile-Asn	metab_32007	B(i)	71.3	0	HMDB0028902	M-H	C10H19N3O4	0.69085	-	neg	244.13036010802	0.060285056970203	52.7	-
neg_790	Cytidine 3'-Phosphate	metab_32017	B(i)	97.8	0	HMDB0240312	M-H	C9H14N3O8P	0.6962	C05822	neg	322.04466202913	0.060723096729521	57.6	84-52-6
neg_793	Fexofenadine	metab_32020	B(i)	45.8	0	HMDB0005030;PW_C002373	M+Hac-H	C32H39NO4	0.6962	C06999	neg	560.30503311798	0.15179603444662	43.7	83799-24-0
neg_807	Propynoic Acid	metab_32034	B(ii)	0	41.5	HMDB0006804	M+FA-H	C3H2O2	0.71208333333333	C00804	neg	115.00375660483	0.029587596947835	47.9	471-25-0
neg_808	L-Malic Acid	metab_32035	B(i)	99.4	0	HMDB0000156;PW_C000101	M-H	C4H6O5	0.71208333333333	C00149	neg	133.01430402584	0.021568048366738	59.7	97-67-6
neg_812	Dhghab	metab_32039	B(ii)	0	58.6	HMDB0243628	M+FA-H	C22H17N5O8	0.71208333333333	-	neg	524.10354978945	0.02922945170335	46.6	-
neg_819	Ajugose	metab_32046	B(ii)	0	95.4	HMDB0304260	M-H	C36H62O31	0.72268333333333	C08238;_	neg	989.31989890271	0.039297672035264	57.9	512-72-1;_
neg_825	Sialyl-Lewisx	metab_32052	B(ii)	0	62.9	HMDB0258283	M+Na-2H	C31H52N2O23	0.728	-	neg	841.26482700836	0.037611640617852	48.7	-
neg_828	Hexcer(29:1_2O/21:1)	metab_32055	B(i)	43.1	0	-	M+Hac-H	C56H107NO8	0.728	-	neg	980.817076	0.03005042303872	40.7	-
neg_840	Pelargonidin 3-Rutinoside	metab_32067	B(ii)	0	59.6	HMDB0303787	M+FA-H	C27H31O14+	0.73863333333333	C12644	neg	624.16694565675	0.28703912916383	47.4	_;
neg_841	Sesaminol Glucosyl-(1->2)-Glucoside	metab_32068	B(ii)	0	89.8	HMDB0041210	M+Hac-H	C32H38O17	0.73863333333333	-	neg	753.23076415263	0.13416423009182	53.4	157469-82-4
neg_864	Succinic Anhydride	metab_32091	B(ii)	0	67	HMDB0032523	M+FA-H	C4H4O3	0.74923333333333	C19524	neg	145.01434303729	0.032014622070207	52.9	108-30-5
neg_873	3-Dehydroshikimic Acid	metab_32100	B(i)	72.2	0	-	M-H	C7H8O5	0.75451666666667	-	neg	171.02997992034	0.18006664685167	52.6	-
neg_874	N-Fructosyl Pyroglutamate	metab_32101	B(i)	66.1	0	-	M-H	C11H17NO8	0.75451666666667	-	neg	290.08827993759	0.013137514626585	51.2	-
neg_898	B-D-Glucuronopyranosyl-(1->3)-A-D-Galacturonopyranosyl-(1->2)-L-Rhamnose	metab_32125	B(ii)	0	67.5	HMDB0039728	M-H2O-H	C18H28O17	0.77075	-	neg	497.11252886257	0.022096235141121	50.4	-
neg_908	Safflomin C	metab_32135	B(ii)	0	73	HMDB0034052	M+FA-H	C30H30O14	0.77605	-	neg	659.16529369231	0.01719959498422	50.7	126093-98-9;
neg_916	Udp-N-Acetyl-D-Galactosamine	metab_32143	B(i)	83.7	0	HMDB0006591	M-H	C17H27N3O17P2	0.78135	C00203	neg	606.07440525152	0.041181554520046	54.4	-
neg_926	2-Hydroxy-1,4-Benzoxazin-3-One Glucuronide	metab_32153	B(ii)	0	48.5	HMDB0240438	2M+FA-H	C14H15NO9	0.78668333333333	-	neg	727.15287437417	0.059839676063776	45.5	-
neg_965	Sesamose	metab_32192	B(ii)	0	95.1	HMDB0038871	M+FA-H	C24H42O21	0.8931	C08248	neg	711.22024920891	0.011862162750655	58.7	58985-23-2;_
neg_973	2-(3,4-Dicarboxy-3-Hydroxybutanoyl)Oxypropane-1,2,3-Tricarboxylic Acid	metab_32200	B(ii)	0	46.9	HMDB0257792	M-H2O-H	C12H14O13	0.90375	-	neg	347.02328410055	0.12224195743073	46.5	-
neg_978	Henagliflozin	metab_32205	B(ii)	0	69	HMDB0253086	2M-H	C22H24ClFO7	0.90375	-	neg	907.23420702712	0.17678137527515	46.2	-
neg_986	Oxaprozin Glucuronide	metab_32213	B(ii)	0	53.9	HMDB0255967	2M+FA-H	C24H23NO9	0.92508333333333	-	neg	983.27196052809	0.002921104876326	47.6	-
neg_991	Diethyl Phosphate	metab_32218	B(i)	57.7	0	HMDB0012209	M+FA-H	C4H11O4P	0.93576666666667	C06608	neg	199.03774903134	0.015000325349434	50.9	598-02-7
neg_993	(Z)-Narceine Imide	metab_32220	B(ii)	0	62.4	HMDB0030378	M+Cl	C23H26N2O6	0.93576666666667	-	neg	461.15117163251	0.052624883450621	46.7	55968-77-9
neg_996	Core Oligosaccharide	metab_32223	B(ii)	0	94.7	HMDB0250452	M-H	C27H48O24	0.94106666666667	-	neg	755.24630589103	0.03088904224739	58.2	-
neg_1019	Diaportheone A	metab_32246	B(i)	47.7	0	-	M+Hac-H	C12H10O4	0.96238333333333	-	neg	277.07363238968	0.087893971968035	45.3	-

neg_1021	Petanin	metab_32248	B(ii)	0	43.6	HMDB0038095	M-H	C43H49O23+	0.96238333333333	C12139;_	neg	932.25863684535	0.2776361621234	45.5	69915-09-9;
neg_1044	Endothion	metab_32271	B(ii)	0	41.9	HMDB0251802	M-H	C9H13O6PS	0.97838333333333	C18982	neg	279.00988571099	0.038453832091593	45.7	2778/4/3;_
neg_1046	Benazeprilat	metab_32273	B(ii)	0	42.1	HMDB0060582	M+Cl	C22H24N2O5	0.97838333333333	_;C21541	neg	431.14078708557	0.038448956163288	44.4	86541-78-8;_
neg_1062	Neoacrimarine G	metab_32289	B(ii)	0	45.7	HMDB0031135	2M-H	C29H25NO8	0.98371666666667	-	neg	1029.312935	0.036671388162442	47.2	195057-37-5
neg_1064	Glu-Ser-Ala	metab_32291	B(i)	67.7	0	-	M-H	C11H19N3O7	0.98905	-	neg	304.11509893712	0.024411537370614	51.1	-
neg_1068	Arg-Arg-Tyr	metab_32295	B(i)	68.7	0	-	M-H	C21H35N9O5	0.98905	-	neg	492.268929	0.11055488661995	51.1	-
neg_1074	Val-Gln	metab_32301	B(i)	91.9	0	HMDB0029125	M-H	C10H19N3O4	0.99435	-	neg	244.13037193678	0.10046615000049	57.1	42854-54-6
neg_1077	Glycosides	metab_32304	B(ii)	0	80.3	HMDB0031448	M+Na-2H	C29H44O12	0.99435	C01443	neg	605.25366147467	0.17062841811867	50.4	-
neg_1079	Mangicrocin	metab_32306	B(ii)	0	50.2	HMDB0034055	M+Hac-H	C45H50O19	0.99435	-	neg	953.30490944255	0.10813817273127	44.1	122575-51-3
neg_1092	Maltohexaose	metab_32319	B(i)	55.6	0	HMDB0012253	M-H	C36H62O31	0.99966666666667	C01936	neg	989.32029139578	0.049393414872509	50.1	34620-77-4
neg_1102	Lacto-N-Difucopentaose Ii	metab_32329	B(ii)	0	62.5	HMDB0001986	M+Cl	C38H65NO29	1.00495	-	neg	1034.3352785705	0.042855316326263	44.3	62258-12-2
neg_1117	Asn-Val-Asn	metab_32344	B(i)	80.1	0	-	M-H	C13H23N5O6	1.0155833333333	-	neg	344.1576148	0.031116148503116	52.8	-
neg_1124	Tetrahydrofolyl-[Glu](N)	metab_32351	B(ii)	0	56.5	HMDB0006826	M+Na-2H	C29H37N9O12	1.0155833333333	C03541	neg	724.22810024239	0.0359310847068	46.2	-;_
neg_1129	Pseudouridine	metab_32356	B(i)	76.8	0	HMDB0000767	M-H	C9H12N2O6	1.0208833333333	C02067	neg	243.06230890956	0.020178904349382	53.9	1445-07-4
neg_1130	Gln-Ala-Thr	metab_32357	B(i)	58.5	0	-	M-H	C12H22N4O6	1.0208833333333	-	neg	317.14668885828	0.28640808787105	49	-
neg_1131	Kyotorphin	metab_32358	B(ii)	0	65.6	HMDB0005768	M-H	C15H23N5O4	1.0208833333333	C02993	neg	336.16784926601	0.20882069164882	50.2	70904-56-2
neg_1155	Lactic Acid	metab_32382	B(i)	65.7	0	-	M-H	C3H6O3	1.0368833333333	C01432	neg	89.024410409527	0.01977361044127	53	50-21-5
neg_1158	6-O-P-Coumaroyl-D-Glucose	metab_32385	B(ii)	0	59.7	HMDB0039169	2M-H	C15H18O8	1.0368833333333	-	neg	651.19901873273	0.02073344115563	46.6	114297-65-3
neg_1160	D-Glucoheptose	metab_32387	B(i)	42.2	0	-	M+Hac-H	C7H14O7	1.0421833333333	-	neg	269.08786512237	0.025848275115394	47.9	-
neg_1164	Gibberellin A8-Catabolite	metab_32391	B(ii)	0	59.2	HMDB0304364	M+Na-2H	C19H22O7	1.0475166666667	C11870;_	neg	383.11302079146	0.05146618463846	47.4	78791-79-4;_;
neg_1167	D-Fructose	metab_32394	B(ii)	0	63.3	HMDB0000660	M+Na-2H	C6H12O6	1.0581166666667	C10906;C00095; C02336;C05003	neg	201.03811546627	0.010465557323116	52.3	57-48-7;-;53188-23-
neg_1171	Methyl 2-((3,4-Dihydro-3,4-Dioxo-1-	metab_32398	B(ii)	0	43.8	HMDB0244408	M+Na-2H	C18H13NO4	1.0581166666667	-	neg	328.05861606666	0.10878245267031	44.8	-
neg_1180	D-Alpha-Hydroxyglutaric Acid	metab_32407	B(i)	98.7	0	-	M-H	C5H8O5	1.0687333333333	-	neg	147.02999373372	0.001758844410108	59.3	-
neg_1190	3'-Sialyl-3-Fucosyllactose	metab_32417	B(ii)	0	45.5	HMDB0006606	M+Na-2H	C29H49NO23	1.0793666666667	-	neg	800.23960190516	0.099356639505116	41	122560-33-2
neg_1191	Angoroside C	metab_32418	B(ii)	0	63.5	HMDB0248432	M+Na-2H	C36H48O19	1.0793666666667	-	neg	805.25473936008	0.047807878966341	48	-
neg_1203	Licorice Glycoside E	metab_32430	B(ii)	0	51.4	HMDB0031996	M+Hac-H	C35H35NO14	1.0846666666667	-	neg	752.22237486616	0.16007604524108	45	_;
neg_1277	Tetraglucoside	metab_32504	B(ii)	0	62.3	HMDB0258892	M+FA-H	C24H42O21	1.1326333333333	-	neg	711.22021397996	0.013350367199413	50.8	-
neg_1290	H-Tyr-Gly-Gly-Oh	metab_32517	B(ii)	0	54.9	HMDB0247865	M-H	C13H17N3O5	1.1538666666667	-	neg	294.10971160046	0.02961898179546	48.6	-
neg_1293	Isocitric Acid	metab_32520	B(i)	75.8	0	HMDB0000193;PW_C000125	M-H2O-H	C6H8O7	1.15915	C00311	neg	173.00922994668	0.009002270007246	54.4	320-77-4
neg_1301	Ser-Glu-Gln	metab_32528	B(i)	69.8	0	-	M-H2O-H	C13H22N4O8	1.1644666666667	-	neg	343.12584765843	0.029886878434724	51	-
neg_1303	Cholylmethionine	metab_32530	B(ii)	0	44.1	HMDB0242382	M+Na-2H	C29H49NO6S	1.1644666666667	-	neg	560.3051444	0.004939844936632	44.6	-
neg_1304	D-Glucaro-1,4-Lactone	metab_32531	B(ii)	0	49.9	HMDB0041862	M+Na-2H	C6H8O7	1.1697666666667	C21095;_	neg	213.00178301923	0.009589499163518	48.7	5027-63-4
neg_1308	Quercetin-3'-Glucuronide	metab_32535	B(ii)	0	45.6	HMDB0029213	M+Cl	C21H20O13	1.1697666666667	-	neg	515.05569846285	0.055983098664761	44.9	-
neg_1313	Streptomycin	metab_32540	B(ii)	0	84.6	HMDB0015214	M-H2O-H	C21H39N7O12	1.1753333333333	_;C00413	neg	562.24788032804	0.021336928530376	55.9	57-92-1;_
neg_1315	3,7-Dihydropurin-6-One	metab_32542	B(i)	85.1	0	-	M-H	C5H4N4O	1.1806833333333	-	neg	135.03136405304	0.03524344095596	55.4	-
neg_1320	D-Xylulose 5-Phosphate	metab_32547	B(i)	70.9	0	HMDB0000868	M-H2O-H	C5H11O8P	1.1913333333333	C00231	neg	211.00138787027	0.022806978709779	53.4	-
neg_1323	Cefovecin	metab_32550	B(ii)	0	43.4	HMDB0249771	M+Na-2H	C17H19N5O6S	1.1966333333333	-	neg	474.05320015805	0.023228466367202	44.5	-
neg_1326	Uridine 2'-Phosphate	metab_32553	B(ii)	0	59.7	HMDB0011641	M+Na-2H	C9H13N2O9P	1.20195	C03031	neg	345.01050370654	0.05927678174186	50.2	-;131-83-9
neg_1332	Lys-Gln-Ile	metab_32559	B(i)	38.6	0	-	M-H	C17H33N5O5	1.2072333333333	-	neg	386.24104056099	0.006026693728177	43.9	-
neg_1335	3-Aminopentanedioic Acid	metab_32562	B(i)	45	0	-	M-H2O-H	C5H9NO4	1.2126	C05574	neg	128.03540906973	0.005637338772164	48.4	-
neg_1336	L-2-Amino-4-Methylenepentanedioic Acid	metab_32563	B(ii)	0	49.4	HMDB0029433	M+FA-H	C6H9NO4	1.2126	C00651	neg	204.05141658967	0.015215929246781	48.4	16804-57-2
neg_1337	Glu-Gly-Asp	metab_32564	B(i)	60.8	0	-	M-H2O-H	C11H17N3O8	1.2126	-	neg	300.08389052175	0.044442980003319	49.3	-
neg_1344	Asp-Ser-Pro	metab_32571	B(i)	42.7	0	-	M-H	C12H19N3O7	1.21795	-	neg	316.11508220031	0.076601369500832	45.8	-
neg_1355	Glu-Asn-Leu	metab_32582	B(i)	58.4	0	-	M-H	C15H26N4O7	1.2285333333333	-	neg	373.17240986922	0.049132332895633	48.9	-
neg_1356	Catechol	metab_32583	B(i)	56.8	0	-;HMDB0000957	M-H	C6H6O2	1.23385	C00090;C15571	neg	109.02957789923	0.18612153837282	42.6	120-80-9;-

neg_1357	Gly-Val-Asn	metab_32584	B(i)	42.2	0	-	M+Hac-H	C11H20N4O5	1.23385	-	neg	347.15746511017	0.076462385619045	45.8	-
neg_1376	Asn-Thr-Tyr	metab_32603	B(i)	52.5	0	-	M-H	C17H24N4O7	1.2606166666667	-	neg	395.15648579928	0.22758970382646	46.7	-
neg_1378	Cortistatin A	metab_32605	B(ii)	0	43.9	HMDB0250469	M-H	C30H36N2O3	1.2606166666667	-	neg	471.26863918323	0.11542912524747	43.4	-
neg_1382	De-O-Methylsimmondsin	metab_32609	B(ii)	0	94.5	HMDB0041208	M+FA-H	C15H23NO9	1.2659666666667	-	neg	406.13441887755	0.002921104876326	54.8	135105-75-8
neg_1391	Arabitol	metab_32618	B(i)	43.9	0	HMDB0000568	M-H2O-H	C5H12O5	1.2766666666667	C01904	neg	133.05072552381	0.021706407595529	48.5	488-82-4
neg_1396	S-(1,2-Dicarboxyethyl)Glutathione	metab_32623	B(i)	59.1	0	HMDB0257378	M-H	C14H21N3O10	1.2766666666667	-	neg	422.0873221	0.12398134441341	47.8	-
neg_1398	Ile-Ser	metab_32625	B(i)	86.9	0	HMDB0028916	M-H	C9H18N2O4	1.2819666666667	-	neg	217.11945556768	0.005832404337333	56.6	-
neg_1400	7-Oxoprostaglandin I2	metab_32627	B(ii)	0	47.3	HMDB0247284	M+Hac-H	C20H30O6	1.2819666666667	-	neg	425.21534169822	0.002921104876326	44.6	-
neg_1413	Leu-Gly-Asp	metab_32640	B(i)	51.4	0	-	M-H	C12H21N3O6	1.3138333333333	-	neg	302.13655299422	0.097686391240378	47.3	-
neg_1418	Delafloxacin	metab_32645	B(ii)	0	57.8	HMDB0250964	M+Na-2H	C18H12ClF3N4O4	1.3244333333333	-	neg	461.02162477102	0.26167235396959	43.2	-
neg_1419	11-(3-Methyl-5-Propylfuran-2-Yl)Undecanoyl-Coa	metab_32646	B(ii)	0	41.1	HMDB0301550	M-H2O-H	C40H66N7O18P3S	1.3244333333333	-	neg	1038.3205155183	0.059483180520128	43.7	-
neg_1424	Adenylosuccinic Acid	metab_32651	B(ii)	0	72.6	HMDB0248010	M+Na-2H	C14H18N5O11	1.3350833333333	_;C03794	neg	484.04891494076	0.10387712289754	52.5	19046-78-7;_
neg_1425	Citraconic Acid	metab_32652	B(i)	62.7	0	HMDB0000634;LMFA01170099	M+Hac-H	C5H6O4	1.3404166666667	C02226	neg	189.04053458171	0.046380888250926	51.3	498-23-7;
neg_1426	Methylisocitric Acid	metab_32653	B(ii)	0	42.9	HMDB0006471	M+Na-2H	C7H10O7	1.3404166666667	C04593	neg	227.01742210526	0.026497026371165	47.2	-;_
neg_1429	8-Oxo-Dgmp	metab_32656	B(ii)	0	59.5	HMDB0011670	M+Na-2H	C10H14N5O8P	1.3404166666667	_;C19968	neg	384.03215888807	0.15183039729091	50	127027-50-3;_
neg_1436	Bilirubin Diglucuronide	metab_32663	B(ii)	0	56.1	HMDB0003325	M+Na-2H	C45H52N4O18	1.3457166666667	C05787	neg	957.29396406045	0.23171688791868	46.9	17459-92-6
neg_1452	Etodolac	metab_32679	B(ii)	0	40.8	HMDB0014887	M+Na-2H	C17H21NO3	1.3563333333333	C06991	neg	308.12540243254	0.042679301041736	45.1	41340-25-4
neg_1466	Pretyrosine	metab_32693	B(ii)	0	56.9	HMDB0304809	M+Na-2H	C10H13NO5	1.3670333333333	C00826	neg	248.05416253436	0.007056223326900	50.4	53078-86-7;_
neg_1472	Dl-M-Tyrosine	metab_32699	B(i)	97.3	0	-	M-H	C9H11NO3	1.3776333333333	-	neg	180.06667140328	0.004232675350441	57.3	-
neg_1482	Leu-Gln	metab_32709	B(i)	92.3	0	HMDB0028927	M-H	C11H21N3O4	1.4148666666667	-	neg	258.14603985931	0.005271800361060	57.6	-
neg_1490	N-(2-Methyl-5-Nitrophenyl)-N-Methylsulonylmethanesulonamide	metab_32717	B(i)	48.5	0	-	M+Hac-H	C9H12N2O6S2	1.47345	-	neg	367.02588440381	0.061753673995059	44.6	-
neg_1491	3-Oxoadipic Acid	metab_32718	B(ii)	0	73.8	HMDB0000398	M+FA-H	C6H8O5	1.47875	C00846	neg	205.0354684	0.004071593154074	54.3	689-31-6
neg_1492	Glucose Pyruvate Acetate	metab_32719	B(ii)	0	49.5	HMDB0252787	M+Na-2H	C11H16O11	1.47875	-	neg	345.0440297	0.026903093471688	47.1	-
neg_1503	Propionic Acid	metab_32730	B(i)	44.8	0	HMDB0000237;LMFA01010003;PW_C000159	M-H	C3H6O2	1.5212	C00163	neg	73.029559756811	0.018099842942238	48.6	1979/9/4;79-09-4;
neg_1504	Succinic Acid	metab_32731	B(i)	99.6	0	HMDB0000254;HMDB0303309;LMFA01170043;PW_C000174	M-H	C4H6O4	1.5212	C00042;_	neg	117.01936419628	0.01048873623431	59.8	110-15-6;_;
neg_1508	6-Methyl 2-Galloylgalactarate	metab_32735	B(ii)	0	59.8	HMDB0036934	M+Na-2H	C14H16O12	1.5212	-	neg	397.03656111559	0.015234670777477	49.6	-
neg_1516	Cyanidin 5-O-Beta-D-Glucoside	metab_32743	B(ii)	0	50.3	HMDB0304305	M+Cl	C21H19O11-	1.5265	-	neg	482.06585834796	0.076982017529053	44.2	-
neg_1527	1H,1H,7H-Dodecafluoroheptan-1-Ol	metab_32754	B(i)	48.9	0	-	M-H	C7H4F12O	1.5530666666667	-	neg	331.00236015069	0.15761118024201	46.5	-
neg_1529	Arg-Leu	metab_32756	B(i)	93.9	0	HMDB0028713	M-H	C12H25N5O3	1.55835	-	neg	286.18860471416	0.006975205710256	57.5	1188-24-5
neg_1536	N-(2-Hydroxy-1,3-Dioxoinden-2-Yl)-4-(Thiadiazol-4-	metab_32763	B(i)	85	0	-	M+FA-H	C18H11N3O4S	1.57435	-	neg	410.04507099084	0.26777861669806	53.4	-
neg_1538	Doxantrazole	metab_32765	B(ii)	0	43.1	HMDB0251600	M+Cl	C14H8N4O3S	1.5849666666667	-	neg	346.99968315323	0.15185143597669	43.5	-
neg_1546	Kojic Acid	metab_32773	B(i)	72	0	HMDB0032923	M-H	C6H6O4	1.60625	C14516	neg	141.0194585	0.083175764113183	52.7	501-30-4
neg_1548	Ala-Gly-Met	metab_32775	B(i)	67.5	0	-	M-H	C10H19N3O4S	1.6221666666667	-	neg	276.10253816723	0.006391425356009	52	-
neg_1550	N-Acetyl-L-Alanine	metab_32777	B(i)	93.8	0	HMDB0000766	M-H	C5H9NO3	1.6275333333333	-	neg	130.05106145147	0.004408849961013	58.1	97-69-8
neg_1553	Ala-Met	metab_32780	B(i)	91.5	0	HMDB0028693	M-H	C8H16N2O3S	1.6328333333333	-	neg	219.08108248663	0.021916955207261	56.2	14486-05-6
neg_1555	Gamma-Aminobutyric Acid Glutamate	metab_32782	B(ii)	0	53.8	HMDB0252609	M+Na-2H	C9H16N2O5	1.6434833333333	-	neg	253.0808261	0.021452932395062	48.7	-
neg_1559	22-Deoxocucurbitacin D	metab_32786	B(ii)	0	57.4	HMDB0034705	M+FA-H	C30H46O6	1.65945	-	neg	547.32959568241	0.031898974417552	47.8	15371-78-5
neg_1561	Met-Ser-Asn	metab_32788	B(i)	76.3	0	-	M-H	C12H22N4O6S	1.6700333333333	-	neg	349.11889486555	0.18963054111767	54	-
neg_1563	Difructose Anhydride Iii	metab_32790	B(ii)	0	42.1	HMDB0251271	M+Hac-H	C12H20O10	1.6753333333333	C04420;_	neg	383.12255746085	0.083566901254506	45.1	-;_
neg_1564	Skepinone-L	metab_32791	B(ii)	0	74	HMDB0258326	M+Na-2H	C24H21F2NO4	1.6753333333333	-	neg	446.1182803	0.1440701809363	52	-
neg_1575	Uridine	metab_32802	B(i)	94.1	0	HMDB0000296;PW_C000202	M-H	C9H12N2O6	1.6913333333333	C00299	neg	243.06229925996	0.097488168910682	58.7	58-96-8
neg_1579	Mytilin A	metab_32806	B(ii)	0	41.3	HMDB0039002	M+Hac-H	C13H20N2O8	1.6913333333333	-	neg	391.1359622	0.1309109366078	47.5	73112-73-9
neg_1601	Glu-Glu	metab_32828	B(i)	88.5	0	HMDB0028818	M-H2O-H	C10H16N2O7	1.7446	C01425	neg	257.07803139381	0.024251989954459	56	3929-61-1

neg_1607	3',5'-Cyclic Gmp	metab_32834	B(i)	83	0	HMDB0001314	M-H	C10H12N5O7P	1.8193666666667	C00942	neg	344.04027813902	0.085750218116193	55.2	7665-99-8
neg_1610	Grifola Frandosa Lectin	metab_32837	B(ii)	0	41.5	HMDB0303396	M+Cl	C6H11FO5	1.8408833333333	-	neg	217.02997784676	0.038198014516752	42.3	-
neg_1613	Dehydroascorbic Acid	metab_32840	B(ii)	0	52.5	HMDB0001264	M+Na-2H	C6H6O6	1.85155	C00425;C05422	neg	194.99119899594	0.005088024407017	49	490-83-5
neg_1621	Rutacridone Epoxide	metab_32848	B(ii)	0	40.7	HMDB0033853	M+Na-2H	C19H17NO4	1.8941666666667	C10739	neg	344.09007318455	0.035273383599381	45	77996-03-3
neg_1633	Ethyl 4-Hydroxy-3-Methylisoxazolo[5,4-B]Pyridine-5-	metab_32860	B(i)	36.2	0	-	M+FA-H	C10H10N2O4	1.92085	-	neg	267.06014367524	0.082811785960142	43.4	-
neg_1636	Sitostanol-Beta	metab_32863	B(ii)	0	52.2	HMDB0304734	M-H2O-H	C12H14N2O7	1.9264666666667	-	neg	279.06010985685	0.036930987546052	46.8	-
neg_1643	Glu-Arg-Leu	metab_32870	B(i)	65	0	-	M-H	C17H32N6O6	1.9317666666667	-	neg	415.23129535604	0.12965870813409	50.7	-
neg_1654	Gln-Val-Asn	metab_32881	B(i)	69.2	0	-	M-H	C14H25N5O6	1.9424	-	neg	358.17326719628	0.12864676224771	51.7	-
neg_1661	Ile-Glu-Ser	metab_32888	B(i)	50.5	0	-	M-H2O-H	C14H25N3O7	1.9477333333333	-	neg	328.1515689	0.048103052250949	48.7	-
neg_1663	Leu-Gln-Asn	metab_32890	B(i)	77	0	-	M-H	C15H27N5O6	1.9477333333333	-	neg	372.18900469249	0.07200331739592	53.2	-
neg_1672	1-Methyl-4-Piperidyl Acetate	metab_32899	B(ii)	0	40.7	HMDB0243931	M+FA-H	C8H15NO2	1.9530333333333	-	neg	202.10860560293	0.12541484512643	47.2	-
neg_1674	Ile-Glu	metab_32901	B(i)	95.1	0	HMDB0028906	M-H	C11H20N2O5	1.9530333333333	-	neg	259.13004680817	0.008922082671293	58.4	-
neg_1675	Asn-Val-Ala	metab_32902	B(i)	58.4	0	-	M-H	C12H22N4O5	1.9530333333333	-	neg	301.1518692	0.074154533699545	49.3	-
neg_1677	Gln-Gly-Ile	metab_32904	B(i)	53.2	0	-	M-H	C13H24N4O5	1.9530333333333	-	neg	315.16750686603	0.062022837647504	48.4	-
neg_1688	D-Erythroascorbic Acid 1'-A-D-Xylopyranoside	metab_32915	B(ii)	0	49	HMDB0033626	2M-H	C10H14O9	1.9530333333333	-	neg	555.120441	0.03879976864161	48.4	174339-85-6
neg_1698	Ser-Ile-His	metab_32925	B(i)	60.4	0	-	M-H	C15H25N5O5	1.9583833333333	-	neg	354.17657547883	0.002030428003133	48.6	-
neg_1703	1-Methyladenosine	metab_32930	B(ii)	0	42.5	HMDB0003331	2M-H	C11H15N5O4	1.9583833333333	C02494	neg	561.21613353374	0.15278106639182	46.3	15763-06-1
neg_1707	5-Methyl-2-Furancarboxylic Acid	metab_32934	B(i)	83.9	0	-	M-H	C6H6O3	1.9637166666667	-	neg	125.02452874603	0.076026918262233	55.8	-
neg_1708	2-Methylglutaric Acid	metab_32935	B(i)	80.6	0	HMDB0000422	M-H	C6H10O4	1.9637166666667	-	neg	145.05075973031	0.021290543205864	55.7	617-62-9
neg_1712	Inosine	metab_32939	B(i)	90.7	0	HMDB0000195;HMDB0247301;PW_C000127	M-H	C10H12N4O5	1.9637166666667	C00294;_	neg	267.07349281119	0.004170398246351	57.1	58-63-9;_
neg_1715	3'-Adenylic Acid	metab_32942	B(i)	97.1	0	HMDB0003540;HMDB0258140	M-H	C10H14N5O7P	1.9637166666667	C01367;_	neg	346.0558437	0.25331985871969	58.8	84-21-9;_
neg_1741	Gibberellin A8	metab_32968	B(ii)	0	58.7	HMDB0303450	2M-H	C19H24O7	1.9637166666667	_;C03579	neg	727.30168633028	0.081554923942961	47.9	7044-72-6;_;
neg_1743	B-D-Xylopyranosyl-(1->4)-A-L-Rhamnopyranosyl-(1->2)-L-Arabinose	metab_32970	B(ii)	0	87.8	HMDB0041221	2M+FA-H	C16H28O13	1.9637166666667	-	neg	901.30339033853	0.038731338567343	55.4	-
neg_1747	Adenosine	metab_32974	B(i)	88.6	0	HMDB0000050;HMDB0247583;PW_C000034	M-H	C10H13N5O4	1.9690333333333	C00212;_	neg	266.08960885634	0.11039692533946	56.2	58-61-7;_
neg_1748	Guanosine	metab_32975	B(i)	98.5	0	HMDB0000133;PW_C000087	M-H	C10H13N5O5	1.9690333333333	C00387	neg	282.08445631767	0.003150548984342	59	118-00-3
neg_1751	Ile-Thr-Asn	metab_32978	B(i)	86.2	0	-	M-H	C14H26N4O6	1.9690333333333	-	neg	345.17804824448	0.2834822983241	56	-
neg_1752	Amylose	metab_32979	B(ii)	0	64.9	HMDB0003403	M+FA-H	C2H6.[C12H20O11]n	1.9690333333333	C00420;C00718	neg	415.14594513718	0.0073954595583051	32.8	9005-82-7;6118-87-2
neg_1753	Lacto-N-Biose I	metab_32980	B(ii)	0	54.4	HMDB0006575	M+FA-H	C14H25NO11	1.9690333333333	C06372	neg	428.14252670197	0.18281969542458	48.3	50787-09-2
neg_1776	Citrinin	metab_33003	B(ii)	0	50.3	HMDB0041857	2M+FA-H	C13H14O5	1.9743833333333	C16765	neg	545.16677819295	0.029068435505161	49.4	518-75-2
neg_1785	Guanosine 3'-Monophosphate	metab_33012	B(ii)	0	75	HMDB0240587	M+Na-2H	C10H14N5O8P	1.9796833333333	C06193	neg	384.03277728879	0.027191420429558	54	117-68-0
neg_1791	Val-Tyr-Asn	metab_33018	B(i)	68.4	0	-	M-H	C18H26N4O6	1.9902833333333	-	neg	393.17794193013	0.08821860668012	51.9	-
neg_1797	(E)-3-Hydroxy Tamoxifen O-|A-D-Glucuronide	metab_33024	B(ii)	0	41.4	HMDB0244742	M-H	C32H37NO8	1.9902833333333	-	neg	562.24200083812	0.005640847736925	43.2	-
neg_1804	Poly-G-D-Glutamate	metab_33031	B(ii)	0	57.2	HMDB0004135	M+Hac-H	C20H30N4O12	1.9956	C05723	neg	577.19883472427	0.015138299416008	50	-
neg_1807	4-(Glutamylamino) Butanoate	metab_33034	B(ii)	0	46	HMDB0012161	M-H	C9H16N2O5	2.0009	C15767	neg	231.09873592681	0.021615780529395	47	5105-96-4
neg_1810	2-Phenylethyl 6-O-Alpha-L-Lyxofuranosyl-Beta-D-	metab_33037	B(i)	85.4	0	-	M+FA-H	C19H28O10	2.0009	-	neg	461.16576372174	0.072986712831519	55.2	-
neg_1815	Ala-Leu-Ser	metab_33042	B(i)	73.5	0	-	M-H	C12H23N3O5	2.0062166666667	-	neg	288.15656284827	0.016764963921984	53.3	-
neg_1816	(4R,5S,7R,11S)-11,12-Dihydroxy-1(10)-Spirovetiven-2-One 11-Glucoside	metab_33043	B(ii)	0	63.6	HMDB0033150	M+Hac-H	C21H34O8	2.0062166666667	-	neg	473.23662294217	0.1255155896255	49.4	-
neg_1818	2,3-Butanediol Glucoside	metab_33045	B(ii)	0	42.4	HMDB0040822	M-H	C10H20O7	2.0117833333333	-	neg	251.11367553689	0.007972938717167	47.5	146763-54-4
neg_1831	Beclomethasone	metab_33058	B(ii)	0	42.9	HMDB0248924	M-H	C22H29ClO5	2.0224166666667	_;C06842	neg	407.16706448328	0.031945278293641	40.6	4419-39-0;_
neg_1856	Tyr-Ser	metab_33083	B(i)	87.6	0	HMDB0029114	M-H	C12H16N2O5	2.04365	-	neg	267.0987762	0.009325789274709	56.8	-
neg_1867	Asn-Asn-Ile	metab_33094	B(i)	54.4	0	-	M+Hac-H	C14H25N5O6	2.0649333333333	-	neg	418.1942994	0.038036319612079	47.3	-
neg_1875	Biotripyrrin-B	metab_33102	B(ii)	0	42.6	HMDB0003324	M+FA-H	C25H27N3O6	2.07555	-	neg	510.18414416129	0.012211576	45.3	158598-18-6
neg_1877	Val-Glu-Asp	metab_33104	B(i)	39.8	0	-	M-H2O-H	C14H23N3O8	2.0861833333333	-	neg	342.12832925372	0.018902392026667	44.8	-
neg_1880	Dhurrin 6'-Glucoside	metab_33107	B(ii)	0	79.5	HMDB0029776	M+Hac-H	C20H27NO12	2.0861833333333	-	neg	532.1664243	0.018387402768275	54.3	-

neg_1881	Gly-Leu-Ser	metab_33108	B(i)	92.4	0	-	M-H	C11H21N3O5	2.0970833333333	-	neg	274.14097578401	0.012734420311189	57.6	-
neg_1883	13:4+4O Fatty Acyl Hexoside	metab_33110	B(i)	81.9	0	-	M+FA-H	C19H28O10	2.0970833333333	-	neg	461.165386	0.091589116680977	54.7	-
neg_1887	Calystegin A3	metab_33114	B(ii)	0	43.8	HMDB0038593	2M+FA-H	C7H13NO3	2.1131166666667	C10850;_	neg	363.17732285425	0.039374079013199	47.7	131580-36-4
neg_1894	Cichorioside D	metab_33121	B(ii)	0	72.4	HMDB0302051	M-H2O-H	C27H38O13	2.1344833333333	-	neg	551.21042665307	0.19741373949062	50.1	-
neg_1895	Asn-Tyr	metab_33122	B(i)	90.2	0	HMDB0028743	M-H	C13H17N3O5	2.1398333333333	-	neg	294.10972366092	0.016571532797094	56.9	-
neg_1899	Cinnzeylanol	metab_33126	B(ii)	0	41.9	HMDB0036010	M+FA-H	C20H32O7	2.1505	-	neg	429.21052796514	0.29721642027399	44.9	62394-04-1
neg_1902	Arg-Gln-Ile	metab_33129	B(i)	68.9	0	-	M-H	C17H33N7O5	2.1611833333333	-	neg	414.24717726386	0.03063778963811	52.6	-
neg_1905	Glycyl-Tyrosine	metab_33132	B(ii)	0	49	HMDB0304778	M-H	C11H14N2O4	2.1665	-	neg	237.08818379084	0.007693609519023	49	-
neg_1912	Lysosulfatide	metab_33139	B(ii)	0	41.7	HMDB0254273	M+Cl	C24H47NO10S	2.18785	-	neg	576.26369530844	0.1561632746874	44.6	-
neg_1915	Phe-Ser	metab_33142	B(i)	63.3	0	HMDB0029004	M-H	C12H16N2O4	2.2038333333333	-	neg	251.10389266766	0.005982238018878	51.9	16053-39-7
neg_1917	7-Methylguanosine	metab_33144	B(ii)	0	66.1	HMDB0001107	M+Na-2H	C11H16N5O5+	2.2092	-	neg	319.09136690985	0.007768050631326	51.5	20244-86-4
neg_1919	Arg-Ser-Ile	metab_33146	B(i)	82.7	0	-	M-H	C15H30N6O5	2.2145333333333	-	neg	373.22081933428	0.060755010743993	54.5	-
neg_1920	L-Coprine	metab_33147	B(ii)	0	47.9	HMDB0034266	2M-H	C8H14N2O4	2.2145333333333	C08271	neg	403.18396585817	0.052127624362203	48.1	58919-61-2
neg_1922	Asp-Leu-Ser	metab_33149	B(i)	39.7	0	-	M-H	C13H23N3O7	2.2252333333333	-	neg	332.14650518344	0.030144920914312	46.3	-
neg_1933	2-Methylsuccinic Acid	metab_33160	B(i)	41.8	0	HMDB0001844	M+Na-2H	C5H8O4	2.3428	-	neg	153.01698284257	0.089131206933664	47	2174-58-5
neg_1938	Gly-Ile-Asp	metab_33165	B(i)	63.2	0	-	M-H	C12H21N3O6	2.3693	-	neg	302.13594794414	0.088294726181151	51.1	-
neg_1939	4-(7-Hydroxy-6,7-Dihydro-5H-Cyclopenta[D]Pyrimidin-4-Ylamino)Benzonitrile	metab_33166	B(ii)	0	46.1	HMDB0244317	2M-H	C14H12N4O	2.3852333333333	-	neg	503.19969720295	0.04976463575402	46.7	-
neg_1940	Phenylalanyl-Gamma-Glutamate	metab_33167	B(ii)	0	56	HMDB0029009	M-H	C14H19N3O4	2.3958333333333	-	neg	292.13043893835	0.009769566131657	50.2	-
neg_1953	Ala-Ile-Asp	metab_33180	B(i)	72.4	0	-	M-H	C13H23N3O6	2.4810666666667	-	neg	316.15152776439	0.011276261821143	52.6	-
neg_1957	Thr-Tyr	metab_33184	B(i)	93.5	0	HMDB0029073	M-H	C13H18N2O5	2.4970333333333	-	neg	281.11460982623	0.016579739020282	57.5	145295-02-9
neg_1965	(R)-2,3-Dihydroxy-3-Methylvalerate	metab_33192	B(ii)	0	45	HMDB0012140	M-H	C6H12O4	2.60395	C06007	neg	147.06638947356	0.013372776494902	48.7	-;562-43-6
neg_1970	Gly-Gly-Tyr	metab_33197	B(i)	65.5	0	-	M-H	C13H17N3O5	2.6412	-	neg	294.10976943865	0.16971850023162	49.9	-
neg_1974	Asp-Tyr	metab_33201	B(i)	77.3	0	HMDB0028765	M-H	C13H16N2O6	2.6572333333333	-	neg	295.09374851633	0.035646161532907	54.1	-
neg_1979	(S)-A-Amino-2,5-Dihydro-5-Oxo-4-Isoxazolepropanoic Acid N2-Glucoside	metab_33206	B(ii)	0	70.6	HMDB0029404	M+Cl	C12H18N2O9	2.6893333333333	-	neg	369.06811253913	0.052963631252958	51.5	29790-46-3
neg_1980	Ala-Met-Ser	metab_33207	B(i)	80.4	0	-	M-H	C11H21N3O5S	2.6946333333333	-	neg	306.11301598121	0.017746703451982	54	-
neg_1985	Gln-Ile-Asp	metab_33212	B(i)	70.8	0	-	M-H	C15H26N4O7	2.7374333333333	-	neg	373.17297561713	0.015393350373027	52.8	-
neg_1988	Thr-Tyr-Asn	metab_33215	B(i)	69.9	0	-	M-H	C17H24N4O7	2.74275	-	neg	395.15498875287	0.085573542910218	49.6	-
neg_1991	Vigabatrin	metab_33218	B(ii)	0	57	HMDB0015212	M+Hac-H	C6H11NO2	2.7533666666667	C07500	neg	188.0929046	0.023073911500395	51.1	60643-86-9
neg_1996	Gibberellin A20 13-Glucoside	metab_33223	B(ii)	0	61.4	HMDB0033412	M-H	C25H34O10	2.7533666666667	-	neg	493.20305476632	0.11481193638938	48.2	73607-09-7
neg_2012	Ala-Arg-Ile	metab_33239	B(i)	86.2	0	-	M-H	C15H30N6O4	2.7905833333333	-	neg	357.22559875016	0.011692838395698	55.6	-
neg_2015	Asn-Arg-Ala-Ile	metab_33242	B(ii)	0	71	HMDB0304797	M-H	C19H36N8O6	2.8171833333333	-	neg	471.26858318449	0.008687121550730	53.1	-
neg_2022	N(G)-Nitroarginine-4-Nitroanilide	metab_33249	B(ii)	0	47	HMDB0247526	M+Cl	C12H17N7O5	2.8385166666667	-	neg	374.10035680804	0.038382153591811	46.2	-
neg_2025	L-Phenylalanine	metab_33252	B(i)	99.6	0	HMDB0000159;PW_C000104	M-H	C9H11NO2	2.8494166666667	C00079	neg	164.07174559385	0.009090013817348	57.9	63-91-2
neg_2038	Indican	metab_33265	B(ii)	0	40.6	HMDB0061755	M-H2O-H	C14H17NO6	2.8760166666667	C08481;_	neg	276.08549770741	0.085015828190481	45.8	487-60-5;_
neg_2051	Cinncassiol D2 Glucoside	metab_33278	B(ii)	0	70.2	HMDB0034679	M+FA-H	C26H42O11	2.9772666666667	-	neg	575.26907297107	0.017994044473144	50.8	-
neg_2052	Methionyl-Lysine	metab_33279	B(ii)	0	54.7	HMDB0028978	M+Cl	C11H23N3O3S	2.98795	-	neg	312.11791633414	0.03196128295499	46.3	-
neg_2055	Flobufen	metab_33282	B(ii)	0	41	HMDB0252307	M-H	C17H14F2O3	2.9986	-	neg	303.08344102369	0.021862248739418	46.1	-
neg_2066	Valyl-Prolyl-Glycyl-Valyl-Glycine	metab_33293	B(ii)	0	68.1	HMDB0259877	M+Na-2H	C19H33N5O6	3.0416166666667	-	neg	448.21773563987	0.027126666619767	52.3	-
neg_2076	Ser-Gly-Leu	metab_33303	B(i)	78.4	0	-	M-H	C11H21N3O5	3.0789666666667	-	neg	274.1410225	0.02035699194395	54.4	-
neg_2085	Ala-Leu	metab_33312	B(i)	95.3	0	HMDB0028691	M-H	C9H18N2O3	3.095	-	neg	201.12459669161	0.005188373818622	58.3	3303-34-2
neg_2086	Clopidogrel Acyl Glucuronide	metab_33313	B(ii)	0	75.8	HMDB0259598	M+FA-H	C21H22ClNO8	3.095	-	neg	528.07507713793	0.08312250874972	47	-
neg_2087	Ser-Thr-Ile	metab_33314	B(i)	80.1	0	-	M-H	C13H25N3O6	3.1003	-	neg	318.16732145029	0.04890983804234	54.2	-
neg_2093	Netilmicin	metab_33320	B(ii)	0	57.4	HMDB0015090	M-H2O-H	C21H41N5O7	3.1056333333333	C07657	neg	456.28251605496	0.072202508404403	49.6	56391-56-1
neg_2108	Tylosin	metab_33335	B(ii)	0	57.7	HMDB0034108	M-H2O-H	C46H77NO17	3.1163333333333	C01457	neg	896.49614419173	0.008640997075203	47.8	1401-69-0;

neg_2109	Arg-Ile-Leu	metab_33336	B(i)	65.3	0	-	M-H	C18H36N6O4	3.1216833333333	-	neg	399.27265330002	0.017526709799729	52	-
neg_2114	Procyanidin Dimer B7	metab_33341	B(ii)	0	55.2	HMDB0304725	M+Na-2H	C20H22O8	3.1270333333333	-	neg	411.10223800804	0.076980968521677	46.1	-
neg_2117	2-(4-(2-Carboxyethyl)Phenethylamino)-5'-N-Ethylcarboxamidoadenosine	metab_33344	B(ii)	0	69.8	HMDB0249866	M+Hac-H	C23H29N7O6	3.1270333333333	-	neg	558.23579410594	0.012453551712247	51.9	-
neg_2118	Ogyline	metab_33345	B(ii)	0	61	HMDB0254813	2M+FA-H	C20H22O2	3.1270333333333	-	neg	633.32138278439	0.006218017651510	51	-
neg_2121	Benazepril	metab_33348	B(ii)	0	66.7	HMDB0014682	M+Cl	C24H28N2O5	3.1323666666667	C06843	neg	459.17210506143	0.026660126997333	49.1	86541-75-5
neg_2122	Physalolactone	metab_33349	B(ii)	0	78.9	HMDB0034333	M+Hac-H	C28H39ClO8	3.1323666666667	-	neg	597.25231703516	0.038325221122107	47.1	71339-25-8;
neg_2126	N-[(3A,5B,7A)-3-Hydroxy-24-Oxo-7-(Sulfooxy)Cholan-24-Yl]-Glycine	metab_33353	B(ii)	0	75.3	HMDB0002496	M+Cl	C26H43NO8S	3.1377	C01324	neg	564.24258927469	0.019433396220984	51.9	67030-55-1
neg_2132	Candoxatrilat	metab_33359	B(ii)	0	48.5	HMDB0249582	2M+FA-H	C20H33NO7	3.1483	C11721;_	neg	843.44450300395	0.048082786084018	46.1	123898-42-0;_
neg_2135	N-Acetyl-Glucosamine 1-Phosphate	metab_33362	B(ii)	0	56.5	HMDB0001367	M+Hac-H	C8H16NO9P	3.15365	C04256	neg	360.0715402	0.03562336990056	49.7	6866-69-9
neg_2142	Kanokoside C	metab_33369	B(ii)	0	65.5	HMDB0035305	M+FA-H	C27H42O17	3.1589333333333	_;C17430	neg	683.24568530445	0.081139085196676	49.2	-;64703-87-3
neg_2144	Chrysoeriol 7-O-(6''-Malonyl-Apiosyl-Glucoside)	metab_33371	B(ii)	0	92.4	HMDB0301684	M+Cl	C30H32O18	3.1589333333333	-	neg	715.12238437453	0.002921104876326	53.1	-
neg_2149	Ent-6R,16Boh,17-Trihydroxy-7-Oxo-6,7-Seco-19,6-Kauranolide 6-O-Glucoside	metab_33376	B(ii)	0	88	HMDB0038546	M+Hac-H	C26H40O11	3.1642833333333	-	neg	587.27060587365	0.050771634299578	56	132242-50-3
neg_2157	Belotecan	metab_33384	B(ii)	0	47.4	HMDB0248935	M+Cl	C25H27N3O4	3.1752	-	neg	468.16595052435	0.056802965906206	44.8	-
neg_2161	Eribulin	metab_33388	B(ii)	0	54.4	HMDB0251920	M-H	C40H59NO11	3.1752	-	neg	728.40594397227	0.009983367818028	47.3	-
neg_2170	Betanin	metab_33397	B(ii)	0	54	HMDB0029408	M+Hac-H	C24H26N2O13	3.1805	_;C08540	neg	609.15792261057	0.27585940901293	48	7659-95-2
neg_2176	Phlorizin	metab_33403	B(ii)	0	52	HMDB0036634	M+Na-2H	C21H24O10	3.1858	C01604	neg	457.10777603597	0.022546416610549	46.7	60-81-1
neg_2198	Tyr Val Ser Arg	metab_33425	B(i)	71	0	-	M-H	C23H37N7O7	3.1965	-	neg	522.26816405346	0.010836682758499	53.5	-
neg_2200	Leuphasyl	metab_33427	B(ii)	0	72.2	HMDB0247171	M+Na-2H	C29H39N5O7	3.1965	-	neg	590.2555246	0.096671759798694	50.5	-
neg_2210	26-Deoxyactein	metab_33437	B(ii)	0	44.1	HMDB0245709	M-H2O-H	C37H56O10	3.2018166666667	-	neg	641.37434538797	0.032660505590235	45.3	-
neg_2218	Luminespib	metab_33445	B(ii)	0	72.6	HMDB0254202	M+FA-H	C26H31N3O5	3.2071666666667	-	neg	510.22073182041	0.007159089371928	51	-
neg_2219	Kukoamine C	metab_33446	B(ii)	0	44.3	HMDB0060526	M+FA-H	C28H42N4O6	3.2071666666667	C17617	neg	575.30408021198	0.020607513027722	44.9	-;_
neg_2222	Leukotriene C4	metab_33449	B(i)	81	0	HMDB0001198;PW_C000933	M+FA-H	C30H47N3O9S	3.2071666666667	C02166	neg	670.29682154096	0.10494167487038	51.7	72025-60-6
neg_2224	Dialdehyde	metab_33451	B(ii)	0	53.2	HMDB0251125	2M-H	C21H24N2O4	3.2071666666667	_;C11678	neg	735.34114307889	0.037011122442129	49	85955-83-5;_
neg_2237	Medicagenic Acid 3-O-Beta-D-Glucoside	metab_33464	B(ii)	0	65.2	HMDB0034552	M+Na-2H	C36H56O11	3.2125166666667	-	neg	685.35264174632	0.087616168743958	48.3	49792-23-6
neg_2239	Salicyl Alcohol	metab_33466	B(i)	88.6	0	HMDB0059709	M-H	C7H8O2	3.2178166666667	C02323	neg	123.04525496497	0.066414877839197	56.5	1990/1/7
neg_2241	3'-(O-Methyl)Inosine	metab_33468	B(i)	70.9	0	-	M-H	C11H14N4O5	3.2178166666667	-	neg	281.08897651079	0.010861463038649	52.2	-
neg_2261	Dehydroandrographolide Succinate	metab_33488	B(ii)	0	57.5	HMDB0250944	M+Cl	C28H36O10	3.2231166666667	-	neg	567.20369957669	0.014011178733996	48	-
neg_2268	Leu-Ser	metab_33495	B(i)	96.8	0	HMDB0028938	M-H	C9H18N2O4	3.2284166666667	-	neg	217.11947057968	0.004839426737842	59	-
neg_2269	Val-Met	metab_33496	B(i)	87.3	0	HMDB0259742	M-H	C10H20N2O3S	3.2284166666667	-	neg	247.11228744838	0.006049798038435	55.5	-
neg_2270	Pro-Leu-Lys	metab_33497	B(i)	56.1	0	-	M-H	C17H32N4O4	3.2284166666667	-	neg	355.23522053145	0.047354301251941	49.5	-
neg_2291	Deoxyadenosine Monophosphate	metab_33518	B(ii)	0	69	HMDB0000905	M+Hac-H	C10H14N5O6P	3.2337	C00360	neg	390.0822875	0.070243930302164	52.3	653-63-4
neg_2292	Licoricone	metab_33519	B(ii)	0	41.2	HMDB0029515	M+Hac-H	C22H22O6	3.2337	C17765	neg	441.15196747778	0.016343676416814	44.9	51847-92-8;
neg_2293	Isofloxythepin	metab_33520	B(ii)	0	51.1	HMDB0253639	M+Hac-H	C23H29FN2OS	3.2337	-	neg	459.20953248716	0.040638236388747	47.2	-
neg_2294	Alpha-Trisaccharide	metab_33521	B(ii)	0	64.8	HMDB0006595	M-H2O-H	C20H37NO14	3.2337	-	neg	496.20247782251	0.036751223752792	49.9	49777-13-1
neg_2308	Asn-Ala-Tyr	metab_33535	B(i)	91.8	0	-	M-H	C16H22N4O6	3.2390166666667	-	neg	365.14671620908	0.014251706907988	56.9	-
neg_2313	Asn Ile Thr Glu	metab_33540	B(i)	70.1	0	-	M-H	C19H33N5O9	3.2390166666667	-	neg	474.22071737437	0.008813551881019	53.8	-
neg_2320	Arg-Thr-Lys-Arg	metab_33547	B(ii)	0	68.5	HMDB0304796	M+FA-H	C22H45N11O6	3.2390166666667	-	neg	604.35658861551	0.065906507525633	51.6	-
neg_2322	Pg(7:0/18:5)	metab_33549	B(i)	71.6	0	-	M+FA-H	C31H51O10P	3.2390166666667	-	neg	659.31432479097	0.007518275259292	50.2	-
neg_2330	Gly-Arg-Gly-Asp-Ser	metab_33557	B(ii)	0	43	HMDB0252824	M+Cl	C17H30N8O9	3.2443	-	neg	525.1840578	0.037602873878759	47.3	-
neg_2331	Stachyoside A	metab_33558	B(ii)	0	69.3	HMDB0039092	M+Na-2H	C21H34O15	3.2443	-	neg	547.16609535668	0.11121536630539	52.4	131862-10-7
neg_2332	Tyrosylhydroxyproline	metab_33559	B(ii)	0	52.9	HMDB0029106	2M-H	C14H18N2O5	3.2443	-	neg	587.23224511961	0.030651283169444	47.2	936346-34-8
neg_2333	Glycylserylprolylmethionylphenylalanylvalinamide	metab_33560	B(ii)	0	60.4	HMDB0033242	M-H2O-H	C29H45N7O7S	3.2443	-	neg	616.29520572819	0.024454215254371	48.1	115525-98-9
neg_2340	3-Oxo-Alpha-Ionol 9-[Apiosyl-(1->6)-Glucoside]	metab_33567	B(ii)	0	68.2	HMDB0040672	M+Hac-H	C24H38O11	3.2496333333333	-	neg	561.25280025941	0.021286689074988	51.6	143363-62-6
neg_2341	Mascaroside	metab_33568	B(ii)	0	61.4	HMDB0035603	M+Hac-H	C26H36O11	3.2496333333333	C09132	neg	583.23446999058	0.020299765143671	48.2	55465-97-9

neg_2345	Thr-Leu	metab_33572	B(i)	95	0	HMDB0259044	M-H	C10H20N2O4	3.2549833333333	-	neg	231.13515115304	0.025698983106241	58.1	-
neg_2346	Asn-Ile-Leu	metab_33573	B(i)	38	0	-	M-H	C16H30N4O5	3.2549833333333	-	neg	357.21450118784	0.012389732798338	46.3	-
neg_2350	Cadabicine Methyl Ether	metab_33577	B(ii)	0	71.4	HMDB0039857	M+Cl	C26H31N3O4	3.2549833333333	-	neg	484.20309057519	0.086388038270079	50.2	-
neg_2362	Calophymembranside B	metab_33589	B(i)	42.8	0	-	M+FA-H	C20H30O12	3.2603166666667	-	neg	507.16897971721	0.052433898709411	46.1	-
neg_2363	Morellin	metab_33590	B(ii)	0	49.7	HMDB0030794	M-H	C33H36O7	3.2603166666667	C10085;_	neg	543.24228076781	0.032339588087157	45.5	1183-12-6
neg_2384	1-[3,4-Dihydroxy-5-(Hydroxymethyl)Oxolan-2-Yl]-4-Hydroxyhydropyridin-2-One	metab_33611	B(ii)	0	56.1	HMDB0243807	M-H2O-H	C10H13NO6	3.271	-	neg	224.05417683641	0.011421046027376	46.8	-
neg_2386	Thr-Ala-Ile	metab_33613	B(i)	74.5	0	-	M-H	C13H25N3O5	3.271	-	neg	302.17231053968	0.011401397339469	53.3	-
neg_2396	Biocytin	metab_33623	B(ii)	0	63.1	HMDB0003134	2M+FA-H	C16H28N4O4S	3.271	C05552	neg	789.36504493412	0.027943850773582	50.7	576-19-2
neg_2397	Rpr112698	metab_33624	B(ii)	0	64.5	HMDB0060870	M+Hac-H	C44H55NO14	3.271	-	neg	880.38048182439	0.029843711800134	49.9	-
neg_2408	Dgdg(O-18:5/5:0)	metab_33635	B(i)	44.1	0	-	M+Hac-H	C38H62O14	3.27635	-	neg	801.42270948661	0.001382932575447	43.4	-
neg_2412	Lysylmethionine	metab_33639	B(ii)	0	47.2	HMDB0028957	M+Cl	C11H23N3O3S	3.2817	-	neg	312.11792728483	0.033803141943069	45.3	97729-52-7
neg_2415	Gln-Thr-Leu	metab_33642	B(i)	88.1	0	-	M-H	C15H28N4O6	3.2817	-	neg	359.19376238866	0.010303543710273	54.9	-
neg_2434	Agar	metab_33661	B(ii)	0	43.5	HMDB0248056	M-H2O-H	C14H24O9	3.2923833333333	C08815;_	neg	317.12566668011	0.014671269469206	47	9002-18-0;_
neg_2439	Levomefolic Acid	metab_33666	B(ii)	0	43.1	HMDB0254053	M+Hac-H	C20H25N7O6	3.2923833333333	-	neg	518.19988493098	0.03273649836465	46.5	-
neg_2441	7-Hydroxyolanzapine	metab_33668	B(ii)	0	42.7	HMDB0060958	2M-H	C17H20N4OS	3.2923833333333	-	neg	655.2695602	0.072457544172742	44.8	-
neg_2445	Arg-His-Phe-Trp-Gln-Gln	metab_33672	B(ii)	0	67	HMDB0248576	M-H	C42H56N14O9	3.2923833333333	-	neg	899.42333564356	0.020908854000583	49.6	-
neg_2447	Gln-Val-Ala	metab_33674	B(i)	80.5	0	-	M-H	C13H24N4O5	3.2977333333333	-	neg	315.16748493079	0.040610504768313	54	-
neg_2450	Glu-Glu-Ile	metab_33677	B(i)	72.9	0	-	M-H	C16H27N3O8	3.2977333333333	-	neg	388.17278681721	0.063171796197931	51.3	-
neg_2453	Cepharanthine	metab_33680	B(ii)	0	50.8	HMDB0249813	M+FA-H	C37H38N2O6	3.2977333333333	C09391;_	neg	651.27472550382	0.019732570450088	47.9	481-49-2;_
neg_2462	N-Acetylcytidine	metab_33689	B(i)	92	0	HMDB0005923	M-H	C11H15N3O6	3.3030833333333	-	neg	284.08890053627	0.05252171015776	55.9	3768-18-1
neg_2476	Monomethyl Phthalate	metab_33703	B(i)	68	0	HMDB0002130	M-H	C9H8O4	3.30845	-	neg	179.03507219215	0.020901658941914	53.2	4376-18-5
neg_2483	Annomuricatin A	metab_33710	B(ii)	0	85.2	HMDB0303181	M+Na-2H	C27H38N6O7	3.30845	-	neg	579.25230714274	0.008128475811686	54.3	-
neg_2489	Pi(Pgf1Alpha/18:2(9Z,12Z))	metab_33716	B(ii)	0	47.9	HMDB0276748	M+Cl	C47H83O16P	3.30845	-	neg	969.51193801575	0.041304756598228	46.4	-
neg_2493	Pro-Met-Ser	metab_33720	B(i)	74.9	0	-	M-H	C13H23N3O5S	3.3137833333333	-	neg	332.12871504694	0.017032770375951	53.3	-
neg_2509	Prenyl Apiosyl-(1->6)-Glucoside	metab_33736	B(ii)	0	42.9	HMDB0031956	M-H	C16H28O10	3.3191333333333	-	neg	379.16238338625	0.043013110055794	46.2	198832-70-1
neg_2525	Gly-Ala-Ile	metab_33752	B(i)	49.3	0	-	2M-H	C11H21N3O4	3.3244833333333	-	neg	517.29965851704	0.044569606742482	48	-
neg_2527	Gamma-Glutamylproline	metab_33754	B(ii)	0	49.7	HMDB0029157	M+Hac-H	C10H16N2O5	3.3298333333333	-	neg	303.11992237486	0.051252471178928	49.1	53411-63-5
neg_2531	Leu Arg Val Glu	metab_33758	B(i)	69.7	0	-	M-H	C22H41N7O7	3.3298333333333	-	neg	514.29959389422	0.016478658191082	49	-
neg_2535	4-Hydroxymandelic Acid	metab_33762	B(i)	79.3	0	HMDB0000822	M-H	C8H8O4	3.3351333333333	C11527	neg	167.03507949846	0.06906273642288	55	1198-84-1
neg_2542	Isoleucine Glutamate	metab_33769	B(ii)	0	55.7	HMDB0253650	2M-H	C11H20N2O6	3.3351333333333	-	neg	551.25544552396	0.071172401352734	50.3	-
neg_2548	Motexafin	metab_33775	B(ii)	0	69.3	HMDB0254902	M+Cl	C48H67N5O10	3.3351333333333	-	neg	908.45977446412	0.050073226428717	49.4	-
neg_2549	Gln-Leu	metab_33776	B(i)	84.5	0	HMDB0028801	M-H	C11H21N3O4	3.3407166666667	-	neg	258.14603765587	0.012555304255874	56.2	34027-65-1
neg_2557	Sulfolithocholylglycine	metab_33784	B(ii)	0	44.9	HMDB0002639	M+Cl	C26H43NO7S	3.3407166666667	C11301	neg	548.24754814322	0.011824316311499	46	15324-64-8
neg_2573	Aconine	metab_33800	B(ii)	0	54.7	HMDB0247960	M+FA-H	C25H41NO9	3.3460666666667	C19990;_	neg	544.27353121834	0.030931907687337	47.3	509-20-6;_
neg_2596	Varespladib	metab_33823	B(ii)	0	56.9	HMDB0259766	M+Cl	C21H20N2O5	3.3567666666667	-	neg	415.11005844068	0.042826731007333	46.9	-
neg_2597	2-Methoxy-Estradiol-17B 3-Glucuronide	metab_33824	B(ii)	0	67.4	HMDB0006765	M-H2O-H	C25H34O9	3.3567666666667	C11131	neg	459.19851608213	0.033686687592283	45.2	-
neg_2624	Ser-Ala-Ile	metab_33851	B(i)	66.8	0	-	M-H	C12H23N3O5	3.3673833333333	-	neg	288.15664176476	0.050115634553025	52	-
neg_2629	(R)-1-O-[B-D-Apiofuranosyl-(1->2)-B-D-Glucopyranoside]-1,3-Octanediol	metab_33856	B(ii)	0	45.7	HMDB0032798	M+FA-H	C21H40O9	3.3673833333333	-	neg	481.26674651634	0.092305307018773	47.4	-
neg_2636	Nodularin-R	metab_33863	B(ii)	0	95.1	HMDB0255693	M+Cl	C40H58N8O10	3.3673833333333	-	neg	845.40129144741	0.033397671528385	54.7	-
neg_2662	Tryptophyl-Serine	metab_33889	B(ii)	0	55.9	HMDB0029092	M-H	C14H17N3O4	3.3834	-	neg	290.11478077653	0.075322097427831	49.5	-
neg_2680	Dioscin	metab_33907	B(ii)	0	75.5	HMDB0251433	M+Hac-H	C45H72O16	3.3834	_;C08897	neg	927.50225597932	0.025588297421832	51.5	19057-60-4;_;
neg_2681	(Z)-[(4-Hydroxyphenyl)Acetaldehyde Oxime]	metab_33908	B(ii)	0	62.1	HMDB0304004	2M-H	C8H9NO2	3.3887	C04353	neg	301.11715792044	0.036933795435015	49.7	-
neg_2682	O-Desmethylvenlafaxine Glucuronide	metab_33909	B(ii)	0	50.8	HMDB0061172	M-H2O-H	C22H33NO8	3.3887	-	neg	420.19869181253	0.0414244064624	45.4	1021933-98-1;_
neg_2683	Armillane	metab_33910	B(ii)	0	44.5	HMDB0035779	M+Na-2H	C23H32O7	3.3887	-	neg	441.18801649246	0.010749056742746	47.6	126006-69-7

neg_2696	Isoliquiritin	metab_33923	B(i)	49.8	0	HMDB0037318;LMPK12120021	M-H	C21H22O9	3.39405	C16978	neg	417.11529382043	0.021515128683224	46.7	5041-81-6;
neg_2697	Gardenoside	metab_33924	B(ii)	0	65.1	HMDB0252642	M+Cl	C17H24O11	3.39405	C09779;_	neg	439.09732847285	0.052752941938865	49.9	24512-62-7;_
neg_2712	Americine	metab_33939	B(ii)	0	73.4	HMDB0034441	M+Hac-H	C31H39N5O4	3.3994	C09996	neg	604.31084213758	0.074890506076992	49.6	18867-84-0
neg_2722	L-Alanyl-L-Valine	metab_33949	B(i)	64.1	0	HMDB0028700	2M-H	C8H16N2O3	3.4046833333333	-	neg	375.22510065026	0.083945597938737	52	3303-45-5
neg_2727	Tripeptide	metab_33954	B(ii)	0	71.5	HMDB0259261	M+FA-H	C24H35N7O8	3.4046833333333	-	neg	594.25416526181	0.0240133655998	51.9	-
neg_2730	Tryptophyl-Glutamine	metab_33957	B(ii)	0	46.8	HMDB0029081	2M-H	C16H20N4O4	3.4046833333333	-	neg	663.28406686451	0.097567100561779	34.6	-
neg_2733	Capsicoside C3	metab_33960	B(ii)	0	63	HMDB0040950	M-H	C44H70O17	3.4046833333333	-	neg	869.45991813486	0.074683153098725	48.1	125456-10-2
neg_2735	Thr-Ile	metab_33962	B(i)	98	0	HMDB0029064	M-H	C10H20N2O4	3.4100333333333	-	neg	231.13515675981	0.02014435859612	59	129050-49-3
neg_2749	Acotiamide	metab_33976	B(ii)	0	63.7	HMDB0247965	M-H2O-H	C21H30N4O5S	3.4153666666667	C14127	neg	431.17868271329	0.01830516074379	48.2	185106-16-5;_
neg_2759	Gln-Ala-Leu	metab_33986	B(i)	36.2	0	-	M-H	C14H26N4O5	3.4207	-	neg	329.18320787223	0.010664601073602	46.1	-
neg_2760	Ile-Asp-Ser	metab_33987	B(i)	57.8	0	-	M-H	C13H23N3O7	3.4207	-	neg	332.1463862	0.058564934216066	50	-
neg_2761	N-Desmethyltamoxifen	metab_33988	B(ii)	0	65.9	HMDB0013866	M+Cl	C25H27NO	3.4207	C16546	neg	392.17907521677	0.05362808264643	49.8	31750-48-8
neg_2765	4-Hydroxyretinoic Acid Glucuronide	metab_33992	B(ii)	0	73.6	HMDB0061688	M+Hac-H	C26H36O9	3.4207	-	neg	551.2463811	0.017073250363407	52	-
neg_2767	Elexacaftor/Ivacaftor/Tezacaftor	metab_33994	B(ii)	0	47.9	HMDB0304881	M-H2O-H	C26H34F3N7O4S	3.4207	-	neg	578.22257202418	0.031922667958612	43.7	-
neg_2775	N,N-Dimethylguanosine	metab_34002	B(i)	91.9	0	-	M-H	C12H17N5O5	3.4260333333333	-	neg	310.11575629831	0.055192577477293	56.5	-
neg_2776	Ile-Ile-Asp	metab_34003	B(i)	45.2	0	-	M-H	C16H29N3O6	3.4260333333333	-	neg	358.19845364969	0.042332961984411	48	-
neg_2794	Tyr-Pro	metab_34021	B(i)	79.8	0	HMDB0029113	M-H	C14H18N2O4	3.4313833333333	-	neg	277.11956218252	0.009369177915664	55	-
neg_2799	Fluprostenol Serinol Amide	metab_34026	B(i)	40.1	0	-	M+FA-H	C26H36F3NO7	3.4313833333333	-	neg	576.239701	0.027770841422167	42.9	-
neg_2805	Meta-O-Dealkylated Flecainide Lactam	metab_34032	B(ii)	0	50.5	HMDB0060832	M-H	C15H17F3N2O	3.4366833333333	-	neg	345.10715855663	0.016570253784536	47.8	-
neg_2854	Ala-Thr-Ile	metab_34081	B(i)	93.3	0	-	M-H	C13H25N3O5	3.4580333333333	-	neg	302.17231491407	0.056137885049672	57.3	-
neg_2869	Bpd-Ma	metab_34096	B(ii)	0	40.1	HMDB0249026	M+Cl	C42H44N4O8	3.4580333333333	-	neg	767.28593754619	0.024365466003098	44.2	-
neg_2882	Pc(O-16:4/5:0)	metab_34109	B(i)	59.1	0	-	M+FA-H	C29H52NO7P	3.4633833333333	-	neg	602.35104565364	0.04981605766162	47.4	-
neg_2885	Carminomycin Ii	metab_34112	B(ii)	0	53.2	HMDB0249683	M-H	C33H41NO13	3.4633833333333	-	neg	658.2453647	0.056847728871189	47.5	-
neg_2889	Val-Ile-Asp	metab_34116	B(i)	79.3	0	-	M-H	C15H27N3O6	3.4687166666667	-	neg	344.18282811519	0.031080985913054	53.4	-
neg_2899	Ansamitocin P-3	metab_34126	B(ii)	0	70.7	HMDB0248452	M-H	C32H43ClN2O	3.4687166666667	C12045;_	neg	633.26413098442	0.011859622064236	44.8	66584-72-3;_
neg_2904	Asn-Gln-Leu	metab_34131	B(i)	46.1	0	-	M-H	C15H27N5O6	3.4740666666667	-	neg	372.1889467	0.019010110360734	47.3	-
neg_2917	Glu-Ala-Ile	metab_34144	B(i)	73.2	0	-	M-H	C14H25N3O6	3.4794	-	neg	330.16731351107	0.054637493923039	52.4	-
neg_2922	Leu Gln Tyr Asp	metab_34149	B(i)	61.8	0	-	M-H	C24H35N5O9	3.4794	-	neg	536.23664918469	0.073668134754056	50.1	-
neg_2936	Phe-Val-Asn	metab_34163	B(i)	70.2	0	-	M+FA-H	C18H26N4O5	3.4847	-	neg	423.18647664236	0.037626461554743	51.4	-
neg_2939	Epicylindrospermopsin	metab_34166	B(ii)	0	59.6	HMDB0247246	M+Cl	C15H21N5O7S	3.4847	-	neg	450.08803343378	0.017164882899767	48.3	-
neg_2940	Fluorescein 5-Maleimide	metab_34167	B(ii)	0	40.7	HMDB0252363	M+FA-H	C24H13NO7	3.4847	-	neg	472.07013249001	0.054168548784512	43.7	-
neg_2952	Glu-Leu	metab_34179	B(i)	90.7	0	HMDB0028823	M-H	C11H20N2O5	3.49005	-	neg	259.13005570289	0.027504215273562	57.4	5969-52-8
neg_2955	N-(9-Pentofuranosyl-9H-Purin-6-Yl)Aspartic Acid	metab_34182	B(i)	58.4	0	-	M-H	C14H17N5O8	3.49005	-	neg	382.10062195419	0.018061839080729	50.8	-
neg_2958	Estriol Succinate	metab_34185	B(ii)	0	43.8	HMDB0251979	M+Hac-H	C26H32O9	3.49005	-	neg	547.21648410985	0.016460554761087	46.1	-
neg_2969	Asp-Ile	metab_34196	B(i)	87.8	0	HMDB0028756	M-H	C10H18N2O5	3.4953333333333	-	neg	245.114392	0.042244934052086	56.8	-
neg_2983	Ps(Pgf2Alpha/20:4(8Z,11Z,14Z,17Z))	metab_34210	B(ii)	0	70.2	HMDB0282700	M+Cl	C46H76NO13P	3.4953333333333	-	neg	916.46833703014	0.015888053102158	51.5	-
neg_2987	Ac-Ser-Asp-Lys-Pro-Oh	metab_34214	B(ii)	0	77.6	HMDB0062552	M+FA-H	C20H33N5O9	3.5006833333333	-	neg	532.22624832811	0.011507134167988	55.2	-
neg_2988	2-Pyrrolino-Dox	metab_34215	B(ii)	0	60.2	HMDB0245311	M-H2O-H	C31H33NO11	3.5006833333333	-	neg	576.19025868808	0.012533008874329	47.3	-
neg_3005	Lys-Glu-Leu	metab_34232	B(i)	35.4	0	-	M-H2O-H	C17H32N4O6	3.5114	-	neg	369.21447838752	0.039378882950346	45.1	-
neg_3008	Arg-Phe-Ile	metab_34235	B(i)	47.7	0	-	M-H	C21H34N6O4	3.5114	-	neg	433.25704257622	0.004249221422968	48.5	-
neg_3009	Tyr Thr Ala Glu	metab_34236	B(i)	63.4	0	-	M-H	C21H30N4O9	3.5114	-	neg	481.19391565113	0.012849623251114	51.6	-
neg_3014	Golotimod	metab_34241	B(ii)	0	63.9	HMDB0252908	2M-H	C16H19N3O5	3.5114	-	neg	665.25396865243	0.013468115422788	49	-
neg_3015	Neoacrimarine A	metab_34242	B(ii)	0	48.1	HMDB0040384	M-H	C40H43NO9	3.5114	-	neg	680.28324166261	0.011064284355964	45.8	149301-45-1
neg_3019	N-Succinyl-Leu-Leu-Val-Tyr-7-Amido-4-Methylcoumarin, >=90% (Hplc)	metab_34246	B(ii)	0	76.8	HMDB0258535	M+Cl	C40H53N5O10	3.5114	-	neg	798.35553444688	0.013654868449767	52	-

neg_3035	Glu-Phe-Asp	metab_34262	B(i)	92.5	0	-	M-H	C18H23N3O8	3.5220833333333	-	neg	408.14124104821	0.023016719075653	56.8	-
neg_3040	3-Hydroxymelatonin	metab_34267	B(ii)	0	44.2	HMDB0060742	2M+FA-H	C13H18N2O3	3.5220833333333	-	neg	545.2579048	0.018413123297914	44.7	-
neg_3045	2-[1-(2-Oxo-2-{[3-(Trifluoromethyl)Benzyl]Amino}Ethyl)Cyclohexyl]Acetic Acid	metab_34272	B(i)	37.5	0	-	M-H	C18H22F3NO3	3.5273666666667	-	neg	356.14651114335	0.020562684624512	45	-
neg_3046	Arg-Val-Ile	metab_34273	B(i)	54.8	0	-	M-H	C17H34N6O4	3.5273666666667	-	neg	385.25701678928	0.017146342511783	49.1	-
neg_3047	Gln-Ser-Trp	metab_34274	B(i)	80.1	0	-	M-H	C19H25N5O6	3.5273666666667	-	neg	418.17387732805	0.095472717950481	53.3	-
neg_3062	Folinic Acid	metab_34289	B(ii)	0	49.7	HMDB0001562	M-H	C20H23N7O7	3.5327333333333	C03479	neg	472.15886188557	0.009702140747319	49.3	1958/5/9;68538-85-2
neg_3081	25-Desacetylrifampicin	metab_34308	B(ii)	0	64.8	HMDB0245685	M+Cl	C41H56N4O11	3.5380833333333	-	neg	815.35786045571	0.031321829207213	47.6	-
neg_3083	3-Hydroxy-2,3,4-Trimethyl-5-Oxooxolane-2-Carboxylic Acid	metab_34310	B(i)	42.6	0	-	M-H	C8H12O5	3.5433666666667	-	neg	187.06128549066	0.055560239199228	46.6	-
neg_3084	Ile-Gly-Leu	metab_34311	B(i)	61.5	0	-	M-H	C14H27N3O4	3.5433666666667	-	neg	300.19301068537	0.002703552188424	51.4	-
neg_3093	Leukotriene D4 Methyl Ester	metab_34320	B(i)	37.8	0	-	M-H	C26H42N2O6S	3.5433666666667	-	neg	509.27293447814	0.039275961390812	43.4	-
neg_3114	N-Acetylserotonin Glucuronide	metab_34341	B(ii)	0	68.2	HMDB0060833	M+Hac-H	C18H22N2O8	3.5540166666667	-	neg	453.1515099	0.017511471351411	52.5	-
neg_3115	Prednisolone Hemisuccinate	metab_34342	B(ii)	0	59.4	HMDB0245744	M+Hac-H	C25H32O8	3.5540166666667	-	neg	519.22111004946	0.02545029911808	48.5	-
neg_3120	Gamma-Glutamylfelinylglycine	metab_34347	B(ii)	0	67.7	HMDB0252619	2M-H	C15H27N3O7S	3.5540166666667	-	neg	785.31172061415	0.020673218171408	49.4	-
neg_3127	Deoxycholic Acid 3-Glucuronide	metab_34354	B(ii)	0	77.9	HMDB0002596	M-H2O-H	C30H48O10	3.5593333333333	C03033;_	neg	549.30509647909	0.084669270060382	52.6	72504-58-6;
neg_3129	Canesceol	metab_34356	B(ii)	0	71.7	HMDB0034084	M+Cl	C29H44O11	3.5593333333333	-	neg	603.26237184842	0.087169180089501	50.2	82228-15-7
neg_3142	Asn-Gly-Ile	metab_34369	B(i)	94	0	-	M-H	C12H22N4O5	3.5699666666667	-	neg	301.15190440324	0.021846339235551	55.8	-
neg_3145	Mopidamol	metab_34372	B(ii)	0	42.1	HMDB0254878	M+Cl	C19H31N7O4	3.5699666666667	-	neg	456.20992049341	0.009538237352583	45.6	-
neg_3148	Arg Gly Glu Val Leu	metab_34375	B(i)	52.3	0	-	M-H	C24H44N8O8	3.5699666666667	-	neg	571.32078293927	0.034129332978292	49.2	-
neg_3149	Tyr-Ile-Gly-Ser-Arg	metab_34376	B(ii)	0	67.3	HMDB0259343	M-H	C26H42N8O8	3.5699666666667	-	neg	593.30243857509	0.042600232986978	46.8	-
neg_3150	Physalolactone B 3-Glucoside	metab_34377	B(ii)	0	64.3	HMDB0034201	M-H2O-H	C36H54O11	3.5699666666667	-	neg	643.34232293046	0.021459055304535	47.7	82087-30-7
neg_3156	Hydroxytyrosol	metab_34383	B(i)	93	0	HMDB0005784	M-H	C8H10O3	3.5752666666667	-	neg	153.05585352903	0.062162116820898	58	10597-60-1
neg_3160	Gln-Gln-Ile	metab_34387	B(i)	54.3	0	-	M-H	C16H29N5O6	3.5752666666667	-	neg	386.20439013607	0.04745123083731	48.6	-
neg_3162	Antimycin A	metab_34389	B(ii)	0	61.6	HMDB0248488	M+Cl	C28H40N2O9	3.5752666666667	C11339	neg	583.24813454132	0.095181013162199	48.2	-
neg_3165	Gluten Exorphin B5	metab_34392	B(ii)	0	53.7	HMDB0059795	M-H	C30H38N6O11	3.5752666666667	-	neg	657.25345236821	0.022613901750071	49.9	-
neg_3168	Jubanine B	metab_34395	B(ii)	0	72.2	HMDB0030206	M-H	C43H47N5O6	3.5752666666667	-	neg	728.33827062867	0.058523533609921	48.4	60375-08-8
neg_3178	Stercobilin	metab_34405	B(ii)	0	61.2	HMDB0240259	M+Na-2H	C33H46N4O6	3.5805666666667	_;C05793	neg	615.31090483933	0.045940578064173	46.4	34217-90-8
neg_3180	Elatoside G	metab_34407	B(ii)	0	62.3	HMDB0041347	M+Na-2H	C36H56O11	3.5805666666667	-	neg	685.35405474136	0.019703839033122	48.5	171828-77-6
neg_3183	Asp-Lys-Tyr	metab_34410	B(i)	42.3	0	-	M-H	C19H28N4O7	3.5859166666667	-	neg	423.18632930771	0.026004571222273	45.5	-
neg_3189	Leu-Ser-Pro	metab_34416	B(i)	86	0	-	M-H	C14H25N3O5	3.5912333333333	-	neg	314.17231563338	0.008320915582898	53.9	-
neg_3191	Gln-Phe-Ser	metab_34418	B(i)	69	0	-	M-H	C17H24N4O6	3.5912333333333	-	neg	379.16247400906	0.012935384551772	51.8	-
neg_3221	Ser-Phe	metab_34448	B(i)	96.7	0	HMDB0029046	M-H	C12H16N2O4	3.6071333333333	-	neg	251.10392105851	0.005385863279554	58.5	16875-28-8
neg_3236	(5Z)-(15S)-11Alpha-Hydroxy-9,15-Dioxoprostanoate	metab_34463	B(ii)	0	44.3	HMDB0012481	M+FA-H	C20H32O5	3.6124833333333	C04671	neg	397.22071794091	0.015531431183252	45.9	-
neg_3243	Casokefamide	metab_34470	B(ii)	0	59.1	HMDB0249695	M-H	C33H40N6O7	3.6124833333333	-	neg	631.29473867556	0.07884425508622	46.8	-
neg_3244	Tyr-D-Thr-Gly-Phe-Leu-Thr	metab_34471	B(ii)	0	76.1	HMDB0251632	M+Na-2H	C34H48N6O10	3.6124833333333	-	neg	721.31812456088	0.030327000475728	54.1	-
neg_3257	N1,N10-Dicoumaroylspermidine	metab_34484	B(ii)	0	48.9	HMDB0033469	M+FA-H	C25H31N3O4	3.6177666666667	-	neg	482.22565599769	0.027687762321588	46.2	65715-79-9
neg_3273	Ser-Thr-Leu	metab_34500	B(i)	85.1	0	-	M-H	C13H25N3O6	3.6231166666667	-	neg	318.16725822135	0.047175994171614	55	-
neg_3281	Lippioside I	metab_34508	B(ii)	0	72.5	HMDB0034265	M+Cl	C25H30O13	3.6231166666667	-	neg	573.13657127451	0.031682851436521	51.8	220271-93-2
neg_3285	Taurodeoxycholic Acid 3-Glucuronide	metab_34512	B(ii)	0	64	HMDB0240734	M+Cl	C32H53NO12S	3.6231166666667	-	neg	710.30030449466	0.030230951314399	49.9	-
neg_3299	Trp-Glu	metab_34526	B(i)	70.8	0	HMDB0029082	M-H	C16H19N3O5	3.6338166666667	-	neg	332.12599217171	0.014454001835558	52.2	-
neg_3302	Versetamide	metab_34529	B(ii)	0	59.5	HMDB0259796	M-H2O-H	C20H37N5O10	3.6338166666667	-	neg	488.23649255411	0.016421892548825	51	-
neg_3325	2-Acetylpyrrolidine	metab_34552	B(ii)	0	41.8	HMDB0037293	M+FA-H	C6H11NO	3.6445	-	neg	158.08238169864	0.055532644579093	47.2	60026-20-2
neg_3338	Pgp(5-Iso Pgf2Vi/I-14:0)	metab_34565	B(ii)	0	77.7	HMDB0275038	M+Hac-H	C38H70O16P2	3.6445	-	neg	903.42567573661	0.091171742775127	47.6	-
neg_3356	Thr-Asn-Tyr	metab_34583	B(i)	36.2	0	-	M-H	C17H24N4O7	3.6551833333333	-	neg	395.15493937287	0.029101478809203	43.5	-
neg_3365	Val-Gly-Ile	metab_34592	B(i)	70.9	0	-	M-H	C13H25N3O4	3.6604833333333	-	neg	286.17738931442	0.020436058337107	53.1	-

neg_3369	Asp-Tyr-Gln	metab_34596	B(i)	41.2	0	-	M-H2O-H	C18H24N4O8	3.6604833333333	-	neg	405.14198268233	0.027943044850199	46.3	-
neg_3375	Deltaline	metab_34602	B(ii)	0	59.4	HMDB0250984	M+Hac-H	C27H41NO8	3.6604833333333	C08679;_	neg	566.29468098406	0.016505967640566	49.4	6836/11/9;_
neg_3402	Cortisol 21-Mesylate	metab_34629	B(ii)	0	61.2	HMDB0250467	M+FA-H	C22H32O7S	3.6711833333333	-	neg	485.18908506574	0.035586783418135	47.5	-
neg_3425	3-(Methylthio)Propanal	metab_34652	B(ii)	0	41.2	HMDB0031857	M+FA-H	C4H8OS	3.6820333333333	-	neg	149.02794916971	0.060627786273464	46.8	3268-49-3
neg_3429	Phe-Phe-Lys	metab_34656	B(i)	62.5	0	-	M+FA-H	C24H32N4O4	3.6820333333333	-	neg	485.23741870572	0.045030340879227	48.2	-
neg_3444	Clobetasone	metab_34671	B(ii)	0	58.6	HMDB0250332	M+Na-2H	C22H26ClFO4	3.6874	-	neg	429.12554619358	0.025248924311801	44.7	-
neg_3455	Leu-Met	metab_34682	B(i)	85.7	0	HMDB0028935	M-H	C11H22N2O3S	3.69275	-	neg	261.12797341055	0.005599844295023	55.4	-
neg_3469	Gln-Ala-Ile	metab_34696	B(i)	53.8	0	-	M-H	C14H26N4O5	3.6981	-	neg	329.18323093278	0.003084444629844	49	-
neg_3486	Ala-Ser-Leu	metab_34713	B(i)	92	0	-	M-H	C12H23N3O5	3.7034666666667	-	neg	288.15663153881	0.010169368663361	57.4	-
neg_3487	Ile-Glu-Asp	metab_34714	B(i)	36.5	0	-	M-H2O-H	C15H25N3O8	3.7034666666667	-	neg	356.14455539477	0.055349759374167	44.3	-
neg_3488	10-Hydroperoxy-H4-Neuroprostane	metab_34715	B(ii)	0	48.7	HMDB0062274	M-H	C22H32O6	3.7034666666667	-	neg	391.20890060077	0.015767200931945	45.7	-
neg_3490	Melatonin Glucuronide	metab_34717	B(ii)	0	53.8	HMDB0060830	M+FA-H	C19H24N2O8	3.7034666666667	-	neg	453.15162718096	0.043705188563734	48.9	-
neg_3495	Blood Group B Type 1 Tetrasaccharide	metab_34722	B(ii)	0	56.8	HMDB0249293	M+FA-H	C26H45NO20	3.7034666666667	-	neg	736.25651877926	0.2001229521234	49.2	-
neg_3500	Leukotriene-D4	metab_34727	B(ii)	0	51.5	HMDB0304408	M+Na-2H	C25H39N2O6S	3.7088166666667	-	neg	516.23156267455	0.05411864001792	45.4	-
neg_3503	Oxycodone	metab_34730	B(ii)	0	64.2	HMDB0014640	2M-H	C18H21NO4	3.7088166666667	C08018	neg	629.29037795317	0.069616746846843	51.2	76-42-6
neg_3506	Pa(6 Keto-Pgf1Alpha/22:6(4Z,7Z,10Z,13Z,16Z,19Z))	metab_34733	B(ii)	0	41.1	HMDB0266202	M-H2O-H	C45H71O12P	3.7088166666667	-	neg	815.45353857428	0.19804132609346	44.7	-
neg_3509	Leu-Met-Asn	metab_34736	B(i)	47.5	0	-	M-H	C15H28N4O5S	3.7141166666667	-	neg	375.17092925915	0.006646461464546	47.8	-
neg_3510	Lys-Pro-Thr	metab_34737	B(i)	53.3	0	-	M+Hac-H	C15H28N4O5	3.7141166666667	-	neg	403.21996818343	0.023443179563737	50.1	-
neg_3511	Ketoace	metab_34738	B(ii)	0	70.8	HMDB0253787	M+Na-2H	C24H26N2O5	3.7141166666667	-	neg	443.15828253323	0.023321917974508	51.6	-
neg_3519	Ser-Glu-Ile	metab_34746	B(i)	77.9	0	-	M-H	C14H25N3O7	3.71945	-	neg	346.16215011148	0.046420146665744	53.9	-
neg_3530	Ser-Phe-Thr	metab_34757	B(i)	45.5	0	-	M-H	C16H23N3O6	3.72475	-	neg	352.14908728669	0.053741389658749	45.5	-
neg_3531	Val-Gln-Tyr	metab_34758	B(i)	73.5	0	-	M-H	C19H28N4O6	3.72475	-	neg	407.1933623	0.019784754591113	53.3	-
neg_3548	Gamma-Aminobutyric Acid-Betaxanthin	metab_34775	B(ii)	0	55.4	HMDB0304751	M-H	C13H16N2O6	3.7301166666667	-	neg	295.09139729634	0.04766955835956	47.4	-
neg_3570	L-Tryptophan	metab_34797	B(i)	98.6	0	HMDB0000929;PW_C000741	M-H	C11H12N2O2	3.7354166666667	C00078	neg	203.08269829484	0.020565840397856	59.2	73-22-3
neg_3578	3B,6A-Dihydroxy-Alpha-Ionol 9-[Apiosyl-(1->6)-Glucoside]	metab_34805	B(ii)	0	49.6	HMDB0041577	M-H2O-H	C24H40O12	3.7354166666667	-	neg	501.23154404093	0.016427576759798	47.2	177261-72-2
neg_3589	Thr-Phe	metab_34816	B(i)	88.1	0	HMDB0029068	M-H	C13H18N2O4	3.7407333333333	-	neg	265.11951475068	0.013611911546023	56.8	16875-27-7
neg_3593	Melatonin Radical	metab_34820	B(ii)	0	57.6	HMDB0060070	2M-H	C13H18N2O3	3.7407333333333	-	neg	499.2525495	0.063480641567047	43.7	-
neg_3631	Disperse Red 17	metab_34858	B(i)	75.7	0	-	M-H	C17H20N4O4	3.7621333333333	-	neg	343.1411691	0.0478365304836	53.6	-
neg_3642	Glycochenodeoxycholate-3-Sulfate	metab_34869	B(ii)	0	76.2	HMDB0002497	M+FA-H	C26H43NO8S	3.7621333333333	-	neg	574.27336242871	0.047717938243776	51.1	67030-54-0
neg_3645	Avermectin	metab_34872	B(ii)	0	42	HMDB0248739	M+Na-2H	C48H72O14	3.7621333333333	-	neg	893.460377	0.005074753305673	43.9	-
neg_3663	Gentamicin C	metab_34890	B(ii)	0	46.2	HMDB0252688	M+Na-2H	C19H39N5O7	3.7781166666667	C01918;_	neg	470.26212917768	0.053441293771985	43.6	11097-82-8;_
neg_3668	Z-Ile-Glu(O-T-Butyl)-Ala-Leucinal	metab_34895	B(ii)	0	60.6	HMDB0244712	M+Na-2H	C32H50N4O8	3.7781166666667	-	neg	639.3359683	0.062487351844738	48.9	-
neg_3669	(3A,5B)-24-Oxo-24-[(2-Sulfoethyl)Amino]Cholan-3-Yl-B-D-Glucopyranosiduronic Acid	metab_34896	B(ii)	0	68.1	HMDB0002429	M+FA-H	C32H53NO11S	3.7781166666667	-	neg	704.32673465307	0.034076449263938	50.1	99794-82-8
neg_3672	L-Erythro-Phenylserine	metab_34899	B(i)	45.4	0	-	M-H2O-H	C9H11NO3	3.7834666666667	-	neg	162.05606373146	0.004096117854100	48.4	-
neg_3674	Cytosine-5-Carboxylic Acid	metab_34901	B(ii)	0	44.3	HMDB0246202	M+FA-H	C5H5N3O3	3.7834666666667	-	neg	200.0298436	0.009633442495598	45.8	-
neg_3680	3-Amino-3-(4-Chlorophenyl)Propanoic Acid	metab_34907	B(i)	67.3	0	-	2M-H	C9H10ClNO2	3.7834666666667	-	neg	397.07289936809	0.076461074656417	47.7	-
neg_3684	Saicar	metab_34911	B(ii)	0	45.1	HMDB0000797	M+Cl	C13H19N4O12	3.7834666666667	C04823	neg	489.03927177089	0.018821882916196	45.4	3031-95-6
neg_3692	Chalepin Acetate	metab_34919	B(ii)	0	68.1	HMDB0030666	2M+FA-H	C21H24O5	3.7834666666667	C09308	neg	757.31685973011	0.048358270305125	49.4	14882-94-1
neg_3699	Ile-Gln-Pro	metab_34926	B(i)	51.4	0	-	M-H	C16H28N4O5	3.7888166666667	-	neg	355.19884843493	0.04100141230596	48.2	-
neg_3718	N-Docosahexaenoyl Glutamic Acid	metab_34945	B(ii)	0	50.4	HMDB0242013	M+Cl	C27H39NO5	3.7941833333333	-	neg	492.25660610611	0.04083844161667	45.1	_;
neg_3719	(S)-Nerolidol 3-O-[A-L-Rhamnopyranosyl-(1->2)-B-D-Glucopyranoside]	metab_34946	B(ii)	0	55.8	HMDB0040844	M-H	C27H46O10	3.7941833333333	-	neg	529.29931462864	0.060959166109492	47.5	130466-30-7
neg_3727	Thr-Ala-Leu	metab_34954	B(i)	41.4	0	-	M-H	C13H25N3O5	3.7995166666667	-	neg	302.17224567339	0.009053928751086	47.1	-
neg_3732	Amoxicilloyl	metab_34959	B(ii)	0	63.6	HMDB0248351	M+Hac-H	C17H23N3O5S	3.7995166666667	-	neg	440.14608960317	0.011516152167016	45.3	-
neg_3740	1-[6-(2-Carboxypyrrolidin-1-Yl)-6-Oxohexanoyl]Pyrrolidine-2-Carboxylic Acid	metab_34967	B(ii)	0	60.1	HMDB0257669	M+Na-2H	C16H24N2O6	3.80485	-	neg	361.13824486163	0.024422024380385	51.2	-

neg_3748	Paradisin C	metab_34975	B(ii)	0	56.5	HMDB0040155	M+Hac-H	C42H46O11	3.80485	-	neg	785.31136462533	0.003297256772667	47.9	-
neg_3756	Virginiamycin M1	metab_34983	B(ii)	0	88.4	HMDB0259836	M+Na-2H	C28H35N3O7	3.8102	C11299;_	neg	546.22095865081	0.017054533834877	53.2	21411-53-0;_
neg_3762	Leu-Ile-Ser	metab_34989	B(i)	74.3	0	-	M-H2O-H	C15H29N3O5	3.8155333333333	-	neg	312.19293869291	0.057299537062416	52.7	-
neg_3778	Salidroside	metab_35005	B(i)	74.3	0	-;HMDB0257463	M-H	C14H20O7	3.8208833333333	C06046;_	neg	299.11376800762	0.019086344297398	54.1	10338-51-9;_
neg_3779	(1Xi,2Xi)-1-(4-Hydroxyphenyl)-1,2,3-Propanetriol 2-O-Beta-D-Glucopyranoside	metab_35006	B(ii)	0	41	HMDB0040601	M-H	C15H22O9	3.8208833333333	-	neg	345.11917277103	0.022919523077701	48	84744-60-5
neg_3785	Withaperuvin C	metab_35012	B(ii)	0	64.9	HMDB0034077	M+Na-2H	C28H38O7	3.8208833333333	-	neg	507.23236254401	0.016483422987545	48.4	81644-34-0;
neg_3794	Phe-Glu	metab_35021	B(i)	88.3	0	HMDB0028994	M-H	C14H18N2O5	3.8262333333333	-	neg	293.11439879495	0.022454499248412	56.9	3617-45-6
neg_3801	Tangeraxanthin	metab_35028	B(ii)	0	56.2	HMDB0039015	M+Cl	C34H44O2	3.8262333333333	-	neg	519.30337507627	0.12009186409552	47.1	-
neg_3806	2-Cis-Abscisate	metab_35033	B(ii)	0	52.9	HMDB0304067	M+FA-H	C15H19O4-	3.8316	-	neg	308.125376	0.015256619424543	48.7	-
neg_3832	Pe(5-Iso Pgf2Vi/Dime(9,3))	metab_35059	B(ii)	0	53.7	HMDB0285007	M+Hac-H	C41H70NO12P	3.8369333333333	-	neg	858.48394207785	0.08304871010526	46.8	-
neg_3848	Validamycin A	metab_35075	B(ii)	0	79	HMDB0036592	M-H	C20H35NO13	3.8479166666667	C12112	neg	496.20528970902	0.012872419678628	54.1	37248-47-8
neg_3859	Amperozide	metab_35086	B(ii)	0	88.7	HMDB0248358	M+Cl	C23H29F2N3O	3.8532666666667	-	neg	436.19737024377	0.059269730025871	50.7	-
neg_3860	6|A-Hydroxy-7|A-(Thiomethyl)Spirolactone	metab_35087	B(ii)	0	68.9	HMDB0246665	M+Hac-H	C23H32O4S	3.8532666666667	-	neg	463.21603720966	0.049193860322858	50.9	-
neg_3870	N-Acetyl-L-Methionine	metab_35097	B(i)	98.5	0	HMDB0011745	M-H	C7H13NO3S	3.8586	C02712	neg	190.05446504304	0.015188777846595	58.5	65-82-7
neg_3879	Penitrem A	metab_35106	B(ii)	0	77.1	HMDB0242736	M+Na-2H	C37H44ClNO6	3.8586	C20070;_	neg	654.25650997331	0.014462160738237	46.6	12627-35-9;_
neg_3882	3-Hydroxycinnamic Acid	metab_35109	B(i)	82.5	0	HMDB0001713	M-H	C9H8O3	3.86395	C12621	neg	163.04018317404	0.02338796299683	54.7	14755-02-3
neg_3894	Sampatrilat	metab_35121	B(ii)	0	67.1	HMDB0257476	M+Na-2H	C26H40N4O9S	3.86395	-	neg	605.22050460172	0.03680329806646	48.9	-
neg_3900	Val-Arg-Leu	metab_35127	B(i)	90.7	0	-	M-H	C17H34N6O4	3.86925	-	neg	385.25713887751	0.013375617205634	56.2	-
neg_3904	Tetrahydroaldosterone-3-Glucuronide	metab_35131	B(ii)	0	84.7	HMDB0010357	M+Hac-H	C27H40O11	3.86925	-	neg	599.26865184382	0.019879345379294	54.2	20605-81-6;
neg_3911	5-(Delta-Carboxybutyl)Homocysteine	metab_35138	B(ii)	0	44.9	HMDB0246764	2M+FA-H	C8H15NO4S	3.8745333333333	-	neg	487.14589369646	0.10041134934677	44.8	-
neg_3913	2Beta,9Xi-Dihydroxy-8-Oxo-1(10),4,11(13)-Germacratrien-12,6Alpha-Olide	metab_35140	B(ii)	0	54	HMDB0036662	2M+FA-H	C15H18O5	3.8745333333333	-	neg	601.22585819825	0.042044869466342	41.3	-
neg_3916	Ptelatoside B	metab_35143	B(ii)	0	51.6	HMDB0034447	2M-H	C20H28O10	3.8745333333333	-	neg	855.32623489982	0.068688643423348	46.8	90852-99-6
neg_3922	Fluphenazine Enanthate	metab_35149	B(ii)	0	50.2	HMDB0252401	M+Cl	C29H38F3N3O2S	3.8798333333333	C07955	neg	584.23246476191	0.061319186304902	47.9	2746-81-8;_
neg_3925	Hypromellosum	metab_35152	B(ii)	0	71.1	HMDB0256007	M-H2O-H	C32H60O19	3.8798333333333	-	neg	729.35434170209	0.035769053382391	51.9	-
neg_3926	Kinetensin 4-8	metab_35153	B(ii)	0	47.4	HMDB0012987	M+FA-H	C35H46N10O7	3.8798333333333	-	neg	763.35359335611	0.13274681285276	45.5	138482-58-3
neg_3927	Hovenidulcioside A1	metab_35154	B(ii)	0	75.1	HMDB0041028	M-H2O-H	C44H68O16	3.8798333333333	-	neg	833.42788756731	0.007449631729091	51.2	171499-79-9
neg_3929	5-(Hydroxymethyl)-6-(1-Hydroxypropyl)-4-Methoxypyran-2-One	metab_35156	B(i)	64.8	0	-	M-H	C10H14O5	3.8852	-	neg	213.07700212009	0.06490276239536	51.5	-
neg_3936	Evodenoson	metab_35163	B(ii)	0	71.9	HMDB0252137	M+Cl	C23H29N7O6	3.8852	-	neg	534.18446089699	0.061286309113033	50.2	-
neg_3949	Osmanthuside H	metab_35176	B(i)	40.3	0	-	M-H	C19H28O11	3.8958666666667	-	neg	431.15628203048	0.045834174353812	47.2	-
neg_3955	Lycoperoside D	metab_35182	B(ii)	0	75.5	HMDB0034484	M+Hac-H	C39H65NO12	3.8958666666667	-	neg	798.46870611296	0.049809487410544	51.2	81037-16-3
neg_3972	N-Acetylvaline	metab_35199	B(i)	97.4	0	HMDB0011757	M-H	C7H13NO3	3.9119166666667	-	neg	158.08237516789	0.046082205949296	58.4	96-81-1
neg_3974	Secologanin	metab_35201	B(i)	63	0	HMDB0304482;LMPR0102070002	M-H	C17H24O10	3.9119166666667	C01852	neg	387.12910346145	0.028112984924016	50.8	19351-63-4;_;
neg_3976	Phe-Tyr-Tyr	metab_35203	B(i)	44.2	0	-	M-H	C27H29N3O6	3.9119166666667	-	neg	490.19417372646	0.029422844476453	43.8	-
neg_3983	Dg(6 Keto-Pgf1Alpha/0:0/2:0)	metab_35210	B(ii)	0	71.7	HMDB0296914	M+FA-H	C25H42O9	3.9172833333333	-	neg	531.27874446751	0.013769038668362	51.9	-
neg_3994	Thr-Thr-Tyr	metab_35221	B(i)	43.5	0	-	M-H	C17H25N3O7	3.9226333333333	-	neg	382.15969906706	0.027184413676425	45.3	-
neg_3998	Sucrose Laurate	metab_35225	B(ii)	0	53.3	HMDB0258550	M+Na-2H	C24H44O12	3.9226333333333	-	neg	545.25810512059	0.049020861888465	36.4	-
neg_3999	17-Aminogeldanamycin	metab_35226	B(ii)	0	81.6	HMDB0259661	M+Na-2H	C28H39N3O8	3.9226333333333	-	neg	566.24721607415	0.013630403805017	54.8	-
neg_4007	Thr-Glu-Ile	metab_35234	B(i)	89.9	0	-	M-H	C15H27N3O7	3.9279333333333	-	neg	360.17787827891	0.006861081243262	56.6	-
neg_4021	4-Methyl-Umbelliferyl-N-Acetyl-Chitobiose	metab_35248	B(ii)	0	65.2	HMDB0246931	M+Hac-H	C26H34N2O13	3.9332333333333	-	neg	641.21926125085	0.03711532102295	51.5	-
neg_4023	Virginiamycin	metab_35250	B(ii)	0	67.2	HMDB0030520	M+Cl	C43H49N7O10	3.9332333333333	C11269	neg	858.32596055915	0.022611807010237	50.4	11006-76-1
neg_4025	2-Acetamido-3-(4-Acetamidophenyl)Prop-2-Enoic Acid	metab_35252	B(i)	42.5	0	-	M-H	C13H14N2O4	3.9385666666667	-	neg	261.08802113556	0.037442086073595	47.1	-
neg_4046	Leu Ile Gln Asp	metab_35273	B(i)	53.8	0	-	M-H	C21H37N5O8	3.9492666666667	-	neg	486.2572	0.035156275116977	48.8	-
neg_4050	Irinotecan	metab_35277	B(ii)	0	48.5	HMDB0014900	M+Cl	C33H38N4O6	3.9492666666667	C16641	neg	621.25216916729	0.046202015044466	45.1	97682-44-5;100286-90-6

neg_4054	Hispidin	metab_35281	B(ii)	0	41.6	HMDB0253181	M+Hac-H	C13H10O5	3.9546	-	neg	305.06688457701	0.022763030276138	47.4	-
neg_4055	Meta-O-Dealkylated Flecainide	metab_35282	B(ii)	0	60.8	HMDB0060831	M-H	C15H19F3N2O	3.9546	-	neg	331.12777702569	0.023926279342593	50.9	-
neg_4061	Malvidin 3-Alpha-L-Galactoside	metab_35288	B(ii)	0	62	HMDB0303038	M-H2O-H	C25H27O10+	3.9546	-	neg	468.13913212859	0.052623173316943	48.4	-
neg_4065	Thymopentin	metab_35292	B(ii)	0	85.6	HMDB0259057	M+Na-2H	C30H49N9O9	3.9546	-	neg	700.34275582321	0.075773132641851	55.6	-
neg_4068	Richter	metab_35295	B(ii)	0	41	HMDB0257218	M+FA-H	C13H18N2O	3.95995	-	neg	263.14026307069	0.026838433167192	47.6	-
neg_4071	Elafibranor	metab_35298	B(ii)	0	45.1	HMDB0251730	M+Hac-H	C22H24O4S	3.95995	-	neg	443.15510433857	0.10389301588873	42.8	-
neg_4082	Adb-Fubinaca	metab_35309	B(i)	51.4	0	-	M+FA-H	C21H23FN4O2	3.96525	-	neg	427.18134757106	0.03423387671048	46.5	-
neg_4135	Ala Thr Val Glu	metab_35362	B(i)	51.7	0	-	M-H	C17H30N4O8	3.9866	-	neg	417.19935771676	0.026117174484997	49.4	-
neg_4136	Ile Ser Thr Glu	metab_35363	B(i)	67.1	0	-	M-H	C18H32N4O9	3.9866	-	neg	447.20985130818	0.007381203526323	52.8	-
neg_4140	S-(9-Hydroxy-Pga2)-Glutathione	metab_35367	B(ii)	0	73.6	HMDB0013060	M-H	C30H49N3O10	3.9866	-	neg	642.31038605013	0.026825822028752	50.1	-
neg_4144	Gibberellin A29	metab_35371	B(ii)	0	56.1	HMDB0303458	2M+FA-H	C19H24O6	3.9866	C06096;_	neg	741.30584679066	0.033374092260644	46.6	-;_;
neg_4147	Pteroside B	metab_35374	B(ii)	0	73.6	HMDB0030760	M-H	C20H28O7	3.9919333333333	-	neg	379.17256401729	0.038296948863913	51.1	29774-74-1
neg_4148	8-(2-[4-(2-Methoxyphenyl)-1-Piperazinyl]Ethyl)-8-Azaspiro[4.5]Decane-7,9-Dione	metab_35375	B(ii)	0	54.9	HMDB0249327	M+FA-H	C22H31N3O3	3.9919333333333	-	neg	430.23090640885	0.013219494326303	47.6	-
neg_4154	Kelampayoside A	metab_35381	B(ii)	0	58.1	HMDB0038714	M+Hac-H	C20H30O13	3.9919333333333	-	neg	537.17945805515	0.0301744063612	49.3	87562-76-3
neg_4161	Valylhydroxyproline	metab_35388	B(ii)	0	46.9	HMDB0029128	2M-H	C10H18N2O4	3.9972833333333	-	neg	459.24606323446	0.054884909567389	42.2	90965-79-0
neg_4163	5-Alpha-Dihydrotestosterone Glucuronide	metab_35390	B(ii)	0	75.5	HMDB0006203	M+Hac-H	C25H38O8	3.9972833333333	-	neg	525.27195302046	0.025695537593307	53	42037-24-1;
neg_4190	Dihydrozeatin-9-N-Glucoside-O-Glucoside	metab_35417	B(ii)	0	51.7	HMDB0012212	M-H	C22H35N5O11	4.0081333333333	-	neg	544.22635444771	0.020500475386553	50	619326-04-4
neg_4193	Elsamitrucin	metab_35420	B(ii)	0	45	HMDB0251754	M-H2O-H	C33H35NO13	4.0081333333333	-	neg	634.19556972681	0.036063866031302	44.3	-
neg_4196	Ketoleucine	metab_35423	B(i)	50.1	0	HMDB0000695;PW_C000547	M-H	C6H10O3	4.0134333333333	C00233	neg	129.05583757444	0.043036054279836	49.4	816-66-0
neg_4210	Sarmentosin	metab_35437	B(ii)	0	62.7	HMDB0030697	M+Na-2H	C11H17NO7	4.0187833333333	C08340	neg	296.07770917507	0.01029592493845	50	71933-54-5
neg_4212	Phe-Pro-Thr	metab_35439	B(i)	85.9	0	-	M-H	C18H25N3O5	4.0187833333333	-	neg	362.17223355809	0.005716295194133	55.9	-
neg_4223	Aminodeoxykanamycin	metab_35450	B(ii)	0	78.9	HMDB0242522	M+Na-2H	C18H37N5O10	4.0240833333333	-	neg	504.23131459811	0.025879744236129	53.7	-
neg_4253	3-Methoxy-9,10-Dihydrophenanthrene-2,7-Diol	metab_35480	B(i)	46.1	0	-	M-H	C15H14O3	4.0454666666667	-	neg	241.08478600704	0.027530574894566	44.9	-
neg_4258	Gibberellin A24	metab_35485	B(ii)	0	47	HMDB0303459	M+Na-2H	C20H26O5	4.0454666666667	C11861;_	neg	367.15491840945	0.065312165463508	45.3	19427-32-8;_;
neg_4259	N-(1-Carboxy-3-Carboxanilidopropyl)Alanylproline	metab_35486	B(ii)	0	50.4	HMDB0257086	M-H	C19H25N3O6	4.0454666666667	-	neg	390.16723274615	0.030214569139642	48.7	-
neg_4261	Triparanol	metab_35488	B(ii)	0	41.5	HMDB0259258	M-H	C27H32ClNO2	4.0454666666667	-	neg	436.20518586534	0.021216569852342	39	-
neg_4286	Chymostatin	metab_35513	B(ii)	0	83	HMDB0242696	M-H	C31H41N7O6	4.0561166666667	_;C11308	neg	606.29940315906	0.027257772860515	51.9	9076-44-2;_
neg_4291	Tyr-Gly-Ile	metab_35518	B(i)	68.5	0	-	M-H	C17H25N3O5	4.0614666666667	-	neg	350.17225850844	0.008825049447182	52.5	-
neg_4298	Tamoxifen-N-Glucuronide	metab_35525	B(ii)	0	66.2	HMDB0060616	M-H	C32H38NO7+	4.0614666666667	-	neg	547.25562616149	0.032093865298317	50.2	-
neg_4304	Nocloprost	metab_35531	B(ii)	0	69.7	HMDB0255687	M+Na-2H	C22H37ClO4	4.0668	-	neg	421.20912387038	0.010474939671958	46	-
neg_4317	Echujin	metab_35544	B(i)	45.1	0	-	M-H	C42H66O17	4.0668	-	neg	841.41943022641	0.014241566159125	45.6	-
neg_4318	Protocatechuic Aldehyde	metab_35545	B(i)	54.5	0	HMDB0059965	M-H	C7H6O3	4.0721166666667	C16700	neg	137.02452915197	0.052865966789708	49.3	139-85-5
neg_4319	Glu-Phe-Thr	metab_35546	B(i)	83.3	0	-	M-H	C18H25N3O7	4.0721166666667	-	neg	394.16221739199	0.057102189774759	54.6	-
neg_4329	Methyl Reserpate	metab_35556	B(i)	55.6	0	-	M-H	C23H30N2O5	4.0774333333333	-	neg	413.20438323365	0.029207176073182	46.4	-
neg_4339	Subaphylline	metab_35566	B(ii)	0	53.6	HMDB0033463	2M-H	C14H20N2O3	4.0827333333333	C10497	neg	527.28381013003	0.012533065717927	47.7	501-13-3
neg_4344	Kynurenic Acid	metab_35571	B(i)	96	0	HMDB0000715;PW_C000566	M-H	C10H7NO3	4.0880166666667	C01717	neg	188.03543061755	0.017762825406592	58.7	492-27-3
neg_4357	6-Beta-Hydroxymedroxyprogesterone	metab_35584	B(ii)	0	68.1	HMDB0061063	M+FA-H	C22H32O4	4.0933166666667	-	neg	405.22478088258	0.027338637891847	49.2	-
neg_4363	Androstanediol-17G	metab_35590	B(ii)	0	64.2	HMDB0248414	M+FA-H	C25H40O8	4.0933166666667	-	neg	513.26777114865	0.009692836327138	50.1	-
neg_4364	Nintedanib	metab_35591	B(ii)	0	61.4	HMDB0255615	M-H	C31H33N5O4	4.0933166666667	-	neg	538.24198071022	0.024744172193429	49.3	-
neg_4373	Ile-Thr-Pro	metab_35600	B(i)	85.5	0	-	M-H	C15H27N3O5	4.0986333333333	-	neg	328.187956	0.007611156841110	56	-
neg_4375	4R-Hydroxy Solifenacin	metab_35602	B(ii)	0	45.9	HMDB0061127	M+Cl	C23H26N2O3	4.0986333333333	-	neg	413.16585455792	0.030103186563738	45.1	-
neg_4378	Arg-Gly-Asp-Ser	metab_35605	B(ii)	0	60.5	HMDB0248573	M+Hac-H	C15H27N7O8	4.0986333333333	-	neg	492.20803494935	0.007263519164852	49.8	-
neg_4385	Leurocristine	metab_35612	B(ii)	0	70.2	HMDB0259473	M+FA-H	C46H56N4O10	4.0986333333333	C07204;_	neg	869.40114359268	0.015381862011122	48.3	57-22-7;_
neg_4389	Ala-Val-Pro	metab_35616	B(i)	75	0	-	M-H	C13H23N3O4	4.1039166666667	-	neg	284.16173472406	0.030986287117423	53.3	-
neg_4402	4-Formylaminoantipyrine	metab_35629	B(ii)	0	57.8	HMDB0246429	M+Hac-H	C12H13N3O2	4.1145333333333	-	neg	290.11478972434	0.023703416499449	51	-

neg_4407	Caudatin	metab_35634	B(ii)	0	78.1	HMDB0249707	M+Hac-H	C28H42O7	4.1145333333333	-	neg	549.30442695609	0.017270556313243	52.6	-
neg_4410	Pectenotoxin 7	metab_35637	B(ii)	0	89.6	HMDB0032711	M+Cl	C47H68O16	4.1145333333333	-	neg	923.42486305643	0.049051621749093	53.1	-
neg_4414	Linalool 3,6-Oxide Primeveroside	metab_35641	B(ii)	0	55.5	HMDB0035489	M-H	C21H36O11	4.1198333333333	-	neg	463.21768495115	0.037484570154347	49.2	-
neg_4418	Azukisaponin Iii	metab_35645	B(ii)	0	73.7	HMDB0039352	M+FA-H	C42H66O15	4.1198333333333	_;C08930	neg	855.43678668108	0.026215569797615	52.5	82801-38-5
neg_4421	6-Quinolinecarboxylic Acid	metab_35648	B(i)	35.7	0	-	M+FA-H	C10H7NO2	4.1251166666667	-	neg	218.0459582	0.054409919134415	46.8	-
neg_4423	Glu-Ile-Asp	metab_35650	B(i)	82.9	0	-	M-H2O-H	C15H25N3O8	4.1251166666667	-	neg	356.14637895425	0.017765285047916	56.1	-
neg_4442	Robinin	metab_35669	B(i)	60.5	0	HMDB0302847;HMDB0257285;LMPK12111685	M+FA-H	C33H40O19	4.1304166666667	C10178;_	neg	785.21994131061	0.029298568315018	47.4	301-19-9;_;
neg_4443	Leu-Ile	metab_35670	B(i)	93.7	0	HMDB0028932	M-H	C12H24N2O3	4.1357666666667	-	neg	243.17155389049	0.008029562802826	58.1	-
neg_4452	Glutamine-Glutamate	metab_35679	B(ii)	0	58.8	HMDB0252813	2M+FA-H	C10H17N3O7	4.1357666666667	-	neg	627.21582303249	0.079154003661023	47.9	-
neg_4463	Isoleucyl-Gamma-Glutamate	metab_35690	B(ii)	0	59.8	HMDB0028921	M-H2O-H	C11H21N3O4	4.1411	-	neg	240.13551503853	0.018251655728469	51.1	-
neg_4465	Tryptophyl-Asparagine	metab_35692	B(ii)	0	49.8	HMDB0029078	M-H	C15H18N4O4	4.1411	-	neg	317.12574070628	0.058095173410729	47	-
neg_4469	Mirodenafil	metab_35696	B(ii)	0	64.6	HMDB0254750	M+Na-2H	C26H37N5O5S	4.1411	-	neg	552.2296395	0.037987755229906	50.3	-
neg_4478	3-Hydroxy-5-[3,4,5-Trihydroxy-6-(Hydroxymethyl)Oxan-2-Yl]Oxydecanoic Acid	metab_35705	B(i)	93.3	0	-	M+FA-H	C16H30O9	4.1464666666667	-	neg	411.18717511449	0.042951308713615	56.3	-
neg_4488	Phe Tyr	metab_35715	B(i)	88.1	0	-	M-H	C18H20N2O4	4.1518166666667	-	neg	327.13519510244	0.024410469680705	56.2	-
neg_4490	Mimosine	metab_35717	B(ii)	0	52.4	HMDB0015188	2M-H	C8H10N2O4	4.1518166666667	C04771	neg	395.12271960394	0.008081118168324	49.1	500-44-7
neg_4503	1-O-E-Cinnamoyl-(6-Arabinosylglucose)	metab_35730	B(ii)	0	57.3	HMDB0030294	M+FA-H	C20H26O11	4.15715	-	neg	487.14505876243	0.02367662983495	50	181373-66-0
neg_4507	Annomuricatin B	metab_35734	B(ii)	0	91.3	HMDB0303182	M+Cl	C35H49N9O9	4.15715	-	neg	774.33013151408	0.056314955849914	52.3	-
neg_4508	Pg(Pgj2/A-13:0)	metab_35735	B(ii)	0	66.9	HMDB0270563	M+Cl	C39H67O12P	4.15715	-	neg	793.40394828514	0.033312572034376	51.1	-
neg_4515	Dimethylprotoporphyrin Ix Dimethyl Ester	metab_35742	B(ii)	0	43.7	HMDB0000810	M-H2O-H	C36H38N4O4	4.16245	-	neg	571.27401136108	0.040088467774197	44.6	5522-66-7
neg_4519	Phe-Gly-Gln	metab_35746	B(i)	50.1	0	-	M-H2O-H	C16H22N4O5	4.1677833333333	-	neg	331.14127362979	0.020112041300616	48.9	-
neg_4527	Gly-Trp	metab_35754	B(i)	84.8	0	HMDB0028852	M-H	C13H15N3O3	4.1730833333333	-	neg	260.10425445997	0.029240915056687	55.5	-
neg_4537	Retaspimycin	metab_35764	B(ii)	0	91.2	HMDB0257169	M+Na-2H	C31H45N3O8	4.1730833333333	-	neg	608.29386394322	0.009081469419953	56.6	-
neg_4538	Natamycin	metab_35765	B(ii)	0	85.6	HMDB0014964	M-H	C33H47NO13	4.1730833333333	C08073;_	neg	664.2952048	0.009616891330490	55.5	7681-93-8;_
neg_4544	Deoxypyridinoline	metab_35771	B(ii)	0	80.6	HMDB0000569	M+FA-H	C18H28N4O7	4.1787166666667	-	neg	457.1940429	0.009044875732135	55.9	83462-55-9
neg_4548	Naringin	metab_35775	B(ii)	0	50.8	HMDB0002927	M+Cl	C27H32O14	4.1787166666667	C09789	neg	615.15130624228	0.094351614649956	45.7	10236-47-2;
neg_4555	Neuromedin N (1-4)	metab_35782	B(ii)	0	50.6	HMDB0013021	M-H2O-H	C26H40N4O6	4.18405	-	neg	485.27317713818	0.02813767195682	45.9	-
neg_4562	Ramiprilat	metab_35789	B(ii)	0	55.4	HMDB0060579	2M+FA-H	C21H28N2O5	4.18405	-	neg	821.40560389278	0.024261722075136	46.3	_;87269-97-4
neg_4564	Pyridoxal	metab_35791	B(i)	54.9	0	HMDB0001545;PW_C001188	M-H	C8H9NO3	4.1894	C00250	neg	166.0510915	0.056686302356348	49.4	66-72-8
neg_4568	Lactose 6-Phosphate	metab_35795	B(ii)	0	66.2	HMDB0006789	M-H	C12H23O14P	4.1894	C05396	neg	421.07543190285	0.065300792715667	52.2	-
neg_4569	Leu Ser Glu Lys	metab_35796	B(i)	39.8	0	-	M-H	C20H37N5O8	4.1894	-	neg	474.25713103115	0.022319634952779	46.5	-
neg_4571	Omadacycline	metab_35798	B(ii)	0	50.6	HMDB0255962	M-H	C29H40N4O7	4.1894	-	neg	555.27872955016	0.013994810276393	46.8	-
neg_4583	Luteolin 7-Glucuronide	metab_35810	B(ii)	0	62.4	HMDB0240541	M-H2O-H	C21H18O12	4.1947	C03515;_	neg	443.05743994614	0.04064742545599	48.1	_;
neg_4592	Ser-Ser-Trp	metab_35819	B(i)	76.4	0	-	M-H	C17H22N4O6	4.2000333333333	-	neg	377.14676642339	0.02054144594311	51.8	-
neg_4593	Leu-Thr-Tyr	metab_35820	B(i)	67.2	0	-	M-H	C19H29N3O6	4.2000333333333	-	neg	394.19821026414	0.002460451322883	39.6	-
neg_4595	Deflazacort	metab_35822	B(ii)	0	72.5	HMDB0250935	M+Na-2H	C25H31NO6	4.2000333333333	-	neg	462.18591774086	0.023114846347027	47.5	-
neg_4599	Trp-Ala	metab_35826	B(i)	68	0	HMDB0029076	M-H	C14H17N3O3	4.2053833333333	-	neg	274.11986609501	0.007667278402129	52.4	-
neg_4604	Lys-Gln-Tyr	metab_35831	B(i)	55.5	0	-	M+FA-H	C20H31N5O6	4.2053833333333	-	neg	482.22348233171	0.013636499644394	47.1	-
neg_4608	Penitrem B	metab_35835	B(ii)	0	71.6	HMDB0256239	M+FA-H	C37H45NO5	4.2053833333333	C20731;_	neg	628.33125804387	0.15207776981113	50.1	11076-67-8;_
neg_4612	Met-Ile	metab_35839	B(i)	60.9	0	HMDB0028976	M-H	C11H22N2O3S	4.2106833333333	-	neg	261.1273259	0.00637541473058	50.3	-
neg_4617	Rotigaptide	metab_35844	B(ii)	0	75.6	HMDB0257314	M-H	C28H39N7O9	4.2106833333333	-	neg	616.27738167925	0.035182167835498	51.7	-
neg_4633	Polysorbate 20	metab_35860	B(ii)	0	78.3	HMDB0037182	M+Na-2H	C26H50O10	4.2212833333333	C11624	neg	543.31507522787	0.16830816571558	53.1	9005-64-5
neg_4637	Trp-Thr	metab_35864	B(i)	90.3	0	HMDB0029093	M-H	C15H19N3O4	4.2265833333333	-	neg	304.13087556594	0.014005719445453	55.7	-
neg_4656	Decarbamoylsaxitoxin	metab_35883	B(ii)	0	60.4	HMDB0038319	2M+FA-H	C9H16N6O3	4.23195	_;C20021	neg	557.25817876959	0.061478132788315	49.8	58911-04-9
neg_4665	Blennin D	metab_35892	B(ii)	0	51.4	HMDB0031902	M+Cl	C15H22O4	4.24265	-	neg	301.11949941693	0.15446688716055	47.9	72601-39-9
neg_4671	Indicine	metab_35898	B(ii)	0	77.7	HMDB0253457	2M-H	C15H25NO5	4.24265	C10326;_	neg	597.33692974895	0.023458545419643	52.9	480-82-0;_

neg_4678	Gln-Ile-Tyr	metab_35905	B(i)	58.2	0	-	M-H	C20H30N4O6	4.24795	-	neg	421.20907404865	0.030951707278082	49.6	-
neg_4679	Blasticidin S	metab_35906	B(ii)	0	82.5	HMDB0030452	M+Cl	C17H26N8O5	4.24795	C02010	neg	457.17491604795	0.1331130093355	52	2079-00-7
neg_4680	1-(N(Sup Alpha)-Dansyl-L-Arginyl)-4-Ethylpiperidine	metab_35907	B(ii)	0	50.8	HMDB0249398	M-H	C25H38N6O3S	4.24795	-	neg	501.26791652159	0.010468818385247	45.4	-
neg_4688	Methyl Gallate	metab_35915	B(i)	94.8	0	HMDB0254590	M-H	C8H8O5	4.2532833333333	-	neg	183.03000942859	0.008403622526771	58.2	-
neg_4689	(2Z)-3-[2-(Beta-D-Glucopyranosyloxy)Phenyl]Acrylic Acid	metab_35916	B(i)	62.2	0	HMDB0060077	M-H	C15H18O8	4.2532833333333	C05839	neg	325.09304401485	0.024638889618059	51.3	2446-60-8
neg_4692	Indinavir	metab_35919	B(ii)	0	76.2	HMDB0014369	M+Na-2H	C36H47N5O4	4.2532833333333	C07051	neg	634.33247555529	0.072321746371225	48.7	150378-17-9
neg_4693	Heliangin	metab_35920	B(ii)	0	72	HMDB0036692	2M-H	C20H26O6	4.2532833333333	C09474;_	neg	723.33188333419	0.013035573714868	50.4	13323-48-3
neg_4697	5'-Sulfamoyl-2-Chloroadenosine	metab_35924	B(ii)	0	52.4	HMDB0248477	M+Na-2H	C10H13ClN6O6S	4.2586333333333	-	neg	401.00557276466	0.027263776976274	43.4	-
neg_4708	Myricanol 5-Glucoside	metab_35935	B(ii)	0	60.7	HMDB0036525	M-H	C27H36O10	4.2639833333333	-	neg	519.22144071039	0.01626326231045	47.6	90052-02-1
neg_4717	Xi-2,3-Dihydro-3,5-Dihydroxy-6-Methyl-4H-Pyran-4-One	metab_35944	B(ii)	0	47.4	HMDB0036380	M-H	C7H10O4	4.26935	-	neg	157.05074354902	0.029139218022291	48.1	-
neg_4718	2-Isopropylmalic Acid	metab_35945	B(i)	95.5	0	HMDB0000402	M-H	C7H12O5	4.26935	C02504	neg	175.06124383943	0.010022204148187	58.9	-;49601-06-1
neg_4721	Val-Phe	metab_35948	B(i)	85.5	0	HMDB0029134	M-H	C14H20N2O3	4.26935	-	neg	263.14023321977	0.011138572059092	56.4	3918-92-1
neg_4723	Zanamivir	metab_35950	B(ii)	0	53.9	HMDB0014698	M-H	C12H20N4O7	4.26935	C08095	neg	331.12782102663	0.009200318855234	48.6	139110-80-8
neg_4725	Swertiamarin	metab_35952	B(ii)	0	45.2	HMDB0258644	M-H	C16H22O10	4.26935	C09800;_	neg	373.11179422401	0.05719754718813	46.5	17388-39-5;_
neg_4731	6-O-(7-Methyloctanoyl)-Alpha-D-Glucopyranosyl Alpha-D-Glucopyranoside	metab_35958	B(i)	95.1	0	-	M+FA-H	C21H38O12	4.26935	-	neg	527.23451270454	0.1063359711723	58.3	-
neg_4732	Leukotriene D4	metab_35959	B(ii)	0	69.2	HMDB0003080	M+Hac-H	C25H40N2O6S	4.26935	C05951	neg	555.27925609563	0.016240574976112	49.5	73836-78-9
neg_4733	Deserpidine	metab_35960	B(ii)	0	49.8	HMDB0015221	M-H	C32H38N2O8	4.26935	C06541	neg	577.26094855992	0.034251858590047	44.2	131-01-1
neg_4734	Petasinoside	metab_35961	B(ii)	0	41.9	HMDB0030317	M+Hac-H	C28H37NO9	4.26935	-	neg	590.25869726157	0.036793004623818	45.9	70474-34-9
neg_4745	Tyr-Val-Tyr	metab_35972	B(i)	58.7	0	-	M-H	C23H29N3O6	4.2747	-	neg	442.19718734226	0.031931736522653	46.1	-
neg_4748	Pro-Thr-Phe	metab_35975	B(i)	91.7	0	-	M-H	C18H25N3O5	4.2800333333333	-	neg	362.17241110735	0.010112209384756	57.1	-
neg_4752	Taurocholic Acid 3-Sulfate	metab_35979	B(ii)	0	66.4	HMDB0002581	M+Hac-H	C26H45NO10S	4.2800333333333	-	neg	654.25664919559	0.18309181443353	46.8	67030-62-0;
neg_4756	3-Isopropylmalic Acid	metab_35983	B(ii)	0	42.1	HMDB0012156	M+Na-2H	C7H12O5	4.2853833333333	C04411	neg	197.04314901916	0.03451242043501	47.4	126576-14-5
neg_4763	Tricrocin	metab_35990	B(ii)	0	79.3	HMDB0002376	M-H	C38H54O19	4.2853833333333	-	neg	813.31522363196	0.032669816	51.4	55750-84-0
neg_4773	Pimozide	metab_36000	B(ii)	0	43.6	HMDB0015232	M+Hac-H	C28H29F2N3O	4.2907	C07566	neg	520.24002634817	0.031664113993732	47	2062-78-4
neg_4774	5B-Cyprinol Sulfate	metab_36001	B(ii)	0	56.9	HMDB0006888	M+Cl	C27H48O8S	4.2907	C05468	neg	567.27853266833	0.035542389295951	47.9	-
neg_4787	Dgdg(O-16:0/2:0)	metab_36014	B(i)	40.4	0	-	M-H	C33H62O14	4.29605	-	neg	681.40562078266	0.012204519635782	46.8	-
neg_4789	Convalloside	metab_36016	B(ii)	0	62	HMDB0034195	M+Cl	C35H52O15	4.29605	C19986;_	neg	747.29615396792	0.026286174033105	50.1	13473-51-3
neg_4795	Fumagillin	metab_36022	B(i)	36.3	0	-;HMDB0242599;LMPR0103060003	M+FA-H	C26H34O7	4.30135	C09668;_	neg	503.2266011	0.056172698184756	44	23110-15-8;_;
neg_4797	Ergotamine	metab_36024	B(ii)	0	47.3	HMDB0014834	M-H	C33H35N5O5	4.30135	_;C07544	neg	580.26054559041	0.055255056104968	45.1	113-15-5;_
neg_4810	Glycochenodeoxycholic Acid 3-Glucuronide	metab_36037	B(ii)	0	56.6	HMDB0002579	M+Na-2H	C32H51NO11	4.3066666666667	C03033;_	neg	646.32467414199	0.034470783963616	48.1	79254-98-1;
neg_4811	Tryptophan Glutamate	metab_36038	B(ii)	0	70.9	HMDB0259308	2M-H	C16H19N3O6	4.3066666666667	-	neg	697.24879927118	0.10028858111402	52	-
neg_4814	(+)-8-Acetoxycarvone	metab_36041	B(ii)	0	42.2	HMDB0302748	M+FA-H	C12H16O3	4.3119666666667	-	neg	253.1082111	0.045678265102517	46.8	-
neg_4816	Shanzhiside	metab_36043	B(ii)	0	53.7	HMDB0258270	M-H	C16H24O11	4.3119666666667	C17066;_	neg	391.12255998267	0.037467831705581	47.1	-;_
neg_4817	Ala Tyr Ile Asp	metab_36044	B(i)	49.2	0	-	M-H	C22H32N4O8	4.3119666666667	-	neg	479.21494161387	0.013598029804103	48.8	-
neg_4829	3-(1-(Cyclohexylmethyl)-1H-Indazole-3-Carboxamido)-2,2-Dimethylsuccinicacid	metab_36056	B(i)	82.6	0	-	M+FA-H	C21H27N3O5	4.3172666666667	-	neg	446.19078933757	0.045535981881444	54.3	-
neg_4833	Aypgkf	metab_36060	B(ii)	0	67.1	HMDB0248753	M+Cl	C34H47N7O8	4.3172666666667	-	neg	716.32244864058	0.076296874401753	49.2	-
neg_4835	Lusitanicoside	metab_36062	B(ii)	0	51.3	HMDB0034120	M+Hac-H	C21H30O10	4.3225666666667	C10474	neg	501.19673468615	0.019591392612795	48.2	499-35-4;_
neg_4839	Leucyl-Leucine	metab_36066	B(ii)	0	52.7	HMDB0254039	M-H	C12H24N2O3	4.3278666666667	C11332	neg	243.17155549057	0.028740464538357	49.7	2883-36-5;3303-31-9;_
neg_4850	N-Alpha-Acetyl-L-Citrulline	metab_36077	B(ii)	0	51.9	HMDB0000856	2M-H	C8H15N3O4	4.3331666666667	C15532	neg	433.20940112859	0.008787693853636	46.2	33965-42-3
neg_4858	Thesinine 4'-O-Glucoside	metab_36085	B(ii)	0	71.7	HMDB0039900	M+Na-2H	C23H31NO8	4.3385166666667	-	neg	470.17795902715	0.086052614067396	52.3	-
neg_4890	Naltrexone	metab_36117	B(ii)	0	60.2	HMDB0014842	2M+FA-H	C20H23NO4	4.3547166666667	C07253	neg	727.32749462122	0.035591785835299	47.6	16590-41-3
neg_4903	Val-Asp-Leu	metab_36130	B(i)	55.6	0	-	M-H	C15H27N3O6	4.3654	-	neg	344.18280658521	0.017276072145176	50.4	-
neg_4904	Ile-Ser-Tyr	metab_36131	B(i)	71.8	0	-	M-H	C18H27N3O6	4.3654	-	neg	380.1825882	0.015180455813201	52.6	-

neg_4914	5-Hydroxyprimaquine	metab_36141	B(ii)	0	54.1	HMDB0246810	2M+FA-H	C15H21N3O2	4.3654	-	neg	595.32049321683	0.087023652450682	47.7	-
neg_4917	(D-Ala1)-Peptide T	metab_36144	B(ii)	0	83.7	HMDB0260060	M-H2O-H	C35H55N9O16	4.3654	-	neg	838.35922990953	0.008433167947684	54.4	-
neg_4921	Ser-Met-Ile	metab_36148	B(i)	69	0	-	M-H	C14H27N3O5S	4.3707	-	neg	348.1567386	0.000440752940986	50.8	-
neg_4938	Pe(Pgf2Alpha/18:3(9Z,12Z,15Z))	metab_36165	B(ii)	0	43.5	HMDB0261641	M+Na-2H	C43H74NO11P	4.376	-	neg	832.46887960809	0.036475544655556	45	-
neg_4940	Ser-Val-Ile	metab_36167	B(i)	87.3	0	-	M-H	C14H27N3O5	4.3813	-	neg	316.18791544512	0.011227785443961	56.3	-
neg_4941	Glu-Val-Ile	metab_36168	B(i)	81.1	0	-	M-H	C16H29N3O6	4.3813	-	neg	358.1984104	0.059138873329965	54.2	-
neg_4946	(2S,3R)-2-Acetamido-3-Hydroxy-4-Methylpentanoate	metab_36173	B(ii)	0	89	HMDB0242664	M+FA-H	C46H69N7O15	4.3813	-	neg	1004.4807632467	0.031424054057061	55.7	-
neg_4947	Val-Pro-Leu	metab_36174	B(i)	43	0	-	M-H	C16H29N3O4	4.3866	-	neg	326.20866666545	0.012178190471925	47.6	-
neg_4955	Cinncassiol C3	metab_36182	B(ii)	0	76.7	HMDB0036859	2M+FA-H	C20H30O7	4.3866	C17641;_	neg	809.39542586904	0.011654741810109	51.7	-;64979-94-8
neg_4959	Ethyl 1-(Furan-2-Ylmethyl)-4-Hydroxy-5-Oxo-2H-Pyrrole-3-Carboxylate	metab_36186	B(i)	63.1	0	-	M+FA-H	C12H13NO5	4.39195	-	neg	296.07772577401	0.050301110438292	51.9	-
neg_4960	Val-Ile-Tyr	metab_36187	B(i)	61.3	0	-	M-H	C20H31N3O5	4.39195	-	neg	392.21898230597	0.005596567508504	50	-
neg_4965	Ergokryptine	metab_36192	B(ii)	0	47.1	HMDB0248211	M+Hac-H	C32H41N5O5	4.39195	-	neg	634.32067203983	0.014987855773358	46.6	-
neg_4971	Tyr-Gly-Lys	metab_36198	B(i)	86.8	0	-	M-H	C17H26N4O5	4.3972833333333	-	neg	365.18159274347	0.17545125071548	53.3	-
neg_4991	10-Hydroxynortriptyline	metab_36218	B(ii)	0	41	HMDB0244273	2M+FA-H	C19H21NO	4.4026	-	neg	603.32597543389	0.019968403642744	46.1	-
neg_4997	Pi(6:0/8:0)	metab_36224	B(i)	49.5	0	-	M+FA-H	C23H43O13P	4.4079333333333	-	neg	603.24008773531	0.013489977637188	48.5	-
neg_5002	Ps(6 Keto-Pgf1Alpha/15:0)	metab_36229	B(ii)	0	92.1	HMDB0281035	M+FA-H	C41H74NO14P	4.4079333333333	-	neg	880.47869201317	0.015063290625859	56.3	-
neg_5004	Arg-Asp-Asp	metab_36231	B(i)	51.5	0	-	M-H	C14H24N6O8	4.41325	-	neg	403.16218678931	0.014423664976376	47.6	-
neg_5051	Leu-Leu	metab_36278	B(i)	92.2	0	HMDB0028933	M-H	C12H24N2O3	4.4399166666667	C11332	neg	243.17152016242	0.008031477819638	57.8	3303-31-9
neg_5063	L-Cis-Cyclo(Aspartylphenylalanyl)	metab_36290	B(ii)	0	43.7	HMDB0031360	M-H	C13H14N2O4	4.44525	-	neg	261.08817685098	0.021974035846989	47.9	5262/10/2
neg_5064	Quercetin 3-O-Sophoroside	metab_36291	B(ii)	0	52	HMDB0304729	M+Na-2H	C14H16N2O6	4.44525	C12667;C08549	neg	329.07571522289	0.083658140182446	48.6	18609-17-1;_
neg_5087	Wkymvm	metab_36314	B(ii)	0	56.9	HMDB0259899	M-H	C41H61N9O7S	4.4506	-	neg	854.40090241704	0.04020273643123	46.4	-
neg_5093	3-Benzyl-5-(6-Carboxyhexyl)-1-(2-Cyclohexyl-2-Hydroxyethylamino)Hydantoin	metab_36320	B(ii)	0	54.8	HMDB0249489	M-H	C25H37N3O5	4.45595	-	neg	458.26247384636	0.036497900396395	45.8	-
neg_5100	Methyl 7-Epi-12-Hydroxyjasmonate Glucoside	metab_36327	B(ii)	0	80.8	HMDB0031763	M+Cl	C19H30O9	4.4613	-	neg	437.15682712849	0.015440204908511	55.1	142465-56-3
neg_5108	Asp-Val-Ile	metab_36335	B(i)	92.4	0	-	M-H	C15H27N3O6	4.4666333333333	-	neg	344.18277918728	0.021295810834468	57.6	-
neg_5109	2-[[[4-(3-Benzylcyclobutyl)-1,3-Thiazol-2-Yl]Diazenyl]Methyl]Phenol	metab_36336	B(ii)	0	59.2	HMDB0255368	M-H	C21H21N3OS	4.4666333333333	-	neg	362.13589744999	0.014569611024136	47.7	-
neg_5111	Lenapenem	metab_36338	B(ii)	0	51.4	HMDB0254007	M+Cl	C18H29N3O5S	4.4666333333333	-	neg	434.15229633061	0.029468466774801	47.5	-
neg_5115	Thr-Val-Leu	metab_36342	B(i)	90.7	0	-	M-H	C15H29N3O5	4.4719666666667	-	neg	330.20366317086	0.020750409511284	56.8	-
neg_5126	Fluocinolone	metab_36353	B(ii)	0	53.8	HMDB0252347	M+FA-H	C21H26F2O6	4.47725	C07006;_	neg	457.16500073171	0.13093893263904	47.8	807-38-5;_
neg_5141	Apstatin	metab_36368	B(ii)	0	69.2	HMDB0248539	M+Na-2H	C23H33N5O5	4.4878833333333	-	neg	480.22270732541	0.055274030139244	49.9	-
neg_5150	Pf-3845	metab_36377	B(i)	58.7	0	-	M-H	C24H23F3N4O	4.4932333333333	-	neg	455.16729420332	0.016322809860734	48.1	-
neg_5156	Val-Asn-Ile	metab_36383	B(i)	85.1	0	-	M-H	C15H28N4O5	4.4985833333333	-	neg	343.19879188192	0.023833460298267	55.1	-
neg_5160	Verdamicin	metab_36387	B(ii)	0	61.7	HMDB0259789	M+Na-2H	C20H39N5O7	4.4985833333333	-	neg	482.26225934658	0.036798552808168	49.5	-
neg_5166	Brivanib	metab_36393	B(ii)	0	43.3	HMDB0249386	M+Na-2H	C19H19FN4O3	4.5039333333333	-	neg	391.11830610702	0.099289254059937	45.3	-
neg_5176	5-Benzylacyclouridine	metab_36403	B(ii)	0	41.2	HMDB0246756	M-H	C14H16N2O4	4.5095333333333	-	neg	275.10385695908	0.026664152932724	47	-
neg_5177	Nopalinic Acid	metab_36404	B(ii)	0	51.9	HMDB0029437	M+Cl	C10H18N2O6	4.5095333333333	C01683	neg	297.08586098039	0.020002411492914	49.8	-;63409-16-5
neg_5182	Atractyloside B	metab_36409	B(i)	41.7	0	-	M+FA-H	C21H38O10	4.5095333333333	C17859	neg	495.24379390744	0.038717590922919	46.6	-
neg_5203	8-Hydroxycarvedilol	metab_36430	B(ii)	0	52.9	HMDB0013946	M-H2O-H	C24H26N2O5	4.5202166666667	-	neg	403.16250978297	0.032176855398708	46.4	-
neg_5207	Neuromedin B (1-3)	metab_36434	B(ii)	0	77.6	HMDB0013016	2M-H	C12H22N4O5	4.5202166666667	-	neg	603.31493090938	0.006651957493871	51.7	-
neg_5215	3-Trans-Caffeoyltormentic Acid	metab_36442	B(ii)	0	48.1	HMDB0040650	M+FA-H	C39H54O8	4.5255833333333	-	neg	695.38021696491	0.094136036870713	46.3	144604-16-0
neg_5234	Cortisone Acetate	metab_36461	B(ii)	0	47.8	HMDB0015459	M+Na-2H	C23H30O6	4.5415666666667	C08173	neg	423.17734772796	0.07345064467199	46.6	1950/4/4;50-04-4;
neg_5237	Aristospan	metab_36464	B(ii)	0	66.2	HMDB0248600	M+Na-2H	C30H41FO7	4.5415666666667	-	neg	553.26098711258	0.025047298897508	50.8	-
neg_5253	(2S)-2-Amino-3-[3-[(2S)-2-Amino-3-Phenylpropanoyl]Oxyphenyl]Propanoic Acid	metab_36480	B(ii)	0	43.9	HMDB0257687	M-H	C18H20N2O4	4.55225	-	neg	327.13520083427	0.004658430911412	47.9	-
neg_5255	Leu-Arg-Ile	metab_36482	B(i)	93.2	0	-	M-H	C18H36N6O4	4.55225	-	neg	399.27274074578	0.011601501334295	57.2	-

neg_5261	Pi(5-Iso Pgf2Vi/18:0)	metab_36488	B(ii)	0	62.5	HMDB0276385	M+Cl	C45H81O16P	4.55225	-	neg	943.50232695374	0.059207489323266	49.4	-
neg_5273	2-Ethoxyethyl Acetate	metab_36500	B(ii)	0	41.4	HMDB0245111	M-H	C6H12O3	4.5629166666667	-	neg	131.07147197944	0.012071210899843	47.7	-
neg_5280	Metronidazole Phosphate	metab_36507	B(ii)	0	41.8	HMDB0254689	2M+FA-H	C6H10N3O6P	4.5629166666667	-	neg	547.05630119358	0.09115616380624	45.3	-
neg_5290	Leu-His-Ile	metab_36517	B(i)	52.6	0	-	M+Hac-H	C18H31N5O4	4.5682	-	neg	440.25181596465	0.018879841362516	49.3	-
neg_5310	15-Hydroxynorandrostene-3,17-Dione Glucuronide	metab_36537	B(ii)	0	53.9	HMDB0010353	2M+FA-H	C24H32O9	4.5735166666667	C03033;_	neg	973.41618445383	0.040670055486649	45.1	131749-23-0;
neg_5311	Epicatechin	metab_36538	B(i)	90.9	0	HMDB0001871;LMPK12020003	M-H	C15H14O6	4.5788166666667	C09727	neg	289.07199874711	0.002317906041776	56.9	490-46-0;
neg_5312	Val-Gln-Leu	metab_36539	B(i)	84.7	0	-	M-H	C16H30N4O5	4.5788166666667	-	neg	357.21448386425	0.011322526579491	55.7	-
neg_5314	Epigallocatechin Gallate	metab_36541	B(i)	58	0	HMDB0003153	M-H	C22H18O11	4.5788166666667	C09731	neg	457.07777608829	0.034045536460648	49.7	989-51-5
neg_5315	Coumaroyl(3-Ome)(-3)Glc(A1-2B)L-Fruf	metab_36542	B(i)	66	0	-	M-H	C22H30O14	4.5788166666667	-	neg	517.15652552908	0.013587039290318	51.2	-
neg_5320	Val-Thr-Ile	metab_36547	B(i)	87.4	0	-	M-H	C15H29N3O5	4.5841166666667	-	neg	330.20367597638	0.028692576898293	56.1	-
neg_5321	Ala Gly Leu Val Ser	metab_36548	B(i)	60.8	0	-	M-H	C19H35N5O7	4.5841166666667	-	neg	444.24645655202	0.008273346079406	50.2	-
neg_5324	Spinacoside D	metab_36551	B(ii)	0	64.5	HMDB0034871	M+Na-2H	C40H60O14	4.5841166666667	-	neg	785.36843848779	0.013706497894168	50.1	182322-58-3
neg_5325	Sabeluzole	metab_36552	B(ii)	0	50.7	HMDB0257434	2M-H	C22H26FN3O2	4.5841166666667	-	neg	829.33280353362	0.033360017473749	46.8	-
neg_5331	Echinacoside	metab_36558	B(i)	79.3	0	-;HMDB0251686	M-H	C35H46O20	4.5894166666667	C10450;_	neg	785.25134292144	0.013852160872847	55	82854-37-3;_
neg_5334	Ile-Asp-Ile	metab_36561	B(i)	46.6	0	-	M-H	C16H29N3O6	4.59475	-	neg	358.19871734315	0.06175984335976	45.9	-
neg_5356	Carvedilol	metab_36583	B(ii)	0	53.8	HMDB0015267	2M-H	C24H26N2O4	4.6000833333333	C06875	neg	811.3633145	0.015058851839143	45.2	72956-09-3
neg_5367	Sphingosine 1-Phosphate	metab_36594	B(ii)	0	40.8	HMDB0000277	M+Na-2H	C18H38NO5P	4.6107833333333	C06124	neg	400.22021187091	0.03787275833799	42.8	26993-30-6
neg_5368	2-Phenylethyl D-Rutinoside	metab_36595	B(i)	46.4	0	-	M+FA-H	C20H30O10	4.6107833333333	-	neg	475.18334277326	0.024080977492628	47.3	-
neg_5369	Ketotrexate	metab_36596	B(ii)	0	48.5	HMDB0253793	M+Hac-H	C21H27N7O6	4.6107833333333	-	neg	532.21497078793	0.062604594160144	43.6	-
neg_5381	Glu-Ile	metab_36608	B(i)	60.9	0	HMDB0028822	M-H2O-H	C11H20N2O5	4.6214333333333	-	neg	241.11950191083	0.004783379204334	51.5	5879-22-1
neg_5383	Tyr-Asn-Ile	metab_36610	B(i)	53.5	0	-	M-H	C19H28N4O6	4.6214333333333	-	neg	407.19350359187	0.041921454816689	48.2	-
neg_5387	Cucurbitacin A	metab_36614	B(ii)	0	48.5	HMDB0302593	M+Hac-H	C32H46O9	4.6214333333333	C08793;_	neg	633.32604652446	0.15846605494874	47.2	6040-19-3;_;
neg_5399	Bioadykinin Fragment 1-5	metab_36626	B(ii)	0	40.5	HMDB0245648	M+Cl	C27H40N8O6	4.62675	-	neg	607.27447811716	0.055332792690498	43.8	-
neg_5406	Ile-Leu	metab_36633	B(i)	90.2	0	HMDB0028911	M-H	C12H24N2O3	4.6374333333333	-	neg	243.17148469724	0.015467508888145	57.4	-
neg_5409	(2S)-6-Amino-2-(Hexanoylamino)Hexanoic Acid	metab_36636	B(ii)	0	46.1	HMDB0253148	2M-H	C12H24N2O3	4.6374333333333	-	neg	487.35009810037	0.07905088169218	48	-
neg_5424	Bergamottin	metab_36651	B(ii)	0	41.3	HMDB0033782	M-H2O-H	C21H22O4	4.6480666666667	C22152;_	neg	319.13315685902	0.008043940431271	45.7	7380-40-7
neg_5428	Moroxydine	metab_36655	B(ii)	0	64.1	HMDB0254889	2M+FA-H	C6H13N5O	4.6480666666667	-	neg	387.22506168966	0.040738094464794	49.9	-
neg_5436	Galactoarabinan	metab_36663	B(ii)	0	55.9	HMDB0252576	M-H2O-H	C20H36O14	4.6480666666667	-	neg	481.19170484713	0.062701976384143	47.9	-
neg_5444	Lys-His-His	metab_36671	B(i)	46	0	-	M-H2O-H	C18H28N8O4	4.6534333333333	-	neg	401.20442961635	0.018257983943092	46.9	-
neg_5463	Myotoxin A	metab_36690	B(ii)	0	67.9	HMDB0254743	M-H	C29H38O9	4.6641166666667	-	neg	529.24207725539	0.066935667058392	50.5	-
neg_5468	Tyr-Phe-His	metab_36695	B(i)	36.1	0	-	M-H	C24H27N5O5	4.66945	-	neg	464.19434142581	0.021142448568146	45.5	-
neg_5481	Emivirine	metab_36708	B(ii)	0	69.5	HMDB0251775	2M-H	C17H22N2O3	4.6750833333333	-	neg	603.31522786931	0.052056598896489	51.8	-
neg_5488	Salsoline-1-Carboxylate	metab_36715	B(ii)	0	40.2	HMDB0013067	2M-H	C12H15NO4	4.6803666666667	-	neg	473.19086321457	0.041835182116361	46.2	31758-50-6
neg_5490	4-Hydroxy-2-Nonenal-[L-Cys] Conjugate	metab_36717	B(ii)	0	63.2	HMDB0304176	2M+FA-H	C12H23NO4S	4.6803666666667	-	neg	599.26885519654	0.040040130541186	48.9	-
neg_5495	Val-Thr-Leu	metab_36722	B(i)	98.7	0	-	M-H	C15H29N3O5	4.6856666666667	-	neg	330.20372193669	0.080140564271131	57.7	-
neg_5508	Triterpenoid	metab_36735	B(ii)	0	52.4	HMDB0004309	M+Cl	C30H48O7S	4.6910166666667	C06085	neg	587.28684856589	0.049958327630477	45.5	-;_
neg_5515	2-[[2-[(2-Aminoacetyl)Amino]-3-Phenylpropanoyl]Amino]Pentanedioic Acid	metab_36742	B(i)	47.6	0	-	M-H2O-H	C16H21N3O6	4.6963166666667	-	neg	332.12536757996	0.0086138129725549	47.2	-
neg_5518	Ganoderic Acid L	metab_36745	B(ii)	0	65	HMDB0035885	M+Cl	C30H46O8	4.6963166666667	-	neg	569.29387484301	0.028290933805066	48.9	102607-24-9
neg_5527	Plantamajoside	metab_36754	B(ii)	0	92.5	HMDB0256632	M-H	C29H36O16	4.7016666666667	_;C10485	neg	639.1934197	0.009224019460321	57.8	104777-68-6;_
neg_5530	Scillipheosidin 3-[Glucosyl-(1->2)-Rhamnoside]	metab_36757	B(ii)	0	74.1	HMDB0036946	M+Na-2H	C36H52O14	4.7016666666667	-	neg	729.3106112	0.10636029161823	53.5	261158-68-3
neg_5533	Thr-Val-Phe	metab_36760	B(i)	89.3	0	-	M-H	C18H27N3O5	4.707	-	neg	364.18792040425	0.009259751577469	56.9	-
neg_5549	Cinobufotalin	metab_36776	B(ii)	0	61.4	HMDB0250267	M-H2O-H	C26H34O7	4.7123333333333	C16932;_	neg	439.20864396904	0.004166934453001	49.8	1108-68-5;_;
neg_5562	(+/-)-Methyl 5-Acetoxyhexanoate	metab_36789	B(ii)	0	42	HMDB0032387	2M+FA-H	C9H16O4	4.7176833333333	-	neg	421.20952312199	0.020520017347718	45.9	35234-22-1
neg_5578	Pi(Pgf1Alpha/20:1(11Z))	metab_36805	B(ii)	0	90.2	HMDB0277164	M+Cl	C49H89O16P	4.72305	-	neg	999.54875516817	0.048370507979469	53.3	-
neg_5579	2-Glucosyloxy-4-Methoxycinnamic Acid	metab_36806	B(i)	95.1	0	-	M-H2O-H	C16H20O9	4.7283333333333	-	neg	337.09305578891	0.002694128380652	58.3	-

neg_5581	Chrysosplenol D	metab_36808	B(i)	67.6	0	-;LMPK12112998	M+FA-H	C18H16O8	4.7283333333333	C04552	neg	405.08042692528	0.04295437725179	51.1	14965-20-9;
neg_5590	(R)-Laudanidine	metab_36817	B(ii)	0	61	HMDB0030211	2M-H	C20H25NO4	4.7336666666667	C17592	neg	685.35316187387	0.013479475746968	49.6	301-21-3
neg_5600	N-(1-Benzylpiperidin-4-Yl)-6-Phenylthieno[3,2-D]Pyrimidin-4-	metab_36827	B(i)	51.7	0	-	M+FA-H	C24H24N4S	4.7442833333333	-	neg	445.17067284213	0.037307092621251	46.3	-
neg_5605	Dgdg(O-16:3/5:0)	metab_36832	B(i)	39.4	0	-	M+Cl	C36H62O14	4.7442833333333	-	neg	753.37817231754	0.058387647197031	44.2	-
neg_5607	Tunicamycin A	metab_36834	B(ii)	0	77.4	HMDB0259327	M-H2O-H	C38H62N4O16	4.7442833333333	-	neg	811.40048699848	0.039904919277304	50.9	-
neg_5612	5,6-Dihydroxytetradecanedioic Acid	metab_36839	B(i)	47.1	0	-	M-H	C14H26O6	4.7496	-	neg	289.16571372685	0.026795383387067	47.7	-
neg_5622	3-Carboxymedetomidine	metab_36849	B(i)	66.6	0	-	M-H	C13H14N2O2	4.7549	-	neg	229.09836257928	0.028109204138622	52.6	-
neg_5627	Implitapide	metab_36854	B(ii)	0	52	HMDB0259646	M-H	C35H37N3O2	4.7549	-	neg	530.28353364092	0.015040199367242	46.7	-
neg_5630	25-O-Deacetylrifabutin	metab_36857	B(ii)	0	54.8	HMDB0254133	M-H	C44H60N4O10	4.7549	-	neg	803.4264029	0.040277984736104	45.9	-
neg_5634	Trans-Cinnamoyl-Beta-D-Glucoside	metab_36861	B(ii)	0	62.3	HMDB0304505	M+FA-H	C15H18O7	4.76025	C04164	neg	355.10355127103	0.079646213534758	51.1	-
neg_5636	(2E,4E)-8-(Beta-D-Glucopyranosyloxy)-2,7-Dimethyldeca-2,4-Dienedioic Acid	metab_36863	B(i)	46.3	0	-	M-H	C18H28O10	4.76025	-	neg	403.16251495739	0.012095542473415	48	-
neg_5637	Chitobiose	metab_36864	B(ii)	0	64.8	HMDB0003556	M-H	C15H26N2O12	4.76025	C01674	neg	425.1443847	0.05059899115844	50	35061-50-8;577-76-4
neg_5644	Coproporphyrin I	metab_36871	B(ii)	0	62.9	HMDB0000643	M+Hac-H	C36H38N4O8	4.76025	C05769	neg	713.28620002133	0.066819759426704	48	531-14-6
neg_5649	Pi(O-16:4/22:6)	metab_36876	B(i)	91.8	0	-	M+FA-H	C47H73O12P	4.76025	-	neg	905.48229664135	0.01568469744447	56.2	-
neg_5651	Glu-Ala-Leu	metab_36878	B(i)	80.3	0	-	M-H2O-H	C14H25N3O6	4.76555	-	neg	312.15659296314	0.04356861620709	54.4	-
neg_5652	5'-((Z)-Feruloyl) 3-(2'-Methylarabinosylxylose)	metab_36879	B(ii)	0	61.6	HMDB0030231	M-H	C21H28O12	4.76555	-	neg	471.15012139366	0.054312026640756	50	-
neg_5653	A-L-Fucopyranosyl-(1->2)-B-D-Galactopyranosyl-(1->2)-D-	metab_36880	B(ii)	0	68.4	HMDB0041222	M+Cl	C17H30O14	4.76555	-	neg	493.13208133558	0.083769087026911	51.7	130136-27-5
neg_5657	Kynostatin 272	metab_36884	B(ii)	0	43.8	HMDB0253811	M+FA-H	C33H41N5O6S	4.76555	C15655;_	neg	712.24455278383	0.054596731754426	43.8	147318-81-8;_
neg_5667	(E)-6-Hydroxy-6-(1-Hydroxy-4-Methylcyclohex-3-En-1-Yl)-2-Methylhept-2-Enoic Acid	metab_36894	B(i)	35.6	0	-	M+Na-2H	C15H24O4	4.7762	-	neg	289.14064343078	0.031931180318478	44.5	-
neg_5670	Ile-Gln-Ile	metab_36897	B(i)	71.8	0	-	M+Hac-H	C17H32N4O5	4.7762	-	neg	431.25129987184	0.033867382230961	50.2	-
neg_5673	Protease-Activated Receptor-4	metab_36900	B(ii)	0	66.2	HMDB0256865	M-H	C33H46N8O7	4.7762	-	neg	665.34166771731	0.051428898356145	52.1	-
neg_5675	3-Dehydronobilin	metab_36902	B(ii)	0	63	HMDB0036691	2M+FA-H	C20H24O5	4.7762	-	neg	733.32923295496	0.079405201422809	48.3	63529-14-6
neg_5677	Sialyl Lewis X Antigen	metab_36904	B(ii)	0	82.8	HMDB0258280	M+FA-H	C31H52N2O23	4.7762	-	neg	865.296334	0.019782821712656	54.6	-
neg_5679	Ser-Val-Phe	metab_36906	B(i)	81.5	0	-	M-H	C17H25N3O5	4.78155	-	neg	350.17222938025	0.007790257742275	55.2	-
neg_5680	Acorafloxacin	metab_36907	B(ii)	0	50.4	HMDB0247964	M-H	C21H23F2N3O	4.78155	-	neg	418.15978977418	0.038649400869069	47.5	-
neg_5693	Glycitein 7-O-Glucuronide	metab_36920	B(ii)	0	48.2	HMDB0041741	M-H	C22H20O11	4.7868333333333	-	neg	459.0968121	0.21676636882778	45.3	-
neg_5703	Hydrodolasetron	metab_36930	B(ii)	0	42.8	HMDB0253248	M+Na-2H	C19H22N2O3	4.79215	-	neg	347.1349218	0.061564590519893	44.7	-
neg_5720	3,5,6-Trihydroxy-5-(Hydroxymethyl)-2-Methoxy-2-Cyclohexen-1-One	metab_36947	B(ii)	0	40.6	HMDB0041031	M+Na-2H	C8H12O6	4.79745	-	neg	225.0381813	0.080477666462156	46.5	76663-30-4
neg_5722	Tyr-Ser-Ile	metab_36949	B(i)	88.7	0	-	M-H	C18H27N3O6	4.79745	-	neg	380.18283691945	0.043051862043475	55.7	-
neg_5730	Gly-Val-Phe	metab_36957	B(i)	87.2	0	-	M-H	C16H23N3O4	4.8027833333333	-	neg	320.16170457792	0.027794541429682	56.3	-
neg_5737	Lpi(18:5)	metab_36964	B(i)	78.8	0	-	M+FA-H	C27H43O12P	4.8027833333333	-	neg	635.24514429006	0.013384716868414	53.7	-
neg_5740	Benzoic Acid	metab_36967	B(i)	52.2	0	-;HMDB0001870	M-H	C7H6O2	4.8081166666667	C00539;C00180	neg	121.02960551497	0.00457690555095	50.1	65-85-0
neg_5742	Z-Gly-Gly-Arg-Amc	metab_36969	B(ii)	0	46	HMDB0259965	M-H2O-H	C28H33N7O7	4.8081166666667	-	neg	560.2215147	0.029082254594299	45.8	-
neg_5745	Maitansine	metab_36972	B(ii)	0	57	HMDB0254306	M-H	C34H46ClN3O10	4.8081166666667	-	neg	690.27433029521	0.16724594277415	46.5	-
neg_5750	Guanosine, 2'-Deoxy-N-(7,8,9,10-Tetrahydro-7,8,9-Trihydroxybenzo(A)Pyren-10-Yl)-	metab_36977	B(ii)	0	52.7	HMDB0255493	M+Hac-H	C30H27N5O7	4.8134833333333	-	neg	628.20976425423	0.064504093166083	44.9	-
neg_5756	Cdp-Dg(Pgj2/I-12:0)	metab_36983	B(ii)	0	42.2	HMDB0293292	M-H2O-H	C44H71N3O17P2	4.8134833333333	-	neg	956.39891294849	0.13727823752217	41.5	-
neg_5762	Mabioside C	metab_36989	B(ii)	0	76.3	HMDB0040653	M+Cl	C42H64O14	4.8188166666667	-	neg	827.39513811587	0.021599718907858	48.9	156980-54-0
neg_5766	Alpha-Hydroxysalmeterol	metab_36993	B(ii)	0	61.5	HMDB0061045	M+Hac-H	C25H37NO5	4.8241666666667	-	neg	490.27733877669	0.065887172551017	48.7	-
neg_5786	Tyr-Pro-Leu-Gly-Nh2	metab_37013	B(ii)	0	69.2	HMDB0259345	M+Hac-H	C22H33N5O5	4.8401	-	neg	506.2616624	0.019550481582739	51.8	-
neg_5788	Neogitogenin 3-[Glucosyl-(1->2)-Glucosyl-(1->4)-Galactoside]	metab_37015	B(ii)	0	56.8	HMDB0041134	M-H	C45H74O19	4.8401	C10487	neg	917.47462867781	0.01855270250221	49.3	-
neg_5790	Valine-Betaxanthin	metab_37017	B(ii)	0	64.3	HMDB0304749	M-H	C14H18N2O6	4.8454666666667	-	neg	309.10693552181	0.010378061024885	50.1	-
neg_5793	Glu-Glu-Leu	metab_37020	B(i)	89	0	-	M-H2O-H	C16H27N3O8	4.8454666666667	-	neg	370.16211753115	0.008263889704497	56.3	-

neg_5804	5-(2-Methylpropyl)-3,6-Dioxo-2-Piperazinepropanoic Acid	metab_37031	B(i)	96.7	0	-	M-H	C11H18N2O4	4.8508	-	neg	241.11941021811	0.008636824052421	58.9	-
neg_5811	Dimerum Acid	metab_37038	B(ii)	0	54.5	HMDB0251372	M-H	C22H36N4O8	4.8508	-	neg	483.24618888717	0.019876764271167	50.4	-
neg_5833	Batrachotoxin	metab_37060	B(ii)	0	45.2	HMDB0248882	M+Cl	C31H42N2O6	4.8668	C13750;_	neg	573.27032459825	0.028837179488693	46.3	23509-16-2;_
neg_5837	Ile-Phe	metab_37064	B(i)	85.5	0	HMDB0028914	M-H	C15H22N2O3	4.87215	-	neg	277.15587093826	0.013316298770116	56.4	-
neg_5844	Ganoderic Acid I	metab_37071	B(ii)	0	46.9	HMDB0035310	M+FA-H	C30H44O8	4.87215	-	neg	577.30033487124	0.14105539462054	45.4	98665-20-4
neg_5862	Estrone Glucuronide	metab_37089	B(ii)	0	48.1	HMDB0004483	2M-H	C24H30O8	4.8827333333333	C11133	neg	891.38072243584	0.13682646862796	46.2	2479-90-5
neg_5863	2-Nonene-1,4-Diol	metab_37090	B(ii)	0	46.1	HMDB0245271	M+FA-H	C9H18O2	4.88805	-	neg	203.12897465008	0.021841595104836	48.2	-
neg_5864	Glu-Phe-Ser	metab_37091	B(i)	62.6	0	-	M-H	C17H23N3O7	4.88805	-	neg	380.14653638422	0.070226919903181	50.9	-
neg_5865	Tauroursodeoxycholic Acid	metab_37092	B(i)	77.3	0	-;HMDB0000874;LMST05040015	M-H	C26H45NO6S	4.88805	_;C16868	neg	498.29362489333	0.016471944669918	51.6	14605-22-2;
neg_5874	Hydroxyclomipramine	metab_37101	B(ii)	0	45.9	HMDB0060631	2M-H	C19H23ClN2O	4.89335	C00350	neg	659.298576	0.013143101508169	41.7	-
neg_5877	Phthalic Acid	metab_37104	B(i)	73.7	0	HMDB0002107	M-H	C8H6O4	4.89865	C01606	neg	165.01940491341	0.025565230604016	52.9	88-99-3
neg_5880	Vicenin Iii	metab_37107	B(i)	94.6	0	-	M-H	C26H28O14	4.89865	-	neg	563.14090977087	0.01001593228186	57.8	-
neg_5882	Trypanothione	metab_37109	B(ii)	0	80.4	HMDB0060520	M-H	C27H49N9O10S2	4.89865	C03170;C02090	neg	722.29519366683	0.073797040317281	51.9	-;_
neg_5883	Val-Val-Leu	metab_37110	B(i)	73	0	-	M-H	C16H31N3O4	4.9039333333333	-	neg	328.22437852787	0.001684023670162	53.4	-
neg_5884	Histidyltyrosine	metab_37111	B(ii)	0	45.5	HMDB0028897	M+Hac-H	C15H18N4O4	4.9039333333333	-	neg	377.14556263571	0.09289382269714	47.2	35979-00-1
neg_5888	Tyr-Gly-Gly-Trp-Leu	metab_37115	B(ii)	0	54.5	HMDB0259342	M+FA-H	C30H38N6O7	4.9039333333333	-	neg	639.28099363618	0.068534044654888	46.2	-
neg_5902	12-Oxo-C-Ltb3	metab_37129	B(ii)	0	44.8	HMDB0060154	M+Hac-H	C30H47N3O10	4.9092333333333	-	neg	700.31753680602	0.049357029748512	43.1	-
neg_5904	Ophiopogonin C	metab_37131	B(ii)	0	40.9	HMDB0029312	M+Na-2H	C39H62O12	4.9092333333333	C17043	neg	743.39947112525	0.15466204855251	41.9	-
neg_5931	Aureomycin	metab_37158	B(ii)	0	47	HMDB0248728	M-H2O-H	C22H23ClN2O	4.9252333333333	C06571;_	neg	459.09688522577	0.039800991306225	42.9	57-62-5;_
neg_5935	Capecitabine	metab_37162	B(ii)	0	79.7	HMDB0015233	2M-H	C15H22FN3O6	4.9252333333333	C12650	neg	717.28553980377	0.043015546012162	51.5	154361-50-9
neg_5938	(2-Oxo-2,3-Dihydro-1H-Indol-3-Yl)Acetic Acid	metab_37165	B(i)	93.6	0	-	M-H	C10H9NO3	4.9305833333333	-	neg	190.05111707723	0.025731013959971	55.6	-
neg_5939	Valsartan Acid	metab_37166	B(i)	60.9	0	-	M+Hac-H	C14H10N4O2	4.9305833333333	-	neg	325.09312968145	0.066522852610932	48	-
neg_5944	Shexcer(32:4_3O)	metab_37171	B(i)	45.6	0	-	M+Hac-H	C38H67NO12S	4.9305833333333	-	neg	820.45634437517	0.069396704586578	45.3	-
neg_5945	Asp-Val-Phe	metab_37172	B(i)	92.2	0	-	M-H	C18H25N3O6	4.93595	-	neg	378.16729364341	0.052553814884824	56.9	-
neg_5951	3-Carboxy-4-Methyl-5-Propyl-2-Furanpropanoic Acid	metab_37178	B(i)	85	0	LMFA01150004	M-H	C12H16O5	4.94125	-	neg	239.09261757175	0.024976838424709	56.2	0
neg_5952	Phenylalanylhydroxyproline	metab_37179	B(ii)	0	50	HMDB0011176	M+FA-H	C14H18N2O4	4.94125	-	neg	323.12515594125	0.086104242701615	48.7	90965-82-5
neg_5958	Nemifitide	metab_37185	B(ii)	0	50	HMDB0255508	M-H	C33H43FN10O	4.94125	-	neg	693.32207130584	0.029110492966572	42.2	-
neg_5964	Aconitinum	metab_37191	B(ii)	0	53.1	HMDB0247962	M-H	C34H47NO11	4.94655	-	neg	644.30808935142	0.039168034559245	47.4	-
neg_5966	Dimethyl C7	metab_37193	B(ii)	0	82.4	HMDB0251368	M+Hac-H	C43H61N9O9S	4.94655	-	neg	938.44678811811	0.085157666453149	52.7	-
neg_5970	(4-((5,6-Diphenylpyrazin-2-Yl)(Isopropyl)Amino)Butoxy)Acetic	metab_37197	B(ii)	0	57	HMDB0254922	M+FA-H	C25H29N3O3	4.95185	-	neg	464.21561285208	0.055831485214375	47.3	-
neg_5986	Alpha-Hederin	metab_37213	B(ii)	0	78.5	HMDB0248219	M+Na-2H	C41H66O12	4.9572	C08954;_	neg	771.43747351259	0.021727209231549	51.9	27013-91-8;_
neg_5990	Thr-Leu-Ile	metab_37217	B(i)	91.7	0	-	M-H	C16H31N3O5	4.9625	-	neg	344.21923857457	0.048848798430287	56.5	-
neg_5991	Glu-Thr-Phe	metab_37218	B(i)	90.5	0	-	M-H2O-H	C18H25N3O7	4.9625	-	neg	376.15142702752	0.15357705151423	56.3	-
neg_5998	Hordatine B Glucoside	metab_37225	B(ii)	0	51.8	HMDB0030460	M+FA-H	C35H50N8O10	4.9625	-	neg	787.36403319944	0.08654452831979	47.1	-
neg_6004	Isowertin 2''-Rhamnoside	metab_37231	B(ii)	0	56.4	HMDB0037417	M+FA-H	C28H32O14	4.96785	C12629	neg	637.17768851961	0.11154707827345	50.6	64821-00-7
neg_6008	Hydroxyl Frovatriptan	metab_37235	B(ii)	0	51	HMDB0061150	M+Hac-H	C14H19N3O2	4.9731833333333	-	neg	320.16174048186	0.012706598130167	49.7	-
neg_6009	Val-Thr-Phe	metab_37236	B(i)	85.5	0	-	M-H	C18H27N3O5	4.9731833333333	-	neg	364.18801940239	0.018157381664359	55.9	-
neg_6024	Sacubitrilat	metab_37251	B(ii)	0	45.4	HMDB0257440	M+Na-2H	C22H25NO5	4.9785333333333	-	neg	404.14665404375	0.027739673229207	45.7	-
neg_6029	Pisumoside A	metab_37256	B(ii)	0	50.9	HMDB0037126	M-H2O-H	C38H62O21	4.9785333333333	-	neg	835.36085152668	0.17425904507407	47.7	333334-42-2
neg_6030	Ac2Pim1(18:2/14:0)	metab_37257	B(i)	64.8	0	-	M+FA-H	C47H85O18P	4.9785333333333	-	neg	1013.5403	0.05591473694567	49.9	-
neg_6036	Goshonoside F6	metab_37263	B(ii)	0	50.8	HMDB0038542	M-H2O-H	C31H52O12	4.9838666666667	-	neg	597.32580167834	0.019092891673217	43.2	138614-60-5
neg_6053	Pi(Pgf1Alpha/18:1(9Z))	metab_37280	B(ii)	0	71	HMDB0276645	M+Cl	C47H85O16P	4.99455	-	neg	971.53259080499	0.019845673358841	51.8	-
neg_6055	Gly-Pro-Arg-Pro	metab_37282	B(ii)	0	81.7	HMDB0252829	M+Cl	C18H31N7O5	4.9999	-	neg	460.20594944952	0.039505925885944	53.9	-
neg_6065	Ps(Pgf2Alpha/18:2(9Z,12Z))	metab_37292	B(ii)	0	63.1	HMDB0281661	M+FA-H	C44H76NO13P	4.9999	-	neg	902.51081935476	0.069259599785257	48.5	-
neg_6068	Pro-Tyr-Ser	metab_37295	B(i)	50	0	-	M-H2O-H	C17H23N3O6	5.00525	-	neg	346.14094612729	0.023882471294096	48.4	-

neg_6073	Lidoflazine	metab_37300	B(ii)	0	42.4	HMDB0254083	M+Hac-H	C30H35F2N3O	5.00525	-	neg	550.28794234039	0.018752960182148	45.5	-
neg_6082	Quercetin-3-O-Deoxyhexosyl(1-2)Pentoside	metab_37309	B(i)	90.1	0	-	M+FA-H	C26H28O15	5.0107833333333	-	neg	625.1412997	0.002921864755148	57.4	-
neg_6093	Digitoxigenin 3-[Glucosyl-(1->6)-Glucosyl-(1->4)-2,6-Dideoxyribohexoside]	metab_37320	B(ii)	0	78.5	HMDB0034321	M+Cl	C41H64O17	5.0160833333333	-	neg	863.37937486512	0.0088553981152679	53.4	220710-06-5
neg_6097	Tryptophyl-Lysine	metab_37324	B(ii)	0	52.2	HMDB0029088	M+FA-H	C17H24N4O3	5.0214333333333	-	neg	377.18329168869	0.003412532133215	49.8	-
neg_6098	1-Octen-3-Yl Primeveroside	metab_37325	B(ii)	0	66.6	HMDB0032960	M-H	C19H34O10	5.0214333333333	-	neg	421.2095864	0.005866231866389	52	209863-01-4;
neg_6100	Ethyl 3-Hydroxyoctanoate O-[Glucosyl-(1->6)-Glucoside]	metab_37327	B(ii)	0	67.6	HMDB0038738	M-H	C22H40O13	5.0214333333333	-	neg	511.24118863068	0.08492266644946	52.1	-
neg_6106	Phenethyl Sophoroside	metab_37333	B(i)	90.1	0	-	M+FA-H	C20H30O11	5.0267833333333	-	neg	491.17723043459	0.057118293903631	57	-
neg_6111	Lobucavir	metab_37338	B(ii)	0	48.8	HMDB0254142	M-H2O-H	C11H15N5O3	5.0320833333333	-	neg	246.09846262136	0.030369598267153	47.1	-
neg_6120	Sayaendoside	metab_37347	B(i)	35.2	0	-	M+FA-H	C19H28O10	5.03745	-	neg	461.16676410537	0.052488229053782	45.6	-
neg_6122	8-Hydroxymirtazapine	metab_37349	B(ii)	0	49.2	HMDB0060957	2M+FA-H	C17H19N3O	5.03745	-	neg	607.30930447797	0.006677612333544	45.7	-
neg_6123	Erythratine	metab_37350	B(ii)	0	40.4	HMDB0030181	2M+FA-H	C18H21NO4	5.03745	-	neg	675.29572292969	0.053042721574234	45	5550-20-9
neg_6138	Sarcodon Scabrosus Depsipeptide	metab_37365	B(ii)	0	50.9	HMDB0041421	M+Cl	C23H39N3O8	5.0480833333333	-	neg	520.241627	0.019299632388404	49	-
neg_6145	Ala-Leu-Ile	metab_37372	B(i)	89.7	0	-	M-H	C15H29N3O4	5.0533833333333	-	neg	314.20871458308	0.006341681412459	56.7	-
neg_6152	Penitrem D	metab_37379	B(ii)	0	72.7	HMDB0256240	M+Hac-H	C37H45NO4	5.0586833333333	C20596;_	neg	626.35220729453	0.042750723575751	50.3	78213-64-6;_
neg_6156	(1S,2S,4R,8R)-P-Menthane-1,2,8,9-Tetrol	metab_37383	B(ii)	0	40.3	HMDB0039974	M-H	C10H20O4	5.064	-	neg	203.12899513589	0.031787567785497	47	402593-46-8
neg_6157	Pumiliotoxin A	metab_37384	B(ii)	0	51.6	HMDB0256924	M+Na-2H	C19H33NO2	5.064	C20033;_	neg	328.2243781	0.037118594156985	47.4	67054-00-6;_
neg_6158	Cotinine Glucuronide	metab_37385	B(ii)	0	51.8	HMDB0001013	M-H	C16H20N2O7	5.064	C00190;_	neg	351.11989778063	0.059649503385075	48.9	139427-57-9
neg_6164	16-Deacetylgeyerline	metab_37391	B(ii)	0	43.7	HMDB0244720	M+Na-2H	C36H48N2O10	5.064	-	neg	689.3014129	0.089979431976561	45.3	-
neg_6171	Scorzonoside	metab_37398	B(ii)	0	54.9	HMDB0038718	M-H	C27H34O12	5.0693	-	neg	549.19710156906	0.09984380111499	47.5	217650-81-2
neg_6186	Phe-Ile	metab_37413	B(i)	81.1	0	HMDB0028998	M-H	C15H22N2O3	5.0799666666667	-	neg	277.15588078553	0.005946344324863	55.5	22951-94-6
neg_6190	Cadabicine	metab_37417	B(ii)	0	43.1	HMDB0033451	M-H2O-H	C25H29N3O4	5.0799666666667	-	neg	416.19401205203	0.005265996611806	45.2	99964-83-7
neg_6193	Blumenol C O-[Rhamnosyl-(1->6)-Glucoside]	metab_37420	B(ii)	0	72.5	HMDB0031935	M-H	C25H42O11	5.0799666666667	-	neg	517.26327404135	0.066513371355798	48.3	177261-67-5
neg_6201	Aclacinomycin A Zwitterion	metab_37428	B(ii)	0	40.9	HMDB0247943	M+Na-2H	C42H53NO15	5.0799666666667	-	neg	832.32417376481	0.078367621314137	43.4	-
neg_6207	Corymboside	metab_37434	B(i)	86.3	0	HMDB0030803;LMPK12110260	M-H	C26H28O14	5.0852666666667	-	neg	563.14086767671	0.045642969602012	55.4	73543-87-0;
neg_6214	Dihydroouabain	metab_37441	B(ii)	0	67.3	HMDB0251322	M+Na-2H	C29H46O12	5.0905666666667	-	neg	607.27381854664	0.014814066060943	52.6	-
neg_6218	Phe Thr Glu Lys	metab_37445	B(i)	53.7	0	-	M-H	C24H37N5O8	5.0958833333333	-	neg	522.25686213327	0.11360604613048	48.2	-
neg_6228	Divanillyltetrahydrofuran Ferulate	metab_37455	B(ii)	0	63.4	HMDB0032730	M+Na-2H	C30H32O8	5.1011833333333	-	neg	541.18068005712	0.07567772621752	48.2	96917-11-2
neg_6229	17-Aag	metab_37456	B(ii)	0	71.3	HMDB0244762	M-H2O-H	C31H43N3O8	5.1011833333333	-	neg	566.28237045613	0.19148004984982	48.4	-
neg_6238	Foliandrin	metab_37465	B(ii)	0	69.9	HMDB0252427	M+FA-H	C32H48O9	5.1065333333333	-	neg	621.32544306443	0.10739099203544	50.1	-
neg_6246	Galangin 7-Rhamnoside	metab_37473	B(ii)	0	45.5	HMDB0303610	M+FA-H	C21H20O9	5.1118333333333	-	neg	461.11242616845	0.007897542044316	45.9	-
neg_6247	Taurodeoxycholic Acid	metab_37474	B(ii)	0	50.9	HMDB0000896	M-H	C26H45NO6S	5.1118333333333	C05463;_	neg	498.29359918268	0.015250512606061	46.2	-;516-50-7;
neg_6261	Ganoderic Acid N	metab_37488	B(ii)	0	64.3	HMDB0035325	M+FA-H	C30H42O8	5.1171833333333	-	neg	575.28615638899	0.040285449349178	50.5	110241-19-5
neg_6271	Buprenorphine 3-Beta-D-Glucuronide	metab_37498	B(ii)	0	51.8	HMDB0249443	M+Hac-H	C35H49NO10	5.1225333333333	-	neg	702.34704516238	0.08654398470525	48.5	-
neg_6276	(All-E)-1,8,10-Heptadecatriene-4,6-Diyne-3,12-Diol	metab_37503	B(ii)	0	66.9	HMDB0039737	2M+FA-H	C17H22O2	5.1278333333333	-	neg	561.3258519	0.10018505872906	50.6	143966-10-3
neg_6277	Poncirin	metab_37504	B(ii)	0	84.5	HMDB0037487	M+Hac-H	C28H34O14	5.1278333333333	C09830	neg	653.20898566227	0.12471346463356	56	14941-08-3;
neg_6286	25-Hydroxyvitamin D2-25-Glucuronide	metab_37513	B(ii)	0	61	HMDB0010342	M+FA-H	C34H52O8	5.1331833333333	-	neg	633.3604925	0.046093921336595	48.8	-
neg_6292	Pa(Pge1/I-13:0)	metab_37519	B(ii)	0	64.5	HMDB0267508	M+Cl	C36H65O11P	5.1385166666667	-	neg	739.40014264232	0.10865433005648	49	-
neg_6295	Dakh Peptide	metab_37522	B(ii)	0	60.9	HMDB0250831	M+Cl	C47H62N10O1	5.1385166666667	-	neg	1009.4133674353	0.049117595864054	48.2	-
neg_6297	4-[(2,4-Dihydroxy-3,3-Dimethylbutanoyl)Amino]Butanoic Acid	metab_37524	B(ii)	0	54.4	HMDB0246316	M+Na-2H	C10H19NO5	5.14385	-	neg	254.10118001322	0.006568626436675	49.7	-
neg_6303	3-Tert-Butyladipic Acid	metab_37530	B(i)	49.5	0	-	M-H	C10H18O4	5.1491833333333	-	neg	201.11333979486	0.02964890594305	48.6	-
neg_6319	Fluocortin	metab_37546	B(ii)	0	43	HMDB0252349	2M+FA-H	C22H27FO5	5.1598333333333	C21553;_	neg	825.36378011011	0.090051061580827	46.3	33124-50-4;_
neg_6330	Ile-Thr-Leu	metab_37557	B(i)	92.9	0	-	M-H	C16H31N3O5	5.1704166666667	-	neg	344.21916311613	0.016851467319787	57.3	-
neg_6337	(3B,20R,22R)-3,20,27-Trihydroxy-1-Oxowitha-5,24-Dienolide 3-Glucoside	metab_37564	B(ii)	0	79.7	HMDB0033573	M-H	C34H50O11	5.1704166666667	-	neg	633.32620379091	0.033796542269755	52.8	227179-09-1
neg_6349	Equol	metab_37576	B(i)	53.3	0	HMDB0002209;HMDB0243678;LMPK12080003	M+Hac-H	C15H14O3	5.1810833333333	C14131;_	neg	301.1100942	0.16587565448178	46.8	531-95-3;_;

neg_6380	Ethyl 1-(4-Tert-Butylphenyl)-5-Cyano-4-Methyl-6-Oxopyridazine-3-Carboxylate	metab_37607	B(i)	73.6	0	-	M+FA-H	C19H21N3O3	5.1917333333333	-	neg	384.15425268954	0.097974560348108	51.3	-
neg_6389	Dihydroferulic Acid	metab_37616	B(i)	85.6	0	HMDB0062121	M-H	C10H12O4	5.1970833333333	-	neg	195.06635410431	0.027919967723152	55.4	1135-23-5
neg_6390	Creatinine Aspartate	metab_37617	B(ii)	0	45	HMDB0250526	M+Cl	C8H10N4O5	5.1970833333333	-	neg	277.03310920185	0.046878013477617	46.2	-
neg_6392	Ustiloxin D	metab_37619	B(ii)	0	72.6	HMDB0041054	M+Hac-H	C23H34N4O8	5.1970833333333	-	neg	553.25170339232	0.019697447968831	53.2	158243-18-6
neg_6395	Asn-Leu-Leu	metab_37622	B(i)	82.6	0	-	M-H	C16H30N4O5	5.2024333333333	-	neg	357.21447890599	0.0145120657195	55.1	-
neg_6405	Granotapide	metab_37632	B(ii)	0	59.3	HMDB0252935	M+Hac-H	C39H37F3N2O	5.20775	-	neg	777.25859586183	0.030904054123206	46.7	-
neg_6408	Catechin Gallate	metab_37635	B(i)	89.1	0	HMDB0029255	M-H	C22H18O10	5.21305	-	neg	441.0826731	0.05731816467048	55.1	-
neg_6414	Pi(17:2/18:5)	metab_37641	B(i)	91.7	0	-	M+FA-H	C44H71O13P	5.21305	-	neg	883.46599542296	0.03838711035362	54.2	-
neg_6425	Verbascoside	metab_37652	B(i)	88.2	0	HMDB0034843;HMDB0259785	M-H	C29H36O15	5.2236333333333	C10501;_	neg	623.19833964752	0.010401707028879	57.5	61276-17-3;_
neg_6431	Asp-Ile-Leu	metab_37658	B(i)	92.6	0	-	M-H	C16H29N3O6	5.2289833333333	-	neg	358.19877483747	0.020777439096538	56.7	-
neg_6433	Lobetyolin	metab_37660	B(ii)	0	78.9	HMDB0254141	M+Hac-H	C20H28O8	5.2289833333333	-	neg	455.19366063276	0.06928372100636	54.5	-
neg_6447	Amarogentin	metab_37674	B(ii)	0	40.2	HMDB0248275	M+Hac-H	C29H30O13	5.2342833333333	_;C09767	neg	645.18010166079	0.009575964339253	46.9	21018-84-8;_
neg_6461	Piperalol	metab_37688	B(ii)	0	50.8	HMDB0035767	M+Na-2H	C15H24O3	5.2449	-	neg	273.14575457001	0.090108907972445	47.2	100288-35-5
neg_6465	Pa(Pgj2/I-12:0)	metab_37692	B(ii)	0	78	HMDB0267348	M+FA-H	C35H59O10P	5.2449	-	neg	715.37904236265	0.16281998187957	53.5	-
neg_6475	Cilastatin	metab_37702	B(ii)	0	51.2	HMDB0015535	M+Na-2H	C16H26N2O5S	5.25555	C01675	neg	379.12772610634	0.039466699284046	46.7	82009-34-5
neg_6488	Leu-Thr-Ile	metab_37715	B(i)	96.7	0	-	M-H	C16H31N3O5	5.2662166666667	-	neg	344.21913153005	0.046435583346547	58.1	-
neg_6513	4-Hydroxyphenylacetylglutamic Acid	metab_37740	B(ii)	0	44.9	HMDB0006061	M-H	C13H15NO6	5.2821166666667	C05595	neg	280.08281203784	0.028996577001207	46.9	-;1029120-37-
neg_6517	Benzyloxycarbonyl-Val-Ala-Asp(Ome)-Fluoromethylketone	metab_37744	B(ii)	0	69.9	HMDB0244824	M+FA-H	C22H30FN3O7	5.2821166666667	-	neg	512.2040889	0.013167725892814	53	-
neg_6524	Bolton-Hunter Reagent	metab_37751	B(ii)	0	42.5	HMDB0246176	M-H	C13H13NO5	5.28745	-	neg	262.07224674178	0.10340272758336	46.6	-
neg_6528	Amastatin	metab_37755	B(ii)	0	67.1	HMDB0248277	M+Hac-H	C21H38N4O8	5.28745	C01552;_	neg	533.28295975033	0.007268908875773 3	53	67655-94-1;69400-55-
neg_6545	Bafilomycin A1(Baf-A1)	metab_37772	B(ii)	0	41.1	HMDB0247559	M+Cl	C35H58O9	5.2928166666667	-	neg	657.38335205294	0.23279133378589	43.5	-
neg_6550	Glu-Trp	metab_37777	B(i)	79.2	0	HMDB0028830	M-H2O-H	C16H19N3O5	5.29815	-	neg	314.11472127818	0.0125108146442	54.9	38101-59-6
neg_6553	Ser-Ser-Arg	metab_37780	B(i)	75.5	0	-	M+FA-H	C12H24N6O6	5.29815	-	neg	393.1746961	0.052683782758681	53.1	-
neg_6564	Pip(5-Iso Pgf2Vi/16:2(9Z,12Z))	metab_37791	B(ii)	0	71	HMDB0278668	M-H2O-H	C43H74O19P2	5.3034666666667	-	neg	937.418146	0.21865063013054	51.2	-
neg_6570	Leu-Gln-Leu	metab_37797	B(i)	93.4	0	-	M-H	C17H32N4O5	5.3141	-	neg	371.2300909	0.002612894859111	58	-
neg_6600	Nummularine B	metab_37827	B(ii)	0	52.3	HMDB0029334	M-H	C32H41N5O6	5.3300666666667	-	neg	590.30418653964	0.030522999213436	45.9	53947-96-9
neg_6606	Ile-Ser-Leu	metab_37833	B(i)	95.8	0	-	M-H	C15H29N3O5	5.33535	-	neg	330.20364057584	0.023513798761431	57.7	-
neg_6609	Epothilone D	metab_37836	B(ii)	0	89.1	HMDB0251876	M+Cl	C27H41NO5S	5.33535	C12039;_	neg	526.24033641723	0.01118581792134	55.1	189453-10-
neg_6618	Tyr-Val-Phe	metab_37845	B(i)	75.3	0	-	M-H	C23H29N3O5	5.3409	-	neg	426.20336893758	0.010510268237271	52.6	-
neg_6630	Saquinavir-No	metab_37857	B(ii)	0	57.9	HMDB0257486	M+Na-2H	C38H49N7O7	5.3462166666667	-	neg	736.3395148	0.094595667634848	47.2	-
neg_6641	2-(5-Carboxypentanoylamino)Benzoic Acid	metab_37868	B(i)	75.9	0	-	M-H	C13H15NO5	5.3568	-	neg	264.08782032567	0.07251190998143	53.6	-
neg_6659	Pa(6 Keto-Pgf1Alpha/8:0)	metab_37886	B(ii)	0	64	HMDB0266616	M+Na-2H	C31H55O12P	5.3621	-	neg	671.31597913246	0.064878863143075	50.4	-
neg_6662	Seglitide	metab_37889	B(ii)	0	88.4	HMDB0258209	M-H2O-H	C44H56N8O7	5.3621	-	neg	789.41529657453	0.01900625939143	53.8	-
neg_6664	Desacetyllevonantradol	metab_37891	B(ii)	0	43.3	HMDB0251028	M-H2O-H	C25H33NO3	5.3673833333333	-	neg	376.2242824	0.033375129489079	43.7	-
neg_6665	Boc-Pro-Phe(Nme)-Gly-Ome	metab_37892	B(i)	41.5	0	-	M-H	C23H33N3O6	5.3673833333333	-	neg	446.22619884909	0.069677280930167	42.1	-
neg_6667	(R)-6'-O-(4-Geranyloxy-2-Hydroxycinnamoyl)-Marmin	metab_37894	B(ii)	0	74.5	HMDB0039061	M+Na-2H	C38H46O8	5.3673833333333	-	neg	651.29981534513	0.10669007668718	49.5	142628-36-2
neg_6675	Dihydroergocornine	metab_37902	B(ii)	0	74.9	HMDB0251308	M+Na-2H	C31H41N5O5	5.3727333333333	-	neg	584.28427196383	0.028173943553542	52.7	-
neg_6691	N-Acetyl-L-Phenylalanine	metab_37918	B(i)	92.9	0	HMDB0000512	M-H	C11H13NO3	5.38335	C03519	neg	206.08234764265	0.012235680557679	58.1	2018-61-3
neg_6694	Carapin	metab_37921	B(i)	35.1	0	-	M+Na-2H	C27H32O7	5.38335	-	neg	489.18810463242	0.20680428775983	44.8	-
neg_6704	4-Acetyl-3-Hydroxy-5-Methylphenyl Beta-D-Glucopyranoside	metab_37931	B(i)	79.1	0	-	M-H	C15H20O8	5.3887	-	neg	327.10871916018	0.082536659067673	54.4	-
neg_6710	Kiwiionoside	metab_37937	B(ii)	0	74.5	HMDB0038691	M+FA-H	C19H34O9	5.3887	-	neg	451.21994915797	0.022906699338309	53.6	141897-12-3
neg_6719	D-3-Phenyllactic Acid	metab_37946	B(i)	99.5	0	HMDB0000563	M-H	C9H10O3	5.39405	C05607	neg	165.0557314	0.009453679165562	59.7	7326-19-4
neg_6724	D-Malic Acid P-Coumarate	metab_37951	B(ii)	0	50.1	HMDB0303755	M+Na-2H	C13H12O7	5.39405	-	neg	301.03146323686	0.014910202892585	47.7	-
neg_6725	Bisdemethoxycurcumin	metab_37952	B(ii)	0	52.4	HMDB0002114	M+FA-H	C19H16O4	5.39405	C17743;_	neg	353.10074461689	0.036991424291521	48	24939-16-0

neg_6741	Methionyl-Arginine	metab_37968	B(ii)	0	46	HMDB0028967	M+FA-H	C11H23N5O3S	5.39935	-	neg	350.15114585677	0.017462549666706	46.6	-
neg_6750	Alliospiroside C	metab_37977	B(ii)	0	49	HMDB0030914	M+Na-2H	C38H60O13	5.39935	-	neg	745.37103757218	0.029913263578292	44.3	114317-57-6
neg_6753	Ketoisoleucine	metab_37980	B(i)	73.1	0	-	M+Hac-H	C6H10O3	5.4046833333333	-	neg	189.07694516648	0.039333072830984	54.2	-
neg_6763	Periandrin I	metab_37990	B(ii)	0	53.3	HMDB0034522	M-H2O-H	C42H62O16	5.4046833333333	-	neg	803.39398296973	0.005956964978501	45.8	73565-59-0
neg_6764	Indole-3-Butyric Acid	metab_37991	B(i)	37.3	0	HMDB0002096	M-H2O-H	C12H13NO2	5.4100166666667	C11284	neg	184.07691262821	0.14486006052614	45.9	133-32-4
neg_6765	Phe-Gly-Leu	metab_37992	B(i)	46.6	0	-	M-H	C17H25N3O4	5.4100166666667	-	neg	334.17742982068	0.058418098253379	47.6	-
neg_6770	Leu-Glu-Leu	metab_37997	B(i)	67.2	0	-	M-H	C17H31N3O6	5.4153333333333	-	neg	372.21412160879	0.028631730842817	51.6	-
neg_6782	Gln-Phe-Ile	metab_38009	B(i)	39.6	0	-	M-H	C20H30N4O5	5.42065	-	neg	405.21443404979	0.02666723381783	47.1	-
neg_6785	Adouetine Y	metab_38012	B(ii)	0	49.6	HMDB0034101	M-H2O-H	C34H40N4O4	5.42065	C09994	neg	549.28344996318	0.066024006286106	45.8	19542-38-2
neg_6790	Ferulic Acid	metab_38017	B(i)	98.5	0	HMDB0000954	M-H	C10H10O4	5.426	C01494	neg	193.05073887009	0.011593844122889	59	537-98-4
neg_6796	Spinacetin 3-[P-Coumaroyl-(->2)-Glucosyl-(1->6)-[Apiosyl-(1->2)]-Glucoside]	metab_38023	B(ii)	0	46	HMDB0031426	M-H2O-H	C43H48O24	5.426	-	neg	929.23915369131	0.012356057653632	47.3	-
neg_6817	4-Benzofuranacetamide, N-Methyl-N-(7-(1-Pyrrolidinyl)-1-Oxaspiro(4.5)Dec-8-Yl)-	metab_38044	B(ii)	0	62.4	HMDB0249570	M+FA-H	C24H32N2O3	5.44205	-	neg	441.23556968853	0.030441886230851	48	-
neg_6823	(+/-)-Threo-1-(P-Hydroxyphenyl)Propylene Glycol 4'-Glucoside	metab_38050	B(ii)	0	49.4	HMDB0033068	2M+FA-H	C15H22O8	5.44205	-	neg	705.2655543	0.17124372399862	47	-
neg_6824	5-Methyltetrahydropteroyltri-L-Glutamic Acid	metab_38051	B(ii)	0	64.1	HMDB0012177	M-H	C30H39N9O12	5.44205	C04489	neg	716.26596739559	0.047092337499844	50.2	133321-34-3
neg_6829	Forsythiaside	metab_38056	B(ii)	0	60.2	HMDB0252460	M+Na-2H	C29H36O15	5.4474	C10456;_	neg	645.18032068194	0.039595841652581	49.8	79916-77-1;_
neg_6836	Asp-Leu-His	metab_38063	B(i)	43.4	0	-	M+FA-H	C16H25N5O6	5.4527333333333	-	neg	428.1803168	0.081393093789266	46.2	-
neg_6842	Pe(6 Keto-Pgf1Alpha/18:4(6Z,9Z,12Z,15Z))	metab_38069	B(ii)	0	70.3	HMDB0261743	M+Cl	C43H72NO12P	5.4527333333333	-	neg	860.45249231895	0.018915721623649	51.9	-
neg_6845	Ile-Trp	metab_38072	B(i)	71.4	0	HMDB0028918	M-H	C17H23N3O3	5.4580833333333	-	neg	316.16678903989	0.009999529458795	53.3	-
neg_6847	Glu-Phe-Leu	metab_38074	B(i)	92.4	0	-	M-H	C20H29N3O6	5.4580833333333	-	neg	406.19860722007	0.002188385991286	57.1	-
neg_6851	Vitamin P	metab_38078	B(i)	82.6	0	HMDB0003249	M-H	C27H30O16	5.4580833333333	C05625	neg	609.14661036389	0.036026801740118	55.3	153-18-4
neg_6865	Sonchuionoside C	metab_38092	B(ii)	0	73.9	HMDB0035212	2M+FA-H	C19H30O8	5.4633666666667	-	neg	817.38547506389	0.032750745978472	51.2	126585-76-0
neg_6866	Basellasaponin D	metab_38093	B(ii)	0	91.1	HMDB0039402	M-H	C47H68O22	5.4633666666667	-	neg	983.42156769421	0.048279713897214	54.5	354552-06-0
neg_6885	Kudzusaponin Sa4	metab_38112	B(ii)	0	77.2	HMDB0031353	M+Cl	C47H74O20	5.4793666666667	-	neg	993.45373914327	0.031055439935609	51.9	179910-78-2
neg_6886	Tranexamic Acid	metab_38113	B(ii)	0	74.9	HMDB0014447	M+FA-H	C8H15NO2	5.4847333333333	C12535	neg	202.10856242426	0.008980285235345	54.7	1197-18-8
neg_6890	Nivacortol	metab_38117	B(ii)	0	52.3	HMDB0255670	M+FA-H	C28H31FN2O	5.4847333333333	-	neg	475.24117991944	0.018430295419711	47.4	-
neg_6893	Neoacrimarine H	metab_38120	B(ii)	0	80.9	HMDB0033031	M+Hac-H	C33H29NO8	5.4847333333333	-	neg	626.20762943063	0.098646224431393	49	217199-06-9
neg_6896	Scutellarioside Ii	metab_38123	B(i)	42	0	-	M+Hac-H	C24H28O12	5.4900333333333	-	neg	567.17209182063	0.069816552144158	47.9	-
neg_6901	Troxerutin	metab_38128	B(ii)	0	75.8	HMDB0006083	M-H2O-H	C33H42O19	5.4900333333333	C05625;_	neg	723.21442844229	0.065980972956736	52.9	153-18-4;7085-55-4
neg_6909	Cucurbitacin B	metab_38136	B(ii)	0	85.8	HMDB0034927	M+FA-H	C32H46O8	5.4953666666667	C08794	neg	603.31499978201	0.014904283596471	55.4	6199-67-3;
neg_6913	Lyciumoside Ii	metab_38140	B(ii)	0	61.2	HMDB0039576	M+FA-H	C38H64O17	5.4953666666667	-	neg	837.41510557112	0.070858053004498	49.2	150396-01-3
neg_6918	Enterocin 900	metab_38145	B(ii)	0	47.2	HMDB0038284	M+Cl	C31H33NO2	5.5007166666667	-	neg	486.22215174499	0.059902581906269	45.5	179530-08-6
neg_6920	Demethoxycentaureidin 7-O-Rutinoside	metab_38147	B(i)	55.9	0	-	M-H	C29H34O16	5.5007166666667	-	neg	637.17778776927	0.18001610419321	48.8	-
neg_6924	Suberic Acid	metab_38151	B(i)	92.1	0	HMDB0000893;LMFA01170001	M-H	C8H14O4	5.5060166666667	C08278	neg	173.08203706285	0.007851190909919	57.6	505-48-6;
neg_6925	(1R,2R,3S,1'R)-Nepetalinic Acid	metab_38152	B(i)	86.2	0	HMDB0034971	M-H	C10H16O4	5.5060166666667	-	neg	199.09763666894	0.006726288014563	56	32603-11-5
neg_6939	Vinblastine	metab_38166	B(ii)	0	72.3	HMDB0014710	M+Na-2H	C46H58N4O9	5.5116166666667	C07201	neg	831.40116847051	0.014467875829386	50.8	865-21-4
neg_6945	Arginyl-Prolyl-Proline	metab_38172	B(ii)	0	66	HMDB0249363	M+Cl	C16H28N6O4	5.5169666666667	-	neg	403.18357988813	0.055249238337428	49.1	-
neg_6953	Substance P(1-7)	metab_38180	B(ii)	0	81.4	HMDB0258530	M-H2O-H	C41H65N13O1	5.5169666666667	-	neg	880.47808632445	0.053704841659514	52.9	-
neg_6957	Benzoylmesaconine	metab_38184	B(ii)	0	77.1	HMDB0249053	M+FA-H	C31H43NO10	5.5223166666667	-	neg	634.28447873059	0.044095625362788	52.2	-
neg_6964	4-O-P-Coumaroylquinic Acid	metab_38191	B(ii)	0	43	HMDB0302093	2M-H	C16H18O8	5.5276166666667	C10441	neg	675.19077592289	0.040182477479156	45.9	-
neg_6967	N6-[2-(4-Aminophenyl)Ethyl]Adenosine	metab_38194	B(ii)	0	45	HMDB0248518	2M-H	C18H22N6O4	5.5276166666667	-	neg	771.32882683019	0.059859122883995	45.9	-
neg_6975	4-Nitrophenyl 4-(3-Phenoxybenzyl)Piperazine-1-Carboxylate	metab_38202	B(ii)	0	46.6	HMDB0253750	M+Cl	C24H23N3O5	5.5382833333333	-	neg	468.13353119812	0.043182566433467	48	-
neg_6983	Desacetoxyvindoline	metab_38210	B(ii)	0	51.8	HMDB0304331	M+Hac-H	C23H30N2O4	5.5436333333333	C02673;_	neg	457.23076114124	0.039463923948699	46.1	-;_
neg_6987	Pa(5-Iso Pgf2Vi/18:3(6Z,9Z,12Z))	metab_38214	B(ii)	0	57.4	HMDB0264142	M+Cl	C39H65O11P	5.5436333333333	-	neg	775.39758908793	0.088860511251601	47	-
neg_6992	Mauritine A	metab_38219	B(ii)	0	44.3	HMDB0029338	M+Cl	C32H41N5O5	5.54895	-	neg	610.28196703964	0.072172299556595	44.1	38478-72-7

neg_6998	Harmine	metab_38225	B(ii)	0	43.3	HMDB0030311	2M-H	C13H12N2O	5.55425	C06538	neg	423.18106904375	0.24936500668625	47.5	442-51-3
neg_7002	Arphamenine B	metab_38229	B(ii)	0	57	HMDB0248615	2M+FA-H	C16H24N4O4	5.55425	-	neg	717.35807746617	0.019294276881243	47.7	-
neg_7005	N-Acetyl-L-Tryptophan	metab_38232	B(i)	84.2	0	HMDB0013713	M-H	C13H14N2O3	5.55955	-	neg	245.09331629243	0.028556795284627	55.5	1218-34-4
neg_7006	Mono(2-Ethyl-5-Hydroxyhexyl)Terephthalate	metab_38233	B(i)	53.6	0	-	M+FA-H	C16H22O5	5.55955	-	neg	339.1426147	0.055682684153718	47.3	-
neg_7008	Hyaluronan Biosynthesis, Precursor 1	metab_38235	B(ii)	0	44.4	HMDB0060071	M+FA-H	C14H21NO11	5.55955	C04794	neg	424.10722577108	0.006217741907692	46.6	-
neg_7021	Biie-0246	metab_38248	B(ii)	0	86.9	HMDB0249205	M-H	C49H57N11O6	5.5648666666667	-	neg	894.4366298	0.007598900111087	54.1	-
neg_7034	Indole-3-Lactic Acid	metab_38261	B(i)	97.8	0	HMDB0000671	M-H	C11H11NO3	5.5808166666667	C02043	neg	204.0666959	0.024266317893224	59.2	7417-65-4
neg_7035	Leu-Val-Leu	metab_38262	B(i)	90	0	-	M-H	C17H33N3O4	5.5808166666667	-	neg	342.23988654808	0.031850206801889	56.8	-
neg_7042	Coproporphyrinogen Iii	metab_38269	B(ii)	0	53.8	HMDB0001261	M-H	C36H44N4O8	5.5808166666667	C03263	neg	659.30469747848	0.060955475781017	46.5	2624-63-7
neg_7045	Isoquercetin	metab_38272	B(i)	92.5	0	HMDB0037362	M-H	C21H20O12	5.5861166666667	C05623	neg	463.08833543952	0.046755457186428	55.6	482-35-9
neg_7053	Ile-Trp-Pro	metab_38280	B(i)	50.7	0	-	M-H	C22H30N4O4	5.5917166666667	-	neg	413.21966501117	0.048727420116823	48.2	-
neg_7087	Phenazopyridine	metab_38314	B(ii)	0	46.1	HMDB0015506	2M-H	C11H11N5	5.6023666666667	C07429	neg	425.19416917513	0.073103683454118	48.3	94-78-0
neg_7089	Tezampanel	metab_38316	B(ii)	0	44.1	HMDB0258944	2M-H	C13H21N5O2	5.6023666666667	-	neg	557.33051445664	0.037803319482992	46.9	-
neg_7090	Goshonoside F7	metab_38317	B(ii)	0	69.7	HMDB0038378	M-H2O-H	C32H54O12	5.6023666666667	-	neg	611.3405136	0.04653785498637	50.1	-
neg_7094	Scytalone	metab_38321	B(i)	83.5	0	-	M-H	C10H10O4	5.6076666666667	C00779	neg	193.05069994525	0.098607171669428	54.9	49598-85-8
neg_7095	Gly Leu Phe	metab_38322	B(i)	88.2	0	-	M-H	C17H25N3O4	5.6076666666667	-	neg	334.17732750341	0.040659248113851	56.3	-
neg_7111	3-[(3-(2-Carboxyethyl)-4-Methylpyrrol-2-Yl)Methylene]-2-	metab_38338	B(ii)	0	41.3	HMDB0258526	2M-H	C17H16N2O3	5.6183166666667	-	neg	591.22944913695	0.062765811751809	44.5	-
neg_7115	2,2-Bis[4-(2,3-Epoxypropoxy)Phenyl]Propane	metab_38342	B(ii)	0	40.5	HMDB0032737	2M+FA-H	C21H24O4	5.6183166666667	C14348	neg	725.32678563756	0.071822627866528	44.7	1675-54-3
neg_7125	Kaempferol 3-O-Feruloyl-Sophoroside 7-O-Glucoside	metab_38352	B(ii)	0	42.9	HMDB0029269	M-H2O-H	C43H48O24	5.6236166666667	-	neg	929.23920184693	0.088522393489374	46	-
neg_7128	Asp-Phe-Leu	metab_38355	B(i)	93.6	0	-	M-H	C19H27N3O6	5.6289166666667	-	neg	392.18277666856	0.017976012047799	58.2	-
neg_7129	Phe-Ser-Tyr	metab_38356	B(i)	58.1	0	-	M-H	C21H25N3O6	5.6289166666667	-	neg	414.16476443358	0.011237355482261	49	-
neg_7133	L-Pyridosine	metab_38360	B(ii)	0	43.4	HMDB0029443	2M+FA-H	C12H18N2O4	5.6289166666667	-	neg	553.25180935522	0.02407920877655	46.8	31489-08-4
neg_7134	Meproscillarin	metab_38361	B(ii)	0	79.1	HMDB0254457	M+Hac-H	C31H44O8	5.6289166666667	-	neg	603.31531953002	0.022312506588396	54.1	-
neg_7140	Tryptophyl-Phenylalanine	metab_38367	B(ii)	0	70.9	HMDB0029090	M-H	C20H21N3O3	5.63425	-	neg	350.1510572	0.010444794975452	53.2	-
neg_7146	Phe-Gln-Leu	metab_38373	B(i)	68.8	0	-	M-H	C20H30N4O5	5.63955	-	neg	405.21448695306	0.01422260666211	51.8	-
neg_7147	Kinetensin 4-7	metab_38374	B(ii)	0	44.1	HMDB0012986	M+Na-2H	C26H37N9O6	5.63955	-	neg	592.26225745799	0.025203323506105	45.5	138482-56-1
neg_7158	1,2,3,4-Tetrahydroisoquinoline-1-Carboxylic Acid	metab_38385	B(ii)	0	45.3	HMDB0246650	M+Hac-H	C10H11NO2	5.6502333333333	-	neg	236.09286114539	0.009479651133563	48.6	-
neg_7161	Saxalin	metab_38388	B(ii)	0	75.4	HMDB0302843	M+FA-H	C16H15ClO5	5.6502333333333	-	neg	367.06092724598	0.13168428837812	46.8	-
neg_7168	N1,N5,N10-Tris-Trans-P-Coumaroylspermine	metab_38395	B(ii)	0	62.1	HMDB0039962	M+Cl	C37H44N4O6	5.6502333333333	-	neg	675.29870676617	0.054423870522532	47.8	-
neg_7172	Cinncassiol A	metab_38399	B(ii)	0	42	HMDB0035164	2M-H	C20H30O7	5.6502333333333	C17645;_	neg	763.38845883405	0.14848441080243	46.4	-;73599-11-8
neg_7176	Trp-Asn-Ile	metab_38403	B(i)	93.6	0	-	M-H	C21H29N5O5	5.6555666666667	-	neg	430.20981243072	0.017470858167233	57.7	-
neg_7177	Saroglitazar	metab_38404	B(ii)	0	46.8	HMDB0257500	M+Hac-H	C25H29NO4S	5.6555666666667	-	neg	498.19714035635	0.013283663145211	46.4	-
neg_7182	3'-O-Methyl-(-)-Epicatechin-5-O-Sulphate	metab_38409	B(ii)	0	47.9	HMDB0029176	M+Hac-H	C16H16O7S	5.6609	-	neg	411.07572413623	0.067191142817155	48	-
neg_7184	6"-O-Malonyldaidzin	metab_38411	B(ii)	0	50.6	HMDB0041263	M-H	C24H22O12	5.6609	C16191	neg	501.10742576058	0.015397660966098	47.6	124590-31-4
neg_7192	Harderoporphyrinogen	metab_38419	B(ii)	0	57.8	HMDB0002160	M+Na-2H	C35H42N4O6	5.6609	-	neg	635.28554482915	0.13017896008207	50	42607-18-1
neg_7193	Dauricine	metab_38420	B(i)	79.7	0	-;HMDB0250881	M+FA-H	C38H44N2O6	5.6609	C09419;_	neg	669.32324943623	0.05430276617123	51.8	524-17-4;_
neg_7200	Ethyl Caffeate	metab_38427	B(i)	54.9	0	HMDB0301712	2M+FA-H	C11H12O4	5.6715166666667	-	neg	461.1454341	0.061329790653692	48.9	102-37-4
neg_7201	7,8-Dihydrovomifoliol 9-[Rhamnosyl-(1->6)-Glucoside]	metab_38428	B(ii)	0	51.8	HMDB0029770	M+FA-H	C25H42O12	5.6715166666667	-	neg	579.26612174172	0.10672143143538	48.6	177261-69-7
neg_7214	Nystatin	metab_38441	B(ii)	0	82.1	HMDB0014784	M-H2O-H	C47H75NO17	5.6821666666667	C06572	neg	906.48457385803	0.2497132577814	52.4	1400-61-9;
neg_7219	Petasitenine	metab_38446	B(ii)	0	57.5	HMDB0030328	2M-H	C19H27NO7	5.6875	C10359	neg	761.34828106811	0.057901704416331	47.6	60102-37-6
neg_7220	Ile-Val-Phe	metab_38447	B(i)	77.9	0	-	M-H	C20H31N3O4	5.6928333333333	-	neg	376.22445343286	0.019695176704044	53.9	-
neg_7221	Phe-Thr-Phe	metab_38448	B(i)	89.7	0	-	M-H	C22H27N3O5	5.6928333333333	-	neg	412.18787686911	0.14888339696013	54.2	-
neg_7226	5-Fluoro-3-[3-[4-(5-Methoxypyrimidin-4-Yl)Piperazin-1-Yl]Propyl]-1H-Indole	metab_38453	B(ii)	0	55.6	HMDB0249310	2M-H	C20H24FN5O	5.6928333333333	-	neg	737.38424003139	0.10612562533603	49.2	-
neg_7229	11-Hydroxy-9-Tridecenoic Acid	metab_38456	B(ii)	0	62	HMDB0035881	M+FA-H	C13H24O3	5.6981333333333	-	neg	273.17089635502	0.004120090682535	51.8	105798-56-9;
neg_7233	Linalool Oxide D 3-[Apiosyl-(1->6)-Glucoside]	metab_38460	B(ii)	0	73.1	HMDB0031367	M+FA-H	C21H36O11	5.6981333333333	-	neg	509.2241793	0.043127858677612	53.8	-

neg_7239	6-Beta-Hydroxydexamethasone	metab_38466	B(ii)	0	70.4	HMDB0061022	2M+FA-H	C22H29FO6	5.6981333333333	-	neg	861.39049481982	0.015539347332892	49.9	-
neg_7243	Mesobilirubinogen	metab_38470	B(ii)	0	54.4	HMDB0001898	M-H	C33H44N4O6	5.7034166666667	C05790	neg	591.31501592796	0.045626368602117	46.1	14684-37-8
neg_7250	Apigenin-7-Apioglucoside	metab_38477	B(i)	88.5	0	HMDB0030843	M-H	C26H28O14	5.7087666666667	C04858	neg	563.14095940884	0.047823848238872	56	26544-34-3
neg_7259	Gentiopicroside	metab_38486	B(ii)	0	57.8	HMDB0252696	2M-H	C16H20O9	5.7141	C09782;_	neg	711.21453120357	0.086751313630316	49.1	20831-76-9;_
neg_7265	Hesperetin 7-O-Rutinoside	metab_38492	B(i)	49.7	0	HMDB0003265	M-H	C28H34O15	5.7194	C09755	neg	609.18199506821	0.12677752339671	46.9	520-26-3
neg_7269	D-Pantothenoyl-L-Cysteine	metab_38496	B(ii)	0	52.1	HMDB0006834	M+Na-2H	C12H22N2O6S	5.7247333333333	C04079	neg	343.09134197365	0.087905423024711	46.5	-;_
neg_7273	Janthitrem E	metab_38500	B(ii)	0	48.8	HMDB0030529	M-H2O-H	C37H49NO6	5.7247333333333	-	neg	584.34195619239	0.008580679815555	44.3	90986-50-8
neg_7282	Glycylalanylprolylmethionylphenylalanylvalinamide	metab_38509	B(ii)	0	84.6	HMDB0033241	M+Na-2H	C29H45N7O6S	5.7301	-	neg	640.2842149	0.044723829672838	51.9	115525-99-0
neg_7284	Cyclosquamosin D	metab_38511	B(ii)	0	79.3	HMDB0303369	M-H2O-H	C41H56N8O11	5.7301	-	neg	817.38933018137	0.065891076282512	54.6	-
neg_7286	Ethyl 7-Epi-12-Hydroxyjasmonate Glucoside	metab_38513	B(ii)	0	51.3	HMDB0036340	M+Hac-H	C20H32O9	5.7354666666667	-	neg	475.22006522312	0.023061243586683	48.7	-
neg_7291	N-Docosahexaenoyl Glutamine	metab_38518	B(ii)	0	54.1	HMDB0242012	M+Na-2H	C27H40N2O4	5.7407666666667	-	neg	477.27217138788	0.059030082023404	47.8	_;
neg_7294	Sucrose 1',4'-(4,4'-Dihydroxy-3,3'-Dimethoxy-B-Truxinate)	metab_38521	B(ii)	0	78	HMDB0036978	M-H	C32H38O17	5.7407666666667	-	neg	693.20398408856	0.10119026803969	53.6	-
neg_7307	Fa(9:0)	metab_38534	B(i)	58.2	0	HMDB0000847	M+FA-H	C9H18O2	5.7514	C01601	neg	203.12900581435	0.010684253629722	50.5	112-05-0
neg_7308	Hydroxyisonobilin	metab_38535	B(ii)	0	49.6	HMDB0034475	M+Hac-H	C20H26O6	5.7514	-	neg	421.18870020554	0.025338584355304	48	-
neg_7316	Cdp-Dg(Pgd2/A-17:0)	metab_38543	B(ii)	0	68.5	HMDB0292974	M-H2O-H	C49H83N3O18P2	5.7514	-	neg	1044.4954494558	0.18362907522069	51	-
neg_7317	Capromorelin	metab_38544	B(ii)	0	64.4	HMDB0249597	M+Na-2H	C28H35N5O4	5.7567	-	neg	526.2411877	0.14842411860888	49.4	-
neg_7321	Physagulin E	metab_38548	B(ii)	0	81.4	HMDB0039693	M+Cl	C36H50O14	5.7567	-	neg	741.29358382767	0.11867040071157	51	148054-13-1
neg_7340	S-(11-Hydroxy-9-Deoxy-Delta12-Pgd2)-Glutathione	metab_38567	B(ii)	0	96.2	HMDB0013055	M+Hac-H	C30H51N3O10	5.7623166666667	-	neg	704.34695548241	0.019051169569303	55	-
neg_7343	Ps(5-Iso Pgf2Vi/14:0)	metab_38570	B(ii)	0	79.2	HMDB0280844	M+Cl	C38H68NO13P	5.7623166666667	-	neg	812.41784320392	0.037943601061562	52.4	-
neg_7349	Glu-Tyr-Phe	metab_38576	B(i)	65.5	0	-	M-H2O-H	C23H27N3O7	5.7676166666667	-	neg	438.16727584869	0.19574539556415	50.7	-
neg_7351	Isepamicine	metab_38578	B(ii)	0	75.3	HMDB0253048	M-H	C22H43N5O12	5.7676166666667	-	neg	568.27790670845	0.020769952998393	51.9	-
neg_7354	Paromomycin	metab_38581	B(ii)	0	78.8	HMDB0015490	M+Na-2H	C23H45N5O14	5.7676166666667	C00832;_	neg	636.26542420641	0.047235333895246	52.6	7542-37-2;1263-89-4;_
neg_7362	Rhoifolin	metab_38589	B(i)	90	0	HMDB0038848;HMDB0303149;HMDB0038848;HMDB0303149	M-H	C27H30O14	5.7729166666667	C12627	neg	577.15678494325	0.045584101654008	55.5	17306-46-6;_
neg_7373	Kaempferol-3-O-Rutinoside	metab_38600	B(i)	95.6	0	-	M-H	C27H30O15	5.77825	C21833	neg	593.15149381055	0.02096829719954	57.5	17650-84-9
neg_7386	(3B,16A,20R)-25-Acetoxy-3,16,20,22-Tetrahydroxy-5-Cucurbiten-11-One 3-Glucoside	metab_38613	B(ii)	0	42.5	HMDB0035346	M+Cl	C38H62O12	5.7836	-	neg	745.38966468879	0.051361835247048	45.8	178062-95-8
neg_7391	Leu-Leu-Ile	metab_38618	B(i)	88.8	0	-	M-H	C18H35N3O4	5.7889666666667	-	neg	356.2556051	0.024951864026505	56.5	-
neg_7392	Trp-Asp-Leu	metab_38619	B(i)	77.1	0	-	M-H	C21H28N4O6	5.7889666666667	-	neg	431.1937854	0.081447935821082	44.5	-
neg_7403	Cyclosquamosin E	metab_38630	B(ii)	0	90	HMDB0303370	M-H	C50H65N9O13	5.7889666666667	-	neg	998.45766423288	0.021000259090709	54.6	-
neg_7404	3-Methyldioxyindole	metab_38631	B(ii)	0	47.1	HMDB0004186	M-H2O-H	C9H9NO2	5.7943	C05834	neg	144.04562291077	0.039257869599051	48.4	-;3040-34-4
neg_7409	Trp-Tyr-Phe	metab_38636	B(i)	52	0	-	M-H	C29H30N4O5	5.7943	-	neg	513.2143498	0.026957763479875	49.2	-
neg_7410	Desethylenenorfloxacin	metab_38637	B(ii)	0	51.3	HMDB0251038	2M-H	C14H14FN3O3	5.7943	-	neg	581.20174587837	0.11796559128954	47.9	-
neg_7414	Pa(5-Iso Pgf2Vi/18:3(9Z,12Z,15Z))	metab_38641	B(ii)	0	98.4	HMDB0264245	M+Cl	C39H65O11P	5.7943	-	neg	775.39961179625	0.026146454338657	55.1	-
neg_7416	Ps(6 Keto-Pgf1Alpha/22:5(4Z,7Z,10Z,13Z,16Z))	metab_38643	B(ii)	0	76.6	HMDB0283321	M+Na-2H	C48H78NO14P	5.7943	-	neg	944.48531564008	0.011320414530081	51	-
neg_7417	2-Hydroxysebacic Acid	metab_38644	B(i)	87.4	0	HMDB0000424	M-H	C10H18O5	5.7996333333333	-	neg	217.10825178254	0.024576596693554	56.8	103963-71-9
neg_7419	Val-Phe-Leu	metab_38646	B(i)	40	0	-	M-H	C20H31N3O4	5.7996333333333	-	neg	376.22429820003	0.04962171725347	46	-
neg_7435	Deltorphin Ii	metab_38662	B(ii)	0	81.9	HMDB0250987	M-H2O-H	C38H54N8O10	5.805	C18095;_	neg	763.37823378774	0.078914329435774	53.5	122752-16-3;_
neg_7442	Monoaspartyl Chlorin E6	metab_38669	B(ii)	0	64.7	HMDB0254849	M-H	C38H41N5O9	5.8103333333333	-	neg	710.28431759543	0.037745152837226	49.9	-
neg_7460	25-Acetyl-6,7-Didehydrofevicordin F 3-Glucoside	metab_38687	B(ii)	0	48.7	HMDB0036338	M+Cl	C37H52O13	5.8156333333333	-	neg	739.30596561398	0.043141131532623	41.3	178062-92-5
neg_7470	Melledonal B	metab_38697	B(ii)	0	56.6	HMDB0037043	M+Cl	C23H27ClO8	5.8209666666667	-	neg	501.10751005886	0.032994509881057	45.3	117458-36-3
neg_7489	Vignatic Acid B	metab_38716	B(ii)	0	79.6	HMDB0033617	M-H	C27H41N3O7	5.8263	-	neg	518.28390808825	0.024672640445852	53.4	181485-20-1
neg_7518	Leucomycin A5	metab_38745	B(ii)	0	66.2	HMDB0254036	M+Cl	C39H65NO14	5.8316	C14058;_	neg	806.41327354095	0.020979266973031	51.6	18361-45-0;_
neg_7543	Panax Ginseng Tetrapeptide	metab_38770	B(ii)	0	71.2	HMDB0302146	M+FA-H	C18H33N7O7	5.8422166666667	-	neg	504.24422170022	0.084448129566574	49	-
neg_7544	Paucine	metab_38771	B(ii)	0	58.7	HMDB0029876	2M+FA-H	C13H18N2O3	5.8422166666667	C03002	neg	545.25816141542	0.034352864667247	49.3	29554-26-5

neg_7545	Fosteabine	metab_38772	B(ii)	0	74.9	HMDB0252470	M-H	C27H50N3O8P	5.8422166666667	C21596;_	neg	574.32545080828	0.006169726067047	54.5	73532-83-9;_
neg_7548	Reserpine	metab_38775	B(ii)	0	47.4	HMDB0014351	M+FA-H	C33H40N2O9	5.8422166666667	C06539	neg	653.27296689403	0.036285084501109	47.7	50-55-5
neg_7556	4,5-Dihydrovomifoliol	metab_38783	B(ii)	0	40.7	HMDB0040615	M+FA-H	C13H22O3	5.8475666666667	-	neg	271.15524415644	0.009419858853544	47.5	142173-08-8
neg_7557	4-Hydroxyvalproic Acid	metab_38784	B(ii)	0	42	HMDB0013900	2M-H	C8H16O3	5.8475666666667	C16649	neg	319.212742	0.022582651502542	46.9	60113-82-8;
neg_7561	Oleuropein	metab_38788	B(i)	95.7	0	-;HMDB0035872	M-H	C25H32O13	5.8475666666667	C09794;_	neg	539.17728083893	0.057157264260213	58.4	32619-42-4
neg_7573	Glu-Ile-Leu	metab_38800	B(i)	81.9	0	-	M-H	C17H31N3O6	5.8529166666667	-	neg	372.21410151128	0.011174045492064	55.1	-
neg_7575	11-Deoxycortisol-3-Carboxymethyloxime	metab_38802	B(ii)	0	50.2	HMDB0244352	M+Na-2H	C23H33NO6	5.8529166666667	-	neg	440.20172509338	0.071560999368218	38.1	-
neg_7590	12-Ketoporrigenin 3-[4'-(2''-Glucosyl-3''-Xylosyl)-Glucosyl)-Galactoside]	metab_38817	B(ii)	0	49.1	HMDB0038351	M-H2O-H	C50H80O24	5.8529166666667	-	neg	1045.4776905116	0.006296687648297	44	289690-76-2
neg_7593	Met Leu Phe	metab_38820	B(i)	36.3	0	-	M-H	C20H31N3O4S	5.8582666666667	-	neg	408.19284608956	0.048222663092648	43	-
neg_7616	(24R)-5B,8B-Epidioxyergosta-6,22E-Dien-3B-Ol 3-Glucoside	metab_38843	B(ii)	0	46	HMDB0037956	M+Cl	C34H54O8	5.8636	-	neg	625.35659305185	0.017987400362127	44.9	348144-81-0
neg_7634	Estradiol 17-Dihydrotrigonelline	metab_38861	B(ii)	0	42.2	HMDB0259553	M-H	C25H31NO3	5.8689333333333	-	neg	392.21927847629	0.008772049779532	44.3	-
neg_7660	Posatirelin	metab_38887	B(ii)	0	46.2	HMDB0256710	M+FA-H	C17H28N4O4	5.8743	-	neg	397.20935820766	0.003973388309214	48.9	-
neg_7666	Arctiin	metab_38893	B(i)	84.7	0	HMDB0301809;HMDB0248568	M+FA-H	C27H34O11	5.8743	C16915;_	neg	579.20865857619	0.019876316116948	56.4	20362-31-6;_
neg_7719	Asparaginyl-Proline	metab_38946	B(ii)	0	49.2	HMDB0028739	2M+FA-H	C9H15N3O4	5.8849666666667	-	neg	503.21493690674	0.050248894857951	45.8	-
neg_7733	Salinomycin	metab_38960	B(ii)	0	69.4	HMDB0257465	M+Na-2H	C42H70O11	5.8849666666667	C15690;_	neg	771.45991085884	0.1525606393663	48.3	53003-10-4;_
neg_7745	P-Menthane-3,8-Diol	metab_38972	B(ii)	0	43.8	HMDB0036145	M+FA-H	C10H20O2	5.8902666666667	C02904;_	neg	217.14466766693	0.021672587325041	48	91739-72-9;42822-86-6
neg_7749	6-Thioinosine-5'-Monophosphate	metab_38976	B(ii)	0	49.1	HMDB0060416	M+FA-H	C10H13N4O7P	5.8902666666667	C04646	neg	409.02366289529	0.066751582448731	47.7	53-83-8;_
neg_7750	Osmanthuside A	metab_38977	B(ii)	0	52.2	HMDB0038748	M-H	C23H26O9	5.8902666666667	-	neg	445.15021080198	0.009490452741259	49.8	97457-19-7
neg_7756	Tri-N-Acetylchitotriose	metab_38983	B(ii)	0	43.9	HMDB0006698	M-H	C24H41N3O16	5.8902666666667	-	neg	626.24739819856	0.064567830575746	45.4	38864-21-0
neg_7765	Acetylsalvipisone	metab_38992	B(ii)	0	62.8	HMDB0031877	2M+FA-H	C22H26O4	5.8902666666667	-	neg	753.3581991	0.0454694167899	47.9	-
neg_7767	Aclacinomycin N	metab_38994	B(ii)	0	81.3	HMDB0247946	M-H2O-H	C42H55NO15	5.8902666666667	C18637;_	neg	794.33715423509	0.032314345681344	53.1	-;_
neg_7770	Ps(6 Keto-Pgf1Alpha/22:5(7Z,10Z,13Z,16Z,19Z))	metab_38997	B(ii)	0	66.6	HMDB0283425	M+Na-2H	C48H78NO14P	5.8902666666667	-	neg	944.48530759682	0.091421256571374	49.9	-
neg_7777	Andromedoside	metab_39004	B(i)	46.5	0	-	M-H	C25H28O13	5.8956166666667	-	neg	535.14623421182	0.041722827556845	46.8	-
neg_7785	Pgp(Pgj2/I-15:0)	metab_39012	B(ii)	0	40.9	HMDB0275130	M+Cl	C41H72O15P2	5.8956166666667	-	neg	901.40356285323	0.043876857643507	46.2	-
neg_7788	Cobinamide	metab_39015	B(ii)	0	58.3	HMDB0006902	M-H	C48H72CoN11O8+2	5.8956166666667	C05774	neg	988.49072785219	0.031596660418138	49.1	-;13497-85-3
neg_7804	Matricin	metab_39031	B(ii)	0	71.9	HMDB0036643	2M+FA-H	C17H22O5	5.9009833333333	C09499	neg	657.28932798862	0.035824038657657	50.8	29041-35-8;_;
neg_7818	Traumatic Acid	metab_39045	B(i)	35.1	0	HMDB0000933;LMFA01170002	M-H	C12H20O4	5.9062833333333	C16308	neg	227.12901538942	0.021157437905468	45.7	6402-36-4;
neg_7823	Aloesol 7-Glucoside	metab_39050	B(ii)	0	53.3	HMDB0040565	M+Hac-H	C19H24O9	5.9062833333333	-	neg	455.15595960969	0.032426179639558	50.2	94356-36-2
neg_7825	Acuminoside	metab_39052	B(ii)	0	54.1	HMDB0029347	M+FA-H	C21H36O10	5.9062833333333	-	neg	493.2304228	0.063387520902579	48.7	120163-17-9
neg_7829	Alpha-Hydroxy-Tamoxifen-O-Glucuronide	metab_39056	B(ii)	0	49.2	HMDB0060808	M+Na-2H	C32H37NO8	5.9062833333333	-	neg	584.22548868768	0.1039890169149	47.4	-
neg_7839	Corynoxeine	metab_39066	B(ii)	0	50	HMDB0250480	2M+FA-H	C22H26N2O4	5.9062833333333	-	neg	809.3718576	0.013362834867266	40.5	-
neg_7843	(+/-)-Pelletierine	metab_39070	B(ii)	0	44.3	HMDB0030325	M+FA-H	C8H15NO	5.9116166666667	C06182	neg	186.11363785654	0.023341179789639	47.8	539-00-4
neg_7853	10-Acetoxyligustroside	metab_39080	B(ii)	0	60.3	HMDB0035217	M-H	C27H34O14	5.9116166666667	-	neg	581.18796061708	0.008036722563659	51.6	57799-95-8
neg_7869	5-Phenyl-1,3-Oxazinane-2,4-Dione	metab_39096	B(ii)	0	40.8	HMDB0060400	M+Cl	C10H9NO3	5.9169666666667	C16596	neg	226.02775977397	0.067899195695602	46.1	-;_
neg_7879	Mycophenolic Acid O-Acyl-Glucuronide	metab_39106	B(ii)	0	44.8	HMDB0060491	M-H2O-H	C23H28O12	5.9169666666667	C20383	neg	477.13798500427	0.056481857171954	46	-;_
neg_7885	Monoglucuronylglycyrrhetinic Acid	metab_39112	B(ii)	0	71.7	HMDB0037827	M-H	C36H54O10	5.9169666666667	-	neg	645.362109	0.007030362911948	52.2	34096-83-8
neg_7897	Ps(Pgf2Alpha/18:4(6Z,9Z,12Z,15Z))	metab_39124	B(ii)	0	81.3	HMDB0281972	M+Na-2H	C44H72NO13P	5.9169666666667	-	neg	874.4468234	0.031701453015195	54.5	-
neg_7903	2-{1-[2-(4-Benzhydrylpiperazino)-2-Oxoethyl]Cyclopentyl}Acetic Acid	metab_39130	B(i)	56.9	0	-	M+Hac-H	C26H32N2O3	5.9223	-	neg	479.25138967859	0.015065803817582	47.4	-
neg_7904	Ascorbyl Stearate	metab_39131	B(ii)	0	52.3	HMDB0038242	M+FA-H	C24H42O7	5.9223	-	neg	487.29318737714	0.011084448219584	47.4	10605-09-1;
neg_7905	Glu Thr Asp Arg	metab_39132	B(i)	57.6	0	-	M-H	C19H33N7O10	5.9223	-	neg	518.22604649306	0.028088962444399	48.5	-
neg_7909	Diethylenetriamine Crosslinked With Epichlorohydrin	metab_39136	B(ii)	0	42.3	HMDB0032216	2M+FA-H	C14H22ClNO	5.9223	-	neg	555.28072158635	0.023511508554764	41	25085-17-0
neg_7913	L-Prolinamide,D-Tyrosyl-L-Arginylglycyl-4-Nitro-L-[illegible]	metab_39140	B(ii)	0	52.9	HMDB0247954	M-H2O-H	C31H42N10O8	5.9223	-	neg	663.30572761131	0.069272660193729	46.2	-
neg_7915	Zearalanone	metab_39142	B(ii)	0	53.8	HMDB0259993	2M+FA-H	C18H24O5	5.9223	_;C14754	neg	685.32122868141	0.028653770780971	46.7	5975-78-0;_

neg_7919	Pa(6 Keto-Pgf1Alpha/I-13:0)	metab_39146	B(ii)	0	71.9	HMDB0267448	M+FA-H	C36H65O12P	5.9223	-	neg	765.41483410866	0.19940337187394	50.8	-
neg_7927	(3A,5B,7A)-23-Carboxy-7-Hydroxy-24-Norcholan-3-Yl-B-D-Glucopyranosiduronic Acid	metab_39154	B(ii)	0	40.6	HMDB0002430	M-H	C30H48O10	5.9279	-	neg	567.31519354673	0.021868839948825	44.3	58814-71-4
neg_7953	1-Acetyl-3,14,20-Trihydroxywitha-5,24-Dienolide 3-Glucoside	metab_39180	B(ii)	0	76.2	HMDB0033572	M-H	C36H54O12	5.93325	-	neg	677.35193654942	0.050468140389878	53.2	227179-07-9
neg_7968	(E)-N-(2-Amino-4-Fluorophenyl)-3-(1-Cinnamyl-1H-Pyrazol-4-Yl)Acrylamide	metab_39195	B(ii)	0	42.8	HMDB0257192	M+Hac-H	C21H19FN4O	5.9386	-	neg	421.16522381247	0.11549172319958	44.2	-
neg_7971	1-O,6-O-Bis(4-Hydroxy-Trans-Cinnamoyl)-Beta-D-	metab_39198	B(i)	66.5	0	-	M-H	C24H24O10	5.9386	-	neg	471.12976751252	0.06195904685021	50.5	-
neg_7973	Ligustroside	metab_39200	B(i)	97.6	0	HMDB0034751	M-H	C25H32O12	5.9386	-	neg	523.18218463428	0.003485306754824	59.3	35897-92-8
neg_7989	(E)-7-(4-Methoxy-5-Methyl-6-Oxopyran-2-Yl)Hept-6-Enoic	metab_39216	B(i)	59.2	0	-	M-H	C14H18O5	5.94395	-	neg	265.10603896077	0.037752625680773	48.2	-
neg_8018	Coproporphyrinogen I	metab_39245	B(ii)	0	56.6	HMDB0002158	M-H	C36H44N4O8	5.9492333333333	C05768	neg	659.30413310191	0.11405223488913	47.1	-;31110-56-2
neg_8022	Azaspiracid	metab_39249	B(ii)	0	79.5	HMDB0033805	M+Cl	C47H71NO12	5.9492333333333	C16907;_	neg	876.47334434615	0.068470114782748	51.9	214899-21-5
neg_8024	Azelaic Acid	metab_39251	B(i)	98.6	0	HMDB0000784;LMFA01170054	M-H	C9H16O4	5.9545833333333	C08261	neg	187.09763022885	0.008688413930394	59.4	123-99-9;
neg_8030	(5E,7E)-Undeca-2,5,7-Trienedioylcarnitine	metab_39257	B(ii)	0	64.7	HMDB0241202	M-H	C18H27NO6	5.9545833333333	-	neg	352.17663330481	0.017621864643144	51.8	-
neg_8050	Vinbarbital	metab_39277	B(i)	52.6	0	HMDB0259808	M-H	C11H16N2O3	5.9599166666667	-	neg	223.10891688213	0.062890718190268	48.7	-
neg_8051	L,L-Cyclo(Leucylprolyl)	metab_39278	B(ii)	0	60.7	HMDB0034276	M+Hac-H	C11H18N2O2	5.9599166666667	-	neg	269.15077369817	0.007745927882340	51.9	2873-36-1
neg_8052	10-(2,3-Dihydroxypropoxy)-10-Oxodecanoic Acid	metab_39279	B(ii)	0	42.6	HMDB0257660	M+Na-2H	C13H24O6	5.9599166666667	-	neg	297.13216623781	0.063397526258037	46.5	-
neg_8054	6-Hydroxyemedastine	metab_39281	B(ii)	0	43.9	HMDB0060785	M-H	C17H26N4O2	5.9599166666667	-	neg	317.19710067128	0.004705115742920	46.9	-
neg_8060	Dexniguldipine	metab_39287	B(ii)	0	59.1	HMDB0251090	M-H	C36H39N3O6	5.9599166666667	C11236	neg	608.27319541957	0.029988821598083	48.3	102993-22-6;_
neg_8085	Phe-Glu-Leu	metab_39312	B(i)	53.9	0	-	M-H2O-H	C20H29N3O6	5.9706333333333	-	neg	388.1879381	0.020171762403431	49.9	-
neg_8086	Semagacestat	metab_39313	B(ii)	0	56.3	HMDB0258218	M+Hac-H	C19H27N3O4	5.9706333333333	-	neg	420.2144696	0.035944452013464	50.1	-
neg_8088	Minocycline	metab_39315	B(ii)	0	63.6	HMDB0015152	M-H	C23H27N3O7	5.9706333333333	C07225	neg	456.17528722479	0.085309102473433	48.2	10118-90-8;
neg_8093	Dihydrocytochalasin B	metab_39320	B(ii)	0	45.1	HMDB0251302	M+FA-H	C29H39NO5	5.9706333333333	-	neg	526.2785098	0.095824146613981	45.7	-
neg_8103	Dihydrofoliamenthic Acid	metab_39330	B(i)	57	0	-	M+FA-H	C10H18O3	5.9759166666667	-	neg	231.12391001459	0.025513838384355	50.5	-
neg_8106	Tetranorprostanedioic Acid	metab_39333	B(ii)	0	70	HMDB0258926	M+Hac-H	C16H28O4	5.9759166666667	-	neg	343.21270736186	0.004694002313559	53.5	-
neg_8116	Caffeine, 8-((3-Methoxypropyl)Amino)-	metab_39343	B(ii)	0	68.4	HMDB0247409	2M+FA-H	C12H19N5O3	5.9759166666667	-	neg	607.29847042551	0.071186767184915	50.5	-
neg_8122	Medicoside H	metab_39349	B(ii)	0	88.1	HMDB0039291	M+Cl	C47H74O19	5.9759166666667	-	neg	977.44444340653	0.028218497529786	53.7	125448-43-3
neg_8141	6-Ketoprostaglandin F1Alpha	metab_39368	B(i)	45.3	0	HMDB0002886	M+FA-H	C20H34O6	5.9865666666667	C05961	neg	415.23189145262	0.023113765575335	46.4	58962-34-8
neg_8147	Leu-Arg-Asn-Arg	metab_39374	B(ii)	0	73.8	HMDB0304803	M-H	C22H43N11O6	5.9865666666667	-	neg	556.33527043251	0.066898972716407	52.6	-
neg_8151	5-Azanonane Deriv.	metab_39378	B(ii)	0	58.7	HMDB0249975	M+Cl	C29H49N5O7	5.9865666666667	-	neg	614.33143986659	0.05305315006886	49	-
neg_8155	Hexaminolevulinate	metab_39382	B(ii)	0	44.2	HMDB0253144	M-H	C11H21NO3	5.9918666666667	-	neg	214.14500262752	0.040711953159795	47.4	-
neg_8158	Alpha-Hydroxy-N-Desmethyltamoxifen	metab_39385	B(ii)	0	72.1	HMDB0060668	M+FA-H	C25H27NO2	5.9918666666667	C16549	neg	418.19892508011	0.054585766132385	49.5	-;_
neg_8163	Cdp-Dg(5-Iso Pgf2Vi/I-12:0)	metab_39390	B(ii)	0	58.1	HMDB0293304	M+Na-2H	C42H71N3O18P2	5.9918666666667	-	neg	988.40321743459	0.12962229601698	48.5	-
neg_8166	(+)-Dehydrovomifoliol	metab_39393	B(ii)	0	65.7	HMDB0302822	M+FA-H	C13H18O3	5.9971666666667	C02533	neg	267.12361895111	0.013350384970715	52.6	-
neg_8169	N'-Hydroxyneosaxitoxin	metab_39396	B(ii)	0	54.1	HMDB0033665	M+Na-2H	C10H17N7O6	5.9971666666667	-	neg	352.10146404611	0.091074383173085	47.9	153856-78-1
neg_8178	Methylisopelletierine	metab_39405	B(ii)	0	55	HMDB0030326	M+FA-H	C9H17NO	6.0024833333333	C06184	neg	200.1293504	0.012159319434473	50.1	-
neg_8183	9E-Heptadecenoic Acid	metab_39410	B(ii)	0	44.3	HMDB0031046	M+Cl	C17H32O2	6.0024833333333	C16536	neg	303.20808575492	0.087150631096689	44.1	-;76261-97-7;
neg_8195	Amikacin	metab_39422	B(ii)	0	89.1	HMDB0014622	M+Na-2H	C22H43N5O13	6.0024833333333	C06820;_	neg	606.25703486565	0.24148950343548	55	37517-28-5;_
neg_8225	4-Ethyloctanoic Acid	metab_39452	B(i)	37.3	0	HMDB0036136;LMFA01030983	M+Hac-H	C10H20O2	6.0184833333333	-	neg	231.16026556688	0.024543790189854	47.1	16493-80-4;
neg_8227	Luteolin	metab_39454	B(i)	84.8	0	HMDB0005800;LMPK12110006	M-H	C15H10O6	6.0184833333333	C01514	neg	285.04055114562	0.051311684735425	55.8	491-70-3;
neg_8230	Arg-Ser-Pro	metab_39457	B(i)	84.1	0	-	M+Hac-H	C14H26N6O5	6.0184833333333	-	neg	417.21320871647	0.034838398127448	54.5	-
neg_8254	Hexadecanedioic Acid	metab_39481	B(ii)	0	69.1	HMDB0000672	M+Hac-H	C16H30O4	6.0291166666667	C19615;C08260	neg	345.22823245523	0.006966149191287	53.1	-;505-54-4;
neg_8272	1-(2,4,6-Trimethylphenoxy)Propan-2-Amine	metab_39499	B(ii)	0	61.4	HMDB0246512	M+FA-H	C12H19NO	6.0344166666667	-	neg	238.14496135654	0.092607068402262	50.2	-
neg_8275	3-Hydroxy-5-Methoxy-2,2-Dimethyl-7-Pentyl-3,4-Dihydrochromene-8-Carboxylic Acid	metab_39502	B(i)	50.5	0	-	M+FA-H	C18H26O5	6.0344166666667	-	neg	367.17404151059	0.056098026459661	46.7	-
neg_8277	Butyl (Z)-5,8,9-Trihydroxy-4-[(E)-2-Methylbut-2-Enoyl]Oxydec-6-Enoate	metab_39504	B(i)	56	0	-	M+Cl	C19H32O7	6.0344166666667	-	neg	407.18502043267	0.086290711126376	48.5	-
neg_8289	(Z)-Dodec-5-Enedioic Acid	metab_39516	B(i)	77.1	0	-	M-H	C12H20O4	6.0397166666667	-	neg	227.12897319068	0.026523636615051	53.8	-

neg_8311	1-(4-Hydroxy-1,3-Dihydro-2-Benzofuran-1-Yl)Octane-2,3,7-	metab_39538	B(i)	40.6	0	-	M+FA-H	C16H24O5	6.0450666666667	-	neg	341.1583058	0.015743132933271	45.4	-
neg_8315	2,3-Dihydroxypropyl (9Z,14Z)-12,13,16-Trihydroxyoctadeca-9,14-Dienoate	metab_39542	B(i)	74.9	0	-	M+FA-H	C21H38O7	6.0450666666667	-	neg	447.26005316073	0.077084723601302	51.2	-
neg_8325	Bisdethiobis(Methylthio)Gliotoxin	metab_39552	B(ii)	0	68.7	HMDB0249258	M+FA-H	C15H20N2O4S	6.05035	-	neg	401.08798397067	0.018274091689614	49.6	-
neg_8327	Isoorientin	metab_39554	B(ii)	0	47.9	HMDB0253666	M+Na-2H	C21H20O11	6.05035	_;C01821	neg	469.07526283969	0.017009730733644	47.2	4261-42-1;_;
neg_8337	P-Hpea-Eda	metab_39564	B(ii)	0	54.5	HMDB0029305	M+Hac-H	C17H20O5	6.0557	-	neg	363.14551965152	0.15054005900688	49.2	-
neg_8347	3-Hydroxyoctanoic Acid	metab_39574	B(i)	89.2	0	-;HMDB0001954	M-H	C8H16O3	6.0609833333333	C20793;_	neg	159.1027709	0.013430102540098	57.5	14292-27-4;33796-86-0
neg_8349	Parasorbic Acid	metab_39576	B(ii)	0	44	HMDB0301801	2M-H	C6H8O2	6.0609833333333	C08502;_	neg	223.09717634107	0.02218216085094	48.2	10048-32-5
neg_8357	Dodecyl-Beta-D-Maltoside	metab_39584	B(ii)	0	76.1	HMDB0247203	M-H2O-H	C24H46O11	6.0609833333333	-	neg	491.28640317023	0.011701273096583	54	-
neg_8361	Vpgpr Enterostatin	metab_39588	B(ii)	0	71.5	HMDB0003577	M+Cl	C23H40N8O6	6.0609833333333	-	neg	559.27378273593	0.076267190089713	51.5	144964-56-7
neg_8376	Psoromic Acid	metab_39603	B(i)	54.1	0	-	M-H	C18H14O8	6.0716833333333	-	neg	357.06160905129	0.020438465308857	48.8	-
neg_8380	Apigenin	metab_39607	B(i)	71.4	0	HMDB0002124;LMPK12110005	M-H	C15H10O5	6.0770333333333	C01477	neg	269.04569236128	0.014306636037593	53.4	520-36-5;
neg_8388	(3R)-8-Hydroxy-3-(4-Methoxyphenyl)-3,4-Dihydroisochromen-	metab_39615	B(i)	68	0	-	M+FA-H	C16H14O4	6.0823166666667	-	neg	315.08512543694	0.1453479451263	50	-
neg_8392	Isopetasoside	metab_39619	B(ii)	0	74.4	HMDB0029622	M+Cl	C21H32O7	6.0823166666667	-	neg	431.18213493427	0.11964269641756	51.7	69809-29-6
neg_8400	Phenylpyruvic Acid	metab_39627	B(i)	73.4	0	HMDB0000205;PW_C000131	M-H	C9H8O3	6.08765	C00166	neg	163.04018028372	0.052054216163913	53.6	156-06-9
neg_8420	Ibuprofen Metabolite A	metab_39647	B(i)	79.4	0	-	M-H2O-H	C13H18O3	6.0985333333333	-	neg	203.10784573567	0.016217067328541	55.3	-
neg_8421	4'-O-Methylhydrangenol	metab_39648	B(i)	37.2	0	-	M-H	C16H14O4	6.0985333333333	-	neg	269.08091942336	0.031229348964541	45.1	-
neg_8430	16-Hydroxy-9-Oxooctadeca-10,12,14-Trienoic Acid	metab_39657	B(i)	51.8	0	-	M-H	C18H28O4	6.1038333333333	-	neg	307.19149395696	0.028960641110262	48.9	-
neg_8433	Prostaglandin D3	metab_39660	B(i)	51.7	0	HMDB0003034	M+FA-H	C20H30O5	6.1038333333333	C13802	neg	395.2055091	0.007964626812898	47.5	71902-47-1
neg_8438	Cabergoline	metab_39665	B(ii)	0	47.2	HMDB0014393	M+Na-2H	C26H37N5O2	6.1038333333333	C08187	neg	472.26648443793	0.12919631252613	47	81409-90-7
neg_8441	Ethyl P-Coumarate	metab_39668	B(i)	74.7	0	-	M-H	C11H12O3	6.1091833333333	-	neg	191.07144143685	0.037084346399807	53.7	-
neg_8442	Undecanedioic Acid	metab_39669	B(i)	96.4	0	HMDB0000888;LMFA01170007	M-H	C11H20O4	6.1091833333333	-	neg	215.12896399799	0.009693893237209	58.2	1852-04-6;
neg_8460	2,3,5,15-Tetrahydroxyhexadecanoic Acid	metab_39687	B(i)	41.7	0	-	M-H2O-H	C16H32O6	6.1145166666667	-	neg	301.20209644071	0.017142466104974	47.6	-
neg_8461	3-Acetamido-5-Ethyladamantane-1-Carboxylic Acid	metab_39688	B(i)	73.9	0	-	M+FA-H	C15H23NO3	6.1145166666667	-	neg	310.16389277971	0.068118793787276	49.8	-
neg_8474	(Z)-6-Methyl-2-(4-Methylpent-3-Enyl)Hept-2-Enedioic Acid	metab_39701	B(i)	79.3	0	-	M-H	C14H22O4	6.1198666666667	-	neg	253.14459695643	0.014787429745567	55.1	-
neg_8483	Capryloylglycine	metab_39710	B(i)	90.1	0	HMDB0000832	M-H	C10H19NO3	6.1252	-	neg	200.12933180494	0.047229122337264	56.7	14246-53-8
neg_8492	2-Hydroxycaprylic Acid	metab_39719	B(i)	91	0	HMDB0000711	M-H	C8H16O3	6.1305	-	neg	159.10272229565	0.013704241900379	57.7	617-73-2
neg_8495	4-(1,7-Dihydroxyoctyl)-2-Hydroxy-3-Methyl-2H-Furan-5-One	metab_39722	B(i)	84.7	0	-	M-H2O-H	C13H22O5	6.1305	-	neg	239.12897078532	0.047898880652481	56.1	-
neg_8496	Blumealactone C	metab_39723	B(ii)	0	53.9	HMDB0035357	M-H	C17H24O6	6.1305	-	neg	323.14783613319	0.015245183600584	47.8	111545-48-3
neg_8515	5,8,11-Trihydroxyoctadec-9-Enoic Acid	metab_39742	B(i)	81.5	0	-	M-H	C18H34O5	6.14115	-	neg	329.23336037072	0.006688961516024	55.7	-
neg_8522	22-Angeloylbarringtogenol C	metab_39749	B(ii)	0	42.3	HMDB0032835	M+Cl	C35H56O6	6.14115	-	neg	607.38268438211	0.040611288708359	44.2	200492-43-9
neg_8524	Dg(Pge2/I-13:0/0:0)	metab_39751	B(ii)	0	83.3	HMDB0298566	M+Hac-H	C36H62O8	6.14115	-	neg	681.45612748266	0.019715902370943	55.3	-
neg_8536	3-Chlorobenzoic Acid	metab_39763	B(i)	99.4	0	HMDB0001544	M-H	C7H5ClO2	6.1517333333333	-	neg	154.99065913797	0.010259760181079	54.6	535-80-8
neg_8537	3-Methylsuberic Acid	metab_39764	B(ii)	0	45.5	HMDB0059783	M-H2O-H	C9H16O4	6.1517333333333	-	neg	169.08711144274	0.29079266321123	46.9	_;
neg_8548	2-(2-Carboxy-2-Methylpropyl)-4,6-Dimethylbenzoic Acid	metab_39775	B(i)	80.4	0	-	M-H	C14H18O4	6.1570333333333	-	neg	249.11326524567	0.047775666718944	54.8	-
neg_8555	2-Octynoic Acid	metab_39782	B(ii)	0	41.4	HMDB0245278	2M+FA-H	C8H12O2	6.1623666666667	-	neg	325.16348358529	0.043982441194955	45.9	_;
neg_8558	10,11-Dihydro-20-Trihydroxy-Leukotriene B4	metab_39785	B(ii)	0	44	HMDB0012503	M+Hac-H	C20H34O7	6.1623666666667	-	neg	445.24427093968	0.11057361682541	45.5	_;
neg_8564	2-{2-Oxo-2-[(2-Oxo-3-Azepanyl)Amino]Ethoxy}Acetic Acid	metab_39791	B(i)	70.9	0	-	M+FA-H	C10H16N2O5	6.1676833333333	-	neg	289.10579763838	0.21060701494099	51	-
neg_8580	9,10,13-Trihydroxystearic Acid	metab_39807	B(ii)	0	67.4	HMDB0030935	M-H	C18H36O5	6.173	-	neg	331.24875161611	0.059746349829111	52.4	50439-74-2
neg_8589	Pyropheophorbide A	metab_39816	B(ii)	0	46.5	HMDB0256993	M-H	C33H34N4O3	6.173	C18064;_	neg	533.26025873681	0.08080912579889	44.4	-;_
neg_8599	Ent-8-Iso Prostaglandin F2Alpha	metab_39826	B(i)	67.5	0	-	M+FA-H	C20H34O5	6.1782833333333	-	neg	399.23773784148	0.060291610747352	49.7	-
neg_8617	Hyocholic Acid	metab_39844	B(i)	54.9	0	-;HMDB0000760;LMST04010064	M-H	C24H40O5	6.1889666666667	C17649;_	neg	407.28026182984	0.026357481000788	48.9	547-75-1;
neg_8631	Vanillylacetone	metab_39858	B(i)	90.9	0	HMDB0032590	M-H	C11H14O3	6.1996166666667	C17497	neg	193.08708611896	0.050991383232573	57.4	122-48-5
neg_8632	3-[[5-Methyl-2-(1-Methylethyl)Cyclohexyl]Oxy]-1,2-Propanediol	metab_39859	B(ii)	0	60	HMDB0036133	M+FA-H	C13H26O3	6.1996166666667	-	neg	275.186503	0.035837915187013	51.3	87061-04-9
neg_8633	(+/-)-3-Hydroxynonanoic Acid	metab_39860	B(ii)	0	64.7	HMDB0031513	2M-H	C9H18O3	6.1996166666667	-	neg	347.2442055	0.034134428080593	51.8	88930-09-0

neg_8635	Dihydroceramide	metab_39862	B(ii)	0	53	HMDB0006752	M+Hac-H	C19H39NO3	6.1996166666667	C12126	neg	388.30701434689	0.089360559333046	49.3	-;_
neg_8636	Fluticasone 17Beta-Carboxylic Acid	metab_39863	B(ii)	0	54.6	HMDB0061092	M+Na-2H	C21H26F2O5	6.1996166666667	-	neg	417.15094236941	0.060777976573722	47.1	-
neg_8638	Zaldaride	metab_39865	B(ii)	0	51.2	HMDB0259982	M+Hac-H	C26H28N4O2	6.1996166666667	-	neg	487.237135	0.012835094175402	48.3	-
neg_8653	Dg(Pgj2/I-12:0/0:0)	metab_39880	B(ii)	0	49.5	HMDB0298375	M+FA-H	C35H58O7	6.2049166666667	-	neg	635.41407775251	0.058169549439685	46.8	-
neg_8666	Muricin E	metab_39893	B(ii)	0	59.8	HMDB0036974	M+FA-H	C33H60O7	6.2102166666667	-	neg	613.43199600402	0.045405659465768	50.1	-
neg_8673	Capsidiol	metab_39900	B(ii)	0	59.1	HMDB0002352	M+FA-H	C15H24O2	6.21555	C09627	neg	281.17588902298	0.015187559004226	51.4	37208-05-2;
neg_8674	10Z-Nonadecenoic Acid	metab_39901	B(ii)	0	55.8	HMDB0013622	M+Cl	C19H36O2	6.21555	-	neg	331.23945979251	0.062026549995969	47.3	73033-09-7;
neg_8686	3-Hydroxymyristic Acid	metab_39913	B(i)	54.2	0	HMDB0061656	M+Hac-H	C14H28O3	6.22085	-	neg	303.21775885656	0.017390400401064	50.3	35683-15-9
neg_8694	3-Hydroxydecanoic Acid	metab_39921	B(i)	56.6	0	HMDB0002203	M-H	C10H20O3	6.2261666666667	-	neg	187.13404987481	0.024008084006685	50.7	19526-23-9
neg_8703	Dg(Pge1/I-22:0/0:0)	metab_39930	B(ii)	0	53.6	HMDB0300571	M-H2O-H	C45H82O8	6.2261666666667	-	neg	731.587146	0.061952902672971	46.1	-
neg_8712	10Z-Pentadecenoic Acid	metab_39939	B(ii)	0	62.5	HMDB0304816	M+FA-H	C15H28O2	6.2368	-	neg	285.20717213682	0.005987753125226	52.1	_;
neg_8716	Methyl (Z)-9,10,11-Trihydroxyoctadec-12-Enoate	metab_39943	B(i)	73.6	0	-	M+Cl	C19H36O5	6.2368	-	neg	379.22525036239	0.055903868458914	51.5	-
neg_8741	Cincassiol B	metab_39968	B(ii)	0	41.5	HMDB0036855	M+Na-2H	C20H32O8	6.2474333333333	-	neg	421.18229634691	0.020236322009613	45.2	73599-13-0
neg_8749	Tridecanoic Acid	metab_39976	B(i)	44.3	0	HMDB0000910;LMFA01010013	M+FA-H	C13H26O2	6.2527833333333	C17076	neg	259.19159369843	0.017684889479766	48.2	638-53-9;
neg_8751	Enoximone Sulfoxide	metab_39978	B(ii)	0	45.2	HMDB0247510	M+Cl	C12H12N2O3S	6.2527833333333	-	neg	299.02654938349	0.19236187965676	47.8	-
neg_8757	Arg-Trp-Arg	metab_39984	B(i)	84.9	0	-	M+FA-H	C23H36N10O4	6.2527833333333	-	neg	561.2916985	0.016496391194793	54.7	-
neg_8766	Thr-Leu-Lys	metab_39993	B(i)	42	0	-	M-H	C16H32N4O5	6.2583333333333	-	neg	359.22669101234	0.20413632308634	44.1	-
neg_8786	16-Hydroxyhexadecanoic Acid	metab_40013	B(i)	51.1	0	HMDB0006294	M+Hac-H	C16H32O3	6.2689833333333	C18218	neg	331.2490778	0.004903452273433	49.6	506-13-8
neg_8798	16-(1-Carboxyethoxy)-16-Oxohexadecanoic Acid	metab_40025	B(i)	42.2	0	-	M+FA-H	C19H34O6	6.2743333333333	-	neg	403.23385218925	0.01408477568995	48.1	-
neg_8806	(2S,3R,4R)-2-Amino-3,4-Dihydroxy-2-(Hydroxymethyl)-14-Oxoicos-6-Enoic Acid	metab_40033	B(ii)	0	42.6	HMDB0242536	M-H2O-H	C21H39NO6	6.2796833333333	-	neg	382.25999538291	0.15976280002198	45.4	-
neg_8820	1B,3A,7A-Trihydroxy-5B-Cholanoic Acid	metab_40047	B(i)	59.2	0	HMDB0000414	M-H	C24H40O5	6.28505	-	neg	407.28045771326	0.013216410675543	50.6	99598-04-6
neg_8823	Cholic Acid	metab_40050	B(i)	96.6	0	HMDB0000619;LMST04010001;PW_C000480	M+FA-H	C24H40O5	6.28505	C00695	neg	453.28565925354	0.027359313729371	58.9	81-25-4;
neg_8828	1-(6-((3-Methoxyestra-1,3,5(10)-Trien-17-Yl)Amino)Hexyl)-1H-Pyrrole-2,5-Dione	metab_40055	B(ii)	0	61.1	HMDB0244394	M+Hac-H	C29H40N2O3	6.28505	-	neg	523.319456	0.15491654755152	49.3	-
neg_8833	Muricin A	metab_40060	B(ii)	0	64.4	HMDB0036977	M+FA-H	C35H64O7	6.28505	-	neg	641.46341210548	0.04486745268407	50.6	-
neg_8871	Fa(18:1+2O)	metab_40098	B(i)	90.3	0	-	M-H	C18H34O4	6.3063333333333	-	neg	313.23845874438	0.017240591127711	57.4	-
neg_8876	(3Beta,6Alpha,19Alpha)-3,6,19-Trihydroxy-12-Ursen-28-Oic	metab_40103	B(i)	81.9	0	HMDB0036961	M-H	C30H48O5	6.3063333333333	-	neg	487.34307522858	0.024894993141186	55.8	130289-31-5
neg_8878	3-Hydroxy-5,5,8A-Trimethyl-3,4,4A,6,7,8-Hexahydronaphthalene-2-Carboxylic Acid	metab_40105	B(i)	64.3	0	-	M-H	C14H22O3	6.3116833333333	-	neg	237.14977327887	0.043876998768281	51.2	-
neg_8879	Farnesol	metab_40106	B(ii)	0	55.1	HMDB0004305	M+FA-H	C15H26O	6.3116833333333	C01493;C06081;	neg	267.19667464491	0.040663766915126	50.2	4602-84-0;_
neg_8888	Dg(Pge2/A-15:0/0:0)	metab_40115	B(ii)	0	71.5	HMDB0297528	M-H	C38H66O8	6.3116833333333	-	neg	649.46607359343	0.06315921882583	51.6	-
neg_8895	N-Lauroyl Cysteine	metab_40122	B(ii)	0	43.7	HMDB0242030	M-H	C15H29NO3S	6.3169833333333	-	neg	302.17959011797	0.070811611237412	46.9	-
neg_8901	Lnape(3:0/N-15:1)	metab_40128	B(i)	44	0	-	M-H	C23H44NO8P	6.3169833333333	-	neg	492.27309487462	0.023911750121214	47.1	-
neg_8909	(9Z)-Heptadeca-1,9-Dien-4,6-Diyne-3,8,11-Triol	metab_40136	B(i)	35.8	0	-	M-H	C17H24O3	6.3223333333333	-	neg	275.16536000691	0.007804930115703	44.6	-
neg_8911	Coronaric Acid	metab_40138	B(i)	77.1	0	HMDB0004701	M+Hac-H	C18H32O3	6.3223333333333	C14825	neg	355.24912498632	0.013754882203959	53.9	-
neg_8934	Dgmg(18:3)	metab_40161	B(i)	82.9	0	-	M+FA-H	C33H56O14	6.3382833333333	-	neg	721.3655014	0.061761527251873	54.7	-
neg_8944	Dinoterb	metab_40171	B(i)	63.7	0	-	M-H	C10H12N2O5	6.3489833333333	C18671	neg	239.06742210198	0.037507085616043	51.4	1420-07-1
neg_8951	Schinalactone C	metab_40178	B(i)	52.9	0	-	M-H	C30H46O5	6.3489833333333	-	neg	485.3278301	0.068621128150005	48.8	-
neg_8985	Enalaprilat	metab_40212	B(ii)	0	62	HMDB0041886	M+Na-2H	C18H24N2O5	6.3702333333333	C11720	neg	369.14508673397	0.075025042908752	49	76420-72-9
neg_8986	8,9-Dhet	metab_40213	B(i)	45.8	0	HMDB0002311	M+FA-H	C20H34O4	6.3702333333333	C14773	neg	383.24182209516	0.003397841265793	46.8	-
neg_9012	Dihydrocortisol	metab_40239	B(ii)	0	42.3	HMDB0003259	M-H	C21H32O5	6.3861666666667	C05471	neg	363.21530046143	0.01422680020713	45.8	1482-50-4;
neg_9029	Mgmg(18:3)	metab_40256	B(i)	44.7	0	-	M+FA-H	C27H46O9	6.3914666666667	-	neg	559.31220645344	0.019363203611414	47.9	-
neg_9030	Amphibine H	metab_40257	B(ii)	0	58	HMDB0029333	M-H2O-H	C33H43N5O6	6.3914666666667	-	neg	586.29982109063	0.084073623268091	45.3	52659-55-9
neg_9038	Lpc(14:0)	metab_40265	B(i)	93.4	0	HMDB0010379	M+FA-H	C22H46NO7P	6.3967666666667	-	neg	512.29994338979	0.025033876982918	58	20559-16-4
neg_9050	3-(3,5-Di-Tert-Butyl-4-Hydroxyphenyl)Propionic Acid	metab_40277	B(ii)	0	56.6	HMDB0245577	M-H	C17H26O3	6.4074	-	neg	277.18096992934	0.041490825318476	48.9	-
neg_9051	Deoxycholic Acid	metab_40278	B(i)	86.7	0	HMDB0000626;LMST04010040;PW_C000487	M-H	C24H40O4	6.4074	C04483	neg	391.28548175574	0.020983530692413	56.3	83-44-3;

neg_9063	3-Epiarmefolin	metab_40290	B(ii)	0	42.8	HMDB0036135	2M-H	C15H20O4	6.41275	-	neg	527.26162643474	0.063852478584791	46	293313-26-5
neg_9083	Hexosyl Lpe(18:2)	metab_40310	B(i)	43.8	0	-	M-H	C29H54NO12P	6.4289833333333	-	neg	638.3310474	0.048054084301672	48	-
neg_9088	Ethisterone	metab_40315	B(i)	65.3	0	HMDB0060580	M-H	C21H28O2	6.4343333333333	C14487	neg	311.20169750021	0.012563687724625	52.3	434-03-7;_
neg_9089	Cannabidiolic Acid	metab_40316	B(i)	41.4	0	-;HMDB0249587;LMPK13120003	M+Na-2H	C22H30O4	6.4343333333333	C10784;_	neg	379.18933798766	0.077382707892741	45.6	1244-58-2;_;
neg_9090	Bufadienolide	metab_40317	B(ii)	0	68.4	HMDB0249427	M+FA-H	C24H34O2	6.4343333333333	_;C16921	neg	399.25406282805	0.033347915037842	51.9	29565-35-3;_
neg_9093	Asimilobin	metab_40320	B(ii)	0	40.4	HMDB0029960	M-H	C35H62O6	6.4343333333333	-	neg	577.44761981171	0.037359054584879	46.3	168075-15-8
neg_9100	Mgdg(O-8:0/6:0)	metab_40327	B(i)	51.5	0	-	M+FA-H	C23H44O9	6.4396333333333	-	neg	509.29668715027	0.035009404208909	48.8	-
neg_9101	Pc(Pgf1Alpha/P-16:0)	metab_40328	B(ii)	0	88.2	HMDB0289522	M-H	C44H84NO10P	6.4396333333333	-	neg	816.5761915	0.11848055380963	54.8	-
neg_9103	Cis-9,10-Epoxystearic Acid	metab_40330	B(i)	58.1	0	HMDB0247617	M-H	C18H34O3	6.4449333333333	C19418	neg	297.24354310411	0.008176248068606	51	-
neg_9105	Ricinoleic Acid	metab_40332	B(i)	82.5	0	HMDB0034297;LMFA02000184	M+FA-H	C18H33NaO3	6.4449333333333	C08365	neg	365.23105558431	0.01120912616447	55.8	141-22-0;
neg_9108	Dihydrocelastryl Diacetate	metab_40335	B(i)	53.9	0	-	M-H	C33H44O6	6.4449333333333	-	neg	535.31259485442	0.17147938787948	41	-
neg_9111	9-Hydroxynonanoic Acid	metab_40338	B(i)	65	0	-	M-H	C9H18O3	6.4502333333333	-	neg	173.11842563985	0.006831837540025	51	-
neg_9116	Asebotoxin	metab_40343	B(ii)	0	41.6	HMDB0248654	M+Na-2H	C22H36O7	6.4502333333333	-	neg	433.21860097241	0.017247061810305	45.5	-
neg_9121	Cholylglycyltyrosine	metab_40348	B(ii)	0	49.9	HMDB0250147	M+Na-2H	C35H52N2O8	6.4502333333333	-	neg	649.34334049448	0.060610591181078	45.4	-
neg_9126	Lpe(18:2)	metab_40353	B(i)	97.7	0	HMDB0011477	M-H	C23H44NO7P	6.4555833333333	-	neg	476.27848419628	0.016434827721587	59.1	-
neg_9132	2-Hydroxymyristic Acid	metab_40359	B(i)	87.6	0	HMDB0002261;LMFA01050484	M-H	C14H28O3	6.4609166666667	-	neg	243.1966429	0.039592906577504	56.7	2507-55-3;
neg_9133	Butofilolol	metab_40360	B(ii)	0	48.4	HMDB0249467	M+FA-H	C17H26FNO3	6.4609166666667	-	neg	356.18678504544	0.008453803736541	48	-
neg_9136	Haloperidol Decanoate	metab_40363	B(ii)	0	53.6	HMDB0253043	M+Hac-H	C31H41ClFNO	6.4609166666667	-	neg	588.29207127236	0.074882762814836	41.9	-
neg_9139	15-Hede	metab_40366	B(i)	90.3	0	-	M-H	C20H36O3	6.4662666666667	-	neg	323.25934513113	0.10102979753868	55.7	-
neg_9152	2-Hydroxy-6-Pentadeca-8,11,14-Trienylbenzoic Acid	metab_40379	B(i)	78.7	0	-	M-H	C22H30O3	6.4769166666667	-	neg	341.21183883057	0.090886024268513	52.8	-
neg_9153	Lpe(15:0)	metab_40380	B(i)	79.7	0	HMDB0011472	M-H	C20H42NO7P	6.4769166666667	-	neg	438.26272372447	0.058603092252502	53.4	-
neg_9163	(+/-)11(12)-Eet Ethanolamide	metab_40390	B(ii)	0	48.6	HMDB0013652	M+FA-H	C22H37NO3	6.4822333333333	-	neg	408.2759893	0.086360153006132	47.3	-
neg_9187	Ajulemic Acid	metab_40414	B(ii)	0	63.2	HMDB0248075	M-H	C25H36O4	6.5035	-	neg	399.2543257	0.051046693277981	50.4	-
neg_9188	16,16-Dimethyl Prostaglandin E1	metab_40415	B(ii)	0	43.8	HMDB0244726	M+Hac-H	C22H38O5	6.5035	-	neg	441.28573232375	0.037317207636195	47.7	-
neg_9191	Dgdg(O-13:1/5:0)	metab_40418	B(i)	53.9	0	-	M+FA-H	C33H60O14	6.5035	-	neg	725.39648759801	0.049108303429224	49.5	-
neg_9193	N-Linoleoyl Serine	metab_40420	B(ii)	0	65	HMDB0241984	M-H	C21H37NO4	6.5088	-	neg	366.26498232197	0.051297600439928	48.7	-
neg_9205	3-Hydroxy-2-(9-Oxodecyl)Pentanedioic Acid	metab_40432	B(i)	88.9	0	-	M+FA-H	C15H26O6	6.5193833333333	-	neg	347.17161183905	0.021124768549753	56.9	-
neg_9212	Ginsenoside Rh6	metab_40439	B(ii)	0	78.9	HMDB0039436	M-H2O-H	C36H62O11	6.5246833333333	-	neg	651.41150272237	0.13704050818843	54.8	343780-67-6
neg_9225	25-Cinnamoyl-Vulgaroside	metab_40452	B(ii)	0	71	HMDB0041367	M+Hac-H	C34H46O7	6.5407166666667	-	neg	625.34369828384	0.026555906898726	48.6	172616-90-9
neg_9230	Lpe(16:0)	metab_40457	B(i)	97.3	0	HMDB0011473	M-H	C21H44NO7P	6.54605	-	neg	452.27824404568	0.037232645705292	59.3	-
neg_9238	Malformin	metab_40465	B(ii)	0	43.7	HMDB0029429	M+Hac-H	C23H39N5O5S	6.5514	-	neg	588.25326915732	0.03858329037185	45.1	53571-13-4
neg_9243	Dodecyl Gallate	metab_40470	B(i)	44.1	0	HMDB0038720	M-H	C19H30O5	6.5566833333333	-	neg	337.19969980154	0.01078061598713	45.2	1166-52-5
neg_9245	Estradiol Enanthate	metab_40472	B(ii)	0	57.9	HMDB0251964	M-H	C25H36O3	6.5566833333333	-	neg	383.25906911633	0.003371685916338	50.2	-
neg_9248	Phe-Phe-Pro-Arg	metab_40475	B(ii)	0	64.4	HMDB0304807	M-H2O-H	C29H39N7O5	6.5566833333333	-	neg	546.28149375679	0.020631030039083	49.7	-
neg_9254	7A-Hydroxytestosterone	metab_40481	B(ii)	0	47.4	HMDB0003956	M-H	C19H28O3	6.56205	C05291	neg	303.19675150014	0.13874829625992	47.8	2141-17-5
neg_9267	Dibutyl Phthalate	metab_40494	B(i)	76.9	0	HMDB0033244	M-H	C16H22O4	6.5780833333333	C14214	neg	277.14461976906	0.041863429190454	54.3	84-74-2
neg_9278	Fahfa(18:3/3:0)	metab_40505	B(i)	96.1	0	-	M-H	C21H34O4	6.5886666666667	-	neg	349.23860498831	0.046336161688885	57.8	-
neg_9297	11-Oxahexadecanolide	metab_40524	B(ii)	0	45.3	HMDB0303921	M+Na-2H	C15H28O3	6.5996	-	neg	277.18098915239	0.047072799282052	45.1	-
neg_9318	Dgdg(O-8:0/10:0)	metab_40545	B(i)	58.5	0	-	M+FA-H	C33H62O14	6.6155333333333	-	neg	727.41142100268	0.047970641809462	49.7	-
neg_9319	Salicylic Acid	metab_40546	B(i)	79.5	0	HMDB0001895;PW_C001263	M-H	C7H6O3	6.6208666666667	C00805	neg	137.02452525317	0.019751191786859	53.5	69-72-7
neg_9323	Lpe(O-18:2)	metab_40550	B(i)	53.8	0	-	M-H	C23H46NO6P	6.6208666666667	-	neg	462.29885460315	0.20944027056403	45.9	-
neg_9328	Austalide L	metab_40555	B(ii)	0	42.3	HMDB0030158	M+Na-2H	C25H32O6	6.6262166666667	-	neg	449.1928014	0.14598431679019	43.4	87833-52-1
neg_9340	Cer(14:0_2O/6:0)	metab_40567	B(i)	66.4	0	-	M+FA-H	C20H41NO3	6.6368666666667	-	neg	388.30679261289	0.029596736507961	51.4	-
neg_9342	Mucronine D	metab_40569	B(ii)	0	45.6	HMDB0029335	M-H2O-H	C37H51N5O6	6.6368666666667	C10009	neg	642.36264961853	0.2989779092997	43.9	38496-00-3
neg_9357	Delta-Tocotrienol	metab_40584	B(ii)	0	42.4	HMDB0030008	M+FA-H	C27H40O2	6.65805	C14156	neg	441.301298	0.041212949868175	46.9	25612-59-3;
neg_9371	Fahfa(6:0/22:5)	metab_40598	B(i)	38.7	0	-	M+FA-H	C28H44O4	6.6687333333333	-	neg	489.32229603289	0.079026079124488	45.1	-

neg_9372	Lpc(18:0)	metab_40599	B(i)	95.2	0	HMDB0010384	M+FA-H	C26H54NO7P	6.6687333333333	C04230	neg	568.36220058633	0.021094249582958	58.3	19420-57-6
neg_9375	Mgdg(18:2/13:0)	metab_40602	B(i)	51.7	0	-	M-H	C40H72O10	6.6687333333333	-	neg	711.50536453167	0.027700192211269	49.1	-
neg_9378	2-Acetoxy-3-Geranylgeranyl-1,4-Dihydroxybenzene	metab_40605	B(ii)	0	42.8	HMDB0040748	M-H	C28H40O4	6.6740666666667	-	neg	439.28568746525	0.07305822992502	46.6	126398-82-1
neg_9380	Isofucosterol 3-O-[6-O-Hexadecanoyl-B-D-Glucopyranoside]	metab_40607	B(ii)	0	71.8	HMDB0032811	M-H	C51H88O7	6.6740666666667	-	neg	811.64561465271	0.037358839289272	54	209903-43-5
neg_9391	Fa(19:0)	metab_40618	B(i)	59.7	0	HMDB0000772	M+FA-H	C19H38O2	6.6846666666667	C16535	neg	343.28541387011	0.027967686102017	50.1	646-30-0
neg_9396	Ganoderiol I	metab_40623	B(ii)	0	83	HMDB0037778	M+Na-2H	C31H50O5	6.6846666666667	-	neg	523.34056759213	0.027174525269686	56.1	114567-49-6
neg_9416	Trehalose	metab_40643	B(i)	99.7	0	HMDB0000975;PW_C000776	M+FA-H	C12H22O11	6.69525	C01083	neg	387.11452375056	0.11074396036099	57.6	99-20-7
neg_9419	P-Hydroxyhippuryl-His-Leu-Oh	metab_40646	B(ii)	0	46.7	HMDB0256043	M+Na-2H	C21H27N5O6	6.69525	-	neg	466.16659029769	0.14459011614191	46.2	-
neg_9421	Fa(18:2)	metab_40648	B(i)	52.6	0	-	2M-H	C18H32O2	6.69525	-	neg	559.47329590449	0.12442981603955	48.3	-
neg_9431	8-Hydroxy-9,10-Epoxystearic Acid	metab_40658	B(i)	44.4	0	-	M-H2O-H	C18H34O4	6.7059	-	neg	295.22799849389	0.06226101794007	45.6	-
neg_9436	Carbenoxolone	metab_40663	B(ii)	0	42.8	HMDB0242751	M+Na-2H	C34H50O7	6.7059	-	neg	591.32805081622	0.036161678977347	46.4	-
neg_9439	Fahfa(2:0/16:0)	metab_40666	B(i)	85.8	0	-	M-H	C18H34O4	6.7112666666667	-	neg	313.23857655852	0.044958030248998	55.6	-
neg_9441	Lps(16:0)	metab_40668	B(i)	96.4	0	HMDB0240605	M-H	C22H44NO9P	6.7112666666667	-	neg	496.26819306286	0.019059340680097	57	116947-34-3
neg_9467	Furostanol	metab_40694	B(ii)	0	50.8	HMDB0252548	M+Na-2H	C27H46O2	6.7538166666667	-	neg	423.32693130102	0.006263117869211	48.3	-
neg_9474	Bullatanocin	metab_40701	B(ii)	0	71	HMDB0034739	M+Na-2H	C37H66O8	6.7594166666667	C08505	neg	659.45069134104	0.013202448204817	52.8	-
neg_9499	Prostaglandin G2 2-Glyceryl Ester	metab_40726	B(ii)	0	72.4	HMDB0062591	M+Cl	C23H38O8	6.8071666666667	-	neg	477.2236601	0.082706399228521	50.9	-
neg_9500	3,17-Androstanediol Glucuronide	metab_40727	B(ii)	0	67	HMDB0010321	M+Cl	C25H40O8	6.8071666666667	-	neg	503.23948999782	0.032785418235886	50.2	-
neg_9516	Cer(18:2_2O/18:2_(2Oh))	metab_40743	B(i)	88.2	0	-	M+FA-H	C36H65NO4	6.8177833333333	-	neg	620.48967979922	0.029910323552848	56.5	-
neg_9521	Cyclonormammein	metab_40748	B(ii)	0	52.1	HMDB0030164	M-H2O-H	C21H26O6	6.8231	-	neg	355.15830024692	0.16791715297361	47.9	30563-61-2
neg_9522	1-Palmitoyl Lysophosphatidic Acid	metab_40749	B(i)	91	0	-	M-H	C19H39O7P	6.8231	-	neg	409.23618787088	0.056736610976709	57.6	-
neg_9537	Arginyl-Glycyl-Glutamyl-Serine	metab_40764	B(ii)	0	64.7	HMDB0248575	M+Na-2H	C16H29N7O8	6.8284166666667	-	neg	468.18221746577	0.13983316794509	51.2	-
neg_9560	Mgdg(O-13:1/18:2)	metab_40787	B(i)	50.2	0	-	M-H	C40H72O9	6.8390833333333	-	neg	695.51024231247	0.054310103109862	48.3	-
neg_9571	Stearaldehyde	metab_40798	B(i)	74.2	0	HMDB0002384;LMFA06000098	M+FA-H	C18H36O	6.8922166666667	C01838	neg	313.27494479581	0.10165504484389	53.2	638-66-4;
neg_9573	Endomorphin-1	metab_40800	B(ii)	0	79.1	HMDB0005773	M+Cl	C34H38N6O5	6.8922166666667	C15890;_	neg	645.25634302267	0.23229693812523	51	-;189388-22-5
neg_9575	Donhexocin	metab_40802	B(ii)	0	71.2	HMDB0033087	M+Na-2H	C35H66O8	6.8975333333333	-	neg	635.45053	0.019572018657383	52.5	-
neg_9582	(3Beta,17Alpha,23S)-17,23-Epoxy-3,28,29-Trihydroxy-27-Norlanost-8-En-24-One	metab_40809	B(ii)	0	85.5	HMDB0035221	M+Na-2H	C29H46O5	6.92435	-	neg	495.30935800453	0.098880881610245	55.6	-
neg_9591	Orthothymotinic Acid	metab_40818	B(i)	69.4	0	-	M-H	C11H14O3	6.9350333333333	-	neg	193.08711604802	0.02022962840966	51.9	-
neg_9606	4-(4-Methylpiperazin-1-Yl)-7-(Trifluoromethyl)Pyrrolo[1,2-A]Quinoxaline	metab_40833	B(ii)	0	61.7	HMDB0249864	M-H	C17H17F3N4	6.9774666666667	-	neg	333.13548030332	0.070342286974944	47.8	-
neg_9607	2-Octaprenyl-3-Methyl-6-Methoxy-1,4-Benzoquinol	metab_40834	B(ii)	0	52.7	HMDB0011661	M+Na-2H	C38H60O3	6.9827666666667	-	neg	585.43135208217	0.024256181537131	47.7	-
neg_9610	15-Hydroxyicosanoic Acid	metab_40837	B(ii)	0	68	HMDB0061665	M-H	C20H40O3	6.9934166666667	-	neg	327.29050956741	0.17821149372509	52.9	-
neg_9614	Epoxymurin A	metab_40841	B(ii)	0	47	HMDB0040920	M+FA-H	C35H62O3	6.9987166666667	C08484	neg	575.46821582317	0.037356465294459	48.6	151484-64-9
neg_9620	Lpg(14:0)	metab_40847	B(i)	82.2	0	LMGP04050012	M-H	C20H41O9P	7.0199833333333	-	neg	455.24159462635	0.051861942697657	54.9	0
neg_9624	4A-Methylzymosterol-4-Carboxylic Acid	metab_40851	B(ii)	0	43.3	HMDB0006927	M-H	C29H46O3	7.0253	C15808;C06050	neg	441.33765005477	0.14261700567931	46.9	-
neg_9625	Euscaphic Acid	metab_40852	B(ii)	0	82.2	HMDB0036651	M+Na-2H	C30H48O5	7.0253	C17890	neg	509.32503961666	0.15279079640296	54.4	53155-25-2
neg_9630	(3B,4B,11B,14B)-11-Ethoxy-3,4-Epoxy-14-Hydroxy-12-Cyathen-15-Al 14-Xyloside	metab_40857	B(ii)	0	43.6	HMDB0034617	M+Cl	C27H42O8	7.0412833333333	-	neg	529.25487343533	0.039667586715899	44.3	178120-49-5
neg_9637	4-(Decan-4-Yl)Benzenesulfonic Acid	metab_40864	B(i)	80.6	0	-	M-H	C16H26O3S	7.0786333333333	-	neg	297.15309935705	0.008343216552201	54.5	-
neg_9645	Lpg(18:2)	metab_40872	B(i)	92.5	0	HMDB0240600	M-H	C24H45O9P	7.12645	-	neg	507.273031	0.011502787072503	57.3	2093174-30-0
neg_9646	Lpi(18:2)	metab_40873	B(i)	89.1	0	HMDB0240597	M-H	C27H49O12P	7.1370833333333	-	neg	595.28900461491	0.004960027817162	56.6	1425501-10-5
neg_9659	22-Hydroxydocosanoic Acid	metab_40886	B(ii)	0	48.3	HMDB0302963	M-H	C22H44O3	7.2330166666667	C19623	neg	355.32177152471	0.29551007064744	48	-;_
neg_9665	Glyceryl Lactooleate	metab_40892	B(ii)	0	66.3	HMDB0032298	M+Cl	C24H44O6	7.2917833333333	-	neg	463.28319411541	0.042698679968114	51.1	30283-16-0;
neg_9673	5-(4-Sulfophenyl)Undecane	metab_40900	B(i)	75.1	0	-	M-H	C17H28O3S	7.3344166666667	-	neg	311.16877735928	0.014493199246427	53.5	-
neg_9679	Prednisone	metab_40906	B(ii)	0	68.1	HMDB0014773	M+Na-2H	C21H26O5	7.34525	C07370	neg	379.15620746536	0.024376028699669	48.8	1953/3/2;53-03-2;
neg_9683	Hexcer(17:2_2O/18:0_O)	metab_40910	B(i)	96.5	0	-	M+FA-H	C41H77NO9	7.3558666666667	-	neg	772.55827554744	0.078732250619748	57.8	-

neg_9684	Ps(Pgf2Alpha/22:4(7Z,10Z,13Z,16Z))	metab_40911	B(ii)	0	83.1	HMDB0283219	M-H	C48H80NO13P	7.3558666666667	-	neg	908.53317809925	0.24804858654061	51.5	-
neg_9688	Glycerophospho-N-Oleoyl Ethanolamine	metab_40915	B(i)	89.1	0	-	M-H	C23H46NO7P	7.3718666666667	-	neg	478.2942282	0.031884594450886	56.3	-
neg_9697	1-Stearoylglycerophosphoserine	metab_40924	B(ii)	0	75.1	HMDB0061698	M+Na-2H	C24H48NO9P	7.3877666666667	-	neg	546.28157766245	0.022610850171584	52	-
neg_9738	Physapubescin	metab_40965	B(ii)	0	73.4	HMDB0033796	M+Na-2H	C30H42O8	7.4410166666667	-	neg	551.26056145953	0.006466194281122	51.2	74747-52-7
neg_9760	1,4-Dihydro-2-Methylbenzoicacid	metab_40987	B(ii)	0	62.7	HMDB0247383	M+Na-2H	C22H28O5	7.47305	-	neg	393.17192928278	0.026462502740501	47.9	-
neg_9778	Lpg(16:0)	metab_41005	B(i)	96	0	HMDB0240601	M-H	C22H45O9P	7.4890666666667	-	neg	483.27304862642	0.011437776469117	58.6	116870-26-9
neg_10100	Pe(O-8:0/10:0)	metab_41327	B(i)	86	0	-	M+Hac-H	C23H48NO7P	7.5050666666667	-	neg	540.33100620985	0.075992150336869	55	-
neg_10107	Lpi(18:1)	metab_41334	B(i)	81.1	0	HMDB0061693	M-H	C27H51O12P	7.4944	-	neg	597.30487223696	0.012796981356413	55.1	1114770-15-8
neg_10109	Lpi(16:0)	metab_41336	B(i)	86.8	0	HMDB0061695	M-H	C25H49O12P	7.4944	-	neg	571.28914092211	0.006616605170702	56.1	1425501-12-7
neg_10110	4-Dodecylbenzenesulfonic Acid	metab_41337	B(i)	89	0	HMDB0059915	M-H	C18H30O3S	7.4944	-	neg	325.18446104569	0.002993680254549	56.5	-
neg_10222	Sorbitan Palmitate	metab_41449	B(ii)	0	61.2	HMDB0029887	M+Cl	C22H42O6	7.3130833333333	-	neg	437.26749224044	0.023933237983682	50.3	26266-57-9;
neg_10223	Falecalcitriol	metab_41450	B(ii)	0	59.3	HMDB0252159	M-H2O-H	C27H38F6O3	7.30245	-	neg	505.25509102333	0.085347516165748	47.3	-
neg_10224	Polypodine B	metab_41451	B(ii)	0	62	HMDB0302993	M+Cl	C27H44O8	7.2917833333333	C08834;_	neg	531.27063063412	0.12618645109357	47.3	18069-14-2;_;
neg_10229	Hexcer(19:2_2O/15:0_O)	metab_41456	B(i)	96.4	0	-	M+FA-H	C40H75NO9	7.249	-	neg	758.54242752534	0.18455386610585	57.9	-
neg_10242	Zaluzanin D	metab_41469	B(ii)	0	59.7	HMDB0302712	2M-H	C17H20O4	7.1477833333333	-	neg	575.26029844558	0.046047348450045	49.2	-
neg_10243	Cer(18:0_2O/18:5)	metab_41470	B(i)	74.1	0	-	M+FA-H	C36H63NO3	7.1370833333333	-	neg	602.47917113621	0.077675626577412	51.5	-
neg_10252	Dgdg(18:2/18:2)	metab_41479	B(i)	48.2	0	-	M+FA-H	C51H88O15	7.08395	-	neg	985.61052740441	0.13849729734617	45.6	-
neg_10256	Colforsin Daropate	metab_41483	B(ii)	0	80.8	HMDB0250404	M+Cl	C27H43NO8	7.06265	-	neg	544.26592008565	0.020237164630158	53.5	-
neg_10265	Aglepristone	metab_41492	B(ii)	0	55.2	HMDB0248061	M+FA-H	C29H37NO2	7.0253	-	neg	476.27832763758	0.04239561759717	48.5	-
neg_10266	Resmethrin	metab_41493	B(ii)	0	44.7	HMDB0257162	M+FA-H	C22H26O3	7.0253	C10991	neg	383.18969591011	0.26528420079227	46.2	10453-86-8;_
neg_10271	Dodecyl Hydrogen Sulfate	metab_41498	B(i)	80.7	0	-	M-H	C12H26O4S	7.0199833333333	-	neg	265.14799091431	0.020272420684697	55.1	-
neg_10274	Retinyl Beta-Glucuronide	metab_41501	B(ii)	0	66.6	HMDB0010340	M-H2O-H	C26H38O7	7.0093333333333	-	neg	443.24759866301	0.19697248869851	49.8	-
neg_10276	Naglyser(18:0/10:0)	metab_41503	B(i)	61.5	0	-	M+FA-H	C33H62N2O7	6.9987166666667	-	neg	643.45557981727	0.025030375522421	50.2	-
neg_10282	Sorbitan Stearate	metab_41509	B(ii)	0	51.3	HMDB0029888	M+Hac-H	C24H46O6	6.9881166666667	-	neg	489.34332312751	0.12469056438829	48.5	1338-41-6;
neg_10299	Gamma-Tocotrienol	metab_41526	B(ii)	0	45.9	HMDB0012958	M-H	C28H42O2	6.9350333333333	C14155	neg	409.31129037293	0.022535586479509	48.5	-;14101-61-2;
neg_10303	Cer(20:3_2O/16:1_(2Oh))	metab_41530	B(i)	85.6	0	-	M+FA-H	C36H65NO4	6.9297	-	neg	620.48964220245	0.07608185782895	54.7	-
neg_10304	7A,12A-Dihydroxy-5B-Cholestan-3-One	metab_41531	B(ii)	0	60.6	HMDB0006887	M+Na-2H	C27H46O3	6.9297	C05453;_	neg	439.32185608687	0.027695259143273	49.2	-
neg_10309	3-Dehydroteasterone	metab_41536	B(ii)	0	68.2	HMDB0041527	M-H2O-H	C28H46O4	6.92435	_;C15792	neg	427.3219359	0.026929046654752	52.7	-;124853-28-
neg_10313	Testosterone Glucuronide	metab_41540	B(i)	41	0	HMDB0003193;LMST05010012;PW_C001802	M-H	C25H36O8	6.9081333333333	C11134	neg	463.233827	0.15285527455246	45.4	1180-25-2;
neg_10316	Muricatacin	metab_41543	B(ii)	0	64.8	HMDB0038685	2M-H	C17H32O3	6.8975333333333	-	neg	567.46321767139	0.032277253075929	50.1	134698-86-5;
neg_10317	1,1,1-Trifluoroheptadecan-2-One	metab_41544	B(ii)	0	59.6	HMDB0256073	2M+FA-H	C17H31F3O	6.8922166666667	-	neg	661.46612681949	0.029331759326655	48.7	-
neg_10324	Cer(18:0_3O/18:2_(2Oh))	metab_41551	B(i)	92.3	0	-	M+FA-H	C36H69NO5	6.86035	-	neg	640.51605323062	0.25445526035456	54.8	-
neg_10326	1-Oxohederagenin	metab_41553	B(i)	39	0	-	M+Hac-H	C30H46O5	6.8550333333333	-	neg	545.35056388098	0.17453480731633	44	-
neg_10332	Ginsenoside F1	metab_41559	B(i)	39.6	0	-;HMDB0039555	M-H	C36H62O9	6.8390833333333	C20780;_	neg	637.43263367516	0.049120738975559	45.7	-;53963-43-2
neg_10334	Cohibin B	metab_41561	B(ii)	0	44.2	HMDB0031169	M-H2O-H	C35H64O4	6.8390833333333	-	neg	529.45765330168	0.070300682891735	42.1	189508-32-5
neg_10335	Lpg(18:3)	metab_41562	B(i)	52.4	0	LMGP04050020	M-H	C24H43O9P	6.8390833333333	-	neg	505.25777962895	0.018143781115074	47.6	0
neg_10338	Ochrephilone	metab_41565	B(i)	39.8	0	-	M-H	C23H26O5	6.8390833333333	-	neg	381.17397160468	0.073786766377368	45.5	-
neg_10358	Norethindrone Acetate	metab_41585	B(i)	69.7	0	-;HMDB0255731	M-H	C22H28O3	6.8284166666667	C08152;_	neg	339.19996534206	0.053300743469139	49.2	51-98-9;_
neg_10366	24,24-Difluoro-1Alpha,25-Dihydroxyvitamin D3	metab_41593	B(ii)	0	68.7	HMDB0242645	M+Na-2H	C27H42F2O3	6.8231	-	neg	473.28292235892	0.031144109956073	51.6	_;
neg_10367	1-Oleoyl Lysophosphatidic Acid	metab_41594	B(i)	83.6	0	-	M-H	C21H41O7P	6.8231	-	neg	435.25176310675	0.036778520681806	56.3	-
neg_10372	Isophthalic Acid	metab_41599	B(i)	82.9	0	-	M-H	C8H6O4	6.8231	C22203	neg	165.01937455105	0.059260297156974	55.1	121-91-5
neg_10376	Glycerophospho-N-Palmitoyl Ethanolamine	metab_41603	B(i)	36.7	0	-	M+Na-2H	C21H44NO7P	6.8177833333333	-	neg	474.26313664134	0.024266333739918	44.9	-
neg_10380	Mgdg(13:0/16:0)	metab_41607	B(i)	38.7	0	-	M-H	C38H72O10	6.8124833333333	-	neg	687.50554114587	0.078883880599019	44.9	-
neg_10382	Fahfa(18:1/3:0)	metab_41609	B(i)	92.2	0	-	M-H	C21H38O4	6.8124833333333	-	neg	353.2698495	0.038350676187914	57.5	-
neg_10383	Fahfa(16:0/3:0)	metab_41610	B(i)	93.8	0	-	M-H	C19H36O4	6.8124833333333	-	neg	327.25420053463	0.046867441638703	57.9	-
neg_10384	(R)-2-Hydroxystearic Acid	metab_41611	B(i)	97	0	-	M-H	C18H36O3	6.8124833333333	C03042	neg	299.25919978817	0.075278824270162	58.8	629-22-1

neg_10385	Mgdg(13:0/18:1)	metab_41612	B(i)	35.6	0	-	M-H	C40H74O10	6.8071666666667	-	neg	713.52097927844	0.061844603187908	44.2	-
neg_10392	Cer(15:0_2O/7:0)	metab_41619	B(i)	58.9	0	-	M+FA-H	C22H45NO3	6.8071666666667	-	neg	416.33823310476	0.13313408866778	49.4	-
neg_10416	Fahfa(18:2/16:1)	metab_41643	B(i)	36.2	0	-	M+Hac-H	C34H60O4	6.7594166666667	-	neg	591.46313119518	0.005816027452696	46.9	-
neg_10420	Dg(Txb2/I-16:0/0:0)	metab_41647	B(ii)	0	67	HMDB0299217	M+Na-2H	C39H70O9	6.7538166666667	-	neg	703.47686771591	0.068266774596469	49.4	-
neg_10438	Lps(18:1)	metab_41665	B(i)	95.1	0	HMDB0240603	M-H	C24H46NO9P	6.7165666666667	-	neg	522.28398093625	0.048674709657088	55.8	89319-60-8
neg_10440	Heptadecanal	metab_41667	B(ii)	0	58.2	HMDB0031039	M+Hac-H	C17H34O	6.7165666666667	-	neg	313.2749184	0.2115219993664	50.9	629-90-3;
neg_10450	Fahfa(18:2/16:2)	metab_41677	B(i)	36.1	0	-	M+Hac-H	C34H58O4	6.7059	-	neg	589.44742973244	0.044969128815966	45.5	-
neg_10455	Terephthalic Acid	metab_41682	B(i)	81	0	HMDB0002428	M-H	C8H6O4	6.7059	C06337	neg	165.01937577676	0.009097658881951	54.7	100-21-0
neg_10457	Fmnh2	metab_41684	B(ii)	0	68	HMDB0001142	M-H	C17H23N4O9P	6.7005666666667	C01847	neg	457.11310251772	0.068413788136001	50.7	-;5666-16-0
neg_10465	Pe-Cer(12:1_2O/12:0)	metab_41692	B(i)	41.1	0	-	M+FA-H	C26H53N2O6P	6.69525	-	neg	565.35946738157	0.019171939525366	46.3	-
neg_10471	1,5-Dibutyl Methyl Hydroxycitrate	metab_41698	B(ii)	0	63.9	HMDB0040461	M+FA-H	C15H26O8	6.69525	-	neg	379.15826826559	0.015448846082479	49.2	-
neg_10473	3B,15B,17A-Trihydroxy-Pregnenone	metab_41700	B(ii)	0	61.2	HMDB0000353	M-H	C21H32O4	6.69525	-	neg	347.2204075	0.031689174197413	48.9	80380-40-1
neg_10485	Lpa(18:2)	metab_41712	B(i)	76.5	0	HMDB0007852	M-H	C21H39O7P	6.6899666666667	-	neg	433.23618819334	0.026107009472586	55	-
neg_10493	Stearoyl Lysophosphatidylethanolamine	metab_41720	B(i)	97.8	0	-	M-H	C23H48NO7P	6.6846666666667	-	neg	480.3097375	0.028972973409259	58.8	-
neg_10497	Fahfa(18:2/3:0)	metab_41724	B(i)	98.3	0	-	M-H	C21H36O4	6.6846666666667	-	neg	351.25406781126	0.024363931155222	58.9	-
neg_10499	Prosaikogenin A	metab_41726	B(ii)	0	71.9	HMDB0256850	M-H	C36H58O8	6.6793666666667	-	neg	617.40598155533	0.0749977093412	51.9	-
neg_10502	Digitoxigenin	metab_41729	B(ii)	0	47.3	HMDB0251274	M+FA-H	C23H34O4	6.6793666666667	-	neg	419.24157250117	0.033344874762983	46.4	_;
neg_10503	Fahfa(18:2/4:0)	metab_41730	B(i)	93.8	0	-	M-H	C22H38O4	6.6793666666667	-	neg	365.26957184471	0.10048979885518	57.1	-
neg_10513	Ginsenoside Rh7	metab_41740	B(ii)	0	78.7	HMDB0039446	M-H	C36H60O9	6.6687333333333	-	neg	635.41666297006	0.10620064732018	54.7	343780-68-7
neg_10516	Fahfa(16:0/5:0)	metab_41743	B(i)	45.9	0	-	M+Cl	C21H40O4	6.6687333333333	-	neg	391.26209694263	0.01216115582378	44.7	-
neg_10519	5-Hexyltetrahydro-2-Furanoctanoic Acid	metab_41746	B(ii)	0	42.4	HMDB0031127	M-H	C18H34O3	6.6687333333333	-	neg	297.24358305828	0.031717736621888	47.7	61781-98-4
neg_10523	Androsterone Glucuronide	metab_41750	B(ii)	0	82.3	HMDB0002829	M+Cl	C25H38O8	6.6633833333333	C11135;C11136	neg	501.22377280358	0.021104784073851	54	1852-43-3;3602-09-3
neg_10525	15-Hexadecanolide	metab_41752	B(ii)	0	44.8	HMDB0031711	M+FA-H	C16H30O2	6.6633833333333	-	neg	299.22282348713	0.066557631619409	47	69297-56-9
neg_10533	Arachidonic Acid	metab_41760	B(i)	60.7	0	HMDB0001043;LMFA01030001;PW_C000821	M-H	C20H32O2	6.65805	C00219	neg	303.23301029712	0.049223907940009	50.2	506-32-1;
neg_10534	2-Hydroxypalmitic Acid	metab_41761	B(i)	95.4	0	HMDB0031057	M-H	C16H32O3	6.65805	-	neg	271.22789099491	0.022966084936343	58.5	16452-52-1
neg_10547	1-Myristoyl-Sn-Glycerol 3-Phosphate	metab_41774	B(i)	62.1	0	LMGP10050007	M-H	C17H35O7P	6.6368666666667	-	neg	381.20477541531	0.046995571304591	50.6	0
neg_10548	12'-Apo-B-Carotene-3,12'-Diol	metab_41775	B(ii)	0	50.7	HMDB0036054	M-H	C25H36O2	6.6368666666667	-	neg	367.26417084578	0.031325252609738	49.4	120021-87-6
neg_10563	Rollitacin	metab_41790	B(ii)	0	85.7	HMDB0031136	M+Na-2H	C37H66O8	6.6208666666667	-	neg	659.45018873917	0.2132396410918	54.1	187523-67-7
neg_10570	Lpe(O-16:1)	metab_41797	B(i)	37.9	0	-	M-H	C21H44NO6P	6.6155333333333	-	neg	436.28375028061	0.042116518068383	44.3	-
neg_10571	N-Palmitoyl Threonine	metab_41798	B(ii)	0	43.3	HMDB0241933	M-H	C20H39NO4	6.6155333333333	-	neg	356.28062062611	0.11227370777783	44.8	_;
neg_10582	[(3S,4S,5S,6R)-3,4,5-Trihydroxy-6-(Hydroxymethyl)Oxan-2-Yl] Octadec-9-Enoate	metab_41809	B(ii)	0	82.3	HMDB0260228	M+FA-H	C24H44O7	6.5996	-	neg	489.30693254166	0.0022765531842512	56.1	-
neg_10587	Docosahexaenoic Acid	metab_41814	B(i)	73.3	0	HMDB0002183;PW_C001472	M-H	C22H32O2	6.5996	C06429	neg	327.23285881613	0.084756157737168	52.9	-;6217-54-5
neg_10588	9-Hydroxy-10,12-Octadecadienoic Acid	metab_41815	B(i)	48	0	LMFA02000318	M-H2O-H	C18H32O3	6.5996	-	neg	277.21744070927	0.022632678311319	48.1	0
neg_10595	Teneligliptin	metab_41822	B(ii)	0	46.3	HMDB0258815	M-H2O-H	C22H30N6OS	6.59425	-	neg	407.20298278127	0.048670668928708	44.7	-
neg_10596	Gartanin	metab_41823	B(ii)	0	50.9	HMDB0030700	M-H2O-H	C23H24O6	6.59425	C10063	neg	377.1426699	0.021056062145703	47.5	33390-42-0
neg_10607	13,14-Dihydro Pgf-1A	metab_41834	B(ii)	0	64.6	HMDB0005076	M+Hac-H	C20H38O5	6.5886666666667	-	neg	417.28588776116	0.021099214838578	52.4	-
neg_10608	Spiroxamine	metab_41835	B(ii)	0	44.7	HMDB0258432	M+FA-H	C18H35NO2	6.5886666666667	C11124	neg	342.2649353	0.039033466452259	47.3	118134-30-8;_
neg_10614	Lpa(18:3)	metab_41841	B(i)	74.4	0	HMDB0114743	M-H	C21H37O7P	6.5833833333333	-	neg	431.22052328513	0.029189029307205	53.9	-
neg_10619	11-Hydroxy-12-Methyltetradecanoic Acid	metab_41846	B(i)	46.6	0	-	M-H	C15H30O3	6.5780833333333	-	neg	257.21235355791	0.068643917872734	46.6	-
neg_10633	2,3-Dinor-Txb1	metab_41860	B(ii)	0	57.9	HMDB0245413	M+Na-2H	C19H34O5	6.56205	-	neg	363.2154903	0.10044366694559	48.5	_;
neg_10637	5-Hydroxylysinonorleucine	metab_41864	B(ii)	0	55.3	HMDB0246805	2M-H	C12H25N3O5	6.5566833333333	-	neg	581.35454593433	0.075400300014432	42.1	-
neg_10639	St(29:2_O_S)	metab_41866	B(i)	50.3	0	-	M+FA-H	C29H48O4S	6.5566833333333	-	neg	537.32819427033	0.018762131267446	47.8	-
neg_10640	Lpe(18:1)	metab_41867	B(i)	96.6	0	HMDB0011475	M-H	C23H46NO7P	6.5566833333333	-	neg	478.29413609696	0.021494316420865	58.8	-
neg_10666	Lpc(18:1)	metab_41893	B(i)	98	0	HMDB0002815	M+FA-H	C26H52NO7P	6.53535	-	neg	566.34649386084	0.017790540963196	59.3	19420-56-5

neg_10670	2-(20-Hydroxyicosa-5,14-Dienoylamino)Acetic Acid	metab_41897	B(ii)	0	52.6	HMDB0255357	M-H	C22H39NO4	6.5300166666667	-	neg	380.2807099	0.16093783353096	47.6	-
neg_10671	N-Linoleoyl Gaba	metab_41898	B(ii)	0	52.7	HMDB0062334	M-H	C22H39NO3	6.5300166666667	-	neg	364.28575145239	0.038782602403159	49.3	-
neg_10674	Pc(Pgf1Alpha/24:0)	metab_41901	B(ii)	0	45.5	HMDB0288691	M+Na-2H	C52H100NO11	6.5246833333333	-	neg	966.6831646	0.049992616666726	45.6	-
neg_10675	1-O-[(2Alpha,3Beta)-2,3-Dihydroxy-28-Oxoolean-12-En-28-Yl]-Beta-D-Glucopyranose	metab_41902	B(i)	98.7	0	-	M-H	C36H58O9	6.5246833333333	-	neg	633.40094583301	0.10868084099601	58.4	-
neg_10677	Cer(14:2_2O/7:0)	metab_41904	B(i)	66.3	0	-	M+FA-H	C21H39NO3	6.5246833333333	-	neg	398.29134009528	0.055703619293337	50.7	-
neg_10678	Rickinic Acid A	metab_41905	B(i)	94.6	0	-	M-H	C15H22O3	6.5246833333333	-	neg	249.14969525761	0.016922554992101	57.9	-
neg_10683	Lpc(16:0)	metab_41910	B(i)	96.9	0	HMDB0010382	M+FA-H	C24H50NO7P	6.5193833333333	C04230	neg	540.33096734371	0.030771172668845	59.2	17364-16-8
neg_10699	Hexosyl Lpe(16:0)	metab_41926	B(i)	41.2	0	-	M-H	C27H54NO12P	6.5035	-	neg	614.33104997939	0.032749190896965	47.4	-
neg_10716	Dgdg(O-8:0/8:0)	metab_41943	B(i)	58.1	0	-	M+FA-H	C31H58O14	6.4929	-	neg	699.38102363869	0.03981894030924	51.2	-
neg_10722	Boldenone	metab_41949	B(ii)	0	44.4	HMDB0249339	2M+FA-H	C19H26O2	6.4875833333333	C14502;_	neg	617.38468557732	0.064719987099927	45.5	846-48-0;_
neg_10724	Nirvanol	metab_41951	B(ii)	0	49.3	HMDB0060533	M+Cl	C11H12N2O2	6.4875833333333	C14916	neg	239.05994943967	0.10784263009136	47.5	631-07-2;_
neg_10725	Lucyoside N	metab_41952	B(ii)	0	84	HMDB0041040	M+Cl	C36H58O10	6.4822333333333	-	neg	685.37216939601	0.18995034323651	55.7	152845-77-7
neg_10727	Sapacitabine	metab_41954	B(ii)	0	66.9	HMDB0257481	M+Na-2H	C26H42N4O5	6.4822333333333	-	neg	511.28822968931	0.049577119431604	49.4	-
neg_10728	Mevastatin	metab_41955	B(ii)	0	41.2	HMDB0254692	M+FA-H	C23H34O5	6.4822333333333	_;C13963	neg	435.23846142312	0.059151126095412	45.5	73573-88-3;_
neg_10736	Mgmg(18:2)	metab_41963	B(i)	42.4	0	-	M+FA-H	C27H48O9	6.4769166666667	-	neg	561.32816400493	0.016437824860252	48.2	-
neg_10746	Estreptoquinasa	metab_41973	B(ii)	0	50.1	HMDB0258508	2M-H	C11H19NO2	6.4716	-	neg	393.27608567442	0.051253399076041	48.9	-
neg_10758	Cryptomeridiol 11-Rhamnoside	metab_41985	B(ii)	0	49.8	HMDB0038018	M+Na-2H	C21H38O6	6.4609166666667	-	neg	407.24185018319	0.041160491922616	47.3	349112-30-7
neg_10778	Prostaglandin D1 Alcohol	metab_42005	B(i)	68.8	0	-	M-H	C20H36O4	6.4502333333333	-	neg	339.25411642206	0.016054230859955	52.9	-
neg_10785	Norcholic Acid	metab_42012	B(ii)	0	53.2	HMDB0255727	M+Na-2H	C23H38O5	6.4449333333333	-	neg	415.2492954	0.019806264	48	_;
neg_10786	6-((8Z,11Z,14Z)-Heptadeca-8,11,14-Trien-1-Yl)Salicylic Acid	metab_42013	B(i)	38.8	0	LMPK15040005	M-H	C24H34O3	6.4449333333333	-	neg	369.2432773	0.1310452970925	44.5	0
neg_10791	Lpi(18:0)	metab_42018	B(i)	48.8	0	HMDB0061704	M-H	C27H53O12P	6.4396333333333	-	neg	599.31988750397	0.12645995717699	47.1	1913287-87-2
neg_10797	Lpc(18:2)	metab_42024	B(i)	95.1	0	HMDB0010386	M+FA-H	C26H50NO7P	6.4343333333333	-	neg	564.33096525704	0.021061436711133	58.8	22252-07-9
neg_10798	Lpe(16:1)	metab_42025	B(i)	92.1	0	HMDB0011474	M-H	C21H42NO7P	6.4343333333333	-	neg	450.26285676309	0.042166723466606	56.9	-
neg_10803	Isodihydroauroglaucin	metab_42030	B(i)	43.5	0	-	M-H	C19H24O3	6.4343333333333	-	neg	299.16535240706	0.046383418696938	47.4	-
neg_10817	2,6-Di-Tert-Butyl-4-Nitrophenol	metab_42044	B(i)	70.8	0	-	M-H	C14H21NO3	6.4234333333333	-	neg	250.14498973285	0.043626142152905	53.2	-
neg_10821	Lpe(14:0)	metab_42048	B(i)	77	0	HMDB0011470	M-H	C19H40NO7P	6.4181166666667	-	neg	424.24700411915	0.047876449863623	54.4	-
neg_10823	Fisetin	metab_42050	B(i)	60.7	0	-;LMPK12111566	M-H	C15H10O6	6.4181166666667	C10041	neg	285.04059964089	0.03799795349849	51.3	528-48-3;
neg_10827	25-Acetylvulgaroside	metab_42054	B(ii)	0	72.4	HMDB0041365	M-H2O-H	C27H42O7	6.41275	-	neg	459.27309946641	0.013325851851645	51.8	172616-88-5
neg_10831	Pc(Pgd1/P-16:0)	metab_42058	B(ii)	0	77	HMDB0289520	M-H	C44H82NO10P	6.4074	-	neg	814.56056794087	0.10305088061148	49.9	-
neg_10836	Echinocystic Aicd	metab_42063	B(i)	53.5	0	-	M-H	C30H48O4	6.4074	-	neg	471.34822279239	0.10376877954513	48.9	-
neg_10839	12-Oxo-2,3-Dinor-10,15-Phytodienoic Acid	metab_42066	B(i)	53.8	0	HMDB0032090	M-H	C16H24O3	6.4074	-	neg	263.16534886805	0.01997926316401	49.8	197247-23-7
neg_10840	Glyceric Acid	metab_42067	B(i)	85.6	0	HMDB0000139;PW_C000090	M+Hac-H	C3H6O4	6.4074	C00258	neg	165.04084319019	0.092695837734276	55.7	473-81-4
neg_10842	Dg(Pgj2/I-19:0/0:0)	metab_42069	B(ii)	0	52.8	HMDB0299828	M+Na-2H	C42H72O7	6.4021166666667	-	neg	709.50236629343	0.058433100698115	47.7	-
neg_10845	Lpe(22:6)	metab_42072	B(i)	84.6	0	HMDB0011496	M-H	C27H44NO7P	6.4021166666667	-	neg	524.27822772136	0.028468851100222	55.1	-
neg_10846	(9R,10E,12Z,15Z)-9-Hydroxyoctadeca-10,12,15-Trienoic Acid	metab_42073	B(i)	93.6	0	-	M-H	C18H30O3	6.4021166666667	-	neg	293.21231838372	0.02309567936223	58	-
neg_10848	(9E)-10-Nitrooctadec-9-Enoylcarnitine	metab_42075	B(ii)	0	78.9	HMDB0241823	2M+FA-H	C25H46N2O6	6.3967666666667	-	neg	985.66154615759	0.002921104876326	49.1	-
neg_10852	Lpe(18:3)	metab_42079	B(i)	95	0	HMDB0011478	M-H	C23H42NO7P	6.3967666666667	-	neg	474.26271117908	0.020396441222997	57.7	-
neg_10854	Pa(Pgf2Alpha/22:1(13Z))	metab_42081	B(ii)	0	56.1	HMDB0265685	M-H2O-H	C45H81O11P	6.3914666666667	-	neg	809.54192474176	0.060141025920815	48.6	-
neg_10868	Dg(Pgj2/I-15:0/0:0)	metab_42095	B(ii)	0	77.6	HMDB0298998	M-H2O-H	C38H64O7	6.3861666666667	-	neg	613.4450113	0.058860339233341	53.5	-
neg_10874	Tyr-Ser-Gln	metab_42101	B(i)	70	0	-	M-H	C17H24N4O7	6.3861666666667	-	neg	395.15329291382	0.19667127061301	50.3	-
neg_10875	16B-Hydroxystanozolol	metab_42102	B(ii)	0	74.2	HMDB0003166	M+Na-2H	C21H32N2O2	6.3861666666667	-	neg	365.22102224023	0.068658854547513	50.6	125590-76-3
neg_10878	8-Hydroxylinoleic Acid	metab_42105	B(i)	77.6	0	-	M+Cl	C18H32O3	6.3861666666667	C08318	neg	331.20447450319	0.016452970790877	54.3	138231-04-6
neg_10879	Coriolic Acid	metab_42106	B(i)	94.5	0	LMFA02000154	M-H	C18H32O3	6.3861666666667	C14762	neg	295.22780665345	0.018466538507923	58.3	0
neg_10884	Mangalkanyl Glucoside	metab_42111	B(ii)	0	45.5	HMDB0036015	M+Na-2H	C21H38O6	6.3808666666667	-	neg	407.24146406809	0.086350685020636	47.3	259144-63-3
neg_10888	Dg(Pgf2Alpha/0:0/15:0)	metab_42115	B(ii)	0	76.8	HMDB0295254	M-H2O-H	C38H68O8	6.3755666666667	-	neg	633.47096275009	0.026749002377234	52.2	-

neg_10897	Hexosyl Lpe 18:3	metab_42124	B(i)	59.5	0	-	M-H	C29H52NO12P	6.3702333333333	-	neg	636.31555440808	0.028729637448967	50.3	-
neg_10901	1A,1B-Dihomo Prostaglandin F2Alpha	metab_42128	B(i)	40.6	0	-	M+FA-H	C22H38O5	6.3702333333333	-	neg	427.26809674694	0.051916110272003	44.5	-
neg_10902	Methyl (9Z,14Z)-12,13,16-Trihydroxyoctadeca-9,14-Dienoate	metab_42129	B(i)	36.8	0	-	M+Cl	C19H34O5	6.3702333333333	-	neg	377.20994706534	0.007478544099977	45.1	-
neg_10904	9,10-Dihydroxystearic Acid	metab_42131	B(ii)	0	65.4	HMDB0302281	M-H	C18H36O4	6.3702333333333	C19622;_;C0831	neg	315.25411078315	0.007469697681335	52.4	120-87-6;_;
neg_10916	St(24:2_O4)	metab_42143	B(i)	36.6	0	-	M-H	C24H38O4	6.3596333333333	-	neg	389.26981302905	0.021464972913329	45.5	-
neg_10942	11,11-Dimethyl-8-Methylenebicyclo[7.2.0]Undec-4-Ene-4-Carboxylic Acid	metab_42169	B(i)	87.4	0	-	M+FA-H	C15H22O2	6.3489833333333	-	neg	279.16029584874	0.012516293738597	56.6	-
neg_10944	(3R)-3-Hydroxydodecanoic Acid	metab_42171	B(i)	68.4	0	HMDB0010728	M-H	C12H24O3	6.3489833333333	-	neg	215.16536669745	0.018470253937827	52.6	-
neg_10952	Aloe-Emodin	metab_42179	B(i)	75.6	0	HMDB0030829;LMPK13040002	M-H	C15H10O5	6.3436333333333	C10294	neg	269.04567364233	0.070497508219757	53.8	481-72-1;
neg_10955	3-Trans-P-Coumaroylrotundic Acid	metab_42182	B(ii)	0	86.1	HMDB0040667	M-H	C39H54O7	6.3382833333333	-	neg	633.37970548168	0.1061956947077	56	144624-03-3
neg_10975	Tropolone A	metab_42202	B(ii)	0	91.2	HMDB0255800	M+Cl	C24H33NO6	6.3223333333333	-	neg	466.20368656313	0.13362935463849	52.4	-
neg_10985	Physapruin B	metab_42212	B(ii)	0	42.1	HMDB0040671	M+Na-2H	C34H50O9	6.3169833333333	-	neg	623.3178231	0.089874518405926	46.1	-
neg_10989	Prostaglandin E2 Methyl Ester	metab_42216	B(ii)	0	47.2	HMDB0256853	M+Hac-H	C21H34O5	6.3169833333333	-	neg	425.25229146312	0.068874195484084	46.6	-
neg_11005	5,7-Megastigmadien-9-Ol Glucoside	metab_42232	B(ii)	0	59.5	HMDB0041044	M+Na-2H	C19H32O6	6.3116833333333	-	neg	377.19458317724	0.030910636404327	50.3	146610-77-7
neg_11006	2,3-Dinor-8-Iso-Pgf2A	metab_42233	B(i)	41.6	0	-	M-H	C18H30O5	6.3116833333333	-	neg	325.20206333495	0.028149814491997	46.2	-
neg_11017	Fa(18:3+2O)	metab_42244	B(i)	75.6	0	-	M-H	C18H30O4	6.3063333333333	-	neg	309.20723286001	0.004263480679642	54.4	-
neg_11022	Dg(5-Iso Pgf2Vi/0:0/15:0)	metab_42249	B(ii)	0	56.7	HMDB0295282	M+Na-2H	C36H64O8	6.30105	-	neg	645.43475090172	0.034962175112754	48.4	-
neg_11028	Fa(14:0)	metab_42255	B(i)	64.4	0	HMDB0000806	M+Hac-H	C14H28O2	6.30105	C06424	neg	287.2228186	0.007811517545664	52.3	544-63-8
neg_11044	9(S)-Hpode	metab_42271	B(i)	81	0	HMDB0006940;HMDB0062434;HMDB0006940	M-H	C18H32O4	6.2904	C14827	neg	311.22282284107	0.007566576143767	55.6	29774-12-7;_
neg_11051	Dg(Txb2/I-19:0/0:0)	metab_42278	B(ii)	0	69	HMDB0299840	M-H	C42H76O9	6.28505	-	neg	723.53532809098	0.018545034950891	47.9	-
neg_11059	2-Stearoylglycerophosphoglycerol	metab_42286	B(ii)	0	51.6	HMDB0061703	M+Cl	C24H49O9P	6.28505	-	neg	547.28042846105	0.021270181372693	49.3	-
neg_11098	Isoprostane F2Alpha-I	metab_42325	B(i)	40.5	0	HMDB0243554	M+FA-H	C20H34O5	6.2689833333333	-	neg	399.2365722	0.009104417407972	45.5	-
neg_11106	4-(2,6,6-Trimethyl-1-Cyclohexenyl)-2-Butanol	metab_42333	B(ii)	0	44.5	HMDB0036172	M+FA-H	C13H24O	6.2689833333333	-	neg	241.18101958012	0.027866399847633	46.6	3293-47-8
neg_11135	Decenoylcarnitine	metab_42362	B(ii)	0	51.6	HMDB0250918	M+Na-2H	C17H31NO4	6.2527833333333	-	neg	334.20232479256	0.037483345083793	48	-
neg_11137	Tanacetol A	metab_42364	B(ii)	0	53	HMDB0035722	M-H	C17H26O4	6.2527833333333	-	neg	293.17602660787	0.010769249378016	49.5	86778-06-5
neg_11140	(Z)-2-Oct-7-Enylpent-2-Enedioic Acid	metab_42367	B(i)	84.2	0	-	M-H	C13H20O4	6.2527833333333	-	neg	239.12898811219	0.012581296340265	56	-
neg_11141	Geranyl Acetate	metab_42368	B(i)	67.6	0	HMDB0035157	M-H	C12H20O2	6.2527833333333	C09861	neg	195.13914734263	0.011278842236818	52.5	105-87-3
neg_11148	Ptaquiloside	metab_42375	B(ii)	0	50.1	HMDB0242690	M+Na-2H	C20H30O8	6.2474333333333	C19515;_	neg	419.16643555218	0.086625135271771	46.9	87625-62-5;_
neg_11180	(5Z,9E,14Z)-(8Xi,11R,12S)-11,12-Epoxy-8-Hydroxyicosa-5,9,14-Trienoic Acid	metab_42407	B(ii)	0	41.7	HMDB0062619	M+Hac-H	C20H32O4	6.2315	C04849	neg	395.24394665965	0.015203506990197	45	-;_
neg_11194	Annoglaxin	metab_42421	B(ii)	0	52	HMDB0033603	M+FA-H	C35H62O8	6.22085	-	neg	655.44087061163	0.049065801914049	47.3	-
neg_11195	Frangulanine	metab_42422	B(ii)	0	57	HMDB0030199	M-H	C28H44N4O4	6.22085	C10003	neg	499.32707025861	0.063197144281127	46.4	25350-22-5
neg_11212	7-[2-(1-Hydroxyhexyl)-3,6-Dihydro-2H-Pyran-6-Yl]Heptanoic	metab_42439	B(i)	65.7	0	-	M+Hac-H	C18H32O4	6.21555	-	neg	371.24410138691	0.013763115440052	51.9	-
neg_11213	Arctiopicrin	metab_42440	B(ii)	0	60.1	HMDB0301857	M-H	C19H26O6	6.21555	C09297	neg	349.1634616	0.038238890296059	48.7	19889-01-1;_
neg_11214	(E)-8,9,10-Trihydroxyoctadec-6-Enoic Acid	metab_42441	B(i)	76.2	0	-	M-H	C18H34O5	6.21555	-	neg	329.23337627342	0.044666485407525	54.6	-
neg_11216	Ethotoin	metab_42443	B(ii)	0	44.6	HMDB0014892	M+Cl	C11H12N2O2	6.21555	C07839	neg	239.05983105053	0.063408134737439	46.4	86-35-1
neg_11239	Lupinic Acid	metab_42466	B(ii)	0	40.2	HMDB0302524	M+Hac-H	C10H17NO2	6.2049166666667	C01513;_	neg	242.13992993242	0.064227258849445	46.7	67392-79-4;_
neg_11246	Mgdg(2:0/13:1)	metab_42473	B(i)	69.8	0	-	M+FA-H	C24H42O10	6.1996166666667	-	neg	535.27569175688	0.039972035481978	52.6	-
neg_11254	Alpha-Bisabolol Oxide A	metab_42481	B(ii)	0	53.2	HMDB0038196	M+FA-H	C15H26O2	6.1996166666667	C16773	neg	283.19152571153	0.014251300222245	50.2	22567-36-8
neg_11268	1-(4-O-Beta-D-Glucopyranosyl-3-Methoxyphenyl)-3,5-Dihydroxydecane	metab_42495	B(ii)	0	79.1	HMDB0303085	M-H	C25H42O7	6.1889666666667	-	neg	453.28574448471	0.027713918174738	54.9	-
neg_11272	Coumarin-Saha	metab_42499	B(i)	44.4	0	-	M-H	C18H22N2O5	6.1889666666667	-	neg	345.14716608706	0.12681314711145	45.4	-
neg_11275	Cucurbic Acid	metab_42502	B(ii)	0	57.2	HMDB0029388	M-H	C12H20O3	6.1889666666667	C08482;_	neg	211.13401960056	0.018253835891824	50.8	58240-50-9
neg_11279	8-Isoprostaglandin F1Alpha	metab_42506	B(i)	35.8	0	-	M-H	C20H36O5	6.1836333333333	-	neg	355.24923366725	0.012954064330396	44.2	-
neg_11284	(8E,10E)-12-Oxotrideca-8,10-Dienoic Acid	metab_42511	B(i)	47.4	0	-	M-H	C13H20O3	6.1836333333333	-	neg	223.13405594936	0.032096382472151	48.7	-
neg_11322	Ipomeatetrahydrofuran	metab_42549	B(ii)	0	66.9	HMDB0040904	M+FA-H	C15H28O3	6.1676833333333	-	neg	301.20210991664	0.046994191258705	52.9	92448-62-9
neg_11324	Cycloheximide	metab_42551	B(ii)	0	43.7	HMDB0250657	M-H2O-H	C15H23NO4	6.1676833333333	C06685;_	neg	262.1449578	0.077280618551859	47.8	66-81-9;_

neg_11325	2-Octylpent-2-Enedioic Acid	metab_42552	B(i)	61.6	0	-	M-H	C13H22O4	6.1676833333333	-	neg	241.14470755791	0.077281109332336	50	-
neg_11338	Tetradecanedioic Acid	metab_42565	B(i)	67.2	0	HMDB0000872;LMFA01170018	M-H	C14H26O4	6.1570333333333	_;C11002	neg	257.17590022682	0.020151715659581	52.8	821-38-5;
neg_11358	B-Octylglucoside	metab_42585	B(ii)	0	55.3	HMDB0245753	M-H2O-H	C14H28O6	6.14645	-	neg	273.17082291876	0.01886606022923	50.3	-
neg_11360	Saponin E	metab_42587	B(ii)	0	40.3	HMDB0035958	M+Na-2H	C42H68O14	6.14115	-	neg	817.43072118939	0.089770865903111	42.4	85191-73-7
neg_11371	3Z-Dodecenedioic Acid	metab_42598	B(i)	49.1	0	LMFA01170137	M-H2O-H	C12H20O4	6.14115	-	neg	209.11834450667	0.029523553810103	49.3	0
neg_11386	Dg(Pge2/I-12:0/0:0)	metab_42613	B(ii)	0	63.4	HMDB0298359	M+FA-H	C35H60O8	6.1305	-	neg	653.42472454345	0.074049667496524	50.7	-
neg_11390	Fa(18:2+3O)	metab_42617	B(i)	67.3	0	-	M-H	C18H32O5	6.1305	-	neg	327.21780576154	0.01095596624184	52.8	-
neg_11391	Helenalin	metab_42618	B(ii)	0	68.5	HMDB0253066	M-H	C15H18O4	6.1305	_;C09473	neg	261.11098548135	0.097320553242296	49.2	6754-13-8;_
neg_11403	Decanal	metab_42630	B(i)	46.1	0	HMDB0011623;LMFA06000052	M+FA-H	C10H20O	6.1252	C12307	neg	201.14968869727	0.033728188821538	48.4	112-31-2;
neg_11406	Oxidized Glutathione	metab_42633	B(ii)	0	49.9	HMDB0003337	M-H2O-H	C20H32N6O12S2	6.1198666666667	C00127	neg	593.13016278994	0.037487026314192	46.1	27025-41-8
neg_11408	Nandrolone Glucuronide	metab_42635	B(i)	51.2	0	-	M-H	C24H34O8	6.1198666666667	-	neg	449.22132970384	0.058506465777789	46.5	-
neg_11426	Ochromycinone	metab_42653	B(i)	45.7	0	HMDB0258477	M-H	C19H14O4	6.1145166666667	-	neg	305.08177584654	0.006584817671689	47	-
neg_11437	Abexinostat	metab_42664	B(ii)	0	46.5	HMDB0256171	M+Hac-H	C21H23N3O5	6.1091833333333	-	neg	456.17786973713	0.12401182492233	48.8	-
neg_11442	Phloionolic Acid	metab_42669	B(i)	83.3	0	HMDB0034295;LMFA02000147	M-H	C18H36O5	6.1091833333333	C19621;_	neg	331.24912436315	0.0174067578374	55.6	17705-68-9;
neg_11445	Decanoic Acid	metab_42672	B(i)	67.7	0	HMDB0000511;HMDB0000513;HMDB0000511;LMFA01010010	M+Hac-H	C10H20O2	6.1091833333333	C01571	neg	231.16023861419	0.034397865149248	53.2	334-48-5;
neg_11446	Ibuprofen	metab_42673	B(i)	55.3	0	HMDB0001925;PW_C001284	M-H	C13H18O2	6.1091833333333	C01588	neg	205.12347666223	0.03798414728685	50.4	15687-27-1
neg_11451	Metapro	metab_42678	B(ii)	0	57.3	HMDB0256718	M+Na-2H	C22H36N2O6	6.1038333333333	-	neg	445.2300399	0.017631967689153	48.7	-
neg_11465	Aleuretic Acid	metab_42692	B(i)	70.3	0	-	M-H	C16H32O5	6.0985333333333	-	neg	303.21760108639	0.007173985762242	53	-
neg_11468	Hexyl Glucoside	metab_42695	B(ii)	0	53.3	HMDB0031688	M-H2O-H	C12H24O6	6.0985333333333	-	neg	245.13957864629	0.031572169273546	49.2	59080-45-4
neg_11469	Dihydroisocarveol	metab_42696	B(ii)	0	79.5	HMDB0302262	M+FA-H	C10H18O	6.0985333333333	C11396	neg	199.13403892717	0.012237389172799	55.6	-
neg_11474	Paxilline	metab_42701	B(ii)	0	61.8	HMDB0030323	M-H2O-H	C27H33NO4	6.09325	C13782	neg	416.21909248512	0.029055679096448	47	57186-25-1
neg_11492	Dactolisib	metab_42719	B(ii)	0	43.5	HMDB0247649	M+Na-2H	C30H23N5O	6.0823166666667	-	neg	490.16209978654	0.038651220870659	45.8	-
neg_11493	4-(Tert-Butyl)-N-{4-[3,5-Bis(Tert-Butyl)-1H-Pyrazol-1-Yl]Phenyl}Benzamide	metab_42720	B(i)	37.8	0	-	M+Cl	C28H37N3O	6.0823166666667	-	neg	466.26584991644	0.0041062485237258	43.3	-
neg_11494	Mg(5-Iso Pgf2Vi/0:0/0:0)	metab_42721	B(ii)	0	67	HMDB0260485	M+FA-H	C21H36O7	6.0823166666667	-	neg	445.24432907187	0.17682145029482	51	-
neg_11497	Xanthine Amine Congener	metab_42724	B(ii)	0	45.1	HMDB0259918	M-H2O-H	C21H28N6O4	6.0823166666667	-	neg	409.19986163659	0.007772718912206	46	-
neg_11506	Probenecid	metab_42733	B(i)	62.5	0	HMDB0015166	M-H	C13H19NO4S	6.0770333333333	C07372	neg	284.09299928893	0.052128095635629	48.6	57-66-9
neg_11507	Piliformic Acid	metab_42734	B(i)	94.3	0	-	M-H	C11H18O4	6.0770333333333	-	neg	213.11333511163	0.022958683453802	57.6	-
neg_11520	(S)-10,16-Dihydroxyhexadecanoic Acid	metab_42747	B(ii)	0	66.7	HMDB0037798	M+Hac-H	C16H32O4	6.06635	C08285	neg	347.24428920955	0.004641126046853	52.8	69232-67-3
neg_11525	Zizybeoside Ii	metab_42752	B(ii)	0	79.2	HMDB0034955	M+Hac-H	C25H38O16	6.0609833333333	C17565	neg	653.22528039845	0.16398214308473	52.1	-;81417-79-0
neg_11533	Met-Thr-Phe	metab_42760	B(i)	73.1	0	-	M+FA-H	C18H27N3O5S	6.0609833333333	-	neg	442.16220949998	0.18910175091253	50.6	-
neg_11556	Mono(2-Ethyl-5-Hydroxyhexyl)Adipate	metab_42783	B(i)	59.1	0	-	2M-H	C14H26O5	6.05035	-	neg	547.34898970517	0.18242684848499	50.6	-
neg_11564	Sebacic Acid	metab_42791	B(i)	91.5	0	HMDB0000792;LMFA01170006	M-H	C10H18O4	6.05035	C08277	neg	201.11334038113	0.027969631768886	57.3	111-20-6;
neg_11569	Urceolide	metab_42796	B(i)	40.3	0	-	M+FA-H	C21H34O11	6.0450666666667	-	neg	507.20813450158	0.028260247546584	47.7	-
neg_11571	Pyrazosulfuron-Ethyl	metab_42798	B(ii)	0	46.5	HMDB0256958	M+FA-H	C14H18N6O7S	6.0450666666667	C18444	neg	459.0935666	0.070332406936059	46.6	93697-74-6;_
neg_11575	(1S,2R,4R,8S)-P-Menthane-2,8,9-Triol 2-Glucoside	metab_42802	B(ii)	0	63.5	HMDB0039977	M-H	C16H30O8	6.0450666666667	-	neg	349.18643603479	0.062142801511945	51.4	378254-07-0
neg_11576	2,3-Dinor Prostaglandin E1	metab_42803	B(i)	63.1	0	-	M-H	C18H30O5	6.0450666666667	-	neg	325.2021226	0.016028056119784	51.2	-
neg_11578	3-Oxododecanoic Acid	metab_42805	B(ii)	0	44.5	HMDB0010727	M+Hac-H	C12H22O3	6.0450666666667	C02367	neg	273.17083752759	0.011430536437539	48.3	-;_
neg_11586	Cholylcysteine	metab_42813	B(ii)	0	79.2	HMDB0242372	M+FA-H	C27H45NO6S	6.0397166666667	-	neg	556.29920034583	0.028326626242809	51.5	-
neg_11595	Jaceosidin	metab_42822	B(i)	95.6	0	LMPK12111235	M-H	C17H14O7	6.0397166666667	-	neg	329.06692052074	0.018903950035962	58.1	0
neg_11606	Dhesn	metab_42833	B(ii)	0	47	HMDB0251109	M+FA-H	C30H39N5O5	6.0344166666667	-	neg	594.29383034083	0.10386835280109	47.6	-
neg_11612	Lysylarginine	metab_42839	B(ii)	0	49.3	HMDB0028945	M+Hac-H	C12H26N6O3	6.0344166666667	-	neg	361.22335460148	0.014036343216888	46.2	29586-66-1
neg_11613	Apo-12'-Zeaxanthinal	metab_42840	B(ii)	0	51	HMDB0302023	M-H2O-H	C25H34O2	6.0344166666667	-	neg	347.23469367992	0.013319750920368	44	-
neg_11614	8-Hydroxy-4(6)-Lactarene-5,14-Diol	metab_42841	B(ii)	0	47.7	HMDB0035780	M+FA-H	C15H26O3	6.0344166666667	-	neg	299.18650602389	0.013155644577977	48.9	-
neg_11616	Momorcharaside B	metab_42843	B(ii)	0	50.5	HMDB0032941	M+Hac-H	C36H62O10	6.0291166666667	-	neg	713.44587450268	0.074216618580723	47.9	134886-64-9

neg_11634	3,5-Dihydroxydecanoic Acid	metab_42861	B(i)	85.3	0	-	M-H	C10H20O4	6.0291166666667	-	neg	203.12899375436	0.021558987197289	56.4	-
neg_11645	(S)-2-Methyl-1-Butanol O-Beta-D-Glucopyranoside	metab_42872	B(ii)	0	43.2	HMDB0303770	M+Na-2H	C11H22O6	6.0238166666667	-	neg	271.1165559	0.059970895503051	46.6	-
neg_11651	Ergocornine	metab_42878	B(ii)	0	80.2	HMDB0251906	M+Na-2H	C31H39N5O5	6.0184833333333	C09162;_	neg	582.27210715045	0.053973584875618	53	564-36-3;_
neg_11656	12-Hydroxyjasmonic Acid Glucoside	metab_42883	B(ii)	0	53.6	HMDB0301837	M+Na-2H	C18H28O9	6.0184833333333	C08558	neg	409.14869794839	0.029253922778662	49.4	-
neg_11667	Pinometostat	metab_42894	B(ii)	0	60	HMDB0256563	M-H	C30H42N8O3	6.0131333333333	-	neg	561.32958278344	0.08125395560332	49.3	-
neg_11671	Ligstroside-Aglycone	metab_42898	B(ii)	0	64.3	HMDB0301750	M+Hac-H	C19H22O7	6.0131333333333	-	neg	421.15074035799	0.03612698703388	51.7	-
neg_11676	2-Decenedioic Acid	metab_42903	B(i)	89.9	0	-	M-H	C10H16O4	6.0131333333333	-	neg	199.09766431359	0.012578324054621	56.5	-
neg_11683	Ibuprofen Glucuronide	metab_42910	B(ii)	0	43.2	HMDB0010343	M+Hac-H	C19H26O8	6.0078	C03033;_	neg	441.17677711071	0.056781438826375	48	98649-76-4;_
neg_11692	Nelfinavir	metab_42919	B(ii)	0	60.4	HMDB0014365	M+Cl	C32H45N3O4S	6.0024833333333	C07257	neg	602.28333597414	0.072407339616303	48.3	159989-64-7
neg_11703	Xi-8-Hydroxyhexadecanedioic Acid	metab_42930	B(ii)	0	51.6	HMDB0037831	M-H	C16H30O5	6.0024833333333	-	neg	301.2021992	0.01851037135591	49.5	-
neg_11705	2-(3-Hexyl-4-Methyl-2,5-Dioxopyrrol-1-Yl)-3-Hydroxybutanoic	metab_42932	B(i)	41.9	0	-	M-H2O-H	C15H23NO5	6.0024833333333	-	neg	278.13991852317	0.023668635649155	47.3	-
neg_11720	N-Succinyl-L,L-2,6-Diaminopimelate	metab_42947	B(ii)	0	54.6	HMDB0012267	M-H	C11H18N2O7	5.9971666666667	C04421	neg	289.10583158926	0.018460042257216	48.9	26605-36-7
neg_11724	Gliadorphin	metab_42951	B(ii)	0	72.3	HMDB0059786	M+FA-H	C43H57N9O11	5.9918666666667	-	neg	920.4161824	0.050217115198179	53.7	-
neg_11730	2,3-Dihydroxypropyl Dodecanoate	metab_42957	B(ii)	0	57	HMDB0245396	M+FA-H	C15H30O4	5.9918666666667	-	neg	319.21274587385	0.011592924336672	50.5	-
neg_11746	Sativic Acid	metab_42973	B(ii)	0	75	HMDB0302836	M-H	C18H36O6	5.9865666666667	-	neg	347.244103	0.02214319607849	54.3	_;
neg_11749	2-(6-Hydroxyhexyl)-3-Methylenesuccinic Acid	metab_42976	B(i)	83.7	0	-	M-H	C11H18O5	5.9865666666667	-	neg	229.1082726	0.026530767961391	55.6	-
neg_11758	6"-Malonylgenistin	metab_42985	B(ii)	0	58.6	HMDB0029529	M-H	C24H22O13	5.9812666666667	C16192	neg	517.09903117767	0.065116906235828	50.2	51011-05-3
neg_11760	Phenylalanyl-Prolyl-Arginine	metab_42987	B(ii)	0	57.5	HMDB0253027	M+Na-2H	C20H30N6O4	5.9812666666667	-	neg	439.20873762979	0.040360003489739	50.1	-
neg_11763	Pgp(Pge1/I-18:0)	metab_42990	B(ii)	0	72.6	HMDB0275500	M+Hac-H	C44H82O16P2	5.9759166666667	-	neg	987.53067246961	0.099400851930164	50.8	-
neg_11785	3-Hydroxybenzoic Acid	metab_43012	B(i)	98.4	0	HMDB0002466	M-H	C7H6O3	5.9759166666667	C00587	neg	137.02453887107	0.009410340766684 5	59.2	1999/6/9;99-06-9
neg_11791	Sl(11:0_O/18:4)	metab_43018	B(i)	60.3	0	-	M+FA-H	C29H51NO5S	5.9706333333333	-	neg	570.35154763472	0.19288382792703	47.2	-
neg_11792	Musabalbisiane C	metab_43019	B(ii)	0	62	HMDB0038682	M-H2O-H	C28H40O12	5.9706333333333	-	neg	549.23518573389	0.18708258777037	46.5	143183-60-2
neg_11795	Estriol-16-Glucuronide	metab_43022	B(ii)	0	59.9	HMDB0006766	M+FA-H	C24H32O9	5.9706333333333	C05504	neg	509.20411480361	0.092582770775933	49.9	1852-50-2
neg_11798	Devazepide	metab_43025	B(ii)	0	45.8	HMDB0251074	M+FA-H	C26H36N2O3	5.9706333333333	C11710;C13763	neg	469.26669236846	0.052328820454699	45.1	103420-77-5;_
neg_11800	Trp-Glu-Leu	metab_43027	B(i)	51.2	0	-	M-H2O-H	C22H30N4O6	5.9706333333333	-	neg	427.19828951925	0.054790458222469	48.9	-
neg_11813	Trans-Zeatin-O-Glucoside Riboside	metab_43040	B(ii)	0	79	HMDB0032880	M-H	C21H31N5O10	5.9652666666667	-	neg	512.20390170929	0.060999052794362	53.4	62512-97-4
neg_11824	Aclacinomycin S	metab_43051	B(ii)	0	70.3	HMDB0247947	M-H	C36H45NO13	5.9599166666667	C18635;_	neg	698.27950361764	0.23504372828781	49.6	-;_
neg_11830	Lys-Gly-His	metab_43057	B(i)	58.6	0	-	M+FA-H	C14H24N6O4	5.9599166666667	-	neg	385.18454012744	0.032235719455928	49.9	-
neg_11833	Menthyl Pyrrolidone Carboxylate	metab_43060	B(ii)	0	49.9	HMDB0032368	M-H2O-H	C16H30O6	5.9599166666667	C03962	neg	299.18653904716	0.01778305838899	49.1	52528-10-6
neg_11835	Dodecanedioic Acid	metab_43062	B(i)	47.8	0	HMDB0000623;LMFA01170009	M-H	C12H22O4	5.9599166666667	C02678	neg	229.14463556293	0.005550505524614	48.8	693-23-2;
neg_11860	Atracurium	metab_43087	B(ii)	0	64.3	HMDB0014870	M+FA-H	C53H72N2O12 +2	5.9492333333333	C07548	neg	973.5156213	0.026406200003553	48.5	64228-79-1
neg_11874	16-Hydroxy-10-Oxohexadecanoic Acid	metab_43101	B(ii)	0	57.7	HMDB0041287	M+Hac-H	C16H30O4	5.9492333333333	-	neg	345.22834812914	0.022317314312677	50.9	53833-25-3
neg_11876	10-Hydroxydecanoic Acid	metab_43103	B(i)	62.1	0	HMDB0244272	M+Hac-H	C10H20O3	5.9492333333333	C02774	neg	247.15523048523	0.016224360120181	52.1	1679-53-4;_
neg_11895	(E)-4-Hydroxydodec-2-Enedioic Acid	metab_43122	B(i)	93.6	0	-	M-H	C12H20O5	5.94395	-	neg	243.12390016441	0.016159979707792	58	-
neg_11897	Casomorphin	metab_43124	B(ii)	0	67.7	HMDB0059787	M+Hac-H	C44H61N7O11	5.9386	-	neg	922.45712531337	0.010893005255139	52.9	-
neg_11908	2"-Methoxy-(S)-Oleuropein	metab_43135	B(ii)	0	85.3	HMDB0035445	M+Na-2H	C26H34O14	5.9386	-	neg	591.16970919188	0.03303393306956	56.5	256498-10-9
neg_11919	Val-Pro-Tyr	metab_43146	B(i)	70	0	-	M-H	C19H27N3O5	5.9386	-	neg	376.18522969609	0.026539180739641	48.6	-
neg_11920	Glu-Ile-Ile	metab_43147	B(i)	92.6	0	-	M-H2O-H	C17H31N3O6	5.9386	-	neg	354.20356119374	0.008586387599362	57.5	-
neg_11923	(S)-2-Acetamido-3-(4-Chlorophenyl)Propanoic Acid	metab_43150	B(ii)	0	59.1	HMDB0243712	M-H	C11H12ClNO3	5.9386	-	neg	240.04343303205	0.018086083348965	46.4	-
neg_11946	2,5-Octadien-1-Ol	metab_43173	B(ii)	0	45.5	HMDB0040146	M+FA-H	C8H14O	5.93325	-	neg	171.10277376649	0.04198878393523	47.9	83861-75-0
neg_11967	Lyciumoside Ix	metab_43194	B(ii)	0	61.7	HMDB0033502	M+FA-H	C35H56O15	5.9223	-	neg	761.36380852928	0.038618709833259	48.4	212773-48-3
neg_12000	Citalopram	metab_43227	B(ii)	0	46.3	HMDB0005038	2M-H	C20H21FN2O	5.9169666666667	C07572	neg	647.31973546646	0.031974018850933	46.9	59729-33-8
neg_12004	1-[(4-Amino-3-Methylphenyl)Methyl]-5-(2,2-Diphenylacetyl)-6,7-Dihydro-4H-Imidazo[4,5-C]Pyridine-6-Carboxylic Acid	metab_43231	B(ii)	0	45.3	HMDB0260240	M+Hac-H	C29H28N4O3	5.9169666666667	C15552	neg	539.2300676	0.030425633945871	48.6	-
neg_12033	Vignatic Acid A	metab_43260	B(ii)	0	65.8	HMDB0033599	M-H	C30H39N3O7	5.9116166666667	-	neg	552.26842527726	0.041764812831972	50.2	181485-19-8

neg_12034	Epothilone B	metab_43261	B(ii)	0	85.9	HMDB0251874	M-H	C27H41NO6S	5.9116166666667	C12154;_	neg	506.26254775234	0.015200111900361	50.9	152044-54-
neg_12036	Malvidin 3-Rhamnoside	metab_43263	B(ii)	0	46.1	HMDB0303639	M-H2O-H	C23H25O11+	5.9116166666667	-	neg	458.1206405	0.12215961318966	47	-
neg_12039	Adenosine, 8-(Butylamino)-N-Cyclopentyl-	metab_43266	B(ii)	0	58.2	HMDB0258096	M-H	C19H30N6O4	5.9116166666667	-	neg	405.2245977	0.017781523123974	50	-
neg_12064	9-Oxononanoic Acid	metab_43291	B(i)	56.6	0	HMDB0094711	M+Hac-H	C9H16O3	5.9062833333333	C16322	neg	231.12391531935	0.03791363087507	51	2553-17-5;_
neg_12084	Musabalbisiane B	metab_43311	B(ii)	0	68.1	HMDB0038681	M+Na-2H	C23H30O12	5.9009833333333	-	neg	519.14651049572	0.11697055062019	50.8	143199-58-0
neg_12085	Kanokoside A	metab_43312	B(ii)	0	62.9	HMDB0035635	M+Na-2H	C21H32O12	5.9009833333333	C09993;C17428	neg	497.16434503991	0.076912104686796	51	-;64703-85-1
neg_12095	Pgp(Lte4/A-17:0)	metab_43322	B(ii)	0	70.5	HMDB0274508	M-H	C46H83NO16P 2S	5.8956166666667	-	neg	998.48491581929	0.039775244558705	50.9	-
neg_12118	Iralukast	metab_43345	B(ii)	0	51.2	HMDB0253578	M+Hac-H	C38H37F3O8S	5.8902666666667	-	neg	769.23532828361	0.053323858047632	46.6	-
neg_12137	3-Hydroxytridecanoic Acid	metab_43364	B(ii)	0	50.4	HMDB0061655	M+FA-H	C13H26O3	5.8902666666667	-	neg	275.18652947007	0.014489568893643	49.3	-
neg_12142	Ps(6 Keto-Pgf1Alpha/18:4(6Z,9Z,12Z,15Z))	metab_43369	B(ii)	0	43.8	HMDB0281970	M+FA-H	C44H72NO14P	5.8849666666667	-	neg	914.46906286999	0.055767674325536	41.9	-
neg_12149	Hydroxyzine	metab_43376	B(ii)	0	40	HMDB0014697	2M-H	C21H27ClN2O	5.8849666666667	C07045	neg	747.34892566093	0.065749314767753	40	68-88-2
neg_12164	Ancymidol	metab_43391	B(ii)	0	40.7	HMDB0248390	M+FA-H	C15H16N2O2	5.8849666666667	C18774	neg	301.11951094068	0.054994674553893	47.6	12771-68-5;_
neg_12196	Midecamycin Acetate	metab_43423	B(ii)	0	40.7	HMDB0254742	M-H2O-H	C45H71NO17	5.8743	-	neg	878.44782630367	0.018887442500059	44.4	-
neg_12199	Cis-Zeatin-7-N-Glucoside	metab_43426	B(ii)	0	60.4	HMDB0012201	2M+FA-H	C16H23N5O6	5.8743	-	neg	807.33227164359	0.012940776623015	46.1	823188-69-8
neg_12211	Cucumopine	metab_43438	B(ii)	0	40.6	HMDB0301842	2M-H	C11H13N3O6	5.8743	C08475	neg	565.15662898079	0.095636839222997	44.6	110342-24-0;_
neg_12213	7-Sulfocholic Acid	metab_43440	B(ii)	0	49.4	HMDB0002421	M-H	C24H40O8S	5.8743	-	neg	487.24108922717	0.067669213675195	45.8	60320-05-0;
neg_12243	Phe-Ile-Leu	metab_43470	B(i)	60.6	0	-	M-H	C21H33N3O4	5.8689333333333	-	neg	390.2399641	0.008656027985947	50.6	-
neg_12244	Dihydrokaempferol	metab_43471	B(i)	83.8	0	HMDB0030847;LMPK12140720	M-H	C15H12O6	5.8689333333333	C00974	neg	287.0563024	0.090955488343496	54.9	480-20-6;
neg_12281	Endoxifen O-Glucuronide	metab_43508	B(ii)	0	49.9	HMDB0060622	M-H	C31H37NO8	5.8582666666667	-	neg	550.2440139	0.030615833756506	47.1	-
neg_12303	Trifolin	metab_43530	B(i)	83.2	0	HMDB0030864;HMDB0257804;LMPK12111663	M-H	C21H20O11	5.8529166666667	_;C12626	neg	447.09344987631	0.043845393081652	55.1	23627-87-4;_;
neg_12306	Ile-Leu-Ile	metab_43533	B(i)	79.8	0	-	M-H	C18H35N3O4	5.8529166666667	-	neg	356.25559351959	0.046561323947155	54.5	-
neg_12312	[Bala8]-Neurokinin A(4-10)	metab_43539	B(ii)	0	88.7	HMDB0244469	M-H2O-H	C35H56N8O10	5.8475666666667	-	neg	761.36317609732	0.009757093875202	54.9	-
neg_12318	Felotaxel	metab_43545	B(ii)	0	80.8	HMDB0252189	M-H	C31H32N2O6	5.8475666666667	-	neg	527.21366209231	0.027383520860348	53.1	-
neg_12321	Mono(2-Ethyl-5-Hydroxyhexyl)Phthalate	metab_43548	B(i)	49.8	0	HMDB0094679	M+FA-H	C16H22O5	5.8475666666667	-	neg	339.14262760532	0.035637796178052	46.6	-
neg_12325	Pgp(5-Iso Pgf2Vi/I-15:0)	metab_43552	B(ii)	0	42	HMDB0275142	M+FA-H	C39H72O16P2	5.8422166666667	-	neg	903.43490731335	0.17148781035308	42	-
neg_12330	S-(9-Deoxy-Delta12-Pgd2)-Glutathione	metab_43557	B(ii)	0	41.1	HMDB0013057	M-H	C30H49N3O10	5.8422166666667	-	neg	642.31277741114	0.050435460026229	43.9	-
neg_12347	Protoporphyrinogen Ix	metab_43574	B(ii)	0	79.1	HMDB0001097	M-H2O-H	C34H40N4O4	5.8369	C01079	neg	549.2914672	0.10682560763125	47.8	7412-77-3
neg_12348	Ribostamycin	metab_43575	B(ii)	0	73	HMDB0257216	M+Cl	C17H34N4O10	5.8369	C01759;_	neg	489.19926742098	0.14183929318122	52.8	25546-65-0;_
neg_12370	Epothilone A	metab_43597	B(ii)	0	88.4	HMDB0251873	M-H	C26H39NO6S	5.8316	C12153;_	neg	492.24696012005	0.042842237019605	52.2	152044-53-
neg_12394	9-(2,3-Dihydroxypropoxy)-9-Oxononanoic Acid	metab_43621	B(i)	92.3	0	-	M-H	C12H22O6	5.8263	-	neg	261.13450803208	0.021582087778964	57.2	-
neg_12404	Protoporphyrin Ix	metab_43631	B(ii)	0	46	HMDB0000241	M+Cl	C34H34N4O4	5.8209666666667	C02191	neg	597.23219439006	0.049152658740141	45	553-12-8
neg_12408	Osimertinib	metab_43635	B(ii)	0	47.8	HMDB0248778	M+Cl	C28H33N7O2	5.8209666666667	-	neg	534.23609527347	0.012244619574547	48	-
neg_12410	Lucidenic Acid A	metab_43637	B(ii)	0	58.8	HMDB0037611	M+Cl	C27H38O6	5.8209666666667	-	neg	493.2320653	0.051965298580353	46.8	95311-94-7
neg_12411	Gln-Phe-Leu	metab_43638	B(i)	51.4	0	-	M+Hac-H	C20H30N4O5	5.8209666666667	-	neg	465.23522198655	0.023689491399546	47.5	-
neg_12415	Isophorone Diisocyanate	metab_43642	B(ii)	0	44.9	HMDB0253670	M+FA-H	C12H18N2O2	5.8209666666667	-	neg	267.13509843504	0.018677005468296	46.2	-
neg_12426	Plazomicin	metab_43653	B(ii)	0	47.4	HMDB0256636	M-H2O-H	C25H48N6O10	5.8156333333333	-	neg	573.32590974031	0.034489256218535	43.8	-
neg_12436	Smilanippin A	metab_43663	B(ii)	0	55.8	HMDB0041354	M+Cl	C39H64O12	5.8103333333333	-	neg	759.40506797426	0.03530025183126	46.6	166736-13-6
neg_12453	Isoferulic Acid	metab_43680	B(i)	73.3	0	HMDB0000955;HMDB0259693	M-H	C10H10O4	5.805	_;C10470	neg	193.05070960225	0.10477191343368	52.7	537-73-5;25522-33-
neg_12458	Licorice Glycoside A	metab_43685	B(ii)	0	47	HMDB0031989	M+Hac-H	C36H38O16	5.7996333333333	-	neg	785.23028821464	0.16077252610946	47.3	-
neg_12460	Janthitrem G	metab_43687	B(ii)	0	65	HMDB0030531	M-H	C39H51NO6	5.7996333333333	-	neg	628.36764195007	0.048768054001156	46.5	90986-51-9
neg_12496	Milbemycin Alpha10	metab_43723	B(ii)	0	53.7	HMDB0254722	M+Cl	C37H49NO9	5.7836	-	neg	686.31591580125	0.014028770703066	47.2	-
neg_12502	Phe-Phe-Asp	metab_43729	B(i)	43.9	0	-	M-H	C22H25N3O6	5.7836	-	neg	426.16723749988	0.037695236963257	47.7	-
neg_12512	Xi-3,5-Dimethyl-2(5H)-Furanone	metab_43739	B(ii)	0	42.7	HMDB0039778	M+Hac-H	C6H8O2	5.77825	-	neg	171.06641695468	0.044673744117267	47.2	-
neg_12517	Goshonoside F4	metab_43744	B(ii)	0	50.7	HMDB0038377	M-H	C32H54O12	5.7729166666667	-	neg	629.35141897911	0.051800386704318	45	90851-27-7

neg_12520	Taraxacoside	metab_43747	B(ii)	0	72.7	HMDB0030055	M+Hac-H	C18H22O10	5.7729166666667	-	neg	457.13661789581	0.011623688735245	53.1	98449-40-2
neg_12525	Canarigenin 3-[Glucosyl-(1->4)-6-Deoxy-Alloside]	metab_43752	B(ii)	0	45.3	HMDB0035433	M-H2O-H	C35H52O13	5.7676166666667	-	neg	661.32656904345	0.11369216707617	44.6	229319-08-8
neg_12532	Bufotenine O-Glucoside	metab_43759	B(ii)	0	54.4	HMDB0029564	M+Hac-H	C18H26N2O6	5.7676166666667	-	neg	425.19330928304	0.070431429431837	48.9	64656-15-1
neg_12550	Pip(6 Keto-Pgf1Alpha/16:1(9Z))	metab_43777	B(ii)	0	45.5	HMDB0278548	M-H2O-H	C45H80O20P2	5.7567	-	neg	983.44442990801	0.037665951854788	41.2	-
neg_12570	Val-Gly-Val-Ala-Pro-Gly	metab_43797	B(ii)	0	79.7	HMDB0253033	M+FA-H	C22H38N6O7	5.7514	-	neg	543.27845671002	0.048965019452055	53.6	-
neg_12581	Isopentenyl Adenosine	metab_43808	B(ii)	0	42.4	HMDB0304396	M+FA-H	C15H21N5O4	5.7460666666667	C16427	neg	380.15770072246	0.024384354997444	46.1	7724-76-7;_
neg_12582	2,4,6-Trimethylbenzenesulfonic Acid	metab_43809	B(i)	43.4	0	-	M+Hac-H	C9H12O3S	5.7460666666667	-	neg	259.06317655637	0.057364279288365	46	-
neg_12586	Avenestergenin A1	metab_43813	B(ii)	0	43.6	HMDB0035264	M+Na-2H	C38H55NO7	5.7407666666667	-	neg	658.37839942194	0.19733003094585	43.4	90578-35-1
neg_12589	His-Tyr-Phe	metab_43816	B(i)	44	0	-	M-H	C24H27N5O5	5.7407666666667	-	neg	464.19353645517	0.023244612945474	46.7	-
neg_12592	Rosamicin	metab_43819	B(ii)	0	78.9	HMDB0257306	M+FA-H	C31H51NO9	5.7354666666667	-	neg	626.35261309047	0.12000316582809	52.4	-
neg_12609	Tyr-Lys-Ile	metab_43836	B(i)	78.2	0	-	M+FA-H	C21H34N4O5	5.7247333333333	-	neg	467.24833023483	0.13782866333599	51.5	-
neg_12616	L-1,2,3,4-Tetrahydro-Beta-Carboline-3-Carboxylic Acid	metab_43843	B(ii)	0	41.8	HMDB0035665	M+Hac-H	C12H12N2O2	5.7194	-	neg	275.10389791823	0.0349478992357	47.9	42438-90-4
neg_12617	1,2-Cyclohexanediol, 1-Methyl-4-(1-Methylethenyl)-	metab_43844	B(ii)	0	71.7	HMDB0256048	M+FA-H	C10H18O2	5.7194	C18020	neg	215.12898514151	0.009793844951870	53.5	-
neg_12624	Glu-Val-Phe	metab_43851	B(i)	61.2	0	-	M-H2O-H	C19H27N3O6	5.7141	-	neg	374.1723322	0.076845617752283	50.6	-
neg_12640	Etamucine	metab_43867	B(ii)	0	67.6	HMDB0251987	M+Cl	C33H54N2O23	5.6981333333333	-	neg	881.27843476204	0.058733565550451	49.4	-
neg_12659	Phe-Thr-Ile	metab_43886	B(i)	83.1	0	-	M-H	C19H29N3O5	5.6928333333333	-	neg	378.20379570643	0.29425008740782	53.9	-
neg_12660	Deca-3,5,7-Trienedioylcarnitine	metab_43887	B(ii)	0	48.7	HMDB0241129	M-H	C17H25NO6	5.6928333333333	-	neg	338.16104774253	0.16532219470825	47.8	-
neg_12662	Z-Asp-Glu-Val-Asp-Fluoromethylketone	metab_43889	B(ii)	0	43.2	HMDB0259962	M-H2O-H	C27H35FN4O1	5.6875	-	neg	607.20327297041	0.062276798348261	46.3	-
neg_12670	Cerebrocrast	metab_43897	B(ii)	0	40.4	HMDB0249819	M+FA-H	C26H35F2NO7	5.6821666666667	-	neg	556.23971077017	0.022972980780125	45.5	-
neg_12672	Melagatran	metab_43899	B(ii)	0	55.4	HMDB0254416	M+Hac-H	C22H31N5O4	5.6821666666667	C21551;_	neg	488.2517399	0.006698635425303	50.9	159776-70-2;_
neg_12684	Swertiajaponin	metab_43911	B(ii)	0	56.6	HMDB0030569	M-H2O-H	C22H22O11	5.6662	C10187;_	neg	443.10181382784	0.005564981796784	49	6980-25-2;
neg_12689	Physagulin D	metab_43916	B(ii)	0	58.4	HMDB0041049	M-H2O-H	C34H52O10	5.6609	-	neg	601.33608678612	0.012025410037777	48.7	146713-92-0;
neg_12693	Epitheaflagallin 3-O-Gallate	metab_43920	B(ii)	0	43.6	HMDB0033288	M-H2O-H	C27H20O13	5.6609	-	neg	533.07124655991	0.026123624346567	45.9	102067-92-5
neg_12696	Cilligen	metab_43923	B(ii)	0	41.3	HMDB0256235	M+Cl	C22H30N4O5S	5.6609	-	neg	497.16427296438	0.031835437309907	43.4	-
neg_12697	Oxoamide	metab_43924	B(ii)	0	55.4	HMDB0001004	2M+FA-H	C10H12N2O2	5.6609	-	neg	429.17700216008	0.005645988036862	49.2	713-05-3
neg_12698	Tragopogonsaponin M	metab_43925	B(ii)	0	62.7	HMDB0037929	M-H	C51H76O16	5.6555666666667	-	neg	943.51080249594	0.10390723326373	46.1	-
neg_12702	10-Hydroxyligstroside	metab_43929	B(i)	94.1	0	-	M-H	C25H32O13	5.6555666666667	-	neg	539.17721237697	0.032689289340226	57.7	-
neg_12703	Semustine	metab_43930	B(ii)	0	80.6	HMDB0258228	2M-H	C10H18ClN3O	5.6555666666667	C07640	neg	493.20582526761	0.11225031187444	49	13909-09-6;_
neg_12704	Phe-Phe-Ser	metab_43931	B(i)	75.6	0	-	M-H	C21H25N3O5	5.6555666666667	-	neg	398.17232843422	0.10963602902384	52.7	-
neg_12710	Jubanine A	metab_43937	B(ii)	0	84	HMDB0030205	M+Cl	C40H49N5O6	5.6502333333333	-	neg	730.33167157995	0.011648450994227	53.2	60375-07-7
neg_12713	3',5'-Di-C-Glucosylphloretin	metab_43940	B(i)	72.3	0	-	M-H2O-H	C27H34O15	5.6502333333333	-	neg	579.17217341959	0.06338927252703	52.2	-
neg_12716	5-Amino-3-(4-Methoxyphenyl)-5-Oxopentanoic Acid	metab_43943	B(i)	39.6	0	-	2M-H	C12H15NO4	5.6502333333333	-	neg	473.19426414411	0.14859215447696	46.9	-
neg_12719	Voriconazole	metab_43946	B(ii)	0	42.3	HMDB0014720	M-H	C16H14F3N5O	5.6502333333333	C07622	neg	348.10663235719	0.12707823830538	45.5	137234-62-9
neg_12732	Mppa (Methyl Pyropheophorbide-A)	metab_43959	B(ii)	0	59.9	HMDB0254917	M+Hac-H	C34H36N4O3	5.63955	-	neg	607.28875051819	0.045151896807204	47.8	-
neg_12734	Glu-Tyr-Leu	metab_43961	B(i)	80.8	0	-	M-H2O-H	C20H29N3O7	5.63955	-	neg	404.18289737348	0.1031326170178	53	-
neg_12741	Taraxacolide 1-O-B-D-Glucopyranoside	metab_43968	B(ii)	0	59	HMDB0035610	2M+FA-H	C21H32O9	5.6289166666667	-	neg	901.40582985772	0.12538863870625	48.6	75911-12-5
neg_12759	Gentiobiosyl 2-Methyl-6-Oxo-2E,4E-Heptadienoate	metab_43986	B(ii)	0	51	HMDB0032134	M-H	C20H30O13	5.6236166666667	-	neg	477.16041008851	0.08668927578667	47.3	-
neg_12772	Pa(6 Keto-Pgf1Alpha/19:2(10Z,13Z))	metab_43999	B(ii)	0	81.9	HMDB0264541	M+Cl	C42H73O12P	5.6130166666667	-	neg	835.44746070186	0.014698618907114	53.6	-
neg_12773	Kaempfetrin	metab_44000	B(i)	82.5	0	-	M-H	C33H40O20	5.6130166666667	-	neg	755.20419314779	0.02639965351894	55.2	-
neg_12792	Ser-Trp-Ile	metab_44019	B(i)	61.6	0	-	M-H	C20H28N4O5	5.6023666666667	-	neg	403.19876254484	0.082921037863218	50.3	-
neg_12811	Pi(O-12:0/26:7)	metab_44038	B(i)	96.1	0	-	M+FA-H	C47H79O12P	5.5970166666667	-	neg	911.53232982891	0.13855702097419	56	-
neg_12835	Pa(5-Iso Pgf2Vi/8:0)	metab_44062	B(ii)	0	50.6	HMDB0266632	M+FA-H	C29H51O11P	5.5808166666667	-	neg	651.31933826461	0.032656949668169	47.9	-
neg_12842	Biopterin	metab_44069	B(ii)	0	40.4	HMDB0000468	M+Cl	C9H11N5O3	5.5808166666667	C06313	neg	272.05414864551	0.076177451294066	45.7	22150-76-1
neg_12855	Cdp-Dg(5-Iso Pgf2Vi/A-13:0)	metab_44082	B(ii)	0	62.7	HMDB0292784	M-H2O-H	C43H73N3O18P2	5.5648666666667	-	neg	962.42444890862	0.0747053542485	48.6	-
neg_12860	Phe-Ser-Ile	metab_44087	B(i)	82.6	0	-	M-H	C18H27N3O5	5.5648666666667	-	neg	364.18794710663	0.004592219357501	55.1	-

neg_12864	Lisinopril, Epsilon-Biotinamidocaproyl-	metab_44091	B(ii)	0	48	HMDB0244524	M+Na-2H	C37H56N6O8S	5.55955	-	neg	765.36629599154	0.073346980664613	45.2	-
neg_12867	(23S)-23,25-Dihdroxy-24-Oxovitamine D3 23-(Beta-	metab_44094	B(ii)	0	42.8	HMDB0010361	M+Cl	C33H50O10	5.55955	-	neg	641.30542427079	0.035982844623186	45.6	-
neg_12869	Dodecenoylcarnitine	metab_44096	B(ii)	0	49.6	HMDB0251567	M+Cl	C19H35NO4	5.55955	-	neg	376.2248944	0.079625482476076	47.5	-
neg_12878	Asp-Trp-Leu	metab_44105	B(i)	92.5	0	-	M-H	C21H28N4O6	5.55425	-	neg	431.19418801436	0.015296190352951	54	-
neg_12879	21-Desacetyl Deflazacort	metab_44106	B(ii)	0	66.1	HMDB0245604	M+Na-2H	C23H29NO5	5.55425	-	neg	420.17780291911	0.023072456974923	50.2	-
neg_12885	Ganosporeric Acid A	metab_44112	B(ii)	0	42	HMDB0033022	M+FA-H	C30H38O8	5.54895	C16526;_	neg	571.25256104149	0.020475247187082	46.1	135357-25-4;
neg_12890	Pa(Pge1/I-14:0)	metab_44117	B(ii)	0	85.4	HMDB0267612	M+Cl	C37H67O11P	5.5436333333333	-	neg	753.41546887541	0.04581796812443	54.4	-
neg_12895	Asn-Phe-Ile	metab_44122	B(i)	80	0	-	M-H	C19H28N4O5	5.5436333333333	-	neg	391.19806351023	0.006231301319986	53.9	-
neg_12900	Cymarine	metab_44127	B(i)	57.7	0	-	M+FA-H	C30H44O9	5.5382833333333	-	neg	593.29171436038	0.096127741774353	45.8	-
neg_12905	Indole-3-Acetylglutamic Acid	metab_44132	B(ii)	0	45.7	HMDB0038665	2M-H	C15H16N2O5	5.53295	-	neg	607.20342431713	0.009131541309047	46	57105-48-3
neg_12917	Eujambolin	metab_44144	B(ii)	0	42.8	HMDB0037970	M-H2O-H	C24H24O13	5.5276166666667	-	neg	501.1074097	0.012537169164608	45.4	-
neg_12933	Tetraxetan	metab_44160	B(ii)	0	62.8	HMDB0251592	M+Na-2H	C16H28N4O8	5.5169666666667	-	neg	425.16554130095	0.11571532721543	50.2	-
neg_12935	Libenzapril	metab_44162	B(ii)	0	56.4	HMDB0254077	M-H	C18H25N3O5	5.5169666666667	-	neg	362.16981488738	0.015115632633917	48.8	-
neg_12936	Glu-Val-Leu	metab_44163	B(i)	92.4	0	-	M-H2O-H	C16H29N3O6	5.5169666666667	-	neg	340.18782416184	0.003986044972804	57.6	-
neg_12949	Butirosina	metab_44176	B(ii)	0	51.5	HMDB0248282	M-H	C21H41N5O12	5.5060166666667	-	neg	554.27148630574	0.12837067475758	47	-
neg_12955	25-Acetyl-6,7-Didehydrofevicordin F 3-[Glucosyl-(1->6)-	metab_44182	B(ii)	0	61.1	HMDB0036339	M-H	C43H62O18	5.5007166666667	-	neg	865.39269097071	0.26333689858354	46	178062-91-4
neg_12964	Phe-Pro-Leu	metab_44191	B(i)	90.4	0	-	M-H	C20H29N3O4	5.5007166666667	-	neg	374.2085985	0.007631918290322	57.2	-
neg_12967	Euglobal Ia1	metab_44194	B(ii)	0	42.5	HMDB0030032	2M-H	C23H30O5	5.4953666666667	C09927	neg	771.40620898445	0.030518842961648	44.1	77844-93-0
neg_12969	Thapsigargin	metab_44196	B(ii)	0	66.5	HMDB0258957	M+Na-2H	C34H50O12	5.4953666666667	C09561;_	neg	671.30257243248	0.035254190763163	49.9	67526-95-8;_;
neg_12973	(E)-6-Hydroxy-2,7-Dimethyloct-2-Enedioic Acid	metab_44200	B(i)	83.5	0	-	M-H	C10H16O5	5.4953666666667	-	neg	215.09265024821	0.03848280758802	54.8	-
neg_12986	Ergovaline	metab_44213	B(ii)	0	47.8	HMDB0251917	M+Na-2H	C29H35N5O5	5.4847333333333	-	neg	554.2359783	0.032019302497635	46.7	-
neg_13002	(3S,5R,6S,7E,9X)-7-Megastigmene-3,6,9-Triol 9-Glucoside	metab_44229	B(ii)	0	63	HMDB0041176	M+Hac-H	C19H34O8	5.4740166666667	-	neg	449.24067425602	0.006176794383333	49.9	_;
neg_13004	Fluvastatin	metab_44231	B(ii)	0	70.2	HMDB0015227	2M-H	C24H26FNO4	5.4686833333333	C07014	neg	821.36931248669	0.004164899790635	49.5	93957-54-1
neg_13025	Alpha-Tetrasaccharide	metab_44252	B(ii)	0	70.6	HMDB0000445	M-H2O-H	C26H45NO20	5.4580833333333	C06768	neg	672.23019854791	0.084316573465739	51	59957-92-5
neg_13035	Parishin C	metab_44262	B(ii)	0	61.3	HMDB0256129	M-H2O-H	C32H40O19	5.4527333333333	_;C17466	neg	709.20053917674	0.2512905545793	49.4	-;_
neg_13041	Minoxidil Glucuronide	metab_44268	B(ii)	0	42.5	HMDB0254740	M-H	C15H23N5O7	5.4527333333333	-	neg	384.15420940277	0.009530882663991	46.6	-
neg_13055	Pendimethalin	metab_44282	B(ii)	0	44.8	HMDB0256221	2M+FA-H	C13H19N3O4	5.44205	C11019	neg	607.27104917573	0.055430752249791	45.9	40487-42-1;_
neg_13059	21-Deoxycortisol	metab_44286	B(ii)	0	47.8	HMDB0004030	M+FA-H	C21H30O4	5.44205	C05497;_	neg	391.20986087756	0.068190471361617	45.8	641-77-0;
neg_13066	Petromyzonol Sulfate	metab_44293	B(ii)	0	59.7	HMDB0256356	M+Hac-H	C24H42O7S	5.4367	-	neg	533.28305611291	0.029517245652216	48.1	-
neg_13067	His Thr Gln Glu	metab_44294	B(i)	52.9	0	-	M-H	C20H31N7O9	5.4367	-	neg	512.21570105433	0.001302553498649	48.2	-
neg_13074	1-(2-(4-(3-Phenyl-2H-1-Benzopyran-2-	metab_44301	B(ii)	0	44.5	HMDB0249745	M+Cl	C28H29NO2	5.43135	-	neg	446.19110840001	0.021891764155336	45.5	-
neg_13076	Leu-Thr-Phe	metab_44303	B(i)	95.2	0	-	M-H	C19H29N3O5	5.43135	-	neg	378.20354428303	0.02842455874451	58	-
neg_13078	Hgluvalleupnsaspalaglupheoh	metab_44305	B(ii)	0	82.9	HMDB0253807	M-H2O-H	C47H66N8O16	5.426	-	neg	979.43836360715	0.047064210411166	52.3	-
neg_13086	Ps(Pgf2Alpha/16:1(9Z))	metab_44313	B(ii)	0	84.8	HMDB0281245	M+Na-2H	C42H74NO13P	5.42065	-	neg	852.45846394134	0.052658211955605	54.1	-
neg_13093	Met-Glu-Leu	metab_44320	B(i)	60.5	0	-	M-H2O-H	C16H29N3O6S	5.42065	-	neg	372.15999525203	0.080197114584291	48.3	-
neg_13094	Asp-Ile-Ile	metab_44321	B(i)	96.1	0	-	M-H	C16H29N3O6	5.42065	-	neg	358.19846662747	0.026839378269051	57.7	-
neg_13105	Val-Leu-Leu	metab_44332	B(i)	74.1	0	-	M-H	C17H33N3O4	5.4153333333333	-	neg	342.23992139734	0.041256361238386	53.5	-
neg_13108	Linalool 3,7-Oxide Beta-Primeveroside	metab_44335	B(ii)	0	68.1	HMDB0036571	M+FA-H	C21H36O11	5.4100166666667	-	neg	509.22415070274	0.024770886256767	52.6	-
neg_13117	Scd1 Inhibitor	metab_44344	B(ii)	0	50.9	HMDB0244474	M-H	C21H20F3N3O	5.4046833333333	-	neg	418.13856860597	0.033327804588853	49.1	-
neg_13120	Adrenosterone	metab_44347	B(ii)	0	48.8	HMDB0006772	M+Na-2H	C19H24O3	5.4046833333333	C05285	neg	321.1457609	0.050164995188844	46.7	382-45-6
neg_13127	Actodigin	metab_44354	B(ii)	0	77.2	HMDB0247988	M+Hac-H	C29H44O9	5.39935	-	neg	595.31015391933	0.005283087812219	52.1	-
neg_13136	4-Coumaric Acid	metab_44363	B(i)	93.2	0	HMDB0030677	M-H	C9H8O3	5.39935	C06738	neg	163.04016598094	0.006232567881701	58.2	4501-31-9
neg_13138	Methyl Nomilinate 17-Glucoside	metab_44365	B(ii)	0	40.6	HMDB0039887	M+Hac-H	C35H50O16	5.39405	-	neg	785.32582409379	0.027524280103742	45.5	-
neg_13158	Indacaterol-8-O-Glucuronide	metab_44385	B(ii)	0	68.8	HMDB0061152	M+Hac-H	C30H36N2O9	5.3887	-	neg	627.25781967068	0.046556624073629	52.7	-
neg_13159	Ac-Pro-Gly-Pro-Oh	metab_44386	B(ii)	0	61.7	HMDB0245745	2M-H	C14H21N3O5	5.3887	-	neg	621.28916844912	0.0798957613049	51	-
neg_13161	Moexipril	metab_44388	B(ii)	0	41.4	HMDB0014829	M+Na-2H	C27H34N2O7	5.3887	C07704	neg	519.20755361459	0.034854917970197	43.6	103775-10-6

neg_13167	Phe-Leu-Ser	metab_44394	B(i)	62.6	0	-	M-H	C18H27N3O5	5.3887	-	neg	364.18789639984	0.017283291575717	51.5	-
neg_13170	Abacavir	metab_44397	B(ii)	0	49.8	HMDB0015182	M+Na-2H	C14H18N6O	5.3887	_;C07624	neg	307.13030562388	0.067588418645421	47.3	136470-78-5;_
neg_13181	6-Lactoyltetrahydropterin	metab_44408	B(ii)	0	43.1	HMDB0002065	M+Cl	C9H13N5O3	5.38335	C04244	neg	274.06984416591	0.005072628126471	46.8	33405-80-0
neg_13183	Docetaxel	metab_44410	B(ii)	0	46.8	HMDB0015378	M+Na-2H	C43H53NO14	5.3780333333333	C11231	neg	828.32930867107	0.11721839532347	44.6	114977-28-5
neg_13184	Abbott-195773	metab_44411	B(ii)	0	76.5	HMDB0249831	M-H	C42H59N3O10	5.3780333333333	_;C12020	neg	764.41010808239	0.062581798028961	51.8	205110-48-1;_
neg_13199	Hispiduloside	metab_44426	B(ii)	0	41.2	HMDB0302930	M-H2O-H	C22H22O11	5.3727333333333	C17762;_	neg	443.10185840723	0.040355369145548	45.3	-;_
neg_13207	Ala Leu Gly Ile Ser	metab_44434	B(i)	49.2	0	-	M-H	C20H37N5O7	5.3673833333333	-	neg	458.26214412478	0.0221119222814921	48.1	-
neg_13213	Manassantin A	metab_44440	B(ii)	0	69.8	HMDB0254321	M-H	C42H52O11	5.3621	-	neg	731.33724152327	0.032752550708817	42.4	-
neg_13217	Indolapril	metab_44444	B(ii)	0	40.7	HMDB0253467	M-H	C24H34N2O5	5.3621	-	neg	429.23595731513	0.037299114402412	43.6	-
neg_13218	Asn-Phe-Leu	metab_44445	B(i)	80.6	0	-	M-H	C19H28N4O5	5.3621	-	neg	391.19858705912	0.008783159712761	55.5	-
neg_13220	Sibrafiban	metab_44447	B(ii)	0	72.6	HMDB0258287	2M+FA-H	C20H28N4O6	5.3568	-	neg	885.39940299375	0.17609615352505	53	-
neg_13221	Pgp(Pgf1Alpha/I-13:0)	metab_44448	B(ii)	0	51.8	HMDB0274986	M-H2O-H	C39H74O16P2	5.3568	-	neg	841.42158478984	0.007700778600329	48	-
neg_13227	Fumitremorgin B	metab_44454	B(ii)	0	53.4	HMDB0030203	M-H	C27H33N3O5	5.3568	C20630;_	neg	478.2311134	0.008987190224265	47.5	12626-17-4
neg_13230	Gly-Val-Tyr	metab_44457	B(i)	50.2	0	-	M+FA-H	C16H23N3O5	5.3568	-	neg	382.1621131	0.065911548335102	49.1	-
neg_13240	Caprylic Acid	metab_44467	B(i)	58.8	0	HMDB0000482;LMFA01010008;PW_C000366	M+Hac-H	C8H16O2	5.3515	C06423	neg	203.12899260712	0.006172514946943 9	51.4	124-07-2;74-81-7;124-07-
neg_13257	Purpurogallin	metab_44484	B(ii)	0	40	HMDB0256929	M+Hac-H	C11H8O5	5.3409	C09964	neg	279.05119429955	0.013259825375538	47.4	569-77-7;_
neg_13266	Tetraethylene Glycol	metab_44493	B(ii)	0	58.2	HMDB0094708	2M-H	C8H18O5	5.33535	-	neg	387.22503842367	0.053046910671038	49.7	-
neg_13274	Endomorphin-2	metab_44501	B(ii)	0	40	HMDB0005774	M-H	C32H37N5O5	5.3300666666667	C15891;_	neg	570.26879943938	0.013976216922455	45.2	-;141801-26-5
neg_13283	3-Hydroxy-Beta-Ionol 3-[Glucosyl-(1->6)-Glucoside]	metab_44510	B(ii)	0	51	HMDB0037527	M+Hac-H	C25H42O12	5.3247	-	neg	593.28313998602	0.085705034593787	47.7	137606-56-5
neg_13301	Phe-Lys-Thr	metab_44528	B(i)	46.3	0	-	M+FA-H	C19H30N4O5	5.3141	-	neg	439.21739793309	0.009012219297730	47.1	-
neg_13307	Leucomycin	metab_44534	B(ii)	0	85.3	HMDB0254035	M-H2O-H	C35H59NO13	5.30875	C14064;_	neg	682.37820147128	0.04042196194283	53.1	1392-21-8;_
neg_13323	Ile-Asp-Leu	metab_44550	B(i)	42.5	0	-	M-H2O-H	C16H29N3O6	5.29815	-	neg	340.18791600215	0.074848722370511	46.6	-
neg_13332	Pilocarpine	metab_44559	B(ii)	0	52.2	HMDB0015217	2M+FA-H	C11H16N2O2	5.2928166666667	C07474	neg	461.24090700353	0.010035611880121	47.8	92-13-7;54-71-7
neg_13349	Gly-Leu-Leu	metab_44576	B(i)	96.6	0	-	M-H	C14H27N3O4	5.28745	-	neg	300.19300315467	0.008378074476302	58.4	-
neg_13350	Sinapic Acid	metab_44577	B(i)	96.8	0	HMDB0032616	M-H	C11H12O5	5.28745	C00482	neg	223.06128524704	0.025156152338741	58.2	530-59-6;7362-37-
neg_13351	Ps(5-Iso Pgf2Vi/18:2(9Z,12Z))	metab_44578	B(ii)	0	70.9	HMDB0281675	M+FA-H	C42H72NO13P	5.2821166666667	-	neg	874.46826631081	0.028356959738439	50.8	-
neg_13363	Ps(Pgf2Alpha/22:5(7Z,10Z,13Z,16Z,19Z))	metab_44590	B(ii)	0	90.7	HMDB0283427	M+FA-H	C48H78NO13P	5.2768166666667	-	neg	952.52177982091	0.070705211329846	54.6	-
neg_13373	Cynaroside A	metab_44600	B(ii)	0	40.5	HMDB0040532	M-H2O-H	C21H32O10	5.2715	-	neg	425.18365797329	0.052944947565748	45.8	117804-06-5
neg_13381	Val-Phe-Tyr	metab_44608	B(i)	48.1	0	-	M-H	C23H29N3O5	5.2662166666667	-	neg	426.20336172896	0.059473321664689	46.2	-
neg_13395	Sacubitril	metab_44622	B(ii)	0	76.8	HMDB0257439	M+Na-2H	C24H29NO5	5.25555	-	neg	432.17547278503	0.061813170138838	50.5	-
neg_13404	Phe-Ser-Leu	metab_44631	B(i)	91.8	0	-	M-H	C18H27N3O5	5.2502	-	neg	364.18790556379	0.015573525793943	57.1	-
neg_13405	Ala-Ile-Leu	metab_44632	B(i)	70.1	0	-	M-H	C15H29N3O4	5.2502	-	neg	314.20863991801	0.011182087205045	52.6	-
neg_13411	Pi(6 Keto-Pgf1Alpha/16:2(9Z,12Z))	metab_44638	B(ii)	0	76.4	HMDB0276265	M-H2O-H	C45H77O17P	5.2396	-	neg	901.46341007848	0.02449050413476	50.7	-
neg_13416	Parakmerin A	metab_44643	B(ii)	0	62.5	HMDB0033950	2M+FA-H	C19H20O3	5.2396	-	neg	637.28184193742	0.17750779647569	51.3	119555-99-6
neg_13430	Ser-Leu-Ile	metab_44657	B(i)	93.7	0	-	M-H	C15H29N3O5	5.2342833333333	-	neg	330.2036293	0.009491490259332	57.6	-
neg_13435	Amaranthin	metab_44662	B(ii)	0	44.4	HMDB0029406	M-H2O-H	C30H34N2O19	5.2289833333333	C08537	neg	707.15192492285	0.082629225492146	40.8	15167-84-7
neg_13438	Enhydrin	metab_44665	B(ii)	0	48.2	HMDB0251809	M+Hac-H	C23H28O10	5.2289833333333	C09405;_	neg	523.18171273628	0.031450034780669	47.6	33880-85-2;_
neg_13443	Desthiobiotin	metab_44670	B(i)	77	0	HMDB0003581	M-H	C10H18N2O3	5.2289833333333	C01909	neg	213.1245985	0.053267080714478	53.8	533-48-2
neg_13452	Pi(2:0/22:6)	metab_44679	B(i)	48.7	0	-	M+FA-H	C33H51O13P	5.21835	-	neg	731.29890071062	0.15855236924492	46.3	-
neg_13460	(3B,6A,12B,20S,23E)-Dammar-23-Ene-3,6,12,20,25-Pentol 6,20-Diglucoside	metab_44687	B(ii)	0	58.3	HMDB0034668	M-H	C42H72O15	5.21305	-	neg	815.47864295413	0.003165144258764 3	50.1	226712-34-1
neg_13466	Pgp(Pgf1Alpha/A-13:0)	metab_44693	B(ii)	0	50.2	HMDB0274363	M-H2O-H	C39H74O16P2	5.20775	-	neg	841.42155915792	0.014317900802545	46.9	-
neg_13473	15-Deacetylneosolaniol	metab_44700	B(ii)	0	65.7	HMDB0036157	2M-H	C17H24O7	5.2024333333333	C02951	neg	679.2946326	0.009997311678522	49.8	76348-84-0
neg_13474	Cys-Arg-Glu-Lys-Ala	metab_44701	B(ii)	0	68.8	HMDB0250699	M+Na-2H	C23H43N9O8S	5.2024333333333	-	neg	626.27066067451	0.23004742780885	52.4	-

neg_13485	Cucurbitacin I 2-Glucoside	metab_44712	B(ii)	0	83.6	HMDB0035701	M-H	C36H52O12	5.1917333333333	-	neg	675.33246166998	0.075298464010227	52.7	29803-94-9
neg_13491	Val-Leu-Ile	metab_44718	B(i)	76.6	0	-	M-H	C17H33N3O4	5.1917333333333	-	neg	342.23990253555	0.027474088339125	53.9	-
neg_13492	Tryptophyl-Isoleucine	metab_44719	B(ii)	0	57.4	HMDB0029086	M-H	C17H23N3O3	5.1917333333333	-	neg	316.16682726725	0.01242532499201	50.3	-
neg_13495	Acetylleucine	metab_44722	B(i)	99.5	0	HMDB0011756	M-H	C8H15NO3	5.1917333333333	C02710	neg	172.09798516656	0.018632418212463	59.4	1188-21-2
neg_13499	Calceolarioside B	metab_44726	B(i)	52.4	0	-	M-H	C23H26O11	5.1863833333333	-	neg	477.14032109749	0.041594116639179	48.9	-
neg_13509	2-Hydroxy-4-Methylvaleric Acid	metab_44736	B(i)	76.8	0	HMDB0000665	M-H	C6H12O3	5.1863833333333	-	neg	131.07145395046	0.005468453477245	55	498-36-2
neg_13511	25-O-Desacetyl Rifabutin	metab_44738	B(ii)	0	80	HMDB0060732	M-H2O-H	C41H56N4O11	5.1810833333333	-	neg	761.38398124502	0.066739055572438	49.9	-
neg_13516	Hydroxyrepaglinide	metab_44743	B(ii)	0	41	HMDB0060979	M+Na-2H	C27H36N2O5	5.1810833333333	-	neg	489.23584154636	0.012810700601882	46.4	-
neg_13519	Leu-Gln-Ile	metab_44746	B(i)	53.9	0	-	M-H	C17H32N4O5	5.1810833333333	-	neg	371.23012553636	0.028325760056795	49	-
neg_13521	(+/-)-Ethylketocyclazocine	metab_44748	B(ii)	0	52.1	HMDB0242274	M+Na-2H	C19H25NO2	5.1810833333333	-	neg	320.16175500268	0.096069768213715	47.5	-
neg_13528	(4S,6R)-P-Mentha-1,8-Diene-6,7-Diol 7-Glucoside	metab_44755	B(ii)	0	49.7	HMDB0039056	M+Cl	C16H26O7	5.1757833333333	-	neg	365.13555721233	0.09258635147779	47.7	402593-54-8
neg_13534	Steviobioside	metab_44761	B(ii)	0	49.4	HMDB0036707	M-H2O-H	C32H50O13	5.1704166666667	-	neg	623.30249579412	0.016531733875959	46.7	41093-60-1
neg_13539	His-Leu-Leu	metab_44766	B(i)	38.5	0	-	M+Hac-H	C18H31N5O4	5.1704166666667	-	neg	440.2516429	0.021707920463755	46.8	-
neg_13541	Leu-Ala-Ile	metab_44768	B(i)	89.4	0	-	M-H	C15H29N3O4	5.1704166666667	-	neg	314.20867163701	0.020887527656373	56.6	-
neg_13551	Mikamycin B	metab_44778	B(ii)	0	44.5	HMDB0256777	M+FA-H	C45H54N8O10	5.1598333333333	C11615	neg	911.402295	0.024786717692438	46.1	3131/3/1;_
neg_13559	Y1 Receptor Antagonist 1	metab_44786	B(ii)	0	61.3	HMDB0259945	M+FA-H	C28H33N5O3	5.1545166666667	-	neg	532.26086566976	0.13475468419223	47.7	-
neg_13564	Leu-Pro-Ile	metab_44791	B(ii)	0	62.7	HMDB0304805	M-H	C17H31N3O4	5.1491833333333	-	neg	340.22426977226	0.022441103261312	51.4	-
neg_13569	Goldinodox	metab_44796	B(ii)	0	61.9	HMDB0030466	M+FA-H	C44H62N2O12	5.14385	-	neg	855.42247798097	0.081767387225363	47.5	12704-90-4
neg_13575	8-Amino-7-Oxononanoic Acid	metab_44802	B(ii)	0	70.6	HMDB0240687	M-H	C9H17NO3	5.14385	C01092	neg	186.11363206067	0.006098040403299	53.4	-;682799-71-9
neg_13576	Oxytocin	metab_44803	B(ii)	0	95.6	HMDB0002865	M-H2O-H	C43H66N12O12S2	5.1385166666667	C00746;_	neg	987.42839503311	0.034184996451542	55.2	50-56-6;_
neg_13581	Elarofiban	metab_44808	B(ii)	0	70	HMDB0251734	M+Hac-H	C22H32N4O4	5.1385166666667	-	neg	475.25675916305	0.034887231943491	48	-
neg_13597	Gly-Ile-Leu	metab_44824	B(i)	91.1	0	-	M-H	C14H27N3O4	5.1278333333333	-	neg	300.19305346949	0.004302806485177	57	-
neg_13598	Pi(Pgf1Alpha/18:1(11Z))	metab_44825	B(ii)	0	83.3	HMDB0276541	M+Cl	C47H85O16P	5.1225333333333	-	neg	971.53215019086	0.031796492486231	53.1	-
neg_13606	Aginoside Progenin	metab_44833	B(ii)	0	84.5	HMDB0034595	M+Na-2H	C39H64O15	5.1171833333333	-	neg	793.40041447105	0.025290689058751	54.9	61478-53-3
neg_13608	Dihydroergocryptine	metab_44835	B(ii)	0	46.1	HMDB0251310	M+Cl	C32H43N5O5	5.1171833333333	-	neg	612.3002371	0.051465215137866	44.6	-
neg_13610	8-Acetoxy-4'-Methoxypinoresinol 4-Glucoside	metab_44837	B(ii)	0	40.9	HMDB0033278	M-H2O-H	C29H36O13	5.1171833333333	-	neg	573.19735647246	0.24287037980845	45.8	-
neg_13612	7,14-Dihydroxyoctadeca-4,8,10,12-Tetraenedioylcarnitine	metab_44839	B(ii)	0	51.7	HMDB0241558	M-H2O-H	C25H39NO8	5.1171833333333	-	neg	462.24669267993	0.040883786093385	46	-
neg_13626	Asp-Leu-Ile	metab_44853	B(i)	87.5	0	-	M-H	C16H29N3O6	5.1118333333333	-	neg	358.19861741939	0.07228538572363	56	-
neg_13627	L-Gamma-Glutamyl-Beta-Phenyl-Beta-L-Alanine	metab_44854	B(ii)	0	68.6	HMDB0038514	M-H2O-H	C14H18N2O5	5.1118333333333	-	neg	275.10382401074	0.004865832540335	53	10275-84-0
neg_13634	Pyridinoline	metab_44861	B(ii)	0	72	HMDB0000851	M+FA-H	C18H28N4O8	5.1065333333333	-	neg	473.19306514698	0.004983997578617	51.6	63800-01-1
neg_13638	Coixol	metab_44865	B(i)	90.7	0	HMDB0036582	M-H	C8H7NO3	5.1065333333333	-	neg	164.03546501994	0.030011736487483	57.5	532-91-2
neg_13651	Glycocholic Acid 3-Sulfate	metab_44878	B(ii)	0	68.7	HMDB0240728	M-H	C26H43NO9S	5.0958833333333	-	neg	544.26252191395	0.09662198134472	49.6	-
neg_13660	Gln-Leu-Ile	metab_44887	B(i)	79.2	0	-	M-H	C17H32N4O5	5.0905666666667	-	neg	371.23012784657	0.011896139889127	54.7	-
neg_13667	71-Hydroxychlorophyll A	metab_44894	B(ii)	0	87	HMDB0304244	M+FA-H	C55H71MgN4O6-	5.0799666666667	-	neg	952.52233021674	0.043329892840784	50	-
neg_13671	(3'X,5'A,9'X,10'B)-O-(3-Hydroxy-6-Oxo-7-Drimen-11-Yl)Umbelliferone	metab_44898	B(ii)	0	72.4	HMDB0039042	2M-H	C24H28O5	5.0799666666667	-	neg	791.38650744126	0.023106319232237	49.8	-
neg_13680	Prostaglandin Pge2 Glyceryl Ester	metab_44907	B(ii)	0	58.4	HMDB0013045	M+FA-H	C23H38O7	5.0799666666667	-	neg	471.25763569253	0.032413251993566	49.1	-
neg_13681	Flecainide	metab_44908	B(ii)	0	40.3	HMDB0015326	M-H	C17H20F6N2O	5.0799666666667	C07001	neg	413.13060309313	0.026308093727022	47.1	54143-55-4
neg_13686	Pg(Pgd1/A-17:0)	metab_44913	B(ii)	0	64.5	HMDB0270832	M+Na-2H	C43H79O13P	5.0746	-	neg	855.494449	0.086761590175061	48.2	-
neg_13700	His Tyr Ile Asp	metab_44927	B(i)	65.8	0	-	M-H	C25H34N6O8	5.0693	-	neg	545.23635996202	0.25124819268945	50.3	-
neg_13702	(2Z,5Z,9Z)-Hexadeca-2,5,9-Trienedioylcarnitine	metab_44929	B(ii)	0	57.2	HMDB0241503	M-H2O-H	C23H37NO6	5.0693	-	neg	404.24035720117	0.022998381080077	48	-
neg_13709	Hericenone B	metab_44936	B(ii)	0	50.4	HMDB0036548	M+FA-H	C27H31NO4	5.064	-	neg	478.21925607869	0.079619407278568	45.6	126654-53-3
neg_13710	3-(1,1-Dimethylallyl)Scopoletin 7-Glucoside	metab_44937	B(ii)	0	46	HMDB0032853	M-H	C21H26O9	5.064	-	neg	421.15067379093	0.0471140448401	48.6	208333-72-6
neg_13711	1-(2-Hydroxy-4,6-Dimethoxyphenyl)-3-(3,4,5-Trimethoxyphenyl)Propan-1-One	metab_44938	B(i)	46	0	-	M-H	C20H24O7	5.064	-	neg	375.14501504639	0.022155629922512	48.5	-

neg_13713	Ser-Ile-Leu	metab_44940	B(i)	85.7	0	-	M-H	C15H29N3O5	5.064	-	neg	330.20364377548	0.022327096431611	55.9	-
neg_13748	Antibiotic Ll-Ab 664	metab_44975	B(ii)	0	42.5	HMDB0248480	M+Na-2H	C18H30N8O8	5.03745	-	neg	507.19645420872	0.077208203054197	46.3	-
neg_13776	Citreoviridinol A1	metab_45003	B(ii)	0	81.1	HMDB0035369	2M+FA-H	C22H28O8	5.0160833333333	-	neg	885.36095463604	0.15009841260129	52	94161-12-3
neg_13788	Preladenant	metab_45015	B(ii)	0	46.4	HMDB0256760	M+Hac-H	C25H29N9O3	5.0107833333333	-	neg	562.25210655831	0.023272931708393	47.8	-
neg_13794	(1S,2S,4R,8S)-P-Menthane-1,2,8,9-Tetrol 2-Glucoside	metab_45021	B(ii)	0	75.6	HMDB0039975	2M-H	C16H30O9	5.00525	-	neg	731.37380455347	0.01964031840445	50.6	402593-52-6
neg_13810	Pl	metab_45037	B(ii)	0	79.9	HMDB0302594	M+Na-2H	C28H46O10	4.9999	C18210;_	neg	563.28310080598	0.068118735159604	52.3	-;_
neg_13811	Lactose-Lysine	metab_45038	B(ii)	0	65.9	HMDB0253951	M+FA-H	C18H34N2O12	4.9999	-	neg	515.21266839168	0.051412949157618	50.4	-
neg_13814	Val-Tyr-Leu	metab_45041	B(i)	67.3	0	-	M-H	C20H31N3O5	4.9999	-	neg	392.21929384865	0.019900780731452	52	-
neg_13815	Pi(Pgf2Alpha/20:3(8Z,11Z,14Z))	metab_45042	B(ii)	0	71.8	HMDB0277410	M+Cl	C49H83O16P	4.99455	-	neg	993.51437328593	0.0846987800596	50.2	-
neg_13828	N-Acetyl-D-Alloisoleucine	metab_45055	B(i)	97.7	0	-	M-H	C8H15NO3	4.9892166666667	-	neg	172.09800598464	0.014452557208906	58.6	-
neg_13830	Pi(5-Iso Pgf2Vi/20:4(5Z,8Z,11Z,14Z))	metab_45057	B(ii)	0	77.1	HMDB0277528	M+Na-2H	C47H77O16P	4.9838666666667	-	neg	949.4767998	0.039183812168279	51.9	-
neg_13832	Nafenopin	metab_45059	B(ii)	0	44.5	HMDB0255421	2M-H	C20H22O3	4.9838666666667	C11371	neg	619.30800008079	0.027332530524447	44	3771-19-5;_
neg_13844	N-Acetyl-9-Aminominocycline, (4R)-	metab_45071	B(ii)	0	51.5	HMDB0259457	M+Cl	C20H31NO7	4.9785333333333	-	neg	432.17552899948	0.074719827218108	45.9	-
neg_13845	Leu-Val-Ile	metab_45072	B(i)	73.3	0	-	M-H	C17H33N3O4	4.9785333333333	-	neg	342.24016764726	0.086473559744519	52.5	-
neg_13855	Dehydroxypaxilline	metab_45082	B(ii)	0	52.4	HMDB0037531	M+Cl	C27H33NO3	4.9731833333333	-	neg	454.21952562194	0.06420904804002	46.7	112900-05-7
neg_13857	Val-Phe-Ser	metab_45084	B(i)	74.4	0	-	M+Hac-H	C17H25N3O5	4.9731833333333	-	neg	410.19330572072	0.004342581219422	54.7	-
neg_13871	Forsythoside B	metab_45098	B(i)	56.3	0	HMDB0252461	M-H	C34H44O19	4.9625	-	neg	755.24098902232	0.033740472674191	50	-
neg_13897	Teleocidin B 2	metab_45124	B(ii)	0	54.9	HMDB0258787	M+Cl	C28H41N3O2	4.95185	-	neg	486.29360329496	0.035369737773293	46.1	-
neg_13902	6-(3-Benzoyloxy-2-Hydroxypropoxy)-3,4,5-Trihydroxyoxane-2-Carboxylic Acid	metab_45129	B(i)	76.1	0	-	M-H	C16H20O10	4.95185	-	neg	371.09847880493	0.028424984071867	54.1	-
neg_13904	Thr Ile Asp Phe Glu	metab_45131	B(i)	61.1	0	-	M-H	C28H41N5O11	4.94655	-	neg	622.27289262539	0.029899774184281	50.2	-
neg_13928	Chenodeoxycholylaspartic Acid	metab_45155	B(ii)	0	80.7	HMDB0242402	M+FA-H	C28H45NO7	4.9305833333333	-	neg	552.31598603564	0.049159052320498	54.2	-
neg_13929	Leu-Ser-Leu	metab_45156	B(i)	66.2	0	-	M-H	C15H29N3O5	4.9305833333333	-	neg	330.20374481396	0.021367490677509	51.6	-
neg_13940	4-Hydroxytrtradeca-7,10-Dienoylcarnitine	metab_45167	B(ii)	0	53.5	HMDB0241385	M+Na-2H	C21H37NO5	4.9252333333333	-	neg	404.24040605723	0.006980201337069	48.6	-
neg_13950	Phe-Arg-Ile	metab_45177	B(i)	78.4	0	-	M-H	C21H34N6O4	4.9198833333333	-	neg	433.25703703577	0.023265643997659	53.6	-
neg_13959	Citronellyl Beta-Sophoroside	metab_45186	B(ii)	0	45.4	HMDB0032839	M-H	C22H40O11	4.9145833333333	-	neg	479.25118826161	0.032767971824744	47.1	209681-41-4
neg_13961	Ile-Pro-Leu	metab_45188	B(i)	88.1	0	-	M-H	C17H31N3O4	4.9145833333333	-	neg	340.22424990311	0.02922635619307	56.5	-
neg_13963	N-4-[2(R)-Amino-3-Mercaptopropyl]Amino-2-Naphthylbenzoyl-(L)-Leucine, Tfa	metab_45190	B(ii)	0	42.5	HMDB0252709	2M-H	C26H31N3O3S	4.9092333333333	-	neg	929.41156103408	0.043487468402028	46.1	-
neg_13964	Purpureaside C	metab_45191	B(i)	84.2	0	-	M-H	C35H46O20	4.9092333333333	C10488	neg	785.25094740694	0.016473859197095	55.1	108648-07-3
neg_13969	Taurolithocholic Acid	metab_45196	B(i)	58.9	0	HMDB0000722;LMST05040003	M+FA-H	C26H45NO5S	4.9092333333333	C02592	neg	528.30402908622	0.03914482754145	44	516-90-5;
neg_13984	Phe-Asn-Pro	metab_45211	B(i)	70.2	0	-	M-H	C18H24N4O5	4.9039333333333	-	neg	375.1675276	0.003792041877678	52.8	-
neg_13989	Pro-Val-Phe	metab_45216	B(i)	49.3	0	-	M-H	C19H27N3O4	4.89865	-	neg	360.19319304168	0.029498256993337	48.1	-
neg_13993	Licoagrodin	metab_45220	B(ii)	0	57.1	HMDB0035372	M-H	C45H44O9	4.89335	-	neg	727.28599262042	0.07788520089459	47	-
neg_13994	Lampranthin Ii	metab_45221	B(ii)	0	70.6	HMDB0304696	M+Na-2H	C27H30O16	4.89335	C12634;C08552	neg	631.12831698235	0.05983002152484	51.6	32074-65-0;_
neg_14007	Boc-Val-Pro-Arg-Mca	metab_45234	B(ii)	0	72.8	HMDB0249335	M+Hac-H	C31H45N7O7	4.8827333333333	-	neg	686.34827785567	0.094084805637357	50.3	-
neg_14016	Erythromycylamine	metab_45243	B(ii)	0	60.7	HMDB0251939	M+Cl	C37H70N2O12	4.87215	-	neg	769.4581252	0.042406781033846	49.8	-
neg_14017	Pc(Pgf1Alpha/2:0)	metab_45244	B(ii)	0	63.1	HMDB0288899	M+Hac-H	C30H56NO11P	4.87215	-	neg	696.37311655817	0.021180604853078	51.7	-
neg_14018	Moxidectin	metab_45245	B(ii)	0	82.5	HMDB0242623	M+Cl	C37H53NO8	4.87215	-	neg	674.35230732657	0.017685837985516	52	-
neg_14030	Cyclo(Arg-Gly-Asp-D-Phe-Val)	metab_45257	B(ii)	0	83.6	HMDB0250680	M+Cl	C26H38N8O7	4.8668	-	neg	609.25220252564	0.082287738627863	52.5	-
neg_14035	5-Caffeoylshikimic Acid	metab_45262	B(i)	88.3	0	HMDB0033999	M-H	C16H16O8	4.8668	C10434	neg	335.07741399162	0.075973187871049	56.5	73263-62-4
neg_14036	Avermectin A1A	metab_45263	B(ii)	0	84.4	HMDB0242563	M+FA-H	C49H74O14	4.8615	C11984;_	neg	931.51061523762	0.05678747907265	52.3	65195-51-9;_;
neg_14043	Methylprednisolone Hemisuccinate	metab_45270	B(ii)	0	56.9	HMDB0254662	M-H2O-H	C26H34O8	4.8561666666667	-	neg	455.21143663286	0.033568916207978	47.5	-
neg_14046	Desacetylvinblastine Amide	metab_45273	B(ii)	0	72.7	HMDB0250415	M+Cl	C43H55N5O7	4.8508	-	neg	788.38034391626	0.045607499289532	51.3	-
neg_14048	Pa(Pgj2/I-13:0)	metab_45275	B(ii)	0	40.1	HMDB0267452	M+Cl	C36H61O10P	4.8508	-	neg	719.3739333	0.009892702213945	44.4	-
neg_14053	Trp-Glu-Ile	metab_45280	B(i)	54.1	0	-	M+Hac-H	C22H30N4O6	4.8508	-	neg	505.22831738434	0.026207824331637	49.3	-

neg_14058	2-(4-Methyl-5-Thiazolyl)Ethyl Octanoate	metab_45285	B(ii)	0	44.1	HMDB0032423	M+Cl	C14H23NO2S	4.8508	-	neg	304.11514310249	0.031066519950949	45.3	163266-17-9
neg_14067	Lys Phe Thr Asp	metab_45294	B(i)	39.3	0	-	M-H	C23H35N5O8	4.8454666666667	-	neg	508.24147192801	0.020244830214956	46.1	-
neg_14075	Phenylacetylglutamine	metab_45302	B(i)	58.6	0	HMDB0006344;PW_C002728	M-H	C13H16N2O4	4.8454666666667	C04148	neg	263.10146296633	0.017743818284942	48.2	28047-15-6
neg_14076	Prolyl	metab_45303	B(ii)	0	61.7	HMDB0256800	2M-H	C5H9NO	4.8454666666667	-	neg	197.12962542846	0.015057216333649	52	-
neg_14104	16, 17-Dihydro-16Alpha, 17-Dihydroxy Ga9	metab_45331	B(ii)	0	45.4	HMDB0304025	2M-H	C19H25O6-	4.8188166666667	-	neg	697.32072380207	0.032615178467399	43.6	-
neg_14106	Trp-Gly-Ile	metab_45333	B(i)	41.9	0	-	M-H	C19H26N4O4	4.8188166666667	-	neg	373.18835065824	0.078522897081691	47.5	-
neg_14108	Gentisate Aldehyde	metab_45335	B(i)	91	0	HMDB0004062	M-H	C7H6O3	4.8188166666667	C05585	neg	137.02451198909	0.094278348267322	56.7	-;1194-98-5
neg_14113	5'-O-Beta-D-Glucosylpyridoxine	metab_45340	B(ii)	0	78	HMDB0246884	2M-H	C14H21NO8	4.8134833333333	_;C03996	neg	661.24808198795	0.19658403616489	53.3	-;_
neg_14127	Gamma-Amanitin	metab_45354	B(ii)	0	54.7	HMDB0252608	M-H	C39H54N10O13S	4.8027833333333	-	neg	901.35858901725	0.07441363721512	46.6	-
neg_14130	(2R,3Z)-Phycocyanobilin	metab_45357	B(ii)	0	48.8	HMDB0242598	M-H2O-H	C33H38N4O6	4.8027833333333	-	neg	567.25745501951	0.015616180371798	46.9	-
neg_14136	Ac2Pim1(14:0/18:2)	metab_45363	B(i)	62.4	0	-	M+FA-H	C47H85O18P	4.79745	-	neg	1013.5401783986	0.072435457707083	49.3	-
neg_14144	Ala-Val-Phe	metab_45371	B(i)	85.7	0	-	M-H	C17H25N3O4	4.79745	-	neg	334.17738731057	0.034589802000935	55.8	-
neg_14146	3-Hydroxy-3-Methylglutaric Methyl Ester	metab_45373	B(i)	83.6	0	-	M-H2O-H	C7H12O5	4.79745	-	neg	157.05073317342	0.02217453410295	56.2	-
neg_14149	Milataxel	metab_45376	B(ii)	0	69.5	HMDB0254719	M-H2O-H	C44H55NO16	4.79215	-	neg	834.33046756568	0.05042684553017	45.6	-
neg_14159	Scaposin	metab_45386	B(i)	75.1	0	LMPK12111488	M+FA-H	C19H18O9	4.79215	-	neg	435.09102713548	0.096872275960442	51.9	0
neg_14161	Feruloylquinic Acid	metab_45388	B(i)	91.5	0	HMDB0302684	M-H	C17H20O9	4.79215	-	neg	367.10349934349	0.035060694729848	57.4	-
neg_14175	Phe-Ala-Met	metab_45402	B(i)	38.2	0	-	M-H	C17H25N3O4S	4.7868333333333	-	neg	366.14647280239	0.045299131226424	44.3	-
neg_14199	2,6-Dihydroxybenzoic Acid	metab_45426	B(i)	97.6	0	HMDB0013676	M-H	C7H6O4	4.7762	-	neg	153.01947777761	0.016534430945698	58.5	303-07-1
neg_14209	Naloxonazine	metab_45436	B(ii)	0	50.2	HMDB0255437	M+Cl	C38H42N4O6	4.76555	-	neg	685.28431510502	0.065381127047348	45.5	-
neg_14215	Melanotan Ii	metab_45442	B(ii)	0	95.9	HMDB0254419	M-H	C50H69N15O9	4.76025	-	neg	1022.5318786376	0.023681372014848	56.4	-
neg_14218	Concanamycin A	metab_45445	B(ii)	0	86	HMDB0250430	M+Cl	C46H75NO14	4.76025	C15761;_	neg	900.49135251647	0.051890627672161	53.4	80890-47-7;_
neg_14252	Asn-Val-Phe	metab_45479	B(i)	78.8	0	-	M-H	C18H26N4O5	4.7496	-	neg	377.18314168116	0.000269651641793	54.5	-
neg_14253	N-Lactoyl-Tryptophan	metab_45480	B(ii)	0	46.8	HMDB0062178	M+Na-2H	C14H16N2O4	4.7496	-	neg	297.08587553659	0.025372456750002	48.1	-
neg_14256	5,10-Methylene-Thf	metab_45483	B(ii)	0	68.9	HMDB0001533	2M-H	C20H23N7O6	4.7442833333333	C00143	neg	913.33525628401	0.16480505874615	49.3	3432-99-3;31690-11-6
neg_14257	Corchoionoside B	metab_45484	B(ii)	0	56.8	HMDB0030975	2M+FA-H	C19H28O9	4.7442833333333	-	neg	845.34773363673	0.02116429953026	45.6	189344-54-5
neg_14269	Mesaconitine	metab_45496	B(ii)	0	57	HMDB0254473	M+Na-2H	C33H45NO11	4.7389666666667	C08698;_	neg	652.27783837567	0.025301121830251	44.8	2752-64-9;_
neg_14271	Prolyl-Phenylalanyl-Arginine-4-Nitroanilide	metab_45498	B(ii)	0	40	HMDB0259666	M+FA-H	C26H34N8O5	4.7389666666667	-	neg	583.26276412791	0.14620552363955	45.7	-
neg_14274	Gln-Ile-Leu	metab_45501	B(i)	59.5	0	-	M-H	C17H32N4O5	4.7389666666667	-	neg	371.23027345838	0.033624163421965	49.9	-
neg_14279	Mimic A	metab_45506	B(ii)	0	57	HMDB0254736	M-H2O-H	C28H36N2O9	4.7336666666667	-	neg	525.21905644563	0.14473742904952	48	-
neg_14282	Janthitrem F	metab_45509	B(ii)	0	44.6	HMDB0030530	M-H2O-H	C39H51NO7	4.7283333333333	-	neg	626.35236935644	0.072443796030135	46	90986-52-0
neg_14288	Ps(Pgf1Alpha/22:5(7Z,10Z,13Z,16Z,19Z))	metab_45515	B(ii)	0	56	HMDB0283493	M+Na-2H	C48H80NO13P	4.72305	-	neg	930.50552361513	0.078218854516123	47.5	-
neg_14291	Dynorphin B (6-9)	metab_45518	B(ii)	0	84.2	HMDB0012937	M-H2O-H	C26H43N11O6	4.72305	-	neg	586.32105506907	0.005031668953691	54.1	-
neg_14308	Leustroducsin B	metab_45535	B(ii)	0	48.8	HMDB0254044	M-H	C34H56NO10P	4.7123333333333	-	neg	668.36275450627	0.014763009441102	46.4	-
neg_14312	Withaperuvin G	metab_45539	B(ii)	0	55	HMDB0033943	M+Cl	C28H38O8	4.7123333333333	-	neg	537.22224969104	0.045939116816114	47.2	-
neg_14313	Apadenoson	metab_45540	B(ii)	0	66.3	HMDB0248502	M+Na-2H	C23H30N6O6	4.7123333333333	-	neg	507.19615020461	0.025477912911482	52.2	-
neg_14332	4'-Hydroxycilostazol, Trans-	metab_45559	B(ii)	0	40.9	HMDB0247541	M-H2O-H	C20H27N5O3	4.707	-	neg	366.19379009611	0.045275136290297	44.5	-
neg_14338	Citbismine A	metab_45565	B(ii)	0	66	HMDB0041086	M+Na-2H	C35H32N2O10	4.7016666666667	-	neg	661.17491812171	0.11858902985511	48.9	161068-61-7
neg_14342	Laninamivir	metab_45569	B(ii)	0	59.3	HMDB0253972	M-H	C13H22N4O7	4.7016666666667	_;C21557	neg	345.14332875676	0.02659085350697	50	203120-17-6;_
neg_14350	2-Hydroxy-6-(8,11,14-Pentadecatrienyl)Benzoic Acid	metab_45577	B(ii)	0	45.6	HMDB0031059	M+Cl	C22H30O3	4.6963166666667	-	neg	377.19142796532	0.047451023926866	44.4	103904-73-0
neg_14357	Cucurbitacin E	metab_45584	B(ii)	0	45.4	HMDB0250581	M+Cl	C32H44O8	4.6910166666667	C08797;_	neg	591.27794395886	0.11223286667232	44.5	18444-66-1;_;
neg_14363	Val-Glu-Leu	metab_45590	B(i)	88.7	0	-	M-H	C16H29N3O6	4.6910166666667	-	neg	358.19839930534	0.031228854030884	56.2	-
neg_14367	10-Hydroxy-8-Nor-2-Fenchanone Glucoside	metab_45594	B(ii)	0	71.3	HMDB0033643	2M-H	C15H24O7	4.6856666666667	-	neg	631.29473694245	0.23868435066453	50.4	240495-82-3
neg_14368	Cholic Acid Glucuronide	metab_45595	B(ii)	0	57.2	HMDB0002577	M+Na-2H	C30H48O11	4.6856666666667	-	neg	605.29430898416	0.006154733608469	48.1	76060-22-5;
neg_14377	Glu Lys Phe Asp	metab_45604	B(i)	38.5	0	-	M-H	C24H35N5O9	4.6803666666667	-	neg	536.23674391986	0.045936029865353	46.5	-

neg_14381	2,4-Dihydroxycinnamic Acid	metab_45608	B(i)	90.9	0	-	M-H	C9H8O4	4.6803666666667	-	neg	179.03512130235	0.011173642676722	57.6	-
neg_14385	N-Formyl-Met-Leu-Phe-Lys	metab_45612	B(ii)	0	69.2	HMDB0252432	M-H2O-H	C27H43N5O6S	4.6750833333333	-	neg	546.27837975315	0.033593061103359	50.6	-
neg_14392	Troleandomycin	metab_45619	B(ii)	0	65.1	HMDB0015448	M+Cl	C41H67NO15	4.66945	C12753	neg	848.41637082822	0.037807178230444	50.9	2751/9/9;2751-09-9;
neg_14395	Peridinin	metab_45622	B(ii)	0	67.4	HMDB0256341	M+Na-2H	C39H50O7	4.66945	-	neg	651.32636289238	0.021930901094864	49.4	_;
neg_14398	Diphenhydramine N-Glucuronide	metab_45625	B(ii)	0	42.7	HMDB0060897	M-H	C23H30NO7+	4.66945	-	neg	431.19296628887	0.017599205249963	43.6	-
neg_14399	Glu-Gly-Leu	metab_45626	B(i)	68.7	0	-	M-H2O-H	C13H23N3O6	4.66945	-	neg	298.14084077561	0.03531180245348	50.6	-
neg_14407	Pravastatin	metab_45634	B(ii)	0	63.8	HMDB0005022	M-H2O-H	C23H36O7	4.6641166666667	C01844;_	neg	405.22456853278	0.014475407898618	49.9	81093-37-0;_;
neg_14410	Antiarrhythmic Peptide	metab_45637	B(ii)	0	54.9	HMDB0248473	2M-H	C19H30N6O8	4.6587666666667	-	neg	939.42517789219	0.17868021245443	44.2	-
neg_14426	Ixocarpalactone B	metab_45653	B(ii)	0	77.4	HMDB0034339	M+Hac-H	C28H38O8	4.6480666666667	-	neg	561.2702461	0.047660353495277	51.2	71801-44-0
neg_14429	Clindamycin	metab_45656	B(ii)	0	40.8	HMDB0015321	M+Cl	C18H33ClN2O5S	4.6480666666667	C06914	neg	459.14754390288	0.0071579707555259	41.1	18323-44-9
neg_14435	Pro-Tyr-Asn	metab_45662	B(i)	76.2	0	-	M-H	C18H24N4O6	4.6480666666667	-	neg	391.16005234502	0.021155979144206	52.9	-
neg_14441	Rubiscolin-6	metab_45668	B(ii)	0	77.1	HMDB0257355	M-H2O-H	C39H54N6O10	4.6427333333333	-	neg	747.36901584234	0.061297067802261	51.6	-
neg_14470	Paramethasone	metab_45697	B(ii)	0	59	HMDB0015462	M+Cl	C22H29FO5	4.62675	C07413	neg	427.17213401163	0.18130351442081	48.1	53-33-8
neg_14471	Sinefungin	metab_45698	B(ii)	0	92.2	HMDB0258302	M+Cl	C15H23N7O5	4.62675	-	neg	416.14182813513	0.045615897985742	54.1	-
neg_14472	Ala-Pro-Tyr	metab_45699	B(i)	64.5	0	-	M-H	C17H23N3O5	4.62675	-	neg	348.1539232	0.051361726711434	49.1	-
neg_14480	Glu-Trp-Gln	metab_45707	B(i)	73.2	0	-	M-H2O-H	C21H27N5O7	4.6214333333333	-	neg	442.17406503227	0.054161887332033	52.4	-
neg_14482	Trp-Tyr	metab_45709	B(i)	80.7	0	HMDB0029095	M-H	C20H21N3O4	4.6214333333333	-	neg	366.14583098461	0.040355385127984	54.1	-
neg_14483	Acetyl-N-Formyl-5-Methoxykynurenamine	metab_45710	B(i)	47	0	HMDB0004259;PW_C002112	M+FA-H	C13H16N2O4	4.6214333333333	C05642	neg	309.10697583978	0.010911125241305	45.8	52450-38-1
neg_14509	Thymulin	metab_45736	B(ii)	0	84.5	HMDB0247858	M+Na-2H	C33H54N12O1	4.6000833333333	-	neg	879.35052500529	0.021922264779067	53.8	-
neg_14512	Obacunone 17-O-Beta-D-Glucoside	metab_45739	B(ii)	0	78.2	HMDB0302542	M-H2O-H	C32H42O13	4.6000833333333	-	neg	615.23960579356	0.01726111684168	51.6	-
neg_14513	Glutaminyl-Gamma-Glutamate	metab_45740	B(ii)	0	41.1	HMDB0028811	2M+FA-H	C10H18N4O5	4.6000833333333	-	neg	593.25797238942	0.008950571281130	43.3	-
neg_14524	N-Acetyl-D-Glucosaminyldiphosphodolichol	metab_45751	B(ii)	0	59.3	HMDB0001445	M-H	C23H43NO12P	4.59475	C04500	neg	586.21340507312	0.03177165862941	49.3	-;_
neg_14535	Leu-Val-Tyr	metab_45762	B(i)	88.6	0	-	M-H	C20H31N3O5	4.5894166666667	-	neg	392.21910398072	0.004272586893735	56.4	-
neg_14548	Ser-Phe-Lys	metab_45775	B(i)	45.6	0	-	M+FA-H	C18H28N4O5	4.5788166666667	-	neg	425.2027052	0.013984142853882	46.2	-
neg_14551	Crustacean Erythrophore Concentrating Hormone	metab_45778	B(ii)	0	75.7	HMDB0257325	M-H	C45H59N11O1	4.5735166666667	-	neg	928.42846064707	0.033777851179862	51.9	-
neg_14555	Benzoylhypaconine	metab_45782	B(ii)	0	60.3	HMDB0249052	M+Hac-H	C31H43NO9	4.5735166666667	-	neg	632.30807502836	0.12823503941133	50.2	-
neg_14575	Trans-4-Hydroxycyclohexanecarboxylic Acid	metab_45802	B(i)	75.6	0	-	M+Hac-H	C7H12O3	4.5682	-	neg	203.09259191653	0.041968454518565	54.5	-
neg_14586	Val-Ala-Ile	metab_45813	B(i)	93.6	0	-	M-H	C14H27N3O4	4.5629166666667	-	neg	300.19302476692	0.038787638027751	57.3	-
neg_14598	3,4-Methylenedioxybenzoic Acid	metab_45825	B(ii)	0	56.3	HMDB0032613	M+FA-H	C8H6O4	4.5576	-	neg	211.02491312702	0.009031607228658	50.5	94-53-1
neg_14602	Viloxazine	metab_45829	B(ii)	0	40.3	HMDB0259807	2M+FA-H	C13H19NO3	4.55225	-	neg	519.26671171152	0.029445094441473	45.2	-
neg_14606	Phe-Gly-Lys	metab_45833	B(i)	89.9	0	-	M+FA-H	C17H26N4O4	4.55225	-	neg	395.19239895348	0.038430823445609	56.4	-
neg_14607	Na-P-Hydroxycoumaroyltryptophan	metab_45834	B(ii)	0	46.3	HMDB0041370	M-H	C20H18N2O4	4.55225	-	neg	349.11700246053	0.081985338460387	45.8	-
neg_14608	Val Trp	metab_45835	B(i)	84.6	0	-	M-H	C16H21N3O3	4.55225	-	neg	302.15119077238	0.047773776876516	55.5	-
neg_14610	S-Glutaryldihydrolipoamide	metab_45837	B(ii)	0	47.8	HMDB0006832	2M-H	C13H23NO4S2	4.5469166666667	C06157	neg	641.20898328871	0.063789991136242	45.2	-
neg_14617	Bryo 1	metab_45844	B(ii)	0	63.8	HMDB0249409	M+Cl	C47H68O17	4.5415666666667	-	neg	939.41555656647	0.052498023054163	49.3	-
neg_14619	Bacoside A	metab_45846	B(ii)	0	46.2	HMDB0248842	M+Cl	C41H68O13	4.5415666666667	-	neg	803.43948541264	0.03518615063872	46.2	-
neg_14620	Pa(Pge1/A-17:0)	metab_45847	B(ii)	0	42.6	HMDB0267096	M+Na-2H	C40H73O11P	4.5415666666667	-	neg	781.45764634297	0.02834284530834	45.8	-
neg_14627	Glu-Val-Tyr	metab_45854	B(i)	60.9	0	-	M-H2O-H	C19H27N3O7	4.5415666666667	-	neg	390.16718797936	0.008712982059316	50.9	-
neg_14636	Pe(Pgf2Alpha/Dime(9,3))	metab_45863	B(ii)	0	54	HMDB0284993	M+Na-2H	C43H74NO12P	4.5308833333333	-	neg	848.46142693471	0.038754178623448	45.5	-
neg_14638	Chlorogenoquinone	metab_45865	B(ii)	0	56.3	HMDB0029383	2M-H	C16H16O9	4.5308833333333	-	neg	703.15532784594	0.020652914226906	47.1	24321-18-4
neg_14640	Butyloxycarbonyl-Leucyl-Glycyl-Arginine-4-Nitroanilide	metab_45867	B(ii)	0	51.1	HMDB0247808	M+Na-2H	C25H40N8O7	4.5308833333333	-	neg	585.27785700471	0.21677113243831	47.8	-
neg_14658	Ile-Lys-Leu	metab_45885	B(i)	85.1	0	-	M-H	C18H36N4O4	4.5202166666667	-	neg	371.26648586532	0.013080995418432	55.6	-
neg_14668	Cis-And Trans-Ethyl 2,4-Dimethyl-1,3-Dioxolane-2-Acetate	metab_45895	B(ii)	0	52.2	HMDB0032200	2M+FA-H	C9H16O4	4.5148833333333	-	neg	421.20925797649	0.028774488084424	47.9	-
neg_14682	Riboflavin	metab_45909	B(i)	80.7	0	HMDB0000244;HMDB0247501;PW_C000165	M-H	C17H20N4O6	4.5095333333333	_;C00255	neg	375.13111115469	0.04519297766154	54.4	83-88-5;_

neg_14685	(1Xi,3Xi)-1,2,3,4-Tetrahydro-1-Methyl-Beta-Carboline-3-Carboxylic Acid	metab_45912	B(ii)	0	62.8	HMDB0037942	M-H	C13H14N2O2	4.5095333333333	-	neg	229.09833616414	0.012091450959306	51.9	-
neg_14692	Ganoderic Acid X	metab_45919	B(ii)	0	64.7	HMDB0035332	M+Na-2H	C32H48O5	4.5039333333333	-	neg	533.32062820482	0.04267663987187	48.7	86377-53-9
neg_14694	Gly-Tyr-Lys	metab_45921	B(i)	57.8	0	-	M+FA-H	C17H26N4O5	4.5039333333333	-	neg	411.18631405301	0.057603802478238	49.6	-
neg_14700	Tirbanibulin	metab_45927	B(ii)	0	68.7	HMDB0304857	M+Hac-H	C26H29N3O3	4.4985833333333	-	neg	490.2310029	0.018188897725086	51	-
neg_14703	Lamiide	metab_45930	B(i)	40.5	0	-	M-H2O-H	C17H26O12	4.4985833333333	C11644	neg	403.12470746033	0.05254138012153	47.1	27856-54-8
neg_14705	Ps(Pgf1Alpha/22:5(4Z,7Z,10Z,13Z,16Z))	metab_45932	B(ii)	0	53.1	HMDB0283389	M+Na-2H	C48H80NO13P	4.4932333333333	-	neg	930.51672254374	0.054359496840401	48	-
neg_14720	Geniposide	metab_45947	B(i)	59.4	0	-;HMDB0252679	M+FA-H	C17H24O10	4.4878833333333	_;C09781	neg	433.13541352146	0.076997355105038	47.9	24512-63-8;_
neg_14726	Deacetylnomilin	metab_45953	B(ii)	0	51.2	HMDB0035684	M+Hac-H	C26H32O8	4.48255	-	neg	531.22112419778	0.018862395933564	48.1	3264-90-2
neg_14730	Doramectin	metab_45957	B(ii)	0	87.9	HMDB0242638	M+FA-H	C50H74O14	4.47725	-	neg	943.51078687319	0.022744739979952	53.8	-
neg_14739	Crocin 3	metab_45966	B(ii)	0	81.7	HMDB0039121	M-H	C32H44O14	4.4719666666667	-	neg	651.26341572455	0.023195564645838	52.4	55750-85-1
neg_14764	Proline Betaine	metab_45991	B(ii)	0	63.7	HMDB0004827	M+FA-H	C7H13NO2	4.4613	C10172	neg	188.0928916	0.001810451537912	52.5	471-87-4
neg_14766	Hex2Cer(24:2_2O)	metab_45993	B(i)	79.7	0	-	M+Cl	C36H65NO13	4.45595	-	neg	754.4085189	0.017358695162601	53.1	-
neg_14777	Pg(5-Iso Pgf2Vi/I-12:0)	metab_46004	B(ii)	0	69.1	HMDB0271094	M+FA-H	C36H65O13P	4.4506	-	neg	781.42180430944	0.052115716857568	50	-
neg_14780	Cyclo(D-Trp-D-Asp-Pro-D-Ile-Leu)	metab_46007	B(ii)	0	71.8	HMDB0250629	M+Hac-H	C32H44N6O7	4.4506	-	neg	683.33700932199	0.006243300430657	51.2	-
neg_14788	Gly-Val-Leu	metab_46015	B(i)	92.7	0	-	M-H	C13H25N3O4	4.4506	-	neg	286.17733904424	0.045176569874401	57.6	-
neg_14790	Angiotensin Iv	metab_46017	B(ii)	0	68.4	HMDB0001038	M+FA-H	C40H54N8O8	4.44525	C15849	neg	819.40112271765	0.021347308623981	51.5	12676-15-2
neg_14799	Ala-Val-Ile	metab_46026	B(i)	80.8	0	-	M-H	C14H27N3O4	4.44525	-	neg	300.19301620466	0.002914380888441	55.2	-
neg_14814	Isomorellic Acid	metab_46041	B(ii)	0	56.4	HMDB0030734	M+Hac-H	C33H36O8	4.4345833333333	-	neg	619.25605017736	0.040671650494812	49.2	-
neg_14817	Rh-34	metab_46044	B(i)	70.1	0	-	M+FA-H	C18H19N3O3	4.4345833333333	-	neg	370.13864624564	0.045728774665418	48.6	-
neg_14818	4-O-Beta-D-Glucosyl-4-Coumaric Acid	metab_46045	B(i)	42.2	0	HMDB0039509	M-H	C15H18O8	4.4345833333333	-	neg	325.09305234452	0.066524264856928	47.1	14364-05-7
neg_14822	Met-Glu-His-Phe-Arg-Trp-Gly	metab_46049	B(ii)	0	58.1	HMDB0254491	M-H	C44H59N13O10S	4.4292166666667	-	neg	960.40988584215	0.037938501504875	48.2	-
neg_14847	8-O-Acetyl Shanzhiside Methyl Ester	metab_46074	B(ii)	0	65.3	HMDB0248874	M-H	C19H28O12	4.4185833333333	-	neg	447.15045106159	0.062669282138156	51.1	-
neg_14854	Pa(3:0/22:6)	metab_46081	B(i)	48.6	0	-	M+FA-H	C28H43O8P	4.41325	-	neg	583.27016951889	0.032606877567999	45.2	-
neg_14856	Dg(Pgf2Alpha/0:0/2:0)	metab_46083	B(ii)	0	65.3	HMDB0296918	M+FA-H	C25H42O8	4.41325	-	neg	515.28375027272	0.02260896898888	50.9	-
neg_14858	Pravastatin Lactone	metab_46085	B(ii)	0	48.8	HMDB0256737	M-H	C23H34O6	4.41325	-	neg	405.2246299	0.01068178436084	46.1	-
neg_14863	Linalool (8-Hydroxydihydro-)	metab_46090	B(ii)	0	63.4	HMDB0304700	M+Na-2H	C32H42O14	4.4079333333333	C06740	neg	671.22766458208	0.035107384540469	47.7	-
neg_14868	Phe-Arg-Leu	metab_46095	B(i)	51.2	0	-	M-H	C21H34N6O4	4.4079333333333	-	neg	433.25707219576	0.027793542818722	49.5	-
neg_14876	Verbenalin	metab_46103	B(ii)	0	69.6	HMDB0301853	M+Na-2H	C17H24O10	4.4026	C09802	neg	409.1119103	0.036943153638882	52.7	548-37-8;_;
neg_14878	Phe-Ile-Thr	metab_46105	B(i)	71.3	0	-	M-H	C19H29N3O5	4.4026	-	neg	378.20362984438	0.009667852275272	52.7	-
neg_14891	Leu-Lys-Leu	metab_46118	B(i)	68.8	0	-	M-H	C18H36N4O4	4.3972833333333	-	neg	371.26648201839	0.0233917386346	52.3	-
neg_14893	Coniferin	metab_46120	B(ii)	0	73.7	HMDB0013682	M-H	C16H22O8	4.3972833333333	C00761	neg	341.12416808674	0.007807290405823	54	531-29-3;124151-33-3
neg_14903	Sulfo Jasmonate	metab_46130	B(i)	60.4	0	-	M-H	C12H18O7S	4.39195	-	neg	305.07022117322	0.021520151109437	50	-
neg_14942	Alectrol	metab_46169	B(ii)	0	58.3	HMDB0041372	M+FA-H	C19H22O6	4.3707	C09059	neg	391.13718999178	0.013590382951318	47.8	-;143572-84-3
neg_14944	4-Hydroxybenzoic Acid	metab_46171	B(i)	97.4	0	HMDB0000500;PW_C000382	M-H	C7H6O3	4.3707	C00156	neg	137.02453498005	0.011777173650499	58.9	99-96-7
neg_14946	Beta-Isorhodomycinone	metab_46173	B(ii)	0	83.6	HMDB0249134	M+Hac-H	C40H51NO16	4.3654	-	neg	860.34089816902	0.040844922659869	53.7	-
neg_14959	N-Eicosapentaenoyl Asparagine	metab_46186	B(ii)	0	44.7	HMDB0242066	M-H2O-H	C24H36N2O4	4.3654	-	neg	397.24576652532	0.036164142635388	44.8	-
neg_14969	Silodosin	metab_46196	B(ii)	0	52.3	HMDB0258291	M-H2O-H	C25H32F3N3O	4.3600666666667	-	neg	476.21525782243	0.018848218145022	48.1	-
neg_14981	Ala-Glu-Leu	metab_46208	B(i)	76.2	0	-	M-H2O-H	C14H25N3O6	4.3547166666667	-	neg	312.1564665	0.021672680588522	53.9	-
neg_14987	Olitorin	metab_46214	B(ii)	0	84.4	HMDB0034361	M+Hac-H	C35H52O14	4.3494166666667	-	neg	755.34766883369	0.049472111951496	54.8	13289-20-8
neg_14995	Asn-Val-Ile	metab_46222	B(i)	85.7	0	-	M-H	C15H28N4O5	4.3494166666667	-	neg	343.19877943234	0.012308171632407	54.5	-
neg_15002	Glaucarubin	metab_46229	B(ii)	0	49.5	HMDB0035626	M+Na-2H	C25H36O10	4.3441333333333	C08760	neg	517.20540595683	0.02363493167663	48.5	1448-23-3
neg_15003	Pgp(Pgj2/I-14:0)	metab_46230	B(ii)	0	62.5	HMDB0275026	M+Na-2H	C40H70O15P2	4.3385166666667	-	neg	873.38638021348	0.047804205574013	49.1	-
neg_15012	Rutaretin 9-Rutinoside	metab_46239	B(ii)	0	65.9	HMDB0034611	M-H2O-H	C26H34O14	4.3331666666667	-	neg	551.17415383782	0.12252895268952	48.9	160845-05-6
neg_15036	Asn-Leu-Tyr	metab_46263	B(i)	77.5	0	-	M-H	C19H28N4O6	4.3172666666667	-	neg	407.19388235057	0.040487196587285	53.3	-

neg_15048	Phe-Met	metab_46275	B(i)	83	0	HMDB0029001	M-H	C14H20N2O3S	4.3119666666667	-	neg	295.11232750678	0.017151763995997	54.3	15080-84-9
neg_15054	S-(11-Oh-9-Deoxy-Delta9,12-Pgd2)-Glutathione	metab_46281	B(ii)	0	58.6	HMDB0013056	M-H2O-H	C30H49N3O10	4.3066666666667	-	neg	624.29756577162	0.051740194753981	47.9	-
neg_15065	Sulfoglycolithocholate(2-)	metab_46292	B(ii)	0	43.7	HMDB0062801	M-H	C26H43NO7S	4.30135	-	neg	512.27277001562	0.021173959688199	45.3	-
neg_15071	Voacamine	metab_46298	B(i)	66.4	0	HMDB0015597	M+FA-H	C43H52N4O5	4.29605	C09252	neg	749.39201776286	0.075688705337267	48.3	3371-85-5
neg_15074	Pirarubicinol	metab_46301	B(ii)	0	79.4	HMDB0256589	M+FA-H	C32H39NO12	4.29605	-	neg	674.24069588605	0.044724943629029	51.4	-
neg_15075	Tigecycline	metab_46302	B(ii)	0	84.3	HMDB0014700	M+Na-2H	C29H39N5O8	4.29605	C12012	neg	606.25326473308	0.001919662595195	55.4	220620-09-7
neg_15079	Dyspropterin	metab_46306	B(ii)	0	63.7	HMDB0001195	M-H2O-H	C9H11N5O3	4.29605	C03684	neg	218.06845109918	0.018559156987345	50.4	89687-39-8
neg_15085	Kapporphin	metab_46312	B(ii)	0	41.1	HMDB0303767	M+Hac-H	C25H31N5O8	4.2907	-	neg	588.22700584928	0.0864321951112	44.7	-
neg_15088	Thr Asp Phe Glu	metab_46315	B(i)	53	0	-	M-H	C22H30N4O10	4.2907	-	neg	509.18924866288	0.034073818615688	45.6	-
neg_15092	4-Hydroxytrideca-7,10-Dienoylcarnitine	metab_46319	B(ii)	0	43.7	HMDB0241334	M+Na-2H	C20H35NO5	4.2907	-	neg	390.22449354167	0.031793652581833	45.4	-
neg_15101	Dg(6 Keto-Pgf1Alpha/8:0/0:0)	metab_46328	B(ii)	0	66.7	HMDB0297120	M-H	C31H54O9	4.2853833333333	-	neg	569.36704309881	0.028495034212494	50.1	-
neg_15111	Glu-His-Thr	metab_46338	B(i)	43.4	0	-	M+FA-H	C15H23N5O7	4.2800333333333	-	neg	430.15984660013	0.047830838095172	46.6	-
neg_15112	Etiracetam	metab_46339	B(ii)	0	69.6	HMDB0252105	2M+FA-H	C8H14N2O2	4.2800333333333	-	neg	385.20942048986	0.041127557529783	52.7	-
neg_15115	O1-[4-(Tert-Butyl)Benzoyl]-2-(4-	metab_46342	B(i)	37.1	0	-	M+FA-H	C19H21N3O4	4.2747	-	neg	400.14911699641	0.020708404475361	44.1	-
neg_15132	Tyr-Ala-Lys	metab_46359	B(i)	51.2	0	-	M+FA-H	C18H28N4O5	4.26935	-	neg	425.20234327189	0.058103828126541	46.6	-
neg_15135	Gln-Val-Ile	metab_46362	B(i)	64.8	0	-	M-H	C16H30N4O5	4.26935	-	neg	357.21448907222	0.009547185206934	51.9	-
neg_15177	Puromycin	metab_46404	B(ii)	0	46.6	HMDB0256926	M+Hac-H	C22H29N7O5	4.24795	C01610;_	neg	530.23713201504	0.005584945464052	47.1	53-79-2;_
neg_15186	Ribalinium	metab_46413	B(ii)	0	61.8	HMDB0033346	2M+FA-H	C16H20NO4+	4.24265	C10735	neg	625.28170263117	0.074674136834101	49.1	6883-22-3
neg_15188	Olivil 4'-O-Glucoside	metab_46415	B(i)	61.4	0	-	M+FA-H	C26H34O12	4.24265	-	neg	583.20394782962	0.1192744851733	50.4	-
neg_15196	Ile Phe Asp Arg	metab_46423	B(i)	78.6	0	-	M-H	C25H39N7O7	4.2373166666667	-	neg	548.28431379085	0.043488080394633	53.4	-
neg_15209	Tyr-Ile	metab_46436	B(i)	88.5	0	HMDB0029108	M-H	C15H22N2O4	4.23195	-	neg	293.1508602	0.011078256477125	56.8	-
neg_15214	Arg-Met-Tyr	metab_46441	B(i)	40.4	0	-	M+FA-H	C20H32N6O5S	4.2265833333333	-	neg	513.21807958352	0.099074856178084	40.8	-
neg_15221	N-Cyano-N'-[2-(Trifluoromethyl)Phenyl]Pyrrolidine-1-Carboximidamide	metab_46448	B(i)	75.7	0	-	M+Hac-H	C13H13F3N4	4.2265833333333	-	neg	341.12430905255	0.076779841152352	52.9	-
neg_15225	Ile-Glu-Thr-Asp-Fluoromethyl Ketone	metab_46452	B(ii)	0	76.1	HMDB0253396	M+Hac-H	C20H33FN4O9	4.2212833333333	-	neg	551.2398959	0.023586904987086	49.4	-
neg_15232	Citrusin Iii	metab_46459	B(ii)	0	69.4	HMDB0303682	M+Na-2H	C36H53N7O9	4.2159833333333	-	neg	748.36399026908	0.057124602529509	51.5	-
neg_15234	Sonolisib	metab_46461	B(ii)	0	60.7	HMDB0258378	M+FA-H	C29H35NO8	4.2159833333333	-	neg	570.23197255225	0.24635783871938	46.7	-
neg_15235	Trp-Tyr-His	metab_46462	B(i)	55	0	-	M-H	C26H28N6O5	4.2159833333333	-	neg	503.20026040597	0.075421544360565	46.6	-
neg_15246	Iprofenin	metab_46473	B(ii)	0	61.3	HMDB0253569	M+Na-2H	C15H20N2O5	4.2106833333333	-	neg	329.11492984052	0.053955090070854	48	-
neg_15258	7-Methylxanthosine	metab_46485	B(ii)	0	59.4	HMDB0301918	M+Na-2H	C11H15N4O6+	4.2053833333333	C16352	neg	320.07534276213	0.016172162569012	50	-;_
neg_15260	Tyrosine Lactate	metab_46487	B(ii)	0	54.8	HMDB0259352	M-H	C12H15NO5	4.2053833333333	-	neg	252.08784191277	0.006503342773687	50.2	-
neg_15267	Ser-Val-Leu	metab_46494	B(i)	74.6	0	-	M-H	C14H27N3O5	4.2000333333333	-	neg	316.18790545037	0.010984650579013	53.8	-
neg_15270	Pa(6 Keto-Pgf1Alpha/10:0)	metab_46497	B(ii)	0	54.9	HMDB0262672	M-H2O-H	C33H59O12P	4.1947	-	neg	659.35265490431	0.005547106636980	48.9	-
neg_15277	Asn-Ile-Tyr	metab_46504	B(i)	46.5	0	-	M-H	C19H28N4O6	4.1947	-	neg	407.19358554544	0.037965781123127	47.8	-
neg_15283	Hexapeptide Analogue	metab_46510	B(ii)	0	70.5	HMDB0249946	M-H	C29H46N6O12	4.1894	-	neg	701.28519937532	0.096987102589216	49	-
neg_15290	3-Methylpentyl Glucosinolate	metab_46517	B(ii)	0	41.5	HMDB0036355	M+Cl	C13H25NO9S2	4.1894	-	neg	438.06553285863	0.27804621479231	45.1	-
neg_15291	Arg-Leu-Ile	metab_46518	B(i)	59	0	-	M-H	C18H36N6O4	4.1894	-	neg	399.2726216	0.030177220293774	50.2	-
neg_15293	Caffeoyl Quinic Acid	metab_46520	B(i)	89	0	HMDB0003164	M-H	C16H18O9	4.1894	C00852	neg	353.08788927151	0.021022841116529	57.3	327-97-9;202650-88-2
neg_15294	Quinic Acid	metab_46521	B(i)	85.8	0	HMDB0003072	M-H	C7H12O6	4.1894	C00296	neg	191.05621888918	0.064006113831969	56	77-95-2
neg_15314	Luseogliflozin	metab_46541	B(ii)	0	59.7	HMDB0254222	M+FA-H	C23H30O6S	4.1787166666667	-	neg	479.17622558932	0.018360670346122	48.7	-
neg_15322	Penitrem E	metab_46549	B(ii)	0	75.7	HMDB0256241	M-H	C37H45NO6	4.1730833333333	C20597;_	neg	598.32105302687	0.021903836664166	51.3	78213-66-8;_
neg_15357	Trp-Glu-Ser	metab_46584	B(i)	39.1	0	-	M+FA-H	C19H24N4O7	4.15715	-	neg	465.16313466384	0.011568192288183	44.9	-
neg_15362	Kanzonol V	metab_46589	B(ii)	0	41.9	HMDB0029788	2M+FA-H	C24H24O4	4.1518166666667	-	neg	797.33230465711	0.10939991724493	46.7	184584-65-4
neg_15364	Lnape(24:7/N-3:0)	metab_46591	B(i)	63.5	0	-	M+FA-H	C32H50NO8P	4.1518166666667	-	neg	652.33067843796	0.0227121910103	48.1	-
neg_15371	Gly-Ile-Tyr	metab_46598	B(i)	72.3	0	-	M-H	C17H25N3O5	4.1518166666667	-	neg	350.17226489526	0.016721653358642	53.1	-

neg_15373	Tetrahydrodipicolinate	metab_46600	B(ii)	0	51.5	HMDB0012289	M-H	C7H9NO4	4.1518166666667	C03972	neg	170.04599852056	0.027803087980889	49.4	4226-22-6
neg_15391	Ile-Phe-Ser	metab_46618	B(i)	92.1	0	-	M-H	C18H27N3O5	4.1411	-	neg	364.18800521984	0.024941789848813	56.8	-
neg_15403	3-Hydroxymugineic Acid	metab_46630	B(ii)	0	67.6	HMDB0033927	2M+FA-H	C12H20N2O9	4.1357666666667	-	neg	717.23280202677	0.026370904693027	50.7	74235-23-7
neg_15404	Ichangin 4-Glucoside	metab_46631	B(ii)	0	63.9	HMDB0039340	M+FA-H	C32H42O14	4.1357666666667	-	neg	695.25085769838	0.018418071405921	49.6	-
neg_15405	Resiniferatoxin	metab_46632	B(ii)	0	65.8	HMDB0242596	M+FA-H	C37H40O9	4.1357666666667	C09179;_	neg	673.26898914505	0.004897497968913	50.4	57444-62-9;_
neg_15410	Tyr-Glu-Lys	metab_46637	B(i)	67.7	0	-	M-H	C20H30N4O7	4.1357666666667	-	neg	437.20230467735	0.020362522807211	50.2	-
neg_15411	Phe-Glu-Asp	metab_46638	B(i)	63.1	0	-	M-H	C18H23N3O8	4.1357666666667	-	neg	408.14170279443	0.044146048121533	51.6	-
neg_15415	(+/-)-Catechin	metab_46642	B(i)	61	0	-	M-H	C15H14O6	4.1357666666667	C17590	neg	289.07204520166	0.022347810354461	50.5	-
neg_15419	Ceanothine E	metab_46646	B(ii)	0	77.7	HMDB0029342	M+Na-2H	C34H40N4O4	4.1304166666667	-	neg	589.28416338958	0.030126347622811	49.9	23926-98-9
neg_15421	Dg(6 Keto-Pgf1Alpha/2:0/0:0)	metab_46648	B(ii)	0	66.9	HMDB0296912	M+FA-H	C25H42O9	4.1304166666667	-	neg	531.27896964496	0.031823579249831	51.1	-
neg_15432	Elvitegravir	metab_46659	B(ii)	0	60.6	HMDB0251760	M-H	C23H23ClFNO	4.1251166666667	-	neg	446.11584953191	0.033410007476202	44.6	-
neg_15441	Pi(O-11:0/22:6)	metab_46668	B(i)	49.4	0	-	M+Hac-H	C42H71O12P	4.1198333333333	-	neg	857.48329811338	0.034875106432042	47.3	-
neg_15442	Myristicanol B	metab_46669	B(ii)	0	46.4	HMDB0041312	2M+FA-H	C22H28O7	4.1198333333333	-	neg	853.35855229878	0.058549324987699	46.6	114912-33-3
neg_15453	Tyr-Arg-Leu	metab_46680	B(i)	84.3	0	-	M-H	C21H34N6O5	4.1145333333333	-	neg	449.25263408356	0.0254224811878	54.8	-
neg_15458	Allyl Cysteine	metab_46685	B(ii)	0	61.7	HMDB0248164	M+Hac-H	C6H11NO2S	4.1145333333333	-	neg	220.06502819089	0.014513056720199	49.9	-
neg_15462	Cyclosquamosin C	metab_46689	B(ii)	0	96.7	HMDB0303376	M+FA-H	C39H65N9O11	4.1039166666667	-	neg	912.45861402197	0.041921545011221	54.9	-
neg_15463	10-Deacetyltaxol	metab_46690	B(ii)	0	55.6	HMDB0244265	M+Hac-H	C45H49NO13	4.1039166666667	-	neg	870.33842938163	0.037799919247857	47.3	-
neg_15484	Methyltetrahydrofolic Acid	metab_46711	B(ii)	0	48.8	HMDB0254666	M-H2O-H	C20H25N7O6	4.0986333333333	-	neg	440.16748545402	0.003377910929601	47.2	-
neg_15487	Ile-Ala-Ile	metab_46714	B(i)	60.5	0	-	M-H	C15H29N3O4	4.0986333333333	-	neg	314.20872204761	0.025249663757705	50.7	-
neg_15498	Lysyl-Aspartyl-Glutamyl-Leucine	metab_46725	B(ii)	0	59.9	HMDB0247848	M-H	C21H37N5O9	4.0933166666667	-	neg	502.25221774119	0.022597530678545	51.3	-
neg_15499	His-Trp-Arg	metab_46726	B(i)	42.1	0	-	M-H	C23H31N9O4	4.0933166666667	-	neg	496.23908109725	0.15849517470251	44	-
neg_15501	Ser Leu Ser Gly Leu	metab_46728	B(i)	64.1	0	-	M-H	C20H37N5O8	4.0933166666667	-	neg	474.2571806	0.021580746015866	51.9	-
neg_15502	D-Phe-L-Pro-L-Arg-(Chloromethyl) Ketone	metab_46729	B(ii)	0	41.6	HMDB0257893	M-H2O-H	C22H31ClN6O	4.0933166666667	-	neg	459.18855659425	0.011930787929817	40.1	-
neg_15508	6-Hydroxysandoricin	metab_46735	B(ii)	0	58.1	HMDB0037556	M-H	C31H40O12	4.0880166666667	C01536	neg	603.23962529604	0.10517630901933	47.7	133585-56-5
neg_15521	Pentanoic Acid, 5-(Dipentylamino)-5-Oxo-4-((3-Quinolinylcarbonyl)Amino)-, (R)-	metab_46748	B(ii)	0	63.8	HMDB0247713	M+FA-H	C25H35N3O4	4.0827333333333	-	neg	486.2573113	0.053096075803313	48.9	-
neg_15531	Dextran-70	metab_46758	B(ii)	0	73.6	HMDB0256672	M-H	C17H32O10	4.0774333333333	-	neg	395.19249944322	0.12574779504199	52.8	-
neg_15544	Rifamycins	metab_46771	B(ii)	0	71.3	HMDB0257226	M+Cl	C37H45NO12	4.0668	-	neg	730.26671968749	0.008831531466854	50.7	-
neg_15556	Phenobarbital N-Glucoside	metab_46783	B(ii)	0	46.1	HMDB0256406	M-H	C18H22N2O9	4.0668	-	neg	409.12889569525	0.006552892107489	46	-
neg_15579	Val-Ser-Leu	metab_46806	B(i)	49.1	0	-	M-H2O-H	C14H27N3O5	4.0561166666667	-	neg	298.17737784293	0.012275697558219	48.6	-
neg_15580	Glu-Val	metab_46807	B(i)	76.3	0	HMDB0028832	M-H2O-H	C10H18N2O5	4.0561166666667	-	neg	227.1038449	0.029131341643991	54.2	5879/6/1
neg_15583	Becatecarin	metab_46810	B(ii)	0	46.3	HMDB0248919	M+Cl	C33H34Cl2N4O7	4.0508166666667	-	neg	703.15542677178	0.025650765271769	38.3	-
neg_15605	Pantetheine	metab_46832	B(ii)	0	65	HMDB0003426	M-H	C11H22N2O4S	4.0454666666667	C00831	neg	277.12286256741	0.01308025830266	52.1	496-65-1
neg_15610	S-(9-Hydroxy-Pga1)-Glutathione	metab_46837	B(ii)	0	72.6	HMDB0013059	M-H2O-H	C30H51N3O10	4.0401166666667	-	neg	626.31572622189	0.008636663732153	51.5	-
neg_15622	Val-Val-Tyr	metab_46849	B(i)	75.1	0	-	M-H	C19H29N3O5	4.0347666666667	-	neg	378.20366113674	0.024171621676893	53.6	-
neg_15631	Cyproteron	metab_46858	B(ii)	0	71.8	HMDB0250698	M+Na-2H	C22H27ClO3	4.0294333333333	-	neg	395.13982511304	0.083985333468848	46	-
neg_15648	Gly-Thr-Phe	metab_46875	B(i)	79.1	0	-	M-H	C15H21N3O5	4.0187833333333	-	neg	322.14107165842	0.032237141951997	53.6	-
neg_15650	N-[4'-Hydroxy-(E)-Cinnamoyl]-L-Aspartic Acid	metab_46877	B(ii)	0	42	HMDB0029234	M-H	C13H13NO6	4.0187833333333	-	neg	278.06721	0.082523288844391	47.4	-
neg_15656	Bisphenol B	metab_46883	B(ii)	0	43.1	HMDB0245327	2M+FA-H	C16H18O2	4.0134333333333	C14225	neg	529.26299909985	0.043034448702289	45.9	77-40-7;_
neg_15661	5'-Deoxyadenosine	metab_46888	B(ii)	0	41.5	HMDB0001983	M-H2O-H	C10H13N5O3	4.0134333333333	C05198	neg	232.08279518389	0.072612163017762	45.5	4754-39-6
neg_15674	Senktide	metab_46901	B(ii)	0	69.6	HMDB0258234	M+FA-H	C40H55N7O11	4.0025833333333	-	neg	886.35897440764	0.046028133388303	49	-
neg_15705	Phorbol-12,13-Dibutyrate	metab_46932	B(ii)	0	40.1	HMDB0246702	M+Cl	C28H40O8	3.9919333333333	C03634;_	neg	539.24492251496	0.013285142740439	44.6	37558-16-0;_
neg_15711	Prenyl Arabinosyl-(1->6)-Glucoside	metab_46938	B(ii)	0	49.7	HMDB0041360	M+Hac-H	C16H28O10	3.9919333333333	-	neg	439.18141231405	0.045378125054083	47.5	175737-84-5
neg_15733	Leu-Ile-Thr	metab_46960	B(i)	52.8	0	-	M-H	C16H31N3O5	3.98125	-	neg	344.21913817211	0.02886517260118	49	-
neg_15764	Glu-Leu-Ser	metab_46991	B(i)	92.4	0	-	M-H2O-H	C14H25N3O7	3.97055	-	neg	328.1515724	0.009969309141663	57.5	-

neg_15779	Gln-Thr-Ile	metab_47006	B(i)	64.7	0	-	M-H	C15H28N4O6	3.96525	-	neg	359.19372608204	0.02306720326087	52	-
neg_15787	Cgp71422	metab_47014	B(ii)	0	62.8	HMDB0013864	M-H2O-H	C29H31N7O2	3.95995	-	neg	490.23428268772	0.034494362166969	49	-
neg_15788	Tyrosyl-Glycyl-Glycyl-Phenylalanyl	metab_47015	B(ii)	0	71.2	HMDB0247121	M+FA-H	C22H26N4O6	3.95995	-	neg	487.18173369236	0.043206368457746	50.7	-
neg_15792	Leu-Glu-Asp	metab_47019	B(i)	59.4	0	-	M-H2O-H	C15H25N3O8	3.95995	-	neg	356.14631391171	0.050279598230063	48.3	-
neg_15793	Xanthurenic Acid	metab_47020	B(i)	98.3	0	HMDB0000881;PW_C000703	M-H	C10H7NO4	3.95995	C02470	neg	204.03035819497	0.012758163821903	58.9	59-00-7
neg_15799	Latrunculin A	metab_47026	B(ii)	0	59.4	HMDB0253989	M+Hac-H	C22H31NO5S	3.9546	C16901;_	neg	480.2102912	0.066372675715288	45.6	76343-93-6;_
neg_15801	Glu-Phe-Gln	metab_47028	B(i)	93.7	0	-	M-H	C19H26N4O7	3.9546	-	neg	421.1731334	0.014606944734768	54.3	-
neg_15802	Tyr-Gly-Tyr	metab_47029	B(i)	67.2	0	-	M-H	C20H23N3O6	3.9546	-	neg	400.15158451982	0.031987431189883	52.5	-
neg_15803	L-Prolinamide, 5-Oxo-L-Prolyl-L-Norvalyl-	metab_47030	B(ii)	0	47.5	HMDB0253911	M+FA-H	C15H24N4O4	3.9546	-	neg	369.1781538	0.007321710506977	48.6	-
neg_15808	4-Acetamidobutanoic Acid	metab_47035	B(ii)	0	44.7	HMDB0003681	2M-H	C6H11NO3	3.9546	C02946	neg	289.14068499047	0.064510578175879	48	3025-96-5
neg_15812	Prednisolone Phthalate	metab_47039	B(ii)	0	52.8	HMDB0256749	M+Hac-H	C29H30O9	3.9492666666667	-	neg	581.20374267806	0.049207299323949	48	-
neg_15813	Asparaginylglutamine	metab_47040	B(ii)	0	44.3	HMDB0028729	2M+FA-H	C9H16N4O5	3.9492666666667	-	neg	565.22645346592	0.055693135276362	44.7	14608-83-4
neg_15839	Hydrangenoisde A	metab_47066	B(i)	74.5	0	-	M-H	C31H40O13	3.9332333333333	-	neg	619.23713727331	0.005830154002628	51.8	-
neg_15842	Glu-Thr-Tyr	metab_47069	B(i)	88.3	0	-	M-H2O-H	C18H25N3O8	3.9332333333333	-	neg	392.14702804266	0.042783415590805	55.2	-
neg_15851	Val-Pro-Pro	metab_47078	B(ii)	0	44.4	HMDB0253034	M+FA-H	C15H25N3O4	3.9279333333333	-	neg	356.18288979883	0.010456675669551	48.1	-
neg_15852	Toxin T2 Tetrol	metab_47079	B(ii)	0	44.5	HMDB0036159	M+FA-H	C15H22O6	3.9279333333333	-	neg	343.14132322965	0.032883405684459	46.8	34114-99-3
neg_15858	5-(N-Hexadecanoyl)Aminofluorescin	metab_47085	B(ii)	0	58.6	HMDB0246734	M-H	C36H43NO6	3.9226333333333	-	neg	584.30517078022	0.004227669465080	48	-
neg_15862	2-Methyl-N-(2-Morpholino-3-Pyridinyl)-6-(Trifluoromethyl)Nicotinamide	metab_47089	B(i)	43	0	-	M+FA-H	C17H17F3N4O2	3.9226333333333	-	neg	411.1275179	0.05049225845868	44.6	-
neg_15878	Pro-Leu-Ser	metab_47105	B(i)	70.6	0	-	M-H	C14H25N3O5	3.9172833333333	-	neg	314.17233778058	0.004727097581130	52.8	-
neg_15881	Alanyltryptophan	metab_47108	B(ii)	0	54.1	HMDB0013209	2M+FA-H	C14H17N3O3	3.9119166666667	-	neg	595.25038770193	0.027572264222664	45.8	16305-75-2
neg_15890	N-[(3A,5B,7A,12A)-3,12-Dihydroxy-24-Oxo-7-(Sulfooxy)Cholan-24-Yl]-Glycine	metab_47117	B(ii)	0	61.2	HMDB0002640	M+FA-H	C26H43NO9S	3.9065666666667	-	neg	590.2594362	0.076196463205378	48.3	71977-73-6
neg_15892	(5Alpha,6Beta,14Alpha,20R,22R)-5,6,14,20,27-Pentahydroxy-1-Oxowith-24-Enolide	metab_47119	B(ii)	0	56.8	HMDB0033198	M+Na-2H	C28H42O8	3.9065666666667	-	neg	527.26287598235	0.0083496297667816	50.7	222609-97-4
neg_15894	7,8-Dihydroneopterin	metab_47121	B(ii)	0	47.2	HMDB0002275	M+Cl	C9H13N5O4	3.9065666666667	C04874	neg	290.06478619619	0.037685977985732	47.4	-;1218-98-0
neg_15895	N-Acetyl-L-Tyrosine	metab_47122	B(i)	99.2	0	HMDB0000866;HMDB0244966	M-H	C11H13NO4	3.9065666666667	_;C01657	neg	222.0772943	0.005553855421905	59.2	537-55-3;_
neg_15899	Deoxynivalenol	metab_47126	B(ii)	0	40.2	HMDB0036156	2M+FA-H	C15H20O6	3.9012166666667	C09747	neg	637.24792514397	0.25583581956443	43	51481-10-8
neg_15908	Fluprednisolone	metab_47135	B(ii)	0	61.1	HMDB0252404	M+FA-H	C21H27FO5	3.8958666666667	C14618;_	neg	423.18546441652	0.018067017514233	44.6	53-34-9;_
neg_15911	Actein	metab_47138	B(ii)	0	59.3	HMDB0247977	M-H2O-H	C37H56O11	3.8905333333333	-	neg	657.35800896725	0.16569589718063	46.8	-
neg_15919	Hydroxy Ritonavir	metab_47146	B(ii)	0	64.4	HMDB0061044	M+Na-2H	C37H48N6O6S	3.8852	-	neg	757.27946943467	0.022995343707344	48.2	-
neg_15921	Absinthin	metab_47148	B(ii)	0	51.1	HMDB0035742	M+Na-2H	C30H40O6	3.8852	C09286	neg	517.25402069579	0.06258229108155	45.8	1362-42-1
neg_15927	Val-Ile	metab_47154	B(i)	94.9	0	HMDB0029130	M-H	C11H22N2O3	3.8852	-	neg	229.15588199676	0.006795729390799	58.3	20556-14-3
neg_15942	Anthroylouabain	metab_47169	B(ii)	0	57.6	HMDB0248471	M-H	C44H52O13	3.8745333333333	-	neg	787.33861687703	0.004452129061620	48	-
neg_15947	Argininosuccinic Acid	metab_47174	B(ii)	0	59.9	HMDB0000052	2M-H	C10H18N4O6	3.8745333333333	C03406	neg	579.24266630969	0.020440423329841	47.8	2387-71-5
neg_15950	Beta-Acetyldigoxin	metab_47177	B(ii)	0	74.5	HMDB0249095	M+Cl	C43H66O15	3.86925	C22190;_	neg	857.41260361523	0.007852703995421	51.5	-;_
neg_15957	Forskolin	metab_47184	B(ii)	0	54	HMDB0252457	M-H2O-H	C22H34O7	3.86925	_;C09076	neg	391.20890190338	0.009660868506962	47.4	66575-29-9;_;
neg_15962	Andersoline	metab_47189	B(ii)	0	47.4	HMDB0255817	M+Na-2H	C38H50N2O11	3.86395	-	neg	731.31185615587	0.01793471039993	45.9	-
neg_15966	Paramethasone Acetate	metab_47193	B(i)	41.8	0	-;HMDB0256122;LMST02030123	M+Hac-H	C24H31FO6	3.86395	_;C12656	neg	493.22581037009	0.10151416227038	46.5	1597-82-6;_;
neg_15973	Arbutin	metab_47200	B(ii)	0	45.3	HMDB0029943	M+Na-2H	C12H16O7	3.86395	C06186	neg	293.06440866756	0.14670437566922	47.4	497-76-7
neg_15975	4-Hydroxyphenyllactic Acid	metab_47202	B(i)	97.4	0	HMDB0000755	M-H	C9H10O4	3.86395	C03672	neg	181.05069299113	0.013493951479258	59.2	306-23-0
neg_15976	Phenylacetic Acid	metab_47203	B(i)	50.4	0	HMDB0000209;PW_C000135	M-H	C8H8O2	3.86395	C07086	neg	135.04529043042	0.030963879478302	48.2	103-82-2
neg_15995	Leu Ile Thr Arg	metab_47222	B(i)	88.8	0	-	M-H	C22H43N7O6	3.8532666666667	-	neg	500.32058064488	0.040381613275626	55.9	-
neg_15997	Tyr-Asn-Lys	metab_47224	B(i)	72.5	0	-	M+FA-H	C19H29N5O6	3.8532666666667	-	neg	468.20730578556	0.0127378012168	51.4	-
neg_15998	Ala Leu Val Arg	metab_47225	B(i)	60	0	-	M-H	C20H39N7O5	3.8532666666667	-	neg	456.29356284046	0.10449371346334	49.2	-
neg_16003	4-[2-[[2-(4-Hydroxyphenyl)Acetyl]Amino]Ethylamino]-4-Oxobut-2-Enoic Acid	metab_47230	B(i)	52.1	0	-	M-H	C14H16N2O5	3.8532666666667	-	neg	291.09876836687	0.0082484963481853	49.6	-

neg_16007	N,N-Diallyl-Tyrosyl-Aminoisobutyryl-Aminoisobutyryl-Phenylalanyl-Leucine	metab_47234	B(ii)	0	81.9	HMDB0253352	M-H	C38H53N5O7	3.8479166666667	-	neg	690.38412001907	0.011494539589433	53.2	-
neg_16012	Ile-Ile-Ser	metab_47239	B(i)	88.8	0	-	M-H	C15H29N3O5	3.8479166666667	-	neg	330.20363711129	0.005299041744859	56.4	-
neg_16013	Alpha-Carissanol	metab_47240	B(ii)	0	51.6	HMDB0035597	M+Na-2H	C15H24O3	3.8479166666667	-	neg	273.14571022761	0.016793274825281	47.6	99957-09-2
neg_16015	Gibberellin A34	metab_47242	B(ii)	0	63	HMDB0303448	2M+FA-H	C19H24O6	3.8425666666667	C11868;_	neg	741.31942291354	0.04604969081621	48.6	32630-92-5;_;
neg_16018	Neomycin	metab_47245	B(ii)	0	77.4	HMDB0015129	M+Hac-H	C23H46N6O13	3.8425666666667	C00384;C01737;_	neg	673.33182845935	0.016181946749474	51.8	1404-04-2;_;119-04-0
neg_16022	Hydroxydesmthyl Doxepin Glucuronide	metab_47249	B(ii)	0	52.4	HMDB0060629	M-H	C25H29NO8	3.8425666666667	-	neg	470.17857279862	0.077084437161304	46	-
neg_16023	Syn-Copalyl Diphosphate	metab_47250	B(ii)	0	54.2	HMDB0301829	M-H2O-H	C20H36O7P2	3.8425666666667	C11902	neg	431.17834062396	0.061927376964579	47.8	-;_;
neg_16024	Asp-Glu-Leu	metab_47251	B(i)	89.3	0	-	M-H	C15H25N3O8	3.8425666666667	-	neg	374.15707924192	0.02869920873609	55.4	-
neg_16031	Gluten Exorphin A5	metab_47258	B(ii)	0	81.4	HMDB0059793	M-H	C29H37N5O9	3.8369333333333	-	neg	598.2481814	0.1179048347066	52.4	-
neg_16043	Capsianoside V	metab_47270	B(ii)	0	49.7	HMDB0030737	M+Na-2H	C26H42O10	3.8316	-	neg	535.2524323	0.029102400767731	48.9	121924-06-9
neg_16062	Val-Phe-Asp	metab_47289	B(i)	48.7	0	-	M-H	C18H25N3O6	3.8262333333333	-	neg	378.16673212126	0.043035758984755	48.5	-
neg_16066	Licoricesaponin F3	metab_47293	B(ii)	0	87.9	HMDB0039490	M+Cl	C48H72O19	3.8208833333333	-	neg	987.42915566292	0.053801068593538	53.8	-
neg_16072	Physapruin A	metab_47299	B(ii)	0	46.4	HMDB0303754	M+FA-H	C28H38O7	3.8208833333333	-	neg	531.25725865518	0.091896729998055	45.6	-
neg_16073	Cinncassiol D1 Glucoside	metab_47300	B(ii)	0	47.4	HMDB0034677	M-H	C26H42O10	3.8208833333333	-	neg	513.26800829777	0.031871719298006	43	-
neg_16091	Endoxifen	metab_47318	B(ii)	0	40.7	HMDB0060666	M+Cl	C25H27NO2	3.8155333333333	C16547	neg	408.17415040767	0.063508572831869	46.1	112093-28-4;_
neg_16095	Gln-Ser-Ile	metab_47322	B(i)	87.8	0	-	M-H	C14H26N4O6	3.8155333333333	-	neg	345.17794938055	0.005531933996180	56.6	-
neg_16099	5-Hydroxy-L-Tryptophan	metab_47326	B(ii)	0	51.6	HMDB0000472	M+Hac-H	C11H12N2O3	3.8155333333333	C00643	neg	279.09875861381	0.020660316095973	50	4350/9/8;4350-09-8
neg_16104	12-O-D-Glucuronoside-13-Hydroxyoctadec-9Z-Enoate	metab_47331	B(ii)	0	41.5	HMDB0060119	M-H2O-H	C24H42O10	3.8102	-	neg	471.25742562052	0.01931315276598	46.1	-
neg_16110	2-Methylthio-N6-(Delta2-Isopentenyl)Adenosine	metab_47337	B(ii)	0	54.9	HMDB0301782	2M+FA-H	C16H23N5O4S	3.80485	-	neg	807.29319863723	0.03031298383119	48.2	20859-00-1
neg_16121	Asp-Gly-Leu	metab_47348	B(i)	82.3	0	-	M-H	C12H21N3O6	3.80485	-	neg	302.136001	0.084839012445586	54	-
neg_16128	Gln-Phe-Thr	metab_47355	B(i)	91.9	0	-	M-H	C18H26N4O6	3.7995166666667	-	neg	393.17804256854	0.008826022723775	57.9	-
neg_16132	Lpa(3:0)	metab_47359	B(i)	47.4	0	-	M+FA-H	C6H13O7P	3.7995166666667	-	neg	273.03816903898	0.080107667654069	49.1	-
neg_16134	Candidin	metab_47361	B(ii)	0	97.4	HMDB0259425	M+Cl	C47H71NO17	3.7941833333333	-	neg	956.44613451581	0.013217419358288	56.5	-
neg_16149	6-Methylcoumarin	metab_47376	B(ii)	0	69.9	HMDB0032394	M+FA-H	C10H8O2	3.7941833333333	-	neg	205.0507388	0.032051533186932	53.2	92-48-8
neg_16161	Ile-Val-Asn	metab_47388	B(i)	51.6	0	-	M-H	C15H28N4O5	3.7888166666667	-	neg	343.19884033871	0.042659820867601	48	-
neg_16169	Glycyl-Prolyl-Arginyl-Valyl-Valyl-Glutamic Acid	metab_47396	B(ii)	0	68.4	HMDB0252831	M+Na-2H	C28H49N9O9	3.7834666666667	-	neg	676.34252749022	0.044167634545246	51.6	-
neg_16176	Nidurufin	metab_47403	B(i)	39.3	0	-	M+Cl	C20H16O8	3.7834666666667	-	neg	419.05487941993	0.005610981885844	46.1	-
neg_16186	2-(4-Chlorophenoxy)Propionic Acid	metab_47413	B(ii)	0	50.3	HMDB0244931	M-H2O-H	C9H9ClO3	3.7834666666667	C13701	neg	181.00627580794	0.011271122820273	44.7	3307-39-9;_
neg_16192	Benzoylaconine	metab_47419	B(ii)	0	63	HMDB0249045	M-H	C32H45NO10	3.7781166666667	-	neg	602.29446439867	0.006500571538223	49.2	-
neg_16193	Gomisin D	metab_47420	B(ii)	0	57.7	HMDB0252911	M+FA-H	C28H34O10	3.7781166666667	C17816;_	neg	575.21431985743	0.080638295903663	49.1	60546-10-3;_
neg_16194	Geneticin	metab_47421	B(ii)	0	74.9	HMDB0242565	M+Hac-H	C20H40N4O10	3.7781166666667	_;C17703	neg	555.2899127	0.013982453783298	53.1	49863-47-0;_
neg_16210	Prostaglandin Pge2 1-Glyceryl Ester	metab_47437	B(ii)	0	66.2	HMDB0013043	M+Hac-H	C23H38O7	3.7674666666667	-	neg	485.273267	0.036055785293143	50.9	-
neg_16218	Leukotriene D5	metab_47445	B(ii)	0	47.2	HMDB0012994	M+Hac-H	C25H38N2O6S	3.7621333333333	-	neg	553.26070993655	0.096754389717643	45.3	_;
neg_16243	Pyripyropene A	metab_47470	B(ii)	0	48.6	HMDB0256984	M+Cl	C31H37NO10	3.7514166666667	-	neg	618.21663380771	0.054688306495233	44	-
neg_16272	Glu-Met	metab_47499	B(i)	80.2	0	HMDB0028825	M-H2O-H	C10H18N2O5S	3.7407333333333	-	neg	259.07578747567	0.020138323897952	54.8	4423-22-7
neg_16286	Phenolphthalin	metab_47513	B(i)	50	0	-	M-H	C20H16O4	3.7354166666667	C14223	neg	319.09706303824	0.034508323745401	45.6	81-90-3
neg_16305	Glu-Gln-Leu	metab_47532	B(i)	74.3	0	-	M-H	C16H28N4O7	3.7301166666667	-	neg	387.18871253192	0.013476658571283	50.5	-
neg_16308	Tyr-Ala-Ile	metab_47535	B(i)	36.7	0	-	M-H	C18H27N3O5	3.7301166666667	-	neg	364.18792502601	0.066545061359619	45.3	-
neg_16311	Tyrosyl-Threonine	metab_47538	B(ii)	0	64.2	HMDB0029115	M+Na-2H	C13H18N2O5	3.7301166666667	-	neg	303.09648841875	0.011452338096138	51.4	-
neg_16315	Morph	metab_47542	B(ii)	0	51.3	HMDB0254890	M-H	C10H16N2O4	3.7301166666667	C01516;_	neg	227.10381952984	0.008690196131926	49.8	57-27-2;_
neg_16317	Pentaerythritol Mononitrate	metab_47544	B(ii)	0	45.1	HMDB0256252	M+Na-2H	C5H11NO6	3.7301166666667	-	neg	202.03344878841	0.0855320190771	48.5	-
neg_16330	Ala-Glu-Ile	metab_47557	B(i)	54.7	0	-	M-H	C14H25N3O6	3.72475	-	neg	330.16732147181	0.014596884033966	49.7	-
neg_16336	Beta-Hydroxyisovaleric Acid	metab_47563	B(i)	92.3	0	HMDB0000754	M-H	C5H10O3	3.72475	-	neg	117.05580838596	0.012076109274932	58.1	625-08-1
neg_16345	Cyclosquamosin A	metab_47572	B(ii)	0	83	HMDB0303367	M-H2O-H	C31H43N7O8	3.7141166666667	-	neg	622.294691	0.026480296473622	52.6	-

neg_16346	Crocin 4	metab_47573	B(ii)	0	47	HMDB0039122	M+Hac-H	C27H36O9	3.7141166666667	C00744	neg	563.24722653146	0.024370451510932	47.4	55750-86-2
neg_16353	Val-Val-Pro	metab_47580	B(i)	37.2	0	-	M-H	C15H27N3O4	3.7141166666667	-	neg	312.19302519439	0.04275671995873	46	-
neg_16377	Folcepri	metab_47604	B(ii)	0	79.8	HMDB0251680	M+Hac-H	C29H35N11O1 1S	3.6981	-	neg	804.24432532629	0.15759370718068	51.5	-
neg_16393	Adenine	metab_47620	B(i)	92	0	HMDB0000034;PW_C000024	M-H	C5H5N5	3.6981	C00147	neg	134.04734358309	0.051449960118179	56.8	73-24-5
neg_16398	Vomifoliol 9-[Glucosyl-(1->4)-Xylosyl-(1->6)-Glucoside]	metab_47625	B(ii)	0	74.8	HMDB0038449	M+Na-2H	C30H48O17	3.69275	-	neg	701.25752260867	0.038901292490048	50.5	141947-49-1
neg_16402	Tirofiban	metab_47629	B(ii)	0	52.4	HMDB0014913	M-H	C22H36N2O5S	3.69275	C07965	neg	439.23130739102	0.028561877805854	45.6	144494-65-5
neg_16404	Ile-Leu-Ser	metab_47631	B(i)	92.2	0	-	M-H	C15H29N3O5	3.69275	-	neg	330.20362369962	0.024712297039896	57.4	-
neg_16412	Cucumerin B	metab_47639	B(ii)	0	53.3	HMDB0301966	M+Na-2H	C29H28O11	3.6874	-	neg	573.13668883089	0.015334976297579	47.9	_;
neg_16415	Ppack	metab_47642	B(ii)	0	47.1	HMDB0250787	M+Hac-H	C21H31ClN6O	3.6874	-	neg	509.22549301539	0.086471281123709	41.9	-
neg_16418	{1-[2-(4-Carbamimidoyl-Benzoylamino)-Propionyl]-Piperidin-4-Yloxy}-Acetic Acid	metab_47645	B(ii)	0	50.2	HMDB0257277	M+FA-H	C18H24N4O5	3.6874	-	neg	421.17071167271	0.025999579887587	47.4	-
neg_16421	Gly-Glu-Ile	metab_47648	B(i)	82.4	0	-	M-H	C13H23N3O6	3.6874	-	neg	316.15154779588	0.042245196244002	55.2	-
neg_16422	Leu-Tyr	metab_47649	B(i)	90.7	0	HMDB0028941	M-H	C15H22N2O4	3.6874	-	neg	293.15088199539	0.011557436170045	57.3	-
neg_16425	Inolin	metab_47652	B(ii)	0	42.3	HMDB0253488	2M+FA-H	C19H23NO5	3.6820333333333	-	neg	735.30730918949	0.030447055386997	44.7	-
neg_16428	Artecanin	metab_47655	B(ii)	0	53.4	HMDB0302700	2M+FA-H	C15H18O5	3.6820333333333	C09302;C09354	neg	601.22787938611	0.031439860042592	48.7	29431-84-3;_
neg_16430	3,3'-Dimethoxybenzidine	metab_47657	B(ii)	0	51.1	HMDB0246003	2M+FA-H	C14H16N2O2	3.6820333333333	C19231	neg	533.2401774	0.008665031070304	48.5	119-90-4;_
neg_16431	5-Methyltetrahydrofolic Acid	metab_47658	B(ii)	0	47.9	HMDB0001396	M+Hac-H	C20H25N7O6	3.6820333333333	C00440	neg	518.1997153	0.047644814861534	48.3	134-35-0
neg_16432	Edulisin I	metab_47659	B(ii)	0	75.8	HMDB0030594	M-H	C28H26O8	3.6820333333333	-	neg	489.15813555028	0.050909108659161	50	96608-82-1
neg_16436	3-(Methylthio)Propyl Acetate	metab_47663	B(ii)	0	54.3	HMDB0031717	M+FA-H	C6H12O2S	3.6820333333333	-	neg	193.05422014529	0.009875602582813	50	16630-55-0
neg_16443	Methyl (3X,10R)-Dihydroxy-11-Dodecene-6,8-Diynoate 10-	metab_47670	B(ii)	0	45.6	HMDB0040896	M+Hac-H	C19H26O9	3.6767333333333	-	neg	457.17296184407	0.028801504379877	47.2	152141-43-0
neg_16446	Gly-Phe	metab_47673	B(i)	98.1	0	HMDB0028848	M-H	C11H14N2O3	3.6767333333333	-	neg	221.09328766242	0.015156072062016	58.9	-
neg_16458	Afn911	metab_47685	B(ii)	0	56.3	HMDB0013863	M-H2O-H	C29H33N7O2	3.6711833333333	-	neg	492.25636510159	0.008563905690458	46.1	-
neg_16477	Phe-Ser-Asn	metab_47704	B(i)	46.6	0	-	M-H	C16H22N4O6	3.6658333333333	-	neg	365.14600064495	0.033963339950964	47.4	-
neg_16478	Glutaminylphenylalanine	metab_47705	B(ii)	0	59.7	HMDB0028804	M-H	C14H19N3O4	3.6658333333333	-	neg	292.13043473923	0.019983403912864	50.8	92327-75-8
neg_16491	4'-Demethylepipodophyllotoxin	metab_47718	B(ii)	0	45.4	HMDB0246627	M+Na-2H	C21H20O8	3.6604833333333	C21185;_	neg	421.08660808845	0.047570449319996	45	6559-91-7;_
neg_16492	2-Hydroxy-3-Methyl-4H-Pyran-4-One O-(6E-Cinnamoyl-B-D-Glucoside)	metab_47719	B(ii)	0	70.3	HMDB0041098	M-H2O-H	C21H22O9	3.6604833333333	-	neg	399.1047509	0.014515790162713	50.7	84888-58-4
neg_16503	Verbasoside	metab_47730	B(i)	45.7	0	HMDB0039233	M-H	C20H30O12	3.6551833333333	-	neg	461.16709815372	0.025134101446071	47.4	61548-34-3
neg_16505	Asn-Glu-Leu	metab_47732	B(i)	82.6	0	-	M-H	C15H26N4O7	3.6551833333333	-	neg	373.17298620999	0.020097523407032	55.3	-
neg_16509	Rpr123142	metab_47736	B(ii)	0	79.5	HMDB0060871	M+Na-2H	C44H55NO14	3.6498333333333	-	neg	842.33274834067	0.018157520806136	53.1	-
neg_16516	Phe-Asp-Gln	metab_47743	B(i)	57.3	0	-	M-H	C18H24N4O7	3.6498333333333	-	neg	407.1560295	0.091388166897886	49.4	-
neg_16518	Picrocrocin	metab_47745	B(ii)	0	55.9	HMDB0005796	M+Cl	C16H26O7	3.6498333333333	C17055;_	neg	365.13430158637	0.004804207346548	47.1	138-55-6
neg_16519	Phe-Ala-Thr	metab_47746	B(i)	91.3	0	-	M-H	C16H23N3O5	3.6498333333333	-	neg	336.15670734322	0.0228512708162	57.2	-
neg_16541	2,5-Dihydroxy-1-Octadec-9-Enoyloxypyrrole-3-Sulfonic Acid	metab_47768	B(ii)	0	61.3	HMDB0255399	M+Na-2H	C22H37NO7S	3.63915	-	neg	480.20767737531	0.019889791097363	47.7	-
neg_16564	4-(2,2,6,6-Tetramethyl-1-Oxylpiperidin-4-Ylamino)-4'-Demethylepipodophyllotoxin	metab_47791	B(ii)	0	43.8	HMDB0252920	M-H	C30H38N2O8	3.6284666666667	-	neg	553.2605376	0.005941515770645 6	43.8	-
neg_16568	Asn-Phe	metab_47795	B(i)	89.3	0	HMDB0028738	M-H	C13H17N3O4	3.6284666666667	-	neg	278.11475027718	0.011520573208673	56.9	-
neg_16577	6-O-Oleuropeoylsucrose	metab_47804	B(ii)	0	43.1	HMDB0038117	M+Na-2H	C22H36O13	3.6231166666667	-	neg	529.19420153421	0.01119059138608	46	17651-05-7
neg_16579	Isorhynchophylline	metab_47806	B(ii)	0	58.7	HMDB0253688	M+FA-H	C22H28N2O4	3.6231166666667	C16980;_	neg	429.19932673203	0.027672333547703	47.2	6859/1/4;_
neg_16581	Ketotrilostane	metab_47808	B(ii)	0	40.9	HMDB0253794	M+Na-2H	C20H25NO3	3.6231166666667	-	neg	348.15997008731	0.007648231038617	45	-
neg_16584	Ile-Pro	metab_47811	B(i)	50.4	0	HMDB0011174	M-H	C11H20N2O3	3.6231166666667	-	neg	227.14021874541	0.006179947923067	49.3	37462-92-3
neg_16590	Alliosterol 1-Rhamnoside 16-Galactoside	metab_47817	B(ii)	0	51.9	HMDB0038345	M+FA-H	C39H66O13	3.6177666666667	-	neg	787.44731303902	0.02939644812087	48.9	289690-78-4
neg_16597	Quassimarin	metab_47824	B(ii)	0	63.7	HMDB0036888	M-H2O-H	C27H36O11	3.6177666666667	C08777	neg	517.20941641078	0.021925182762687	50	59938-97-5
neg_16605	Leu-Thr-Pro	metab_47832	B(i)	88.2	0	-	M-H	C15H27N3O5	3.6177666666667	-	neg	328.18792204229	0.021661814416079	55.5	-
neg_16622	Tyr-Tyr	metab_47849	B(i)	96.7	0	HMDB0029117	M-H	C18H20N2O5	3.6124833333333	-	neg	343.1300921	0.017501704818577	58.3	-
neg_16631	Fexaramine	metab_47858	B(ii)	0	46.2	HMDB0252239	M+Na-2H	C32H36N2O3	3.6071333333333	C15649;_	neg	517.25086795501	0.030884050151359	46.4	574013-66-4;_

neg_16640	Enkephalinamide-Leu, Ala(2)-	metab_47867	B(ii)	0	57.9	HMDB0244970	M-H2O-H	C29H40N6O6	3.6018333333333	-	neg	549.28875446224	0.079992419849403	45.9	-
neg_16655	Ala-Ala-Ile	metab_47882	B(i)	55.7	0	-	M-H	C12H23N3O4	3.5965333333333	-	neg	272.1617815	0.018273614145912	50	-
neg_16665	Glu-Gln-Ile	metab_47892	B(i)	82.6	0	-	M-H	C16H28N4O7	3.5912333333333	-	neg	387.1888753	0.034528960310906	54.4	-
neg_16667	Tryptophyl-Threonine	metab_47894	B(ii)	0	53.1	HMDB0029093	M+Hac-H	C15H19N3O4	3.5912333333333	-	neg	364.15144214516	0.007744716353141	50.3	-
neg_16671	S-(Pga1)-Glutathione	metab_47898	B(ii)	0	76.2	HMDB0013061	M+FA-H	C30H49N3O10	3.5859166666667	C11304;_	neg	688.3162139	0.038443194469286	52.5	-;_
neg_16674	Gimatecan	metab_47901	B(ii)	0	52.9	HMDB0252718	M+Na-2H	C25H25N3O5	3.5859166666667	-	neg	468.15601364675	0.10125709834439	46.6	-
neg_16676	26-(2-Glucosyl-6-Acetylglucosyl]-1,3,11,22-Tetrahydroxyergosta-5,24-Dien-26-Oate	metab_47903	B(ii)	0	69.3	HMDB0040406	M+Hac-H	C42H66O17	3.5805666666667	-	neg	901.45166739669	0.032435588184582	49.7	155510-76-2
neg_16678	Lnaps(3:0/N-22:2)	metab_47905	B(i)	84.8	0	-	M+FA-H	C31H56NO10P	3.5805666666667	-	neg	678.35628002996	0.09740455389041	52.4	-
neg_16684	Leu-Gln-Pro	metab_47911	B(i)	82.1	0	-	M-H	C16H28N4O5	3.5805666666667	-	neg	355.19884157879	0.011945999967398	55.1	-
neg_16700	Tyr-Met	metab_47927	B(i)	82.1	0	HMDB0029111	M-H	C14H20N2O4S	3.5752666666667	-	neg	311.10735511845	0.034226901558114	53.2	-
neg_16702	3-Methoxyphenol	metab_47929	B(i)	48.3	0	-	M-H	C7H8O2	3.5752666666667	-	neg	123.04526212295	0.20837709763283	48.4	-
neg_16703	Pi(6:0/16:2)	metab_47930	B(i)	68	0	-	M+FA-H	C31H55O13P	3.5699666666667	-	neg	711.3303067	0.051077798531212	48.8	-
neg_16720	C-Quens	metab_47947	B(ii)	0	46.1	HMDB0249505	M-H	C23H29ClO4	3.5646833333333	-	neg	403.16606954794	0.067511779362604	42.3	-
neg_16721	Leu-Pro-Glu	metab_47948	B(i)	69.5	0	-	M-H	C16H27N3O6	3.5646833333333	-	neg	356.18299738284	0.022842520177895	52.3	-
neg_16722	Arg-Phe	metab_47949	B(i)	96.6	0	HMDB0028716	M-H2O-H	C15H23N5O3	3.5646833333333	-	neg	302.16247480094	0.051018834489355	56.8	2047-13-4
neg_16723	2-[4-(3-Hydroxypropyl)-2-Methoxyphenoxy]-1,3-Propanediol 1-Glucoside	metab_47950	B(ii)	0	57.3	HMDB0040353	2M-H	C19H30O10	3.5593333333333	-	neg	835.35959565527	0.088964184424788	49	68340-35-2
neg_16752	Leu-Val-Asn	metab_47979	B(i)	45.2	0	-	M-H	C15H28N4O5	3.5487333333333	-	neg	343.19879466056	0.029351590911623	47.7	-
neg_16763	Lys Glu Thr Glu	metab_47990	B(i)	54.3	0	-	M-H	C20H35N5O10	3.5433666666667	-	neg	504.23142997618	0.008661069501894	50.4	-
neg_16768	Algestone	metab_47995	B(ii)	0	72.7	HMDB0248134	M+FA-H	C21H30O4	3.5433666666667	C06391;_	neg	391.20916107271	0.01807981282596	50.3	595-77-7;_;
neg_16771	4-(Beta-D-Glucopyranosyloxy)Phenylacetic Acid	metab_47998	B(i)	66.8	0	-	M-H	C14H18O8	3.5433666666667	-	neg	313.09300143758	0.056278925178459	51.8	-
neg_16776	Trypanothione Disulfide	metab_48003	B(ii)	0	54.8	HMDB0060521	M+Na-2H	C27H47N9O10 S2	3.5380833333333	C03170	neg	742.26415499393	0.090675083260692	47.3	96304-42-6;_
neg_16788	Oripavine	metab_48015	B(ii)	0	60.2	HMDB0030251	2M-H	C18H19NO3	3.5327333333333	C06175;_	neg	593.26107720665	0.12119392445064	49.7	467-04-9
neg_16791	(6R)-Folinic Acid	metab_48018	B(ii)	0	65.7	HMDB0247218	M+Na-2H	C20H23N7O7	3.5327333333333	C03479	neg	494.14105236415	0.012878476377988	51	-
neg_16793	Pregnanolone Sulfate	metab_48020	B(ii)	0	44.9	HMDB0240590	M+FA-H	C21H34O5S	3.5327333333333	-	neg	443.2145109	0.022132595326502	45	1477-67-4
neg_16807	Asp Leu Ala Glu	metab_48034	B(i)	39.8	0	-	M-H	C18H30N4O9	3.5273666666667	-	neg	445.19424392806	0.012474184378162	45.9	-
neg_16810	Thr-Val-Ile	metab_48037	B(i)	85.5	0	-	M-H	C15H29N3O5	3.5273666666667	-	neg	330.20363142554	0.004669051202195	55.6	-
neg_16820	Modecainide	metab_48047	B(ii)	0	41.4	HMDB0254822	M+FA-H	C22H28N2O3	3.5220833333333	-	neg	413.2049063	0.024118247320132	43.9	-
neg_16849	Glu-Tyr-Gln	metab_48076	B(i)	90.8	0	-	M-H2O-H	C19H26N4O8	3.5114	-	neg	419.15806142889	0.008326816191003	55.8	-
neg_16853	Trp-Asp	metab_48080	B(i)	87.4	0	HMDB0029079	M-H	C15H17N3O5	3.5114	-	neg	318.10985134178	0.024365518846211	56.2	-
neg_16860	(1Alpha,3Beta,20S,22R,24S,25S)-Pubescenin	metab_48087	B(ii)	0	61.1	HMDB0030011	M-H	C34H52O10	3.5059666666667	-	neg	619.34766499363	0.003934621781615	48.6	100217-93-4
neg_16865	Aldoxorubicin	metab_48092	B(ii)	0	82.4	HMDB0248126	M-H	C37H42N4O13	3.5006833333333	-	neg	749.27430827722	0.030015061759076	52.4	-
neg_16869	Lupex	metab_48096	B(ii)	0	62	HMDB0254216	M-H2O-H	C27H35N5O7S	3.5006833333333	-	neg	554.208446	0.026442771669171	49.5	-
neg_16872	Ac-(5H-Dibenzyl(A,D)Cycloheptene-10,11-Dihydroglycine-Leu-Asp-Ile-Ile-Trp)	metab_48099	B(ii)	0	75.7	HMDB0256174	M+Cl	C52H67N7O10	3.4953333333333	-	neg	984.45611371102	0.020949528040707	49.8	-
neg_16882	5-Methyldeoxycytidine	metab_48109	B(ii)	0	48.3	HMDB0002224	2M+FA-H	C10H15N3O4	3.4953333333333	C03592	neg	527.20844511817	0.031561780631694	46.1	838-07-3
neg_16894	Kynostatin 227	metab_48121	B(ii)	0	73.7	HMDB0253838	M+Na-2H	C35H45N5O6S	3.49005	-	neg	716.26082407976	0.066887684617082	48.1	-
neg_16903	Hydroxy-Lacosamide	metab_48130	B(ii)	0	51.6	HMDB0061146	M-H	C13H18N2O5	3.49005	-	neg	281.11209028874	0.018094628158076	46.9	-
neg_16918	Advantame	metab_48145	B(ii)	0	48.2	HMDB0248034	M-H	C24H30N2O7	3.4847	-	neg	457.19414308557	0.021897467685604	45.2	-
neg_16924	Leu-Val	metab_48151	B(i)	94.3	0	HMDB0028942	M-H	C11H22N2O3	3.4847	-	neg	229.15588519013	0.024484630492049	58.2	-
neg_16942	2-Oxoadipic Acid	metab_48169	B(i)	42.4	0	HMDB0000225	M+Hac-H	C6H8O5	3.4794	C00322	neg	219.05115067272	0.028402377678782	48.1	3184-35-8
neg_16944	Deacetylvinblastine	metab_48171	B(ii)	0	47.3	HMDB0250907	M-H	C44H56N4O8	3.4740666666667	-	neg	767.39508494148	0.021018686565534	44.4	-
neg_16946	Z-Vdvad-Fmk	metab_48173	B(ii)	0	46.8	HMDB0259976	M-H2O-H	C32H46FN5O1	3.4740666666667	-	neg	676.30630785722	0.006295614339476	43.3	-
neg_16952	Pro-Phe-Gln	metab_48179	B(i)	42.5	0	-	M-H	C19H26N4O5	3.4740666666667	-	neg	389.18677350456	0.037783472851854	44.3	-
neg_16954	Gly-Gln-Leu	metab_48181	B(i)	87.6	0	-	M-H	C13H24N4O5	3.4740666666667	-	neg	315.16747832468	0.019239202531114	56.4	-

neg_16965	Lys-Phe-Thr	metab_48192	B(i)	35.4	0	-	M+FA-H	C19H30N4O5	3.4687166666667	-	neg	439.21737045097	0.019675973045755	44	-
neg_16972	Rescinnamine	metab_48199	B(ii)	0	40.9	HMDB0015311	M+Na-2H	C35H42N2O9	3.4633833333333	C06540	neg	655.25843345671	0.081394237308469	43.1	24815-24-5
neg_16979	Rimiterol	metab_48206	B(ii)	0	54.2	HMDB0257235	2M+FA-H	C12H17NO3	3.4633833333333	-	neg	491.23601968384	0.034562797432567	47.9	-
neg_16987	Epimedin B	metab_48214	B(ii)	0	85.6	HMDB0251854	M-H2O-H	C38H48O19	3.4580333333333	-	neg	789.26760940575	0.06663244268458	51.1	_;
neg_17006	Des-Arg(9)-Bradykinin	metab_48233	B(ii)	0	71.6	HMDB0244716	M-H	C44H61N11O1	3.4526833333333	-	neg	902.44965581614	0.034502145203197	50.9	-
neg_17018	Homovanillic Acid Sulfate	metab_48245	B(i)	57.2	0	HMDB0011719	M-H	C9H10O7S	3.4526833333333	-	neg	261.00757238593	0.052085065135044	49.1	38339-06-9
neg_17020	Dermatan L-Iduronate	metab_48247	B(ii)	0	81.1	HMDB0060470	2M-H	C16H27NO11	3.4473333333333	C01490;_	neg	817.30214854688	0.05487068130646	48.8	-;_
neg_17023	Sandoricin	metab_48250	B(ii)	0	52.6	HMDB0037555	M+Na-2H	C31H40O11	3.4473333333333	-	neg	609.23443280694	0.016973780068921	45.7	133585-55-4
neg_17044	Cofaryloside	metab_48271	B(ii)	0	51	HMDB0035096	M-H	C26H42O10	3.4366833333333	-	neg	513.26809940402	0.051722099418488	47.4	63558-43-0
neg_17052	Selamectin	metab_48279	B(ii)	0	56.6	HMDB0242663	M+Na-2H	C43H63NO11	3.4313833333333	-	neg	790.42213956073	0.11142953716305	45.7	-
neg_17053	Gibberellin A36	metab_48280	B(ii)	0	53.6	HMDB0304362	2M-H	C20H26O6	3.4313833333333	C11862;_	neg	723.34188251829	0.083896930338804	48.3	38076-57-2;_;
neg_17059	Antcin B	metab_48286	B(ii)	0	75.4	HMDB0248458	M+Cl	C29H40O5	3.4313833333333	-	neg	503.2529819	0.020462527675933	50.2	-
neg_17063	Pro-Ile-Ser	metab_48290	B(i)	77.3	0	-	M-H	C14H25N3O5	3.4313833333333	-	neg	314.17232207275	0.050969714179422	52.1	-
neg_17067	Tetrapeptide	metab_48294	B(ii)	0	46	HMDB0258927	M+FA-H	C27H40N8O9	3.4260333333333	_;C01960	neg	665.29255531072	0.034658480205581	45.5	-;_
neg_17072	Acetyl-Ser-Asp-Lys-Pro	metab_48299	B(ii)	0	40.2	HMDB0247929	M+Na-2H	C20H33N5O9	3.4260333333333	-	neg	508.20526979248	0.017000826892072	44.9	-
neg_17074	Ala Asp Phe Asp	metab_48301	B(i)	44.6	0	-	M-H	C20H26N4O9	3.4260333333333	-	neg	465.16039621979	0.067168540994821	45.2	-
neg_17078	Enalapril	metab_48305	B(ii)	0	59.8	HMDB0014722	M+Na-2H	C20H28N2O5	3.4260333333333	_;C06977	neg	397.17091916253	0.021308121325934	49	75847-73-3;_
neg_17079	Chondroitin	metab_48306	B(ii)	0	59.2	HMDB0000629	M-H2O-H	H2O.[C14H21 NO11]n	3.4260333333333	C00401	neg	378.10315765313	0.12117964847002	31.2	-;9007-27-6
neg_17089	Glutamine Glutamate Aspartate	metab_48316	B(ii)	0	40.7	HMDB0252809	M+Na-2H	C19H27N5O15	3.4207	-	neg	586.13068153191	0.056899712306526	43.7	-
neg_17106	Trans-Zeatin	metab_48333	B(i)	68.3	0	HMDB0012204	2M-H	C10H13N5O	3.4153666666667	C00371	neg	437.21452747203	0.059738892675598	52	1637-39-4
neg_17112	Cyclo(D-Trp-D-Asp-Pro-D-Val-Leu)	metab_48339	B(ii)	0	84.9	HMDB0249361	M+FA-H	C31H42N6O7	3.4100333333333	-	neg	655.30588820482	0.020369339423285	52.9	-
neg_17114	Postin	metab_48341	B(ii)	0	60	HMDB0005772	M+Cl	C22H40N8O5	3.4100333333333	-	neg	531.27750659741	0.082790563462293	46.7	103745-46-6
neg_17121	Pantothenic Acid	metab_48348	B(i)	98.2	0	HMDB0000210;HMDB0250782;PW_C000136	M-H	C9H17NO5	3.4100333333333	C00864;_	neg	218.10347792619	0.011477715533922	59.2	79-83-4;_
neg_17136	Gly Ile	metab_48363	B(i)	93.6	0	-	M-H	C8H16N2O3	3.4046833333333	-	neg	187.10887209445	0.015796485895287	56.7	-
neg_17152	Noa-Asn-Apns-Dmt-Nh-Tbu	metab_48379	B(ii)	0	63.5	HMDB0255676	M+Na-2H	C36H45N5O7S	3.39405	-	neg	712.27723367543	0.031684125835556	47.3	-
neg_17155	Cyanidin 3-Rutinoside	metab_48382	B(ii)	0	40	HMDB0031458	M+Na-2H	C27H31O15+	3.39405	C08620;_	neg	616.1412882	0.072691105139464	45	28338-59-2;
neg_17160	7-Acetyllycopsamine	metab_48387	B(ii)	0	55	HMDB0302356	M+Na-2H	C17H27NO6	3.39405	C16751	neg	362.15698232429	0.033028390703279	47.9	-
neg_17163	Pro-Leu	metab_48390	B(i)	86.1	0	HMDB0253028	M-H	C11H20N2O3	3.39405	-	neg	227.14024328584	0.016761367092085	56.4	-
neg_17174	Ser-Ser-Ile	metab_48401	B(i)	90	0	-	M-H	C12H23N3O6	3.3887	-	neg	304.15156317488	0.022434728120419	56.6	-
neg_17204	Ala-Ile	metab_48431	B(i)	98.1	0	HMDB0028690	M-H	C9H18N2O3	3.37805	-	neg	201.12457911356	0.019516137255909	59.1	29727-65-9
neg_17217	Serotonin	metab_48444	B(ii)	0	41.1	HMDB0000259	M+FA-H	C10H12N2O	3.3727166666667	C00780	neg	221.0932767	0.008802926531490	47.7	50-67-9
neg_17222	Pe(Pgf2Alpha/14:1(9Z))	metab_48449	B(ii)	0	72.5	HMDB0260705	M-H2O-H	C39H70NO11P	3.3673833333333	-	neg	740.4543254	0.018291656717305	51.5	-
neg_17231	Gly-Phe-Ser	metab_48458	B(i)	90.6	0	-	M-H	C14H19N3O5	3.3673833333333	-	neg	308.12544512739	0.058705501247216	55.7	-
neg_17263	Val-Tyr	metab_48490	B(i)	86.9	0	HMDB0029139	M-H	C14H20N2O4	3.3567666666667	-	neg	279.13511213783	0.010914035016824	56.7	3061-91-4
neg_17270	(2S)-2-Amino-6-[(3-Formylpiperidin-1-Yl)Amino]Hexanoic Acid	metab_48497	B(ii)	0	53.4	HMDB0255325	2M-H	C12H23N3O3	3.3514166666667	-	neg	513.34050204161	0.023911064630289	49.1	-
neg_17280	Olanzapine	metab_48507	B(ii)	0	41.6	HMDB0005012	2M+FA-H	C17H20N4S	3.3460666666667	C07322	neg	669.27379902499	0.021730222276526	44.1	132539-06-1
neg_17281	4-Hydroxy-17Beta-Estradiol-2-S-Glutathione	metab_48508	B(ii)	0	67.8	HMDB0060139	M+FA-H	C28H39N3O9S	3.3460666666667	-	neg	638.24340482385	0.009581206034048	49.3	-
neg_17286	Stallimycin	metab_48513	B(ii)	0	68.5	HMDB0251483	M-H2O-H	C22H27N9O4	3.3460666666667	-	neg	462.20302874403	0.017396307749934	50.4	-
neg_17294	H-Ser-Leu-Ile-Gly-Arg-Leu-Oh	metab_48521	B(ii)	0	61.3	HMDB0253029	M+Cl	C29H55N9O8	3.3407166666667	-	neg	692.38540297311	0.039915766800871	51.1	-
neg_17296	(S)-3-Hydroxy-N-Methylcoclaurine	metab_48523	B(ii)	0	56.3	HMDB0006921	2M-H	C18H21NO4	3.3407166666667	C05202	neg	629.2924415	0.068367906384068	47.7	-
neg_17297	Losartan N2-Glucuronide	metab_48524	B(ii)	0	41.3	HMDB0013847	M-H	C28H31ClN6O	3.3407166666667	-	neg	597.19156169235	0.03479658756387	38.7	-
neg_17298	Pi(5:0/7:0)	metab_48525	B(i)	65.5	0	-	M+FA-H	C21H39O13P	3.3407166666667	-	neg	575.20909698874	0.016928551729567	51.7	-
neg_17300	5-Amino-6-Ribitylamino Uracil	metab_48527	B(ii)	0	62.9	HMDB0011106	2M-H	C9H16N4O6	3.3407166666667	C04732	neg	551.2109399	0.027495699789136	48.6	17014-74-3
neg_17304	Hydrocortisone Hemisuccinate	metab_48531	B(ii)	0	41.7	HMDB0253246	M+FA-H	C25H34O8	3.3407166666667	-	neg	507.22102878745	0.004326429735626	46.9	-
neg_17305	Beclomethasone Propionate	metab_48532	B(ii)	0	51.2	HMDB0248926	M-H	C25H33ClO7	3.3407166666667	-	neg	479.17998122976	0.038518680832935	40.9	-

neg_17306	Tyr-Tyr-Leu	metab_48533	B(i)	41.2	0	-	M-H	C24H31N3O6	3.3407166666667	-	neg	456.21012616183	0.019692994093412	44.7	-
neg_17310	Ile-Asp	metab_48537	B(i)	52.1	0	HMDB0028903	M-H	C10H18N2O5	3.3407166666667	-	neg	245.11438540611	0.022829654608852	45.7	-
neg_17314	Toosendanin	metab_48541	B(ii)	0	67.4	HMDB0259107	M+FA-H	C30H38O11	3.3351333333333	-	neg	619.2425101	0.27445942369379	48.9	-
neg_17331	Thr-Thr-Ile	metab_48558	B(i)	60.8	0	-	M-H	C14H27N3O6	3.3298333333333	-	neg	332.18287365486	0.019528975639722	50.7	-
neg_17350	Glu Lys Ser Glu	metab_48577	B(i)	47.3	0	-	M-H	C19H33N5O10	3.3191333333333	-	neg	490.21589373838	0.006950752648643	48.6	-
neg_17353	2-(Beta-D-Mannopyranosyl)-L-Tryptophan	metab_48580	B(ii)	0	46.3	HMDB0258059	M-H	C17H22N2O7	3.3191333333333	-	neg	365.13578837872	0.061392119449907	48	-
neg_17361	Nicotine Glucuronide	metab_48588	B(ii)	0	41.4	HMDB0001272	2M+FA-H	C16H22N2O6	3.3137833333333	-	neg	721.28934193321	0.028348259330118	45.1	152306-59-7
neg_17370	Asn-Ser-Ile	metab_48597	B(i)	87.2	0	-	M-H	C13H24N4O6	3.3137833333333	-	neg	331.16235172753	0.030247628577816	54.8	-
neg_17371	Asp-Ala-Ile	metab_48598	B(i)	57.5	0	-	M-H	C13H23N3O6	3.3137833333333	-	neg	316.15161898132	0.033986889804259	49.6	-
neg_17380	Cortolone-3-Glucuronide	metab_48607	B(ii)	0	40.1	HMDB0010320	M-H	C27H42O11	3.30845	-	neg	541.26271101935	0.051649142837809	44.5	56162-46-0
neg_17386	Triflusal	metab_48613	B(ii)	0	52.2	HMDB0259212	M-H	C10H7F3O4	3.30845	-	neg	247.02250548119	0.017701950126082	49.9	-
neg_17393	Chloramphenicol Palmitate	metab_48620	B(ii)	0	42.4	HMDB0250087	M-H	C27H42Cl2N2O6	3.3030833333333	C11726;_	neg	559.23737341007	0.012820533785358	37.9	530-43-8;_
neg_17397	Biotripyrrin-A	metab_48624	B(ii)	0	50	HMDB0003323	M-H	C25H27N3O6	3.3030833333333	-	neg	464.17960310206	0.014710379914189	46.4	158649-79-7
neg_17413	Oenanthoside A	metab_48640	B(i)	54.7	0	HMDB0035441	M+FA-H	C16H20O8	3.2977333333333	-	neg	385.11309385154	0.012154085646826	49.3	-
neg_17417	Ps(6 Keto-Pgf1Alpha/18:1(11Z))	metab_48644	B(ii)	0	50.6	HMDB0281451	M+Cl	C44H78NO14P	3.2923833333333	-	neg	910.48655503351	0.017902015739216	47.9	-
neg_17430	Vincristine	metab_48657	B(ii)	0	80.1	HMDB0014681	M-H2O-H	C46H56N4O10	3.2870333333333	C07204	neg	805.38562351794	0.010793633179824	52.1	57-22-7
neg_17440	1-Oleoyl-Sn-Glycero-3-Phospho-D-Myo-Inositol(1-)	metab_48667	B(ii)	0	72.1	HMDB0242160	M+Na-2H	C27H50O12P-	3.2817	-	neg	618.27406766466	0.042586175618968	51.1	-
neg_17442	Icariside E5	metab_48669	B(ii)	0	49.5	HMDB0034749	M+Hac-H	C26H34O11	3.2817	-	neg	581.22051909022	0.009896734043507	46.2	126176-79-2
neg_17451	Asn-Ile	metab_48678	B(i)	84.3	0	HMDB0028734	M-H	C10H19N3O4	3.2817	-	neg	244.13037886142	0.008616743978714	56.2	438533-59-6
neg_17456	Chitin	metab_48683	B(ii)	0	63.3	HMDB0003362	M-H	C16H28N2O11.[C8H13NO5]n	3.27635	C00461;_	neg	626.2420868	0.13578346618112	32.4	1398-61-4
neg_17461	Galactosylhydroxylysine	metab_48688	B(ii)	0	58.3	HMDB0000600	M+Na-2H	C12H24N2O8	3.27635	C05547	neg	345.13050301804	0.05616473345285	48.7	32448-36-5
neg_17462	Glutamylphenylalanine	metab_48689	B(i)	68.8	0	HMDB0029156	M-H	C14H18N2O5	3.27635	-	neg	293.11445293984	0.003570015196252	52.9	20556-22-3
neg_17463	Glu-Pro	metab_48690	B(i)	92.2	0	HMDB0028827	M-H2O-H	C10H16N2O5	3.27635	-	neg	225.08818291388	0.006234895396727	57.7	41745-47-5
neg_17466	Cyclo(Aspartylleucylthreonylvalyltyrosylphenylalanylglycyl)	metab_48693	B(ii)	0	90.6	HMDB0303713	M-H2O-H	C39H53N7O11	3.271	-	neg	776.37017524555	0.02563764888493	53.5	-
neg_17467	Murnac-Tripeptide	metab_48694	B(ii)	0	81.9	HMDB0254953	M+Hac-H	C26H44N6O14	3.271	-	neg	723.3055392	0.019207760190838	55.3	-
neg_17476	Ala-Ala-Leu	metab_48703	B(i)	56.5	0	-	M-H	C12H23N3O4	3.271	-	neg	272.1617378	0.051403160067346	49.2	-
neg_17492	Glu-Thr-Leu	metab_48719	B(i)	64.9	0	-	M-H	C15H27N3O7	3.26565	-	neg	360.17774543975	0.018091260390606	51.2	-
neg_17514	Cis-3-Hexenyl B-Primeveroside	metab_48741	B(ii)	0	69	HMDB0031690	M+FA-H	C17H30O10	3.2549833333333	-	neg	439.18137924763	0.018847333296506	52.4	132278-37-6
neg_17515	Alpha-Methyltyrosine Methyl Ester	metab_48742	B(ii)	0	51	HMDB0248234	2M-H	C11H15NO3	3.2549833333333	-	neg	417.1994354	0.015490234892423	47.3	-
neg_17534	Lexaptepid Pegol	metab_48761	B(ii)	0	66.1	HMDB0251765	2M-H	C18H37N2O10	3.2390166666667	-	neg	943.43828801509	0.041900764864624	48.3	-
neg_17541	Ps(4:0/18:5)	metab_48768	B(i)	46.8	0	-	M-H	C28H44NO10P	3.2390166666667	-	neg	584.26809898248	0.020654617621181	33.8	-
neg_17577	Chlorophyllide A	metab_48804	B(ii)	0	54	HMDB0304288	M+FA-H	C35H34N4O5-2	3.2284166666667	C02139;_	neg	635.24717959832	0.011003718992292	47.9	14897-06-4;724691-52-5;_
neg_17582	Cholylglutamic Acid	metab_48809	B(ii)	0	64.8	HMDB0242384	M+Na-2H	C29H47NO8	3.2284166666667	-	neg	558.30079853305	0.05881816238291	48.2	-
neg_17588	Gln-Phe-Gln	metab_48815	B(i)	71	0	-	M-H	C19H27N5O6	3.2284166666667	-	neg	420.18885758425	0.010641802595509	53.1	-
neg_17590	Phe-Gly-Ser	metab_48817	B(i)	77.1	0	-	M-H	C14H19N3O5	3.2284166666667	-	neg	308.12531545325	0.02519829782642	53.3	-
neg_17593	Metabolite A	metab_48820	B(ii)	0	76.1	HMDB0254494	M+Na-2H	C41H62O15	3.2231166666667	-	neg	815.38597649941	0.023424350974936	52.9	-
neg_17598	Glucosylgalactosyl Hydroxylysine	metab_48825	B(ii)	0	74	HMDB0000585	M+Hac-H	C18H34N2O13	3.2231166666667	-	neg	545.22154274397	0.018907964460745	52.5	32448-35-4
neg_17600	Val Asp Ile Arg	metab_48827	B(i)	75.4	0	-	M-H	C21H39N7O7	3.2231166666667	-	neg	500.28413637025	0.11971128167295	50.5	-
neg_17601	Gln-Gln-Tyr	metab_48828	B(i)	54.9	0	-	M-H	C19H27N5O7	3.2231166666667	-	neg	436.18372896164	0.02861254546544	49.4	-
neg_17602	Clinafloxacin	metab_48829	B(ii)	0	63.3	HMDB0250323	M-H	C17H17ClFN3O3	3.2231166666667	-	neg	364.08760915506	0.039890720639859	45.5	-
neg_17603	2-Methylguanosine	metab_48830	B(i)	77	0	HMDB0005862	M-H	C11H15N5O5	3.2231166666667	-	neg	296.10018628824	0.005617506016676	54.1	2140-77-4
neg_17605	Pgp(Pgd2/I-14:0)	metab_48832	B(ii)	0	60.2	HMDB0275020	M+Na-2H	C40H72O16P2	3.2178166666667	-	neg	891.39693942133	0.005718794449239	49.1	-

neg_17607	Lyciumoside Iv	metab_48834	B(ii)	0	58.4	HMDB0033499	M+Na-2H	C38H64O16	3.2178166666667	-	neg	797.39161131163	0.016361946382924	47.8	212773-31-4
neg_17613	N-((Tetrahydro-5-Oxo-2-Furanyl)Carbonyl)-L-Histidyl-L-	metab_48840	B(ii)	0	46.8	HMDB0252610	M+Hac-H	C16H21N5O5	3.2178166666667	-	neg	422.16828879017	0.024751576238993	47.7	-
neg_17616	Glu-Ser-Ile	metab_48843	B(i)	46.8	0	-	M-H	C14H25N3O7	3.2178166666667	-	neg	346.16209161556	0.007722242388852	48.3	-
neg_17618	Homogentisic Acid	metab_48845	B(i)	97.5	0	HMDB0000130;PW_C000084	M-H	C8H8O4	3.2178166666667	C00544	neg	167.03508413454	0.024163335932324	58.6	451-13-8
neg_17626	Thr Leu Asp Glu	metab_48853	B(i)	75.6	0	-	M-H	C19H32N4O10	3.2125166666667	-	neg	475.20495037092	0.03193179052338	53.4	-
neg_17640	Epanolol	metab_48867	B(ii)	0	48.6	HMDB0251835	2M+FA-H	C20H23N3O4	3.2018166666667	C11773	neg	783.33033768964	0.04558641216017	46.8	86880-51-5;_
neg_17647	Gly-Pro-Gly-Arg-Ala-Phe	metab_48874	B(ii)	0	42.4	HMDB0252833	M-H	C27H41N9O7	3.2018166666667	-	neg	602.30867259781	0.027135846740856	46.7	-
neg_17664	Gypsogenin 3-O-B-D-Glucuronide	metab_48891	B(ii)	0	55.3	HMDB0034520	M+FA-H	C36H54O10	3.1911333333333	-	neg	691.37512203368	0.039458734069955	47	-
neg_17669	Trans-Piceid	metab_48896	B(ii)	0	45.3	HMDB0030564	M+FA-H	C20H22O8	3.1911333333333	C10275	neg	435.12594288843	0.027966214168032	46.7	27208-80-6
neg_17670	Ser-Gln-Tyr	metab_48897	B(i)	81.8	0	-	M-H	C17H24N4O7	3.1911333333333	-	neg	395.15725795399	0.005482984866872	54.5	-
neg_17673	Ps(6 Keto-Pgf1Alpha/20:4(8Z,11Z,14Z,17Z))	metab_48900	B(ii)	0	59.4	HMDB0282698	M+Cl	C46H76NO14P	3.1858	-	neg	932.47098444293	0.015540965350136	50.1	-
neg_17683	Lys-Trp-Leu	metab_48910	B(i)	37.9	0	-	M-H	C23H35N5O4	3.1858	-	neg	444.25798814323	0.004006618826821	44	-
neg_17684	3-Methyluridine	metab_48911	B(i)	62.5	0	HMDB0004813	M+FA-H	C10H14N2O6	3.1858	-	neg	303.08350088063	0.02632011996149	51.3	2140-69-4
neg_17701	N-Allyl-N'-{4-[3,5-	metab_48928	B(i)	66.6	0	-	M+Hac-H	C18H14F6N2O	3.1752	-	neg	479.08978592531	0.009378554064581	49	-
neg_17702	Castanospermine	metab_48929	B(ii)	0	49.4	HMDB0249700	2M-H	C8H15NO4	3.1752	C02256;_	neg	377.19313381225	0.014558184301079	48.8	79831-76-8;_
neg_17703	Val-Ile-Ser	metab_48930	B(i)	59	0	-	M-H	C14H27N3O5	3.1752	-	neg	316.18796066958	0.024544832917255	50	-
neg_17709	Cep-1347	metab_48936	B(ii)	0	54.2	HMDB0249804	M+Hac-H	C33H33N3O5S	3.1642833333333	-	neg	674.20469630232	0.093676577582648	41.5	-
neg_17714	Apricoxib	metab_48941	B(ii)	0	64.3	HMDB0249626	2M+FA-H	C19H20N2O3S	3.1589333333333	-	neg	757.23394837351	0.15172276070274	50	-
neg_17718	Gladiatoside C1	metab_48945	B(ii)	0	40.1	HMDB0036255	M+Hac-H	C29H26O12	3.1589333333333	-	neg	625.15283892693	0.045262525269562	44.4	-
neg_17720	1,2,3,4-Tetrahydroisoquinoline-3-Carboxylic Acid	metab_48947	B(ii)	0	53.9	HMDB0244117	M+Hac-H	C10H11NO2	3.1589333333333	-	neg	236.09291472829	0.012942090187141	50.4	-
neg_17723	Asp-Arg-Ile	metab_48950	B(i)	94.2	0	-	M-H	C16H30N6O6	3.15365	-	neg	401.21565915368	0.027189588031483	57.1	-
neg_17725	Val-Pro	metab_48952	B(i)	76.8	0	HMDB0029135	M-H	C10H18N2O3	3.15365	-	neg	213.12460589838	0.031625305320274	54	20488-27-1
neg_17733	Fluocortolone Caproate	metab_48960	B(ii)	0	73.3	HMDB0252352	M+FA-H	C28H39FO5	3.1377	-	neg	519.27839926662	0.0294200418028	50.7	-
neg_17734	Tyr-Gln-Lys	metab_48961	B(i)	74.3	0	-	M+FA-H	C20H31N5O6	3.1377	-	neg	482.22359332995	0.096461694737401	51.8	-
neg_17736	Delimotecan	metab_48963	B(ii)	0	73.9	HMDB0250970	M+Hac-H	C31H36N6O8	3.1323666666667	-	neg	679.26906793522	0.011686253232672	51.6	-
neg_17742	Limonoate A-Ring-Lactone	metab_48969	B(ii)	0	60	HMDB0302537	M+FA-H	C26H32O9	3.1270333333333	C16718	neg	533.20371656893	0.026379647855897	49.3	-;_
neg_17744	Arg-Phe-Asp	metab_48971	B(i)	44.4	0	-	M-H	C19H28N6O6	3.1270333333333	-	neg	435.20006533767	0.066467602799422	46.4	-
neg_17756	Leu-Ile-Lys	metab_48983	B(i)	40.8	0	-	M-H	C18H36N4O4	3.1163333333333	-	neg	371.26651345445	0.030290779077935	46.1	-
neg_17763	27-O-Demethylrifabutin	metab_48990	B(ii)	0	44.4	HMDB0061043	M-H	C45H60N4O11	3.1056333333333	-	neg	831.42354183471	0.022476053920209	45.2	-
neg_17765	Leu Glu Asp Arg	metab_48992	B(i)	52.5	0	-	M-H	C21H37N7O9	3.1056333333333	-	neg	530.25818807586	0.014716130299863	48.5	-
neg_17767	Tubacin	metab_48994	B(ii)	0	53.9	HMDB0259315	M-H	C41H43N3O7S	3.1003	-	neg	720.27134661028	0.047651157998753	44.9	-
neg_17771	Leu-Pro-His	metab_48998	B(i)	79.3	0	-	M-H	C17H27N5O4	3.1003	-	neg	364.19913041679	0.03026152859899	54.6	-
neg_17774	4-Hydroxy-2-Nonenal-Glutathione Conjugate	metab_49001	B(ii)	0	52.9	HMDB0304177	M+Hac-H	C19H32N3O8S	3.08965	-	neg	521.2005557	0.026536047546735	44.8	-
neg_17782	Etimicin	metab_49009	B(ii)	0	58.9	HMDB0252101	M+Cl	C21H43N5O7	3.0789666666667	-	neg	512.28392789523	0.025885184396135	50.3	-
neg_17784	Val-Val-Ala	metab_49011	B(i)	79.6	0	-	M-H	C13H25N3O4	3.0736333333333	-	neg	286.17743686149	0.004922082940499	54.9	-
neg_17785	N-Acetyl-9-O-Lactoylneuraminic Acid	metab_49012	B(ii)	0	59.6	HMDB0000768	M+FA-H	C14H23NO11	3.0683333333333	-	neg	426.1266564	0.033097236222347	50.2	92935-30-3
neg_17786	Lys-Tyr	metab_49013	B(i)	55.1	0	HMDB0028963	M+FA-H	C15H23N3O4	3.0683333333333	-	neg	354.16484783307	0.015647721368311	48	35978-98-4
neg_17787	Gdc-0834 Racemate	metab_49014	B(ii)	0	48.3	HMDB0252657	M+FA-H	C33H36N6O3S	3.0629833333333	-	neg	641.2539164	0.030545279227011	45.8	-
neg_17791	Thr-Thr-Leu	metab_49018	B(i)	93.7	0	-	M-H	C14H27N3O6	3.04695	-	neg	332.18295164987	0.044382088554293	56.7	-
neg_17795	Leu-Ile-His	metab_49022	B(i)	85.9	0	-	M-H	C18H31N5O4	3.0309333333333	-	neg	380.23041059391	0.017206753393325	56.3	-
neg_17805	Ser-Gly-Tyr	metab_49032	B(i)	55.8	0	-	M-H	C14H19N3O6	2.9986	-	neg	324.12000506764	0.025930140862458	48.9	-
neg_17807	Phe-Glu-Tyr	metab_49034	B(i)	62	0	-	M-H	C23H27N3O7	2.99325	-	neg	456.17384877479	0.0154742142359	48.6	-
neg_17808	5-Methyluridine	metab_49035	B(i)	80.3	0	HMDB0000884	M-H	C10H14N2O6	2.99325	-	neg	257.07801916711	0.018811117880952	55.4	1463-10-1
neg_17809	Estradiol-17Beta-Glucuronide	metab_49036	B(i)	75.5	0	-;HMDB0251969	M+FA-H	C24H32O8	2.9826	C11237;_	neg	493.20354088645	0.019659594876061	51.4	1806-98-0;_
neg_17810	Indolo[2,1-B]Quinazoline-6,12-Dione	metab_49037	B(ii)	0	60.2	HMDB0253473	M+FA-H	C15H8N2O2	2.9826	C10742	neg	293.05483029273	0.2682992657772	47.6	-
neg_17811	N-Methylserotonin	metab_49038	B(ii)	0	50.5	HMDB0004369	2M+FA-H	C11H14N2O	2.9772666666667	C06212	neg	425.21565443747	0.004412063093949	46.6	1134-01-6

neg_17813	Val-Ala-Asp	metab_49040	B(i)	64.4	0	-	M-H	C12H21N3O6	2.9613	-	neg	302.13592541714	0.018507567064693	51.5	-
neg_17814	Xanthosine	metab_49041	B(i)	76.5	0	HMDB0000299;PW_C000203	M-H	C10H12N4O6	2.9560166666667	C01762	neg	283.06838322798	0.038290796383006	53.4	146-80-5
neg_17822	Gamma-Glutamyltyrosine	metab_49049	B(i)	83.3	0	HMDB0011741	M-H	C14H18N2O6	2.8760166666667	-	neg	309.1099311	0.016164844901159	55	7432-23-7
neg_17833	Glutamyltyrosine	metab_49060	B(ii)	0	40.2	HMDB0028831	M+Na-2H	C14H18N2O6	2.8494166666667	-	neg	331.09157662949	0.025370164649992	46.1	3422-39-7
neg_17837	Relamorelin	metab_49064	B(ii)	0	64.1	HMDB0257141	M-H	C43H50N8O5S	2.8385166666667	-	neg	789.3568624	0.004914426321220	47.7	-
neg_17838	2,5-Dibenzyl-3-Hydroxy-6-Methoxypyrazine	metab_49065	B(ii)	0	56.1	HMDB0041543	M+FA-H	C19H18N2O2	2.8385166666667	-	neg	351.13269407354	0.017188668424291	48.6	132213-65-1
neg_17854	N-Formyl-Met-Leu-Phe	metab_49081	B(ii)	0	43.2	HMDB0255144	M+Cl	C21H31N3O5S	2.7745833333333	-	neg	472.16809969389	0.060105187759806	44.8	-
neg_17856	Ser-Leu	metab_49083	B(i)	95.5	0	HMDB0258242	M-H	C9H18N2O4	2.7745833333333	-	neg	217.11946684809	0.00500073289704	58.5	-
neg_17858	Cinnzeylanine	metab_49085	B(ii)	0	52.2	HMDB0036011	M-H	C22H34O8	2.7639833333333	-	neg	425.21500522588	0.078673580717116	47.6	62203-47-8
neg_17882	Oleoside Dimethyl Ester	metab_49109	B(i)	68.8	0	HMDB0031350	M+FA-H	C18H26O11	2.7214	-	neg	463.14282343303	0.18120796725794	49.1	30164-95-5
neg_17884	N-Acetyllactosamine	metab_49111	B(ii)	0	59.5	HMDB0001542	M+FA-H	C14H25NO11	2.7107166666667	C00611	neg	428.14247401518	0.03790653290925	49.3	32181-59-2
neg_17885	Humilixanthin	metab_49112	B(ii)	0	57.8	HMDB0039123	M+Na-2H	C14H18N2O7	2.6946333333333	C08548	neg	347.08620403055	0.02341797956157	49.9	111534-70-4
neg_17886	N-Hydroxy-L-Tryptophan	metab_49113	B(ii)	0	52.3	HMDB0255152	M+Hac-H	C11H12N2O3	2.6946333333333	_;C19716	neg	279.09872865501	0.005397252173991	50.1	-;_
neg_17887	Phe-Thr-Tyr	metab_49114	B(i)	83.3	0	-	M-H	C22H27N3O6	2.6840333333333	-	neg	428.17876224204	0.060141498434285	50	-
neg_17889	2-Nonenoylglycine	metab_49116	B(ii)	0	44.2	HMDB0094807	2M+FA-H	C11H19NO3	2.6572333333333	-	neg	471.26864573357	0.22593298245023	45.8	-
neg_17895	Theogallin	metab_49122	B(ii)	0	40.5	HMDB0039287	M-H	C14H16O10	2.61985	C10834;_	neg	343.06709571174	0.063276783337909	46.8	17365-11-6;53584-43-3
neg_17904	2,3-Dinor-Txb2	metab_49131	B(ii)	0	54.7	HMDB0002904	M+Na-2H	C18H30O6	2.5717333333333	-	neg	363.17731778323	0.029296477734171	48.2	63250-09-9;
neg_17905	Phe-Thr	metab_49132	B(i)	96.8	0	HMDB0029005	M-H	C13H18N2O4	2.5185166666667	-	neg	265.11951169182	0.009602525873711	58.5	51352-44-4
neg_17906	Arg-Ala-Ile	metab_49133	B(i)	89.3	0	-	M-H	C15H30N6O4	2.5078833333333	-	neg	357.22571200116	0.16540921235149	56.4	-
neg_17912	3-Hydroxycotinine Glucuronide	metab_49139	B(ii)	0	44.8	HMDB0245897	M-H2O-H	C16H20N2O8	2.4757666666667	-	neg	349.10211134474	0.15594067139604	45.5	-
neg_17914	2-Hydroxy-Desipramine Glucuronide	metab_49141	B(ii)	0	66.9	HMDB0060716	M+Hac-H	C24H30N2O7	2.4598	-	neg	517.21510430739	0.050412644730415	50.2	-
neg_17916	Asp Phe Glu Lys	metab_49143	B(i)	49.1	0	-	M-H	C24H35N5O9	2.4491	-	neg	536.2363432	0.005376765030471	48.8	-
neg_17924	Cymorcin Diglucoside	metab_49151	B(ii)	0	72.8	HMDB0039386	M+Cl	C22H34O12	2.3905333333333	-	neg	525.17814499563	0.022610954860715	50.9	-
neg_17926	Ala-Gly-Tyr	metab_49153	B(i)	70.8	0	-	M-H	C14H19N3O5	2.3693	-	neg	308.12536305308	0.005080075581562	53.1	-
neg_17928	Cyclacillin	metab_49155	B(ii)	0	67.3	HMDB0015135	M+Cl	C15H23N3O4S	2.3587	C12766	neg	376.11279715018	0.02396040075958	49.9	3485-14-1
neg_17930	Histidinohydroxylysinonorleucine	metab_49157	B(ii)	0	65.1	HMDB0253184	M+FA-H	C18H32N6O7	2.33195	-	neg	489.23166992782	0.18074756653147	51.4	-
neg_17936	Ser-Glu-Met	metab_49163	B(i)	86.1	0	-	M-H	C13H23N3O7S	2.2840666666667	-	neg	364.11863295756	0.23013179419357	54.7	-
neg_17943	Anhydrocinnzeylanine	metab_49170	B(ii)	0	43.7	HMDB0036864	M-H	C22H32O7	2.2145333333333	-	neg	407.20511984618	0.094323082397274	44.6	68799-62-2
neg_17944	Roxadustat	metab_49171	B(ii)	0	50	HMDB0252245	M+Cl	C19H16N2O5	2.2145333333333	-	neg	387.07880813297	0.039695859181524	45.8	-
neg_17950	2-Amino-3-Methyl-1-Pyrrolidin-1-Yl-Butan-1-One	metab_49177	B(ii)	0	65.4	HMDB0246938	M+FA-H	C9H18N2O	2.1932	-	neg	215.14018741064	0.001650611834237	52.8	-
neg_17957	D-Digitoxose	metab_49184	B(ii)	0	60.8	HMDB0251279	M-H	C6H12O4	2.1665	_;C21045	neg	147.06640181697	0.015105851642622	51.7	-;_
neg_17960	Dezinamide	metab_49187	B(ii)	0	51.4	HMDB0251101	M+FA-H	C11H11F3N2O	2.1611833333333	-	neg	305.07575017248	0.057268461634492	49.2	-
neg_17962	D-Pinitol	metab_49189	B(ii)	0	55.7	HMDB0034219	M+Na-2H	C7H14O6	2.15585	C03844	neg	215.05391171619	0.017572238532389	50.4	10284-63-6
neg_17965	(4R,5S,7R,11X)-11,12-Dihydroxy-1(10)-Spirovetiven-2-One 12-Glucoside	metab_49192	B(ii)	0	61.4	HMDB0030895	M+Hac-H	C21H34O8	2.1451666666667	-	neg	473.23618342973	0.031162409763641	48.8	62574-29-2
neg_17966	2,5-Anhydro-D-Mannose	metab_49193	B(ii)	0	41.6	HMDB0245490	M+Na-2H	C6H10O5	2.1398333333333	-	neg	183.02746904331	0.011500925575386	47.4	-
neg_17980	Tyr-Val-Asn	metab_49207	B(i)	79.3	0	-	M-H	C18H26N4O6	2.0861833333333	-	neg	393.17773511515	0.085358680201879	54.8	-
neg_17984	18-Carboxy-Dinor-Lte4	metab_49211	B(ii)	0	55.1	HMDB0012607	M+FA-H	C21H31NO7S	2.07555	-	neg	486.18223961485	0.1162539340827	44.8	_;
neg_17985	Lisinopril	metab_49212	B(ii)	0	44.4	HMDB0001938	M-H2O-H	C21H31N3O5	2.07555	D00362	neg	386.20462849013	0.031521204514272	44.5	83915-83-7;76547-98-3
neg_17986	Gallic Acid	metab_49213	B(i)	93.6	0	HMDB0005807	M-H	C7H6O5	2.07555	C01424	neg	169.01443563863	0.020286952065462	57.5	149-91-7
neg_17997	Acid Yellow 11	metab_49224	B(i)	35.4	0	-	M+FA-H	C16H14N4O4S	2.0543	-	neg	403.07356447201	0.01203199551648	43.6	-
neg_18007	Lys-Phe-Asp	metab_49234	B(i)	54.9	0	-	M-H	C19H28N4O6	2.03835	-	neg	407.1941867	0.12789138749976	48	-
neg_18010	2'-Deoxyguanosine 5'-Phosphate	metab_49237	B(i)	71.4	0	HMDB0001044	2M-H	C10H14N5O7P	2.0330666666667	C00362	neg	693.11922295396	0.069928768869483	53.3	902-04-5
neg_18016	Adenosine 2'-Phosphate	metab_49243	B(i)	90.3	0	HMDB0011617	M-H	C10H14N5O7P	2.0330666666667	C00946	neg	346.05585816572	0.027498210905503	57.5	130-49-4
neg_18018	D-Ribose-1-Phosphate	metab_49245	B(i)	62.1	0	HMDB0001489	M-H2O-H	C5H11O8P	2.0330666666667	C00620	neg	211.00142221963	0.051686219822532	51.3	14075-00-4

neg_18036	Leu-Gly	metab_49263	B(i)	85.3	0	HMDB0000759	M-H	C8H16N2O3	2.0224166666667	C02155	neg	187.10889213026	0.008072723420077	56.5	869-19-2
neg_18042	Phe-Asp-Arg	metab_49269	B(i)	97.1	0	-	M-H	C19H28N6O6	2.0117833333333	-	neg	435.19985913699	0.01426770174965	58.8	-
neg_18052	Didemethylcitalopram	metab_49279	B(ii)	0	65.1	HMDB0060472	2M+FA-H	C18H17FN2O	2.0009	C16609	neg	637.25881979823	0.023222544917386	47.6	62498-69-5;_
neg_18053	3A-Methyl-2,3,4,5,5A,10,10A,10B-Octahydro-1H-Cyclopenta[A]Fluorene-2,3,7-Triol	metab_49280	B(ii)	0	51.2	HMDB0257775	2M-H	C17H22O3	2.0009	-	neg	547.30985425455	0.020090666381134	47.8	-
neg_18057	Ile-Gly-Asp	metab_49284	B(i)	38.8	0	-	M-H	C12H21N3O6	2.0009	-	neg	302.13597544602	0.012157559478261	46.2	-
neg_18062	Piroxantrone	metab_49289	B(ii)	0	58.4	HMDB0256611	M+Hac-H	C21H25N5O4	1.9956	-	neg	470.20116937656	0.005754199586164	47.3	-
neg_18063	Arg-Glu-Leu	metab_49290	B(i)	70.5	0	-	M-H	C17H32N6O6	1.9956	-	neg	415.23133720685	0.031920436184295	52.3	-
neg_18092	Glucose 3-Hydroxybutyrate	metab_49319	B(ii)	0	50.6	HMDB0252771	2M-H	C10H18O8	1.9743833333333	-	neg	531.19297894057	0.11993609388192	43.9	-
neg_18100	Zaragozic Acid A	metab_49327	B(ii)	0	50.1	HMDB0259988	M-H	C35H46O14	1.9690333333333	-	neg	689.27477697653	0.029584923337385	44.3	_;
neg_18118	3-[(2-Methyl-3-Furanyl)Thio]-4-Heptanone	metab_49345	B(ii)	0	51	HMDB0037158	M+Na-2H	C12H18O2S	1.9690333333333	-	neg	247.07588062737	0.042552452630319	46.6	61295-41-8
neg_18119	3-Butene-1,2,3-Tricarboxylic Acid	metab_49346	B(i)	81.4	0	-	M-H	C7H8O6	1.9690333333333	-	neg	187.02491546428	0.005956234960392	55.8	-
neg_18130	Isoleucyl-Prolyl-Arginine-4-Nitroanilide	metab_49357	B(ii)	0	41	HMDB0250210	M+FA-H	C23H36N8O5	1.9637166666667	-	neg	549.27928064662	0.027638996628814	47.1	-
neg_18141	Guanosine 5'-Monophosphate	metab_49368	B(i)	96	0	HMDB0001397	M-H	C10H14N5O8P	1.9637166666667	C00144	neg	362.05072848412	0.005049838143278	58.6	85-32-5
neg_18147	4-(2-Amino-3-Hydroxyphenyl)-2,4-Dioxobutanoic Acid	metab_49374	B(ii)	0	62.7	HMDB0004083	M-H	C10H9NO5	1.9637166666667	C05645	neg	222.0408814	0.007443267241062	51.9	857760-67-9;_
neg_18162	2-Methylcitric Acid	metab_49389	B(i)	73.4	0	HMDB0000379	M-H	C7H10O7	1.9583833333333	-	neg	205.03545915635	0.091021922866621	53.8	6061-96-7
neg_18164	Itaconic Acid	metab_49391	B(i)	89.9	0	HMDB0002092;LMFA01170063	M-H	C5H6O4	1.9583833333333	C00490	neg	129.01946280788	0.035493633029992	56.9	97-65-4;
neg_18175	N-(1-Deoxy-1-Fructosyl)Leucine	metab_49402	B(ii)	0	50.6	HMDB0037840	M-H	C12H23NO7	1.9530333333333	-	neg	292.14031098831	0.031244294401914	49.2	34393-18-5
neg_18176	Ile-Gln	metab_49403	B(i)	86.8	0	HMDB0028905	M-H	C11H21N3O4	1.9530333333333	-	neg	258.14605439945	0.024239957098863	55.5	-
neg_18179	L-Leucyl-L-Alanine	metab_49406	B(i)	88.3	0	HMDB0028922	M-H	C9H18N2O3	1.9530333333333	-	neg	201.12456925124	0.001203959331667	57.1	7298-84-2
neg_18186	Arg-Val-Leu	metab_49413	B(i)	64	0	-	M-H	C17H34N6O4	1.9477333333333	-	neg	385.25699506457	0.059500489638823	51.7	-
neg_18189	Tyr-Glu	metab_49416	B(i)	85.9	0	HMDB0029104	M-H	C14H18N2O6	1.9477333333333	-	neg	309.10937280476	0.017712618445443	55.9	-
neg_18192	Gly-Arg-Gly-Glu-Ser-Pro	metab_49419	B(ii)	0	59.9	HMDB0252941	M-H	C23H39N9O10	1.9424	-	neg	600.27483588383	0.10059726949523	50.9	-
neg_18195	D,L-Buthionine	metab_49422	B(ii)	0	47.3	HMDB0250809	2M+FA-H	C8H17NO2S	1.9424	-	neg	427.19484350807	0.007705299968778	47.5	-
neg_18196	Leu-Val-His	metab_49423	B(i)	76.5	0	-	M-H	C17H29N5O4	1.9424	-	neg	366.2146608	0.094589307390625	53.5	-
neg_18197	Amprenavir	metab_49424	B(ii)	0	47.1	HMDB0014839	M+Hac-H	C25H35N3O6S	1.9371	C08086	neg	564.24268604171	0.037540441175118	44.8	161814-49-9
neg_18201	Asn-Met-Ser	metab_49428	B(i)	64.8	0	-	M-H	C12H22N4O6S	1.9371	-	neg	349.11876838392	0.009786458111505	51.1	-
neg_18202	Gly-Met-Ser	metab_49429	B(i)	57.2	0	-	M-H	C10H19N3O5S	1.9371	-	neg	292.09748992348	0.069090324867973	48.8	-
neg_18203	Tyrosylalanine	metab_49430	B(i)	85.7	0	HMDB0029098	M-H	C12H16N2O4	1.9371	-	neg	251.10389502277	0.095570849418742	55.8	-
neg_18208	3-Pyridinemethanol, 6-Amino-Alpha-(((1-Methyl-4-Phenylbutyl)Amino)Methyl)-	metab_49435	B(ii)	0	49	HMDB0253841	M+Na-2H	C18H25N3O	1.9317666666667	-	neg	320.17298563839	0.044705213525899	47.1	-
neg_18209	Val-Asn-Ser	metab_49436	B(i)	61.5	0	-	M-H	C12H22N4O6	1.9317666666667	-	neg	317.14677842033	0.020248205773458	50.4	-
neg_18215	Cis-4-Hydroxy-D-Proline	metab_49442	B(ii)	0	46.6	HMDB0060460	M+Hac-H	C5H9NO3	1.9264666666667	C03440	neg	190.07223027436	0.057062268919274	48.3	2584-71-6
neg_18227	D-Xylulose	metab_49454	B(ii)	0	71	HMDB0001644	M+Na-2H	C5H10O5	1.8995333333333	C00310	neg	171.02756975729	0.011275235935625	53.7	551-84-8;20750-28-1
neg_18233	3,4-Dihydroxyphenylglycol	metab_49460	B(i)	55.3	0	HMDB0000318;PW_C000222	M-H2O-H	C8H10O4	1.8782333333333	C05576	neg	151.04027393981	0.20127900367444	48.6	28822-73-3;3343-19-9
neg_18234	4-Methoxybenzaldehyde N-[6-(Tert-Butyl)Thieno[3,2-D]Pyrimidin-4-Yl]Hydrazone	metab_49461	B(i)	35.9	0	-	M+FA-H	C18H20N4OS	1.8569	-	neg	385.13599465032	0.039555214208813	42.6	-
neg_18237	2-(Malonylamino)Benzoic Acid	metab_49464	B(ii)	0	63.3	HMDB0039495	M-H	C10H9NO5	1.85155	C03147	neg	222.04088714313	0.02649760066219	51.9	53947-84-5
neg_18241	Pulcherosine	metab_49468	B(ii)	0	65.9	HMDB0040703	M+Na-2H	C27H29N3O9	1.8299666666667	-	neg	560.17033182724	0.14913984107211	47.2	126723-16-8
neg_18245	Arg-Thr-Ile	metab_49472	B(i)	72.7	0	-	M-H	C16H32N6O5	1.79805	-	neg	387.23647264142	0.061363228584445	52.3	-
neg_18274	Biotin Sulfone	metab_49501	B(ii)	0	52.2	HMDB0004818	M+Cl	C10H16N2O5S	1.6913333333333	C20387;_	neg	311.049809	0.09230076194606	47.1	40720-05-6
neg_18280	Mevalonic Acid	metab_49507	B(i)	54.3	0	HMDB0000227;LMFA01050352;PW_C000152	M-H	C6H12O4	1.6859833333333	C00418	neg	147.06642726524	0.0010733843832041	50.1	17817-88-8;;150-97-0
neg_18286	Glycerol Lactate Pyruvate	metab_49513	B(ii)	0	58.2	HMDB0252841	M-H2O-H	C9H14O7	1.6806333333333	-	neg	215.05396876894	0.008896770806826	48.8	-
neg_18287	Reproterol	metab_49514	B(ii)	0	51.3	HMDB0257155	M+Cl	C18H23N5O5	1.6753333333333	-	neg	424.13637729949	0.095224380015575	47.6	-
neg_18297	Aspartyl-Valine	metab_49524	B(ii)	0	61.6	HMDB0028766	M-H	C9H16N2O5	1.6328333333333	-	neg	231.09874683739	0.022259533321008	51.3	-

neg_18299	N-(1-Deoxy-1-Fructosyl)Alanine	metab_49526	B(ii)	0	46.1	HMDB0038662	M+Hac-H	C9H17NO7	1.6275333333333	-	neg	310.11440109896	0.057183629372643	49	16124-24-6
neg_18303	N2-Succinyl-L-Ornithine	metab_49530	B(ii)	0	45.2	HMDB0001199	M-H2O-H	C9H16N2O5	1.6221666666667	C03415	neg	213.08823180096	0.29199900412015	47.4	-;899816-95-6
neg_18309	Gly-Val	metab_49536	B(i)	91.9	0	HMDB0028854	M-H	C7H14N2O3	1.5956333333333	-	neg	173.09329562133	0.17338856837707	56.9	1963-21-9
neg_18312	4-Aminophenylmannoside	metab_49539	B(ii)	0	40.2	HMDB0246178	M+Na-2H	C12H17NO6	1.59035	-	neg	292.07965236911	0.18807333293245	45.1	-
neg_18314	4-Chloro-3,5-Dinitrobenzotrifluoride	metab_49541	B(ii)	0	46.9	HMDB0246388	M-H	C7H2ClF3N2O	1.59035	-	neg	268.95937190987	0.22380326703448	42.2	-
neg_18329	2-[[2-[[(2S)-2-Amino-3-Methylbutanoyl]Amino]Acetyl]Amino]Pentanedioic Acid	metab_49556	B(i)	54.6	0	-	M-H	C12H21N3O6	1.5530666666667	-	neg	302.13595896296	0.2261937078737	48.8	-
neg_18331	Glucose Pyruvate	metab_49558	B(ii)	0	44.9	HMDB0252786	M+Na-2H	C9H14O9	1.54775	-	neg	287.03861709461	0.082564926270997	47.2	-
neg_18345	Methylmalonic Acid	metab_49572	B(i)	44.9	0	HMDB0000202;LMFA01170118;PW_C000130	M-H2O-H	C4H6O4	1.5265	C02170	neg	99.008847622991	0.009131297443276	48.4	516-05-2;
neg_18351	6-Hydroxy-5-Methoxyindole Glucuronide	metab_49578	B(ii)	0	41	HMDB0010362	M-H2O-H	C15H17NO8	1.5212	-	neg	320.07520884572	0.001804346452477	45.3	77463-72-0
neg_18353	Glucaron	metab_49580	B(ii)	0	45	HMDB0247896	M-H	C10H10O8	1.5212	-	neg	257.02788833871	0.007182811525549	46.4	-
neg_18361	Asn-Met	metab_49588	B(i)	83.1	0	HMDB0028737	M-H	C9H17N3O4S	1.4894	-	neg	262.08683681048	0.11420638913461	53.4	-
neg_18373	Ala-Val-Oh	metab_49600	B(i)	44.6	0	-	M-H	C13H16N2O6	1.4202333333333	-	neg	295.09406390492	0.035161451233434	46.7	-
neg_18378	Ser-Met	metab_49605	B(i)	95.8	0	HMDB0029045	M-H	C8H16N2O4S	1.3988666666667	-	neg	235.07591252367	0.023941179027481	57.8	3227/9/6
neg_18385	Sumiki'S Acid	metab_49612	B(ii)	0	47.3	HMDB0002432	M+FA-H	C6H6O4	1.3776333333333	C20448	neg	187.02492627788	0.021137732640667	48.9	6338-41-6
neg_18386	Cis-Aconitic Acid	metab_49613	B(i)	90.5	0	HMDB0000072;PW_C000051	M-H	C6H6O6	1.3776333333333	C00417	neg	173.00923862698	0.021158603228466	57.3	585-84-2
neg_18387	Cefoxitin	metab_49614	B(ii)	0	47.3	HMDB0015426	M-H2O-H	C16H17N3O7S	1.3723333333333	C06887	neg	408.02914858131	0.26774243256264	43.2	35607-66-0
neg_18401	Niazicin	metab_49628	B(ii)	0	54.6	HMDB0038952	M+Hac-H	C17H23NO7S	1.3563333333333	-	neg	444.13726966522	0.12585950517433	46.5	159768-74-8
neg_18403	Ala-His-Tyr	metab_49630	B(i)	46.4	0	-	M-H	C18H23N5O5	1.3563333333333	-	neg	388.16145252531	0.12616360356019	47.5	-
neg_18409	Adenosine Dialdehyde	metab_49636	B(ii)	0	40.8	HMDB0248007	M+Hac-H	C10H11N5O4	1.3563333333333	-	neg	324.09500673022	0.17251929814088	45.6	-
neg_18413	Val-Glu	metab_49640	B(i)	80	0	HMDB0029126	M-H	C10H18N2O5	1.3563333333333	-	neg	245.11440675511	0.002888277060367	55	3062/7/5
neg_18415	Xanthine	metab_49642	B(i)	97.6	0	HMDB0000292;PW_C000198	M-H	C5H4N4O2	1.3563333333333	C00385	neg	151.02626748881	0.012176029440276	58	69-89-6
neg_18423	Fumaric Acid	metab_49650	B(i)	98.6	0	HMDB0000134;PW_C000088	M-H	C4H4O4	1.3510166666667	C00122	neg	115.00375787324	0.003708034449878	59.4	110-17-8
neg_18433	Asn-Val	metab_49660	B(i)	82	0	HMDB0028744	M-H	C9H17N3O4	1.3404166666667	-	neg	230.11475876975	0.026876684805982	54.4	-
neg_18435	Cefminox	metab_49662	B(ii)	0	64	HMDB0249766	M-H2O-H	C16H21N7O7S	1.3350833333333	-	neg	500.04349882689	0.04916890240408	46.1	-
neg_18439	N-Acetyl-Dl-Glutamic Acid	metab_49666	B(i)	63.6	0	-	M-H	C7H11NO5	1.3297333333333	-	neg	188.05658751561	0.068873492345029	50.8	-
neg_18448	Ser-Val	metab_49675	B(i)	96.2	0	HMDB0029052	M-H	C8H16N2O4	1.3085333333333	-	neg	203.103825	0.004721057772499	58.5	51782-06-0
neg_18455	Spongouridin	metab_49682	B(i)	94.4	0	-	M-H	C9H12N2O6	1.2872666666667	-	neg	243.06231219481	0.031638546552869	58.4	-
neg_18458	Alpha-Hydroxyetizolam	metab_49685	B(ii)	0	42.8	HMDB0248221	M+Na-2H	C17H15ClN4O	1.2819666666667	-	neg	379.03731502747	0.02666131246813	39.3	-
neg_18459	Glu-Val-Asn	metab_49686	B(i)	58.6	0	-	M-H	C14H24N4O7	1.2819666666667	-	neg	359.15699497659	0.2292121192844	48.8	-
neg_18464	Leu-Ser-His	metab_49691	B(i)	42.8	0	-	M-H2O-H	C15H25N5O5	1.2766666666667	-	neg	336.1678489	0.092703167997347	45.7	-
neg_18474	Sec-O-Glucosylhamaudol	metab_49701	B(ii)	0	48.1	HMDB0258199	M-H2O-H	C21H26O10	1.2659666666667	C17484;_	neg	419.13076304167	0.049279260049703	45.8	-;_
neg_18478	Pglu-His-Pro	metab_49705	B(ii)	0	54	HMDB0256375	M+Hac-H	C16H21N5O5	1.2606166666667	-	neg	422.16815298621	0.032420642993004	49.4	-
neg_18480	Gln-Glu-Gln	metab_49707	B(i)	49	0	-	M-H2O-H	C15H25N5O8	1.2606166666667	-	neg	384.15255522002	0.041981716695023	48.2	-
neg_18486	Ser-Tyr	metab_49713	B(i)	75.6	0	HMDB0258243	M-H	C12H16N2O5	1.2497666666667	-	neg	267.09888503789	0.046845011418511	52.9	-
neg_18492	Nebularine	metab_49719	B(ii)	0	44.5	HMDB0029956	M-H	C10H12N4O4	1.23915	C01736	neg	251.07732245056	0.013306503738724	46.5	550-33-4
neg_18494	Griseolic Acid	metab_49721	B(ii)	0	58.5	HMDB0242674	M+Cl	C14H13N5O8	1.23385	-	neg	414.04341642766	0.012562657499024	48.5	-
neg_18497	Arg-Ser-Leu	metab_49724	B(i)	80.5	0	-	M-H	C15H30N6O5	1.2285333333333	-	neg	373.22058126571	0.018052889977291	54.3	-
neg_18501	Cyclic-Hpmpc	metab_49728	B(ii)	0	50.4	HMDB0250198	M+Cl	C8H12N3O5P	1.22325	-	neg	296.01872006647	0.16320183468978	46.6	-
neg_18505	Midazolam	metab_49732	B(ii)	0	44.4	HMDB0014821	M+Na-2H	C18H13ClFN3	1.21795	C07524	neg	346.05592525698	0.010789266237684	40.8	59467-70-8
neg_18519	Ile-Gly-Lys	metab_49746	B(i)	74	0	-	M-H	C14H28N4O4	1.2072333333333	-	neg	315.20391910861	0.10749004268171	51.9	-
neg_18527	3-(((4-Chlorophenyl)Sulfonyl)Methyl)-N-Hydroxybenzimidamide	metab_49754	B(i)	52.6	0	-	2M-H	C14H13ClN2O3S	1.1913333333333	-	neg	647.06455597236	0.013849386963831	43.3	-
neg_18528	Uridine 5'-Monophosphate	metab_49755	B(i)	86.5	0	HMDB0000288;PW_C000194	M-H	C9H13N2O9P	1.1913333333333	C00105	neg	323.02869500711	0.000791659279079	56.9	58-97-9
neg_18529	(E)-Casimiroedine	metab_49756	B(ii)	0	45	HMDB0030274	M+Hac-H	C21H27N3O6	1.1860333333333	C10577	neg	476.20017823873	0.060426741955412	44.8	5853/2/1
neg_18538	E-3179	metab_49765	B(ii)	0	63.4	HMDB0013846	2M+FA-H	C22H21ClN6O	1.1697666666667	-	neg	885.29104172507	0.094168463582477	44.3	-

neg_18555	Citric Acid	metab_49782	B(i)	98.1	0	HMDB0000094;PW_C000063	M-H	C6H8O7	1.15915	C00158	neg	191.01974577033	0.016721730090992	59.4	77-92-9
neg_18559	2-Furoic Acid	metab_49786	B(i)	92.9	0	HMDB0000617	M-H	C5H4O3	1.1538666666667	C01546	neg	111.008841	0.012795091108938	58.2	88-14-2
neg_18562	Leu-Asn	metab_49789	B(i)	90.7	0	HMDB0028924	M-H	C10H19N3O4	1.1432166666667	-	neg	244.13033432734	0.023811592420463	56.7	-
neg_18570	Baohuoside Ii	metab_49797	B(ii)	0	58	HMDB0248861	2M+FA-H	C26H28O10	1.1326333333333	-	neg	1045.335365	0.098796048330889	48.9	-
neg_18576	2-O-Galloylgalactaric Acid	metab_49803	B(ii)	0	51.7	HMDB0036932	M+Na-2H	C13H14O12	1.1326333333333	-	neg	383.02091179889	0.063693649675786	46.5	-
neg_18578	Daucic Acid	metab_49805	B(ii)	0	52.2	HMDB0031665	M-H	C7H8O7	1.1326333333333	-	neg	203.01974576665	0.036163308003243	48.9	34098-52-7
neg_18587	Ser Val Asn Glu	metab_49814	B(i)	46.2	0	-	M-H	C17H29N5O9	1.1219833333333	-	neg	446.18939511421	0.039860632516542	46.1	-
neg_18592	Sialyl Lea Tetra	metab_49819	B(ii)	0	69.5	HMDB0006633	M+Na-2H	C37H62N2O28	1.1166833333333	-	neg	1003.3178923397	0.093295242175672	50.5	127321-43-1
neg_18601	Glu-Gln	metab_49828	B(i)	68.8	0	HMDB0028817	M-H2O-H	C10H17N3O6	1.1166833333333	-	neg	256.09395894922	0.011114187365741	52.5	26848-14-6
neg_18612	3-Sialyl Lewis	metab_49839	B(ii)	0	62.6	HMDB0006579	M+Na-2H	C31H52N2O23	1.10605	-	neg	841.26486007133	0.004756615376048	49.1	92448-22-1
neg_18624	Curcumin Diglucoside	metab_49851	B(ii)	0	42.6	HMDB0304302	M+FA-H	C33H40O16	1.1007	_;C17750	neg	737.23601293908	0.053238726319413	43.6	-;_
neg_18665	Pelargonidin 3-(2Glu Glucosylrutinoside)	metab_49892	B(ii)	0	55.5	HMDB0033689	M+FA-H	C33H41O19+	1.0793666666667	-	neg	786.22109487255	0.075312937681558	47.2	-
neg_18668	(E,E)-1,7-Diphenyl-4,6-Heptadien-3-Ol	metab_49895	B(ii)	0	41.6	HMDB0040893	M+Cl	C19H20O	1.0793666666667	-	neg	299.1225939	0.21800073274304	43.2	152323-34-7
neg_18678	Glutaconic Acid	metab_49905	B(i)	89.3	0	HMDB0000620;LMFA01170109	M-H	C5H6O4	1.0687333333333	C02214	neg	129.01943903237	0.019222802874246	57.1	628-48-8;1724-02-3;
neg_18686	Favipiravir	metab_49913	B(ii)	0	48	HMDB0252176	2M-H	C5H4FN3O2	1.0581166666667	-	neg	313.05180569261	0.069546425224099	47.6	-
neg_18688	3-Deoxy-D-Manno-Octulosonate	metab_49915	B(ii)	0	54.2	HMDB0304125	M+Na-2H	C8H14O8	1.0581166666667	C01187	neg	259.04362886877	0.027076536735417	49.4	10149-14-1;_
neg_18693	Naringin 4'-Glucoside	metab_49920	B(ii)	0	41.2	HMDB0033738	M+Na-2H	C33H42O19	1.0475166666667	-	neg	763.21393894793	0.087775651072207	43	17257-21-5;
neg_18697	Leucyl-Arginine	metab_49924	B(ii)	0	72.6	HMDB0028923	M-H	C12H25N5O3	1.0421833333333	-	neg	286.18858218799	0.048925807426286	52.7	-
neg_18700	Loperamide	metab_49927	B(ii)	0	63.1	HMDB0004999	M-H	C29H33ClN2O	1.0368833333333	C07080	neg	475.21598363652	0.04250813179541	45	53179-11-6
neg_18701	2-(3-Mercaptopropyl)Pentanedioic Acid	metab_49928	B(ii)	0	55.8	HMDB0245244	M-H2O-H	C8H14O4S	1.0368833333333	-	neg	187.04196955204	0.010497204507684	48	-
neg_18706	Asp-Val-Asn	metab_49933	B(i)	79	0	-	M-H	C13H22N4O7	1.0262	-	neg	345.14166422615	0.13723038027401	52.5	-
neg_18707	Fructosylvaline	metab_49934	B(ii)	0	49.5	HMDB0252498	M-H	C11H21NO7	1.0262	-	neg	278.12462298822	0.11161296807031	47.7	-
neg_18721	Cytidine-3'-Monophosphate	metab_49948	B(i)	97.8	0	HMDB0240312	M-H	C9H14N3O8P	1.0208833333333	C05822	neg	322.04465165547	0.046211386867903	58.5	84-52-6
neg_18725	N2-Acetylornithine	metab_49952	B(ii)	0	51.9	HMDB0003357	M-H	C7H14N2O3	1.0208833333333	C00437	neg	173.09323790152	0.035632362439909	48.5	6205/8/9
neg_18730	Didymin	metab_49957	B(ii)	0	77.6	HMDB0029482	M-H	C28H34O14	1.0155833333333	-	neg	593.19347487826	0.031946948315784	51.3	14259-47-3;
neg_18731	Syrups, Hydrolyzed Starch, Hydrogenated	metab_49958	B(ii)	0	69.8	HMDB0258656	M-H	C18H34O16	1.0155833333333	-	neg	505.17777135769	0.053248925100071	53	-
neg_18769	Asp-Glu-Thr	metab_49996	B(i)	77	0	-	M-H	C13H21N3O9	0.99966666666667	-	neg	362.12053508338	0.20788694379058	52.9	-
neg_18770	9-O-Acetylneuraminic Acid	metab_49997	B(ii)	0	43.8	HMDB0255839	M-H2O-H	C11H19NO9	0.99966666666667	-	neg	290.08827420632	0.031230092289008	46.7	-
neg_18779	Methyl (9Z)-6'-Oxo-6,6'-Diapo-6-Carotenoate	metab_50006	B(ii)	0	45.2	HMDB0031978	M-H2O-H	C25H30O3	0.99435	-	neg	359.20487103336	0.14255388550591	42.9	201996-46-5
neg_18780	Glu-Asn	metab_50007	B(i)	54	0	HMDB0028814	M-H2O-H	C9H15N3O6	0.99435	-	neg	242.07833204189	0.048781747689642	48.6	36314-37-1
neg_18781	2-Oxoglutaric Acid	metab_50008	B(i)	99.1	0	HMDB0000208	M-H	C5H6O5	0.99435	C00026	neg	145.01434186796	0.011391974127655	59.2	328-50-7
neg_18796	Maltopentaose	metab_50023	B(i)	70.5	0	HMDB0012254	M-H	C30H52O26	0.98371666666667	-	neg	827.26752434139	0.006516279944159	53.3	34620-76-3
neg_18809	2-Keto-3-Deoxy-D-Mannooctanoic Acid	metab_50036	B(ii)	0	46.5	HMDB0250768	M+Na-2H	C8H14O8	0.97838333333333	-	neg	259.04371360287	0.21246955227149	47.8	-
neg_18840	Tricin 7-[Sinapoyl-(->2)-Glucuronyl-(1->2)-Glucuronide]	metab_50067	B(ii)	0	70.9	HMDB0039907	M+Hac-H	C40H40O23	0.95175	-	neg	947.21643011997	0.10937445445777	49.6	-
neg_18857	Pressinoic Acid	metab_50084	B(ii)	0	82.6	HMDB0256768	M+Na-2H	C33H42N8O10S2	0.93038333333333	-	neg	795.22521439081	0.10917090006854	48.5	-
neg_18875	Glucoliquiritin Apioside	metab_50102	B(ii)	0	89.4	HMDB0041149	M+Na-2H	C32H40O18	0.89838333333333	-	neg	733.20230284211	0.009816244672353	53.8	157226-47-6
neg_18876	Maltotetraose	metab_50103	B(i)	90	0	HMDB0001296	M-H	C24H42O21	0.89838333333333	C02052	neg	665.21480093149	0.016340199310859	57.8	34612-38-9
neg_18880	2''-(6''-P-Coumaroylglucosyl)Quercitrin	metab_50107	B(ii)	0	55.7	HMDB0039332	M+FA-H	C36H36O18	0.8931	-	neg	801.19093668808	0.056969539131811	47.4	113447-39-5
neg_18884	Gdp-Glucose	metab_50111	B(i)	87.8	0	HMDB0003351	M-H	C16H25N5O16P2	0.88246666666667	C00394	neg	604.0701691	0.21646975790918	54.1	-;5750-57-2
neg_18886	(8S)-8-Amino-7-Oxononanoyl-Coa	metab_50113	B(ii)	0	72	HMDB0301611	M+Na-2H	C30H51N8O18P3S	0.87711666666667	-	neg	957.19246832279	0.042571457458492	48.6	-
neg_18889	Cyananin	metab_50116	B(ii)	0	44.9	HMDB0037985	M+Na-2H	C16H10Cl2N2O5	0.87181666666667	-	neg	400.96923950409	0.068367546271832	37.2	_;
neg_18891	Urolithin A 8-Glucuronide	metab_50118	B(ii)	0	43	HMDB0240568	2M+FA-H	C19H16O10	0.86653333333333	-	neg	853.15167662921	0.14718690100849	46.4	-
neg_18899	1,2-Diferuloylgentiobiose	metab_50126	B(ii)	0	67.2	HMDB0301720	M+Hac-H	C32H38O17	0.85588333333333	-	neg	753.23073017621	0.12691246484032	49.1	-

neg_18917	Lucuminamide	metab_50144	B(ii)	0	45.3	HMDB0031697	M+FA-H	C19H27NO11	0.80265	-	neg	490.16029846728	0.076387801296327	45.4	-
neg_18925	Eriocitrin	metab_50152	B(ii)	0	86.3	HMDB0005811	M+Na-2H	C27H32O15	0.79198333333333	C09732	neg	617.15467017315	0.022443790193335	52.9	13463-28-0;
neg_18934	Diglycolic Acid	metab_50161	B(ii)	0	40.8	HMDB0251283	M+Na-2H	C4H6O5	0.78668333333333	-	neg	154.99630227947	0.025750830095054	46.9	-
neg_18938	Udp-Galactose	metab_50165	B(i)	90.3	0	HMDB0000302	M-H	C15H24N2O17P2	0.78135	C00052	neg	565.04814321354	0.039925817576385	55.1	2956-16-3
neg_18943	Pentafluorobenzoyl Acetate	metab_50170	B(ii)	0	40.1	HMDB0256261	M-H2O-H	C9H3F5O3	0.78135	-	neg	234.98374665082	0.034374045519387	44.9	-
neg_18946	Panose	metab_50173	B(i)	80.7	0	HMDB0029937	M+Cl	C18H32O16	0.77605	C00713	neg	539.13906325966	0.019608763320598	52.3	33401-87-5
neg_18948	[2,3-Dihydroxy-3-(3-Methoxy-5-Oxo-2H-Furan-2-Yl)-1-Phenylpropyl] Acetate	metab_50175	B(i)	61.9	0	-	M+FA-H	C16H18O7	0.77605	-	neg	367.10508199473	0.059139054765146	48.7	-
neg_18986	6,8-Bis(Sulfanyl)Octanal	metab_50213	B(ii)	0	41	HMDB0258126	2M+FA-H	C8H16OS2	0.74923333333333	-	neg	429.12506947315	0.045847245888853	44.7	-
neg_19022	Nifedipine	metab_50249	B(ii)	0	40	HMDB0015247	2M+FA-H	C17H18N2O6	0.7333	C07266	neg	737.23621510556	0.022242565908946	45.1	21829-25-4
neg_19028	Sialyl Lex Tetra	metab_50255	B(ii)	0	65.6	HMDB0006627	M+Na-2H	C37H62N2O28	0.728	-	neg	1003.3173085052	0.10442124064493	49.1	140913-62-8
neg_19031	Lucuminoside	metab_50258	B(ii)	0	68.9	HMDB0029900	2M+FA-H	C19H25NO10	0.728	C08335	neg	899.28881934245	0.046020281998274	49.9	1392-28-5
neg_19041	Verbascose	metab_50268	B(i)	53.4	0	-;HMDB0259784	M-H	C30H52O26	0.7174	C08252;_	neg	827.26741823399	0.001700965375117	49.9	546-62-3;_
neg_19047	Rafabegron	metab_50274	B(ii)	0	84.6	HMDB0257097	M+Hac-H	C21H23ClN2O	0.71208333333333	-	neg	461.15123755818	0.085495653828123	47.9	-
neg_19049	Zalcitabine	metab_50276	B(ii)	0	62.3	HMDB0015078	M+FA-H	C9H13N3O3	0.71208333333333	C07207	neg	256.09392072166	0.092418173490664	50.4	7481-89-2
neg_19060	Histidine Glutamate	metab_50287	B(ii)	0	67.9	HMDB0253183	M-H	C11H16N4O6	0.70151666666667	-	neg	299.09845255888	0.022719116727978	51.6	-
neg_19064	Streptomycin Sulfate	metab_50291	B(ii)	0	84.9	HMDB0258510	M-H2O-H	C21H39N7O12	0.6962	-	neg	562.24792197405	0.015178778083747	55	-
neg_19072	Val-Asp	metab_50299	B(i)	88.5	0	HMDB0029123	M-H	C9H16N2O5	0.6962	-	neg	231.09869530014	0.062655852522989	56.5	20556-16-5
neg_19088	Glu-Ser-Thr	metab_50315	B(i)	69.1	0	-	M-H	C12H21N3O8	0.68558333333333	-	neg	334.12567228371	0.080353064778839	51.6	-
neg_19090	Ala-Glu-Ser	metab_50317	B(i)	58.2	0	-	M-H	C11H19N3O7	0.68558333333333	-	neg	304.11511203471	0.067130529229131	49.6	-
neg_19092	Ile-Lys	metab_50319	B(i)	94.4	0	HMDB0028912	M-H	C12H25N3O3	0.68558333333333	-	neg	258.18238741542	0.059067412771558	57.2	-
neg_19097	Stachyose	metab_50324	B(i)	51.4	0	HMDB0003553;PW_C001923	M-H	C24H42O21	0.6803	C01613	neg	665.21472511147	0.021779339484537	49.8	10094-58-3;470-55-3;470-55-3
neg_19100	Setipiprant	metab_50327	B(ii)	0	43.5	HMDB0258261	M+FA-H	C24H19FN2O3	0.6803	-	neg	447.1356705	0.05779537041124	45	-
neg_19104	Val-Asn	metab_50331	B(i)	94.8	0	HMDB0029122	M-H	C9H17N3O4	0.6803	-	neg	230.11472185841	0.033011991280215	58	66170-00-1
neg_19106	2-Aminoheptanedioic Acid	metab_50333	B(i)	91.7	0	HMDB0034252	M-H	C7H13NO4	0.6803	-	neg	174.077222	0.060591714657464	57.6	3721-85-5
neg_19107	Glycolaldehyde	metab_50334	B(ii)	0	47.8	HMDB0003344	M+FA-H	C2H4O2	0.6803	C00266	neg	105.01940242261	0.087939452627087	48.9	141-46-8
neg_19111	Cellulase	metab_50338	B(i)	93.1	0	-	M-H	C18H32O16	0.67503333333333	-	neg	503.16189732588	0.020559700044423	57.9	-
neg_19112	Suspensolide F	metab_50339	B(ii)	0	80.9	HMDB0031918	M-H	C21H34O12	0.67503333333333	C17429;_	neg	477.1949919	0.093787205267761	52.2	-;64703-86-2
neg_19117	Asp-Glu	metab_50344	B(i)	95.6	0	HMDB0028752	M-H	C9H14N2O7	0.67503333333333	-	neg	261.07299542599	0.064608732963802	57.6	-
neg_19127	Ile-Gly-His	metab_50354	B(i)	85.9	0	-	M-H	C14H23N5O4	0.66975	-	neg	324.16777082248	0.028049143341294	56	-
neg_19140	Gamma-Glutamylglutamine	metab_50367	B(i)	84.6	0	HMDB0011738	M-H	C10H17N3O6	0.66441666666667	C05283	neg	274.10457612161	0.08762661091603	55.4	10148-81-9
neg_19141	Thr-Ala	metab_50368	B(i)	50.6	0	HMDB0029054	M-H	C7H14N2O4	0.66441666666667	-	neg	189.08817728585	0.01152521477228	48.9	56217-50-6
neg_19146	Ile-Lys-His	metab_50373	B(i)	81.2	0	-	M-H	C18H32N6O4	0.65911666666667	-	neg	395.24124085609	0.072920152253875	53.8	-
neg_19147	Ser-Val-His	metab_50374	B(i)	68.1	0	-	M-H	C14H23N5O5	0.65911666666667	-	neg	340.16268066083	0.032034033478529	52.3	-
neg_19148	5-O-A-L-Arabinofuranosyl-L-Arabinose	metab_50375	B(ii)	0	63	HMDB0038880	M-H2O-H	C10H18O9	0.65911666666667	-	neg	263.07729772228	0.014580048922313	50.9	-
neg_19149	Imidazolelactic Acid	metab_50376	B(i)	98.7	0	HMDB0002320	M-H	C6H8N2O3	0.65911666666667	C05132	neg	155.04631786324	0.081215748787931	58.4	876-19-7
neg_19150	4-Oxobutanoic Acid	metab_50377	B(i)	69.1	0	HMDB0001259	M-H	C4H6O3	0.65911666666667	C00232	neg	101.02447077649	0.054647392205704	53.2	692-29-5
neg_19156	Arg-Glu-Asp	metab_50383	B(i)	61.7	0	-	M-H	C15H26N6O8	0.65378333333333	-	neg	417.17415872041	0.11379147350837	49.3	-
neg_19158	Fipexide	metab_50385	B(ii)	0	67.1	HMDB0252264	M-H	C20H21ClN2O	0.65378333333333	-	neg	387.11435520011	0.016577323544827	46	-
neg_19162	Ethyl Glucuronide	metab_50389	B(i)	87.3	0	HMDB0010325	M+Hac-H	C8H14O7	0.65378333333333	-	neg	281.0878166	0.017238658003124	56.5	17685-04-0
neg_19164	Asp-Ala	metab_50391	B(i)	69.7	0	HMDB0028746	M-H2O-H	C7H12N2O5	0.65378333333333	-	neg	185.05683134529	0.12224988674769	52.1	-
neg_19177	Rosarin	metab_50404	B(i)	41.5	0	-	M+FA-H	C20H28O10	0.6432	-	neg	473.1624982	0.042143944552238	42	-
neg_19179	D-1-[(3-Carboxypropyl)Amino]-1-Deoxyfructose	metab_50406	B(ii)	0	60	HMDB0038663	M-H	C10H19NO7	0.6432	-	neg	264.10896409799	0.082282872338742	49.5	10003-63-1
neg_19187	Gly-Glu	metab_50414	B(i)	77.7	0	HMDB0028840	M-H	C7H12N2O5	0.6379	-	neg	203.0674407	0.032371394405782	54.5	-

neg_19188	Isoscoparin 2"-(6-(E)-Ferulylglucoside)	metab_50415	B(ii)	0	62.8	HMDB0038454	M-H2O-H	C38H40O19	0.63258333333333	-	neg	781.20180166631	0.13086333596492	46.2	97605-26-0;
neg_19189	10-Edam	metab_50416	B(ii)	0	45.5	HMDB0244267	M+Na-2H	C22H25N7O5	0.63258333333333	-	neg	488.16235017304	0.035966182622207	44.2	-
neg_19190	Ser-Ser-Thr	metab_50417	B(i)	79.3	0	-	M-H	C10H19N3O7	0.63258333333333	-	neg	292.11514237153	0.089427840297645	53.2	-
neg_19192	2-Keto-3-Deoxy-D-Gluconic Acid	metab_50419	B(i)	70	0	HMDB0001353;LMFA01050486	M+Hac-H	C6H10O6	0.63258333333333	C01216	neg	237.06163967961	0.084929194176917	52.5	17510-99-5;
neg_19193	Ser-Ala	metab_50420	B(i)	86.9	0	HMDB0029032	M-H	C6H12N2O4	0.63258333333333	-	neg	175.07252542284	0.016972377522984	56.2	6403-17-4
neg_19194	4-Ethoxy-4-Oxobut-2-Enoic Acid	metab_50421	B(i)	39.7	0	HMDB0246416	M-H	C6H8O4	0.63258333333333	-	neg	143.0350786	0.036751941246539	47.3	-
neg_19198	D-Glycero-L-Galacto-Octulose	metab_50425	B(ii)	0	60.4	HMDB0029954	M-H	C8H16O8	0.6273	-	neg	239.07727937039	0.082091774543184	50	-
neg_19201	D-Erythrose	metab_50428	B(i)	81.6	0	HMDB0002649;HMDB0250746	M-H	C4H8O4	0.6273	_;C01796	neg	119.03505554196	0.035140885432116	55.9	583-50-6;_
neg_19203	Glyceraldehyde	metab_50430	B(i)	87.3	0	HMDB0001051;PW_C000828	M-H	C3H6O3	0.6273	C02154	neg	89.024459464588	0.026380788547759	57.2	367-47-5;56-82-6
neg_19204	Verlukast	metab_50431	B(ii)	0	53	HMDB0254776	M+FA-H	C26H27ClN2O3S2	0.62195	_;C11239	neg	559.11521653357	0.29661808325464	40.5	115104-28-4;_
neg_19208	Galactaric Acid	metab_50435	B(i)	88.2	0	HMDB0000639;LMFA01170107	M-H	C6H10O8	0.62195	C00879;_	neg	209.03029757204	0.1334497799568	56	526-99-8;
neg_19209	Hexuronic Acid	metab_50436	B(i)	96.3	0	-	M-H	C6H10O7	0.62195	-	neg	193.03545622574	0.005306128619536	58.4	-
neg_19220	Gly-Ala-Gln	metab_50447	B(i)	73.2	0	-	M-H	C10H18N4O5	0.6113	-	neg	273.12046266445	0.017625148408391	53.4	-
neg_19232	Lactosamine	metab_50459	B(ii)	0	59.6	HMDB0006591	M+Cl	C12H23NO10	0.58985	C00203	neg	376.10144779837	0.084910596090022	49	13000-25-4
neg_19234	Gln-Ser-Asn	metab_50461	B(i)	85.8	0	-	M-H	C12H21N5O7	0.58985	-	neg	346.13696547818	0.036231497467981	54.6	-
neg_19241	Asp-Asn	metab_50468	B(i)	57.6	0	HMDB0028748	M-H	C8H13N3O6	0.58985	-	neg	246.07324670601	0.077461831093864	50	-
neg_19243	Ala-Gly-Ser	metab_50470	B(i)	65.8	0	-	M-H	C8H15N3O5	0.58985	-	neg	232.09390624398	0.016184107446357	52.3	-
neg_19244	Asp-Ser	metab_50471	B(i)	58.1	0	HMDB0028762	M-H	C7H12N2O6	0.58985	-	neg	219.06238807376	0.022453764742781	50.2	-
neg_19245	Ser-Thr	metab_50472	B(i)	92.4	0	HMDB0029049	M-H	C7H14N2O5	0.58985	-	neg	205.0831516	0.050079633171136	56.8	61043-85-4
neg_19247	L-Asparagine	metab_50474	B(i)	90.5	0	HMDB0000168;PW_C000110	M-H	C4H8N2O3	0.58985	C00152	neg	131.0463023	0.019928017646082	57.3	70-47-3
neg_19249	Penicillin V	metab_50476	B(ii)	0	52.8	HMDB0014561	2M+FA-H	C16H18N2O5S	0.58456666666667	C08126	neg	745.18102097752	0.046468438608828	48.1	1987/8/1;87-08-1
neg_19256	Malvidin 3-(6"-Acetyl-Galactoside)	metab_50483	B(ii)	0	56	HMDB0301662	M-H2O-H	C25H27O13+	0.58456666666667	-	neg	516.12518807638	0.087706192504309	46.5	75-07-0
neg_19259	4-((2-Nitro-1H-Imidazol-1-Yl)Methoxy)-2-Buten-1-Ol	metab_50486	B(ii)	0	49.2	HMDB0257251	2M+FA-H	C8H11N3O4	0.58456666666667	-	neg	471.15142728446	0.03632699326217	47.1	-
neg_19261	Trehalose 6-Phosphate	metab_50488	B(i)	64.7	0	HMDB0001124	M-H	C12H23O14P	0.58456666666667	C00689	neg	421.07535911633	0.027952850379595	51.3	4484-88-2
neg_19263	Befloxatone	metab_50490	B(ii)	0	68.4	HMDB0248929	M+FA-H	C15H18F3NO5	0.58456666666667	-	neg	394.11203908102	0.13683565727786	50.7	-
neg_19275	L-Saccharopine	metab_50502	B(i)	97.6	0	HMDB0000279	M-H	C11H20N2O6	0.58456666666667	C00449	neg	275.12498455706	0.030322212180831	57.8	997-68-2
neg_19277	Fructose-6-Phosphate	metab_50504	B(i)	71.1	0	HMDB0000124	M-H	C6H13O9P	0.58456666666667	C00085	neg	259.0224722	0.012701335753269	54	643-13-0
neg_19281	D-Ribose 5-Phosphate	metab_50508	B(i)	94	0	-;HMDB0001548;PW_C001191	M-H	C5H11O8P	0.58456666666667	C00117;C03736	neg	229.0120522	0.11477353060343	58.2	4300-28-1;34980-65-9
neg_19286	L-Aspartic Acid	metab_50513	B(i)	98.7	0	HMDB0000191;PW_C000123	M-H	C4H7NO4	0.58456666666667	C00049	neg	132.03031912769	0.051583763567067	59.2	56-84-8
neg_19318	Glycerophosphoinositol	metab_50545	B(ii)	0	71.9	HMDB0011649	M-H	C9H19O11P	0.5793	C01225	neg	333.05930455353	0.018052297356243	53.8	129830-95-1
neg_19331	Gly-Asn	metab_50558	B(i)	86.1	0	HMDB0028836	M-H	C6H11N3O4	0.5793	-	neg	188.06771873147	0.023600191974308	56.2	-
neg_19374	L-Serine	metab_50601	B(i)	99.4	0	HMDB0000187;PW_C000120	M-H	C3H7NO3	0.574	C00065	neg	104.03539633472	0.032869752695091	59.1	56-45-1
neg_19398	5-Methylcytidine	metab_50625	B(ii)	0	54.8	HMDB0000982	M+Hac-H	C10H15N3O5	0.5687	-	neg	316.11481199818	0.069522518761399	49.4	2140-61-6;_
neg_19400	Arg-Glu	metab_50627	B(i)	64.8	0	HMDB0028708	M-H	C11H21N5O5	0.5687	-	neg	302.14687856422	0.055192726265736	51	15706-89-5
neg_19420	Neocarthamin	metab_50647	B(ii)	0	42.8	HMDB0037476	M+Na-2H	C21H22O11	0.56343333333333	C17675;_	neg	471.09064605127	0.027206400677281	46.2	-;_
neg_19430	Chloramphenicol	metab_50657	B(ii)	0	58.5	HMDB0014589	M+Na-2H	C11H12Cl2N2O5	0.56343333333333	C00918	neg	342.98399083984	0.036954074757594	40.8	56-75-7
neg_19432	Ala-Gly-His	metab_50659	B(i)	59	0	-	M-H	C11H17N5O4	0.56343333333333	-	neg	282.12096986171	0.023769986078194	46.3	-
neg_19433	Arg-Thr	metab_50660	B(i)	69	0	HMDB0028719	M-H	C10H21N5O4	0.56343333333333	-	neg	274.15204154841	0.008709326008098	52.4	106326-78-7
neg_19450	(24E)-3Alpha,15Alpha-Diacetoxy-23-Oxo-7,9(11),24-Lanostatrien-26-Oic Acid	metab_50677	B(ii)	0	52.3	HMDB0035388	M-H	C34H48O7	0.55811666666667	-	neg	567.33356008079	0.041249889600008	46.6	117383-36-5
neg_19471	His-Ala-Ser	metab_50698	B(i)	79	0	-	M-H	C12H19N5O5	0.55811666666667	-	neg	312.13101088001	0.032645211086247	54.8	-
neg_19472	Gly-Ala-Arg	metab_50699	B(i)	76.9	0	-	M-H	C11H22N6O4	0.55811666666667	-	neg	301.16321571454	0.045623009054492	53.5	-
neg_19474	Arg-Ala	metab_50701	B(i)	85.5	0	HMDB0028702	M-H	C9H19N5O3	0.55811666666667	-	neg	244.14162588982	0.008247311312915	55.2	40968-45-4

neg_19476	Dl-Arginine	metab_50703	B(ii)	0	81.8	HMDB0251511	M-H	C6H14N4O2	0.55811666666667	C02385	neg	173.10446453837	0.008571303498731	54.8	-
neg_19544	Gly-Arg	metab_50771	B(i)	82.5	0	HMDB0028835	M-H	C8H17N5O3	0.5528	-	neg	230.12600042482	0.016205509868932	51.2	-
neg_19574	Fructose-Lysine	metab_50801	B(ii)	0	61.8	HMDB0252494	M-H	C12H24N2O7	0.54751666666667	-	neg	307.15125721517	0.013540516617551	51	-
neg_19617	Ser-Gln-Lys	metab_50844	B(i)	64	0	-	M-H	C14H27N5O6	0.53688333333333	-	neg	360.18908281074	0.038573045009289	51.1	-
neg_19626	Ala-Lys	metab_50853	B(i)	91.6	0	HMDB0028692	M-H	C9H19N3O3	0.53688333333333	-	neg	216.13548525823	0.009710711924059	57.5	6366-77-4
neg_19645	Gly-Lys	metab_50872	B(i)	89.4	0	HMDB0028846	M-H	C8H17N3O3	0.53158333333333	-	neg	202.11984711501	0.055190798700131	56.7	-
neg_19646	Dulcoside A	metab_50873	B(ii)	0	55.7	HMDB0036708	M-H2O-H	C38H60O17	0.52628333333333	-	neg	769.35992873171	0.09580114798332	46.2	64432-06-0;
neg_19689	Arginylarginine	metab_50916	B(ii)	0	58.3	HMDB0028703	M-H	C12H26N8O3	0.5157	-	neg	329.20566870804	0.069084421137759	49.7	15483-27-9
neg_19758	Arg-Lys	metab_50985	B(i)	87.1	0	HMDB0028714	M-H	C12H26N6O3	0.49445	-	neg	301.19949900936	0.087074853268016	55.5	40968-46-5
neg_19767	Edoxaban	metab_50994	B(i)	47.2	0	HMDB0251704	M+FA-H	C24H30ClN7O4S	0.4891	-	neg	592.17113015667	0.11928683672269	43.9	-
neg_19801	Gln-His-Lys	metab_51028	B(i)	54.6	0	-	M-H	C17H29N7O5	0.47323333333333	-	neg	410.21587259142	0.007901600060205	49.8	-
neg_19827	Lys-Lys	metab_51054	B(i)	69.9	0	HMDB0028956	M-H	C12H26N4O3	0.4626	-	neg	273.19332837061	0.073756561410774	52	13184-13-9
neg_19851	3,5-Dimethyl-4-(Methylsulfonyl)-Phenol	metab_51078	B(i)	46	0	-	M+Hac-H	C9H12O3S	0.44138333333333	-	neg	259.06314620886	0.091699577238702	45.4	-
neg_19879	Neoacrimarine B	metab_51106	B(ii)	0	59.1	HMDB0040385	M+Na-2H, M+FA-H,	C39H41NO9	3.1965	-	neg	726.29538624184	0.037411012646947	48.5	149301-47-3
neg_19899	Hovenine A	metab_51126	B(ii)	0	41.9	HMDB0030200	M+Cl, M+FA-H	C27H42N4O4	3.6071333333333	-	neg	531.31495760155	0.035171527831021	45.6	52309-78-1
neg_19903	23-Trans-P-Coumaroyloxytormentic Acid	metab_51130	B(ii)	0	62.1	HMDB0040682	M+Na-2H, M+Cl	C39H54O8	4.16245	-	neg	685.35309677092	0.014751100366148	48.6	144604-14-8
neg_19905	Tamoxifen-Ol	metab_51132	B(ii)	0	74.1	HMDB0258707	2M-H, 2M+FA-H	C24H24O2	4.44525	-	neg	687.34623002255	0.18039789499059	53.4	-
neg_19909	1,2-Dihexadecanoyl-Sn-Glycero-3-Phospho-(1D-Myo-Inositol-3,4-Bisphosphate)	metab_51136	B(ii)	0	70.2	HMDB0250777	M-H2O-H, M+Cl	C41H81O19P3	4.87215	-	neg	951.43451155877	0.029104629463614	50.8	-
neg_19915	Smgdg(O-24:5/3:0)	metab_51142	B(i)	61.8	0	-	M+Cl, M+FA-H	C36H60O12S	5.2449	-	neg	761.38418520564	0.059680391805756	48.3	-
neg_19917	Janthitrem B	metab_51144	B(ii)	0	78.5	HMDB0030528	M+Cl, M+FA-H	C37H47NO5	5.4367	C20600;_	neg	630.34789717892	0.07137561067515	52.1	73561-90-7
neg_19923	Jesaconitine	metab_51150	B(ii)	0	65.6	HMDB0253732	M-H2O-H, M+FA-H	C35H49NO12	5.8422166666667	C08692;_	neg	656.30530253182	0.018644474845688	51.4	16298-90-1;_
neg_19939	Pip(5-Iso Pgf2Vi/20:4(8Z,11Z,14Z,17Z))	metab_51166	B(ii)	0	43.8	HMDB0280121	M-H2O-H, M+Na-2H	C47H78O19P2	5.5970166666667	-	neg	989.44691108369	0.0067122220091877	45.3	-
neg_19949	Pgp(6 Keto-Pgf1Alpha/16:1(9Z))	metab_51176	B(ii)	0	49.2	HMDB0272425	M-H2O-H, M+Hac-H	C42H76O17P2	4.7549	-	neg	973.46960509091	0.042753297064585	47.5	-
neg_19954	Lucyoside M	metab_51181	B(ii)	0	75.5	HMDB0034546	M-H2O-H, M+Cl	C44H68O15	4.5415666666667	-	neg	871.42678976289	0.019337630739052	52.5	100156-32-9
neg_19955	N-Formyl-Nle-Leu-Phe-Nle-Tyr-Lys	metab_51182	B(ii)	0	68.2	HMDB0247317	M+Na-2H, M+Cl	C43H65N7O9	4.5415666666667	-	neg	844.45314820341	0.029068532360415	49	-
neg_19956	Dihydroergocristine	metab_51183	B(ii)	0	54.4	HMDB0251309	M+Cl, M+FA-H	C35H41N5O5	4.4719666666667	-	neg	646.28463191475	0.019130736043598	46.9	-
neg_19959	Ps(2:0/18:5)	metab_51186	B(i)	63.4	0	-	M+Na-2H, M+FA-H	C26H40NO10P	4.30135	-	neg	602.24090979973	0.052032370147703	46.1	-
neg_19962	Pe(Dime(13,5)/Pgj2)	metab_51189	B(ii)	0	67.9	HMDB0284890	M+Na-2H, M+Cl	C49H82NO11P	3.8316	-	neg	926.53171150199	0.007327072227923	51.2	-
neg_19974	Plumieride	metab_51201	B(ii)	0	44.1	HMDB0256647	2M-H, 2M+FA-H	C21H26O12	0.98371666666667	_;C09797	neg	985.28613793503	0.020534504237786	45.8	511-89-7;_
neg_19980	Hydroxymethylbilane	metab_51207	B(ii)	0	58.5	HMDB0001137	M+Na-2H, M+FA-H	C40H46N4O17	1.1166833333333	C01024	neg	899.28909449371	0.010935292335286	47.7	71861-60-4;73023-76-4
neg_19982	Fenpiclonil	metab_51209	B(i)	41.1	0	-	M+FA-H, 2M-H	C11H6Cl2N2	1.1644666666667	C14268	neg	280.9892288	0.03637953689644	38.6	74738-17-3

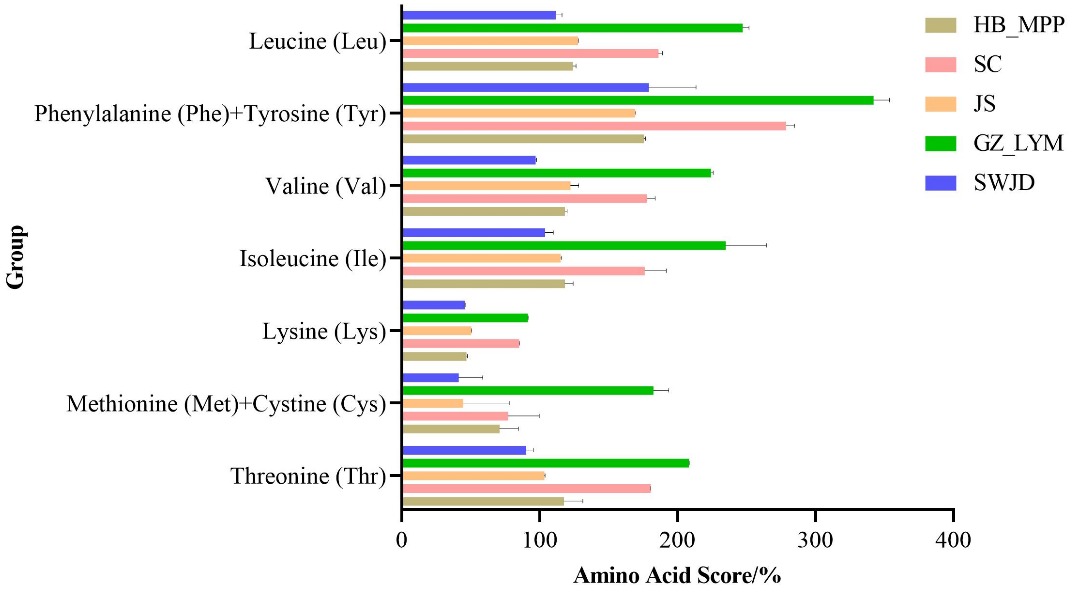

Fig. S1 Amino Acid Score Chart

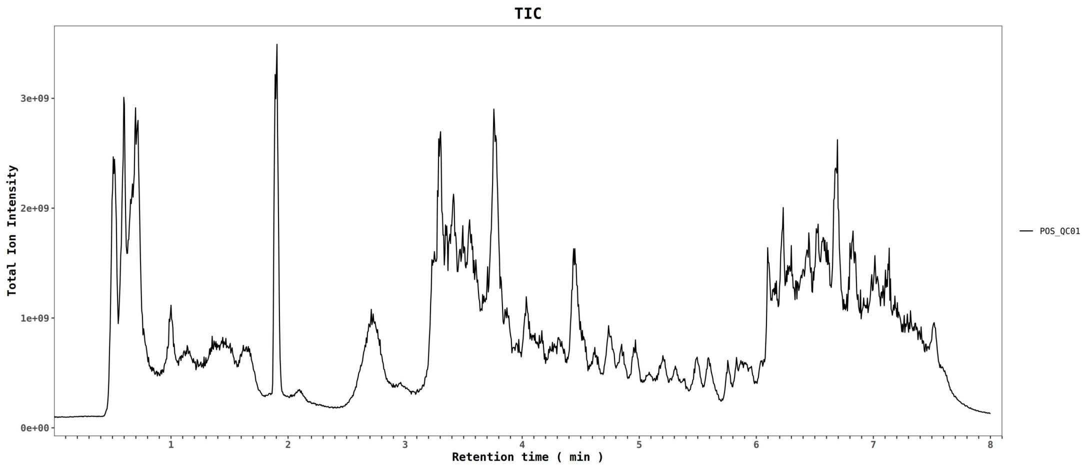

Fig. S2 QC validation results (TIC overlays,Positive ion mode)

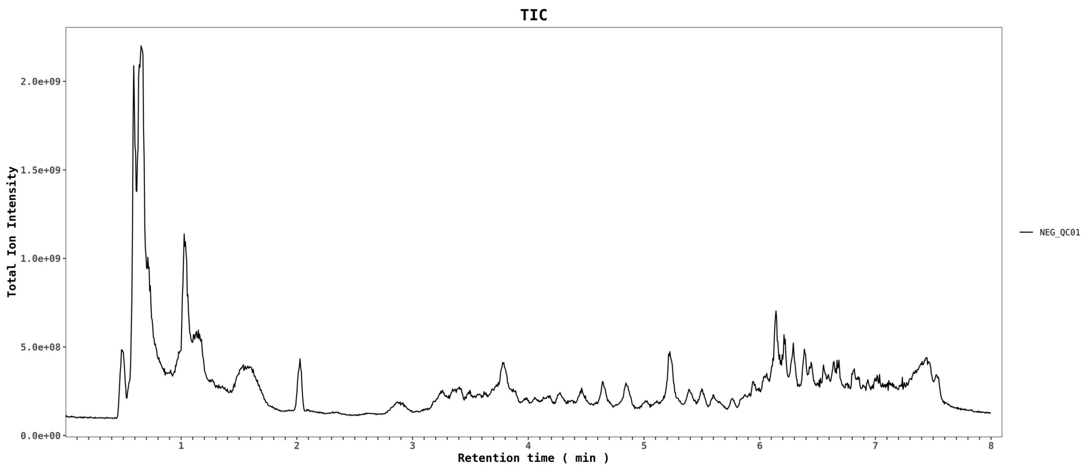

Fig. S3 QC validation results (TIC overlays,Negative ion mode)

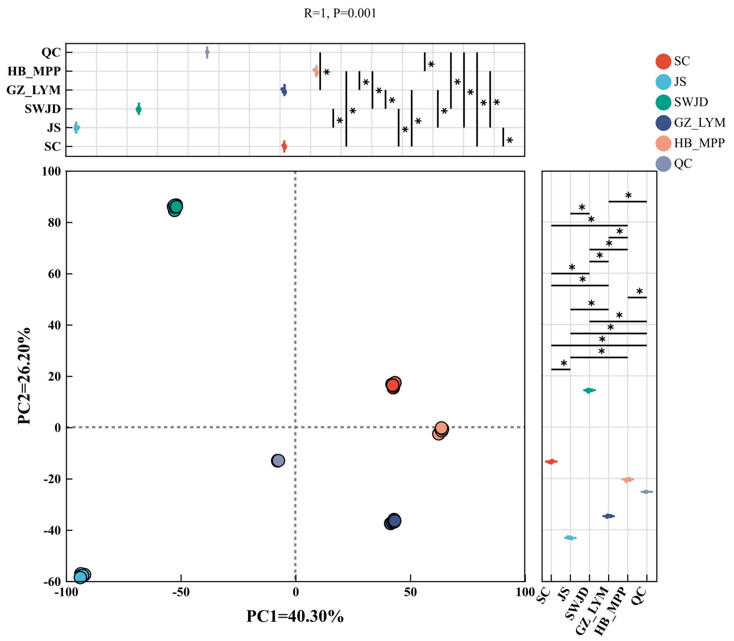

Fig. S4 QC validation results (PCA showing QC clusterings,Positive ion mode)

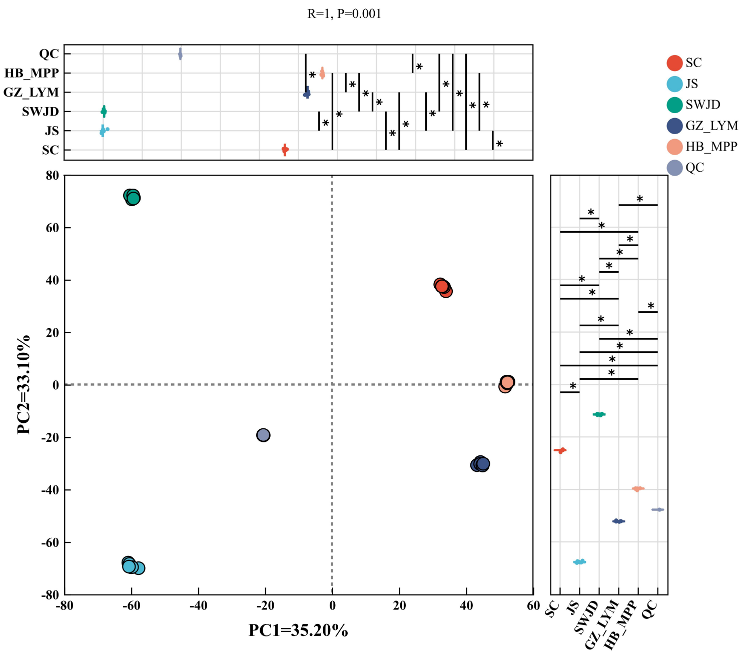

Fig. S5 QC validation results (PCA showing QC clusterings,Negative ion mode)

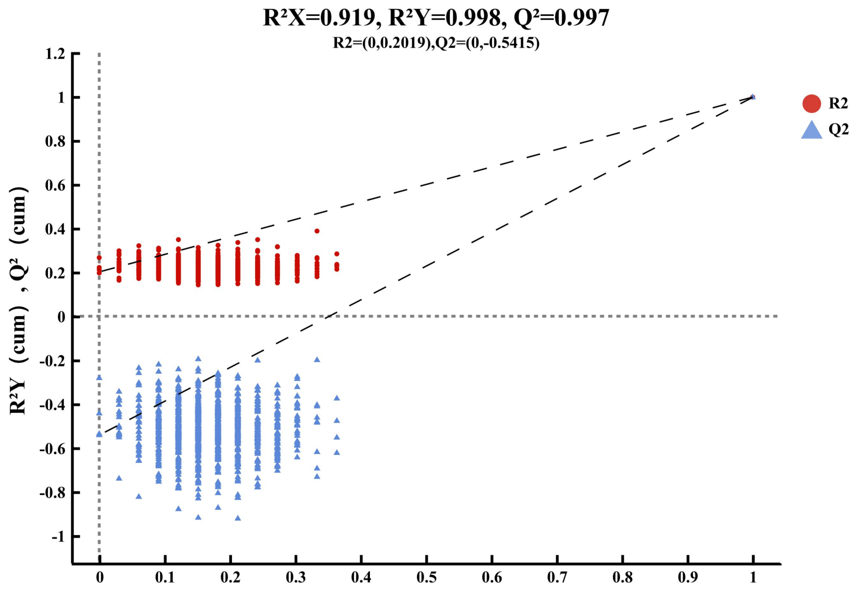

Fig. S6 Positive ion mode OPLS-DA permutation test (1000 permutations)

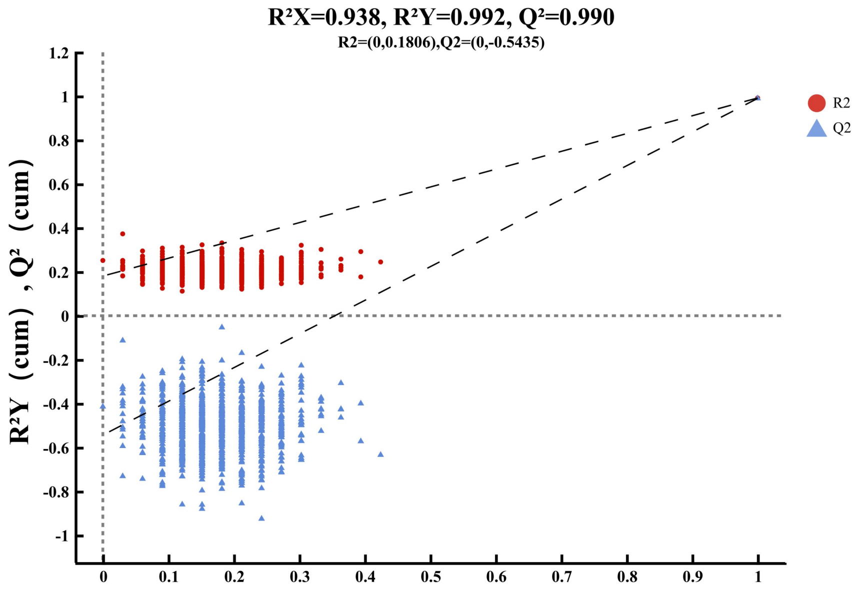

Fig. S7 Negative ion mode OPLS-DA permutation test (1000 permutations)

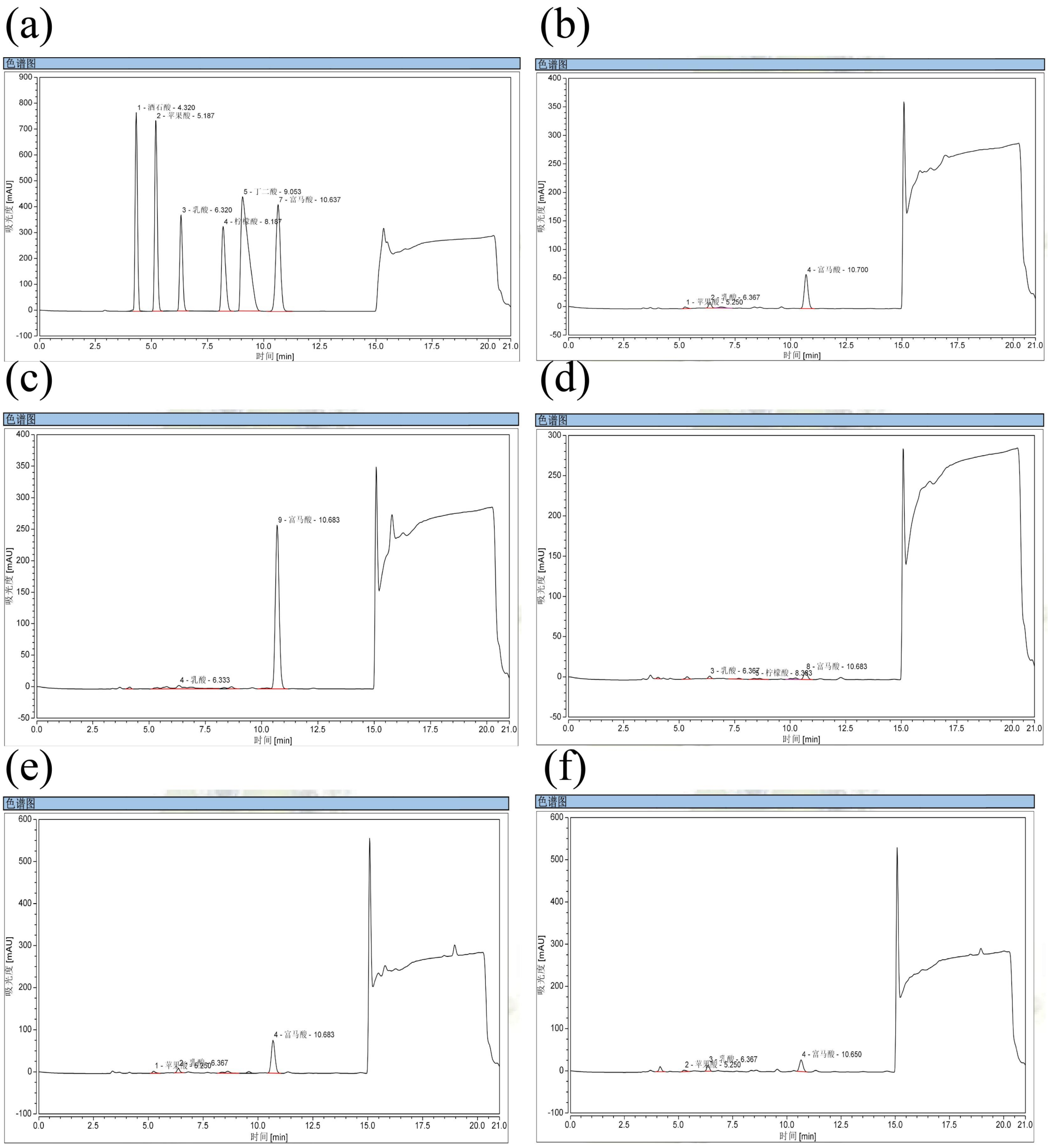

Fig. S8 HPLC chromatogram of organic acid. (a) HPLC chromatogram of organic acid standards. (b) HPLC chromatogram of organic acids in HB_MPP. (c) HPLC chromatogram of organic acids in SC. (d) HPLC chromatogram of organic acids in JS. (e) HPLC chromatogram of organic acids in GZ_LYM. (f) HPLC chromatogram of organic acids in SWJD.(酒石酸：Tartaric acid;苹果酸：Malic acid;乳酸：Lactic acid;柠檬酸：Citric acid;丁二酸：Succinic acid;富马酸：Fumaric acid)

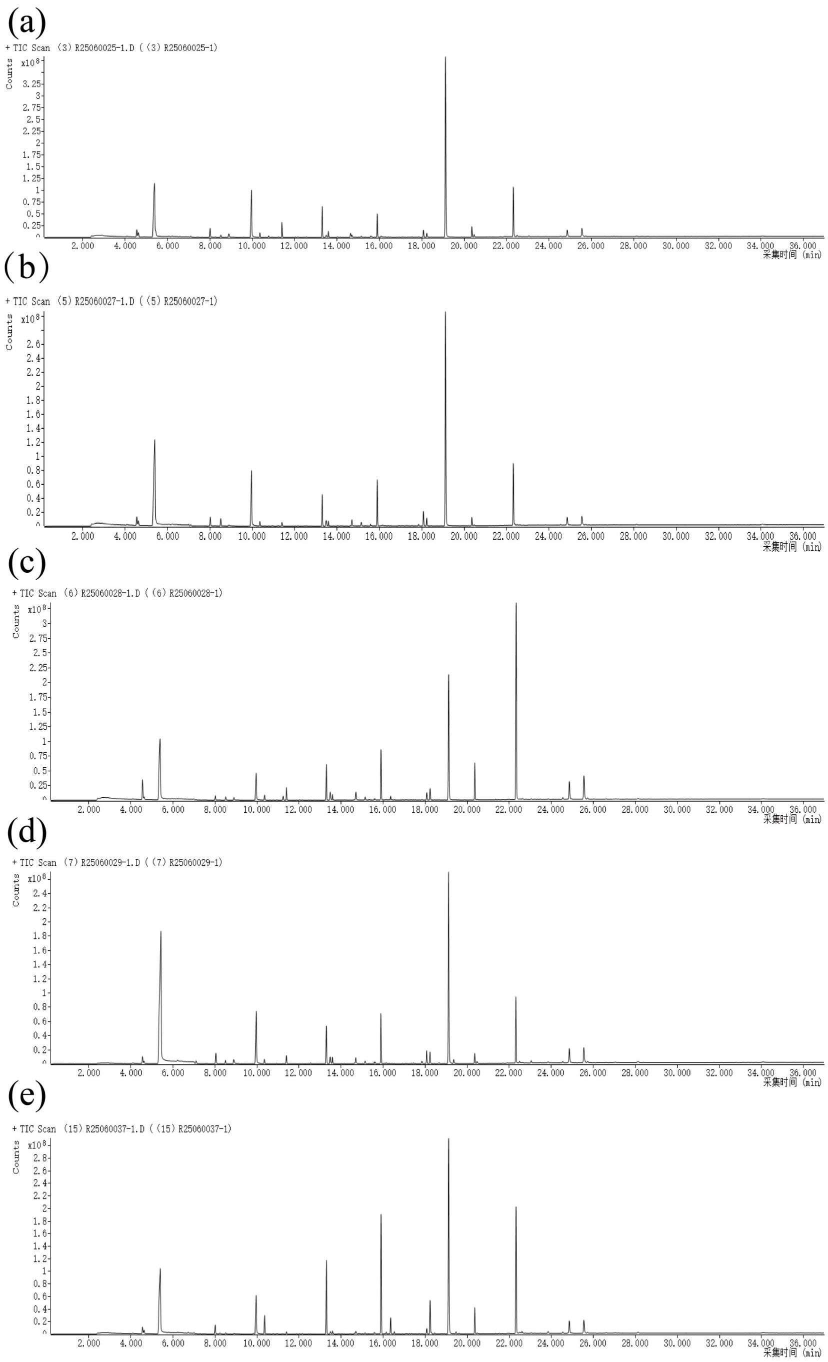

Fig. S9 Total Ion Chromatogram (TIC)(a,HB_MPP;b,SC;c,JS;d:GZ_LYM;e,SWJD)

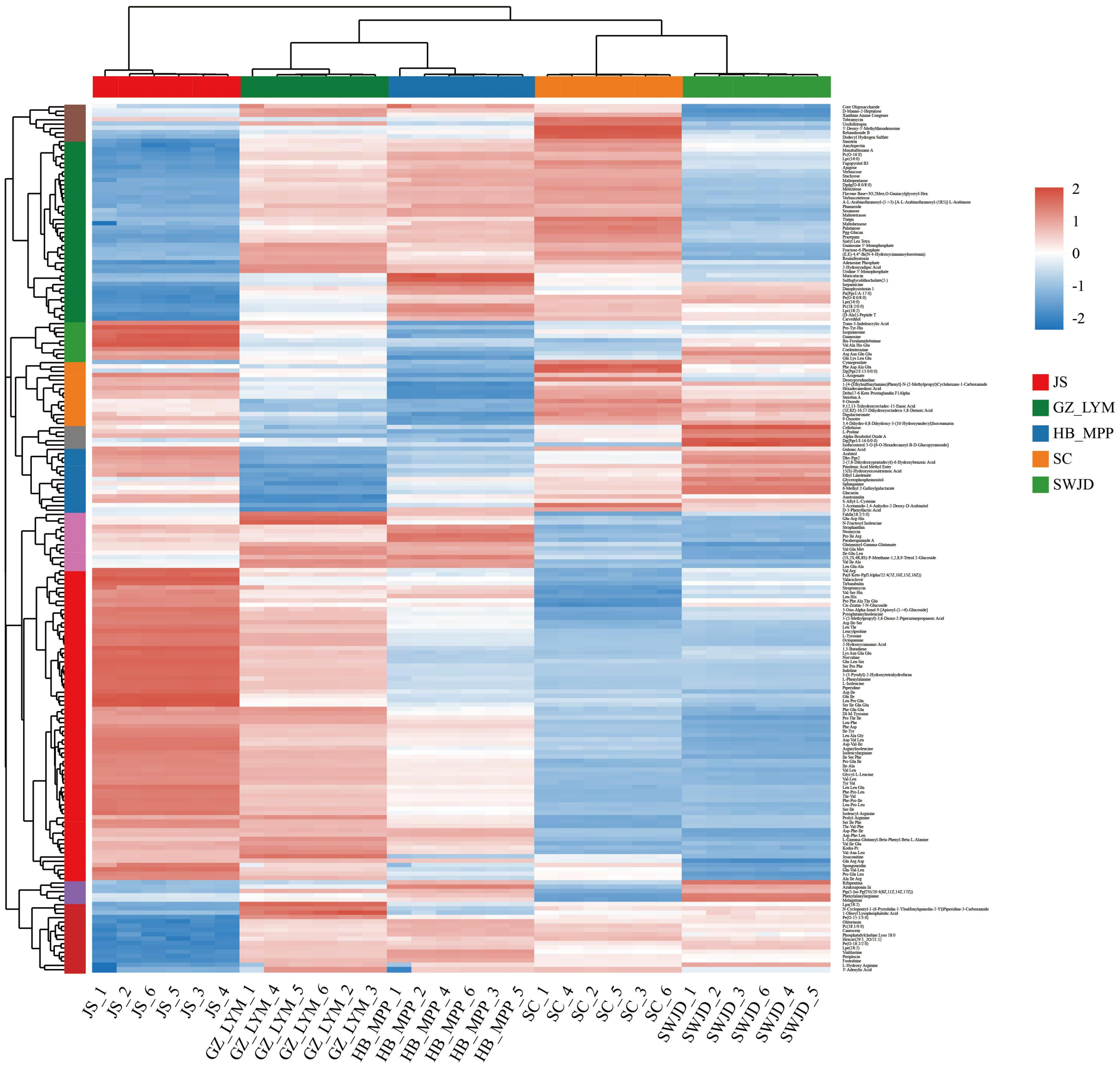

Fig. S10 Full-size heatmap
